# Synthesis of Boron-Containing Nucleoside Analogs

**DOI:** 10.1021/acs.joc.3c02179

**Published:** 2024-01-16

**Authors:** Latifah M. Alhthlol, Christopher L. Orme, Ben S. Jefferis, Sarah A. Herter, Halee E. Kemper, John W. Tomsho

**Affiliations:** † Department of Chemistry & Biochemistry, St Joseph’s University, University City Campus, 600 South 43rd Street, Philadelphia, Pennsylvania 19104, United States; ‡ Department of Chemistry, King Saud bin Abdulaziz University for Health Sciences, Al Mubarraz, Alahsa 36428, Saudi Arabia

## Abstract

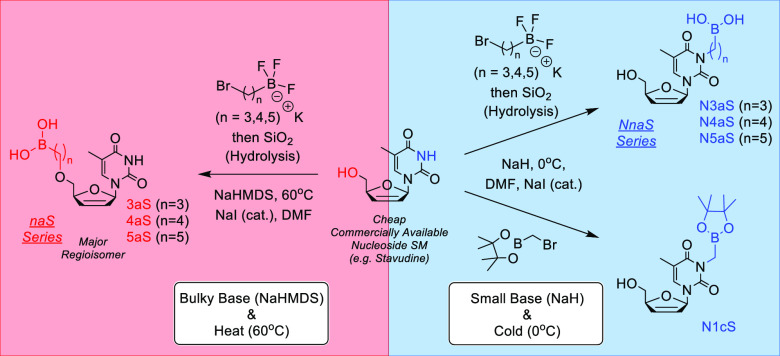

Over the last century, nucleoside-based therapeutics have demonstrated remarkable effectiveness in the treatment of a wide variety of diseases from cancer to HIV. In addition, boron-containing drugs have recently emerged as an exciting and fruitful avenue for medicinal therapies. However, borononucleosides have largely been unexplored in the context of medicinal applications. Herein, we report the synthesis, isolation, and characterization of two novel boron-containing nucleoside compound libraries which may find utility as therapeutic agents. Our synthetic strategy employs efficient one-step substitution reactions between a diverse variety of nucleoside scaffolds and an assortment of *n*-alkyl potassium trifluoroborate-containing electrophiles. We demonstrated that these alkylation reactions are compatible with cyclic and acyclic nucleoside substrates, as well as increasing alkyl chain lengths. Furthermore, regioselective control of product formation can be readily achieved through manipulation of base identity and reaction temperature conditions.

## Introduction

**Figure 1:**
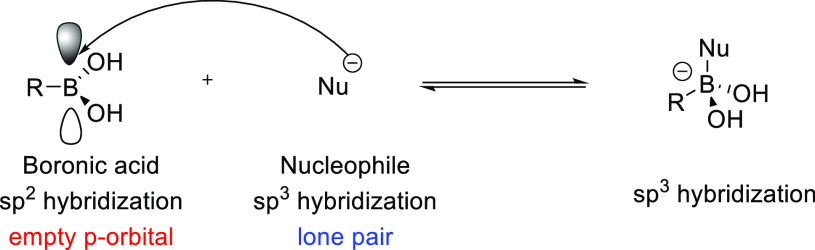
Conversion of boronic acid from a neutral, trigonal planar species to an anionic, tetrahedral species.

Boron-containing drugs represent an exciting, new avenue for potential medicinal applications.^1−6^ Boron has unique chemical characteristics; it does not follow the typical electron octet rule and can form three covalent bonds while retaining a reactive vacant p-orbital center. This distinct property gives boronic acids and their derivatives strong Lewis acid characteristics, giving rise to interesting and exceptional chemistry.^7^ Furthermore, this allows the boron center to convert between two molecular geometries; an uncharged, trigonal planar form and an anionic, tetrahedral state with an electron-rich ligand coordinated to the vacant p-orbital (Figure 1).^8^

**Figure 2:**
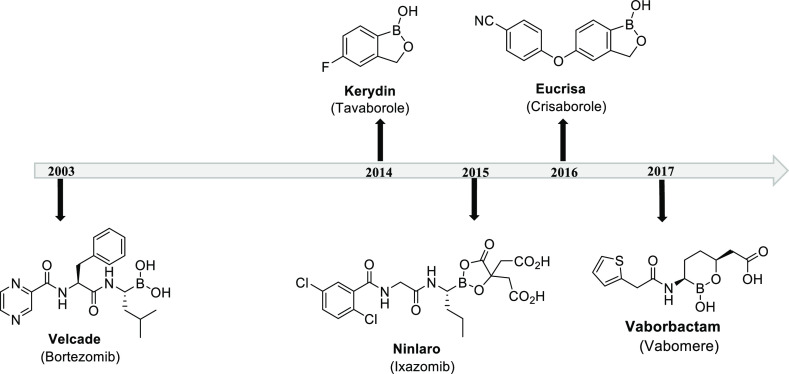
Current FDA-approved boron-containing drugs.

Currently, there are five FDA-approved drugs on the market containing a boron atom center which is critical to therapeutic activity (Figure 2). These drugs include: Velcade, a peptidyl boronic acid treatment for multiple myeloma;^9^ Kerydin, an oxaborole-containing antifungal agent; Ninlaro, a reversible proteasome inhibitor for the treatment of multiple myeloma;^10^ Eucrisa, a nonsteroidal topical medication used for the treatment of eczema;^11^ and Vaborbactam, a β-lactamase inhibitor used for the treatment of urinary tract infections.^12^

Velcade and Ninlaro are alkyl boronic acids that form strong covalent adducts with threonine residues in the active site of proteasomes,^5^ whereas Eucrisa is a benzoxaborole inhibitor that chelates to metal centers in the enzyme active site.^11,13^ These diverse and unique interactions of boron drugs with their biological targets have significantly increased research efforts to further the use of boronic acids in medicinal chemistry. Additionally, with the FDA approval of these drugs largely alleviating toxicity concerns associated with boron, boron-containing compounds show great promise as a new frontier for therapeutic discovery.^3−6^

**Figure 3:**
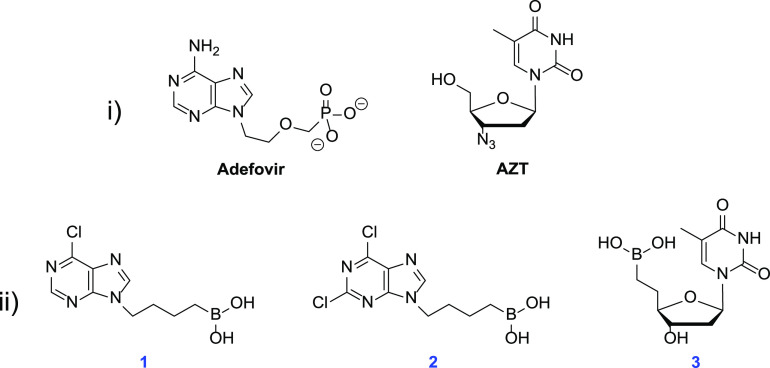
(i) Example of FDA-approved nucleoside analogs; anti-HIV and Hepatitis B drugs, including cyclic azidothymidine (AZT) and acyclic adefovir, respectively. (ii) Two acyclic borononucleoside analogs previously reported to exhibit anti-HIV properties (**1** and **2**). Thymidine borononucleoside analogue **3** behaves as a weak substrate toward human TMP kinase.

Nucleoside drugs have long been effective therapeutic agents for the treatment of a number of diseases.^14,15^ For example, nucleoside analogs such as azidothymidine (AZT) and adefovir have been powerful drugs for the treatment of HIV and Hepatitis B, respectively (Figure 3).^16^ In 1996, Chen and co-workers reported the first study of boronic acids being used as nucleoside analogs for therapeutic applications.^17^ The group synthesized a series of novel acyclic nucleoside boronic acid derivatives containing either a pyrimidine or purine base. These compounds were evaluated for their anti-HIV activity and found a few nucleoside analogs capable of exhibiting mild activity *in vitro*. They obtained EC_50_ values in the low micromolar range for two acyclic borononucleoside compounds: 6-chloro-9-(4-dihydroxyborylbutyl) purine **1** and 2,6-dichloro-9-(4-dihydroxyborylbutyl) purine **2** (Figure 3). These compounds exhibited EC_50_ values for anti-HIV plaque reduction assay of 7.7 ± 1.5 μM and 0.99 ± 0.01 μM, for **1** and **2**, respectively. However, they also observed significant cytotoxicity, with IC_50_ values for CEM-SS cell lines of 43 ± 31.1 μM and 4.9 ± 2.2 μM, for **1** and **2**, respectively. It was noted that they did not investigate the mechanism of action for their anti-HIV agents.

More recently in 2012, El Amri et al. synthesized and evaluated various borononucleoside analogs as 5′-monophosphate isosteres for human NMP kinases.^18^ The group screened eight borononucleoside compounds as substrates toward the five human NMP kinases: hAMPK, hCMPK, hTMPK, hUMPK, and hGMP. They observed that only one of the borononucleoside analogs, **3**, acted as a weak substrate toward human thymidylate monophosphate kinase (TMP) (Figure 3). This result suggests that some borononucleosides can act as substrates for selected human NMP kinases.

These limited works largely represent the extent of research conducted toward synthesizing boronic acid-containing nucleosides for potential drug applications. In considering the above work by Chen et al. and El Amri et al., the synthesized compounds placed a boronic acid moiety near where one would find the phosphate group in a nucleoside monophosphate. The idea that boric acid and boronic acids may act as phosphate bioisosteres has previously been put forward.^19−21^ To this end, we set out to expand the portfolio of boron-containing nucleosides and develop synthetically rigorous methodology for the formation of nucleoside alkylated boronic acid compounds.

## Results and Discussion

**Scheme 1:**
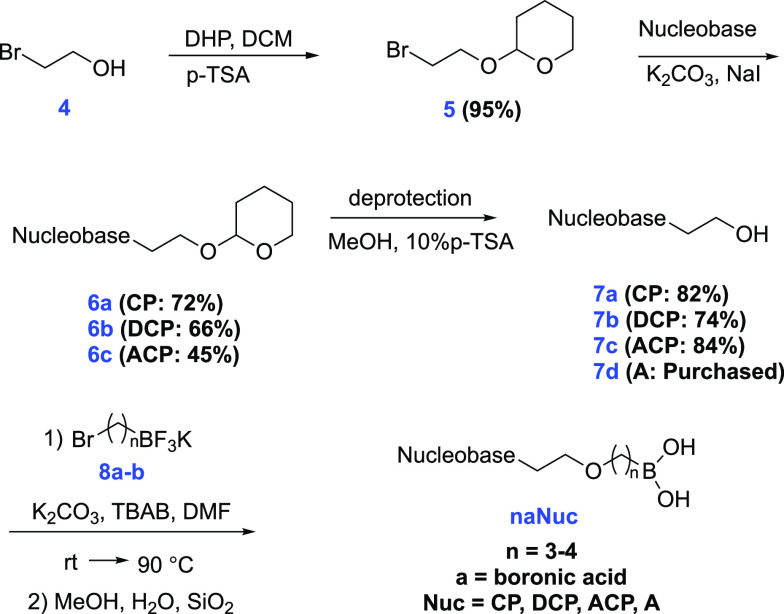
Synthesis of Boronic Acid-Adefovir Analogs(**naNuc**) Compound identification key: **n** = alkyl chain length, **a** = boronic acid, **Nuc** (nucleobase) = CP (6-Chloropurine); DCP (2,6-Dichloropurine); ACP (2-Amino-6-chloropurine); or A (Adenine). Compound **7d** (2-(6-aminopurin-9-yl)ethanol) is commercially available.

First, we looked to expand the structural diversity present in Chen’s borononucleoside compounds by incorporation of an ether linkage similar to that found in adefovir (Figure 3). These adefovir-boronic acid nucleoside analogs (**naNuc**) were synthesized with different nucleobases (6-chloropurine, 2,6-dichloropurine, 2-amino-6-chloropurine, and adenine) and varied alkyl chain linkers (linker length 3–4 between the oxygen and boronic acid warhead). The synthesis of the **naNuc** series began with protecting the hydroxyl group on 2-bromoethanol (**4**) as a tetrahydropyranyl (THP) ether to give **5** (Scheme 1).^22^

**Figure 4:**
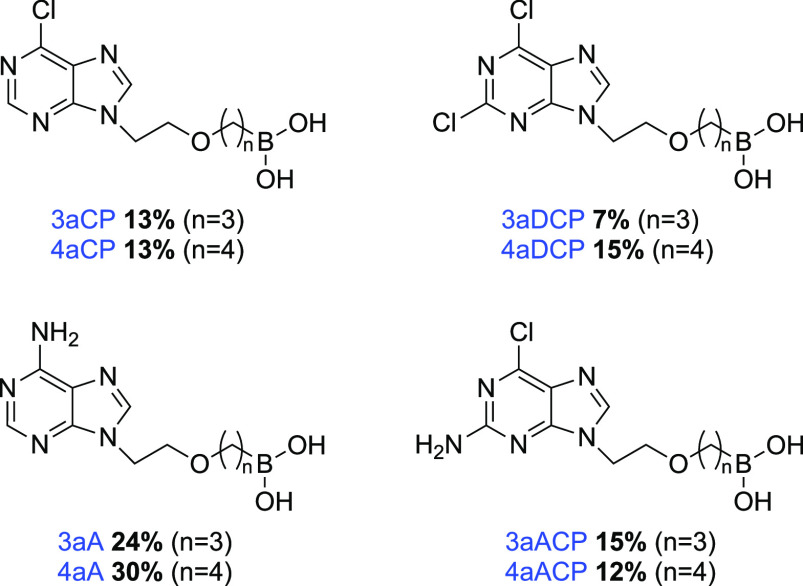
Final compound library of adefovir-boronic acid nucleoside analogs (**naNuc**). Isolated yields reported. All compound purity values ≥95% by quantitative NMR (qNMR). Compound Identification Key: **n** = alkyl chain length, **a** = boronic acid, **Nuc** (nucleobase) = CP (6-Chloropurine); DCP (2,6-Dichloropurine); ACP (2-Amino-6-chloropurine); or A (Adenine).

Next, nucleobases (6-chloropurine (**CP**), 2,6-dichloropurine (**DCP**), and 2-amino-6-chloropurine (**ACP**)) were used in substitution reactions with **5** to produce protected alcohol nucleoside compounds of series **6**. After that, THP deprotection was performed to produce the corresponding primary alcohol nucleosides of series **7**.^23^ Synthesis of bromoalkyl potassium trifluoroborate electrophiles **8a**–**c** (propyl, butyl, and pentyl, respectively) have been reported previously by the Tomsho group.^24,25^ Finally, boronic acid-adefovir analogs were obtained via substitution reactions of bromoalkyl-potassium trifluoroborates (**8a** and **8b)** with various alcohol nucleosides (**7a, 7b, 7c**, and commercially available **7d**). In this substitution reaction, K_2_CO_3_ was used as a base with tetrabutylammonium bromide (TBAB) as a phase transfer catalyst, and the reaction was heated to 90 °C overnight. After the substitution reaction, the trifluoroborate group was hydrolyzed to the free boronic acid.^26,27^ These final compounds were purified by silica gel flash column chromatography (3–10% MeOH in DCM) (Figure 4).

The crude ^1^H NMR yields for adefovir-boronic acid nucleoside analogs range from 55 to 88%. However, the isolated yields for the compounds fall between 7 and 30%. These discrepancies between the ^1^H NMR yields and the isolated yields occurred due to difficulties during column chromatography separation. The retention factor (Rf) of the alcohol compounds **7a**–**d** and the desired final compounds are similar, making the separation challenging. In attempts to optimize these reactions, the reaction time was increased to 2 days to ensure complete conversion of the alcohol compounds **7a**–**d** to the desired final compounds. However, increasing the reaction time resulted in decomposition of both the alcohol compounds **7a**–**d** and the final adefovir-boronic acid nucleoside compounds, which suggests an inherent limitation of the stability of these compounds.

Synthesis of **naNuc** reactions generally proceeded well with high regio- and chemoselectivity. Some alcohol nucleobase analogs (**7a**–**d**) have more than one nucleophilic center with the potential to undergo alkylation following deprotonation (Scheme 1). For example, 2-amino-6-chloropurine and adenine intermediates **7c** and **7d**, respectively, have an exocyclic amine group which may alkylated. We have applied 2-D NMR methods (COSY, HSQC, and HMBC) to confirm the position of substitution on the nucleoside analogs (Figure 4).

In an effort to expand these types of syntheses to include nucleosides with more complex three-dimensional structures, we envisioned generating a second library of borononucleoside compounds with attached ribose-type rings. These include ubiquitous, natural, and medicinally relevant synthetic scaffolds such as thymidine, uridine, stavudine, lamivudine, and emtricitabine. We expected these new target nucleosides may be accessible via a similar one-step substitution reaction as described above, in which the nucleoside is alkylated using a trifluoroborate-containing halide electrophile **8a**–**c**. This would give rise to a large number of synthetic analogs quickly and easily using variations on a single substitution reaction. We anticipated synthesizing an inaugural series of 5′-hydroxyl alkylated boron-containing thymidine compounds (**3aT**, **4aT**, and **5aT**). However, when designing the experimental conditions for substitution, we were cognizant that the nucleoside starting materials have multiple possible sites for substitution. In the example of thymidine, there are three nucleophilic positions on the molecule with the potential to undergo substitution, with a relative nucleophilicity of: 5′-primary hydroxyl >3′-secondary hydroxyl > internal nucleobase amine.

Preliminary experimentation focused on using thymidine as a template nucleoside and 4-carbon trifluoroborate electrophile **8b** as the alkylating agent (Table 1). These reactions were conducted in DMF due to the poor solubility of starting material in other polar aprotic organic solvents like tetrahydrofuran. Experiments were left to react for 6 h. Using sodium hydride (NaH) as a base for the initial deprotonation step, our first optimization experiment was performed using low temperatures (0 °C) with 1.05 equiv of thymidine substrate (Table 1, Exp 1). This resulted in the generation of only one substitution product as seen in the crude ^1^H NMR spectrum, which was subsequently isolated using flash column chromatography (3% MeOH in DCM). Though confirmation of the product formed was nontrivial due to all potential regioisomers having similar physical and spectroscopic properties, we utilized 2D NMR experiments (COSY, HSQC, and HMBC) to elucidate the regioisomer formed. By analyzing the cross-correlation signals between protons and carbons on the alkyl side chain with those on the nucleoside scaffold in the heteronuclear multiple bond correlation (HMBC) NMR spectrum, we determined the product formed was nucleobase-substituted regioisomer, **N4aT** (172 mg, 0.55 mmol, 31%). This substitution pattern was rationalized by the relative p*K*_a_ values of the thymidine labile protons (NH < 5′–OH < 3′–OH), giving rise to the nucleobase-deprotonated intermediate, which acts as a hindered, but viable nucleophile during the reaction.

**Table 1:**
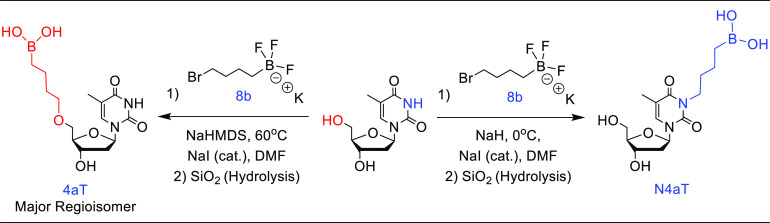
Model Reaction Optimization Studies of **4aT/N4aT** Exploring Base Identity, Stoichiometry, and Temperature Effect on Regioselective Product Formation^a^

exp	base	base eq	temp (°C)	product ratio by ^1^H NMR
1	NaH	1.05	0	N4aT
2	NaH	3.0	0	N4aT: 4aT (8:1)
3	NaH	1.05	r.t	N4aT: 4aT (6:1)
4	NaH	1.05	60	N4aT: 4aT (4:1)
5	NaHMDS	1.05	0	N4aT: 4aT (2:1)
6	NaHMDS	3.0	0	no reaction
7	NaHMDS	1.05	r.t	N4aT: 4aT (1:1)
8	NaHMDS	1.05	60	N4aT: 4aT (1:6)
9	NaHMDS	3.0	60	no reaction
10	NaHMDS	1.05	90	SM degradation

a Compound identification key: **N** = nucleobase-substituted, **n** = alkyl chain length, **a** = boronic acid, **Nuc** (nucleoside) = T (thymidine).

Although we were pleased to have proof of concept and our first borononucleoside generated, we endeavored to continue developing and optimizing the reaction to provide access to the 5′-hydroxyl substituted analogs we had originally envisioned making. To this end, we studied the effects of base identity, stoichiometry, and reaction temperature on the regioselectivity of product formation (Table 1). First, we hypothesized that the use of 3 equiv of NaH base may be sufficient to globally deprotonate all positions on the thymidine nucleoside, which would allow the relative rates of nucleophilicity for each site to determine regioisomer formation (Table 1, Exp 2). By analyzing the crude reaction mixture by ^1^H NMR spectroscopy, we were able to determine the relative ratio of regioisomer products formed. This was achieved with a high degree of confidence by comparing and contrasting the integration intensities of the product peaks corresponding to the 5′-CH_2_ units. These signals resonate at different chemical shift values and have distinct J-coupling constants which are indicative of position of substitution. The product peak identities were also corroborated by tandem HMBC NMR analysis. While we expected the most nucleophilic 5′-hydroxyl location to dominate and give us the desired **4aT** product, this was not observed, and the major isomer was once again **N4aT** (8:1). Next, we hypothesized that increasing the temperature of the nucleoside-base premix would shift the deprotonation event toward the **4aT** product. To this end, we attempted reactions at room temperature (Table 1, Exp 3), as well as under mild heating (Table 1, Exp 4). While increases in the proportion of **4aT** were observed, the predominant regioisomer formed was still the nucleobase-substituted, **N4aT** (6:1 and 4:1, respectively).

**Scheme 2:**
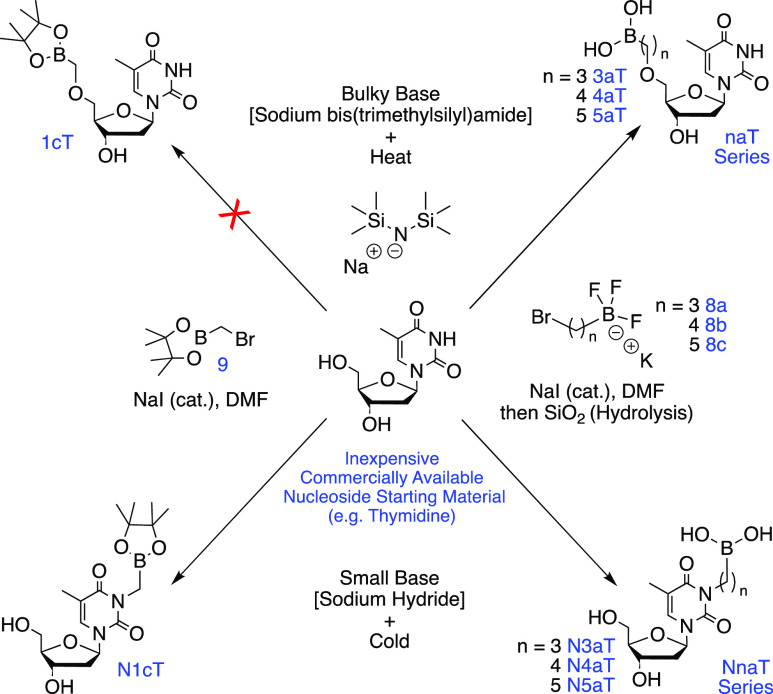
Formation of Boron-Containing Nucleoside Analogs Utilizing Base and Temperature-Dependent Regioselective Control Compound identification key: **N** = nucleobase-substituted, **n** = alkyl chain length, **a** = boronic acid, **c** = pinacolborane, and **Nuc** (nucleoside) = T (Thymidine).

It was determined that the simple sodium hydride base was ultimately too small to deprotonate at any other site than the most acidic proton. As such, it was proposed that the use of a larger, bulkier base may prevent abstraction of the sterically hindered nucleobase proton, favoring the easily accessible 5′-primary hydroxyl instead. To test this theory, sodium bis(trimethylsilyl)amide (NaHMDS) was employed as the base of choice, and all other conditions remained the same as the original experiment (Table 1, Exp 5). Indeed, the largest ratio of **4aT** was observed using the bulkier amide base over sodium hydride (2:1). This gave us encouragement that we might be able to manipulate the reaction conditions further to push the deprotonation event toward the 5′-hydroxyl position. Therefore, we revisited using 3 equiv of base, this time with NaHMDS as the deprotonating agent (Table 1, Exp 6). However, in this instance, no consumption of thymidine was observed and starting material appeared to precipitate out of solution upon addition of base. We considered whether increasing the temperature with NaHMDS might have a similar effect at shifting product formation away from **N4aT**, as was observed in experiments 3 and 4. To examine this, we attempted the reaction at room temperature and at 60 °C (Table 1, Exp 7 and 8, respectively). To our delight, increasing the reaction temperature to room temperature gave a 1:1 ratio of products, and a further increase to 60 °C yielded **4aT** as the major regioisomer (**N4aT**:**4aT**, 1:6). It was noted that increasing the temperature much beyond 60 °C caused degradation of the thymidine substrate, as was observed at 90 °C (Table 1, Exp 10). Having successfully developed and optimized two separate sets of conditions for the model substitution reaction, we have the ability to generate both the nucleobase- (Table 1, Exp 1) and 5′-hydroxyl- (Table 1, Exp 8) substituted analogs using modifications on a single reaction. This gives us the means to synthesize the entire thymidine nucleoside series of analogs under regioselective control of product formation (Scheme 2).

Next, we attempted to extend these reactions to include various chain length electrophiles. Unfortunately, the one-carbon trifluoroborate electrophile (BrCH_2_BF_3_K) cannot be used for alkylation as it is ultimately too unreactive. To circumvent this issue, we proposed using bromomethylpinacolborane **9** as an alternative electrophile to form the one carbon alkyl thymidine analogs. Utilizing analogous methodology (Table 1, Exp 1), we were able to synthesize N-substituted analog **N1cT** (245 mg, 0.64 mmol, 31%). Unfortunately, we were unable to generate the 5′-hydroxyl analog **1cT** regardless of reaction conditions employed, likely due to instability of the resulting product which has yet to be detected (Scheme 2). Furthermore, the two-carbon electrophile (Br(CH_2_)_2_BF_3_K) and its pinacolborane equivalent (Br(CH_2_)_2_BPin) are chemically unstable; therefore, the two-carbon nucleoside analogs remain synthetically inaccessible at this time. As predicted, the three-carbon and five-carbon alkyl trifluoroborate electrophiles, **8a** and **8c**, respectively, behaved analogously to the model four-carbon electrophile **8b** and followed the same chemical reactivity and regioselectivity using the developed methodology. Overall, the inaugural seven-membered thymidine analog series was synthesized using the optimized conditions to give: N1cT (31%), N3aT (33%), N4aT (30%), N5aT (53%), 3aT (24%), 4aT (34%), and 5aT (25%) (Scheme 2).

**Figure 5:**
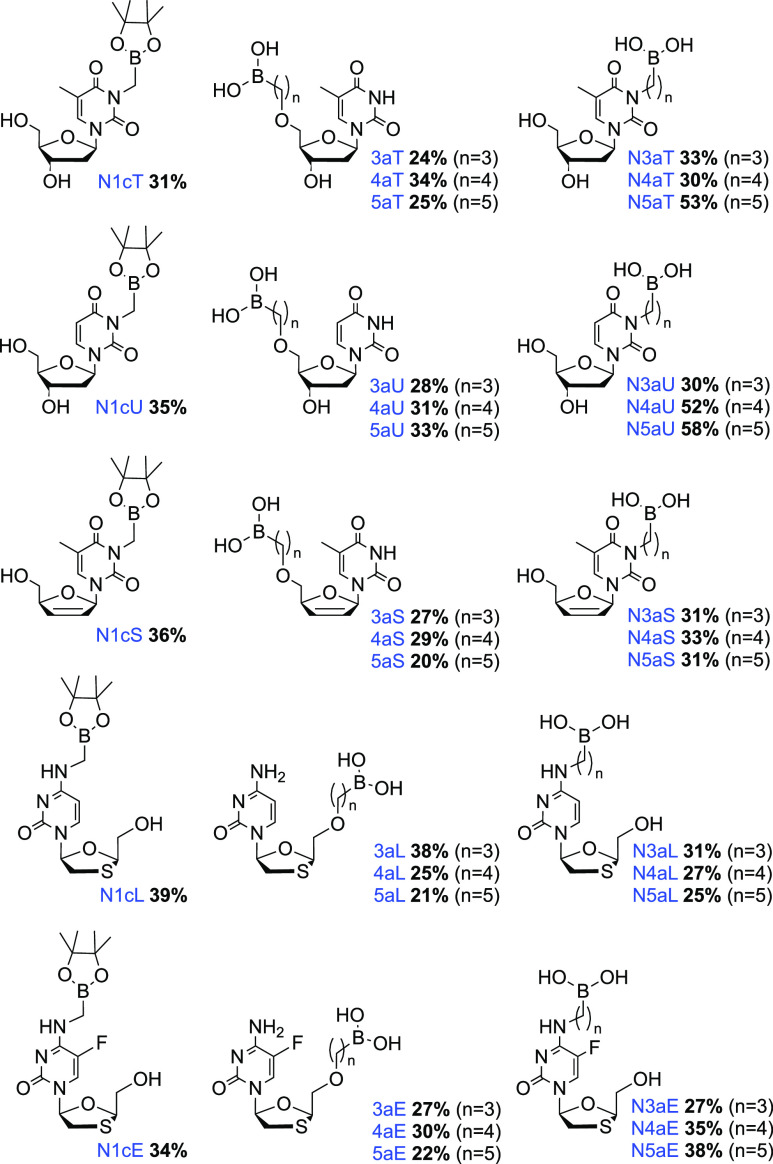
First generation library of boron-containing nucleoside analogs. Isolated yields reported. All compound purity values >95% by quantitative NMR (qNMR). Compound identification key: **N** = nucleobase-substituted, **n** = alkyl chain length, **a** = boronic acid, **c** = pinacolborane, **Nuc** (nucleoside) = T (Thymidine); U (Uridine); S (Stavudine); L (Lamivudine); or E (Emtricitabine).

Isolated yields reported were often significantly lower than those determined by integration of the different compound peaks in the ^1^H NMR of the crude products. Starting material conversion was high; however, a loss in recovered yield is attributed to the tight separation between compounds during preparative scale flash column chromatography. Improved isolated yields may be achieved on a small scale using HPLC separation. The methodology developed to generate the thymidine analog series was then successfully used to expand the scope of nucleoside series to include uridine, stavudine, lamivudine, and emtricitabine (Figure 5). The alkylation reactions showed similar regioselectivity for each nucleoside series under the optimized reaction conditions. Furthermore, these substitution reactions demonstrated functional group tolerance toward all five-nucleoside series employed. This first-generation library of boron-containing nucleoside analogs will be evaluated for their ability to act as potential therapeutic agents.

## Conclusion

In summary, we have synthesized two libraries of boron-containing nucleoside analogs, which may find utility toward therapeutic applications. We have expanded the scope of boron-containing nucleoside compounds to include alkylated boronic acids for a number of different nucleoside scaffolds, including 6-chloropurine, 2,6-dichloropurine, 2-amino-6-chloropurine, adenine, thymidine, uridine, stavudine, lamivudine, and emtricitabine. Furthermore, we have developed methodology to regioselectively access both nucleobase- and 5′-hydroxyl-substituted analogs using condition modifications on a single reaction.

## Experimental Section

### General Experimental Methods

All synthetic routes were performed according to the standard lab safety guidelines described in Prudent Practices in the Laboratory: Handling and Management of Chemical Hazards.^28^ All reactants and reagents were purchased and used without further purification. Almost all reactions were carried out under an inert atmosphere of argon using a glovebox or Schlenk line. Rotary evaporation was used to remove the solvents. Final compound drying was done under high vacuum (ca. 0.01 Torr). Thin layer chromatography (TLC) was performed using silica gel 200 μM precoated polyester backed plates with a fluorescent indicator, and TLC plates were visualized with UV light (254 nm). Flash column chromatography was conducted with the indicated solvent system using normal phase silica gel 40–63 μM, 230–400 mesh. Structural assignments were made with additional information from gCOSY, gHSQC, and gHMBC experiments. ^1^H NMR spectra were recorded on a Bruker Avance at 400 MHz, ^13^C NMR spectra were recorded at 100 MHz, and ^11^B NMR spectra were recorded at 128 MHz. Chemical shifts are reported in δ values (ppm) relative to an internal reference of tetramethyl silane (TMS) or the residual solvent signal. Peak splitting patterns in the ^1^H NMR are reported as follows: s, singlet; d, doublet; t, triplet; q, quartet; m, multiplet. Mass spectra were obtained on a Thermo- Fisher Exactive Orbitrap Mass Spectrometer using Electrospray Ionization. Compounds **8a**–**c** were synthesized using previously reported procedures.^24,25^ Commercially available nucleosides: 6-chloropurine, 2,6-dichloropurine, 2-amino-6-chloropurine, adenine, thymidine, uridine, stavudine, lamivudine, and emtricitabine were purchased from reputable chemical vendors with purity >99%.

### 2-Bromo-1-ethoxytetrahydropyran, 5

To an Ar-flushed and flame-dried rbf containing 2-bromoethanol (16.01 mmol, 1.0 eq, 2.00 g) was added dry DCM (80 mL). Then, p-TSA (1.60 mmol, 10%, 276 mg) was added quickly followed by the addition of DHP (20.8 mmol, 1.3 eq, 1.75 g) at room temperature. The mixture became homogeneous upon vigorous stirring, and the resulting solution was stirred overnight. The reaction was quenched with saturated aqueous NaHCO_3_ and transferred to a separatory funnel. The aqueous layer was extracted with DCM (3 × 50 mL), and the combined organics were washed with water and brine. The organics were combined and concentrated to yield **5** as a yellow oil (3.16 g, 15.99 mmol, 95%). ^1^H NMR (400 MHz, CDCl_3_) δ 4.69 (t, 1H), 4.01 (m, 1H), 3.90 (m, 1H), 3.80 (m, 1H), 3.52 (m, 3H), 1.86 (m, 1H), 1.74 (m, 1H), 1.65 (m, 2H), 1.56 (m, 2H). ^13^C{1H} NMR (100 MHz, CDCl_3_): δ 98.9, 67.5, 62.2, 30.8, 29.3, 25.3, 19.2. HRMS (MAI) *m*/*z*: [M + H]^+^ Calcd for C_7_H_14_BrO_2_ 209.0172; Found 209.0190.

### General Procedure for the Synthesis of Nucleosidyl-9-(1-ethoxytetrahydropyran), 6

A flame-dried Ar-flushed rbf was charged with nucleoside (1.0 equiv) and K_2_CO_3_ (3.5 equiv). These were dissolved in 20 mL DMF and stirred at rt for 50 min. Next, **5** (1.0 equiv) and NaI (10 mol %) were dissolved in 20 mL dry DMF (from an Ar-flushed rbf) and were added to the reaction mixture. Then, the reaction mixture was stirred overnight at rt. The next day, the reaction mixture was filtered, and solids were rinsed with EtOAc. The combined organics were concentrated, and the product was purified by column chromatography (0–3% MeOH in EtOAc) to give nucleosidyl-9-(1-ethoxytetrahydropyran), **6**.

### 6-Chloropurinyl-9-(1-ethoxytetrahydropyran), 6a

Starting material; 6-chloropurine (1.50 g, 9.6 mmol): Column eluant 3% MeOH in EtOAc. ^1^H NMR (400 MHz, *d*_6_-DMSO) δ 8.78 (s, 1H), 8.67 (s, 1H) 4.56 (s, 1H), 4.49 (m, 2H), 4.01 (m, 1H), 3.81 (m, 1H), 3.39 (m, 1H), 3.31 (m, 1H), 1.54 (m, 2H), 1.36 (m, 4H). ^13^C{1H} NMR (100 MHz, *d*_6_-DMSO): δ 151.8, 151.7, 150.8, 146.3, 131.4, 99.1, 65.1, 62.4, 44.3, 30.3, 25.1, 19.3. HRMS (MAI) *m*/*z*: [M + H]^+^ Calcd for C_12_H_16_ClN_4_O_2_ 283.0956; Found 283.0947. Colorless oil. Yield: 1.96 g, 6.9 mmol, 72%.

### 2,6-Dichloropurinyl-9-(1-ethoxytetrahydropyran), 6b

Starting material; 2,6-dichloropurine (3.00 g, 16.0 mmol): Column eluant 100% EtOAc. ^1^H NMR (400 MHz, *d*_6_-DMSO) δ 8.68 (s, 1H), 4.56 (s, 1H), 4.47 (m, 2H), 3.96 (m, 1H), 3.78 (m, 1H), 3.44 (m, 1H), 3.33 (m, 1H), 1.54 (m, 2H), 1.38 (m, 4H). ^13^C{1H} NMR (100 MHz, *d*_6_-DMSO): δ 153.9, 151.4, 149.9, 149.1, 130.7, 98.0, 64.6, 61.6, 44.5, 30.3, 25.3, 19.2. HRMS (MAI) *m*/*z*: [M + H]^+^ Calcd for C_12_H_15_Cl_2_N_4_O_2_ 317.0567; Found 317.0557. Yellow oil. Yield: 3.35 g, 10.6 mmol, 66%.

### 2-Amino-6-chloropurinyl-9-(1-ethoxytetrahydropyran), 6c

Starting material; 2-amino-6-chloropurine (2.44 g, 14.4 mmol): Column eluant 100% EtOAc. ^1^H NMR (400 MHz, *d*_6_-DMSO) δ 8.09 (s, 1H), 6.91 (s, 2H) 4.56 (s, 1H), 4.23 (m, 2H), 3.91 (m, 1H), 3.71 (m, 1H), 3.46 (m, 1H), 3.34 (m, 1H), 1.55 (m, 2H), 1.41 (m, 4H). ^13^C{1H} NMR (100 MHz, *d*_6_-DMSO): δ 160.2, 154.5, 149.7, 144.0, 123.6, 98.0, 64.6, 61.5, 43.5, 30.4, 25.3, 19.2. HRMS (MAI) *m*/*z*: [M + H]+ Calcd for C12H17ClN5O2 298.1065; Found 298.1092. White powder. Yield: 1.93 g, 6.47 mmol, 45%.

### General Procedure for the Synthesis of Alcohol Analogs, 7

A flame-dried Ar-flushed rbf was charged with compound **6** (1.0 equiv) and dissolved in 25 mL of methanol. After that, p-TSA (10%) was added, and the mixture was stirred for 2 h. Product formation was monitored by TLC. When complete, the reaction mixture was filtered and concentrated to yield analogs **7** which was used without further purification.

### 6-Chloropurinyl-9-ethanol, 7a

Starting material; **6a** (987 mg, 3.49 mmol): ^1^H NMR (400 MHz, *d*_6_-DMSO) δ 8.78 (s, 1H), 8.64 (s, 1H), 5.01 (t, 1H), 4.35 (t, 2H), 3.80 (q, 2H). ^13^C{1H} NMR (100 MHz, *d*_6_-DMSO): δ 151.8, 148.4, 128.5, 125.9, 59.3, 47.0. HRMS (MAI) *m*/*z*: [M + H]^+^ Calcd for C_7_H_8_ClN_4_O 199.0381; Found 199.0381. Off-white powder. Yield: 569 mg, 2.86 mmol, 82%.

### 2,6-Dichloropurinyl-9-ethanol, 7b

Starting material; **6b** (1.14 g, 3.63 mmol): ^1^H NMR (400 MHz, *d*_6_-DMSO) δ 8.26 (s, 1H), 4.44 (t, 2H), 4.08 (q, 2H), 2.23 (t, 1H). ^13^C{1H} NMR (100 MHz, *d*_6_-DMSO): δ 153.1, 152.9, 151.8, 146.8, 60.6, 46.6. HRMS (MAI) *m*/*z*: [M + H]^+^ Calcd for C_7_H_7_Cl_2_N_4_O 232.9991; Found 232.9984. Yellow powder. Yield: 624 mg, 2.67 mmol, 74%.

### 2-Amino-6-chloropurinyl-9-ethanol, 7c

Starting material; **6c** (894 mg, 3.01 mmol): ^1^H NMR (400 MHz, *d*_6_-DMSO) δ 8.10 (s, 1H), 4.10 (t, 2H), 4.35 (t, 2H), 3.72. ^13^C{1H} NMR (100 MHz, *d*_6_-DMSO): δ 160.1, 154.5, 149.5, 144.2, 128.5, 59.2, 46.3. HRMS (MAI) *m*/*z*: [M + H]^+^ Calcd for C_7_H_9_ClN_5_O 214.0490; Found 214.0509. Off-white powder. Yield: 538 mg, 2.51 mmol, 84%.

### General Procedure for the Synthesis of O-Substituted Adefovir-Boronic Acid Nucleoside Series, naNuc

An Ar-flushed, oven-dried rbf was charged with alcohol analogs **7** (1.0 equiv) and K_2_CO_3_ (3.5 equiv). These were dissolved in 20 mL dry DMF and stirred at rt for 50 min. Next, **8a** or **8b** electrophile (1.0 equiv) was added to the reaction mixture followed by addition of TBAB (10%). The reaction was heated up to 90 °C in an oil bath and stirred overnight. The reaction mixture was then filtered and concentrated. The crude residue was dissolved in 20 mL MeOH before adding 3 mL H_2_O and (6.0 equiv) silica. The mixture was stirred for 4 h before it was filtered and concentrated. The crude boronic acid was purified by column chromatography, eluting with (4–10% MeOH in DCM) to give **naNuc**.

### Compound Identification Key

**n** = alkyl chain length **a** = boronic acid **Nuc** (nucleobase) =CP (6-Chloropurine) DCP (2,6-Dichloropurine) ACP (2-Amino-6-chloropurine) A (Adenine)

### 6-Chloropurinyl-9-((ethoxymethyl)-3-propyl Boronic Acid, 3aCP

Starting material; **7a** (500 mg, 2.53 mmol): Column eluant 10% MeOH in DCM. ^1^H NMR (400 MHz, CD_3_OD) δ 8.72 (s, 1H), 8.538 (s, 1H), 4.537 (t, 2H, *J* = 5.02 Hz), 3.825 (t, 2H, *J* = 5.05 Hz), 3.388 (t, 2H, *J* = 6.32 Hz), 1.530 (m, 2H), 0.626 (t, 2H, *J* = 7.69 Hz). ^13^C{1H} NMR (100 MHz, CD_3_OD): δ 153.3, 152.8, 151.0, 148.9, 132.1, 73.9, 68.9, 45.4, 24.8. ^11^B NMR (128 MHz, CD_3_OD): δ 31.66. q-NMR purity: 95.25%. HRMS (ESI-TOF) *m*/*z*: [M - H]^−^ Calcd for C_10_H_14_BClN_4_O_3_ 283.0764; Found 283.0764. White powder. Yield: 92 mg, 0.32 mmol, 13%.

### 6-Chloropurinyl-9-((ethoxymethyl)-4-butyl Boronic Acid, 4aCP

Starting material; **7a** (408 mg, 2.06 mmol): Column eluant 10% MeOH in DCM. ^1^H NMR (400 MHz, CD_3_OD) δ 8.728 (s, 1H), 8.53 (s, 1H), 4.54 (t, 2H, *J* = 5.00 Hz), 3.824 (t, 2H, *J* = 5.05 Hz), 3.425 (t, 2H, *J* = 6.30 Hz), 1.45 (m, 2H), 1.26 (m, 2H), 0.667 (t, 2H, *J* = 7.88 Hz). ^13^C{1H} NMR (100 MHz, CD_3_OD): δ 153.3, 152.8, 151.1, 148.9, 132.1, 71.9, 69.0, 45.4, 33.1, 21.3. ^11^B NMR (128 MHz, CD_3_OD): δ 31.64. q-NMR purity: 97.00%. HRMS (ESI-TOF) *m*/*z*: [M - H]^−^ Calcd for C_11_H_16_BClN_4_O_3_ 297.0920; Found 297.0920. White powder. Yield: 82 mg, 0.28 mmol, 13%.

### 2,6-Dichloropurinyl-9-((ethoxymethyl)-3-propyl Boronic Acid, 3aDCP

Starting material; **7b** (500 mg, 2.15 mmol): Column eluant 4% MeOH in DCM. ^1^H NMR (400 MHz, CD_3_OD) δ 8.52 (s, 1H), 4.48 (t, 2H, *J* = 5.03 Hz), 3.81 (t, 2H, *J* = 5.06 Hz), 3.40 (t, 2H, *J* = 6.40 Hz), 1.54 (m, 2H), 0.64 (t, 2H, *J* = 7.71 Hz). ^13^C{1H} NMR (100 MHz, CD_3_OD): δ154.7, 153.7, 151.7, 149.6, 131.4, 73.9, 68.8, 45.5, 24.8. ^11^B NMR (128 MHz, CD_3_OD): δ 31.57. q-NMR purity: 96.02%. HRMS (ESI-TOF) *m*/*z*: [M - H]^−^ Calcd for C_10_H_13_BCl_2_N_4_O_3_ 317.0374; Found 317.0374. Pale yellow powder. Yield: 48 mg, 0.15 mmol, 7%.

### 2,6-Dichloropurinyl-9-((ethoxymethyl)-4-butyl Boronic Acid, 4aDCP

Starting material; **7b** (820 mg, 3.52 mmol): Column eluant 4% MeOH in DCM. ^1^H NMR (400 MHz, CD_3_OD) δ 8.51 (s, 1H), 4.48 (t, 2H, *J* = 5.05 Hz), 3.80 (t, 2H, *J* = 5.09 Hz), 3.43 (t, 2H, *J* = 6.30 Hz), 1.46 (m, 2H), 1.27 (m, 2H), 0.67 (t, 2H, *J* = 7.88 Hz). ^13^C{1H} NMR (100 MHz, CD_3_OD): δ154.7, 153.6, 151.7, 149.6, 131.4, 71.9, 68.9, 45.5, 33.1, 21.3. ^11^B NMR (128 MHz, CD_3_OD): δ 31.67. q-NMR purity: 96.73%. HRMS (ESI-TOF) *m*/*z*: [M - H]^−^ Calcd for C_11_H_15_BCl_2_N_4_O_3_ 331.0531; Found 331.0531. Pale yellow powder. Yield: 173 mg, 0.52 mmol, 15%.

### 2-Amino-6-chloropurinyl-9-((ethoxymethyl)-3-propyl Boronic Acid, 3aACP

Starting material; **7c** (497 mg, 2.18 mmol): Column eluant 10% MeOH in DCM. ^1^H NMR (400 MHz, CD_3_OD) δ 8.04 (s, 1H), 4.29 (t, 2H, *J* = 5.01 Hz), 3.75 (t, 2H, *J* = 5.08 Hz), 3.38 (t, 2H, *J* = 6.35 Hz), 1.55 (m, 2H), 0.65 (t, 2H, *J* = 7.79 Hz). ^13^C{1H} NMR (100 MHz, CD_3_OD): δ 161.5, 155.2, 151.3, 145.2, 124.7, 73.9, 68.9, 44.7, 24.9. ^11^B NMR (128 MHz, CD_3_OD): δ 31.67. q-NMR purity: 96.87%. HRMS (ESI-TOF) *m*/*z*: [M - H]^−^ Calcd for C_10_H_15_BClN_5_O_3_ 298.0873; Found 298.0873. White powder. Yield: 95 mg, 0.32 mmol, 15%.

### 2-Amino-6-chloropurinyl-9-((ethoxymethyl)-4-butyl Boronic Acid, 4aACP

Starting material; **7c** (432 mg, 1.9 mmol): Column eluant 10% MeOH in DCM. ^1^H NMR (400 MHz, CD_3_OD) δ 8.03 (s, 1H), 4.29 (t, 2H, *J* = 5.02 Hz), 3.75 (t, 2H, *J* = 5.06 Hz), 3.42 (t, 2H, *J* = 6.38 Hz), 1.47 (m, 2H), 1.30 (m, 2H), 0.69 (t, 2H, *J* = 7.82 Hz). ^13^C{1H} NMR (100 MHz, CD_3_OD): δ 161.5, 155.2, 151.4, 145.2, 124.7, 72.0, 69.1, 44.72, 33.1, 21.4. ^11^B NMR (128 MHz, CD_3_OD): δ 31.67. q-NMR purity: 97.20%. HRMS (ESI-TOF) *m*/*z*: [M - H]^−^ Calcd for C_11_H_17_BClN_5_O_3_ 312.1029; Found 312.1030. White powder. Yield: 63 mg, 0.20 mmol, 12%.

### Adeninyl-9-((ethoxymethyl)-3-propyl Boronic Acid, 3aA

Starting material; 2-(6-aminopurin-9-yl)ethanol **7d** (500 mg, 2.79 mmol): Column eluant 10% MeOH in DCM. ^1^H NMR (400 MHz, CD_3_OD) δ 8.20 (s, 1H), 8.10 (s, 1H), 4.38 (t, 2H, *J* = 5.04 Hz), 3.76 (t, 2H, *J* = 5.04 Hz), 3.38 (t, 2H, *J* = 6.40 Hz), 1.54 (m, 2H), 0.64 (t, 2H, *J* = 7.64 Hz). ^13^C{1H} NMR (100 MHz, CD_3_OD): δ 157.2, 153.6, 150.6, 143.4, 119.8, 73.9, 69.3, 44.9, 24.9. ^11^B NMR (128 MHz, CD_3_OD): δ 31.68. q-NMR purity: 99.44%. HRMS (ESI-TOF) *m*/*z*: [M - H]^−^ Calcd for C_10_H_16_BN_5_O_3_ 264.1262; Found 264.1262. White powder. Yield: 181 mg, 0.68 mmol, 24%.

### Adeninyl-9-((ethoxymethyl)-4-butyl Boronic Acid, 4aA

Starting material; 2-(6-aminopurin-9-yl) ethanol **7d** (500 mg, 2.79 mmol): Column eluant 10% MeOH in DCM. ^1^H NMR (400 MHz, CD_3_OD) δ 8.20 (s, 1H), 8.11 (s, 1H), 4.38 (t, 2H, *J* = 5.01 Hz), 3.76 (t, 2H, *J* = 5.08 Hz), 3.42 (t, 2H, *J* = 6.33 Hz), 1.50 (m, 2H), 1.26 (m, 2H), 0.16 (t, 2H, *J* = 7.81 Hz). ^13^C{1H} NMR (100 MHz, CD_3_OD): δ 157.2, 153.5, 150.6, 143.5, 119.8. 72.8, 69.4, 44.9, 34.0, 22.6. ^11^B NMR (128 MHz, CD_3_OD): δ 31.64. q-NMR purity: 95.47%. HRMS (ESI-TOF) *m*/*z*: [M - H]^−^ Calcd for C_11_H_18_BN_5_O_3_ 278.1419; Found 278.1419. White powder. Yield: 235 mg, 0.84 mmol, 30%.

### General Procedure for the Synthesis of N-Substituted Boronic Acid Nucleosides, NnaNuc

An oven-dried rbf was entered into the glovebox and loaded with sodium hydride (1.05 equiv) and sodium iodide (0.2 equiv), then sealed, removed, placed under an atmosphere of argon gas and cooled to 0 °C. Next, dry DMF (20 mL) was added, followed by nucleoside (400 mg, 1.0 equiv) as a single solid portion, and the mixture was stirred on ice for 45 min. Br(CH_2_)_n_BF_3_K electrophile **8a**–**c** (1.1 equiv) was then added and the reaction was left to slowly return to rt and stirred for 6 h. The solvents were removed *in vacuo* and the crude mixture was redissolved in methanol (20 mL), to which water (3 mL) and silica (6.0 equiv) were added. The mixture was stirred for 4 h, then filtered and the filtrate concentrated. The crude mixture was subjected to flash column chromatography (3–10% MeOH in DCM).

### Compound Identification Key

**N** = nucleobase-substituted **n** = alkyl chain length **a** = boronic acid **Nuc** (nucleobase) =T (Thymidine) U (Uridine) S (Stavudine) L (Lamivudine) E (Emtricitabine)

### N3aT

Nucleoside starting material; Thymidine (400 mg, 1.65 mmol): Column eluant 5% MeOH in DCM. ^1^H NMR (400 MHz, CD_3_OD): δ 7.83 (1H, s), 6.30 (1H, t, *J* = 7.0 Hz), 4.40 (1H, m), 3.94–3.85 (3H, m), 3.78 (2H, ddd, *J* = 25, 15, 3.5 Hz), 2.32–2.25 (1H, m), 2.24–2.16 (1H, m), 1.90 (3H, d, *J* = 2.0 Hz), 1.75–1.62 (2H, m), 0.78 (2H, t, *J* = 8.0 Hz) ppm. ^13^C{1H} NMR (100 MHz, CD_3_OD): δ 165.6, 152.4, 136.3, 110.7, 88.8, 87.1, 72.1, 62.7, 44.2, 41.4, 22.9, 13.2 ppm. ^11^B NMR (128 MHz, CD_3_OD): δ 31.43 ppm. q-NMR purity: 97.77%. HRMS (ESI-TOF) *m*/*z*: [M-H]^−^ Calcd for C_13_H_20_BN_2_O_7_ 327.1358; Found 327.1360. White powder. Yield: 179 mg, 0.54 mmol, 33%.

### N4aT

Nucleoside starting material; Thymidine (400 mg, 1.65 mmol): Column eluant 5% MeOH in DCM. ^1^H NMR (400 MHz, CD_3_OD): δ 7.82 (1H, s), 6.31 (1H, t, *J* = 7.0 Hz), 4.39 (1H, m), 3.93–3.84 (3H, m), 3.76 (2H, ddd, *J* = 26, 15, 3.5 Hz), 2.31–2.24 (1H, m), 2.23–2.15 (1H, m), 1.90 (3H, s), 1.62–1.54 (2H, m), 1.45–1.34 (2H, m), 0.82 (2H, t, *J* = 8.0 Hz) ppm. ^13^C{1H} NMR (100 MHz, CD_3_OD): δ 165.4, 152.3, 136.4, 110.7, 88.8, 87.1, 72.1, 62.8, 42.2, 41.3, 31.2, 22.5, 13.2 ppm. ^11^B NMR (128 MHz, CD_3_OD): δ 31.64 ppm. q-NMR purity: 96.31%. HRMS (ESI-TOF) *m*/*z*: [M-H]^−^ Calcd for C_14_H_22_BN_2_O_7_ 341.1515; Found 341.1514. White powder. Yield: 170 mg, 0.50 mmol, 30%.

### N5aT

Nucleoside starting material; Thymidine (400 mg, 1.65 mmol): Column eluant 5% MeOH in DCM. ^1^H NMR (400 MHz, CD_3_OD): δ 7.83 (1H, s), 6.30 (1H, t, *J* = 7.0 Hz), 4.39 (1H, m), 3.93–3.86 (3H, m), 3.76 (2H, ddd, *J* = 27, 15, 3.5 Hz), 2.32–2.26 (1H, m), 2.25–2.15 (1H, m), 1.91 (3H, s), 1.64–1.53 (2H, m), 1.46–1.27 (4H, m), 0.78 (2H, t, *J* = 8.0 Hz) ppm. ^13^C{1H} NMR (100 MHz, CD_3_OD): δ 165.4, 152.3, 136.4, 110.7, 88.9, 87.1, 72.1, 62.8, 42.3, 41.3, 30.7, 28.4, 24.6, 13.1 ppm. ^11^B NMR (128 MHz, CD_3_OD): δ 31.88 ppm. q-NMR purity: 96.44%. HRMS (ESI-TOF) *m*/*z*: [M-H]^−^ Calcd for C_15_H_24_BN_2_O_7_ 355.1671; Found 355.1675. White powder. Yield: 312 mg, 0.88 mmol, 53%.

### N3aU

Nucleoside starting material; Uridine (400 mg, 1.75 mmol): Column eluant 10% MeOH in DCM. ^1^H NMR (400 MHz, CD_3_OD): δ 7.97 (1H, d, *J* = 8.0 Hz), 6.27 (1H, t, *J* = 7.0 Hz), 5.75 (1H, d, *J* = 8.0 Hz), 4.39 (1H, m), 3.95–3.83 (3H, m), 3.76 (2H, ddd, *J* = 26, 15, 4 Hz), 2.37–2.28 (1H, m), 2.25–2.14 (1H, m), 1.75–1.60 (2H, m), 0.78 (2H, t, *J* = 8.0 Hz) ppm. ^13^C{1H} NMR (100 MHz, CD_3_OD): δ 165.3, 152.4, 140.5, 101.9, 88.9, 87.5, 72.1, 62.7, 43.9, 41.5, 22.9 ppm. ^11^B NMR (128 MHz, CD_3_OD): δ 31.57 ppm. q-NMR purity: 97.28%. HRMS (ESI-TOF) *m*/*z*: [M-H]^−^ Calcd for C_12_H_18_BN_2_O_7_ 313.1202; Found 313.1204. White powder. Yield: 165 mg, 0.53 mmol, 30%.

### N4aU

Nucleoside starting material; Uridine (400 mg, 1.75 mmol): Column eluant 10% MeOH in DCM. ^1^H NMR (400 MHz, CD_3_OD): δ 7.96 (1H, d, *J* = 8.0 Hz), 6.26 (1H, t, *J* = 7.0 Hz), 5.75 (1H, d, *J* = 8.0 Hz), 4.38 (1H, m), 3.96–3.82 (3H, m), 3.75 (2H, ddd, *J* = 25, 15, 3 Hz), 2.37–2.29 (1H, m), 2.24–2.15 (1H, m), 1.61–1.50 (2H, m), 1.43–1.31 (2H, m), 0.80 (2H, t, *J* = 8.0 Hz) ppm. ^13^C{1H} NMR (100 MHz, CD_3_OD): δ 164.9, 152.1, 140.4, 101.9, 88.8, 87.4, 71.9, 62.7, 41.9, 41.4, 31.1, 22.6, 22.1 ppm. ^11^B NMR (128 MHz, CD_3_OD): δ 31.75 ppm. q-NMR purity: 96.81%. HRMS (ESI-TOF) *m*/*z*: [M-H]^−^ Calcd for C_13_H_20_BN_2_O_7_ 327.1358; Found 327.1359. White powder. Yield: 299 mg, 0.91 mmol, 52%.

### N5aU

Nucleoside starting material; Uridine (400 mg, 1.75 mmol): Column eluant 5% MeOH in DCM. ^1^H NMR (400 MHz, CD_3_OD): δ 7.97 (1H, d, *J* = 8.0 Hz), 6.28 (1H, t, *J* = 7.0 Hz), 5.75 (1H, d, *J* = 8.0 Hz), 4.38 (1H, m), 3.95–3.86 (3H, m), 3.75 (2H, ddd, *J* = 25, 15, 3 Hz), 2.35–2.28 (1H, m), 2.24–2.15 (1H, m), 1.64–1.54 (2H, m), 1.46–1.27 (4H, m), 0.78 (2H, t, *J* = 8.0 Hz) ppm. ^13^C{1H} NMR (100 MHz, CD_3_OD): δ 164.9, 152.2, 140.4, 101.9, 88.9, 87.4, 72.0, 62.9, 49.9, 42.1, 41.4, 30.6, 28.6, 24.5 ppm. ^11^B NMR (128 MHz, CD_3_OD): δ 31.81 ppm. q-NMR purity: 96.27%. HRMS (ESI-TOF) *m*/*z*: [M-H]^−^ Calcd for C_14_H_22_BN_2_O_7_ 341.1515; Found 341.1513. White powder. Yield: 348 mg, 1.02 mmol, 58%.

### N3aS

Nucleoside starting material; Stavudine (400 mg, 1.78 mmol): Column eluant 3% MeOH in DCM. ^1^H NMR (400 MHz, CD_3_OD): δ 7.75 (1H, m), 6.99 (1H, m), 6.40 (1H, m), 5.90 (1H, m), 4.86 (1H, m), 3.92 (2H, m), 3.75 (2H, m), 1.86 (3H, d), 1.69 (2H, m), 0.79 (2H, m) ppm. ^13^C{1H} NMR (100 MHz, CD_3_OD): δ 165.7, 153.0, 137.1, 135.9, 127.4, 110.5, 92.0, 89.0, 63.8, 44.3, 23.0, 13.1 ppm. ^11^B NMR (128 MHz, CD_3_OD): δ 31.53 ppm. q-NMR purity: 96.99%. HRMS (ESI-TOF) *m*/*z*: [M-H]^−^ Calcd for C_13_H_18_BN_2_O_6_ 309.1252; Found 309.1254. White powder. Yield: 172 mg, 0.55 mmol, 31%.

### N4aS

Nucleoside starting material; Stavudine (400 mg, 1.78 mmol): Column eluant 3% MeOH in DCM. ^1^H NMR (400 MHz, CD_3_OD): δ 7.74 (1H, s), 6.99 (1H, m), 6.40 (1H, dt, *J* = 6.0 Hz), 5.91 (1H, m), 4.87 (1H, m), 3.91 (2H, m), 3.76 (2H, m), 1.86 (3H, s), 1.64–1.54 (2H, m), 1.45–1.35 (2H, m), 0.82 (2H, t, *J* = 8.0 Hz) ppm. ^13^C{1H} NMR (100 MHz, CD_3_OD): δ 165.5, 152.9, 137.1, 135.9, 127.3, 110.4, 91.9, 89.0, 63.8, 42.3, 31.2, 22.3, 13.1 ppm. ^11^B NMR (128 MHz, CD_3_OD): δ 31.69 ppm. q-NMR purity: 96.28%. HRMS (ESI-TOF) *m*/*z*: [M-H]^−^ Calcd for C_14_H_20_BN_2_O_6_ 323.1409; Found 323.1410. White powder. Yield: 191 mg, 0.59 mmol, 33%.

### N5aS

Nucleoside starting material; Stavudine (400 mg, 1.8 mmol): Column eluant 5% MeOH in DCM. ^1^H NMR (400 MHz, CD_3_OD): δ 7.75 (1H, s), 7.00 (1H, m), 6.39 (1H, dt, *J* = 6.0 Hz), 5.91 (1H, m), 4.86 (1H, m), 3.90 (2H, m), 3.75 (2H, m), 1.85 (3H, s), 1.63–1.54 (2H, m), 1.46–1.27 (4H, m), 0.79 (2H, t, *J* = 8.0 Hz) ppm. ^13^C{1H} NMR (100 MHz, CD_3_OD): δ 165.5, 152.9, 137.1, 135.9, 127.3, 110.4, 91.9, 88.9, 63.8, 42.4, 30.7, 28.4, 24.6, 13.1 ppm. ^11^B NMR (128 MHz, CD_3_OD): δ 31.70 ppm. q-NMR purity: 96.71%. HRMS (ESI-TOF) *m*/*z*: [M-H]^−^ Calcd for C_15_H_22_BN_2_O_6_ 337.1565; Found 337.1568. White powder. Yield: 187 mg, 0.55 mmol, 31%.

### N3aL

Nucleoside starting material; Lamivudine (400 mg, 1.74 mmol): Column eluant 3% MeOH in DCM. ^1^H NMR (400 MHz, CD_3_OD): δ 7.90 (1H, d, *J* = 8.0 Hz), 6.29 (1H, t, *J* = 5.0 Hz), 5.82 (1H, d, *J* = 8.0 Hz), 5.26 (1H, t, *J* = 4.0 Hz), 3.88 (2H, ddd, *J* = 25, 15, 4 Hz), 3.48 (1H, m), 3.36–3.28 (2H, m), 3.10 (1H, m), 1.63 (2H, m), 0.82 (2H, m) ppm. ^13^C{1H} NMR (100 MHz, CD_3_OD): δ 165.5, 158.3, 140.8, 96.9, 88.8, 87.5, 64.3, 43.9, 38.1, 24.4 ppm. ^11^B NMR (128 MHz, CD_3_OD): δ 31.72 ppm. q-NMR purity: 96.07%. HRMS (ESI-TOF) *m*/*z*: [M-H]^−^ Calcd for C_11_H_17_BN_3_O_5_S 314.0976; Found 314.0979. White powder. Yield: 170 mg, 0.54 mmol, 31%.

### N4aL

Nucleoside starting material; Lamivudine (400 mg, 1.74 mmol): Column eluant 3% MeOH in DCM. ^1^H NMR (400 MHz, CD_3_OD): δ 7.90 (1H, d, *J* = 8.0 Hz), 6.29 (1H, t, *J* = 8.0 Hz), 5.82 (1H, d, *J* = 8.0 Hz), 5.26 (1H, t, *J* = 4.0 Hz), 3.88 (2H, q, *J* = 12.0 Hz), 3.36 (2H, t, *J* = 7.0 Hz), 3.27 (2H, q, *J* = 12.0 Hz), 1.57 (3H, m), 1.43 (2H, m), 0.81 (3H, t, *J* = 8.0 Hz) ppm. ^13^C{1H} NMR (100 MHz, CD_3_OD): δ 163.4, 156.4, 138.8, 94.9, 86.8, 85.5, 62.3, 39.5, 36.1, 30.6, 20.3 ppm. ^11^B NMR (128 MHz, CD_3_OD): δ 31.78 ppm. q-NMR purity: 95.79%. HRMS (ESI-TOF) *m*/*z*: [M-H]^−^ Calcd for C_12_H_19_BN_3_O_5_S 328.1133; Found 328.1133. White powder. Yield: 155 mg, 0.47 mmol, 27%.

### N5aL

Nucleoside starting material; Lamivudine (400 mg, 1.74 mmol): Column eluant 5% MeOH in DCM. ^1^H NMR (400 MHz, CD_3_OD): δ 7.92 (1H, d, *J* = 8.0 Hz), 6.29 (1H, t, *J* = 5.0 Hz), 5.82 (1H, t, *J* = 7.0 Hz), 5.26 (1H, t, *J* = 4.0 Hz), 3.87 (1H, m), 3.28 (2H, m), 1.57 (3H, qu, *J* = 8.0 Hz), 1.37 (4H, m), 0.79 (2H, t, *J* = 7.0 Hz) ppm. ^13^C{1H} NMR (100 MHz, CD_3_OD): δ 165.5, 158.3, 140.8, 96.9, 88.8, 87.5, 64.3, 41.7, 38.2, 30.8, 29.9, 24.7 ppm. ^11^B NMR (128 MHz, CD_3_OD): δ 31.83 ppm. q-NMR purity: 95.91%. HRMS (ESI-TOF) *m*/*z*: [M-H]^−^ Calcd for C_13_H_21_BN_3_O_5_S 342.1289; Found 342.1288. White powder. Yield: 150 mg, 0.44 mmol, 25%.

### N3aE

Nucleoside starting material; Emtricitabine (400 mg, 1.62 mmol): Column eluant 5% MeOH in DCM. ^1^H NMR (400 MHz, CD_3_OD): δ 8.23 (1H, d, *J* = 7.0 Hz), 6.25 (1H, m), 5.27 (1H, t, *J* = 4.0 Hz), 3.93 (2H, ddd, *J* = 50, 12, 4 Hz), 3.51 (1H, m), 3.41 (2H, t, *J* = 7.0 Hz), 3.16 (1H, m), 1.69 (2H, qu, *J* = 7.0 Hz), 0.83 (2H, t, *J* = 8.0 Hz) ppm. ^13^C{1H} NMR (100 MHz, CD_3_OD): δ 157.4, 156.5, 125.7, 125.4, 88.8, 88.4, 63.6, 43.7, 38.7, 24.3 ppm. ^11^B NMR (128 MHz, CD_3_OD): δ 31.71 ppm. q-NMR purity: 97.15%. HRMS (ESI-TOF) *m*/*z*: [M + H]^+^ Calcd for C_11_H_18_BFN_3_O_5_S 334.1039; Found 334.1039. White powder. Yield: 146 mg, 0.44 mmol, 27%.

### N4aE

Nucleoside starting material; Emtricitabine (400 mg, 1.62 mmol): Column eluant 5% MeOH in DCM. ^1^H NMR (400 MHz, CD_3_OD): δ 8.22 (1H, d, *J* = 7.0 Hz), 6.25 (1H, m), 5.27 (1H, t, *J* = 4.0 Hz), 3.93 (2H, ddd, *J* = 50, 13, 3 Hz), 3.51 (1H, m), 3.44 (2H, t, *J* = 7.0 Hz), 3.16 (1H, m), 1.61 (2H, m), 1.43 (2H, m), 0.82 (2H, t, *J* = 8.0 Hz) ppm. ^13^C{1H} NMR (100 MHz, CD_3_OD): δ 157.3, 156.5, 139.7, 137.3, 125.7, 125.4, 88.9, 88.4, 63.6, 41.4, 38.7, 32.6, 22.2 ppm. ^11^B NMR (128 MHz, CD_3_OD): δ 31.70 ppm. q-NMR purity: 97.43%. HRMS (ESI-TOF) *m*/*z*: [M + H]^+^ Calcd for C_12_H_20_BFN_3_O_5_S 348.1195; Found 348.1193. White powder. Yield: 197 mg, 0.57 mmol, 35%.

### N5aE

Nucleoside starting material; Emtricitabine (400 mg, 1.62 mmol): Column eluant 5% MeOH in DCM. ^1^H NMR (400 MHz, CD_3_OD): δ 8.22 (1H, d, *J* = 7.0 Hz), 6.24 (1H, m), 5.27 (1H, t, *J* = 4.0 Hz), 3.92 (2H, ddd, *J* = 50, 13, 3 Hz), 3.51 (1H, m), 3.43 (2H, t, *J* = 7.0 Hz), 3.16 (1H, m), 1.61 (2H, m), 1.38 (4H, m), 0.79 (2H, t, *J* = 7.0 Hz) ppm. ^13^C{1H} NMR (100 MHz, CD_3_OD): δ 156.5, 139.7, 137.3, 125.7, 125.4, 88.9, 88.4, 63.6, 41.5, 38.7, 30.7, 29.9, 24.7 ppm. ^11^B NMR (128 MHz, CD_3_OD): δ 31.86 ppm. q-NMR purity: 97.75%. HRMS (ESI-TOF) *m*/*z*: [M + H]^+^ Calcd for C_13_H_22_BFN_3_O_5_S 362.1352; Found 362.1351. White powder. Yield: 222 mg, 0.61 mmol, 38%.

### General Procedure for the Synthesis of 5′-Hydroxyl Substituted Boronic Acid Nucleosides, naNuc

An oven-dried rbf was entered into the glovebox and loaded with sodium iodide (0.2 equiv), then sealed, removed and placed under an atmosphere of argon gas. Dry DMF (20 mL) was added, followed by nucleoside (400 mg, 1.0 equiv) as a single solid portion, and the mixture was heated to 60 °C in an oil bath. Next, sodium bis(trimethylsilyl)amide (NaHMDS, 1.05 equiv) was added dropwise and the mixture was stirred for 45 min. Br(CH_2_)_n_BF_3_K electrophile **8a**–**c** (1.1 equiv) was then added and the reaction was left to stir at 60 °C in an oil bath for 6 h. The solvents were removed *in vacuo* and the crude mixture was redissolved in methanol (20 mL), to which water (3 mL) and silica (6.0 equiv) was added. The mixture was stirred for 4 h, then filtered and the filtrate concentrated. The crude mixture was subjected to flash column chromatography (3–10% MeOH in DCM).

### Compound Identification Key

**n** = alkyl chain length **a** = boronic acid **Nuc** (nucleobase) =T (Thymidine) U (Uridine) S (Stavudine) L (Lamivudine) E (Emtricitabine)

### 3aT

Nucleoside starting material; Thymidine (400 mg, 1.65 mmol): Column eluant 5% MeOH in DCM. ^1^H NMR (400 MHz, CD_3_OD): δ 7.77 (1H, m), 6.30 (1H, t, *J* = 7.0 Hz), 4.41 (1H, m), 4.01 (1H, dd, *J* = 6.0, 3.0 Hz), 3.66 (2H, ddd, *J* = 38.0, 11.0, 3.0 Hz), 3.56–3.43 (2H, m), 2.30–2.16 (2H, m), 1.89 (3H, d, *J* = 2.0 Hz), 1.70 (2H, qu, *J* = 7.0 Hz), 0.82 (2H, t, *J* = 8.5 Hz) ppm. ^13^C{1H} NMR (100 MHz, CD_3_OD): δ 163.4, 152.3, 137.9, 111.4, 87.8, 86.5, 74.7, 72.9, 71.8, 41.4, 25.3, 12.6 ppm. ^11^B NMR (128 MHz, CD_3_OD): δ 31.78 ppm. q-NMR purity: 95.92%. HRMS (ESI-TOF) *m*/*z*: [M-H]^−^ Calcd for C_13_H_20_BN_2_O_7_ 327.1361; Found 327.1360. White powder. Yield: 130 mg, 0.40 mmol, 24%.

### 4aT

Nucleoside starting material; Thymidine (400 mg, 1.65 mmol): Column eluant 5% MeOH in DCM. ^1^H NMR (400 MHz, CD_3_OD): δ 7.76 (1H, s), 6.30 (1H, t, *J* = 7.0 Hz), 4.40 (1H, m), 4.02 (1H, dd, *J* = 6.0, 3.0 Hz), 3.65 (2H, ddd, *J* = 50.0, 11.0, 3.0 Hz), 3.57–3.46 (2H, m), 2.30–2.15 (2H, m), 1.88 (3H, d, *J* = 2.0 Hz), 1.70–1.55 (2H, m), 1.51–1.40 (2H, m), 0.81 (2H, t, *J* = 8.0 Hz) ppm. ^13^C{1H} NMR (100 MHz, CD_3_OD): δ 166.4, 152.3, 137.9, 111.3, 87.9, 86.5, 73.0, 72.7, 71.8, 54.8, 41.5, 33.6, 21.7, 12.6 ppm. ^11^B NMR (128 MHz, CD_3_OD): δ 31.78 ppm. q-NMR purity: 95.46%. HRMS (ESI-TOF) *m*/*z*: [M-H]^−^ Calcd for C_14_H_22_BN_2_O_7_ 341.1515; Found 341.1516. White powder. Yield: 192 mg, 0.56 mmol, 34%.

### 5aT

Nucleoside starting material; Thymidine (400 mg, 1.65 mmol): Column eluant 5% MeOH in DCM. ^1^H NMR (400 MHz, CD_3_OD): δ 7.76 (1H, s), 6.30 (1H, t, *J* = 7.5 Hz), 4.40 (1H, qu, *J* = 3.0 Hz), 4.02 (1H, q, *J* = 3.0 Hz), 3.66 (2H, ddd, *J* = 38, 11, 3.0 Hz), 3.57–3.46 (3H, m), 2.30–2.16 (2H, m), 1.89 (3H, d, *J* = 1.5 Hz), 1.67–1.57 (2H, m), 1.45–1.33 (4H, m), 0.78 (2H, t, *J* = 7.0 Hz) ppm. ^13^C{1H} NMR (100 MHz, CD_3_OD): δ 166.4, 152.3, 137.9, 111.3, 87.9, 86.6, 73.1, 72.8, 71.8, 41.5, 30.8, 30.2, 24.8, 12.7 ppm. ^11^B NMR (128 MHz, CD_3_OD): δ 31.70 ppm. q-NMR purity: 96.59%. HRMS (ESI-TOF) *m*/*z*: [M-H]^−^ Calcd for C_15_H_24_BN_2_O_7_ 355.1671; Found 355.1672. White powder. Yield: 147 mg, 0.41 mmol, 25%.

### 3aU

Nucleoside starting material; Uridine (400 mg, 1.75 mmol): Column eluant 10% MeOH in DCM. ^1^H NMR (400 MHz, CD_3_OD): δ 8.01 (1H, d, *J* = 8.0 Hz), 6.27 (1H, t, *J* = 7.0 Hz), 5.68 (1H, d, *J* = 8.5 Hz), 4.39 (1H, m), 4.01 (1H, m), 3.65 (2H, ddd, *J* = 25.0, 11.0, 3.0 Hz), 3.47 (2H, m), 2.33–2.17 (1H, m), 1.66 (2H, m), 0.82 (2H, t, *J* = 8.0 Hz) ppm. ^13^C{1H} NMR (100 MHz, CD_3_OD): δ 166.2, 156.2, 142.4, 127.4, 88.9, 87.7, 73.0, 71.5, 39.5, 33.3, 21.6, 13.4 ppm. ^11^B NMR (128 MHz, CD_3_OD): δ 31.67 ppm. q-NMR purity: 95.71%. HRMS (ESI-TOF) *m*/*z*: [M-H]^−^ Calcd for C_12_H_18_BN_2_O_7_ 313.1202; Found 313.1205. White powder. Yield: 154 mg, 0.49 mmol, 28%.

### 4aU

Nucleoside starting material; Uridine (400 mg, 1.75 mmol): Column eluant 10% MeOH in DCM. ^1^H NMR (400 MHz, CD_3_OD): δ 8.01 (1H, d, *J* = 8.0 Hz), 6.27 (1H, t, *J* = 7.0 Hz), 5.70 (1H, d, *J* = 8.0 Hz), 4.40 (1H, m), 4.03 (1H, m), 3.65 (2H, ddd, *J* = 45.0, 11.0, 3.0 Hz), 3.56–3.47 (1H, m), 2.35–2.26 (1H, m), 2.40–2.14 (1H, m), 1.64–1.53 (2H, m), 1.50–1.40 (2H, m), 0.80 (1H, t, *J* = 8.0 Hz) ppm. ^13^C{1H} NMR (100 MHz, CD_3_OD): δ 166.2, 152.2, 142.4, 102.5, 87.9, 86.9, 72.8, 72.6, 71.7, 41.7, 33.5, 21.9, 13.6 ppm. ^11^B NMR (128 MHz, CD_3_OD): δ 31.75 ppm. q-NMR purity: 95.34%. HRMS (ESI-TOF) *m*/*z*: [M-H]^−^ Calcd for C_13_H_20_BN_2_O_7_ 327.1358; Found 327.1360. White powder. Yield: 178 mg, 0.54 mmol, 31%.

### 5aU

Nucleoside starting material; Uridine (400 mg, 1.75 mmol): Column eluant 5% MeOH in DCM. ^1^H NMR (400 MHz, CD_3_OD): δ 8.04 (1H, d, *J* = 8.0 Hz), 6.22 (1H, t, *J* = 7.0 Hz), 5.74 (1H, d, *J*= 8.0 Hz), 4.36 (1H, m), 3.99 (1H, m), 3.59 (2H, ddd, *J* = 27.0, 12.0, 3 Hz), 3.32 (2H, m), 2.37–2.19 (2H, m), 1.63 (4H, m), 0.81 (2H, t, *J* = 8.0 Hz) ppm. ^13^C{1H} NMR (100 MHz, CD_3_OD): δ 168.2, 155.6, 142.9, 123.1, 88.1, 86.6, 72.1, 71.1, 40.5, 39.7, 33.9, 26.4, 21.2, 13.6 ppm. ^11^B NMR (128 MHz, CD_3_OD): δ 31.59 ppm. q-NMR purity: 96.04%. HRMS (ESI-TOF) *m*/*z*: [M-H]^−^ Calcd for C_14_H_22_BN_2_O_7_ 341.1515; Found 341.1516. White powder. Yield: 198 mg, 0.58 mmol, 33%.

### 3aS

Nucleoside starting material; Stavudine (400 mg, 1.8 mmol): Column eluant 3% MeOH in DCM. ^1^H NMR (400 MHz, CD_3_OD): δ 7.64 (1H, s), 6.94 (1H, m), 6.41 (1H, dt, *J* = 6.0 Hz), 5.87 (1H, m), 4.93 (1H, m), 3.66 (2H, ddd, *J* = 20, 10, 3.5 Hz), 3.48–3.39 (2H, m), 1.85 (3H, s), 1.70–1.58 (2H, m), 0.81–0.73 (2H, m) ppm. ^13^C{1H} NMR (100 MHz, CD_3_OD): δ 166.5, 152.8, 138.8, 135.9, 126.8, 111.2, 90.9, 87.5, 74.7, 72.2, 25.1, 12.5 ppm. ^11^B NMR (128 MHz, CD_3_OD): δ 31.68 ppm. q-NMR purity: 96.56%. HRMS (ESI-TOF) *m*/*z*: [M-H]^−^ Calcd for C_13_H_18_BN_2_O_6_ 309.1252; Found 309.1255. White powder. Yield: 149 mg, 0.48 mmol, 27%.

### 4aS

Nucleoside starting material; Stavudine (400 mg, 1.8 mmol): Column eluant 3% MeOH in DCM. ^1^H NMR (400 MHz, CD_3_OD): δ 7.73 (1H, m), 6.93 (1H, m), 6.39 (1H, m), 5.86 (1H, m), 4.94 (1H, m), 6.67 (2H, m), 3.47 (2H, m), 1.56 (2H, m), 1.40 (2H, m), 0.78 (2H, t, *J* = 8.0 Hz) ppm. ^13^C{1H} NMR (100 MHz, CD_3_OD): δ 166.5, 152.8, 138.8, 135.9, 126.8, 110.1, 91.0, 87.5, 72.6, 72.7, 33.3, 21.5, 12.6 ppm. ^11^B NMR (128 MHz, CD_3_OD): δ 31.66 ppm. q-NMR purity: 95.56%. HRMS (ESI-TOF) *m*/*z*: [M-H]^−^ Calcd for C_14_H_20_BN_2_O_6_ 323.1409; Found 323.1409. White powder. Yield: 168 mg, 0.52 mmol, 29%.

### 5aS

Nucleoside starting material; Stavudine (400 mg, 1.8 mmol): Column eluant 5% MeOH in DCM. ^1^H NMR (400 MHz, CD_3_OD): δ 7.63 (1H, m), 6.93 (1H, m), 6.40 (1H, m), 5.87 (1H, m), 3.67 (2H, qu, *J* = 11.0 Hz), 3.47 (2H, m), 1.56 (3H, m), 1.35 (4H, m), 0.76 (2H, t, *J* = 7.0 Hz) ppm. ^13^C{1H} NMR (100 MHz, CD_3_OD): δ 163.4, 152.7, 138.8, 135.9, 126.8, 111.1, 90.1, 87.5, 72.7, 72.3, 30.6, 30.0, 24.8, 12.6 ppm. ^11^B NMR (128 MHz, CD_3_OD): δ 31.81 ppm. q-NMR purity: 96.76%. HRMS (ESI-TOF) *m*/*z*: [M-H]^−^ Calcd for C_15_H_22_BN_2_O_6_ 337.1565; Found 337.1567. White powder. Yield: 121 mg, 0.36 mmol, 20%.

### 3aL

Nucleoside starting material; Lamivudine (400 mg, 1.74 mmol): Column eluant 3% MeOH in DCM. ^1^H NMR (400 MHz, CD_3_OD): δ 8.08 (1H, d, *J* = 8.0 Hz), 6.28 (1H, m), 5.87 (1H, d, *J* = 8.0 Hz), 5.34 (1H, t, *J* = 4.0 Hz), 3.84 (2H, ddd, *J* = 40, 15, 4 Hz), 3.56–3.47 (3H, m), 3.14 (1H, m), 1.67 (2H, m), 0.83 (2H, m) ppm. ^13^C{1H} NMR (100 MHz, CD_3_OD): δ 167.7, 157.8, 142.9, 95.5, 88.9, 86.6, 74.8, 72.4, 38.8, 25.2 ppm. ^11^B NMR (128 MHz, CD_3_OD): δ 31.73 ppm. q-NMR purity: 97.07%. HRMS (ESI-TOF) *m*/*z*: [M-H]^−^ Calcd for C_11_H_17_BN_3_O_5_S 314.0976; Found 314.0979. White powder. Yield: 209 mg, 0.66 mmol, 38%.

### 4aL

Nucleoside starting material; Lamivudine (400 mg, 1.74 mmol): Column eluant 3% MeOH in DCM. ^1^H NMR (400 MHz, CD_3_OD): δ 8.10 (1H, d, *J* = 7.0 Hz), 6.29 (1H, m), 5.87 (1H, d, *J* = 8.0 Hz), 5.34 (1H, t, *J* = 3.0 Hz), 3.85 (2H, q, *J* = 11.0 Hz), 3.57 (2H, t, *J* = 7.0 Hz), 3.32 (2H, q, J = 12.0 Hz), 1.61 (2H, m) 1.47 (2H, m), 0.82 (2H, t, *J* = 7.0 Hz) ppm. ^13^C{1H} NMR (100 MHz, CD_3_OD): δ 165.8, 156.0, 141.0, 86.9, 84.7, 70.8, 70.4, 36.9, 31.4, 19.6 ppm. ^11^B NMR (128 MHz, CD_3_OD): δ 31.66 ppm. q-NMR purity: 96.11%. HRMS (ESI-TOF) *m*/*z*: [M-H]^−^ Calcd for C_12_H_19_BN_3_O_5_S 328.1133; Found 328.1134. White powder. Yield: 144 mg, 0.44 mmol, 25%.

### 5aL

Nucleoside starting material; Lamivudine (400 mg, 1.74 mmol): Column eluant 5% MeOH in DCM. ^1^H NMR (400 MHz, CD_3_OD): δ 8.10 (1H, d, *J*= 8.0 Hz), 6.28 (1H, m), 5.86 (1H, d, *J* = 7.5 Hz), 5.35 (1H, t, *J* = 4.0 Hz), 3.85 (2H, ddd, *J* = 37, 11, 3.5 Hz), 3.56 (2H, t, *J* = 7.0 Hz), 3.51 (1H, dd, *J* = 12, 5.5 Hz), 3.15 (1H, dd, *J* = 12, 5.5 Hz), 1.61 (2H, qu, *J* = 7.0 Hz), 1.45–1.34 (4H, m), 0.79 (2H, t, *J* = 7.5 Hz) ppm. ^13^C{1H} NMR (100 MHz, CD_3_OD): δ 167.8, 157.9, 143.0, 95.4, 88.9, 86.7, 72.9, 72.4, 38.9, 30.7, 30.1, 24.8 ppm. ^11^B NMR (128 MHz, CD_3_OD): δ 31.82 ppm. q-NMR purity: 97.24%. HRMS (ESI-TOF) *m*/*z*: [M-H]^−^ Calcd for C_13_H_21_BN_3_O_5_S 342.1289; Found 342.1286. White powder. Yield: 126 mg, 0.37 mmol, 21%.

### 3aE

Nucleoside starting material; Emtricitabine (400 mg, 1.62 mmol): Column eluant 5% MeOH in DCM. ^1^H NMR (400 MHz, CD_3_OD): δ 8.39 (1H, d, *J* = 7.0 Hz), 6.23 (1H, m), 5.36 (1H, t, *J* = 4.0 Hz), 3.87 (2H, ddd, *J* = 70, 11, 3 Hz), 3.55 (1H, m), 3.39 (1H, m), 1.71 (2H, qu, *J* = 7.5 Hz), 1.71 (2H, m), 0.84 (2H, t, *J* = 7.5 Hz) ppm. ^11^B NMR (128 MHz, CD_3_OD): δ 31.76 ppm. ^13^C{1H} NMR (100 MHz, CD_3_OD): δ 156.2, 151.9, 141.4, 117.9, 87.6, 75.0, 71.6, 39.4, 30.6, 25.1 ppm. q-NMR purity: 97.14%. HRMS (ESI-TOF) *m*/*z*: [M + H]^+^ Calcd for C_11_H_18_BFN_3_O_5_S 334.1039; Found 334.1037. White powder. Yield: 147 mg, 0.44 mmol, 27%.

### 4aE

Nucleoside starting material; Emtricitabine (400 mg, 1.62 mmol): Column eluant 5% MeOH in DCM. ^1^H NMR (400 MHz, CD_3_OD): δ 8.41 (1H, d, *J* = 7.0 Hz), 6.22 (1H, m), 5.36 (1H, t, *J* = 3.5 Hz), 3.88 (2H, ddd, *J* = 77, 12, 3 Hz), 3.54 (1H, m), 3.36 (1H, m), 1.64 (2H, qu, *J* = 7.5 Hz), 1.47 (4H, m), 0.81 (2H, t, *J* = 7.5 Hz) ppm. ^11^B NMR (128 MHz, CD_3_OD): δ 31.67 ppm. ^13^C{1H} NMR (100 MHz, CD_3_OD): δ 156.1, 151.9, 141.6, 127.8, 88.9, 87.7, 73.0, 71.5, 39.5, 33.3, 21.6 ppm. q-NMR purity: 96.52%. HRMS (ESI-TOF) *m*/*z*: [M + H]^+^ Calcd for C_12_H_20_BFN_3_O_5_S 348.1195; Found 348.1197. White powder. Yield: 177 mg, 0.49 mmol, 30%.

### 5aE

Nucleoside starting material; Emtricitabine (400 mg, 1.62 mmol): Column eluant 5% MeOH in DCM. ^1^H NMR (400 MHz, CD_3_OD): δ 7.81 (1H, d, *J* = 7.0 Hz), 6.30 (1H, m), 5.24 (1H, t, *J* = 4.0 Hz), 3.97 (2H, m), 3.88 (2H, ddd, *J* = 27, 12, 4.0 Hz), 3.44 (1H, dd, *J* = 12, 6.0 Hz), 3.17 (1H, dd, *J* = 12, 6.0 Hz), 1.64 (2H, qu, *J* = 7.5 Hz), 1.46–1.28 (4H, m), 0.79 (2H, t, *J* = 7.5 Hz) ppm. ^11^B NMR (128 MHz, CD_3_OD): δ 31.74 ppm. ^13^C{1H} NMR (100 MHz, CD_3_OD): δ 154.2, 153.9, 150.5, 141.0, 138.8, 117.9, 117.5, 88.4, 87.4, 64.0, 49.8, 43.5, 37.4, 30.6, 27.4, 24.7 ppm. q-NMR purity: 96.68%. HRMS (ESI+): *m*/*z* calcd. for [C_13_H_22_BFN_3_O_5_S] [M + H]^+^ HRMS (ESI-TOF) *m*/*z*: [M + H]^+^ Calcd for C_13_H_22_BFN_3_O_5_S 362.1352; Found 362.1352. White powder. Yield: 129 mg, 0.36 mmol, 22%.

### General Procedure for the Synthesis of N-Substituted Pinacolborane Nucleosides, NncNuc

An oven-dried rbf was entered into the glovebox and loaded with sodium hydride (1.05 equiv) and sodium iodide (0.2 equiv), then sealed, removed, placed under an atmosphere of argon gas and cooled to 0 °C. Next, dry DMF (20 mL) was added, followed by nucleoside (500 mg, 1.0 equiv) as a single solid portion, and the mixture was stirred on ice for 45 min. Br(CH_2_)BPin electrophile **9** (1.1 equiv) was then added dropwise and the reaction was left to slowly return to rt and stirred for 6 h. The solvents were removed *in vacuo* and the crude mixture was subjected to flash column chromatography (10% MeOH in DCM).

### Compound Identification Key

**N** = nucleobase-substituted **n** = alkyl chain length **c** = pinacolborane **Nuc** (nucleobase) =T (Thymidine) U (Uridine S (Stavudine) L (Lamivudine) E (Emtricitabine)

### N1cT

Nucleoside starting material; Thymidine (500 mg, 2.06 mmol): ^1^H NMR (400 MHz, CD_3_OD): δ 8.30 (1H, s), 6.25 (1H, t, *J* = 6.5 Hz), 4.40 (1H, m), 3.96 (1H, q, *J* = 3.5 Hz), 3.80 (2H, ddd, *J* = 25, 12, 3.5 Hz), 2.81 (2H, br s), 2.42–2.35 (1H, m), 2.28–2.21 (1H, m), 2.01 (3H, s) 1.24 (12H, br s) ppm. ^13^C{1H} NMR (100 MHz, CD_3_OD): δ 169.6, 150.3, 142.5, 105.6, 89.3, 88.2, 82.7, 75.8, 71.6, 62.3, 41.9, 25.2, 25.0, 12.1 ppm. ^11^B NMR (128 MHz, CD_3_OD): δ 16.89 ppm. q-NMR purity: 97.35%. HRMS (ESI-TOF) *m*/*z*: [M-H]^−^ Calcd for C_17_H_26_BN_2_O_7_ 381.1828; Found 381.1828. White powder. Yield: 245 mg, 0.64 mmol, 31%.

### N1cU

Nucleoside starting material; Uridine (500 mg, 2.19 mmol): ^1^H NMR (400 MHz, CD_3_OD): δ 8.43 (1H, d, *J* = 8.0 Hz), 6.24 (1H, t, *J* = 7.0 Hz), 6.10 (1H, d, *J* = 8.0 Hz), 4.40 (1H, m), 3.98 (1H, q, *J* = 7.0 Hz), 3.78 (2H, ddd, *J* = 30, 15, 3 Hz), 2.81 (2H, br s), 2.46–2.38 (1H, m), 2.30–2.21 (1H, m), 1.23 (12H, br s) ppm. ^13^C{1H} NMR (100 MHz, CD_3_OD): δ 169.6, 150.4, 145.9, 96.2, 89.5, 88.6, 82.8, 71.7, 62.4, 42.0, 25.2, 25.0 ppm. ^11^B NMR (128 MHz, CD_3_OD): δ 17.78 ppm. q-NMR purity: 97.89%. HRMS (ESI-TOF) *m*/*z*: [M-H]^−^ Calcd for C_16_H_24_BN_2_O_7_ 367.1671; Found 367.1668. White powder. Yield: 282 mg, 0.77 mmol, 35%.

### N1cS

Nucleoside starting material; Stavudine (500 mg, 2.23 mmol): ^1^H NMR (400 MHz, DMSO-*d*_6_): δ 7.76 (1H, d, *J* = 1.5 Hz), 6.86 (1H, qu, *J* = 1.5 Hz), 6.42 (1H, dt, *J* = 6.0, 1.5 Hz), 5.92 (1H, m), 5.04 (1H, t, *J* = 5.5 Hz), 4.80 (1H, m), 3.62 (2H, m), 3.10 (2H, d, *J* = 2.5 Hz), 1.79 (3H, d, *J* = 1.0 Hz), 1.16 (12H, br s) ppm. ^13^C{1H} NMR (100 MHz, DMSO-*d*_6_): δ 163.6, 150.6, 136.2, 135.2, 125.7, 107.2, 90.1, 87.5, 82.8, 62.1, 24.6, 24.5, 12.6 ppm. ^11^B NMR (128 MHz, DMSO-*d*_6_): δ 29.19 ppm. q-NMR purity: 95.52%. HRMS (ESI-TOF) *m*/*z*: [M + H]^+^ Calcd for C_17_H_26_BN_2_O_6_ 365.1878; Found 365.1875. White powder. Yield: 292 mg, 0.80 mmol, 36%.

### N1cL

Nucleoside starting material; Lamivudine (500 mg, 2.18 mmol): ^1^H NMR (400 MHz, CD_3_OD): δ 8.20 (1H, d, *J* = 8.0 Hz), 6.31 (1H, m), 6.00 (1H, d, *J* = 8.0 Hz), 5.29 (1H, t, *J* = 4.0 Hz), 3.92 (2H, ddd, *J* = 40, 16, 4 Hz), 3.54 (1H, m), 3.25 (1H, m), 2.66 (2H, br s), 1.16 (12H, br s) ppm. ^13^C{1H} NMR (100 MHz, CD_3_OD): δ 163.6, 150.1, 142.7, 93.2, 88.9, 88.5, 80.6, 63.5, 38.6, 25.1, 24.9 ppm. ^11^B NMR (128 MHz, CD_3_OD): δ 7.99 ppm. q-NMR purity: 97.01%. HRMS (ESI-TOF) *m*/*z*: [M-H]^−^ Calcd for C_15_H_23_BN_3_O_5_S 368.1444; Found 368.1446. White powder. Yield: 314 mg, 0.85 mmol, 39%.

### N1cE

Nucleoside starting material; Emtricitabine (500 mg, 2.02 mmol): ^1^H NMR (400 MHz, CD_3_OD): δ 8.64 (1H, d, *J* = 7.0 Hz), 5.30 (1H, t, *J* = 3.0 Hz), 3.96 (2H, ddd, *J* = 57, 13, 3 Hz), 3.55 (1H, m), 3.30 (1H, m), 2.68 (2H, br s), 1.18 (12H, br s) ppm. ^13^C{1H} NMR (100 MHz, CD_3_OD): 158.2, 157.1, 149.0, 128.3, 128.0, 89.6, 88.7, 80.9, 75.8, 62.8, 39.1, 25.0 ppm. ^11^B NMR (128 MHz, CD_3_OD): δ 8.48 ppm. q-NMR purity: 95.18%. HRMS (ESI-TOF) *m*/*z*: [M-H]^−^ Calcd for C_15_H_22_BFN_3_O_5_S 386.1352; Found 386.1351. White powder. Yield: 266 mg, 0.69 mmol, 34%.

## Data Availability

The data underlying this study are available in the published article and its online Supporting Information.

## Supporting Information Available

These data include spectral data (^1^H, ^13^C, ^11^B, and HMBC NMR; q-NMR; HRMS) for all novel compounds synthesized (PDF)
