# Supplementary material for: Synthesis of Boron-Containing Nucleoside Analogs
Source: J Org Chem. 2024 Jan 16;89(3):1556–66. doi: 10.1021/acs.joc.3c02179 (PMC10845115; doi:10.1021/acs.joc.3c02179)
Supplement: Supplementary file 1 — jo3c02179_si_001.pdf [file jo3c02179_si_001.pdf]

Supporting Information

Synthesis of Boron-Containing Nucleoside Analogs

Latifah M. Alhthlol[a,b,‡], Christopher L. Orme[a,‡], Ben S Jefferis[a], Sarah A. Herter[a], Halee E. Kemper[a], John W. Tomsho[a,*]

[a] Department of Chemistry & Biochemistry, St Joseph's University, University City Campus, 600 South 43rd Street, Philadelphia, PA 19104, USA

[b] Department of Chemistry, King Saud bin Abdulaziz University for Health Sciences, Al Mubarraz, Alahsa 36428, Saudi Arabia

‡ These authors contributed equally to this work; * Corresponding Author, jtomsho@sju.edu

Page Number	Item
S1-4	Index
S5-7	^{1}HNMR, ^{13}C{^{1}H}-NMR, and ^{11}BNMR spectra for compound **3aCP**
S8	HMBC-NMR spectra for compound **3aCP**
S9	HRMS negative mode for compound **3aCP**
S10	qNMR, spectra for compound **3aCP**
S11-13	^{1}HNMR, ^{13}C{^{1}H}-NMR, and ^{11}BNMR spectra for compound **4aCP**
S14	HMBC-NMR spectra for compound **4aCP**
S15	HRMS negative mode for compound **4aCP**
S16	qNMR, spectra for compound **4aCP**
S17-19	^{1}HNMR, ^{13}C{^{1}H}-NMR, and ^{11}BNMR spectra for compound **3aDCP**
S20	HMBC-NMR spectra for compound **3aDCP**
S21	HRMS negative mode for compound **3aDCP**
S22	qNMR, spectra for compound **3aDCP**
S23-25	^{1}HNMR, ^{13}C{^{1}H}-NMR, and ^{11}BNMR spectra for compound **4aDCP**
S26	HMBC-NMR spectra for compound **4aDCP**
S27	HRMS negative mode for compound **4aDCP**
S28	qNMR, spectra for compound **4aDCP**
S29-31	^{1}HNMR, ^{13}C{^{1}H}-NMR, and ^{11}BNMR spectra for compound **3aACP**
S32	HMBC-NMR spectra for compound **3aACP**
S33	HRMS negative mode for compound **3aACP**
S34	qNMR, spectra for compound **3aACP**
S35-37	^{1}HNMR, ^{13}C{^{1}H}-NMR, and ^{11}BNMR spectra for compound **4aACP**
S38	HMBC-NMR spectra for compound **4aACP**
S39	HRMS negative mode for compound **4aACP**
S40	qNMR, spectra for compound **4aACP**
S41-43	^{1}HNMR, ^{13}C{^{1}H}-NMR, and ^{11}BNMR spectra for compound **3aA**
S44	HMBC-NMR spectra for compound **3aA**
S45	HRMS negative mode for compound **3aA**
S46	qNMR, spectra for compound **3aA**
S47-49	^{1}HNMR, ^{13}C{^{1}H}-NMR, and ^{11}BNMR spectra for compound **4aA**
S50	HMBC-NMR spectra for compound **4aA**
S51	HRMS negative mode for compound **4aA**
S52	qNMR, spectra for compound **4aA**
S53-55	^{1}HNMR, ^{13}C{^{1}H}-NMR, and ^{11}BNMR spectra for compound **N3aT**

S56	HMBC-NMR spectra for compound **N3aT**
S57	HRMS negative mode for compound **N3aT**
S58	qNMR, spectra for compound **N3aT**
S59-61	^{1}HNMR, ^{13}C{^{1}H}-NMR, and ^{11}BNMR spectra for compound **N4aT**
S62	HMBC-NMR spectra for compound **N4aT**
S63	HRMS negative mode for compound **N4aT**
S64	qNMR, spectra for compound **N4aT**
S65-67	^{1}HNMR, ^{13}C{^{1}H}-NMR, and ^{11}BNMR spectra for compound **N5aT**
S68	HMBC-NMR spectra for compound **N5aT**
S69	HRMS negative mode for compound **N5aT**
S70	qNMR, spectra for compound **N5aT**
S71-73	^{1}HNMR, ^{13}C{^{1}H}-NMR, and ^{11}BNMR spectra for compound **N3aU**
S74	HMBC-NMR spectra for compound **N3aU**
S75	HRMS negative mode for compound **N3aU**
S76	qNMR, spectra for compound **N3aU**
S77-79	^{1}HNMR, ^{13}C{^{1}H}-NMR, and ^{11}BNMR spectra for compound **N4aU**
S80	HMBC-NMR spectra for compound **N4aU**
S81	HRMS negative mode for compound **N4aU**
S82	qNMR, spectra for compound **N4aU**
S83-84	^{1}HNMR, ^{13}C{^{1}H}-NMR, and ^{11}BNMR spectra for compound **N5aU**
S86	HMBC-NMR spectra for compound **N5aU**
S87	HRMS negative mode for compound **N5aU**
S88	qNMR, spectra for compound **N5aU**
S89-91	^{1}HNMR, ^{13}C{^{1}H}-NMR, and ^{11}BNMR spectra for compound **N3aS**
S92	HMBC-NMR spectra for compound **N3aS**
S93	HRMS negative mode for compound **N3aS**
S94	qNMR, spectra for compound **N3aS**
S95-97	^{1}HNMR, ^{13}C{^{1}H}-NMR, and ^{11}BNMR spectra for compound **N4aS**
S98	HMBC-NMR spectra for compound **N4aS**
S99	HRMS negative mode for compound **N4aS**
S100	qNMR, spectra for compound **N4aS**
S101-103	^{1}HNMR, ^{13}C{^{1}H}-NMR, and ^{11}BNMR spectra for compound **N5aS**
S104	HMBC-NMR spectra for compound **N5aS**
S105	HRMS negative mode for compound **N5aS**
S106	qNMR, spectra for compound **N5aS**
S107-109	^{1}HNMR, ^{13}C{^{1}H}-NMR, and ^{11}BNMR spectra for compound **N3aL**
S110	HMBC-NMR spectra for compound **N3aL**
S111	HRMS negative mode for compound **N3aL**
S112	qNMR, spectra for compound **N3aL**
S113-115	^{1}HNMR, ^{13}C{^{1}H}-NMR, and ^{11}BNMR spectra for compound **N4aL**
S116	HMBC-NMR spectra for compound **N4aL**
S117	HRMS negative mode for compound **N4aL**
S118	qNMR, spectra for compound **N4aL**
S119-121	^{1}HNMR, ^{13}C{^{1}H}-NMR, and ^{11}BNMR spectra for compound **N5aL**
S122	HMBC-NMR spectra for compound **N5aL**
S123	HRMS negative mode for compound **N5aL**
S124	qNMR, spectra for compound **N5aL**

S125-127	^{1}HNMR, ^{13}C{^{1}H}-NMR, and ^{11}BNMR spectra for compound **N3aE**
S128	HMBC-NMR spectra for compound **N3aE**
S129	HRMS positive mode for compound **N3aE**
S130	qNMR, spectra for compound **N3aE**
S131-133	^{1}HNMR, ^{13}C{^{1}H}-NMR, and ^{11}BNMR spectra for compound **N4aE**
S134	HMBC-NMR spectra for compound **N4aE**
S135	HRMS positive mode for compound **N4aE**
S136	qNMR, spectra for compound **N4aE**
S137-139	^{1}HNMR, ^{13}C{^{1}H}-NMR, and ^{11}BNMR spectra for compound **N5aE**
S140	HMBC-NMR spectra for compound **N5aE**
S141	HRMS positive mode for compound **N5aE**
S142	qNMR, spectra for compound **N5aE**
S143-145	^{1}HNMR, ^{13}C{^{1}H}-NMR, and ^{11}BNMR spectra for compound **3aT**
S146	HMBC-NMR spectra for compound **3aT**
S147	HRMS negative mode for compound **3aT**
S148	qNMR, spectra for compound **3aT**
S149-151	^{1}HNMR, ^{13}C{^{1}H}-NMR, and ^{11}BNMR spectra for compound **4aT**
S152	HMBC-NMR spectra for compound **4aT**
S143	HRMS negative mode for compound **4aT**
S154	qNMR, spectra for compound **4aT**
S155-157	^{1}HNMR, ^{13}C{^{1}H}-NMR, and ^{11}BNMR spectra for compound **5aT**
S158	HMBC-NMR spectra for compound **5aT**
S159	HRMS negative mode for compound **5aT**
S160	qNMR, spectra for compound **5aT**
S161-163	^{1}HNMR, ^{13}C{^{1}H}-NMR, and ^{11}BNMR spectra for compound **3aU**
S164	HMBC-NMR spectra for compound **3aU**
S165	HRMS positive mode for compound **3aU**
S166	qNMR, spectra for compound **3aU**
S167-169	^{1}HNMR, ^{13}C{^{1}H}-NMR, and ^{11}BNMR spectra for compound **4aU**
S170	HMBC-NMR spectra for compound **4aU**
S171	HRMS negative mode for compound **4aU**
S172	qNMR, spectra for compound **4aU**
S173-175	^{1}HNMR, ^{13}C{^{1}H}-NMR, and ^{11}BNMR spectra for compound **5aU**
S176	HMBC-NMR spectra for compound **5aU**
S177	HRMS positive mode for compound **5aU**
S178	qNMR, spectra for compound **5aU**
S179-181	^{1}HNMR, ^{13}C{^{1}H}-NMR, and ^{11}BNMR spectra for compound **3aS**
S182	HMBC-NMR spectra for compound **3aS**
S183	HRMS negative mode for compound **3aS**
S184	qNMR, spectra for compound **3aS**
S185-187	^{1}HNMR, ^{13}C{^{1}H}-NMR, and ^{11}BNMR spectra for compound **4aS**
S188	HMBC-NMR spectra for compound **4aS**
S189	HRMS negative mode for compound **4aS**
S190	qNMR, spectra for compound **4aS**
S191-193	^{1}HNMR, ^{13}C{^{1}H}-NMR, and ^{11}BNMR spectra for compound **5aS**
S194	HMBC-NMR spectra for compound **5aS**
S195	HRMS negative mode for compound **5aS**

S196	qNMR, spectra for compound **5aS**
S197-199	^{1}HNMR, ^{13}C{^{1}H}-NMR, and ^{11}BNMR spectra for compound **3aL**
S200	HMBC-NMR spectra for compound **3aL**
S201	HRMS negative mode for compound **3aL**
S202	qNMR, spectra for compound **3aL**
S203-205	^{1}HNMR, ^{13}C{^{1}H}-NMR, and ^{11}BNMR spectra for compound **4aL**
S206	HMBC-NMR spectra for compound **4aL**
S207	HRMS negative mode for compound **4aL**
S208	qNMR, spectra for compound **4aL**
S209-211	^{1}HNMR, ^{13}C{^{1}H}-NMR, and ^{11}BNMR spectra for compound **5aL**
S212	HMBC-NMR spectra for compound **5aL**
S213	HRMS negative mode for compound **5aL**
S214	qNMR, spectra for compound **5aL**
S215-217	^{1}HNMR, ^{13}C{^{1}H}-NMR, and ^{11}BNMR spectra for compound **3aE**
S218	HMBC-NMR spectra for compound **3aE**
S219	HRMS positive mode for compound **3aE**
S220	qNMR, spectra for compound **3aE**
S221-223	^{1}HNMR, ^{13}C{^{1}H}-NMR, and ^{11}BNMR spectra for compound **4aE**
S224	HMBC-NMR spectra for compound **4aE**
S225	HRMS positive mode for compound **4aE**
S226	qNMR, spectra for compound **4aE**
S227-229	^{1}HNMR, ^{13}C{^{1}H}-NMR, and ^{11}BNMR spectra for compound **5aE**
S230	HMBC-NMR spectra for compound **5aE**
S231	HRMS positive mode for compound **5aE**
S232	qNMR, spectra for compound **5aE**
S233-235	^{1}HNMR, ^{13}C{^{1}H}-NMR, and ^{11}BNMR spectra for compound **N1cT**
S236	HMBC-NMR spectra for compound **N1cT**
S237	HRMS negative mode for compound **N1cT**
S238	qNMR, spectra for compound **N1cT**
S239-241	^{1}HNMR, ^{13}C{^{1}H}-NMR, and ^{11}BNMR spectra for compound **N1cU**
S242	HMBC-NMR spectra for compound **N1cU**
S243	HRMS negative mode for compound **N1cU**
S244	qNMR, spectra for compound **N1cU**
S245-247	^{1}HNMR, ^{13}C{^{1}H}-NMR, and ^{11}BNMR spectra for compound **N1cS**
S248	HMBC-NMR spectra for compound **N1cS**
S249	HRMS negative mode for compound **N1cS**
S250	qNMR, spectra for compound **N1cS**
S251-253	^{1}HNMR, ^{13}C{^{1}H}-NMR, and ^{11}BNMR spectra for compound **N1cL**
S254	HMBC-NMR spectra for compound **N1cL**
S255	HRMS negative mode for compound **N1cL**
S256	qNMR, spectra for compound **N1cL**
S257-259	^{1}HNMR, ^{13}C{^{1}H}-NMR, and ^{11}BNMR spectra for compound **N1cE**
S260	HMBC-NMR spectra for compound **N1cE**
S261	HRMS negative mode for compound **N1cE**
S262	qNMR, spectra for compound **N1cE**

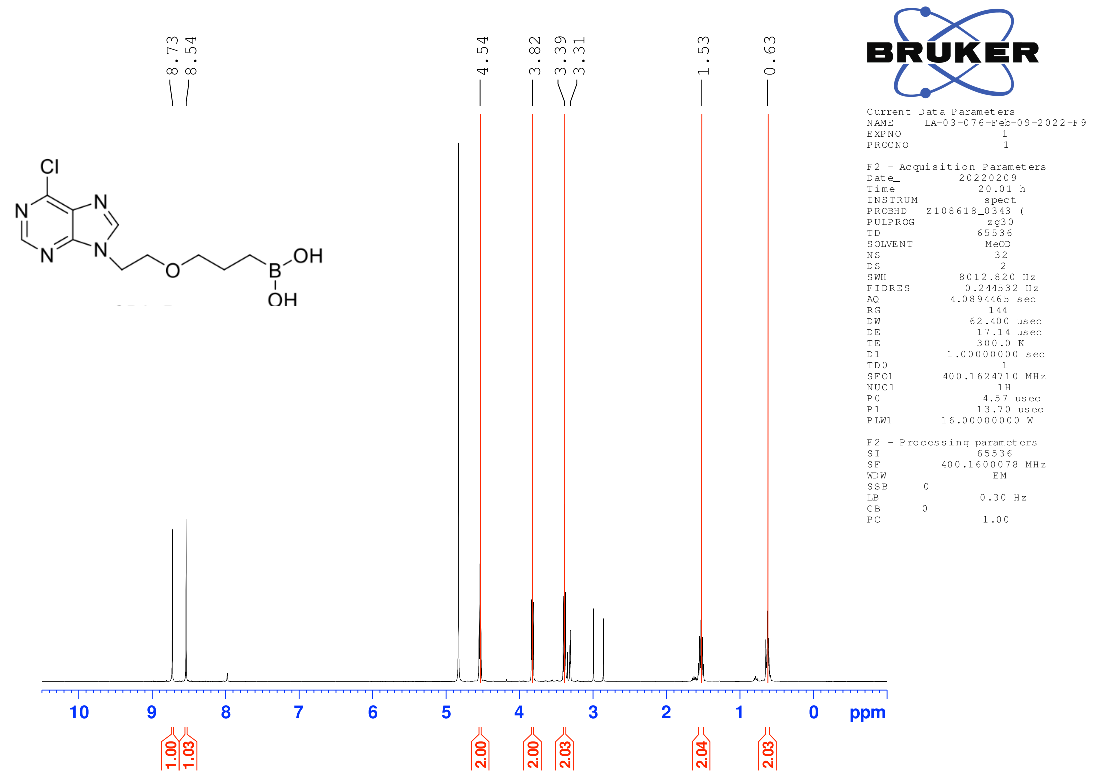

Figure S1 ^{1}HNMR spectra for compound **3aCP**

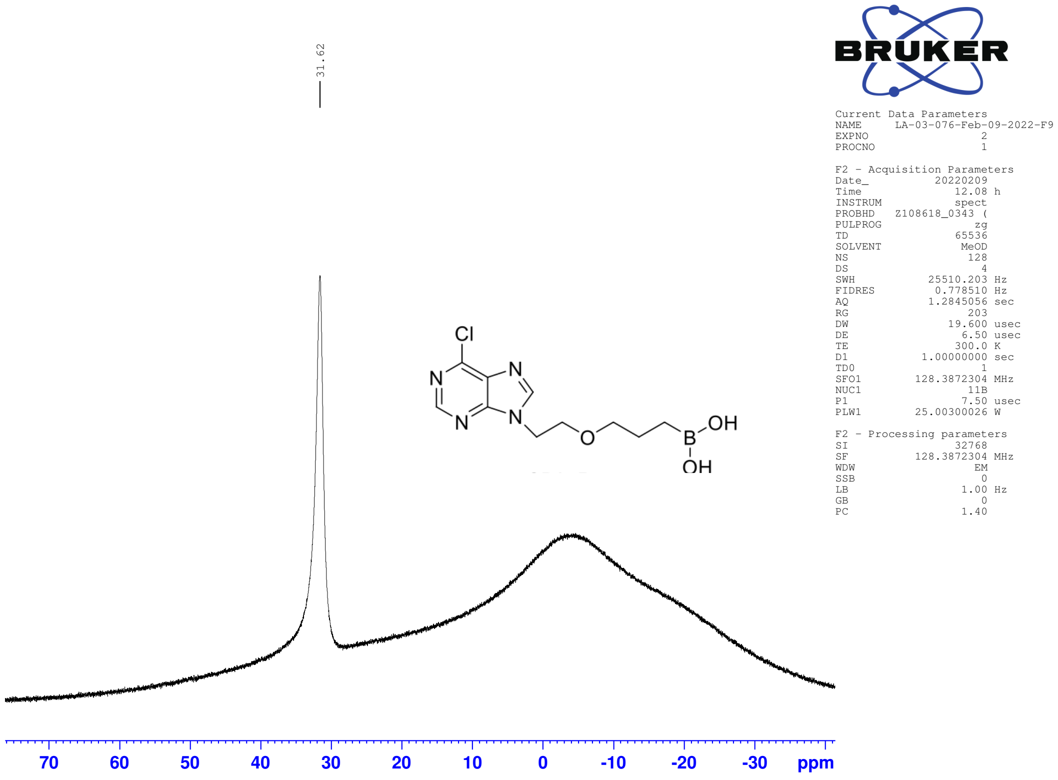

Figure S2 ^{11}BNMR spectra for compound **3aCP**

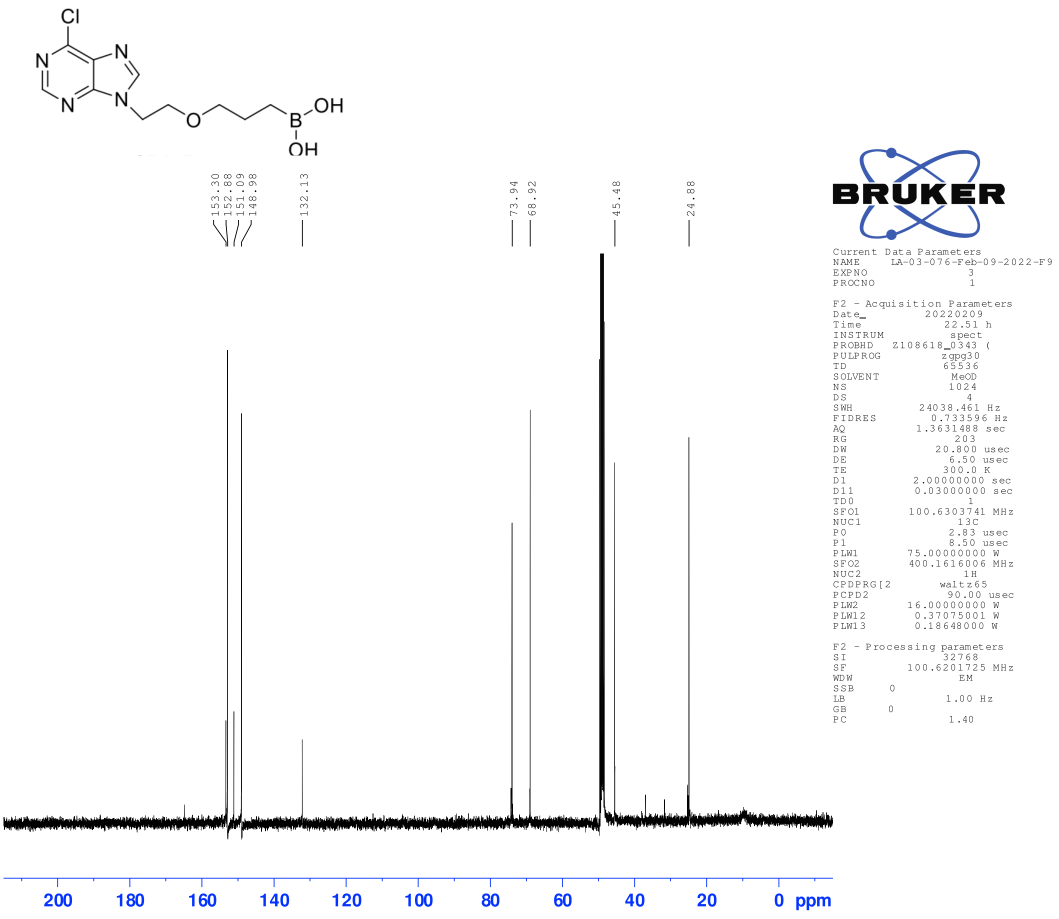

Figure S3 $^{13}C\{^{1}H\}$-NMR spectra for compound **3aCP**

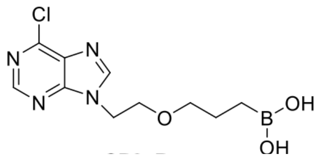

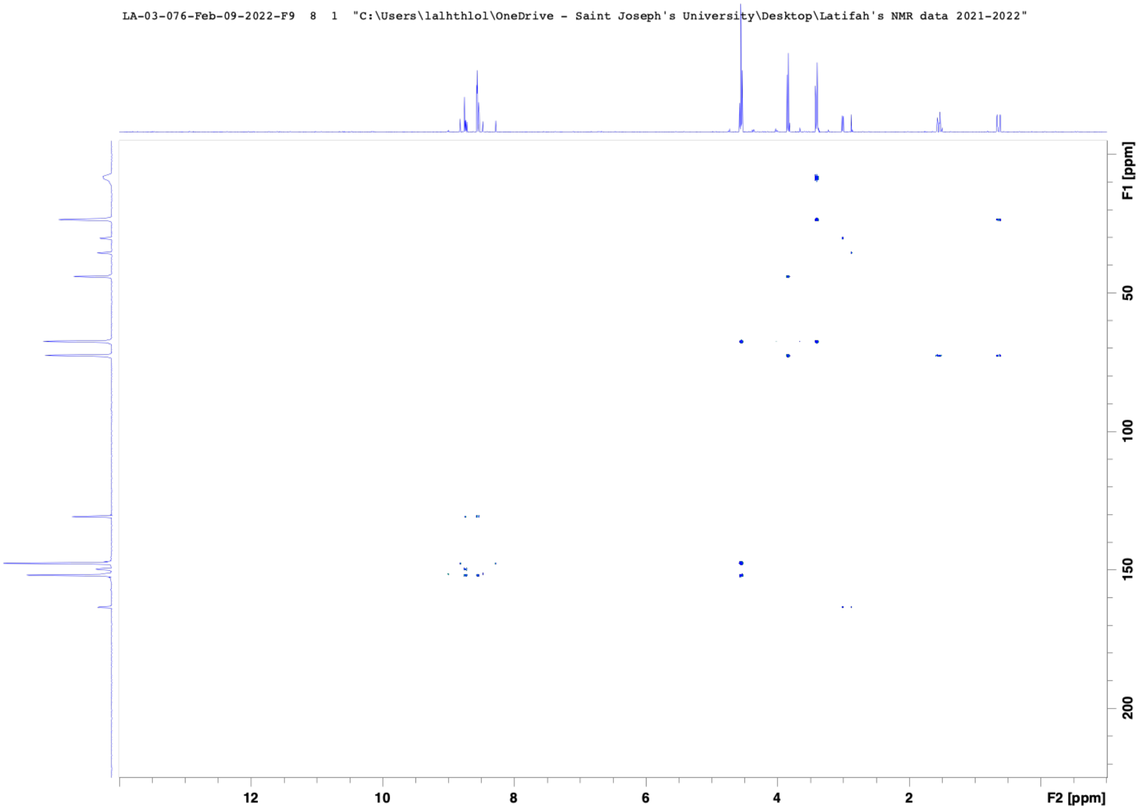

Figure S4 HMBC spectra for compound **3aCP**

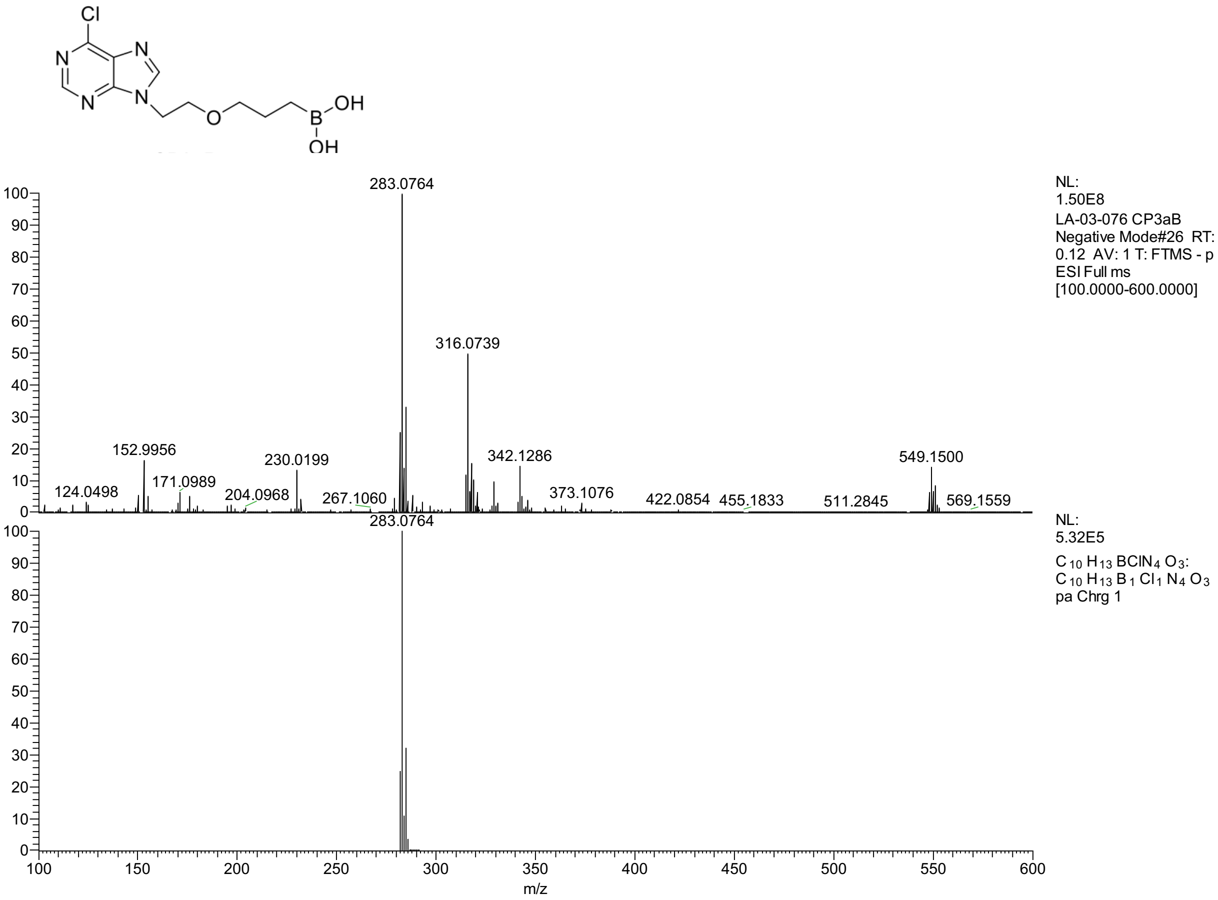

Figure S5 HRMS (ESI) m/z negative mode calculated for compound **3aCP** $C_{10}H_{14}BClN_4O_3$ - H (M - H)$^-$: 283.0764, found 283.0764.

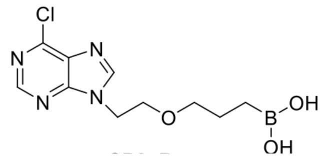

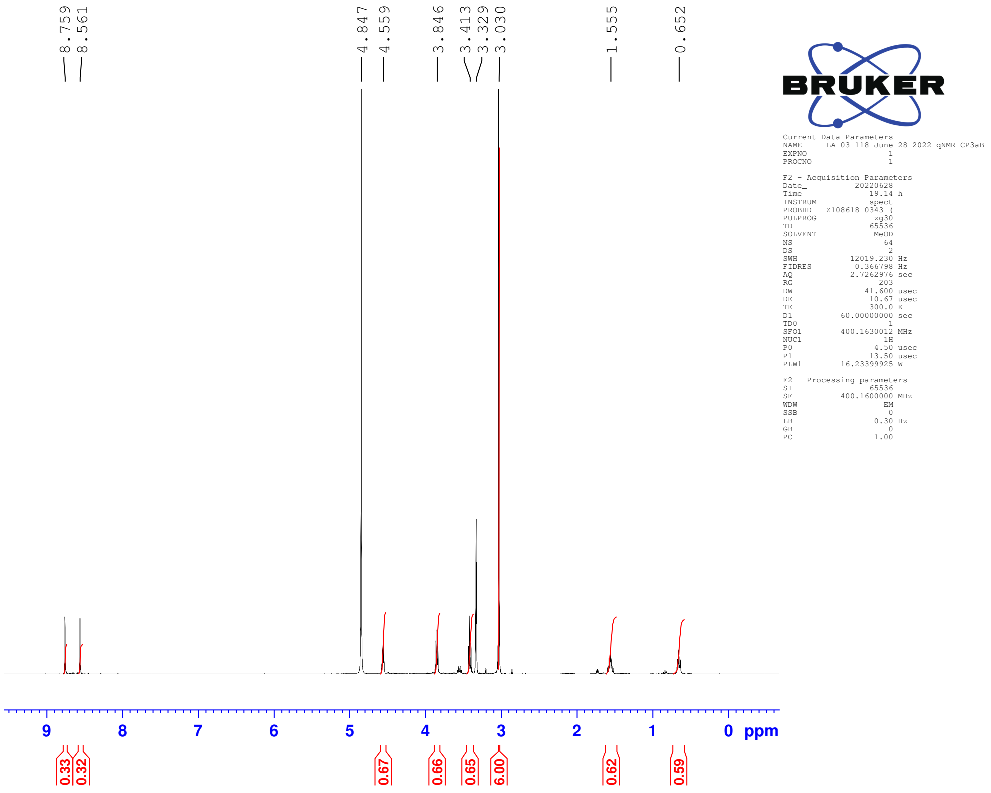

Figure S6 qNMR compound **3aCP**, internal standard DMS Purity 95.25%

$$P_{\text{sample}} = \frac{S_{\text{sample}} \text{ x } N_{\text{std}} \text{ x } m_{\text{std}} \text{ x } M_{\text{sample}}}{S_{\text{std}} \text{ x } N_{\text{sample}} \text{ x } m_{\text{sample}} \text{ x } M_{\text{std}}} \text{ x } P_{\text{std}}$$

$$= \frac{0.6682 \text{ x } 6 \text{ x } 8.6 \text{ mg x } 284.08 \text{ g mol}^{-1}}{6 \text{ x } 2 \text{ x } 9.1 \text{ mg x } 94.13 \text{ g mol}^{-1}} \text{ x } 99.96$$

$$= 95.25\%$$

S = Integrated area of the peak
N = Number of protons represented
m = Prepared mass
M = Molecular weight
P = Purity

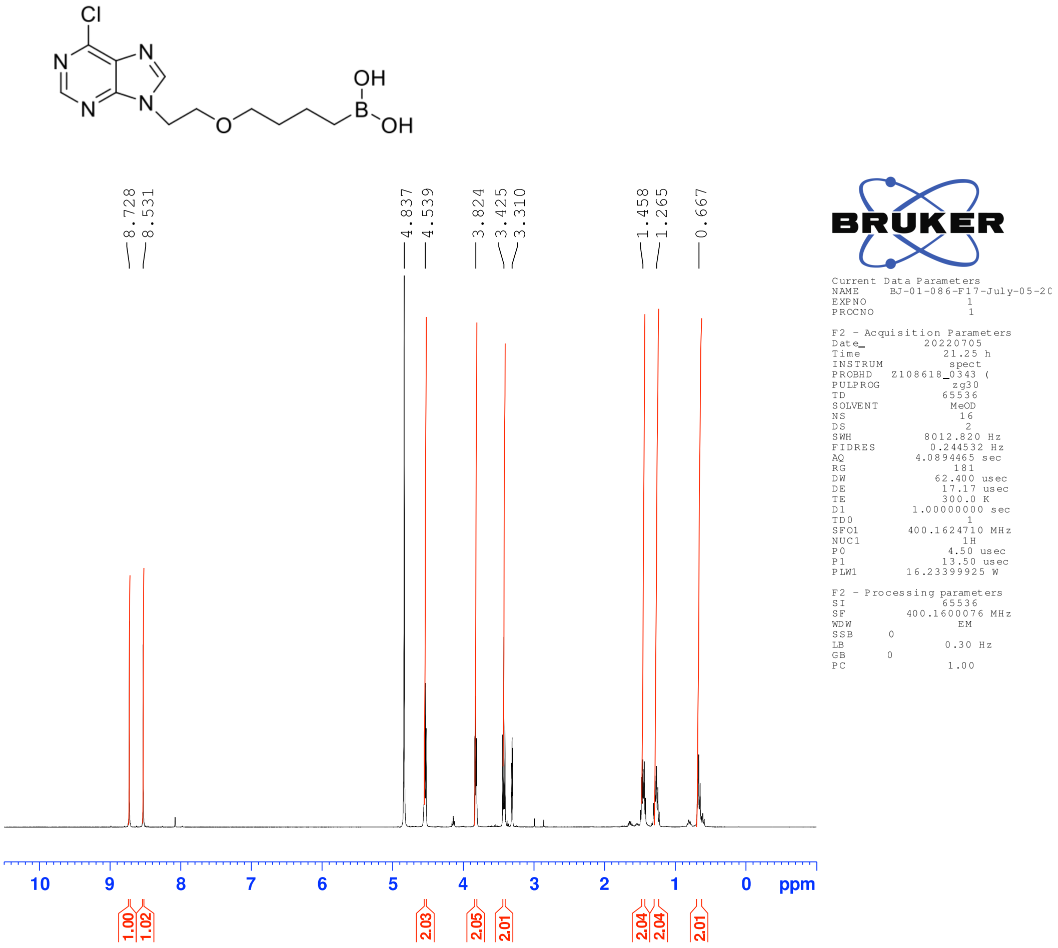

Figure S7 ^{1}HNMR spectra for compound **4aCP**

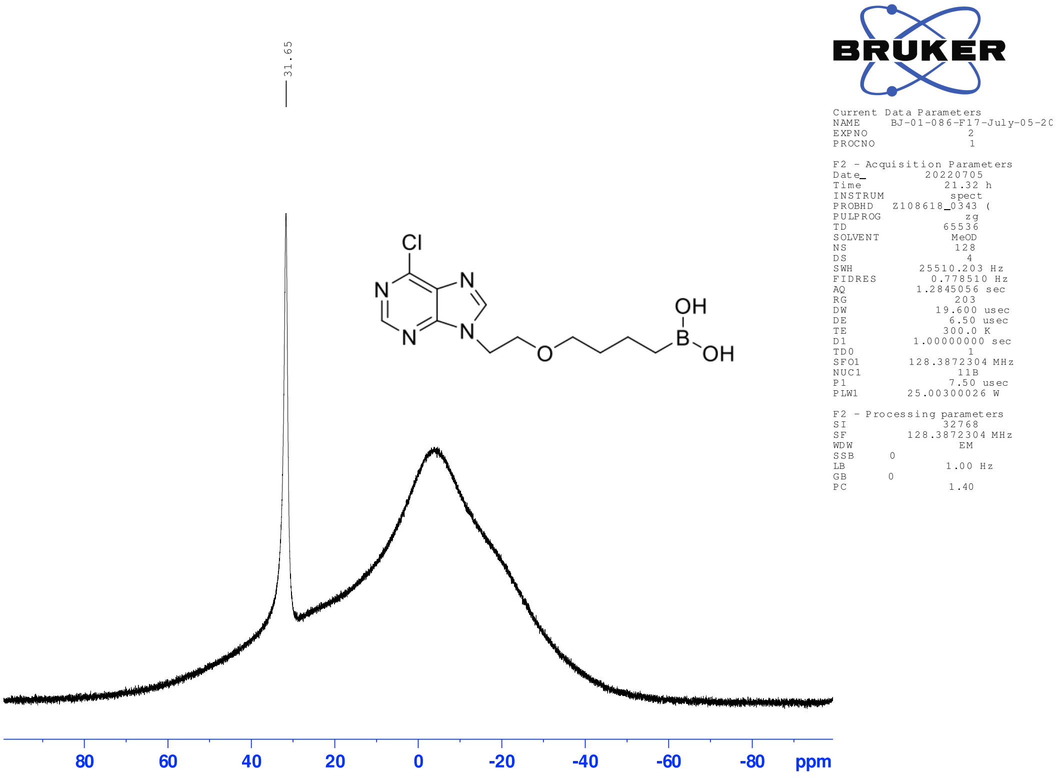

Figure S8 ^{11}BNMR spectra for compound **4aCP**

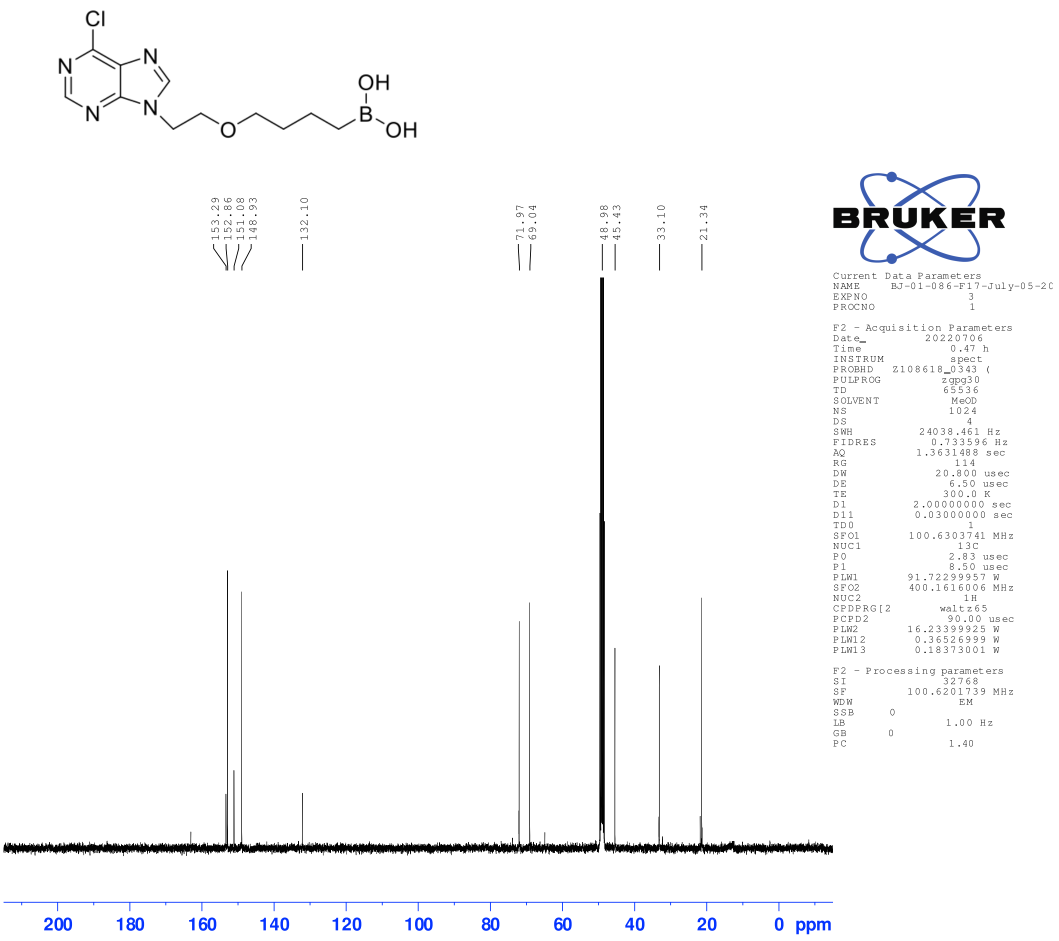

Figure S9 $^{13}C\{^{1}H\}$-NMR spectra for compound **4aCP**

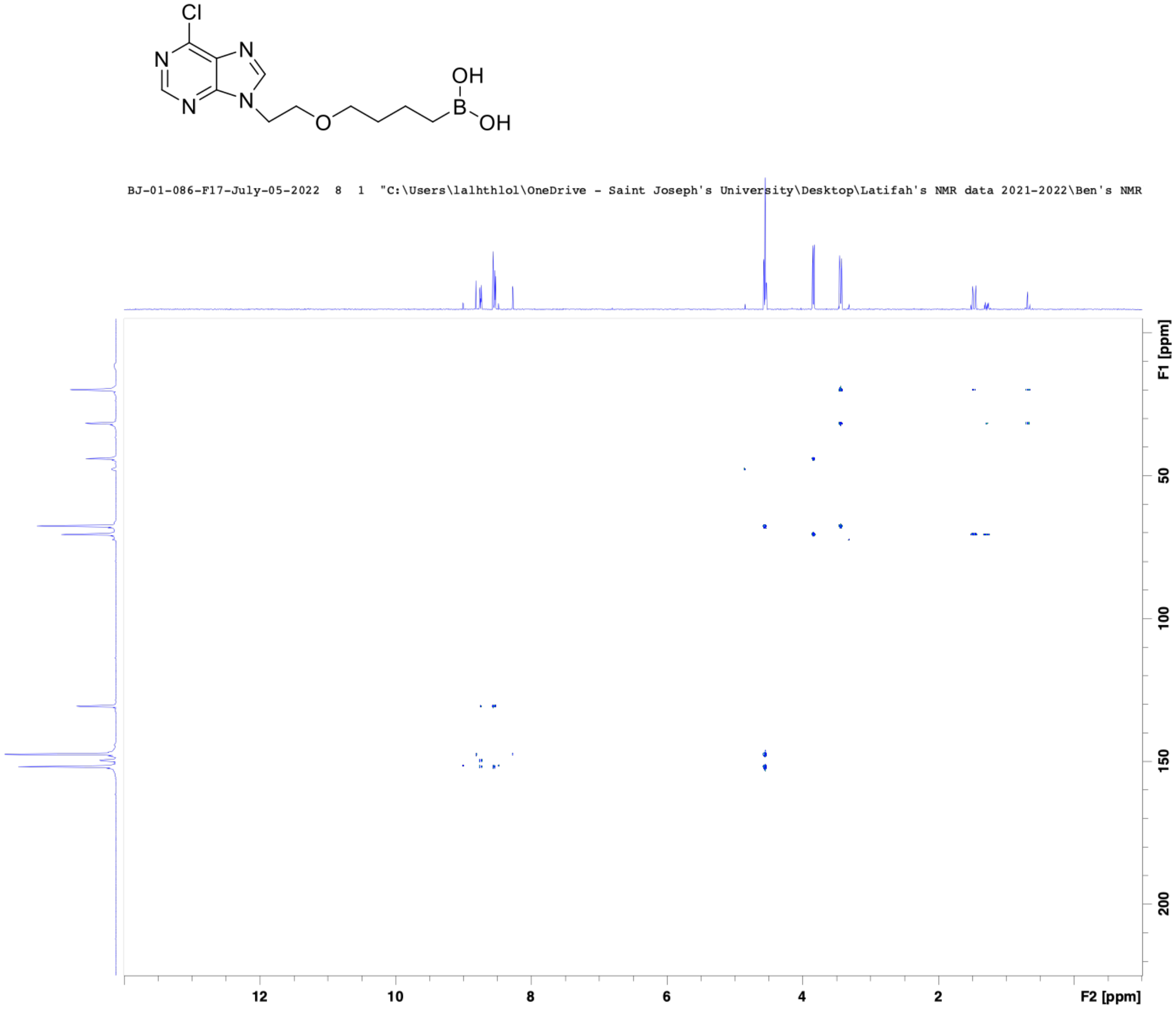

Figure S10 HMBC spectra for compound **4aCP**

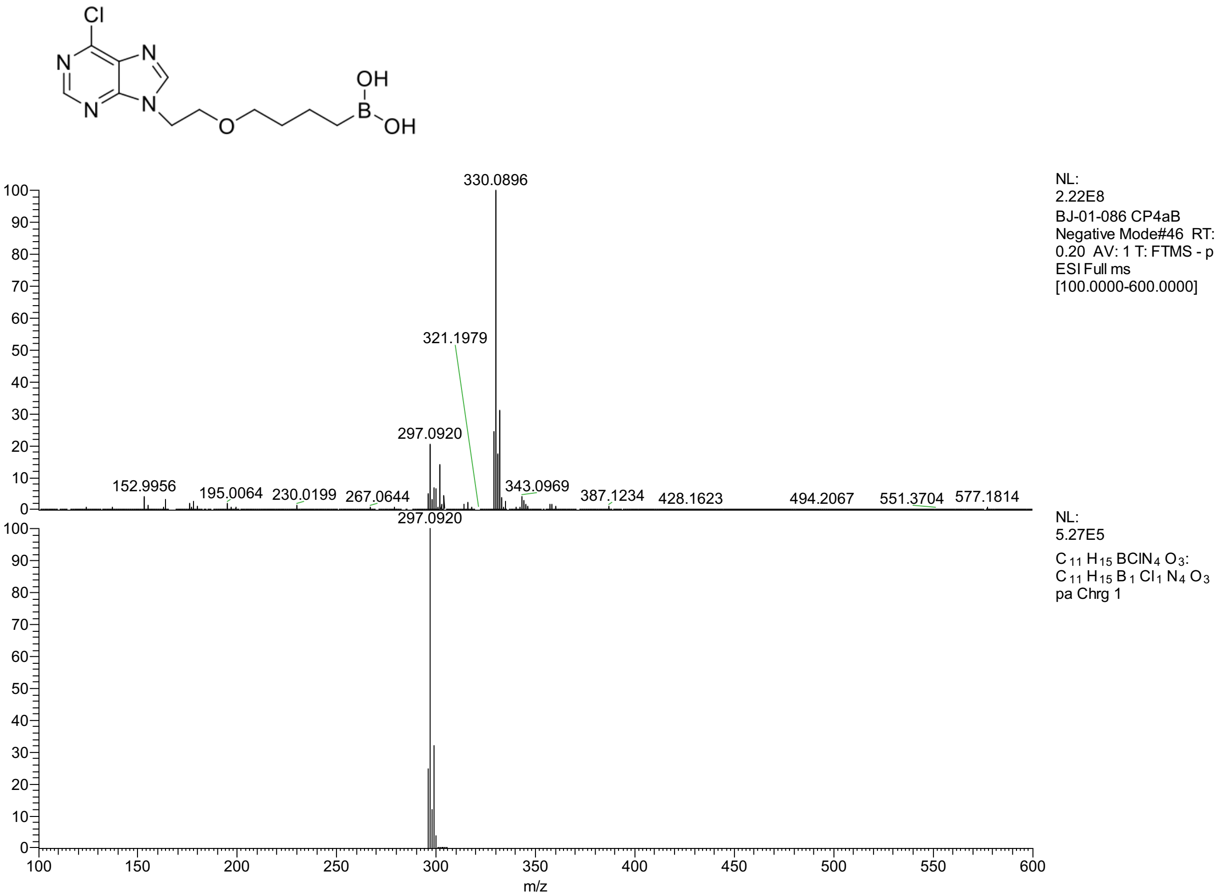

Figure S11 HRMS (ESI) m/z negative mode calculated for compound **4aCP** $C_{11}H_{16}BClN_4O_3$ - H (M - H)$^-$: 297.0920, found 297.0920.

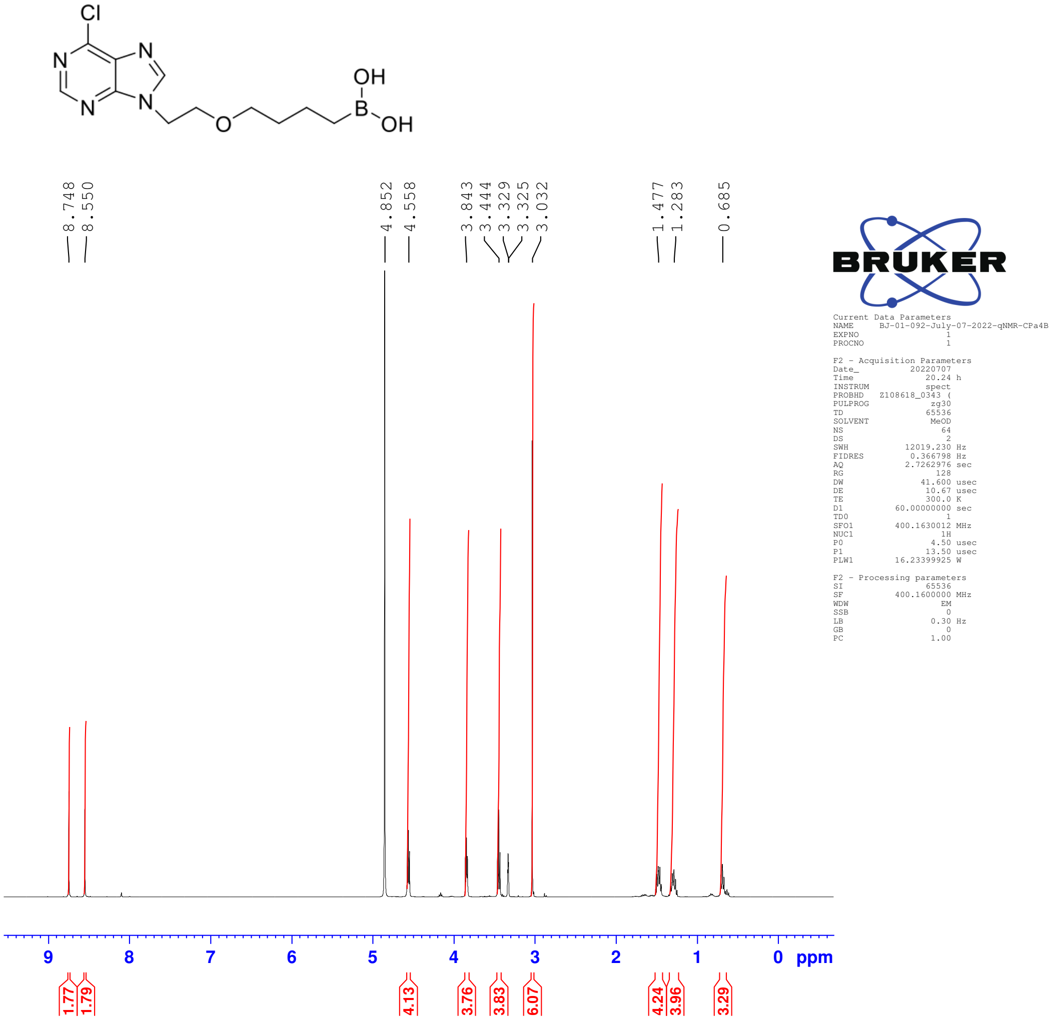

Figure S12 qNMR compound **4aCP**, internal standard DMS Purity 97.00%

$$P_{\text{sample}} = \frac{S_{\text{sample}} \text{ x } N_{\text{std}} \text{ x } m_{\text{std}} \text{ x } M_{\text{sample}}}{S_{\text{std}} \text{ x } N_{\text{sample}} \text{ x } m_{\text{sample}} \text{ x } M_{\text{std}}} \text{ x } P_{\text{std}}$$

$$= \frac{4.1311 \text{ x } 6 \text{ x } 2.7 \text{ mg x } 298.1 \text{ g mol}^{-1}}{6 \text{ x } 2 \text{ x } 18.2 \text{ mg x } 94.13 \text{ g mol}^{-1}} \text{ x } 99.96$$

$$= 97.00\%$$

S = Integrated area of the peak
N = Number of protons represented
m = Prepared mass
M = Molecular weight
P = Purity

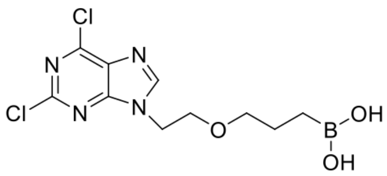

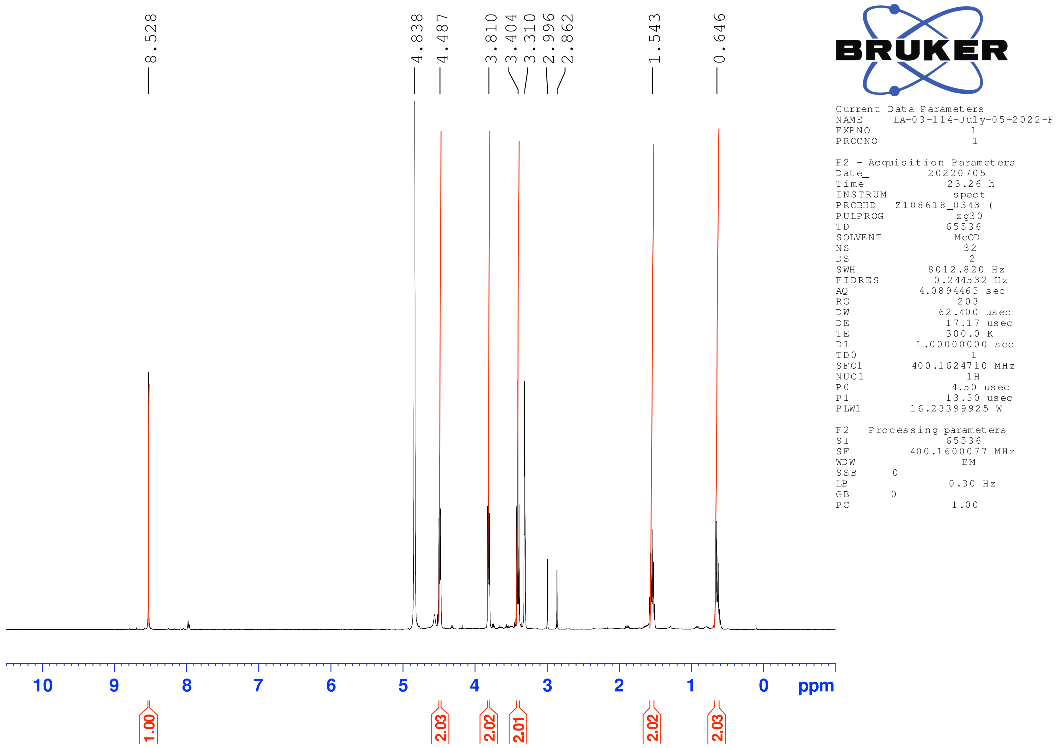

Figure S13 ^{1}HNMR spectra for compound **3aDCP**

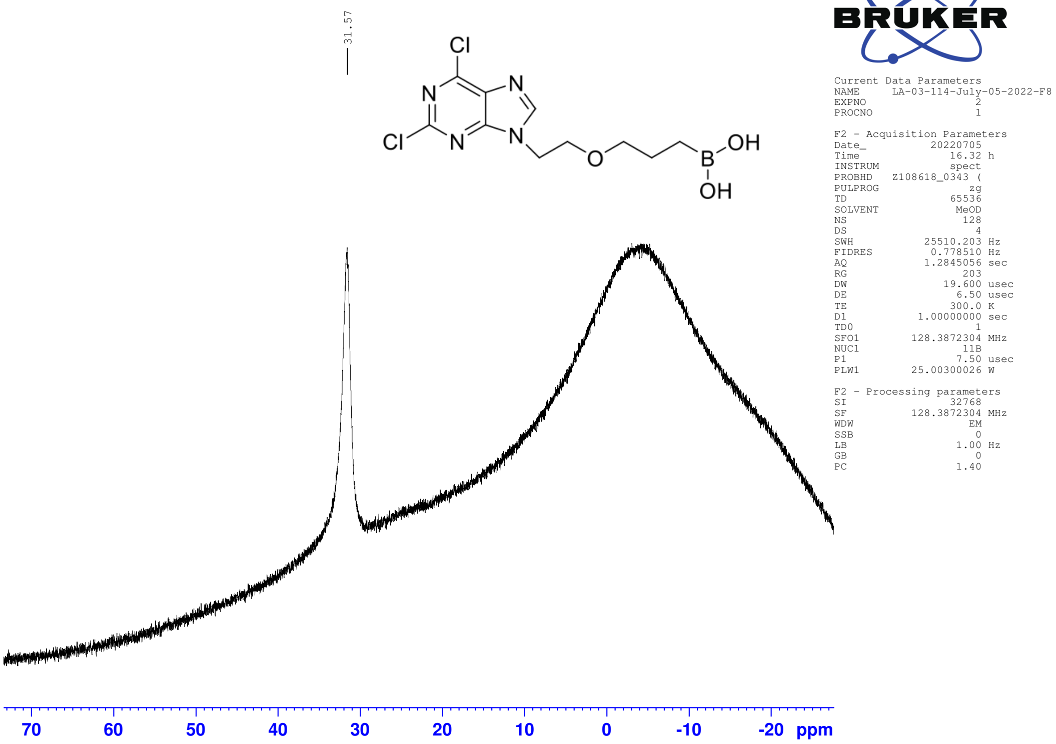

Figure S14 ^{11}BNMR spectra for compound **3aDCP**

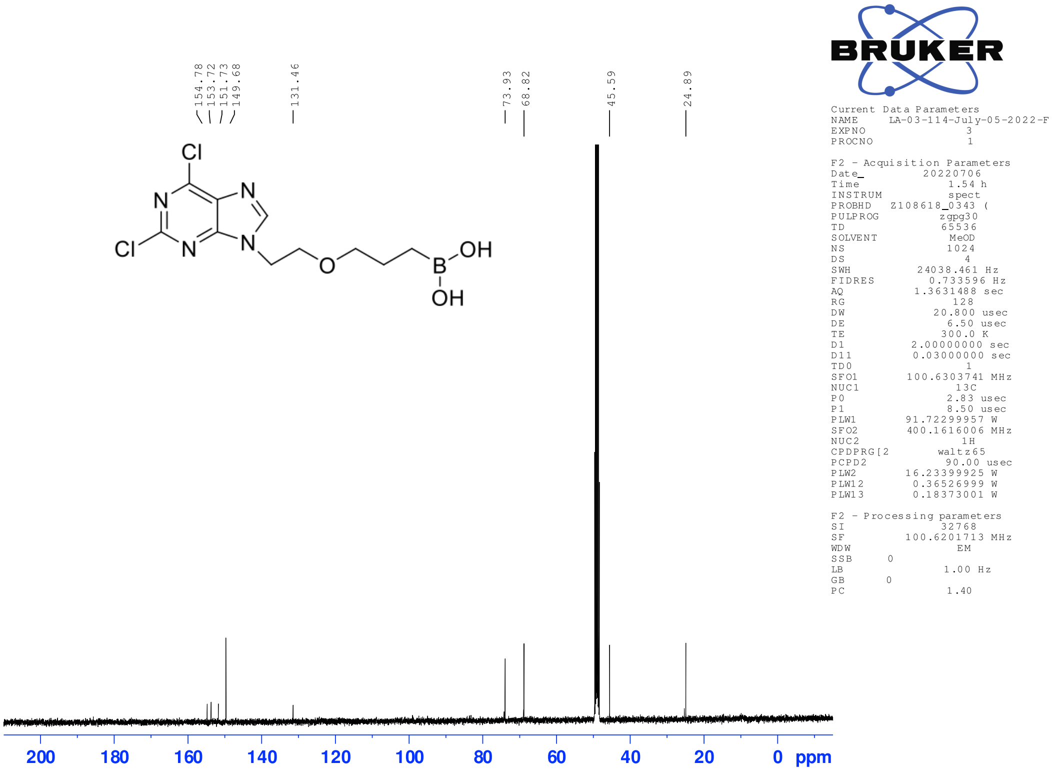

Figure S15 $^{13}C\{^{1}H\}$-NMR spectra for compound **3aDCP**

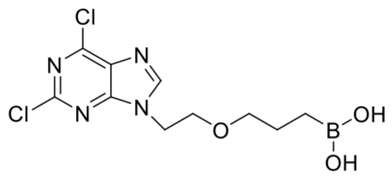

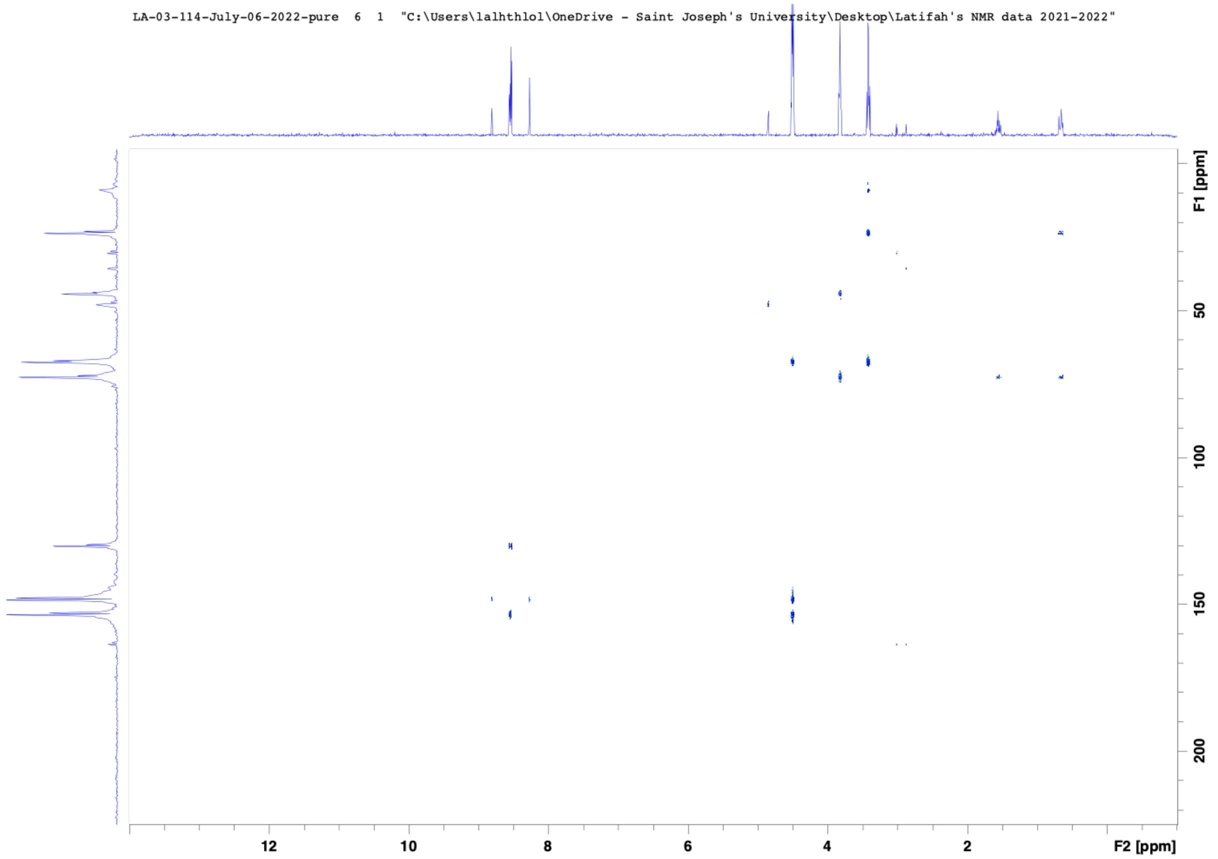

Figure S16 HMBC spectra for compound **3aDCP**

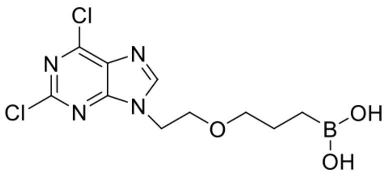

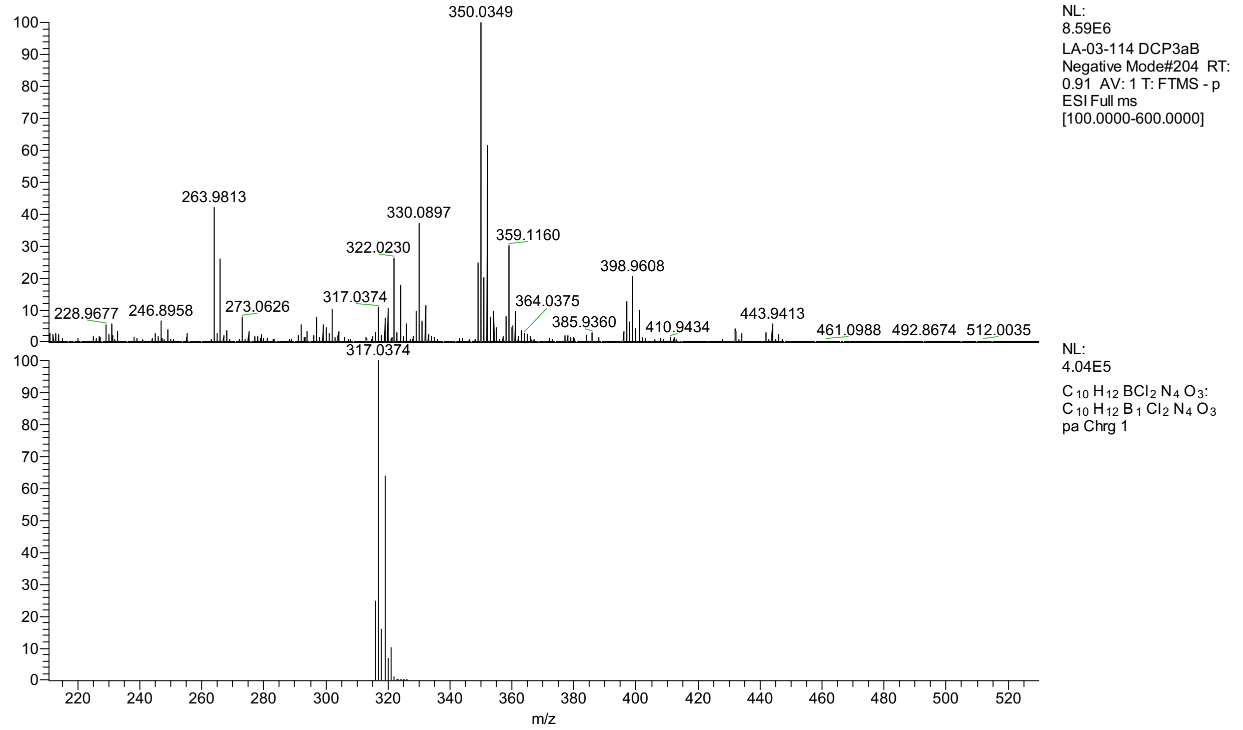

Figure S17 HRMS (ESI) m/z negative mode calculated for compound **3aDCP** $C_{10}H_{13}BCl_2N_4O$ - H (M - H)$^-$: 317.0374, found 317.0374.

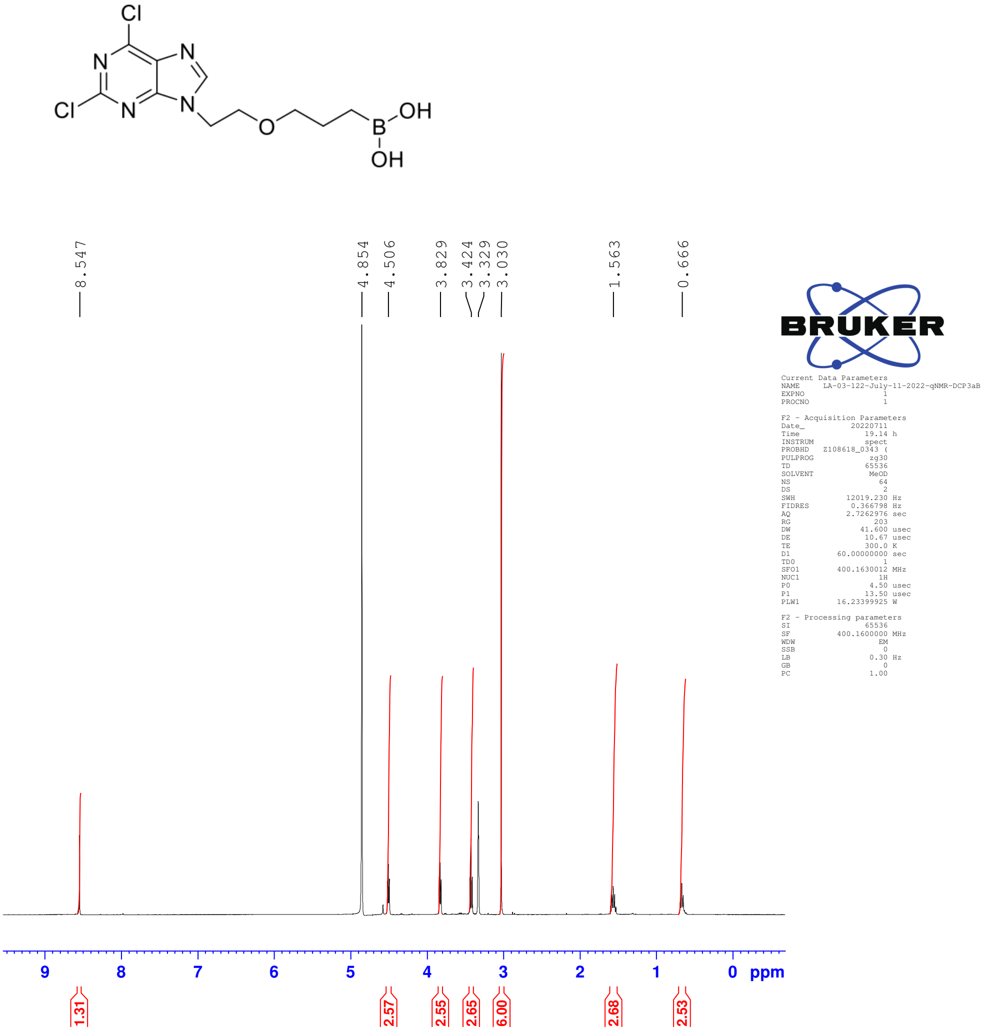

Figure S18 qNMR compound **3aDCP**, internal standard DMS Purity 96.02%

$$P_{\text{sample}} = \frac{S_{\text{sample}} \text{ x } N_{\text{std}} \text{ x } m_{\text{std}} \text{ x } M_{\text{sample}}}{S_{\text{std}} \text{ x } N_{\text{sample}} \text{ x } m_{\text{sample}} \text{ x } M_{\text{std}}} \text{ x } P_{\text{std}}$$

$$= \frac{2.55 \text{ x } 6 \text{ x } 1.65 \text{ mg x } 318.06 \text{ g mol}^{-1}}{6 \text{ x } 2 \text{ x } 7.4 \text{ mg x } 94.13 \text{ g mol}^{-1}} \text{ x } 99.96$$

$$= 96.02\%$$

S = Integrated area of the peak
N = Number of protons represented
m = Prepared mass
M = Molecular weight
P = Purity

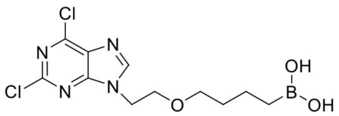

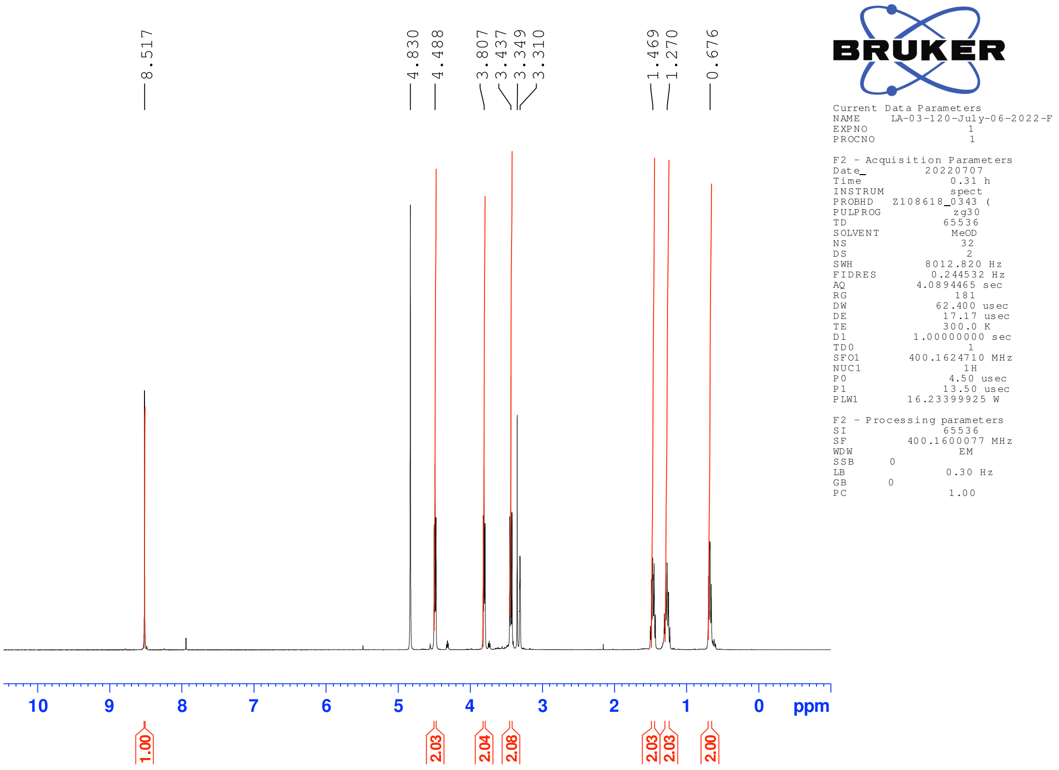

Figure S19 ^{1}HNMR spectra for compound **4aDCP**

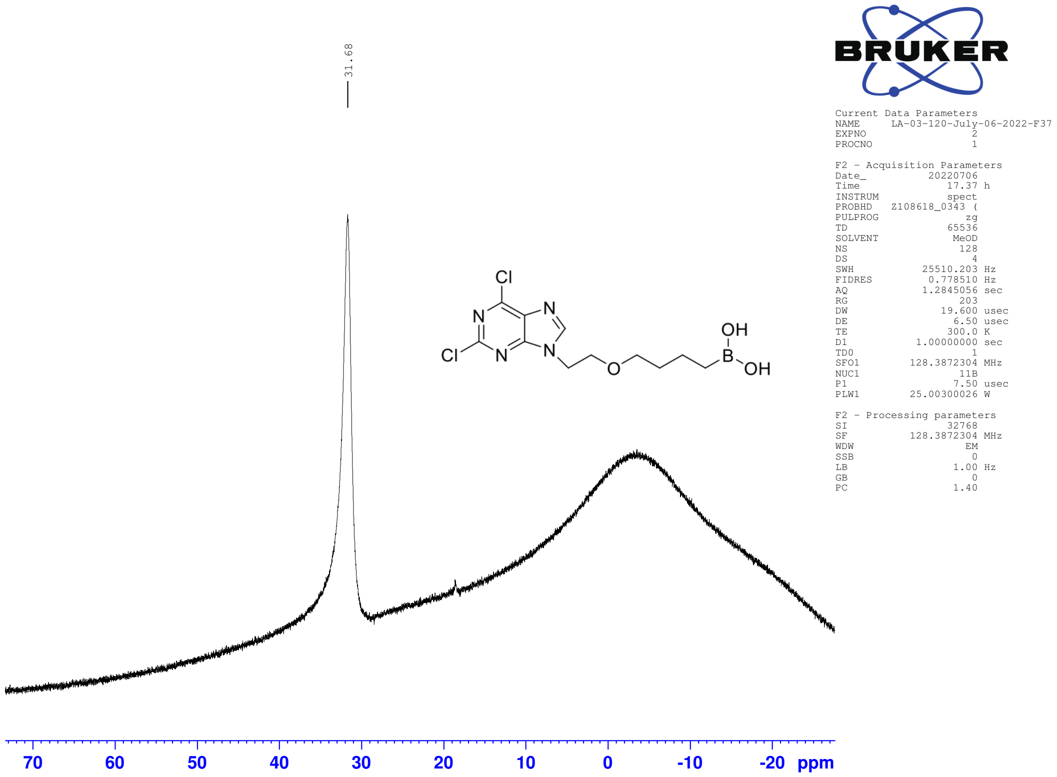

Figure S20 ^{11}BNMR spectra for compound **4aDCP**

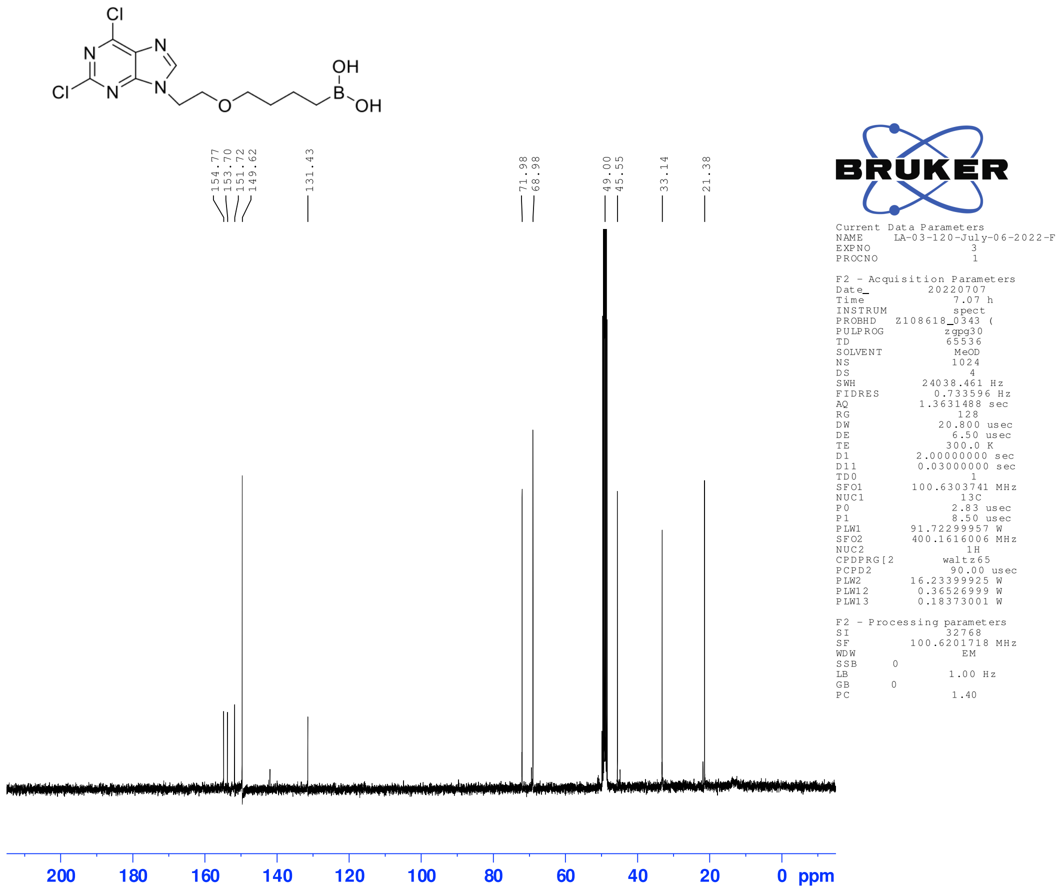

Figure S21 $^{13}C\{^{1}H\}$-NMR spectra for compound **4aDCP**

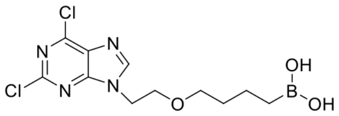

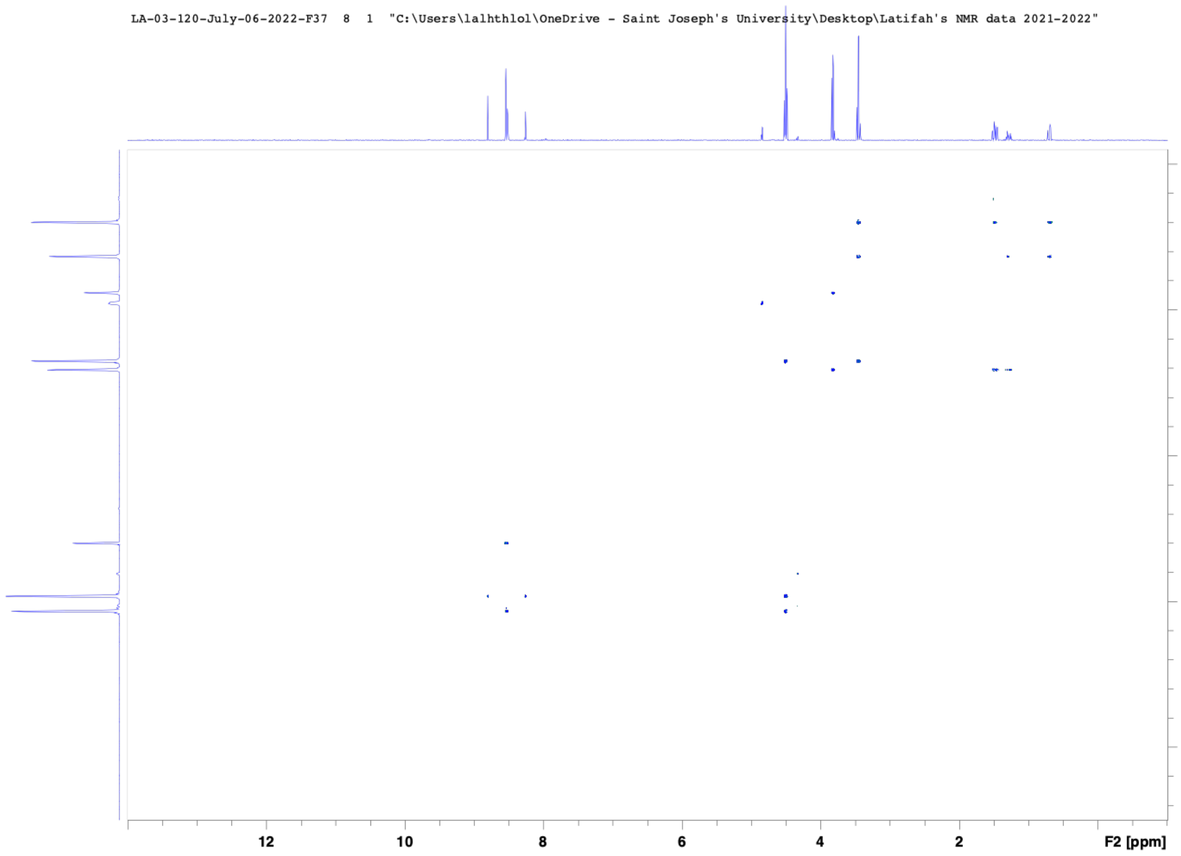

Figure S22 HMBC spectra for compound **4aDCP**

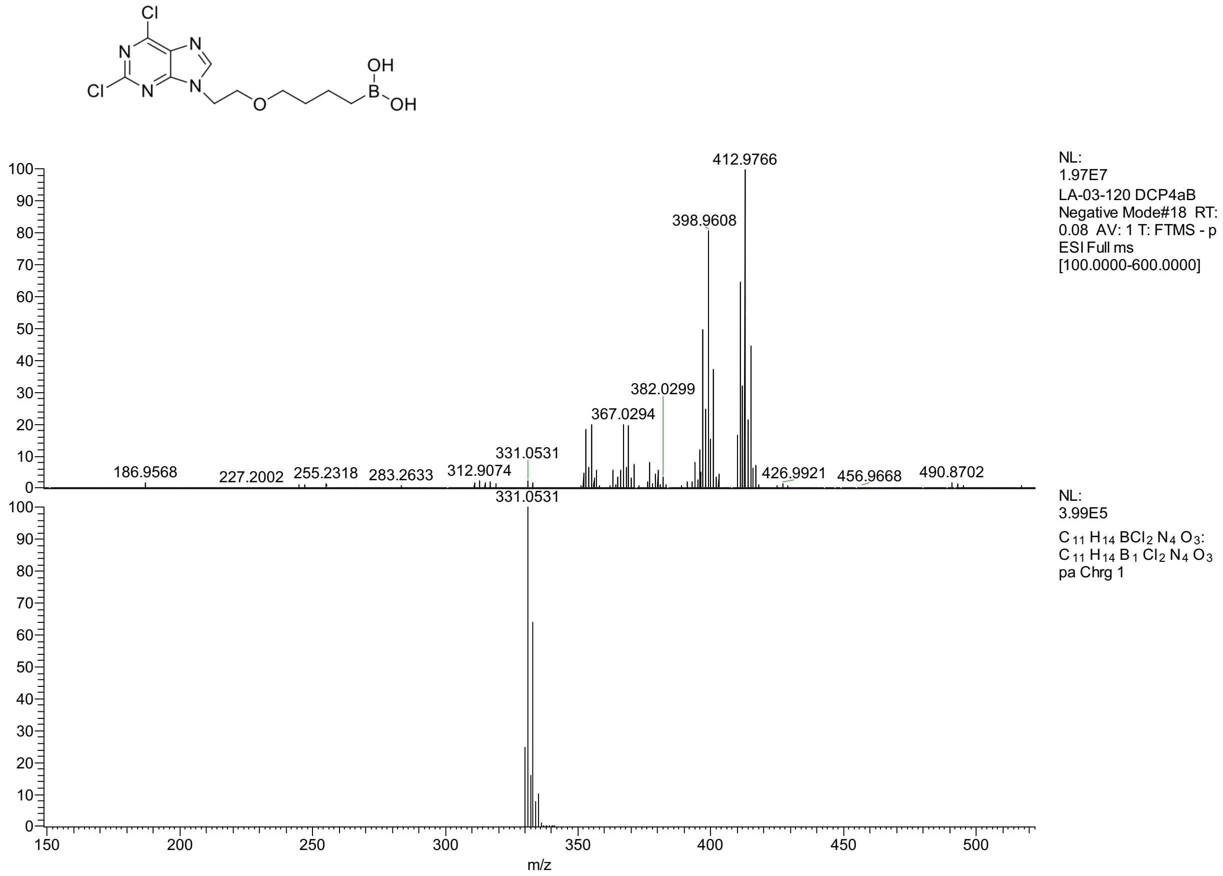

Figure S23 HRMS (ESI) m/z negative mode calculated for compound **4aDCP** $C_{11}H_{15}BCl_2N_4O_3$ - H (M - H)$^-$: 331.0531, found 331.0531.

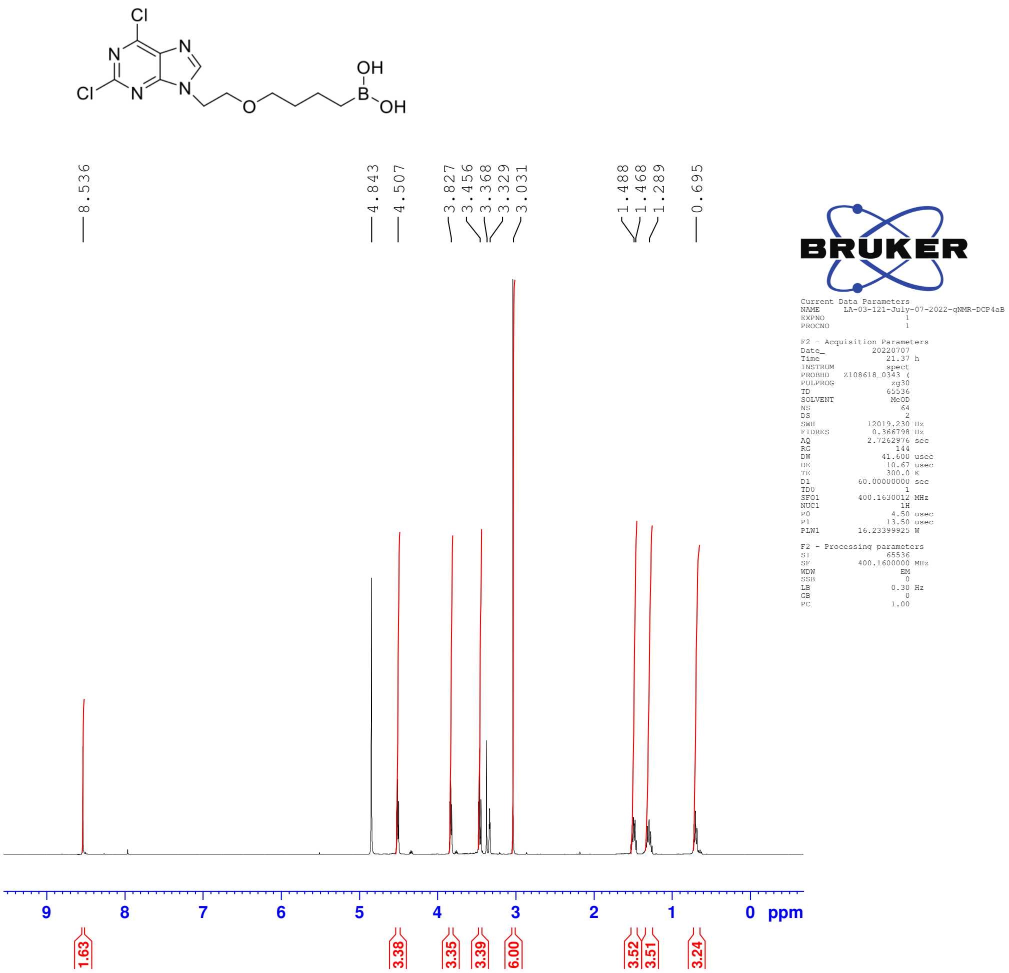

Figure S24 qNMR compound **4aDCP**, internal standard DMS Purity 96.73%

$$P_{\text{sample}} = \frac{S_{\text{sample}} \text{ x } N_{\text{std}} \text{ x } m_{\text{std}} \text{ x } M_{\text{sample}}}{S_{\text{std}} \text{ x } N_{\text{sample}} \text{ x } m_{\text{sample}} \text{ x } M_{\text{std}}} \text{ x } P_{\text{std}}$$

$$= \frac{3.3915 \text{ x } 6 \text{ x } 4.4 \text{ mg x } 332.06 \text{ g mol}^{-1}}{6 \text{ x } 2 \text{ x } 27.2 \text{ mg x } 94.13 \text{ g mol}^{-1}} \text{ x } 99.96$$

$$= 96.73\%$$

S = Integrated area of the peak
N = Number of protons represented
m = Prepared mass
M = Molecular weight
P = Purity

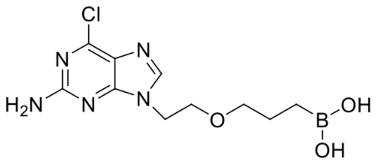

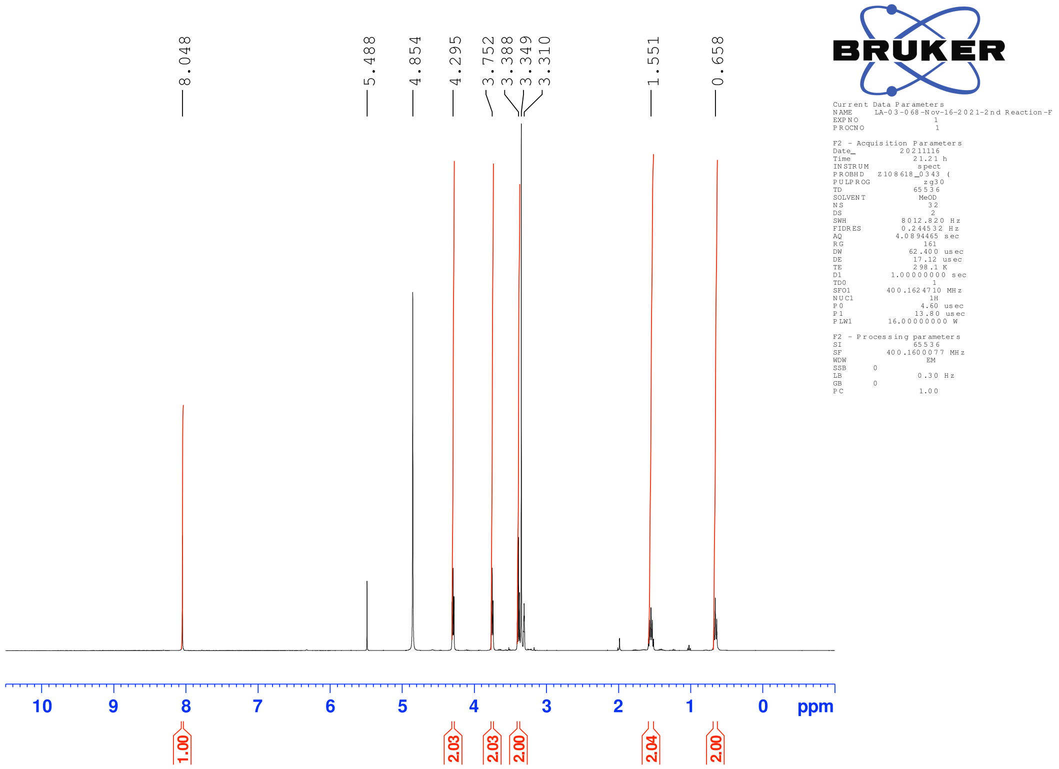

Figure S25 ^{1}HNMR spectra for compound **3aACP**

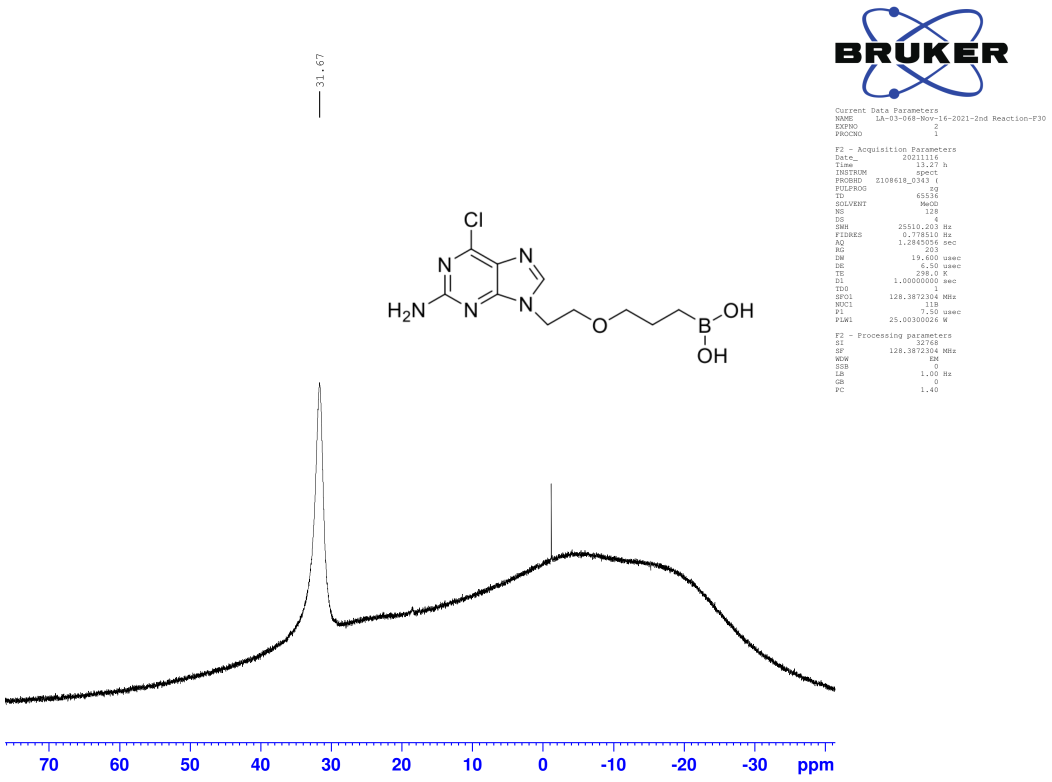

Figure S26 ^{11}BNMR spectra for compound **3aACP**

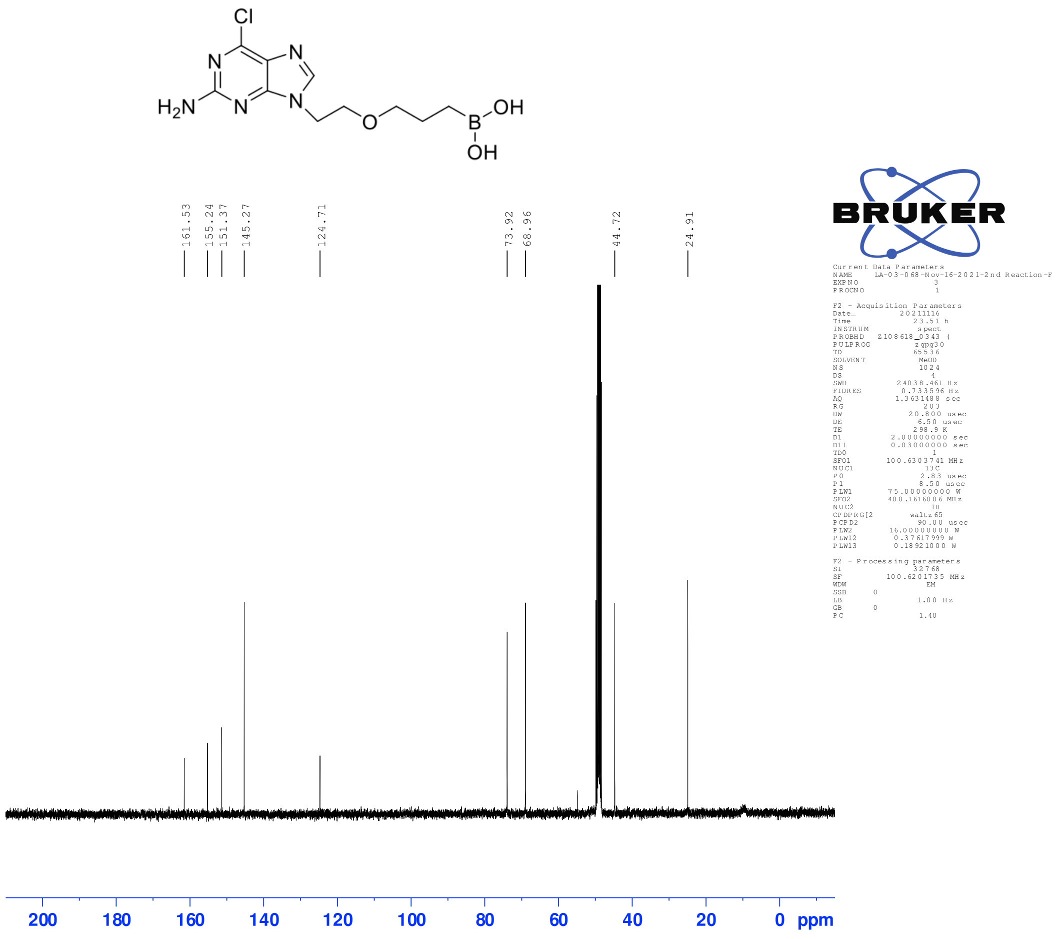

Figure S27 $^{13}C\{^{1}H\}$-NMR spectra for compound **3aACP**

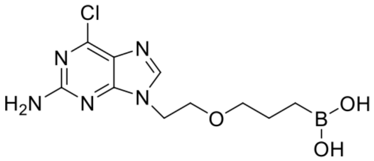

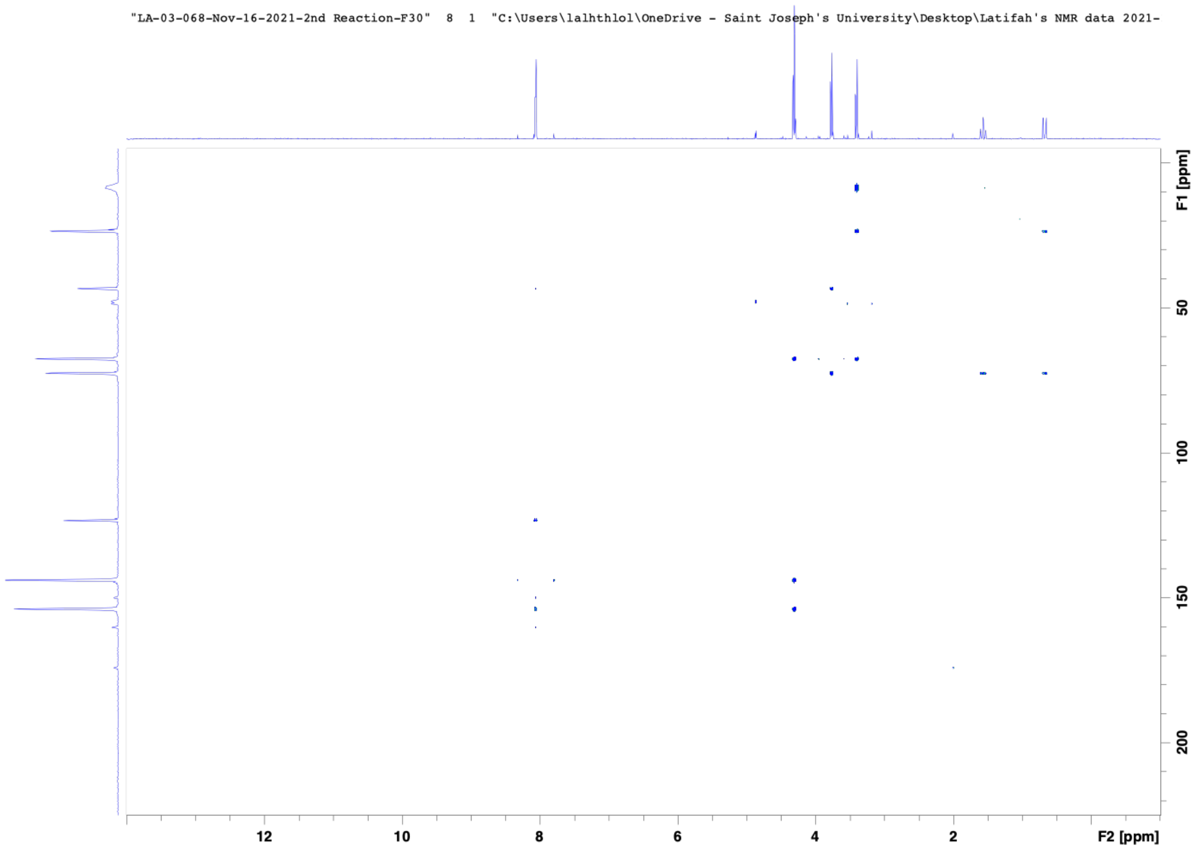

Figure S28 HMBC spectra for compound **3aACP**

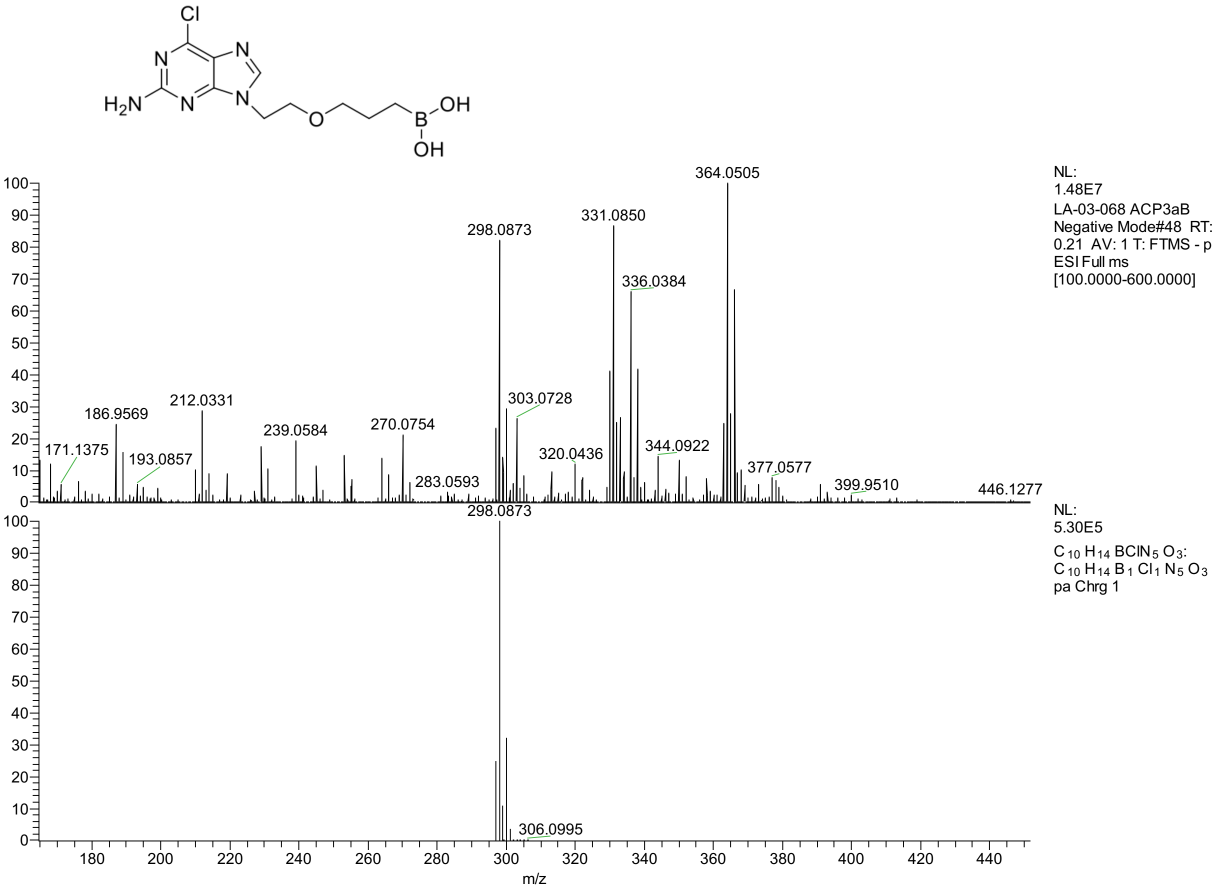

Figure S29 HRMS (ESI) m/z negative mode calculated for compound **3aACP** $C_{10}H_{15}BClN_5O_3$ - H (M - H)$^-$: 298.0873, found 298.0873.

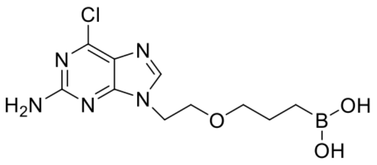

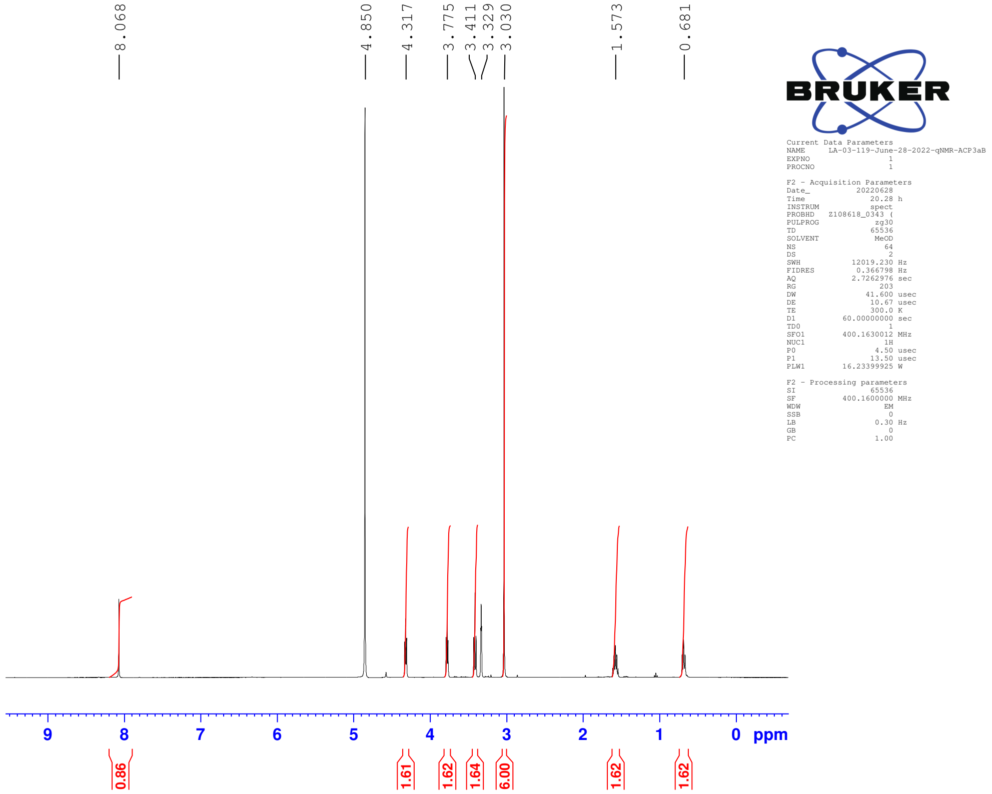

Figure S30 qNMR compound **3aACP**, internal standard DMS Purity 96.87%

$$P_{\text{sample}} = \frac{S_{\text{sample}} \text{ x } N_{\text{std}} \text{ x } m_{\text{std}} \text{ x } M_{\text{sample}}}{S_{\text{std}} \text{ x } N_{\text{sample}} \text{ x } m_{\text{sample}} \text{ x } M_{\text{std}}} \text{ x } P_{\text{std}}$$

$$= \frac{1.6353 \text{ x } 6 \text{ x } 4.7 \text{ mg x } 299.1 \text{ g mol}^{-1}}{6 \text{ x } 2 \text{ x } 12.6 \text{ mg x } 94.13 \text{ g mol}^{-1}} \text{ x } 99.96$$

$$= 96.87\%$$

S = Integrated area of the peak

N = Number of protons represented

m = Prepared mass

M = Molecular weight

P = Purity

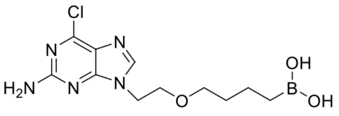

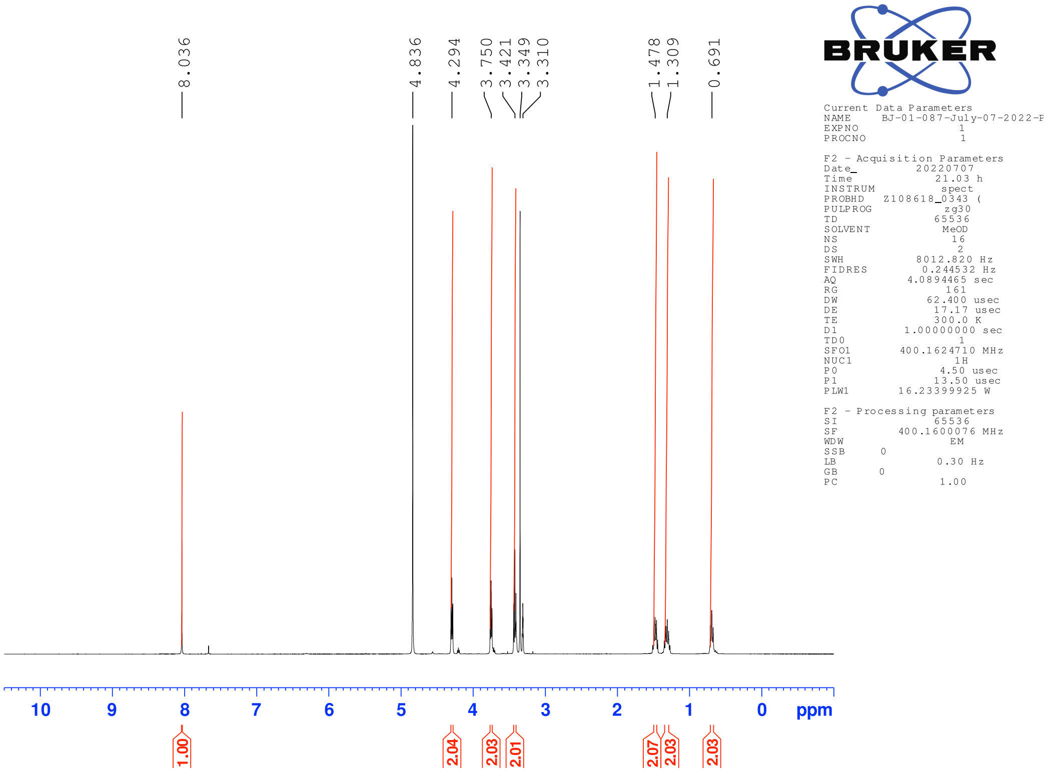

Figure S31 ^{1}HNMR spectra for compound **4aACP**

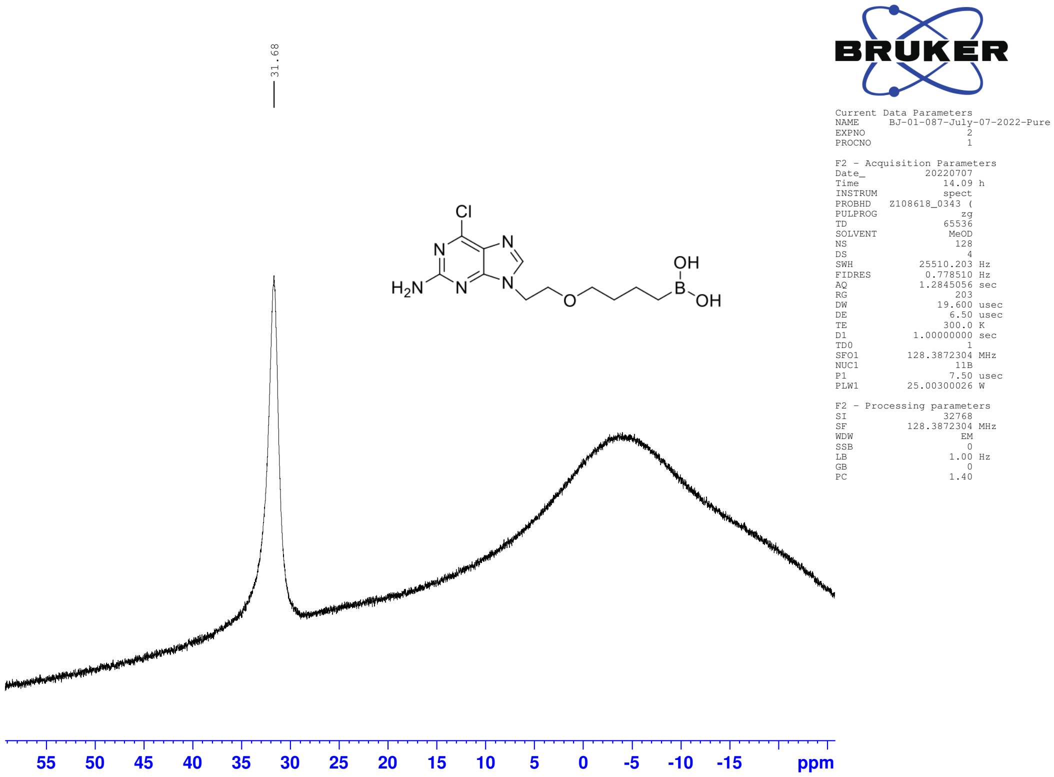

Figure S32 ^{11}BNMR spectra for compound **4aACP**

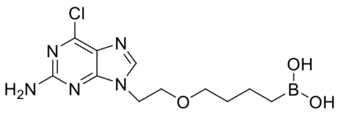

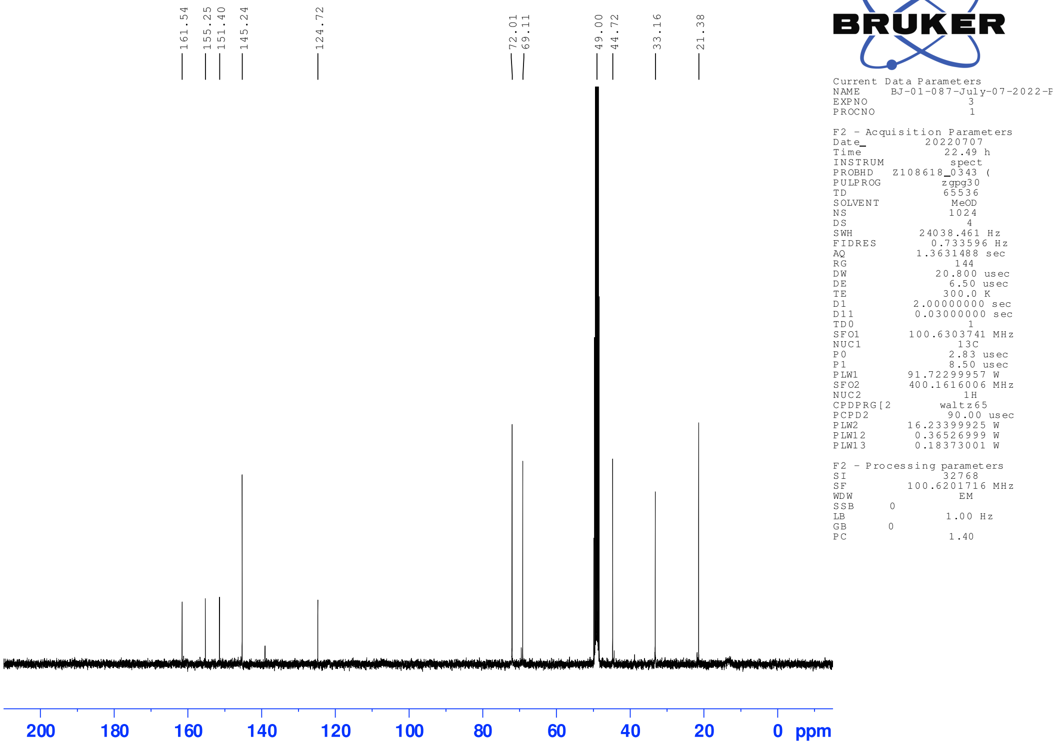

Figure S33 $^{13}C\{^{1}H\}$-NMR spectra for compound **4aACP**

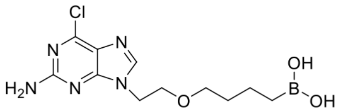

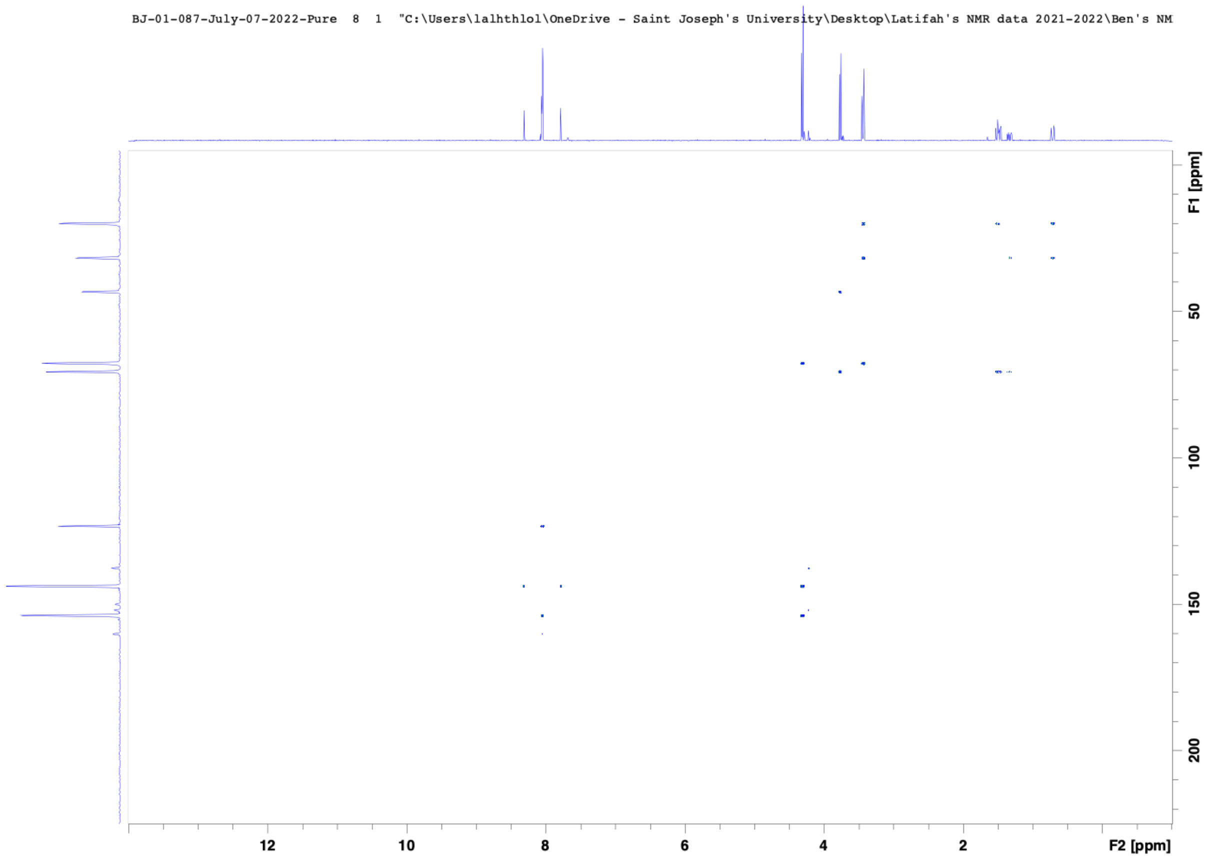

Figure S34 HMBC spectra for compound **4aACP**

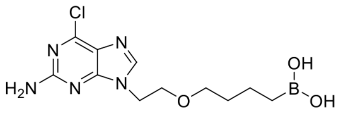

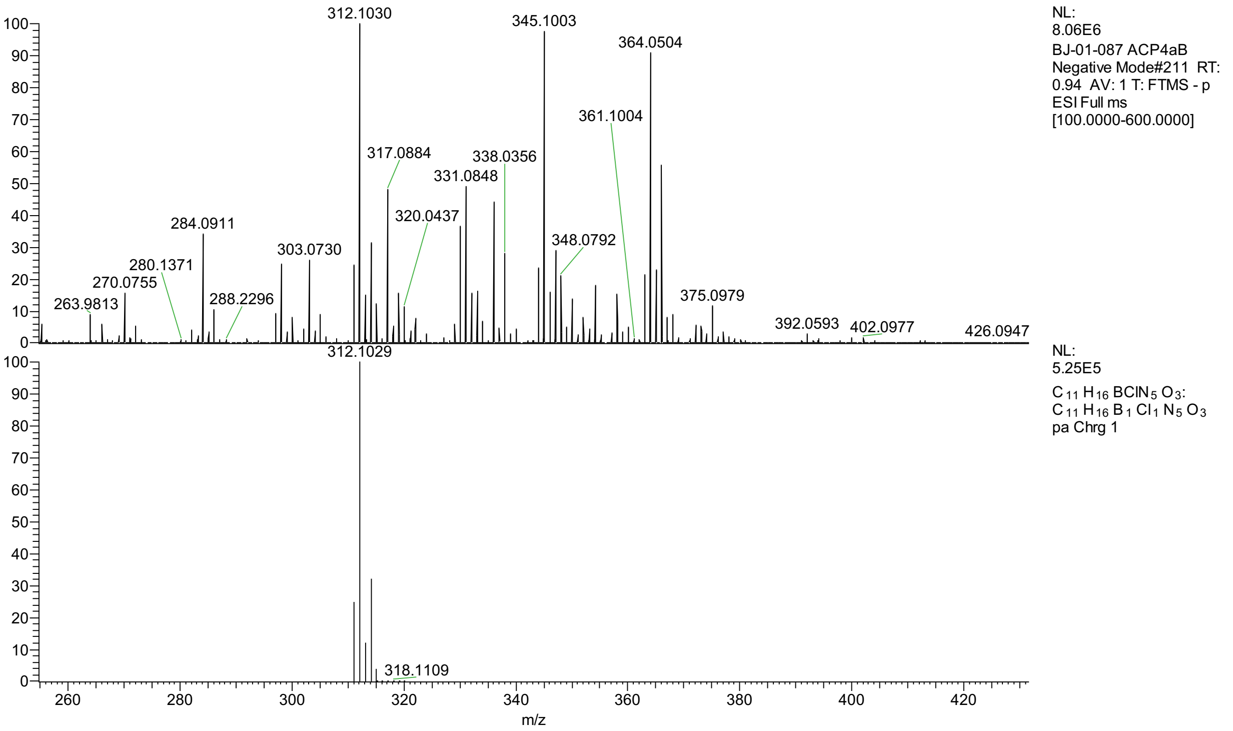

Figure S35 HRMS (ESI) m/z negative mode calculated for compound **4aACP** $C_{11}H_{17}BClN_5O_3$ - H (M - H)$^-$: 312.1029, found 312.1030.

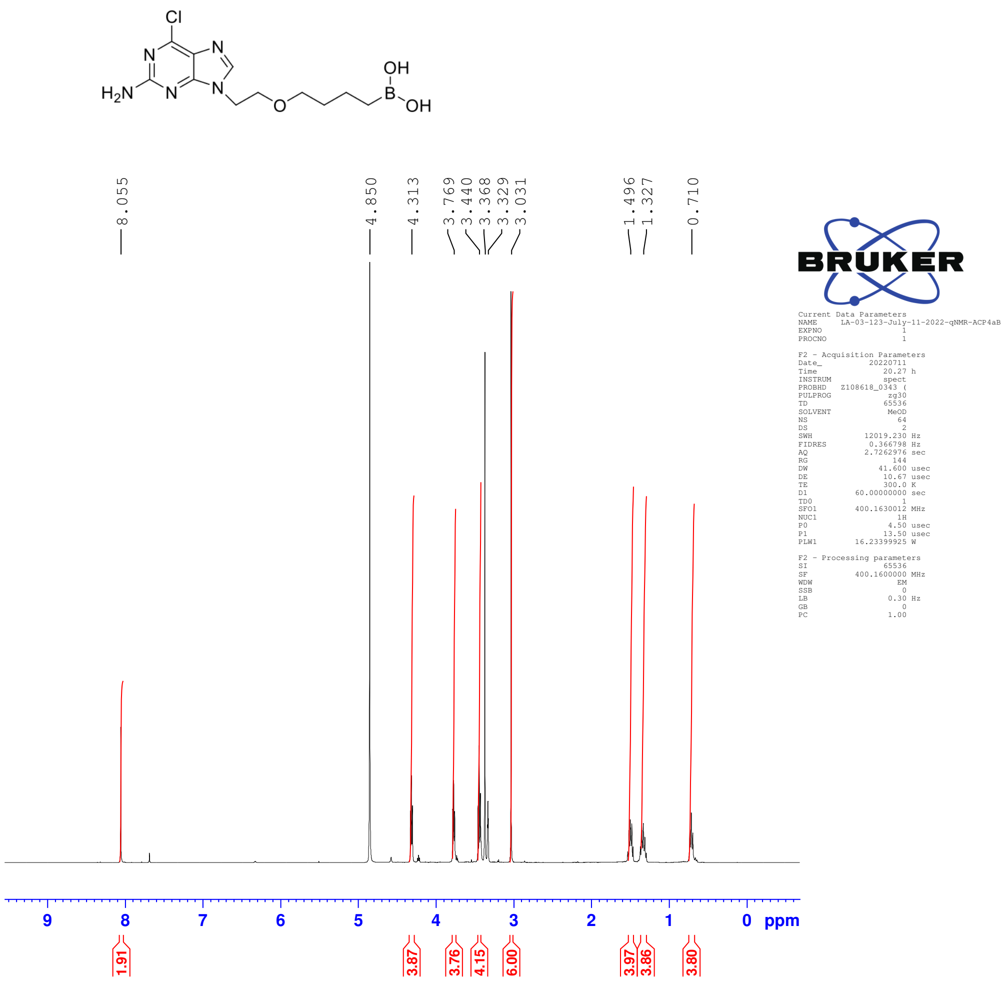

Figure S36 qNMR compound **4aACP**, internal standard DMS Purity 97.20%

$$P_{\text{sample}} = \frac{S_{\text{sample}} \text{ x } N_{\text{std}} \text{ x } m_{\text{std}} \text{ x } M_{\text{sample}}}{S_{\text{std}} \text{ x } N_{\text{sample}} \text{ x } m_{\text{sample}} \text{ x } M_{\text{std}}} \text{ x } P_{\text{std}}$$

$$= \frac{3.9673 \text{ x } 6 \text{ x } 2.8 \text{ mg x } 313.11 \text{ g mol}^{-1}}{6 \text{ x } 2 \text{ x } 19.0 \text{ mg x } 94.13 \text{ g mol}^{-1}} \text{ x } 99.96$$

$$= 97.20\%$$

S = Integrated area of the peak
N = Number of protons represented
m = Prepared mass
M = Molecular weight
P = Purity

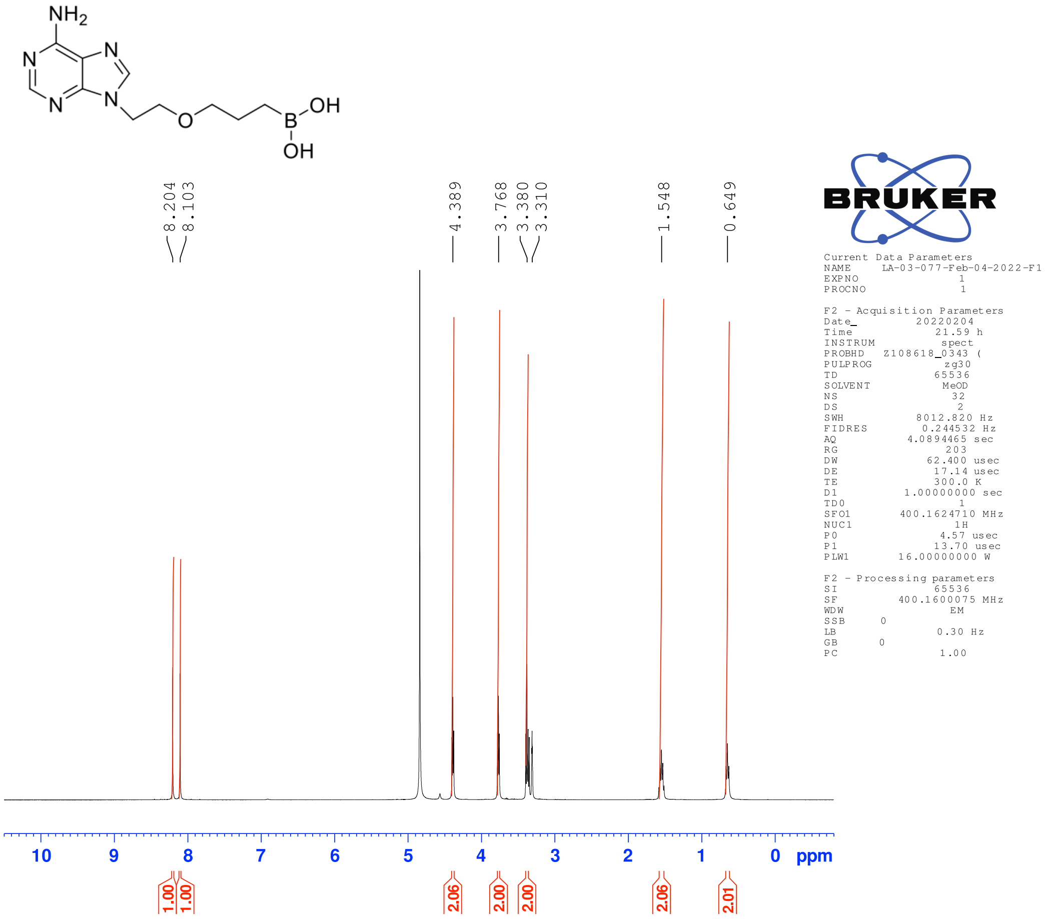

Figure S37 ^{1}HNMR spectra for compound **3aA**

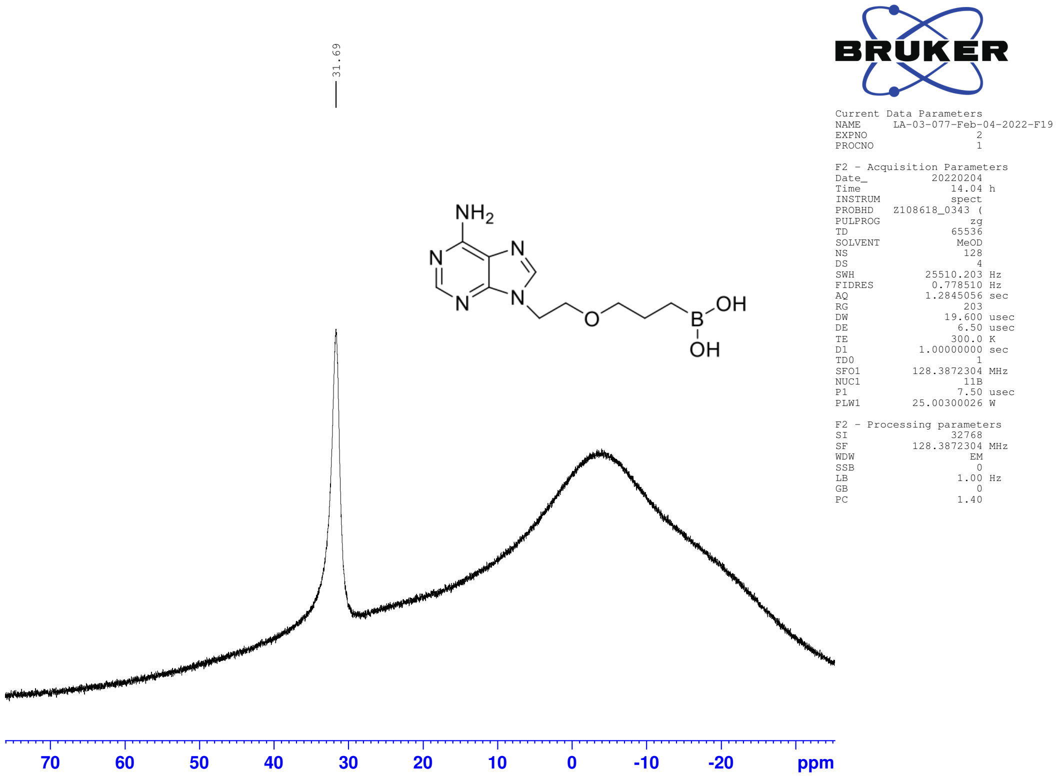

Figure S38 ^{11}BNMR spectra for compound **3aA**

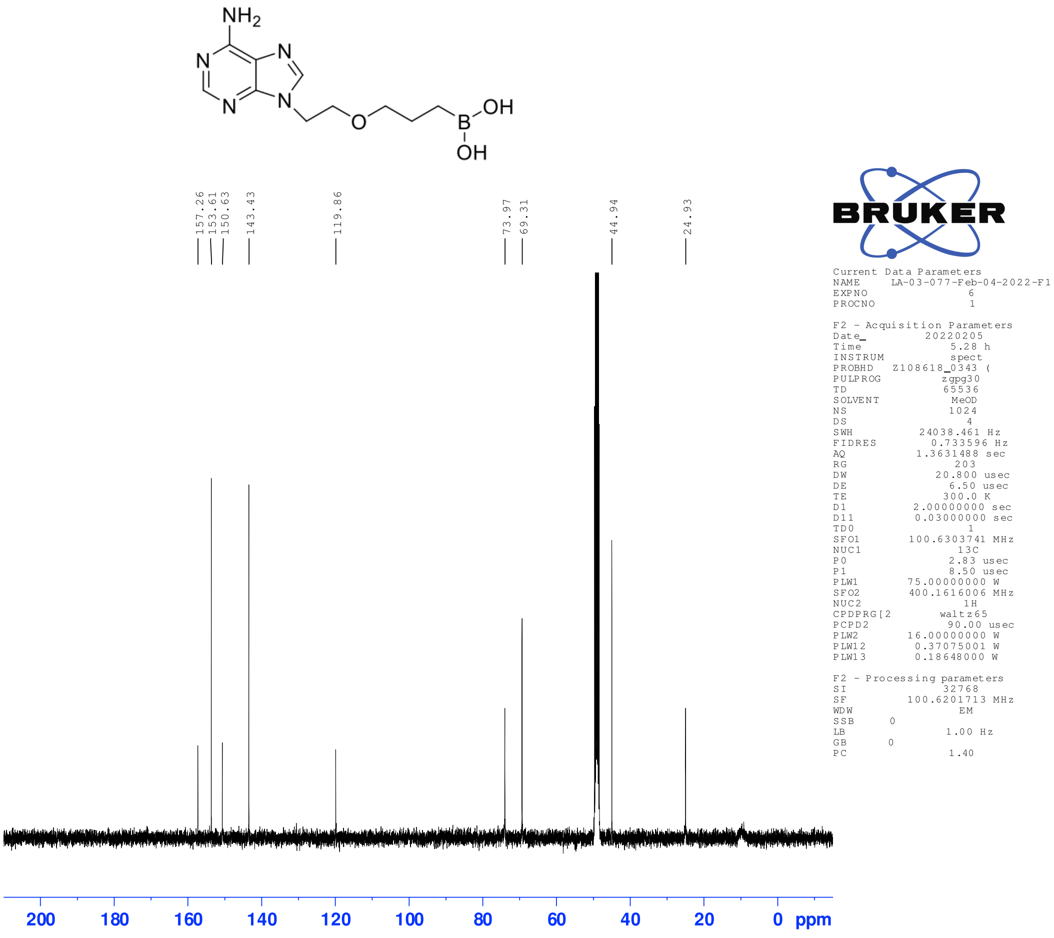

Figure S39 $^{13}C\{^{1}H\}$-NMR spectra for compound **3aA**

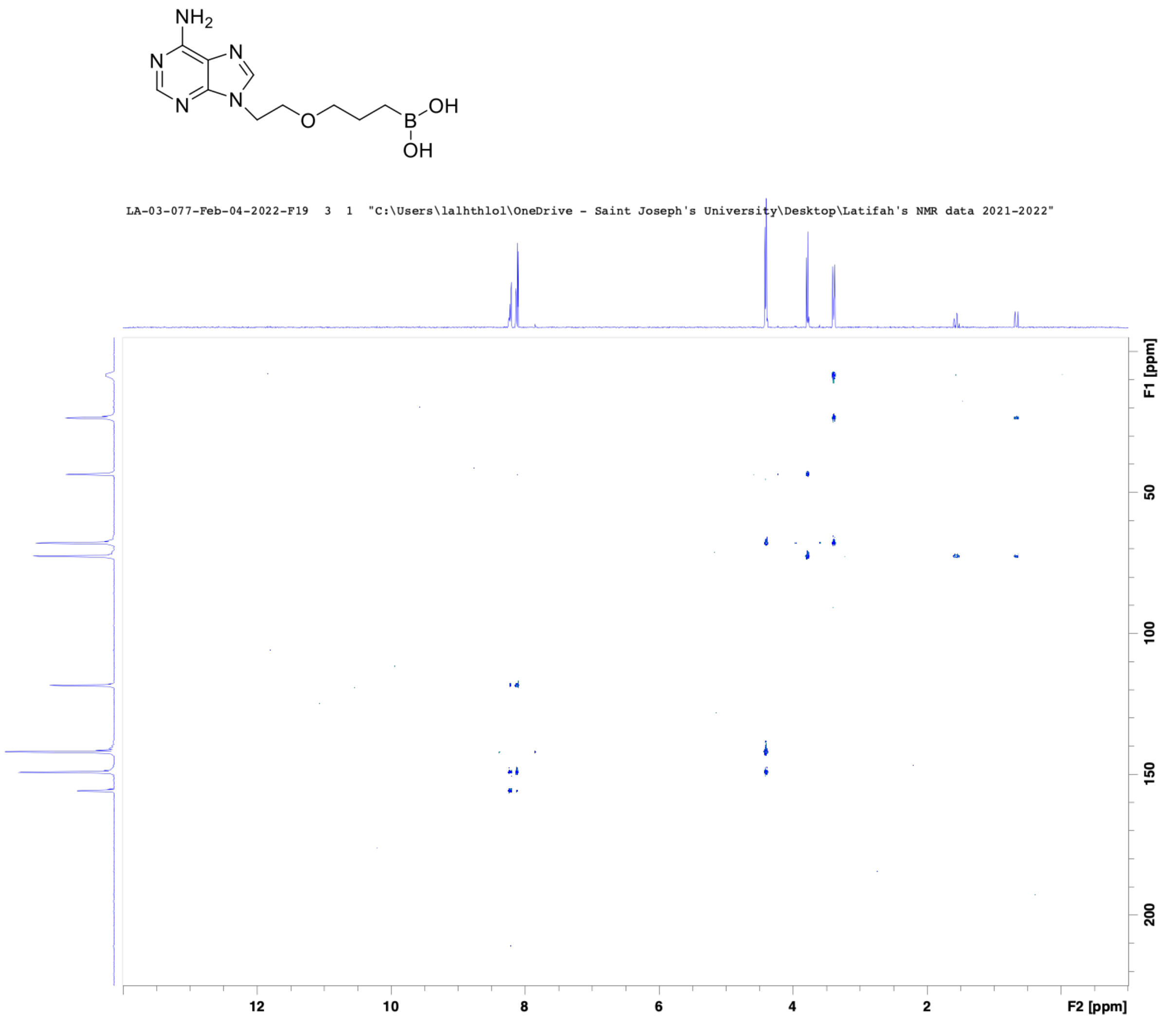

Figure S40 HMBC spectra for compound **3aA**

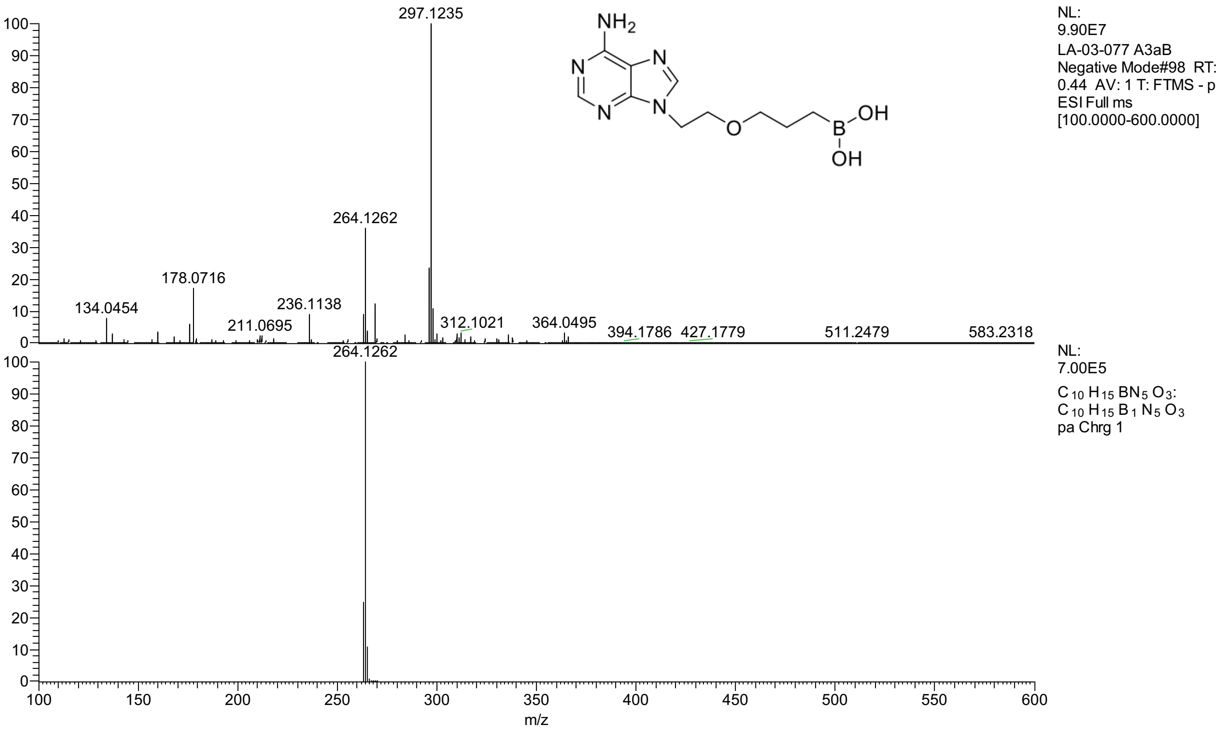

Figure S41 HRMS (ESI) m/z negative mode calculated for compound **3aA** $C_{10}H_{16}BN_5O_3$ - H (M - H)$^-$: 264.1262, found 264.1262.

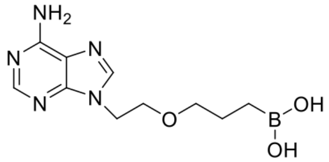

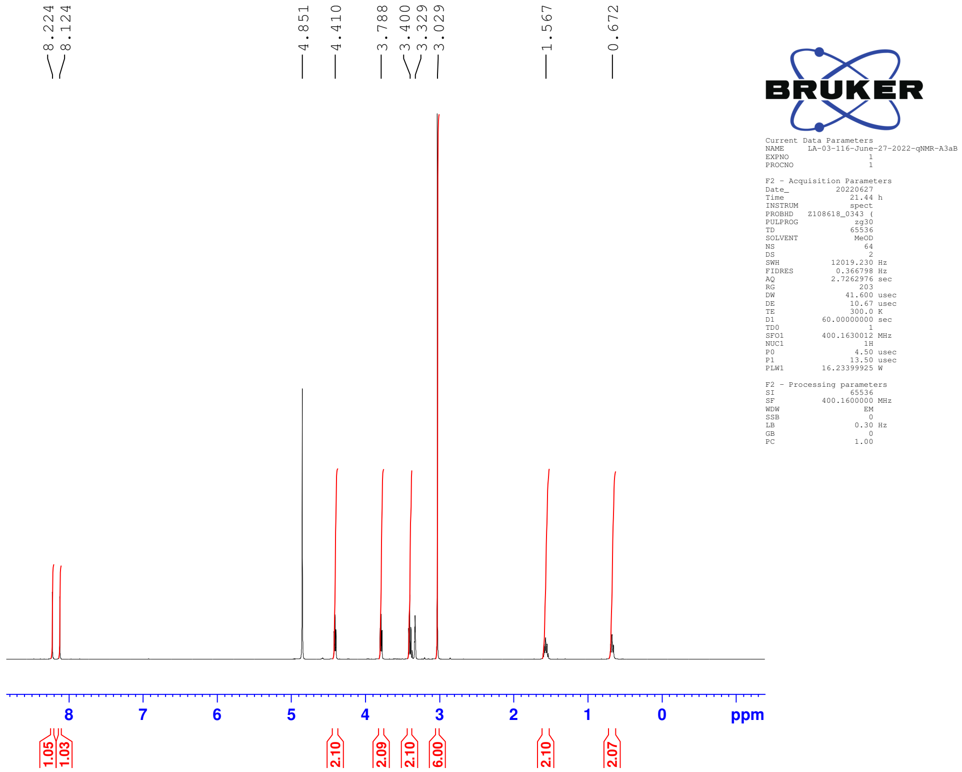

Figure S42 qNMR compound **3aA**, internal standard DMS Purity 99.44%

$$P_{\text{sample}} = \frac{S_{\text{sample}} \text{ x } N_{\text{std}} \text{ x } m_{\text{std}} \text{ x } M_{\text{sample}}}{S_{\text{std}} \text{ x } N_{\text{sample}} \text{ x } m_{\text{sample}} \text{ x } M_{\text{std}}} \text{ x } P_{\text{std}}$$

$$= \frac{2.099 \text{ x } 6 \text{ x } 3.5 \text{ mg x } 265.13 \text{ g mol}^{-1}}{6 \text{ x } 2 \text{ x } 10.4 \text{ mg x } 94.13 \text{ g mol}^{-1}} \text{ x } 99.96$$

$$= 99.44\%$$

S = Integrated area of the peak
N = Number of protons represented
m = Prepared mass
M = Molecular weight
P = Purity

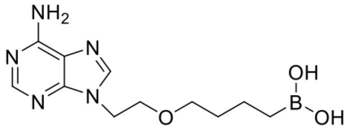

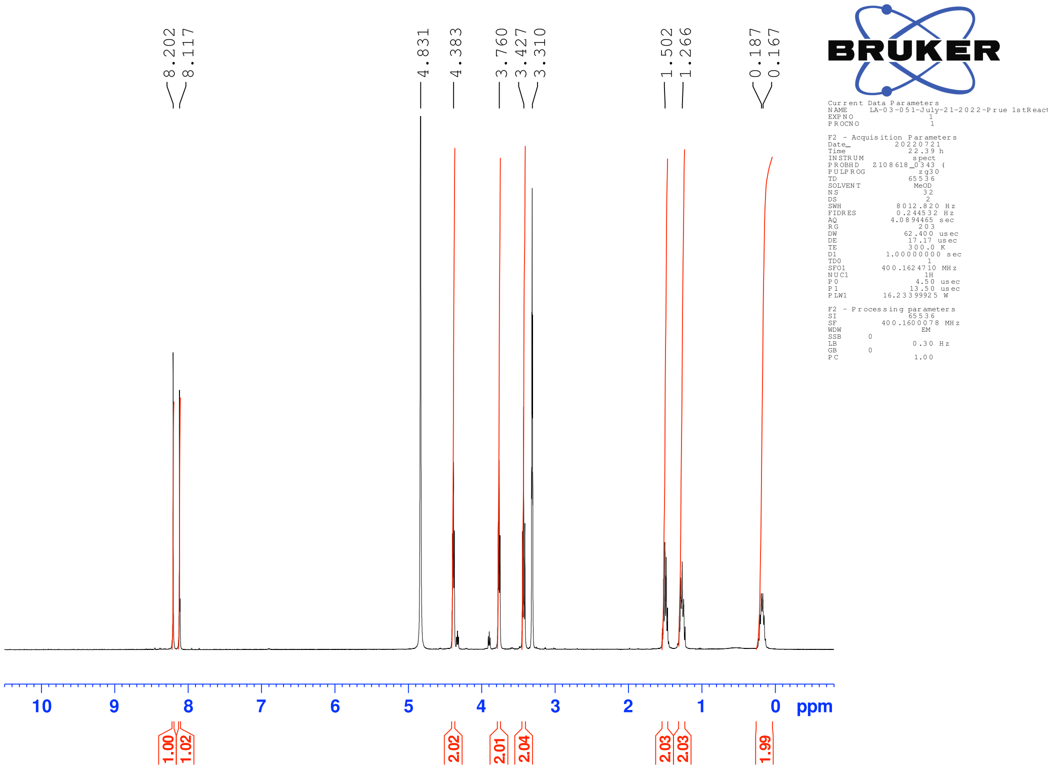

Figure S43 ^{1}HNMR spectra for compound **4aA**

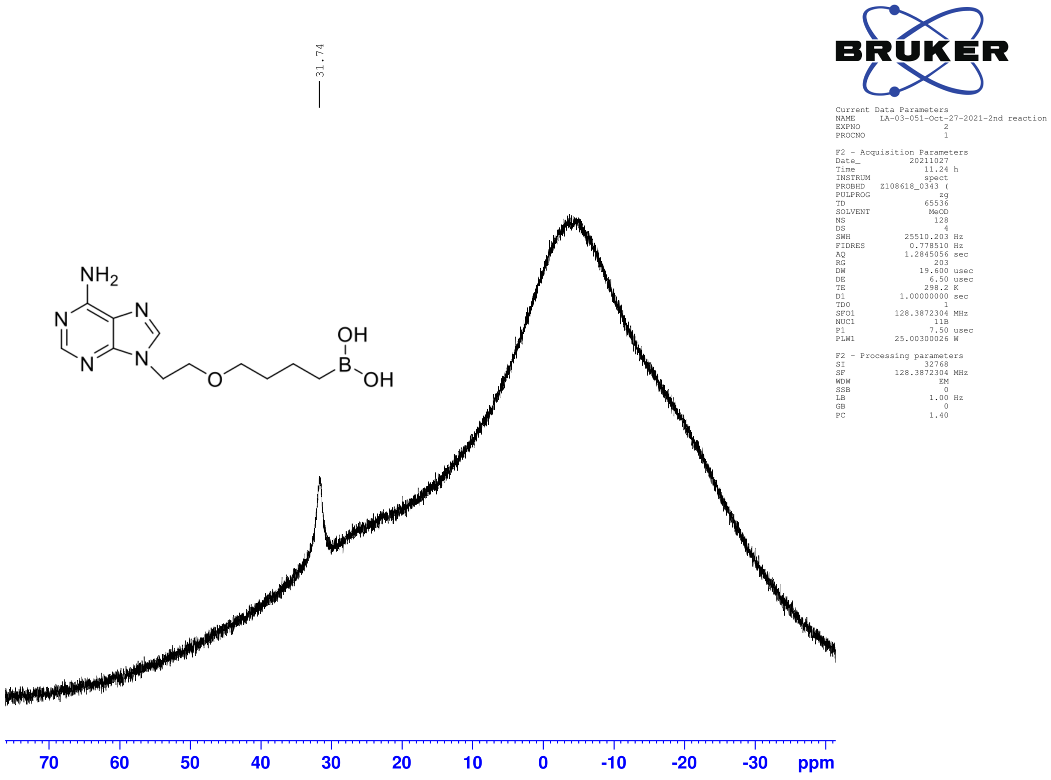

Figure S44 ^{11}BNMR spectra for compound **4aA**

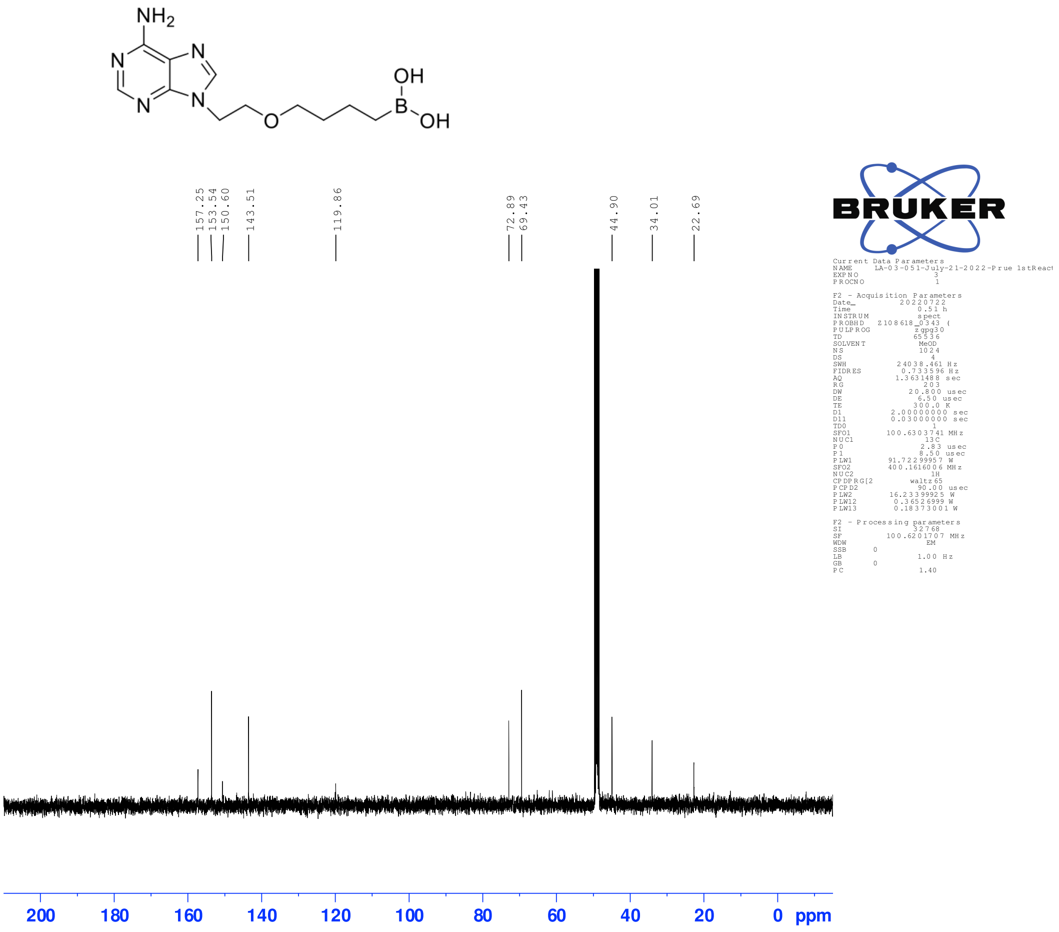

Figure S45 $^{13}C\{^{1}H\}$-NMR spectra for compound **4aA**

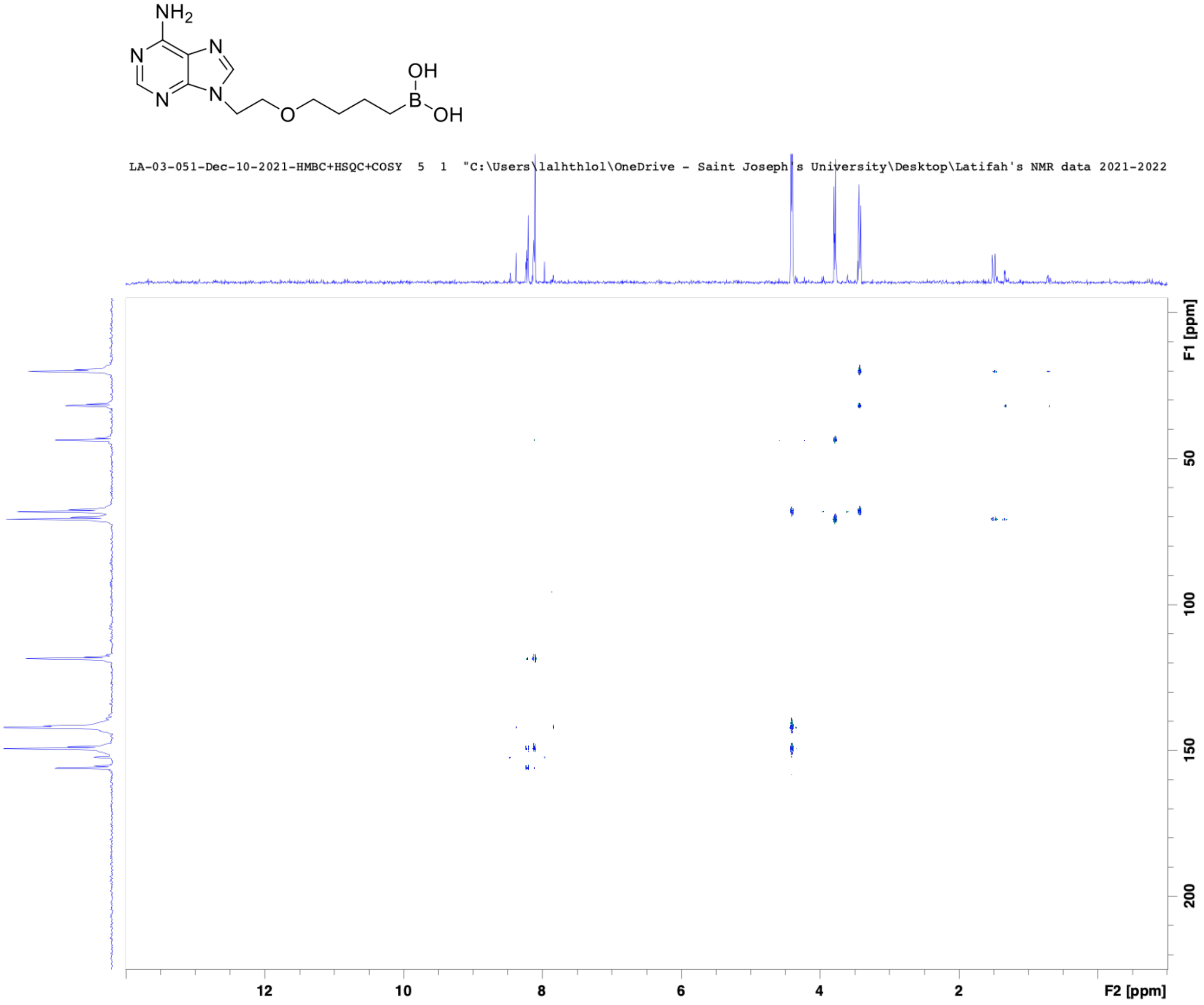

Figure S46 HMBC spectra for compound **4aA**

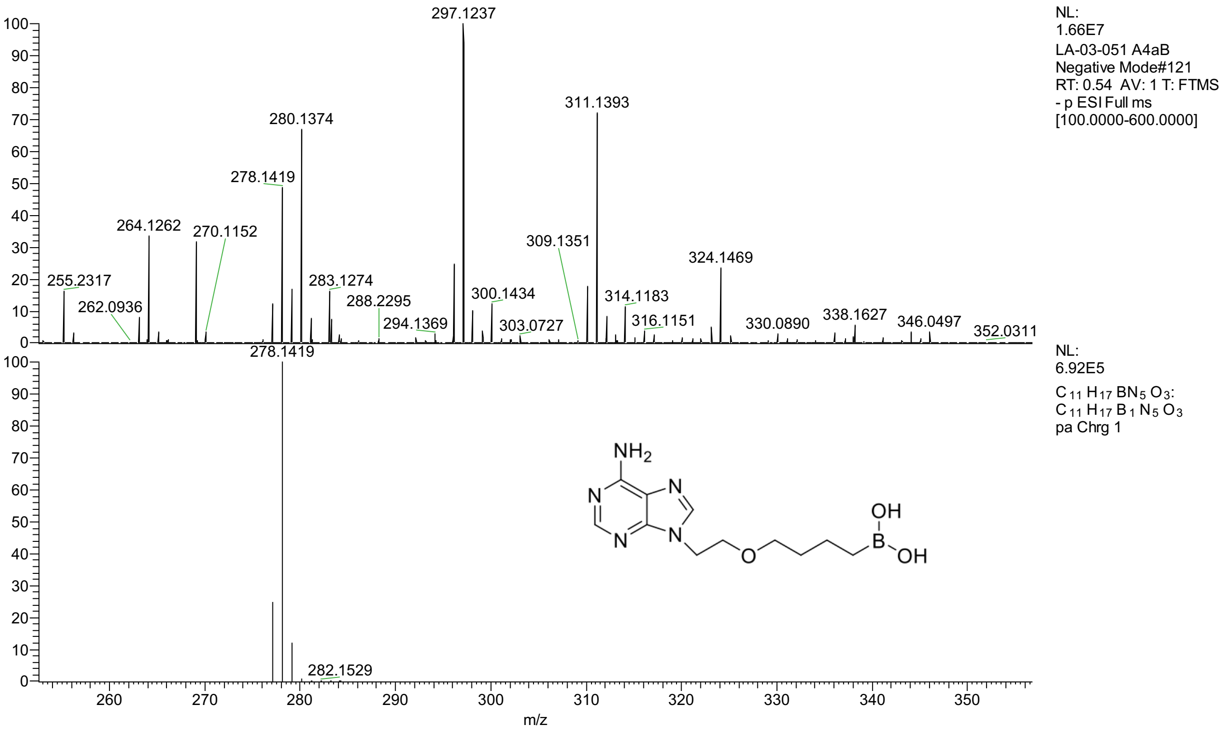

Figure S47 HRMS (ESI) m/z negative mode calculated for compound **4aA** $C_{11}H_{18}BN_5O_3$ - H (M - H)$^-$: 278.1419, found 278.1419.

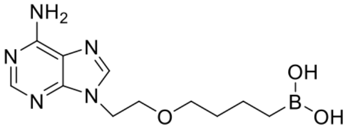

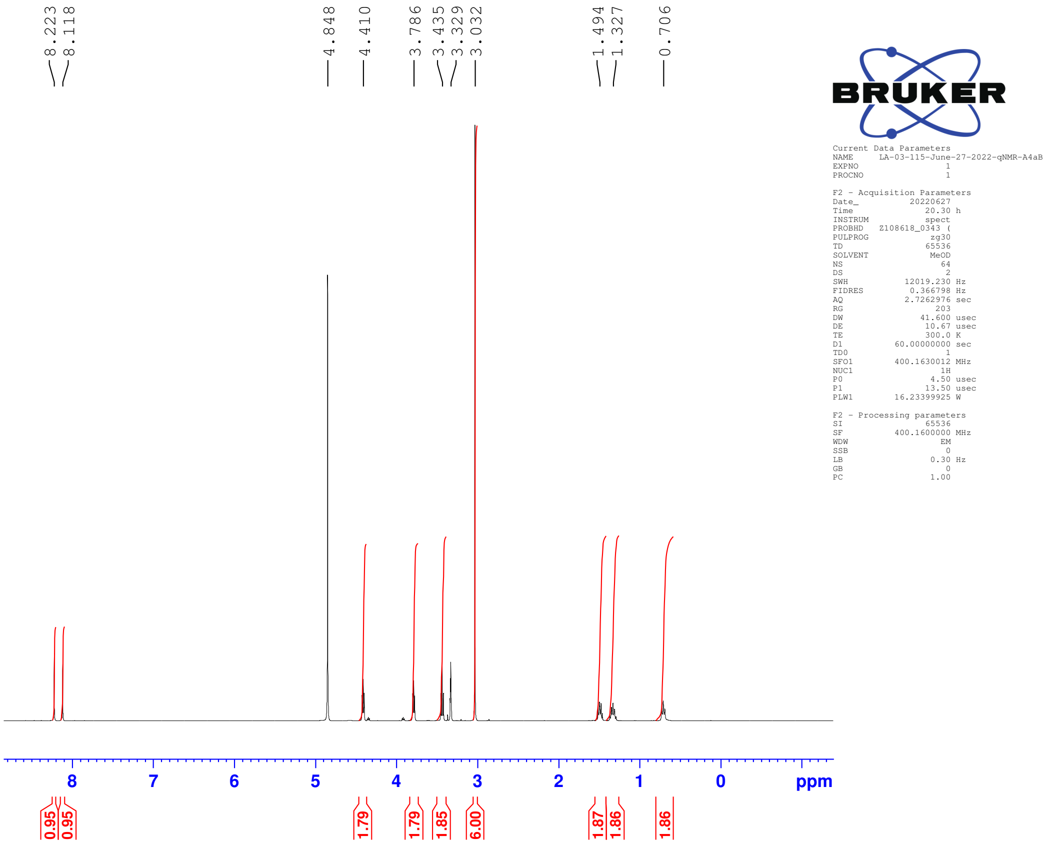

Figure S48 qNMR compound **4aA**, internal standard DMS Purity 95.47%

$$P_{\text{sample}} = \frac{S_{\text{sample}} \text{ x } N_{\text{std}} \text{ x } m_{\text{std}} \text{ x } M_{\text{sample}}}{S_{\text{std}} \text{ x } N_{\text{sample}} \text{ x } m_{\text{sample}} \text{ x } M_{\text{std}}} \text{ x } P_{\text{std}}$$

$$= \frac{1.8646 \text{ x } 6 \text{ x } 3.8 \text{ mg x } 279.15 \text{ g mol}^{-1}}{6 \text{ x } 2 \text{ x } 11.0 \text{ mg x } 94.13 \text{ g mol}^{-1}} \text{ x } 99.96$$

$$= 95.47\%$$

S = Integrated area of the peak
N = Number of protons represented
m = Prepared mass
M = Molecular weight
P = Purity

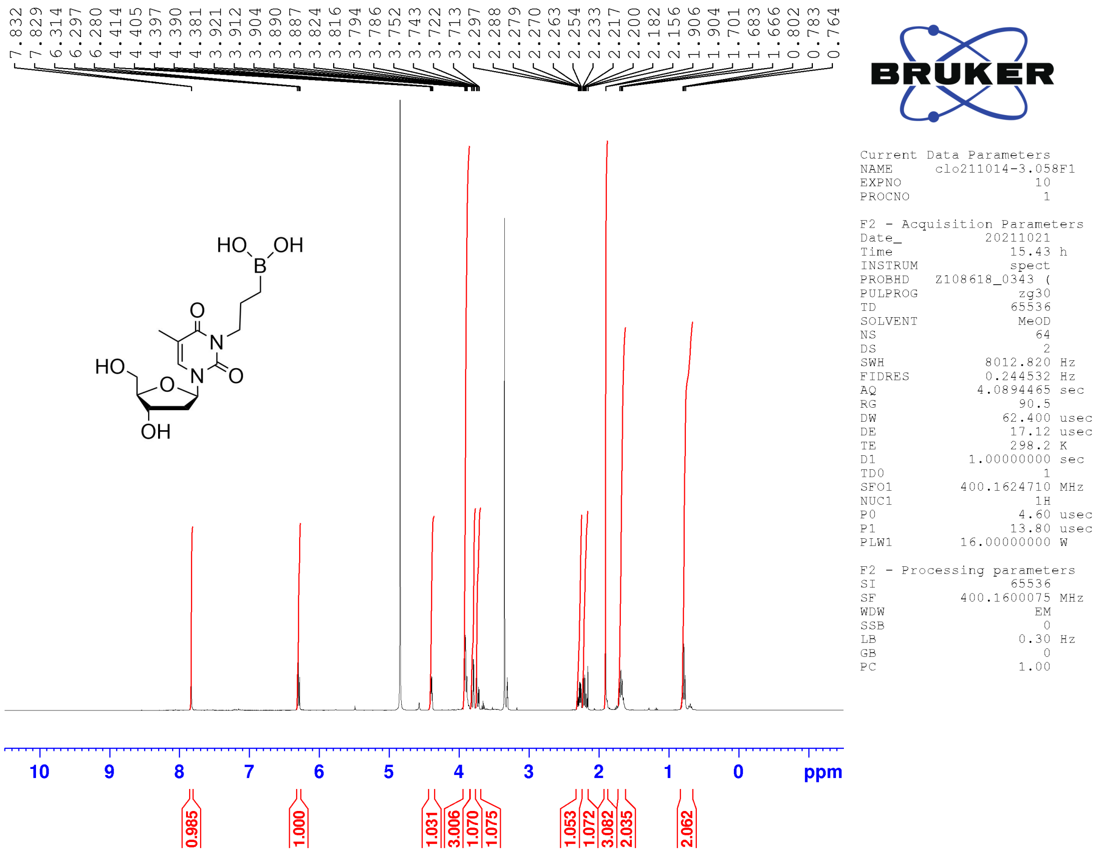

Figure S49 ^{1}HNMR spectra of **N3aT** in CD_3OD.

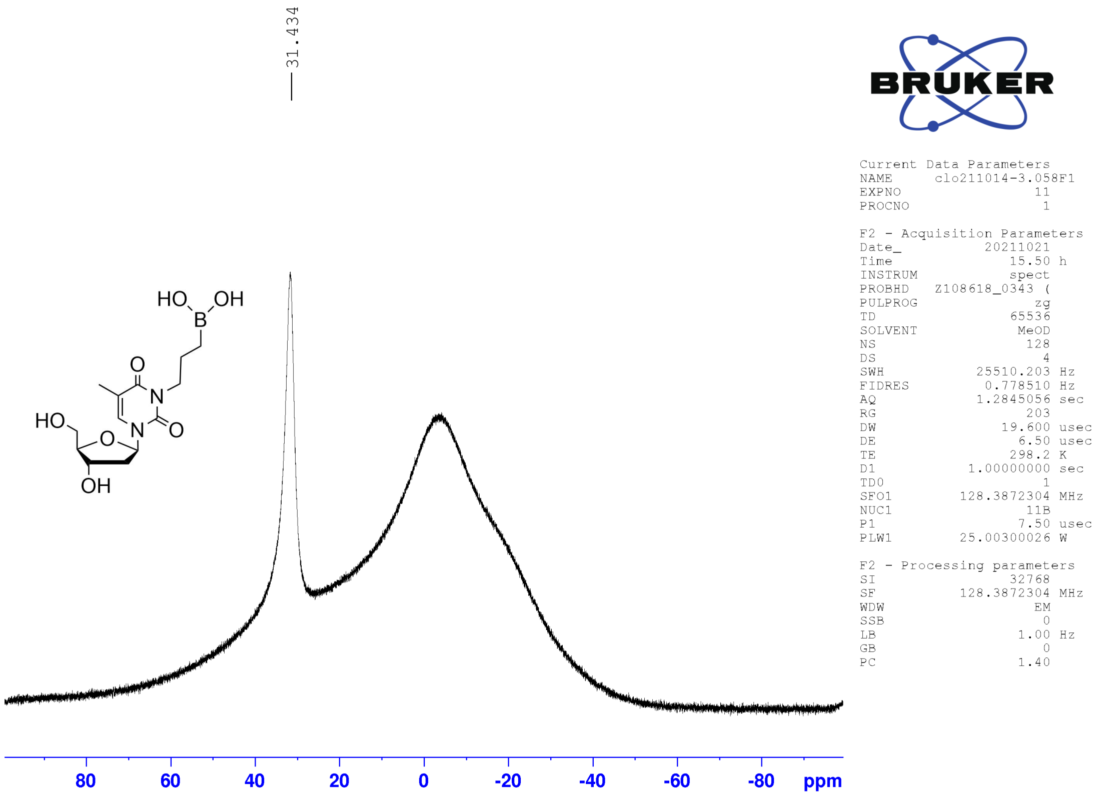

Figure S50 ^{11}BNMR spectra of **N3aT** in CD_3OD.

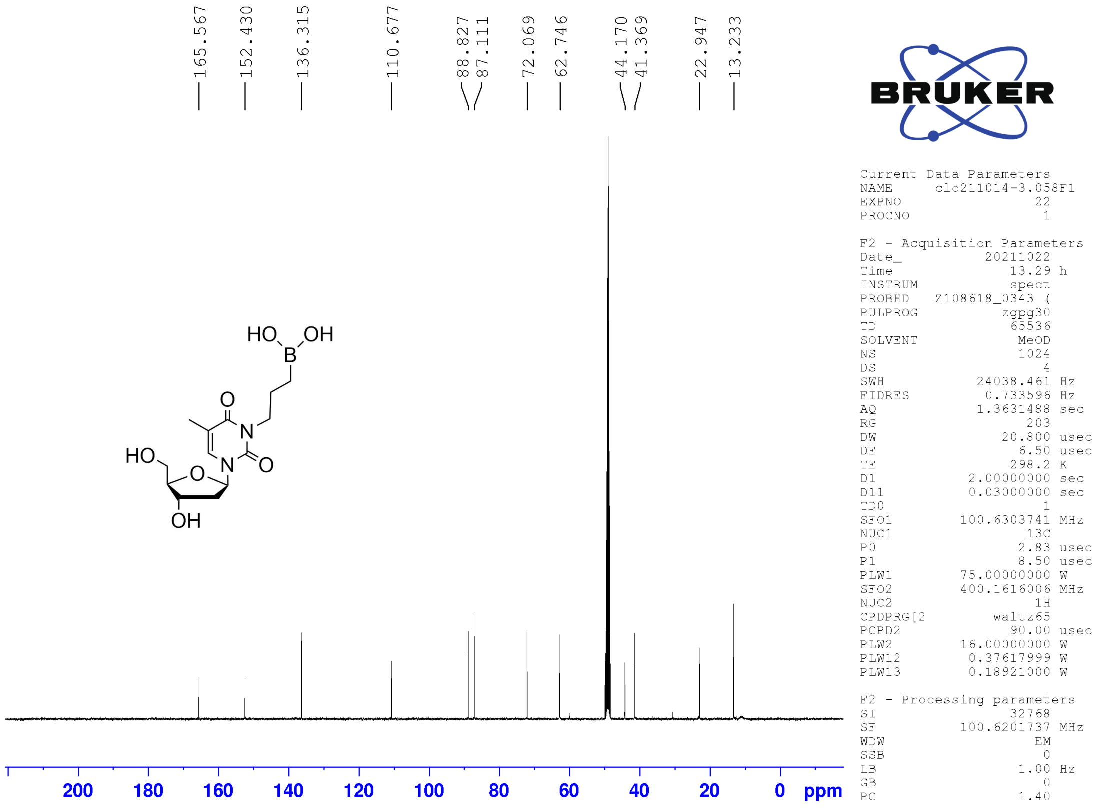

Figure S51 $^{13}C\{^{1}H\}$-NMR spectra of **N3aT** in CD_3OD.

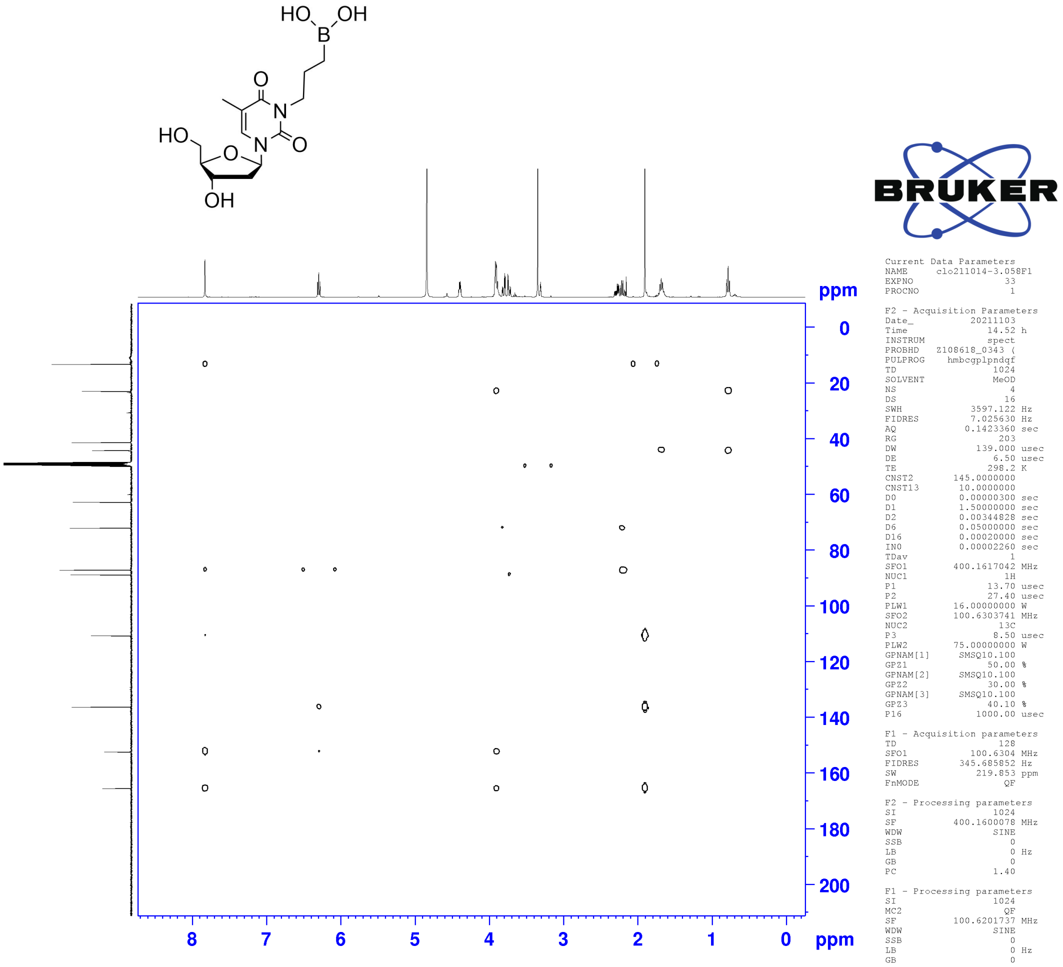

Figure S52 HMBC-NMR spectra of **N3aT** in CD_3OD.

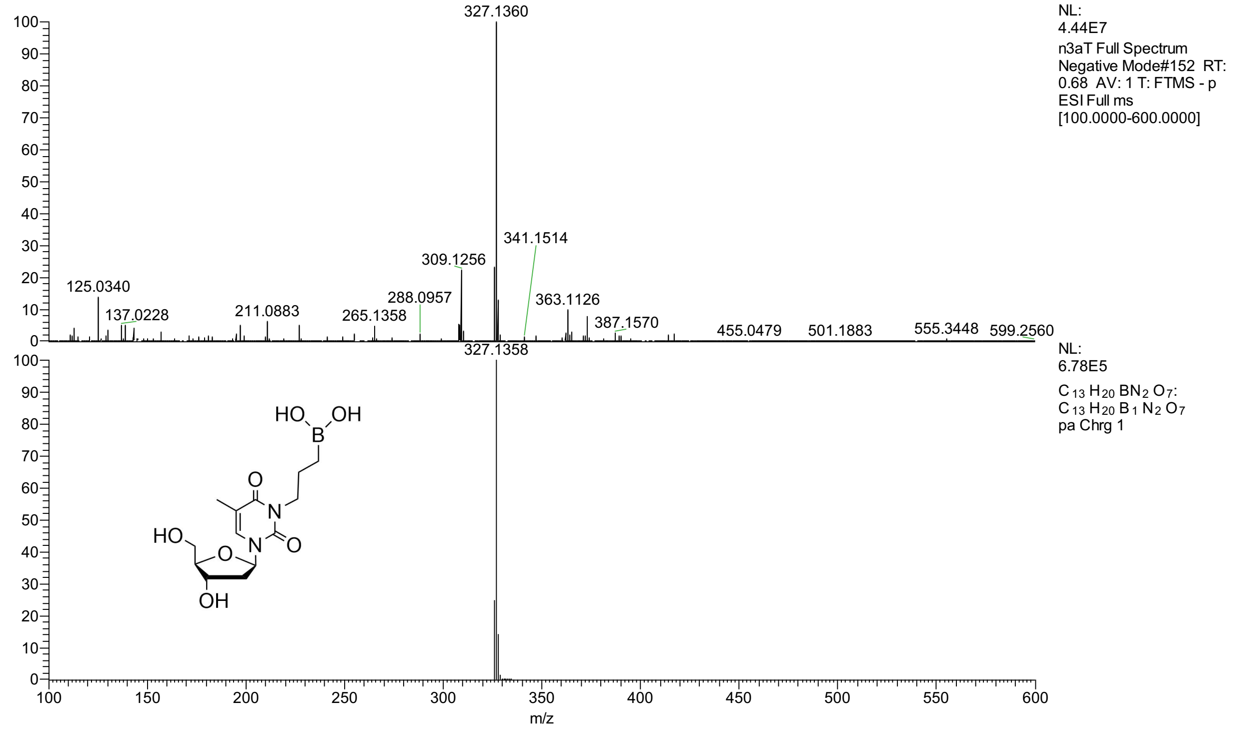

Figure S53 HRMS (ESI-) negative mode m/z calculated for **N3aT** [$C_{13}H_{20}BN_2O_7$] [M-H]$^-$ 327.1358, found 327.1360.

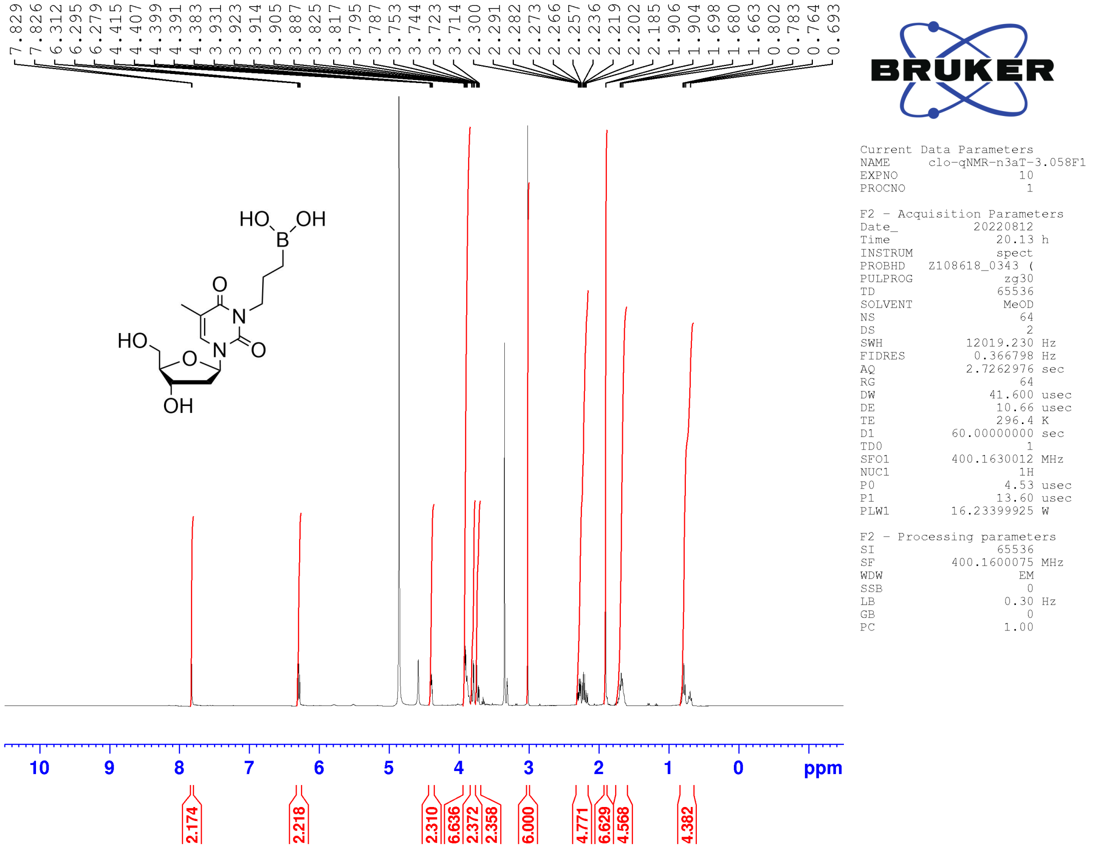

Figure S54 ^{1}H qNMR purity spectrum of **N3aT** with Me_2SO_2 (I.C) in CD_3OD. Purity 97.77%.

$$P_{\text{sample}} = \frac{S_{\text{sample}} \text{ x } N_{\text{std}} \text{ x } m_{\text{std}} \text{ x } M_{\text{sample}}}{S_{\text{std}} \text{ x } N_{\text{sample}} \text{ x } m_{\text{sample}} \text{ x } M_{\text{std}}} \text{ x } P_{\text{std}}$$

$$= \frac{2.218 \text{ x } 6 \text{ x } 4.2 \text{ mg x } 328.13 \text{ g mol}^{-1}}{6 \text{ x } 1 \text{ x } 33.2 \text{ mg x } 94.13 \text{ g mol}^{-1}} \text{ x } 99.96$$

$$= 97.77\%$$

S = Integrated area of the peak
N = Number of protons represented
m = Prepared mass
M = Molecular weight
P = Purity

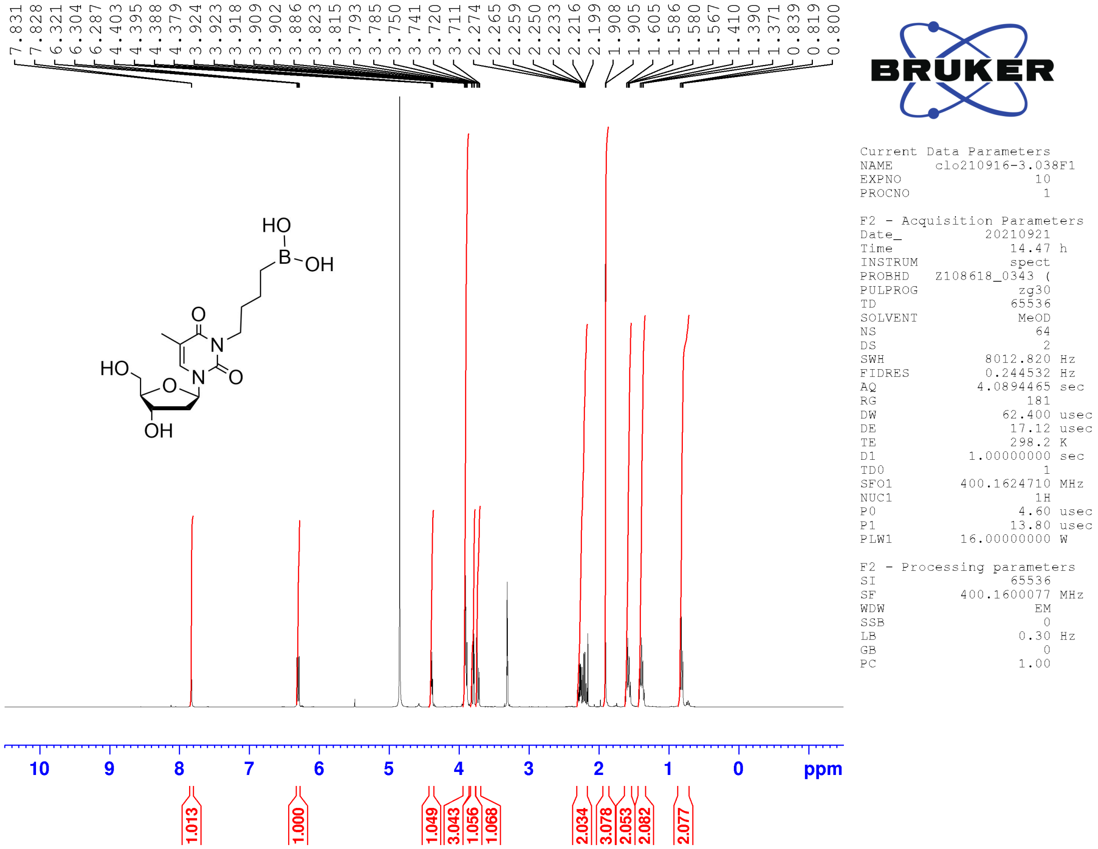

Figure S55 ^{1}HNMR spectra of **N4aT** in CD_3OD.

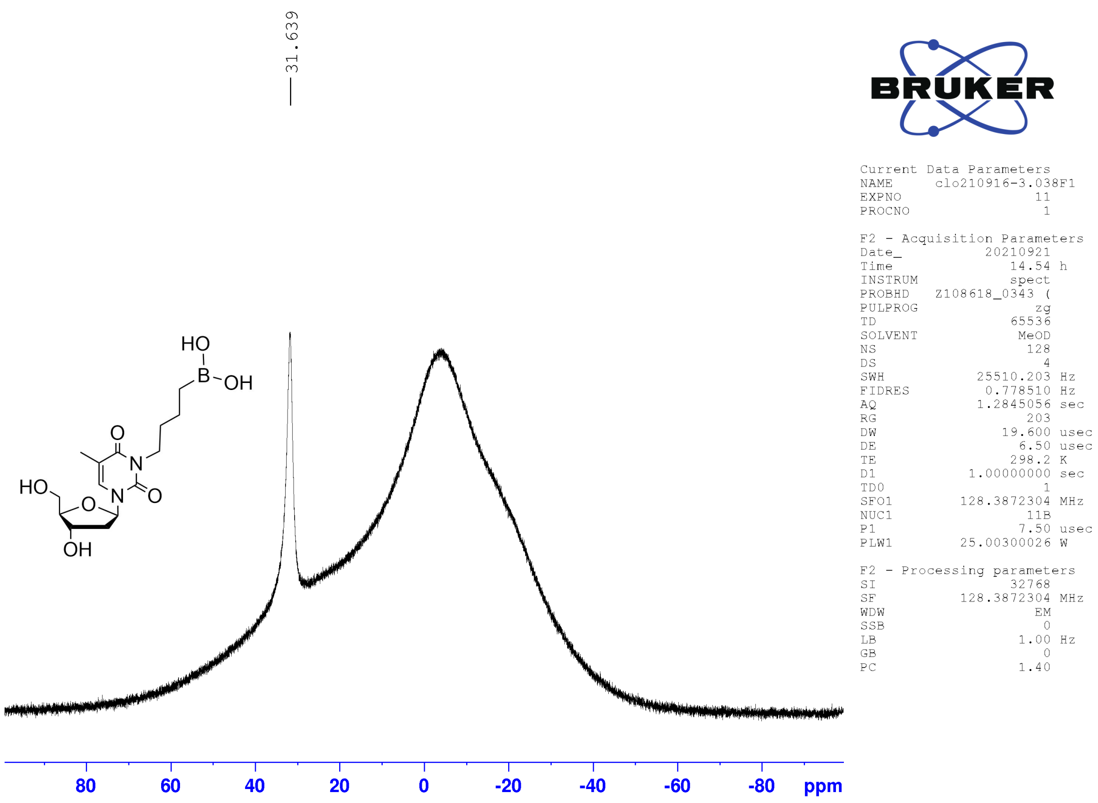

Figure S56 ^{11}BNMR spectra of **N4aT** in CD_3OD.

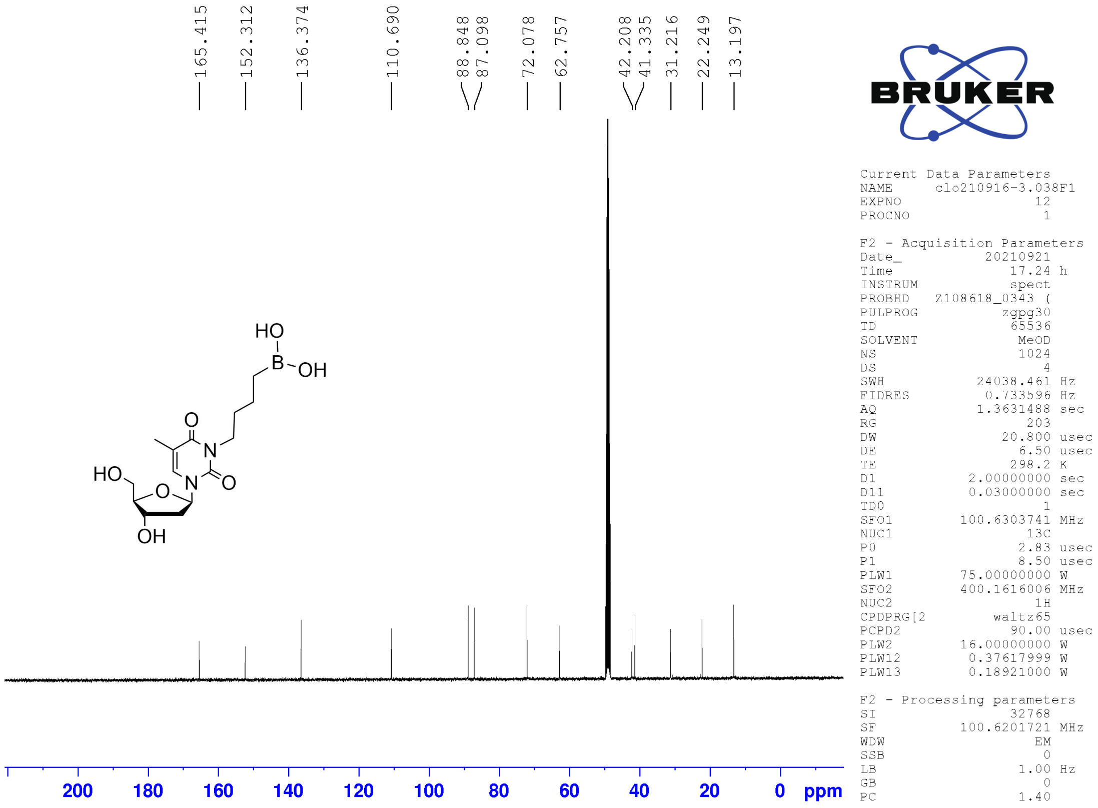

Figure S57 $^{13}C\{^{1}H\}$-NMR spectra of **N4aT** in CD_3OD.

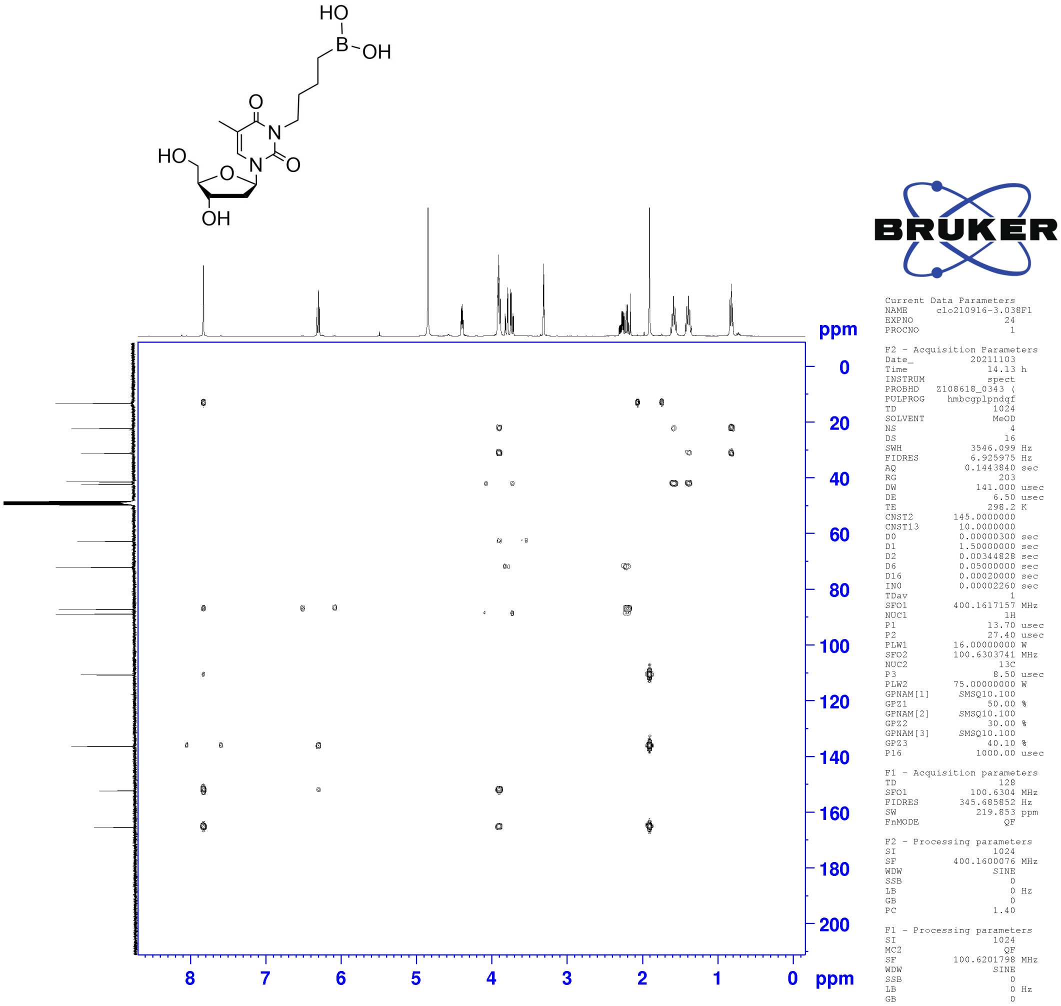

Figure S58 HMBC-NMR spectra of **N4aT** in CD_3OD.

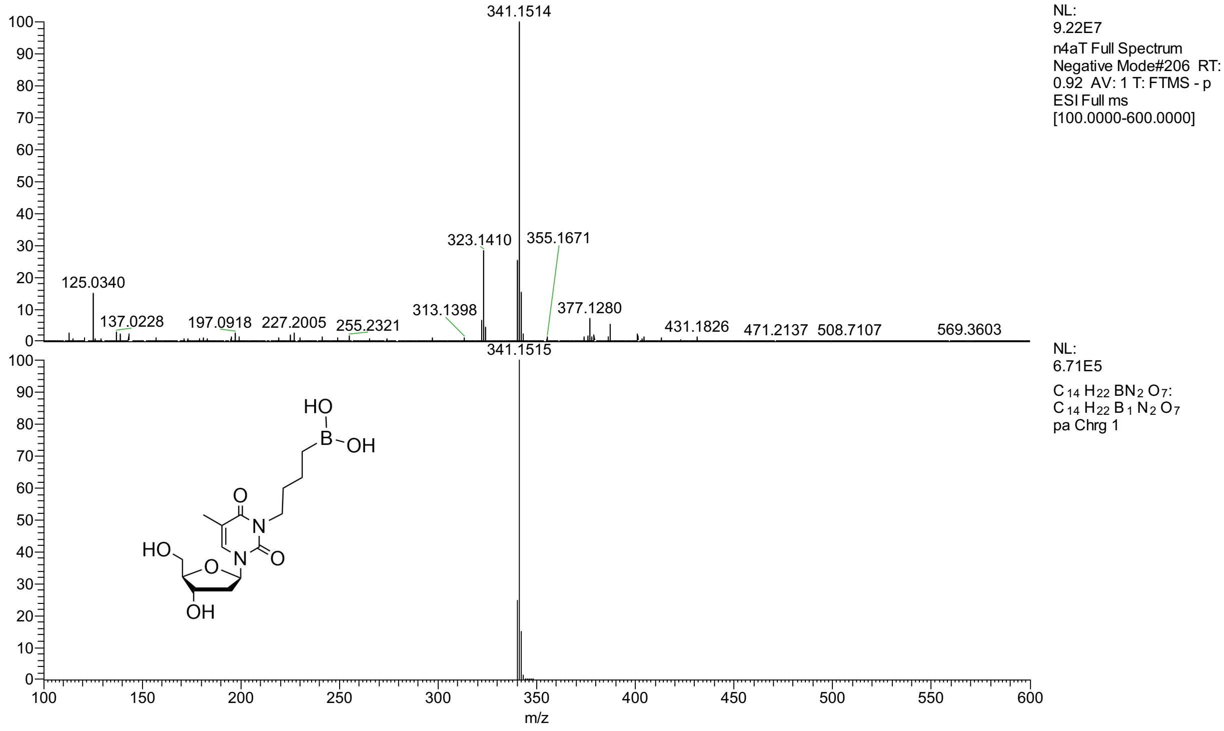

Figure S59 HRMS (ESI-) negative mode m/z calculated for **N4aT** [$C_{14}H_{22}BN_2O_7$] $[M-H]^-$ 341.1515, found 341.1514.

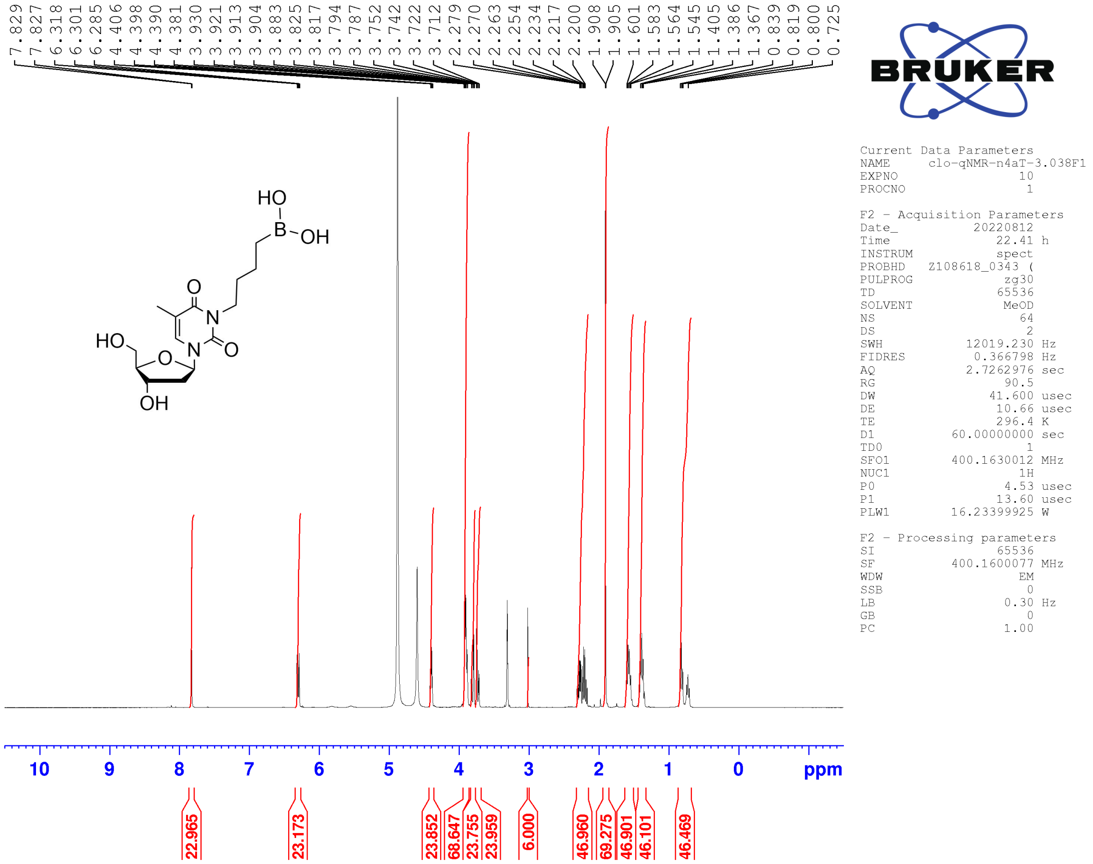

Figure S60 ^{1}H qNMR purity spectrum of **N4aT** with Me_2SO_2 (I.C) in CD_3OD. Purity 96.31%.

$$P_{\text{sample}} = \frac{S_{\text{sample}} \text{ x } N_{\text{std}} \text{ x } m_{\text{std}} \text{ x } M_{\text{sample}}}{S_{\text{std}} \text{ x } N_{\text{sample}} \text{ x } m_{\text{sample}} \text{ x } M_{\text{std}}} \text{ x } P_{\text{std}}$$

$$= \frac{23.173 \text{ x } 6 \text{ x } 0.7 \text{ mg x } 342.16 \text{ g mol}^{-1}}{6 \text{ x } 1 \text{ x } 61.2 \text{ mg x } 94.13 \text{ g mol}^{-1}} \text{ x } 99.96$$

$$= 96.31\%$$

S = Integrated area of the peak
N = Number of protons represented
m = Prepared mass
M = Molecular weight
P = Purity

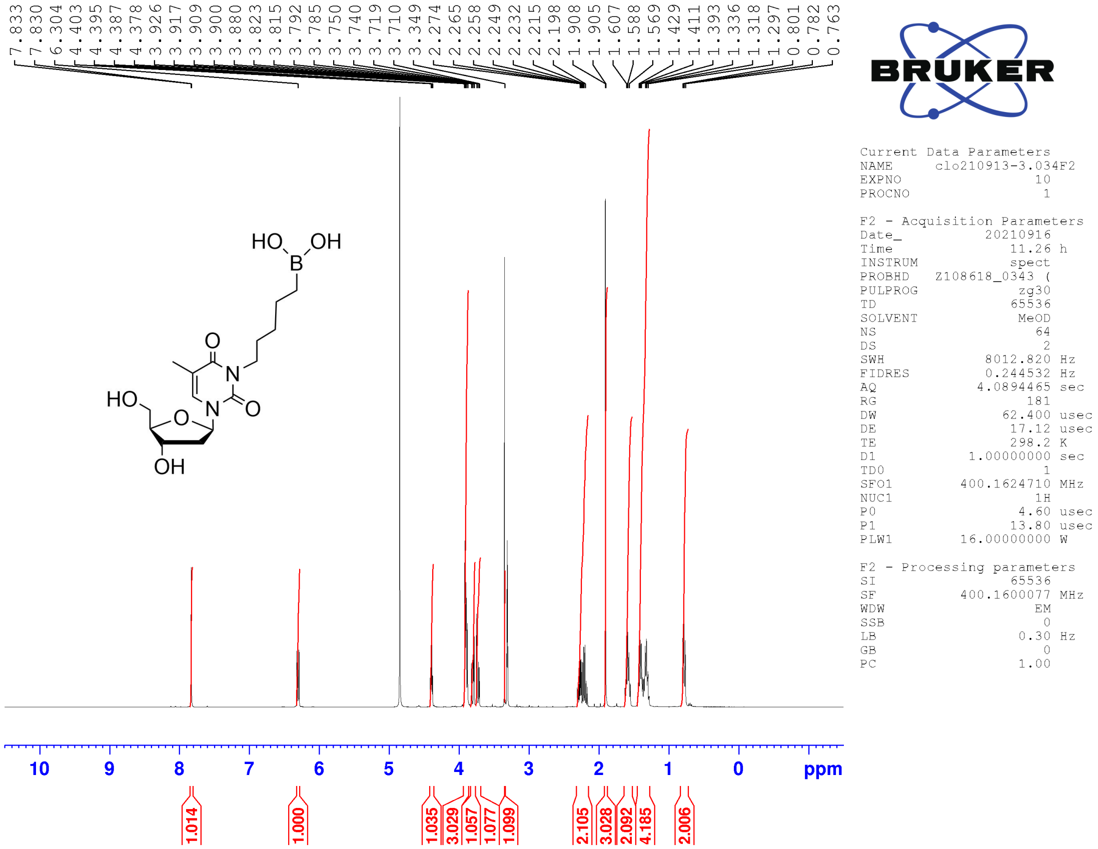

Figure S61 ^{1}HNMR spectra of **N5aT** in CD_3OD.

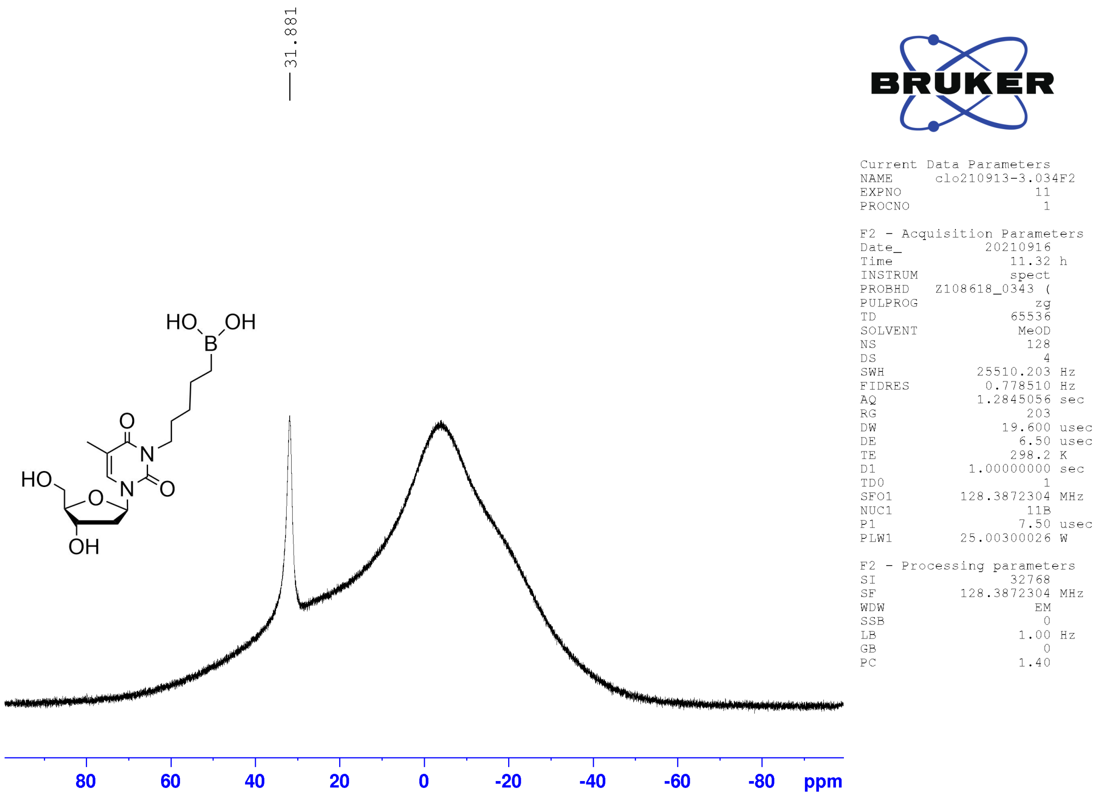

Figure S62 ^{11}BNMR spectra of **N5aT** in CD_3OD.

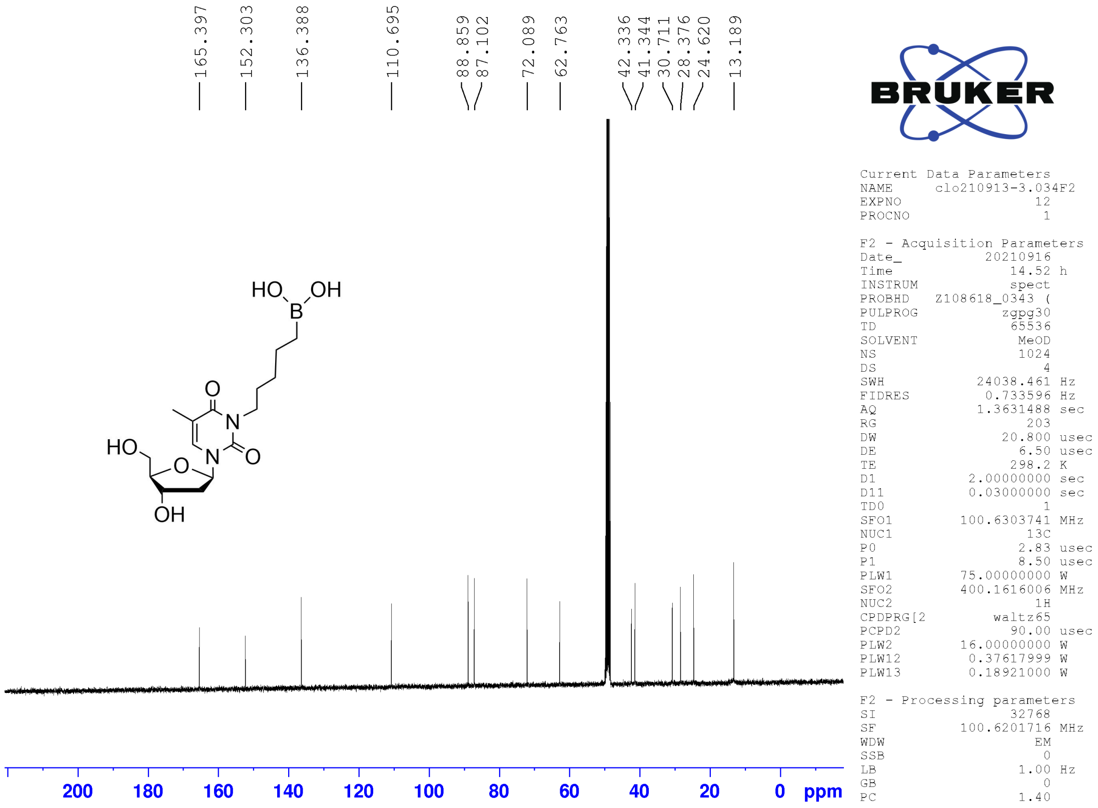

Figure S63 $^{13}C\{^{1}H\}$-NMR spectra of **N5aT** in CD_3OD.

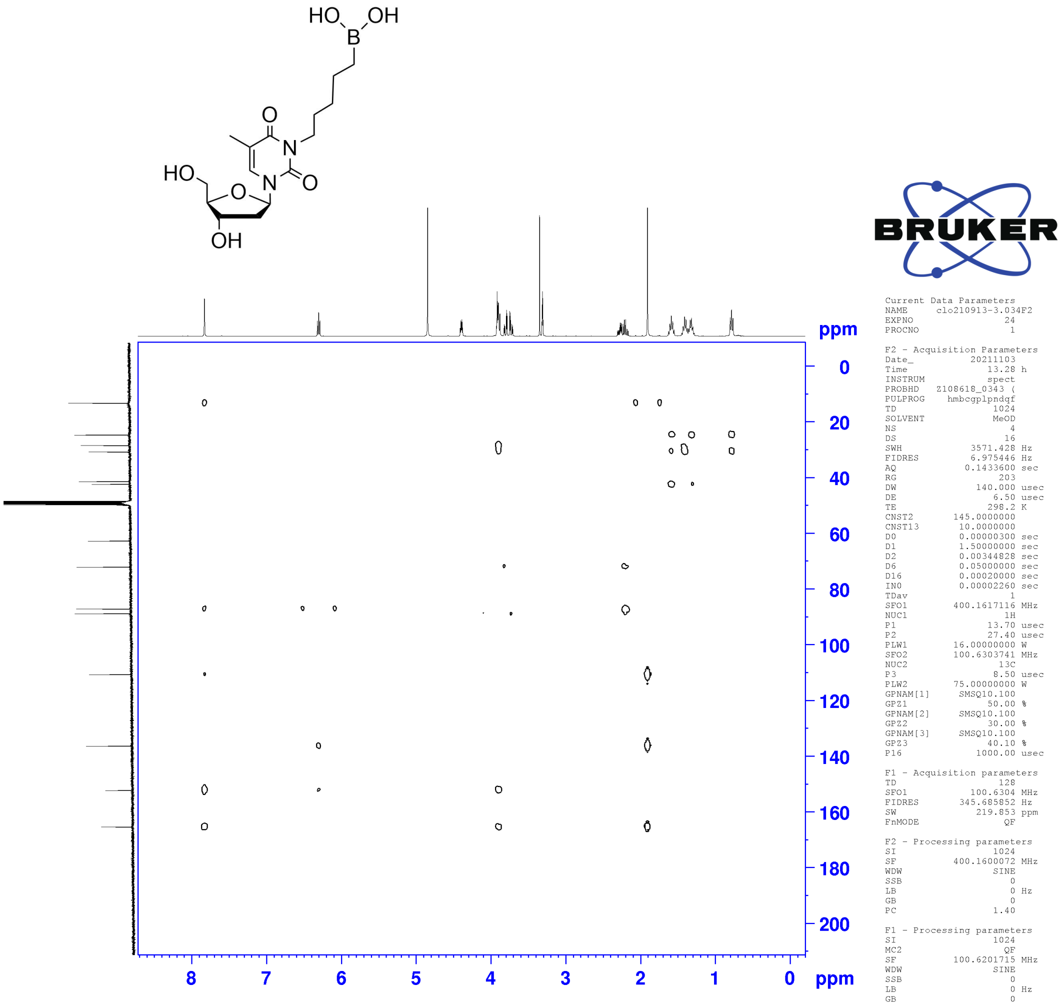

Figure S64 HMBC-NMR spectra of **N5aT** in CD_3OD.

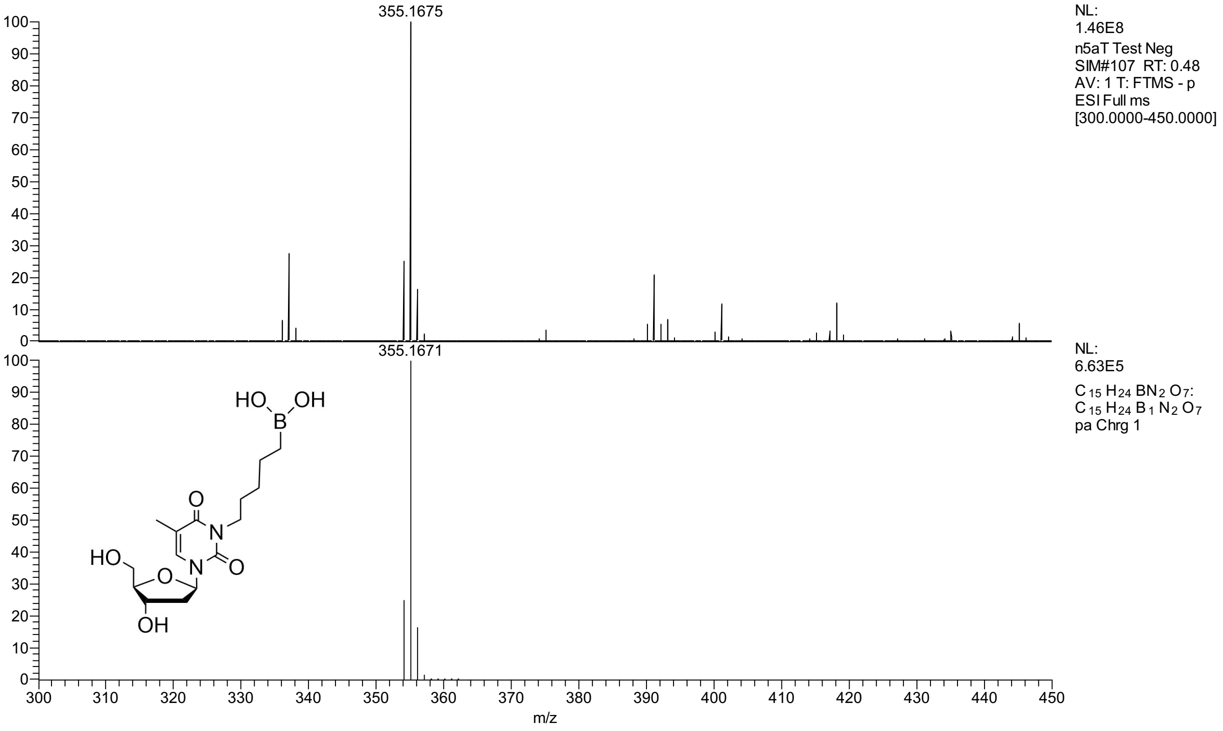

Figure S65 HRMS (ESI-) negative mode m/z calculated for **N5aT** [$C_{15}H_{24}BN_2O_7$] $[M-H]^-$ 355.1671, found 355.1675.

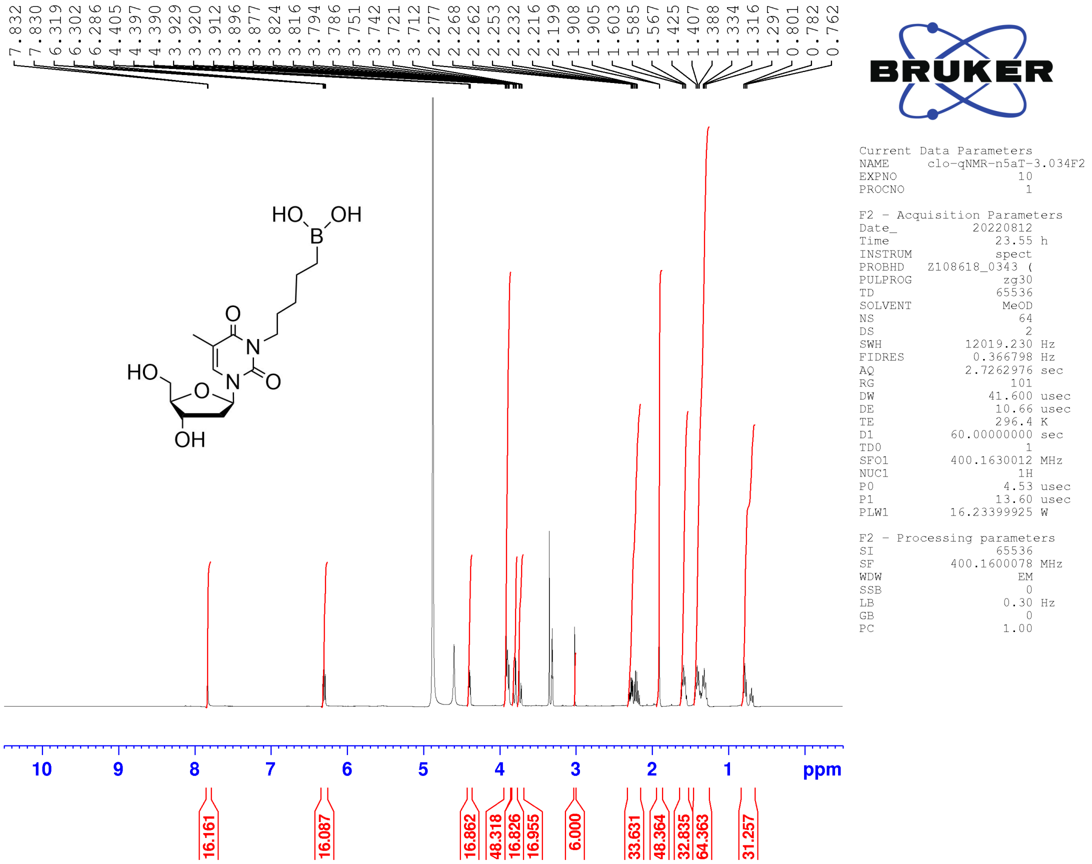

Figure S66 ^{1}H qNMR purity spectrum of **N5aT** with Me_2SO_2 (I.C) in CD_3OD. Purity 96.44%.

$$P_{\text{sample}} = \frac{S_{\text{sample}} \text{ x } N_{\text{std}} \text{ x } m_{\text{std}} \text{ x } M_{\text{sample}}}{S_{\text{std}} \text{ x } N_{\text{sample}} \text{ x } m_{\text{sample}} \text{ x } M_{\text{std}}} \text{ x } P_{\text{std}}$$

$$= \frac{16.087 \text{ x } 6 \text{ x } 1.1 \text{ mg x } 356.18 \text{ g mol}^{-1}}{6 \text{ x } 1 \text{ x } 69.4 \text{ mg x } 94.13 \text{ g mol}^{-1}} \text{ x } 99.96$$

$$= 96.44\%$$

S = Integrated area of the peak
N = Number of protons represented
m = Prepared mass
M = Molecular weight
P = Purity

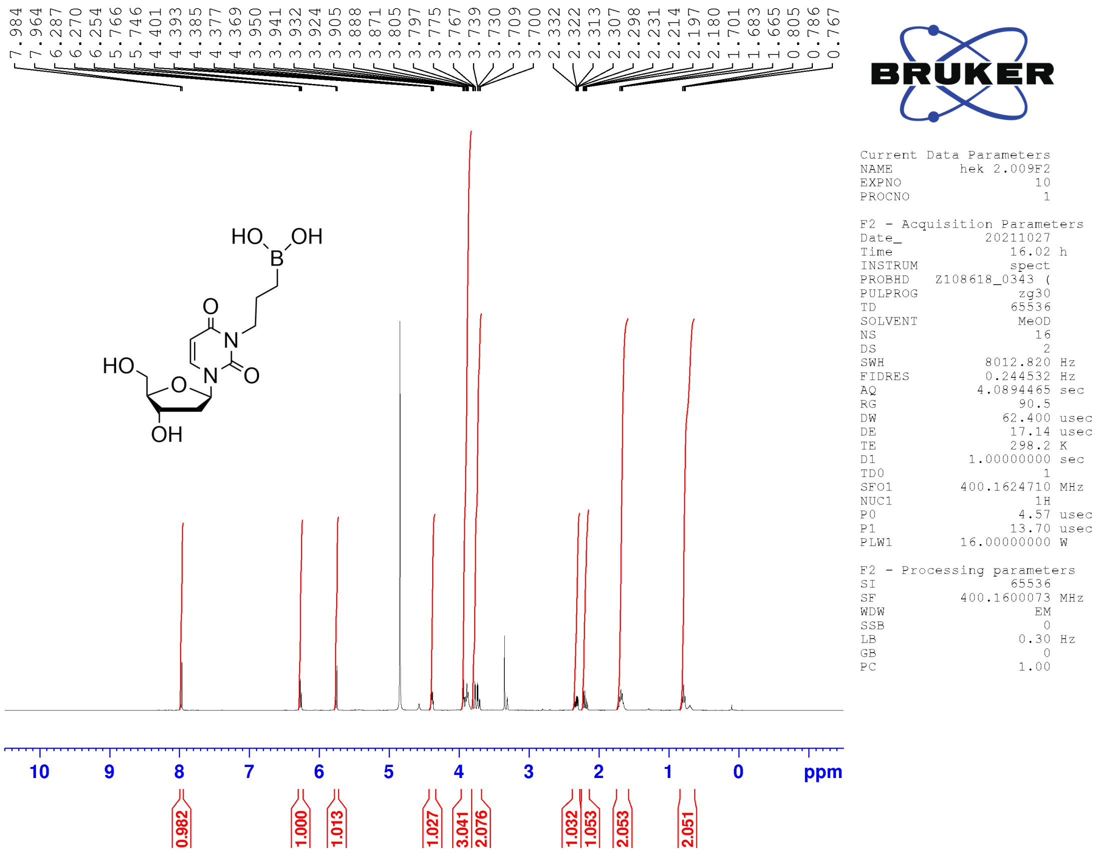

Figure S67 ^{1}HNMR spectra of **N3aU** in CD_3OD.

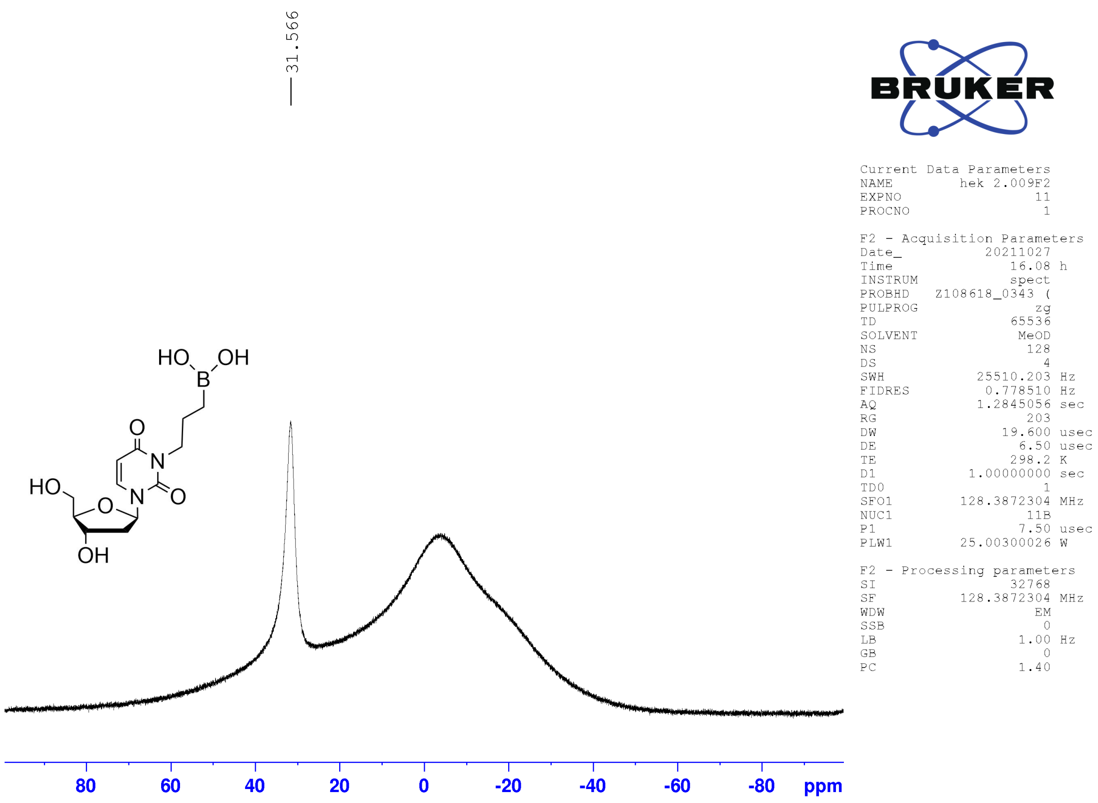

Figure S68 ^{11}BNMR spectra of **N3aU** in CD_3OD.

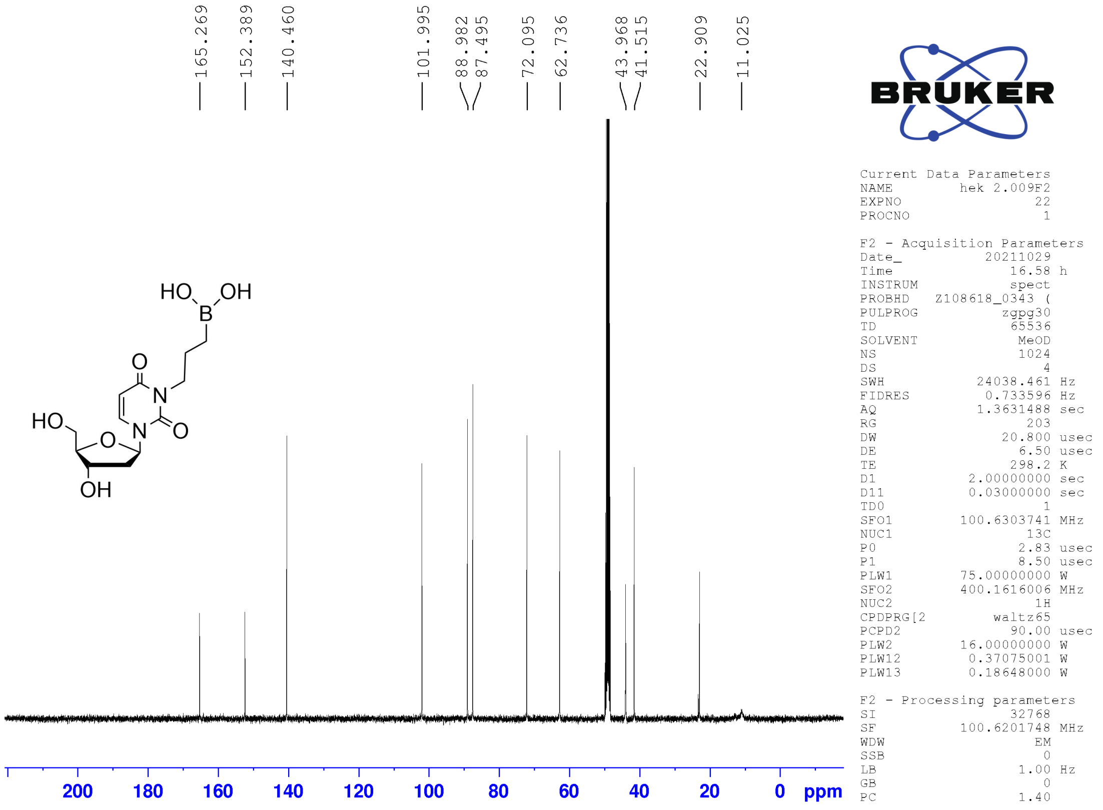

Figure S69 $^{13}C\{^{1}H\}$-NMR spectra of **N3aU** in CD_3OD.

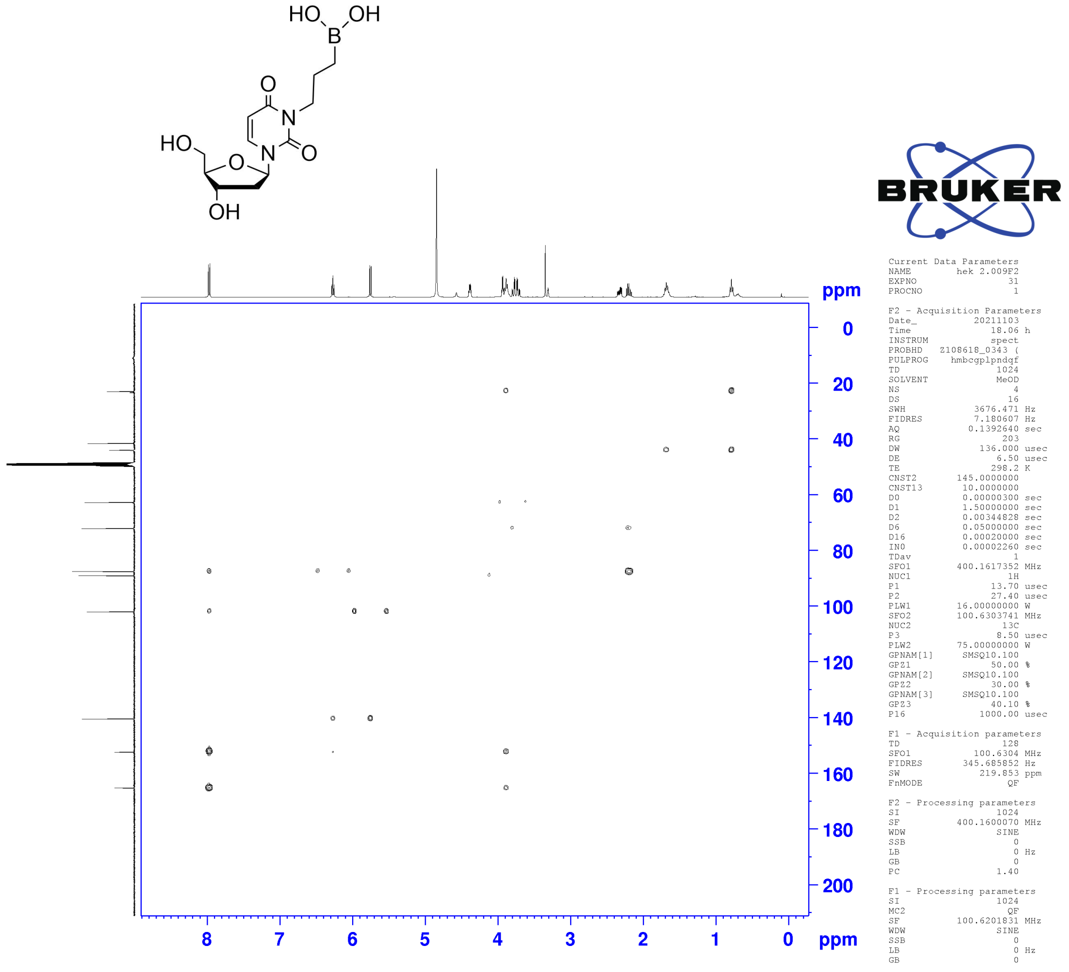

Figure S70 HMBC-NMR spectra of **N3aU** in CD_3OD.

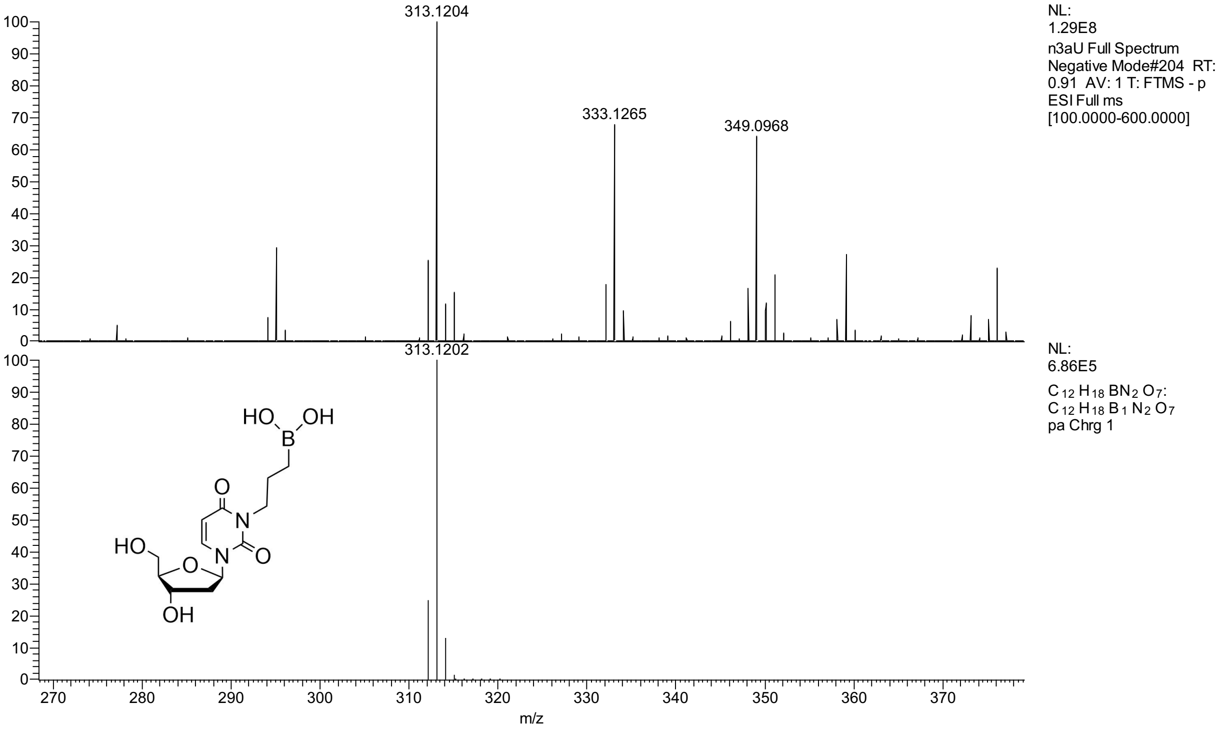

Figure S71 HRMS (ESI-) negative mode m/z calculated for **N3aU** [$C_{12}H_{18}BN_2O_7$] $[M-H]^-$ 313.1202, found 313.1204.

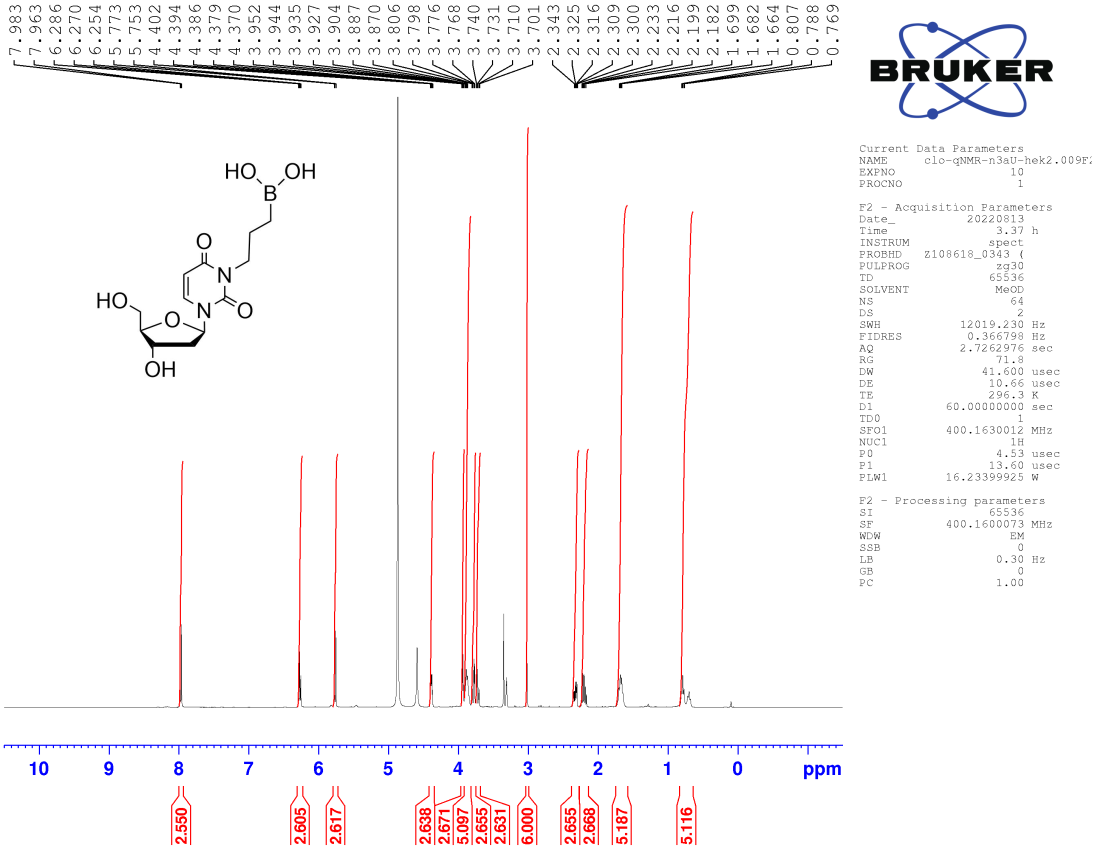

Figure S72 ^{1}H qNMR purity spectrum of **N3aU** with Me_2SO_2 (I.C) in CD_3OD. Purity 97.28%.

$$P_{\text{sample}} = \frac{S_{\text{sample}} \text{ x } N_{\text{std}} \text{ x } m_{\text{std}} \text{ x } M_{\text{sample}}}{S_{\text{std}} \text{ x } N_{\text{sample}} \text{ x } m_{\text{sample}} \text{ x } M_{\text{std}}} \text{ x } P_{\text{std}}$$

$$= \frac{2.550 \text{ x } 6 \text{ x } 3.9 \text{ mg x } 314.10 \text{ g mol}^{-1}}{6 \text{ x } 1 \text{ x } 34.1 \text{ mg x } 94.13 \text{ g mol}^{-1}} \text{ x } 99.96$$

$$= 97.28\%$$

S = Integrated area of the peak
N = Number of protons represented
m = Prepared mass
M = Molecular weight
P = Purity

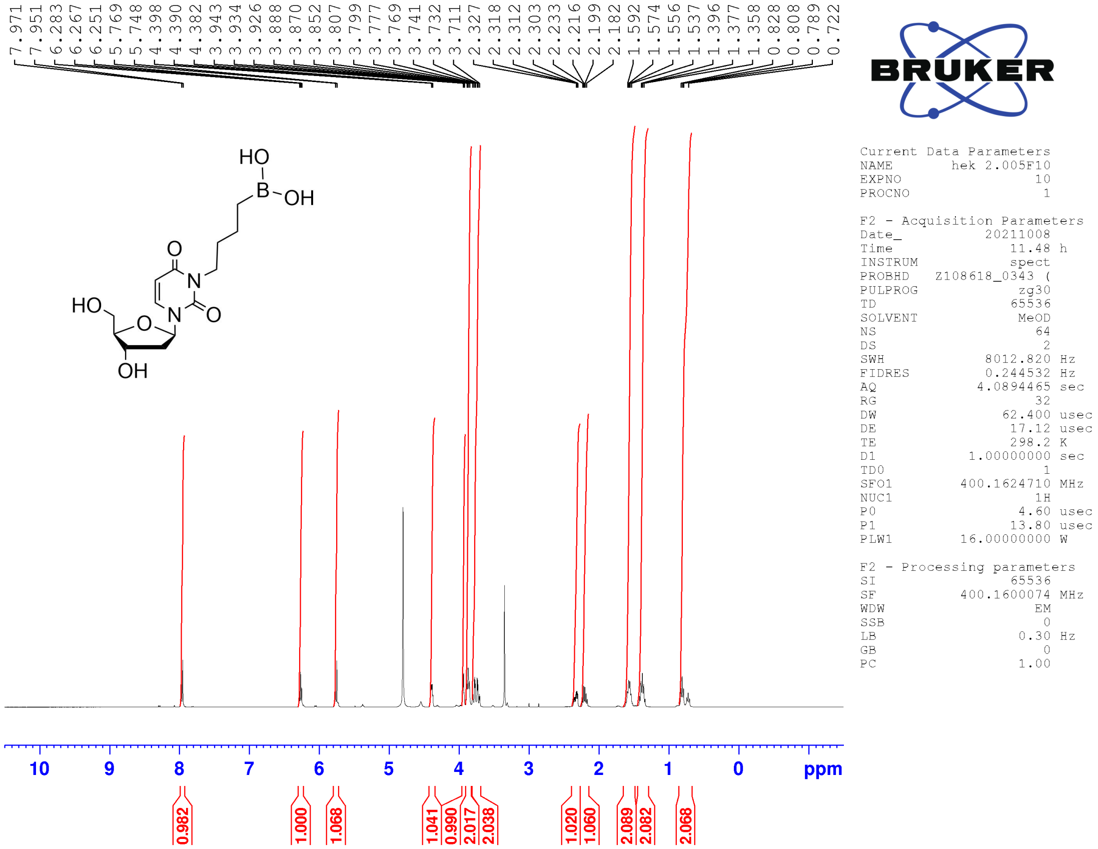

Figure S73 ^{1}HNMR spectra of **N4aU** in CD_3OD.

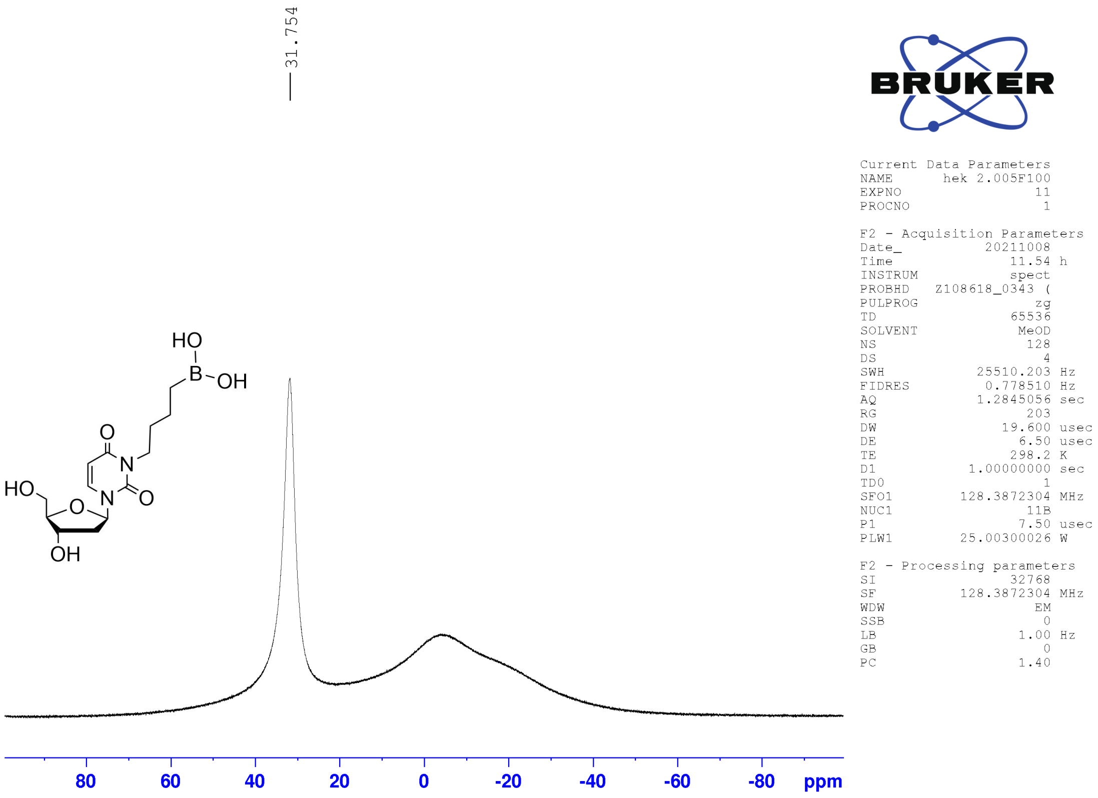

Figure S74 ^{11}BNMR spectra of **N4aU** in CD_3OD.

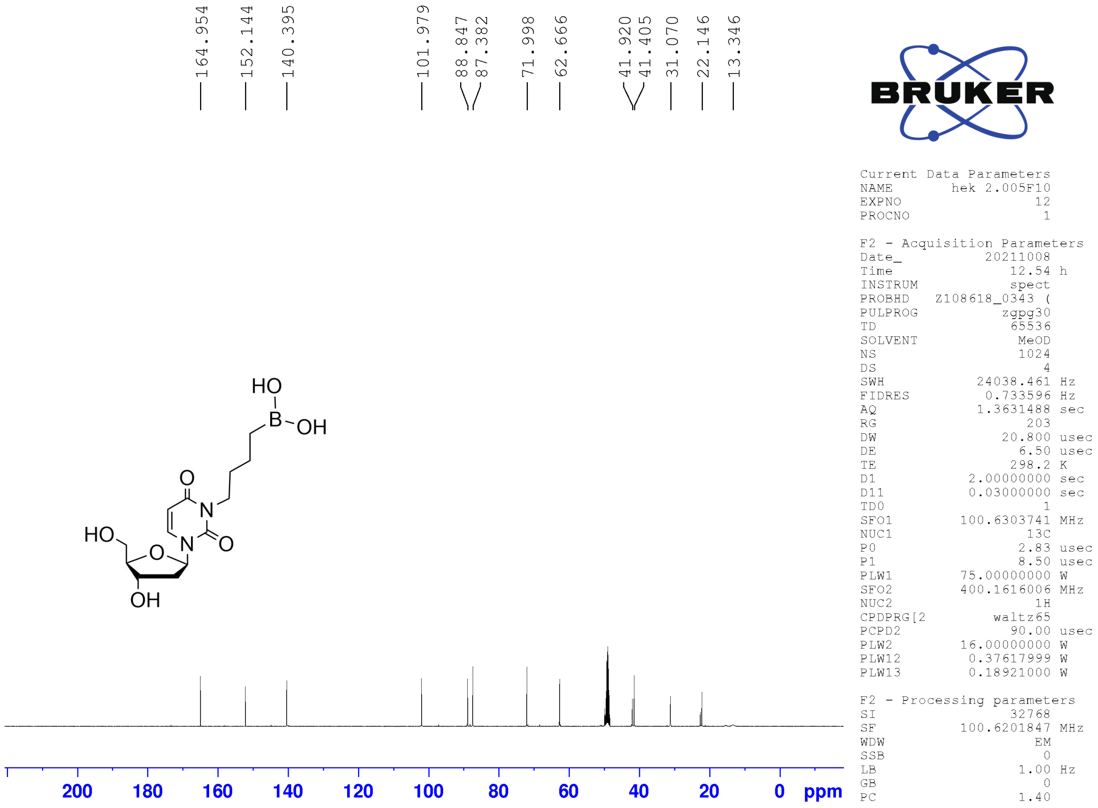

Figure S75 $^{13}C\{^{1}H\}$-NMR spectra of **N4aU** in CD_3OD.

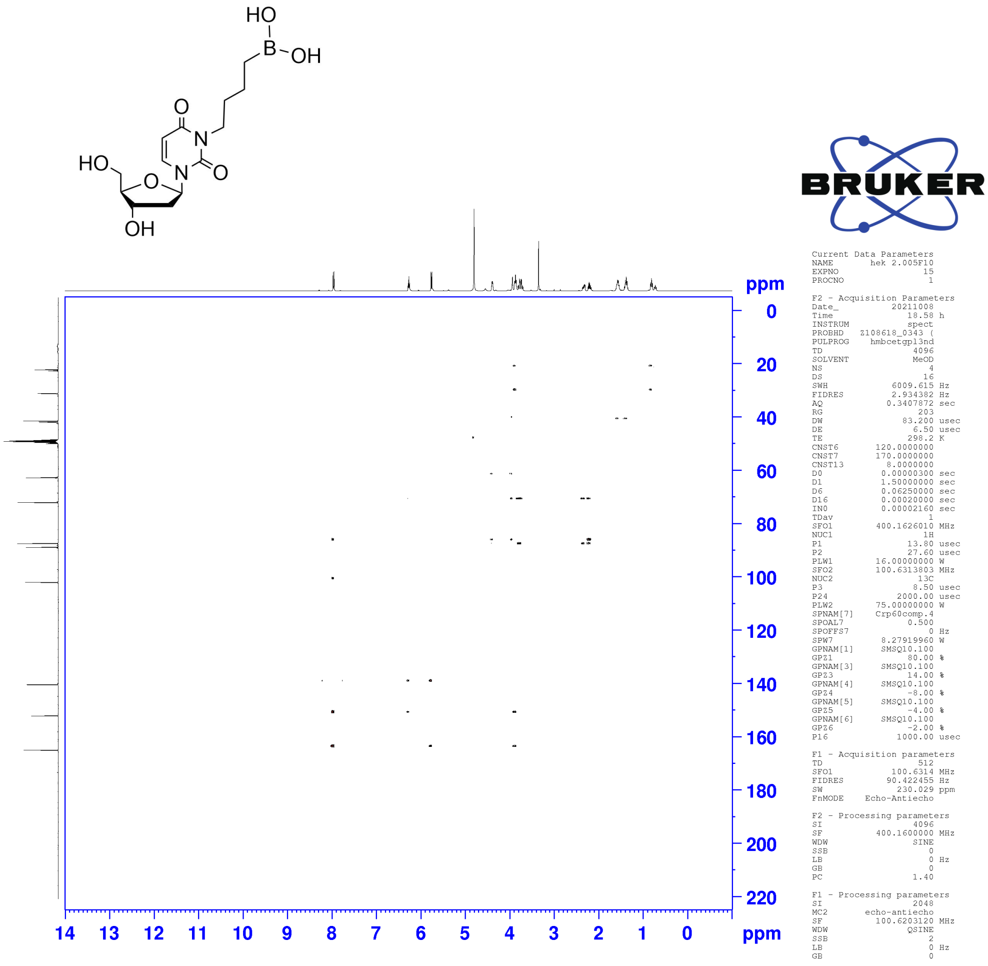

Figure S76 HMBC-NMR spectra of **N4aU** in CD_3OD.

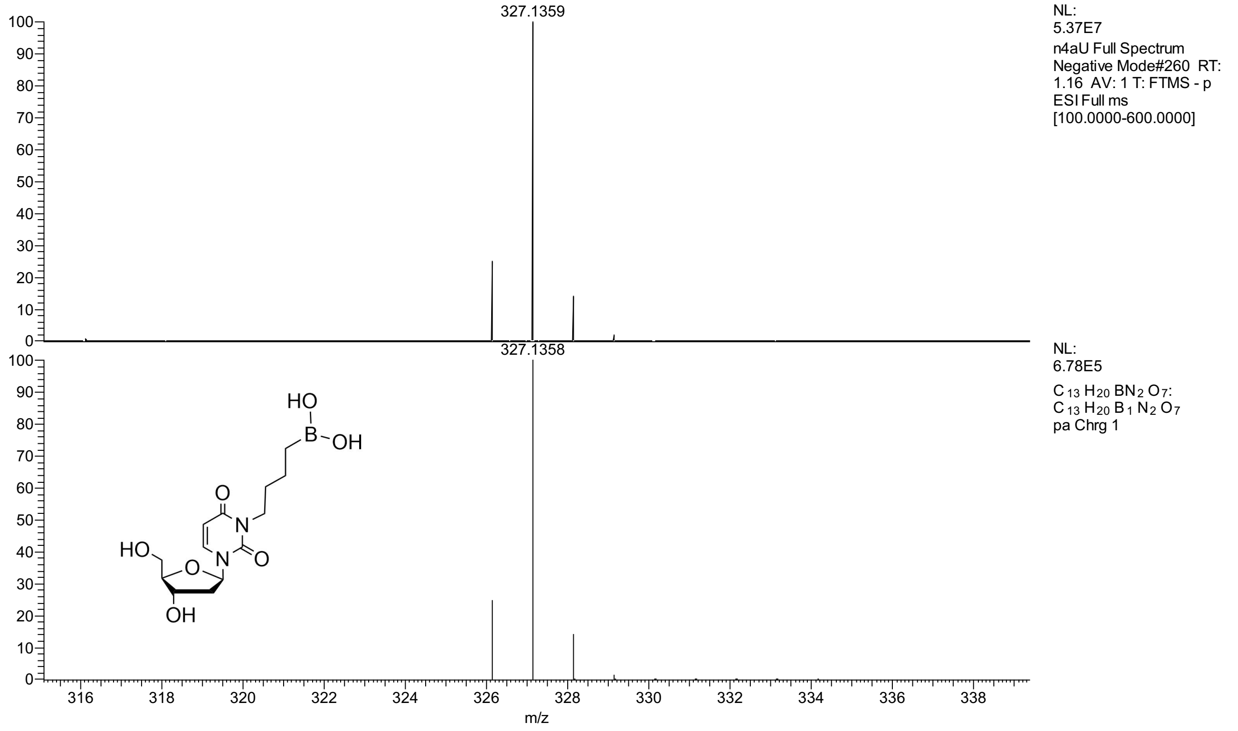

Figure S77 HRMS (ESI-) negative mode m/z calculated for **N4aU** [$C_{13}H_{20}BN_2O_7$] $[M-H]^-$ 327.1358, found 327.1359.

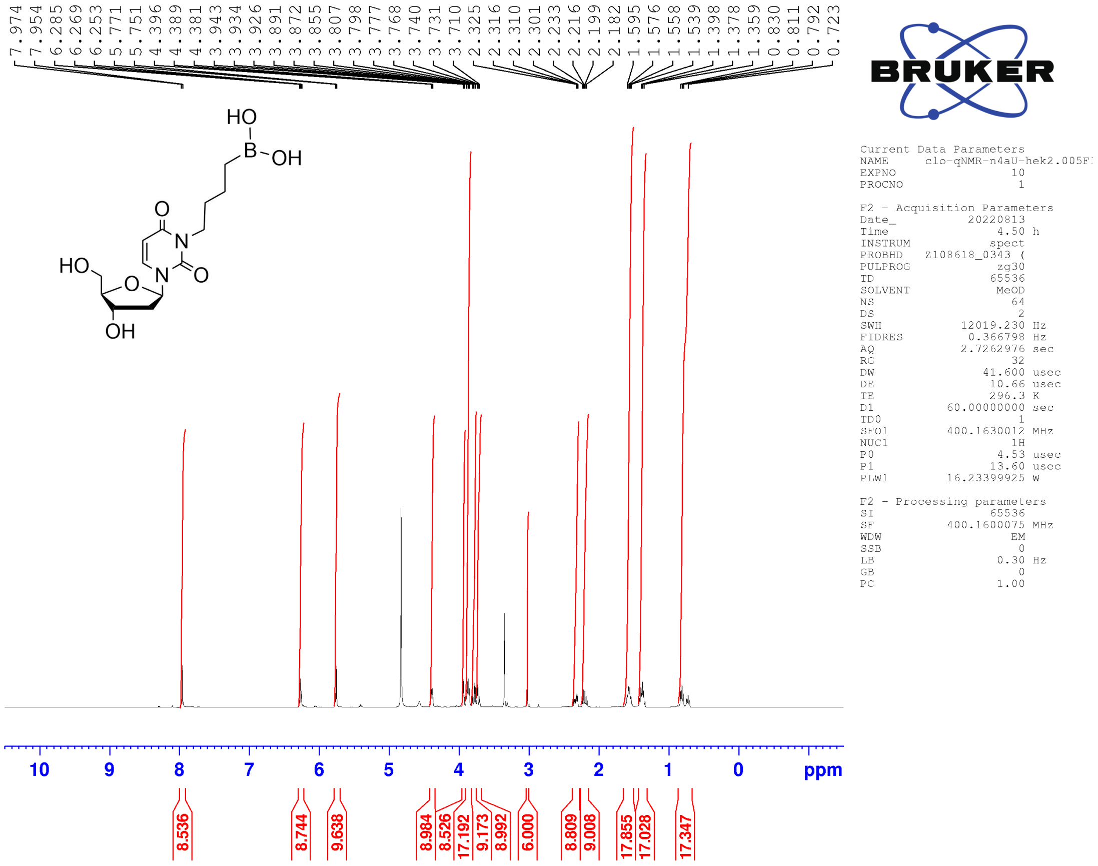

Figure S78 ^{1}H qNMR purity spectrum of **N4aU** with Me_2SO_2 (I.C) in CD_3OD. Purity 96.81%.

$$P_{\text{sample}} = \frac{S_{\text{sample}} \text{ x } N_{\text{std}} \text{ x } m_{\text{std}} \text{ x } M_{\text{sample}}}{S_{\text{std}} \text{ x } N_{\text{sample}} \text{ x } m_{\text{sample}} \text{ x } M_{\text{std}}} \text{ x } P_{\text{std}}$$

$$= \frac{8.744 \text{ x } 6 \text{ x } 1.9 \text{ mg x } 328.13 \text{ g mol}^{-1}}{6 \text{ x } 1 \text{ x } 59.8 \text{ mg x } 94.13 \text{ g mol}^{-1}} \text{ x } 99.96$$

$$= 96.81\%$$

S = Integrated area of the peak
N = Number of protons represented
m = Prepared mass
M = Molecular weight
P = Purity

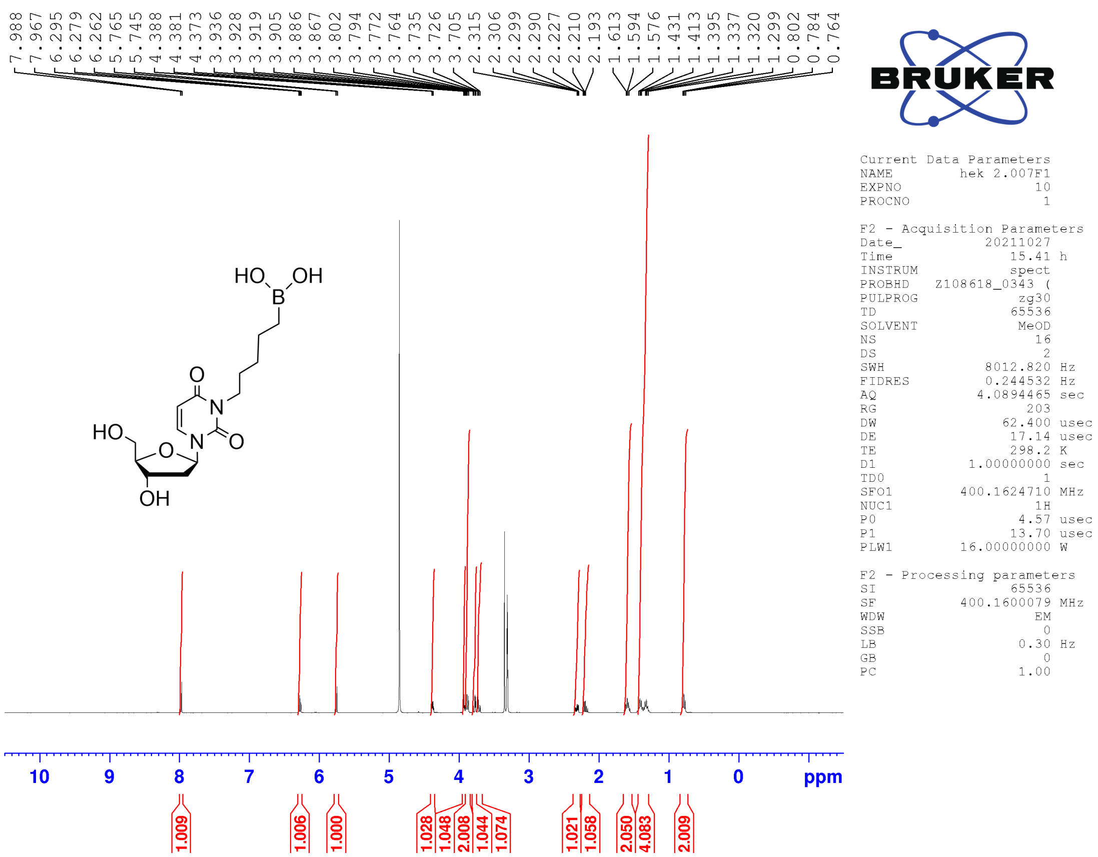

Figure S79 ^{1}HNMR spectra of **N5aU** in CD_3OD.

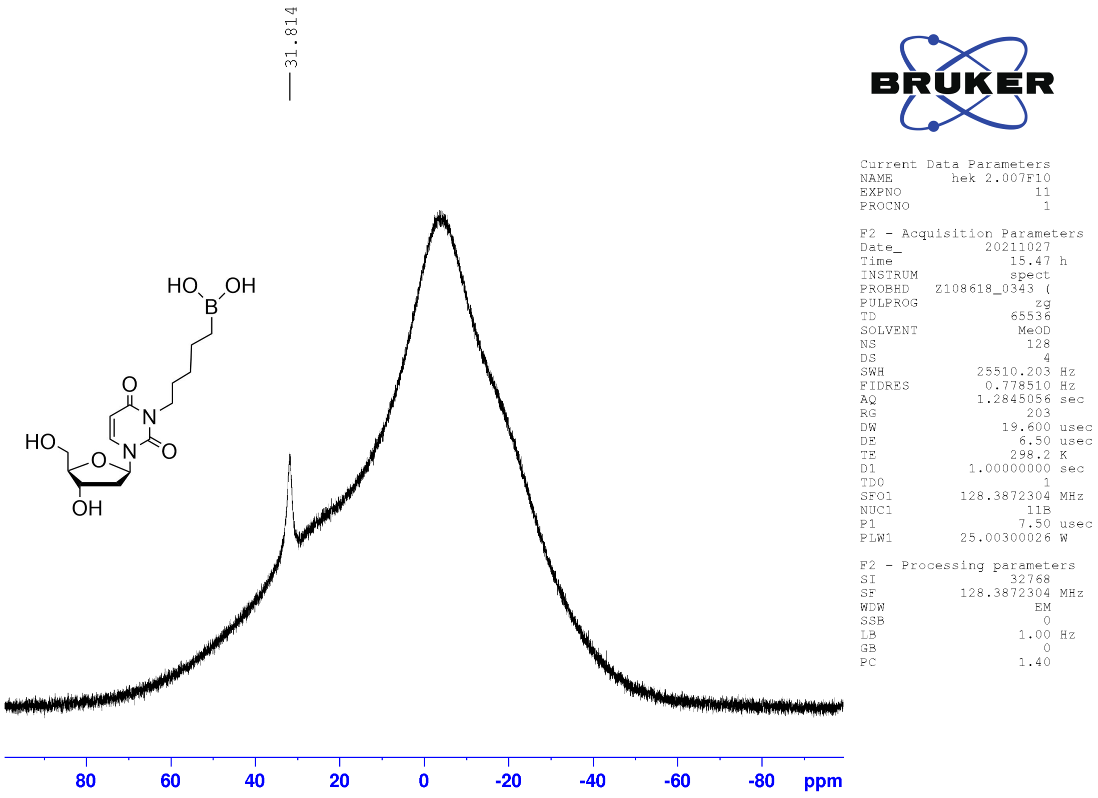

Figure S80 ^{11}BNMR spectra of **N5aU** in CD_3OD.

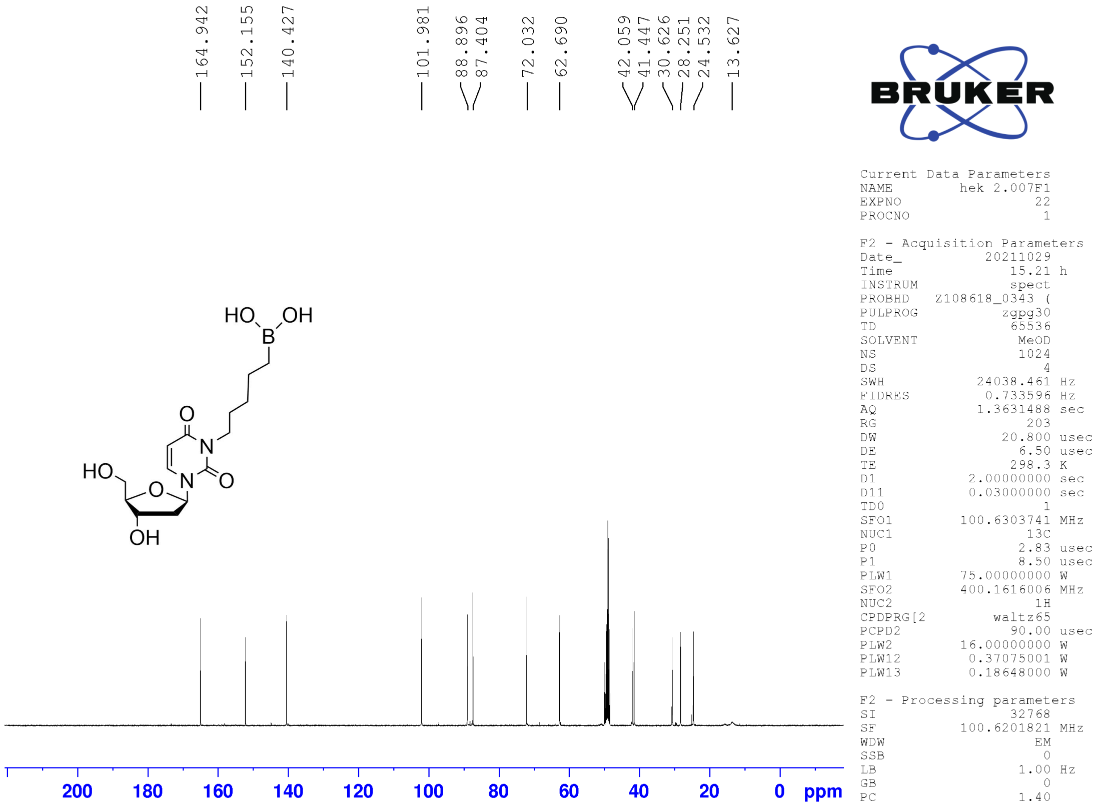

Figure S81 $^{13}C\{^{1}H\}$-NMR spectra of **N5aU** in CD_3OD.

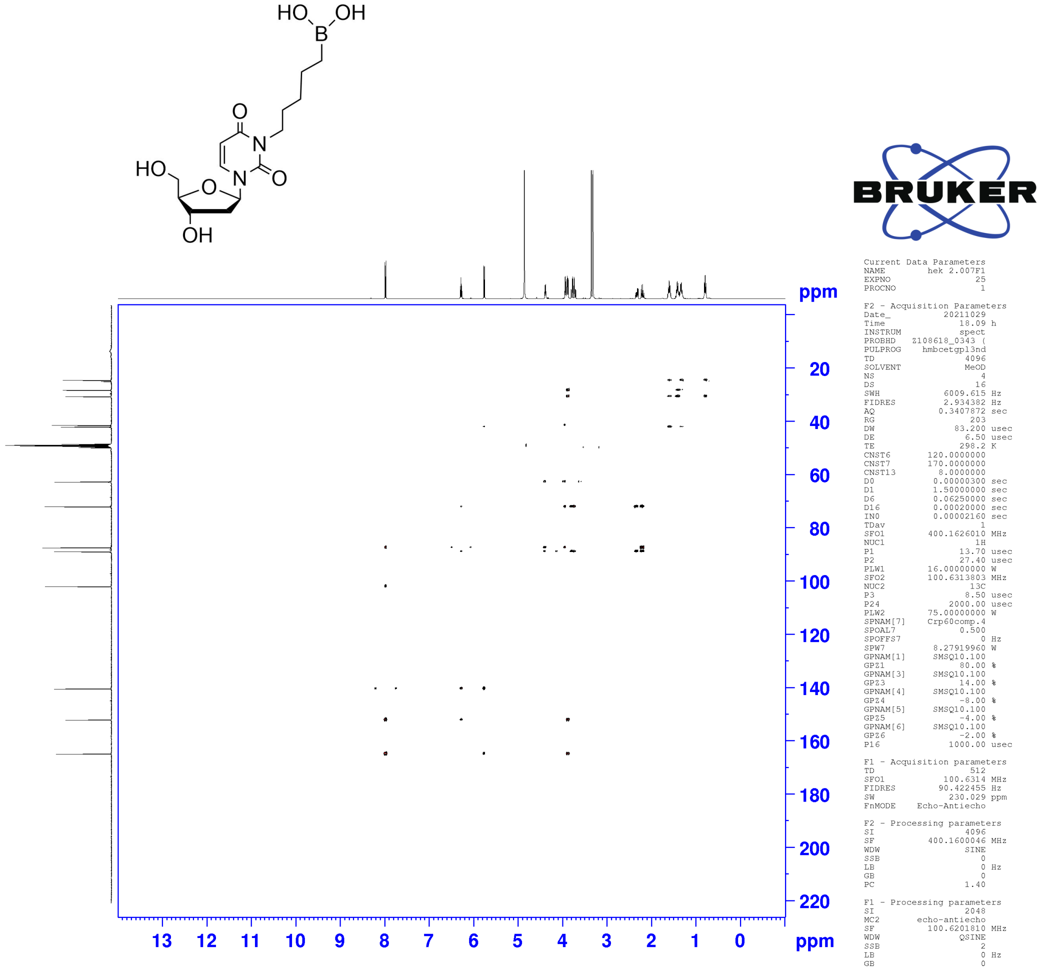

Figure S82 HMBC-NMR spectra of **N5aU** in CD_3OD.

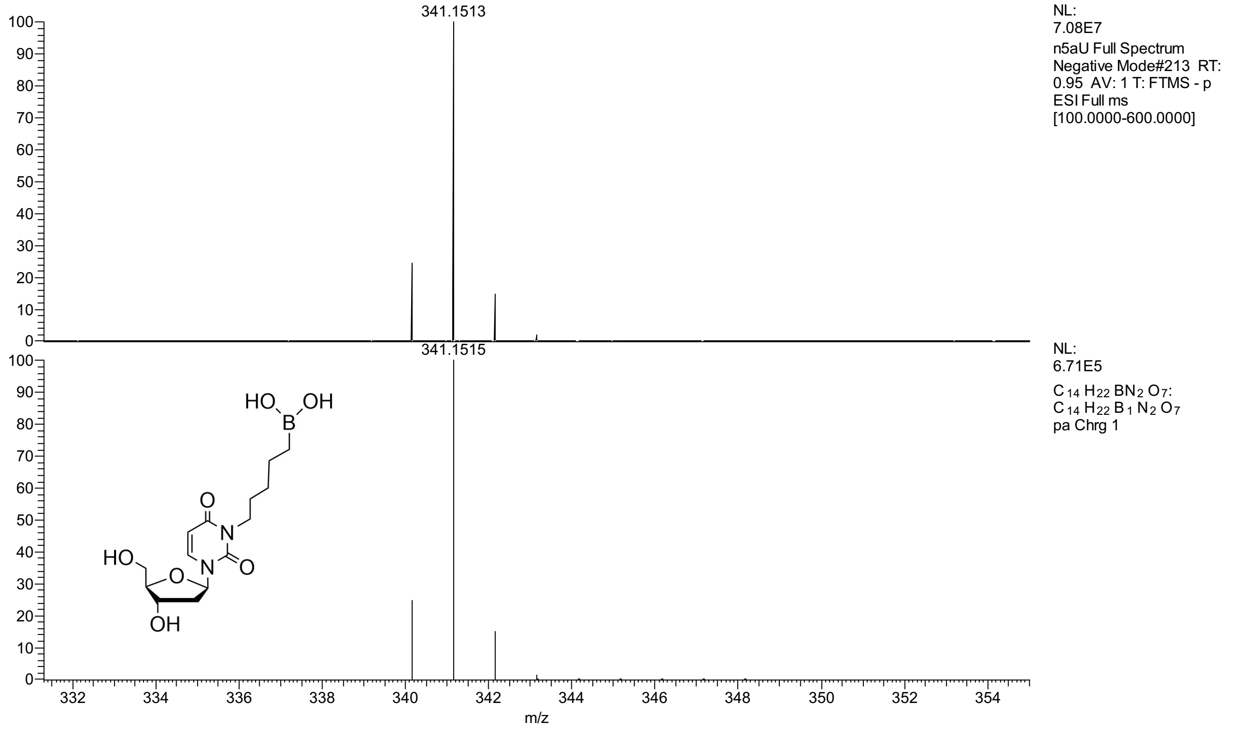

Figure S83 HRMS (ESI-) negative mode m/z calculated for **N5aU** [$C_{14}H_{22}BN_2O_7$] $[M-H]^-$ 341.1515, found 341.1513.

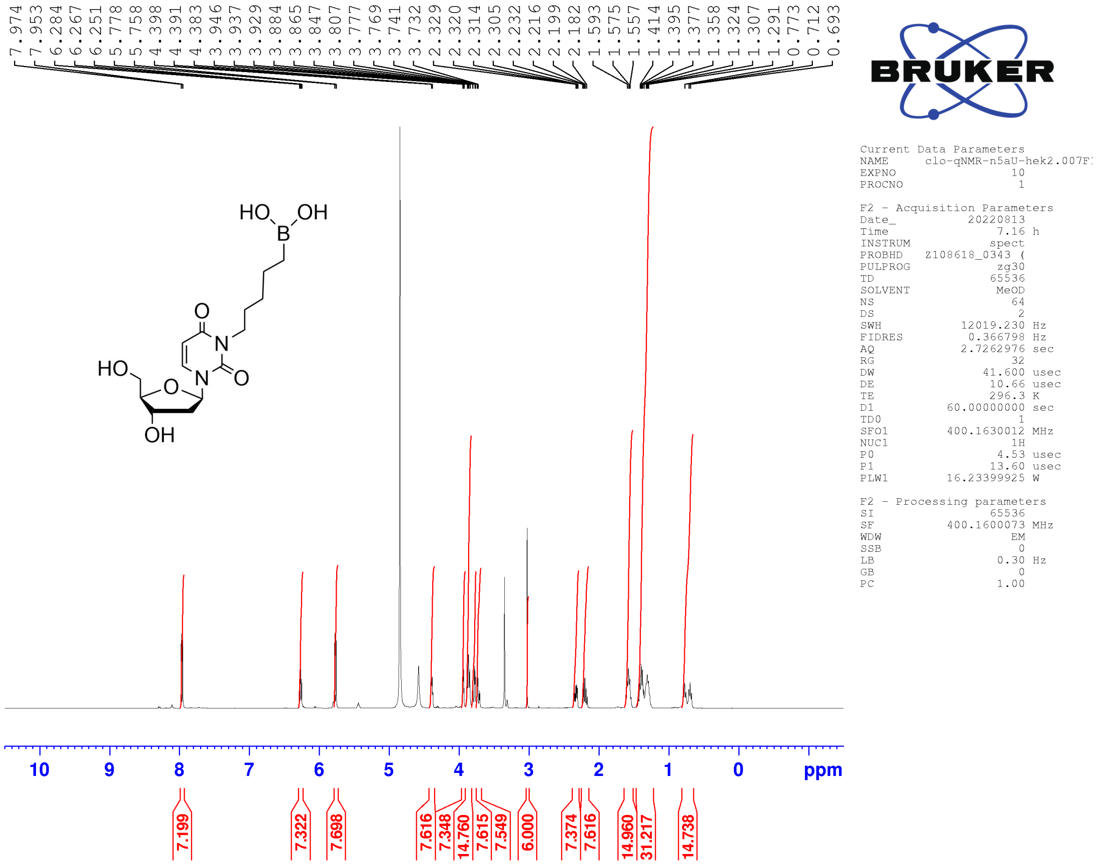

Figure S84 ^{1}H qNMR purity spectrum of **N5aU** with Me_2SO_2 (I.C) in CD_3OD. Purity 96.27%.

$$P_{\text{sample}} = \frac{S_{\text{sample}} \text{ x } N_{\text{std}} \text{ x } m_{\text{std}} \text{ x } M_{\text{sample}}}{S_{\text{std}} \text{ x } N_{\text{sample}} \text{ x } m_{\text{sample}} \text{ x } M_{\text{std}}} \text{ x } P_{\text{std}}$$

$$= \frac{7.322 \text{ x } 6 \text{ x } 2.2 \text{ mg x } 342.16 \text{ g mol}^{-1}}{6 \text{ x } 1 \text{ x } 60.8 \text{ mg x } 94.13 \text{ g mol}^{-1}} \text{ x } 99.96$$

$$= 96.27\%$$

S = Integrated area of the peak
N = Number of protons represented
m = Prepared mass
M = Molecular weight
P = Purity

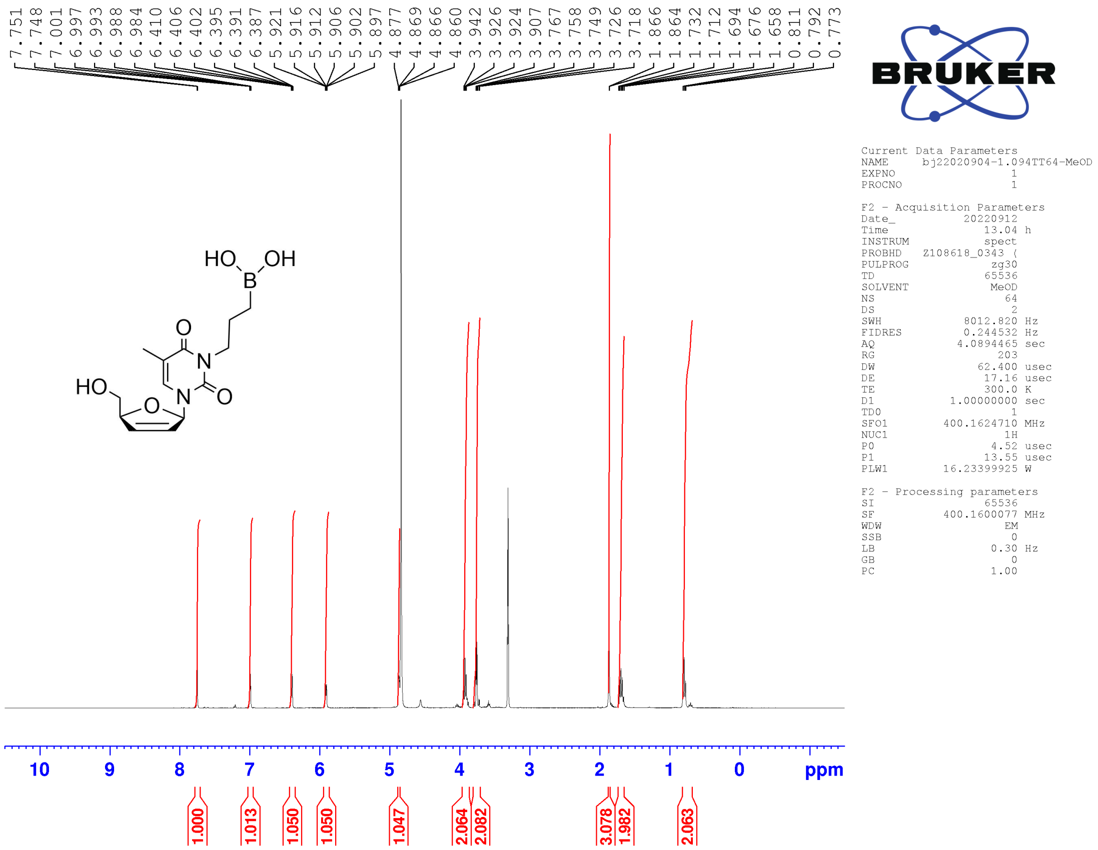

Figure S85 ^{1}HNMR spectra of **N3aS** in CD_3OD.

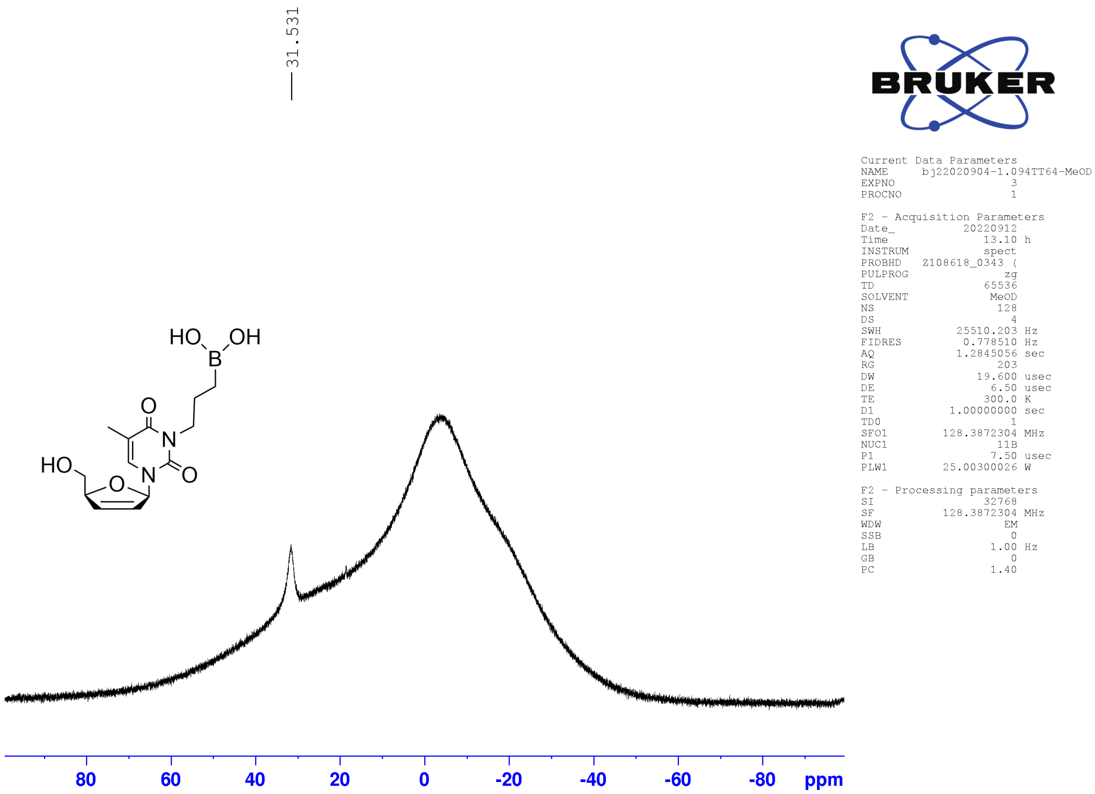

Figure S86 ^{11}BNMR spectra of **N3aS** in CD_3OD.

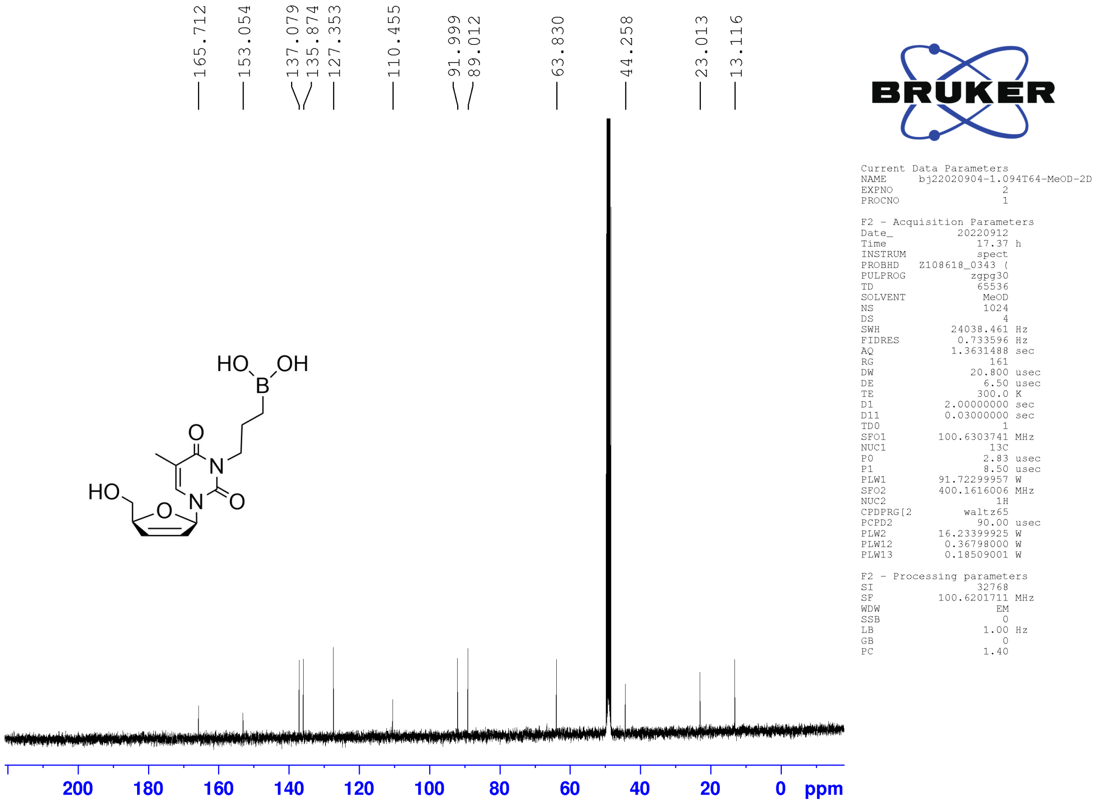

Figure S87 $^{13}C\{^{1}H\}$-NMR spectra of **N3aS** in CD_3OD.

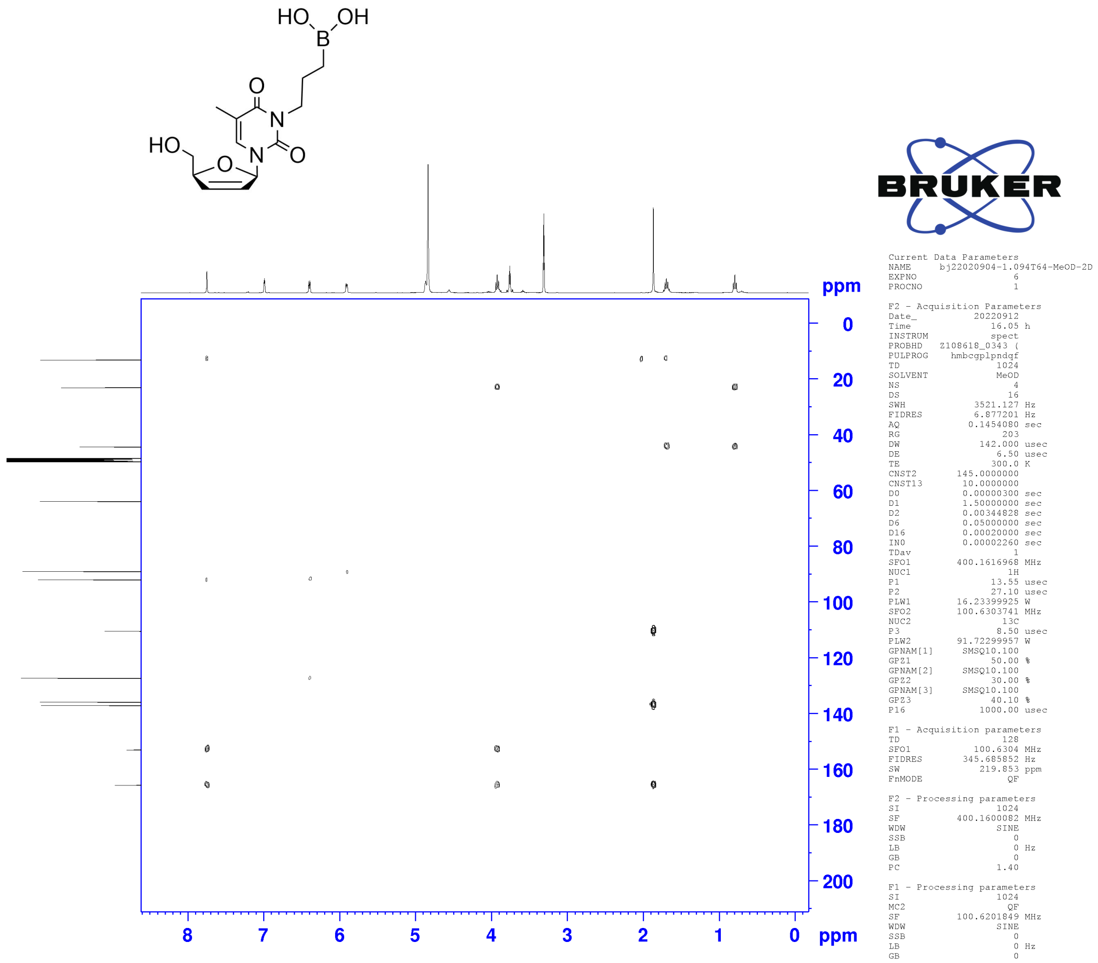

Figure S88 HMBC-NMR spectra of **N3aS** in CD_3OD.

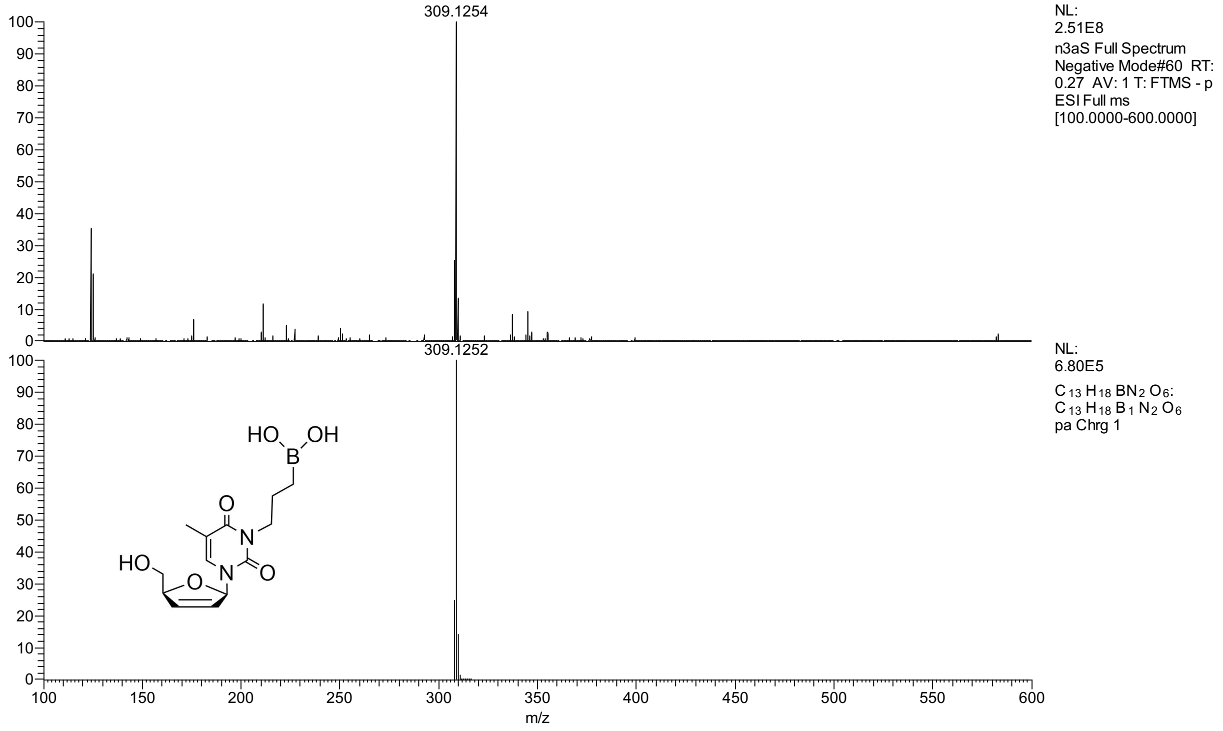

Figure S89 HRMS (ESI-) negative mode m/z calculated for **N3aS** [$C_{13}H_{18}BN_2O_6$] $[M-H]^-$ 309.1252, found 309.1254.

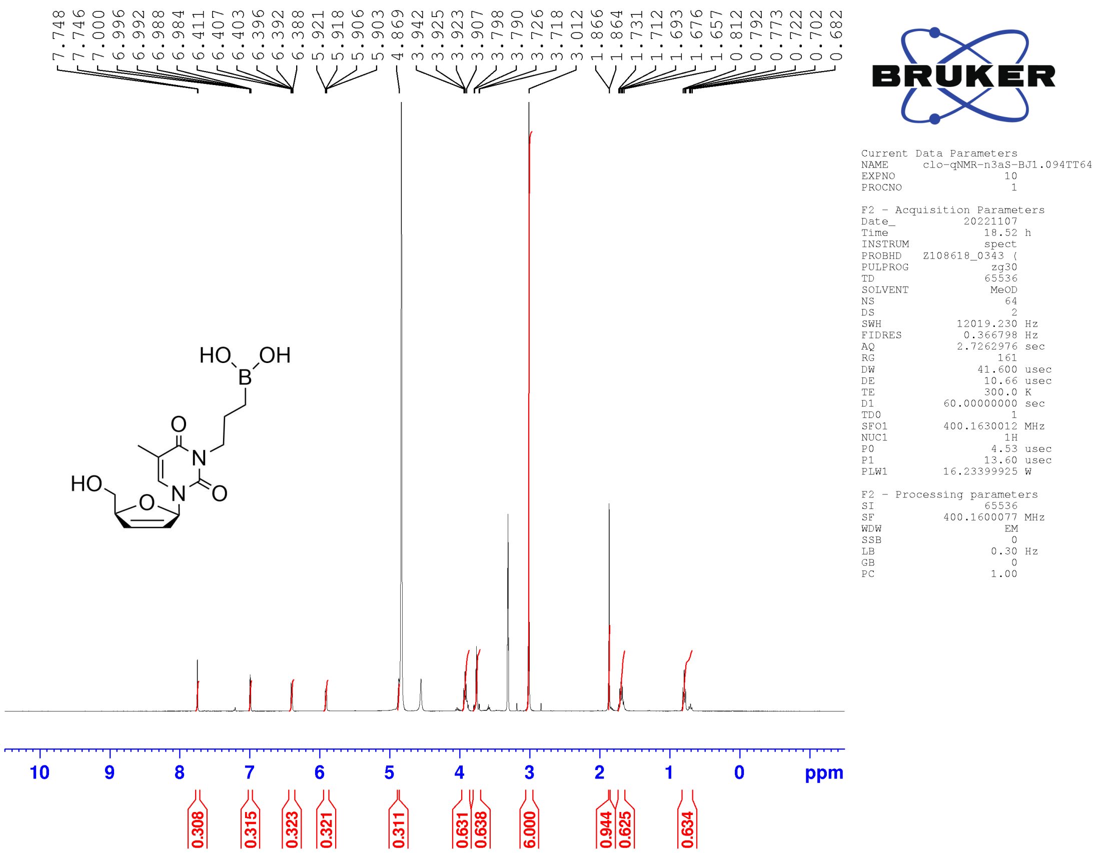

Figure S90 ^{1}H qNMR purity spectrum of **N3aS** with Me_2SO_2 (I.C) in CD_3OD. Purity 96.99%.

$$P_{\text{sample}} = \frac{S_{\text{sample}} \text{ x } N_{\text{std}} \text{ x } m_{\text{std}} \text{ x } M_{\text{sample}}}{S_{\text{std}} \text{ x } N_{\text{sample}} \text{ x } m_{\text{sample}} \text{ x } M_{\text{std}}} \text{ x } P_{\text{std}}$$

$$= \frac{0.321 \text{ x } 6 \text{ x } 3.3 \text{ mg x } 310.11 \text{ g mol}^{-1}}{6 \text{ x } 1 \text{ x } 3.6 \text{ mg x } 94.13 \text{ g mol}^{-1}} \text{ x } 99.96$$

$$= 96.99\%$$

S = Integrated area of the peak
N = Number of protons represented
m = Prepared mass
M = Molecular weight
P = Purity

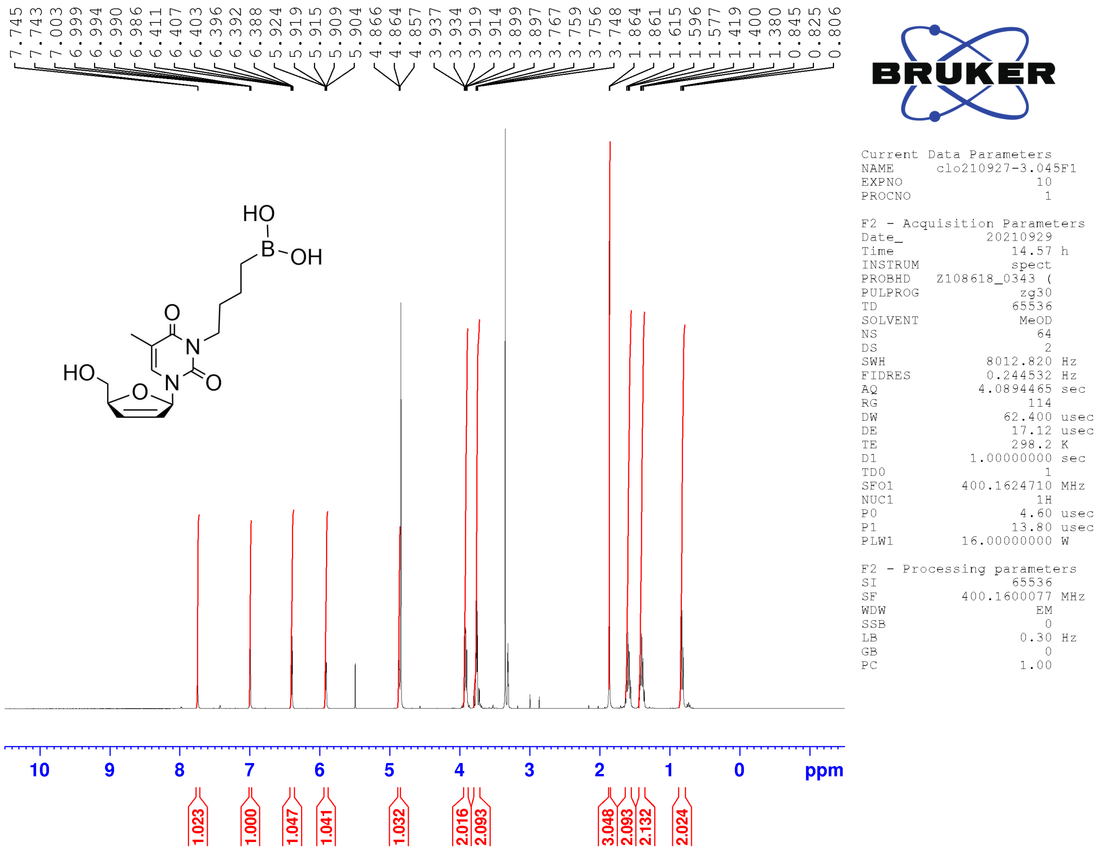

Figure S91 ^{1}HNMR spectra of **N4aS** in CD_3OD.

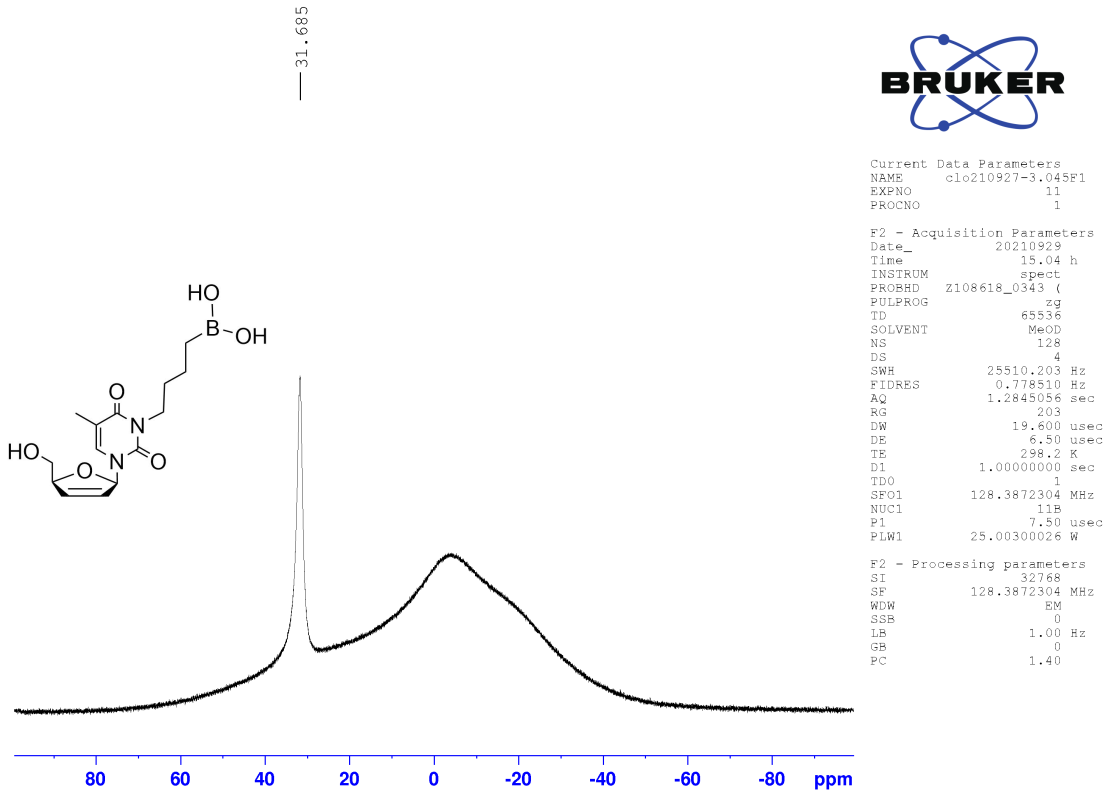

Figure S92 ^{11}BNMR spectra of **N4aS** in CD_3OD.

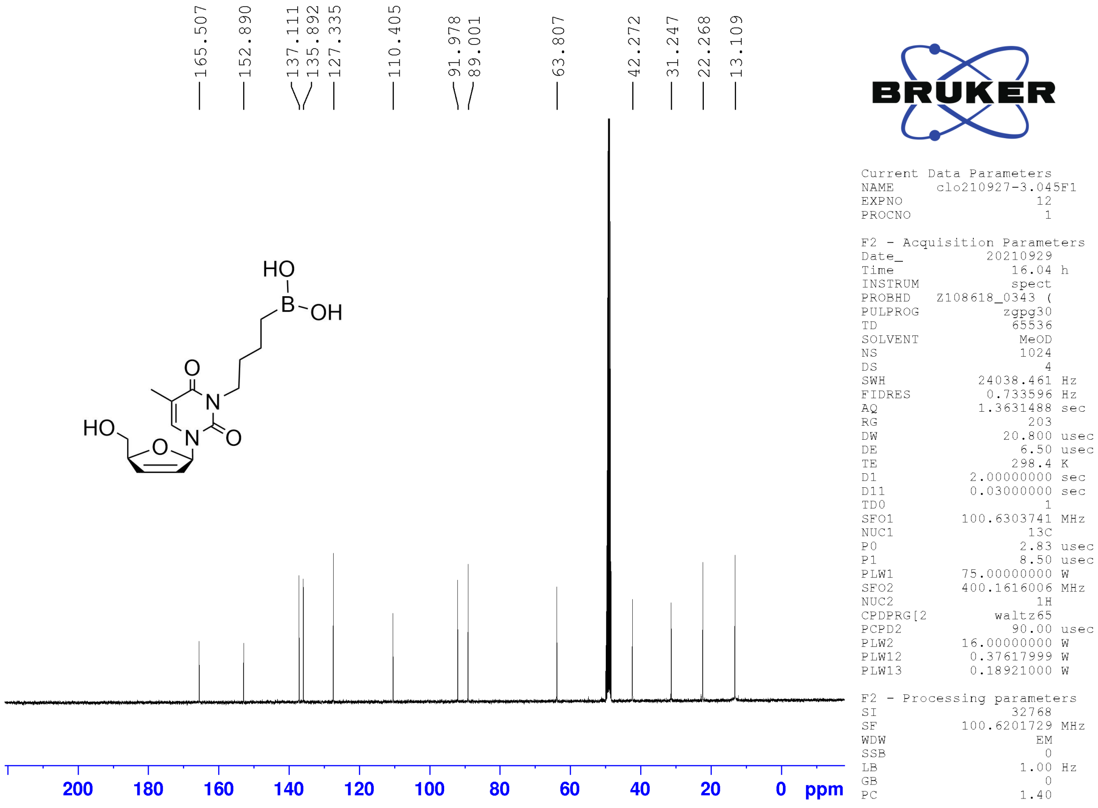

Figure S93 $^{13}C\{^{1}H\}$-NMR spectra of **N4aS** in CD_3OD.

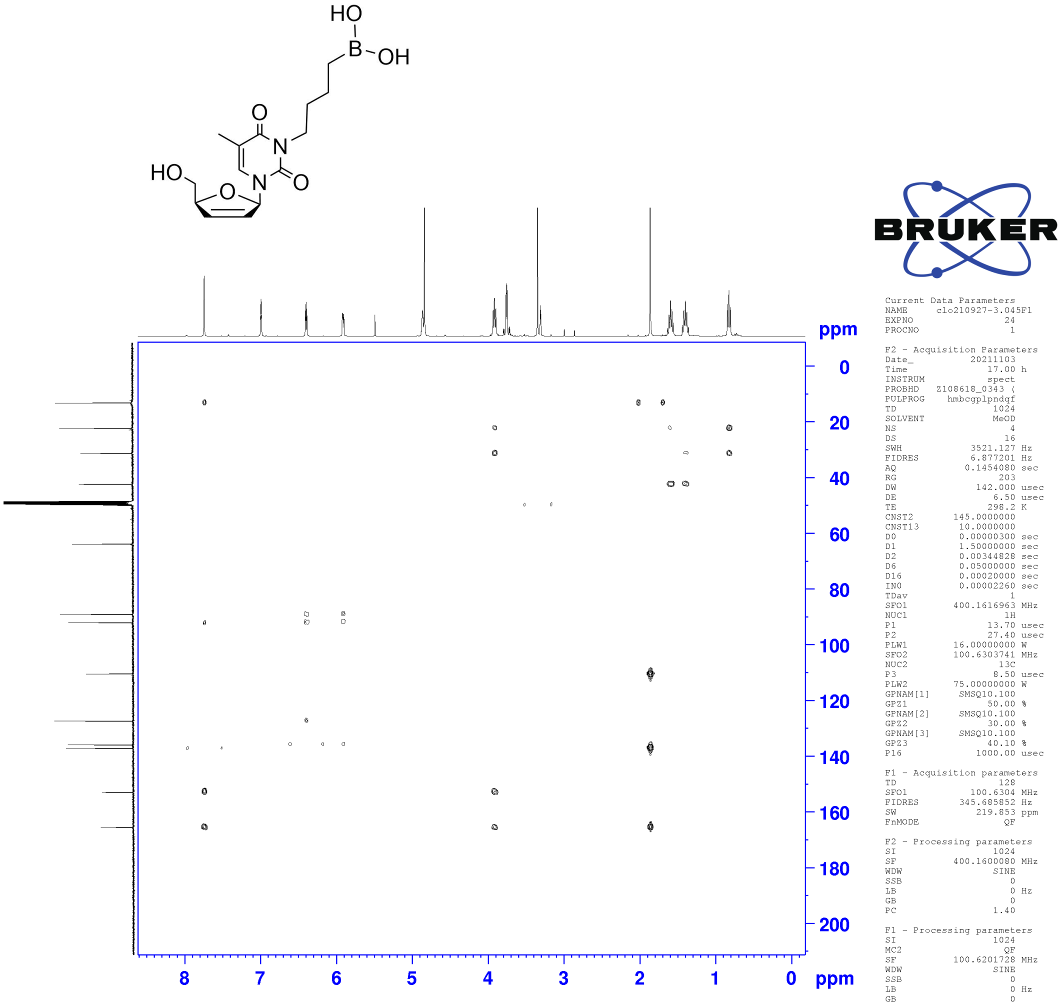

Figure S94 HMBC-NMR spectra of **N4aS** in CD_3OD.

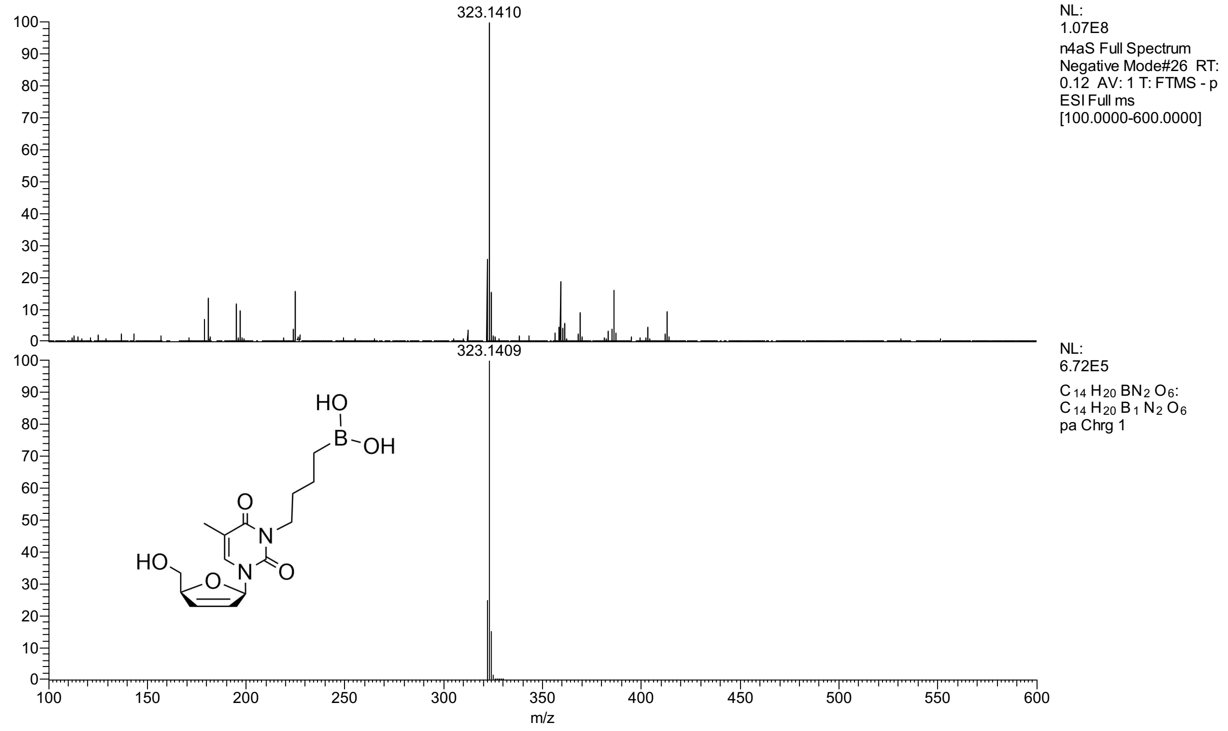

Figure S95 HRMS (ESI-) negative mode m/z calculated for **N4aS** [$C_{14}H_{20}BN_2O_6$] [M-H]$^-$ 323.1409, found 323.1410.

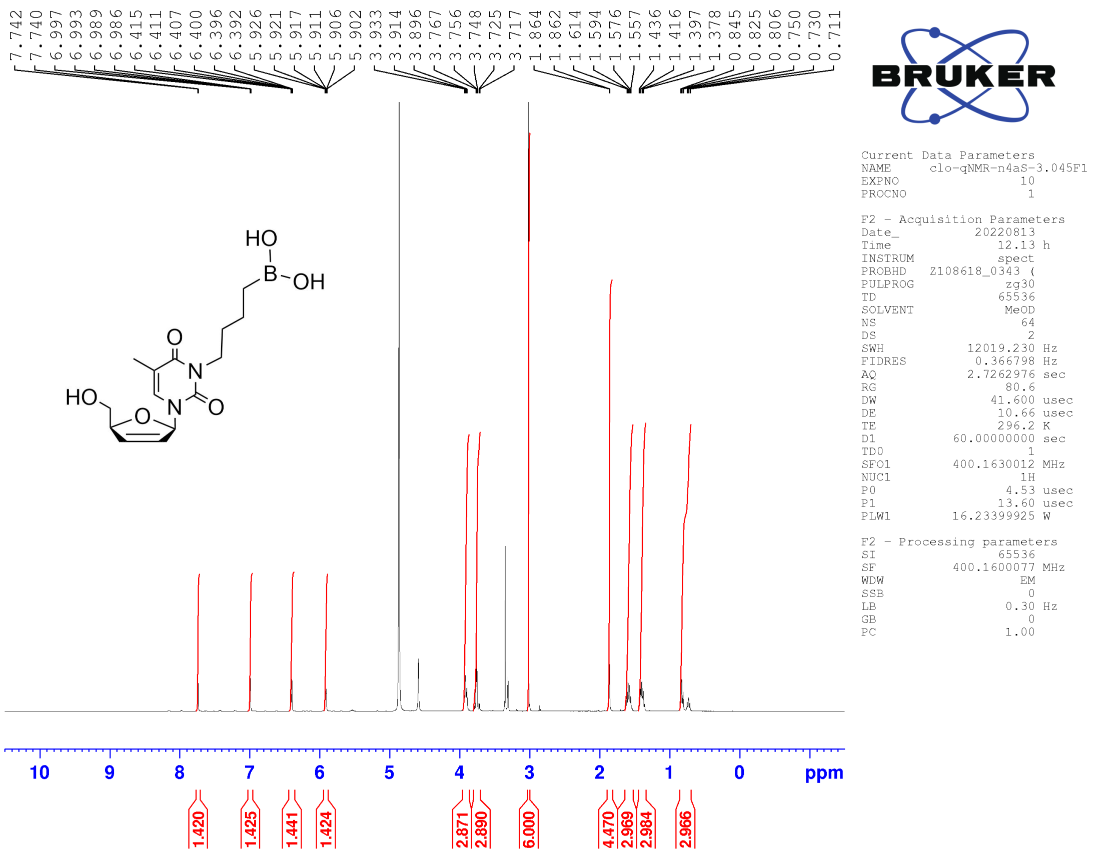

Figure S96 ^{1}H qNMR purity spectrum of **N4aS** with Me_2SO_2 (I.C) in CD_3OD. Purity 96.28%.

$$P_{\text{sample}} = \frac{S_{\text{sample}} \text{ x } N_{\text{std}} \text{ x } m_{\text{std}} \text{ x } M_{\text{sample}}}{S_{\text{std}} \text{ x } N_{\text{sample}} \text{ x } m_{\text{sample}} \text{ x } M_{\text{std}}} \text{ x } P_{\text{std}}$$

$$= \frac{1.424 \text{ x } 6 \text{ x } 4.4 \text{ mg x } 324.14 \text{ g mol}^{-1}}{6 \text{ x } 1 \text{ x } 22.4 \text{ mg x } 94.13 \text{ g mol}^{-1}} \text{ x } 99.96$$

$$= 96.28\%$$

S = Integrated area of the peak
N = Number of protons represented
m = Prepared mass
M = Molecular weight
P = Purity

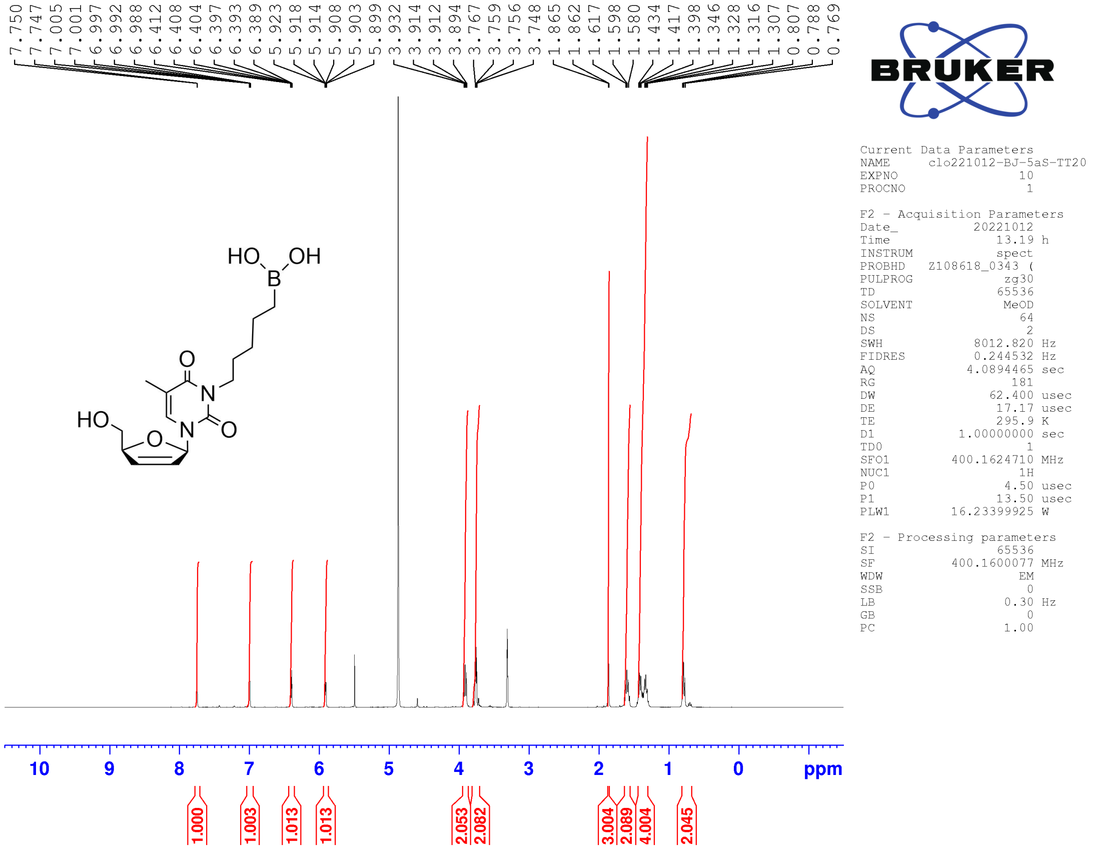

Figure S97 ^{1}HNMR spectra of **N5aS** in CD_3OD.

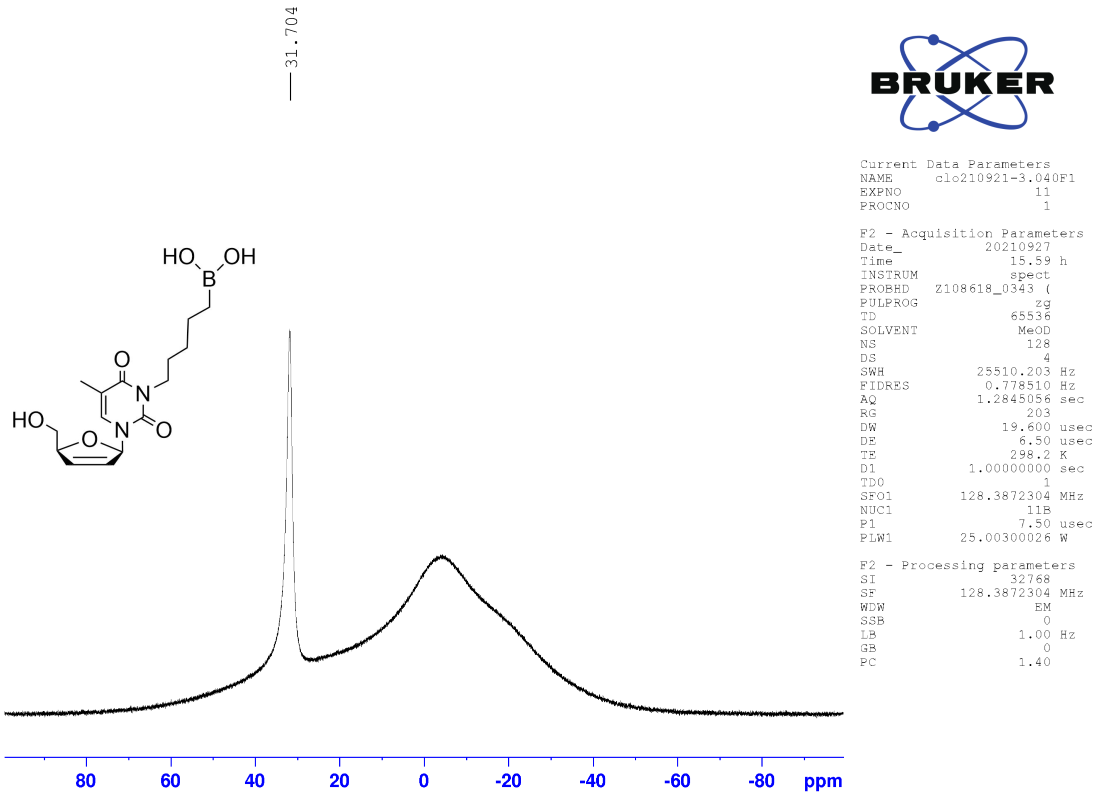

Figure S98 ^{11}BNMR spectra of **N5aS** in CD_3OD.

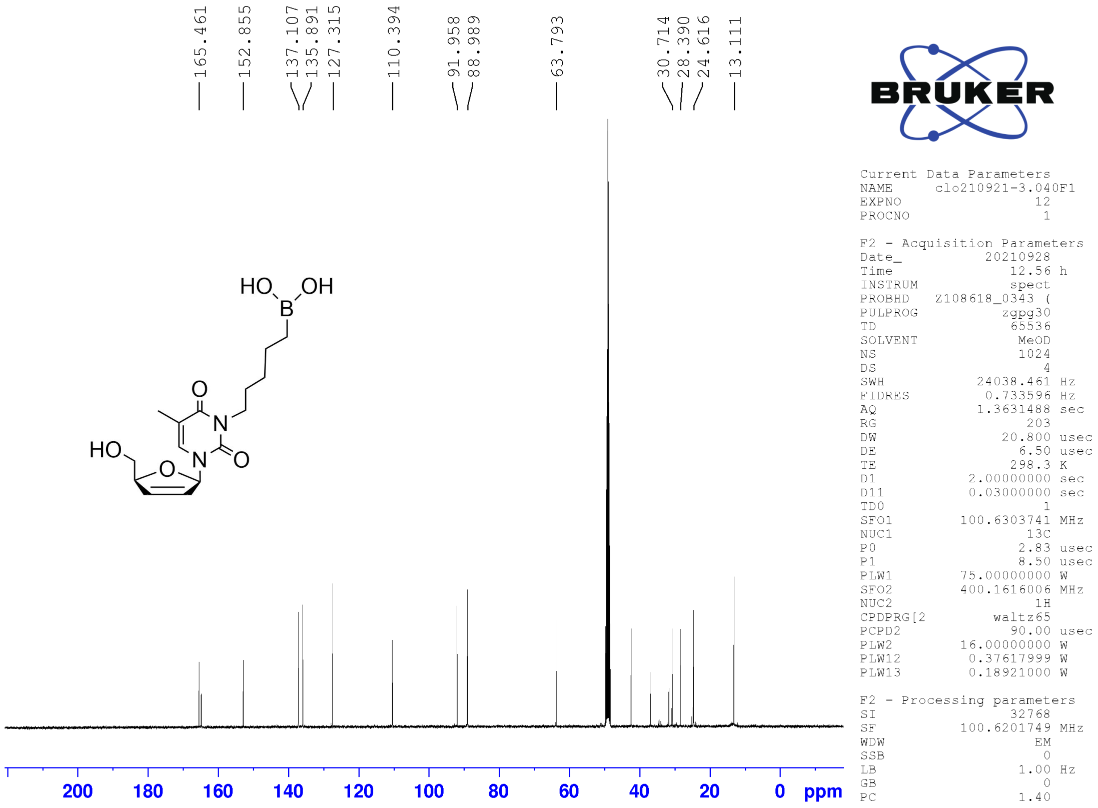

Figure S99 $^{13}C\{^1H\}$-NMR spectra of **N5aS** in CD_3OD.

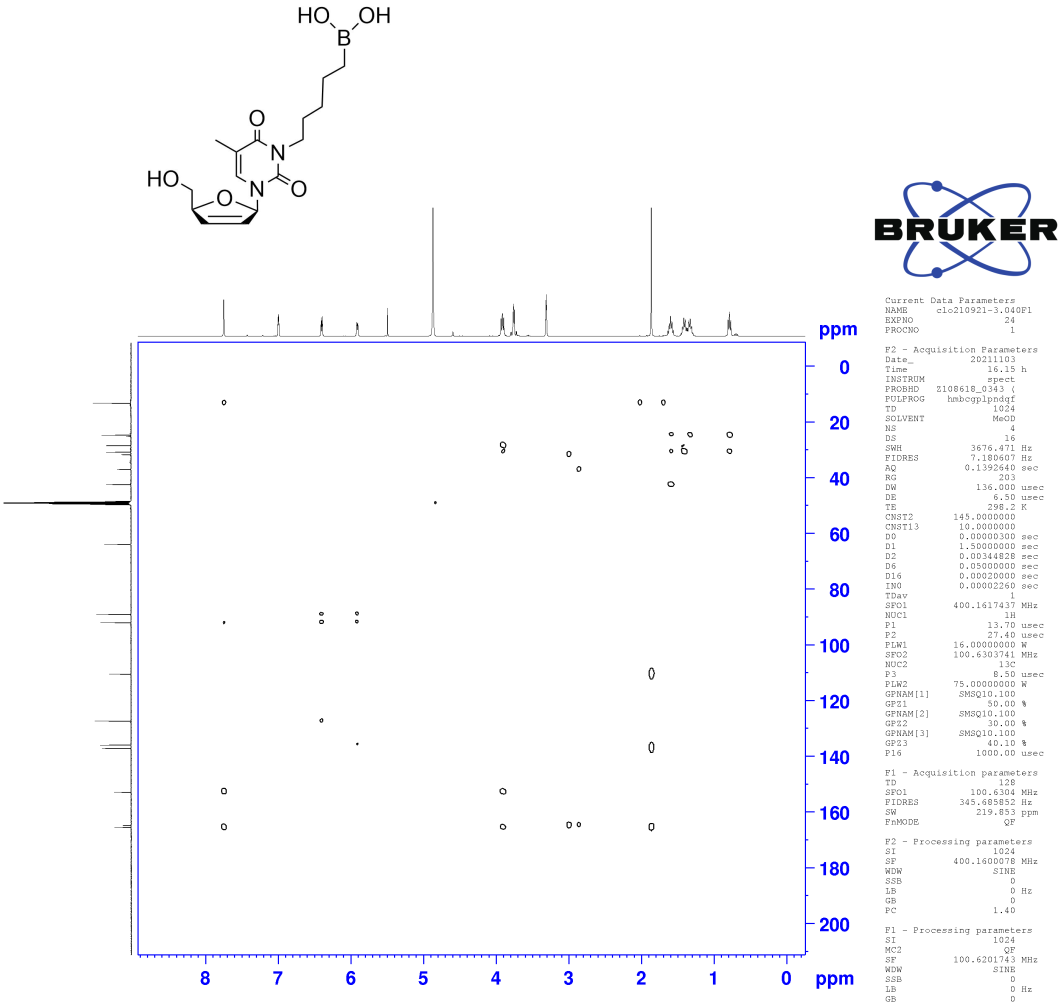

Figure S100 HMBC-NMR spectra of **N5aS** in CD_3OD.

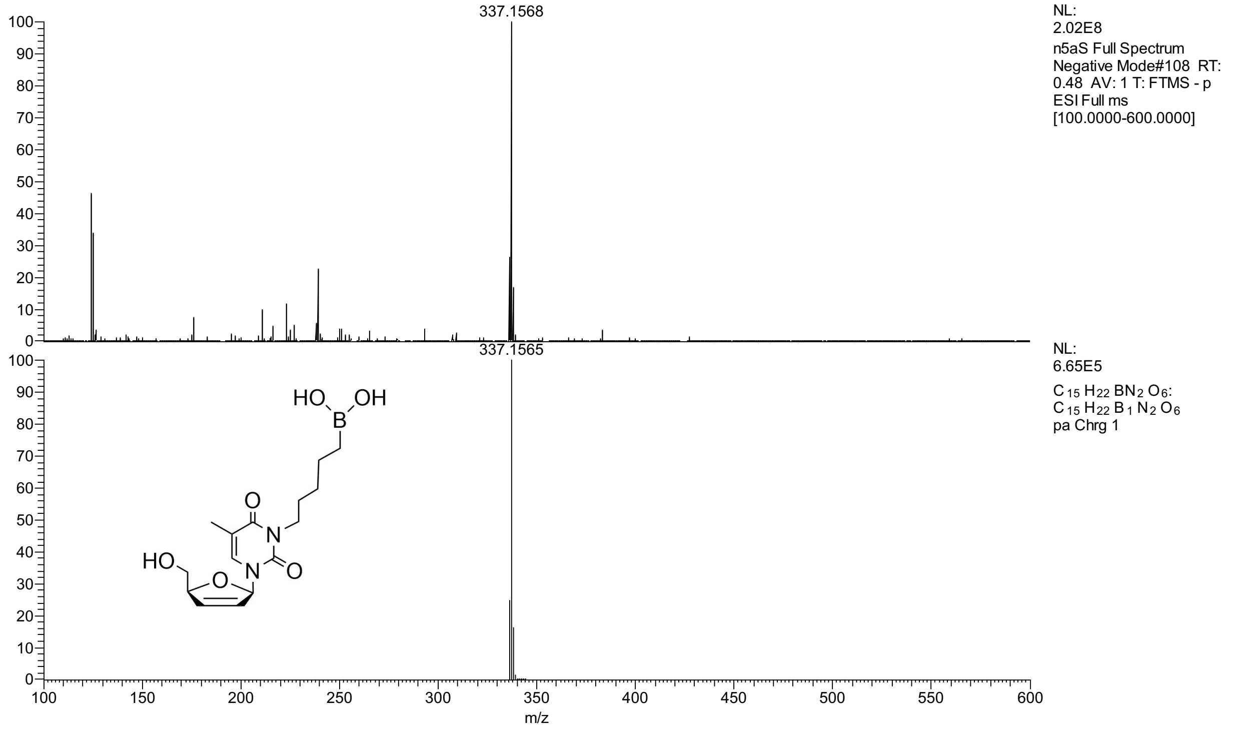

Figure S101 HRMS (ESI-) negative mode m/z calculated for **N5aS** [$C_{15}H_{22}BN_2O_6$] [M-H]$^-$ 337.1565, found 337.1568.

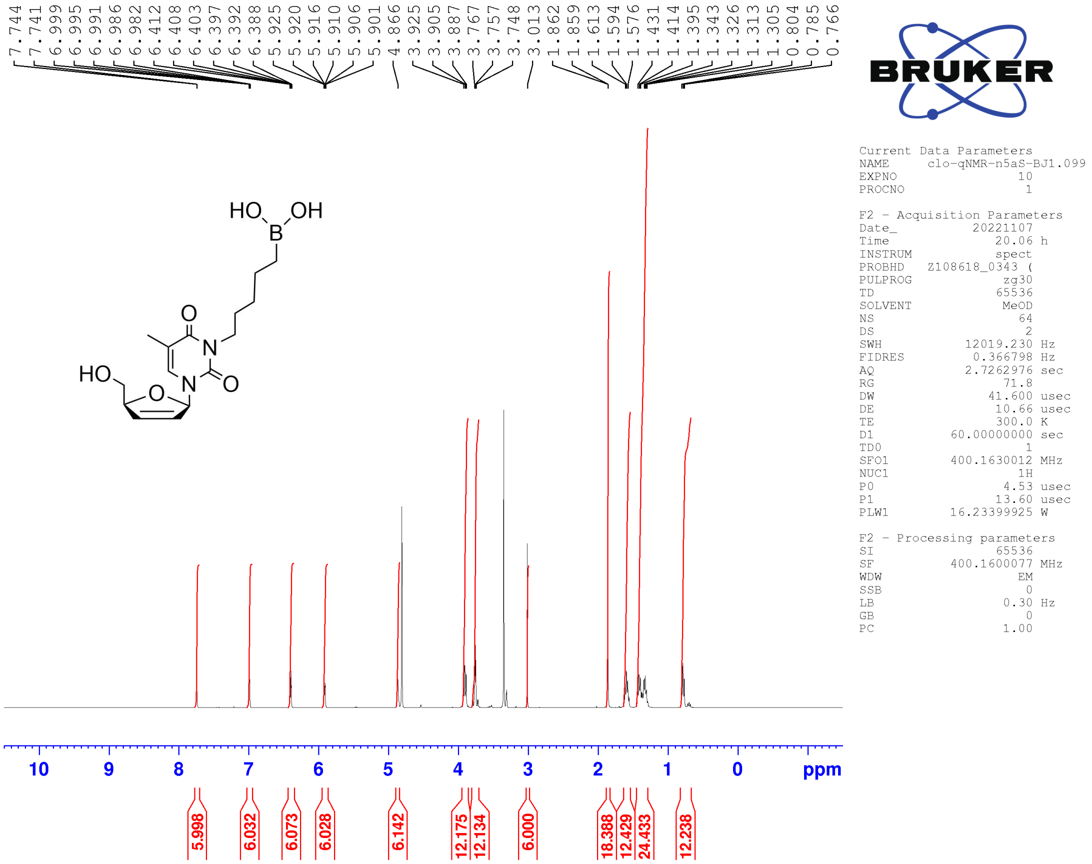

Figure S102 ^{1}H qNMR purity spectrum of **N5aS** with Me_2SO_2 (I.C) in CD_3OD. Purity 96.71%.

$$P_{\text{sample}} = \frac{S_{\text{sample}} \text{ x } N_{\text{std}} \text{ x } m_{\text{std}} \text{ x } M_{\text{sample}}}{S_{\text{std}} \text{ x } N_{\text{sample}} \text{ x } m_{\text{sample}} \text{ x } M_{\text{std}}} \text{ x } P_{\text{std}}$$

$$= \frac{6.028 \text{ x } 6 \text{ x } 2.6 \text{ mg x } 338.17 \text{ g mol}^{-1}}{6 \text{ x } 1 \text{ x } 58.2 \text{ mg x } 94.13 \text{ g mol}^{-1}} \text{ x } 99.96$$

$$= 96.71\%$$

S = Integrated area of the peak
N = Number of protons represented
m = Prepared mass
M = Molecular weight
P = Purity

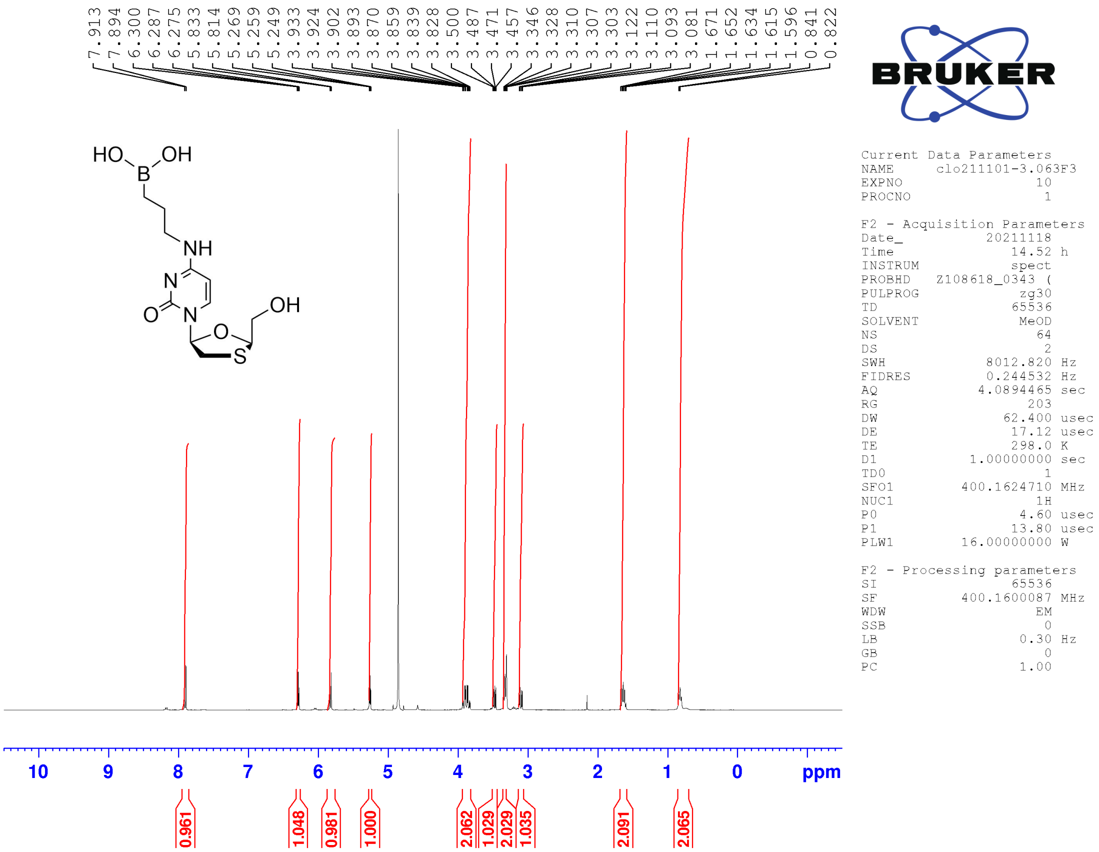

Figure S103 ^{1}HNMR spectra of **N3aL** in CD_3OD.

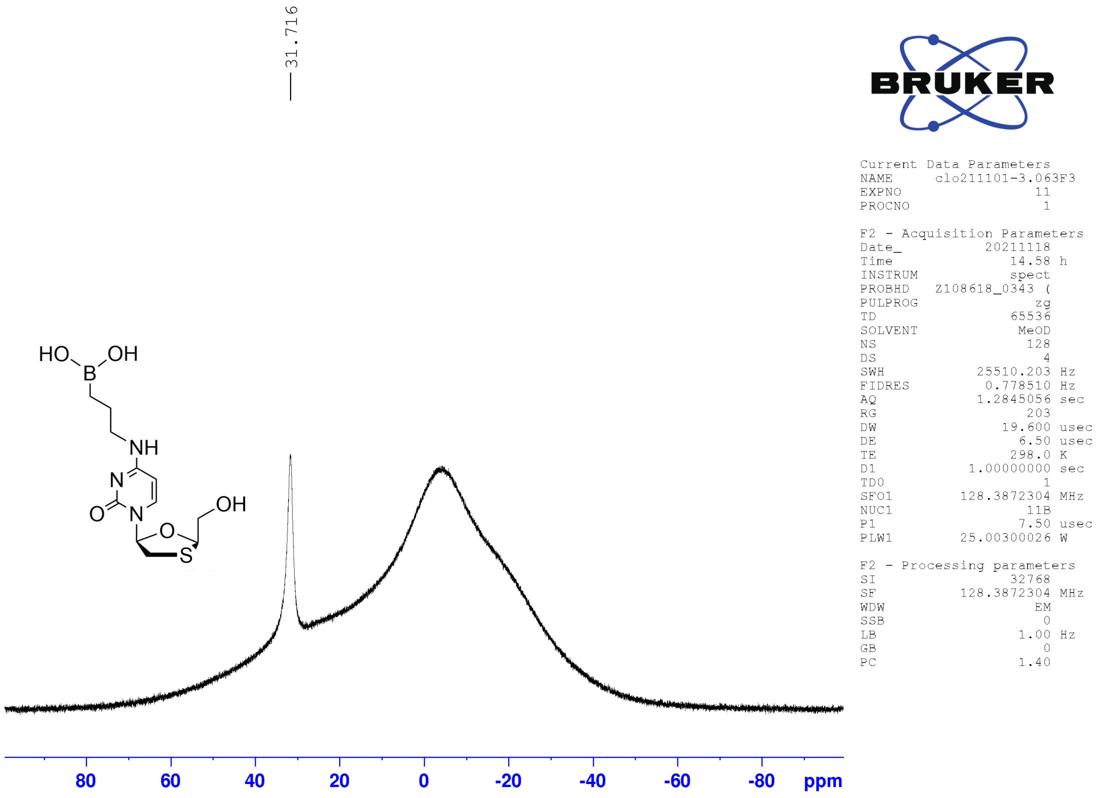

Figure S104 ^{11}BNMR spectra of **N3aL** in CD_3OD.

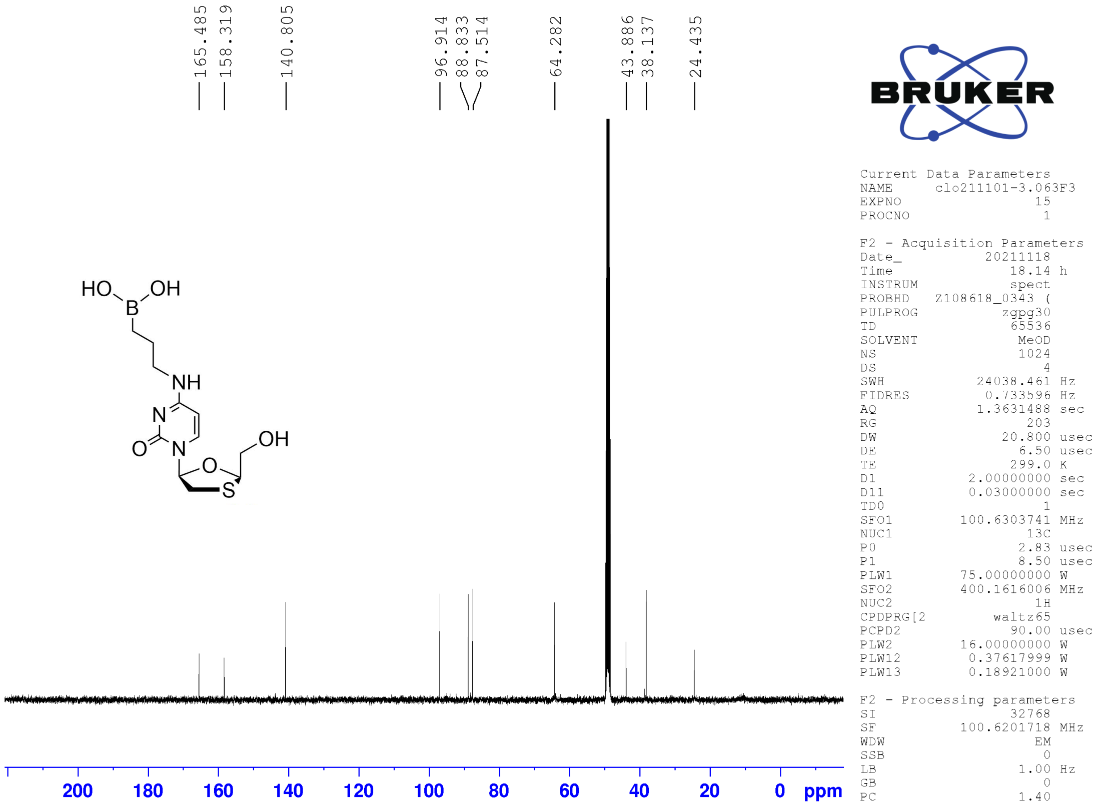

Figure S105 $^{13}C\{^1H\}$-NMR spectra of **N3aL** in CD_3OD.

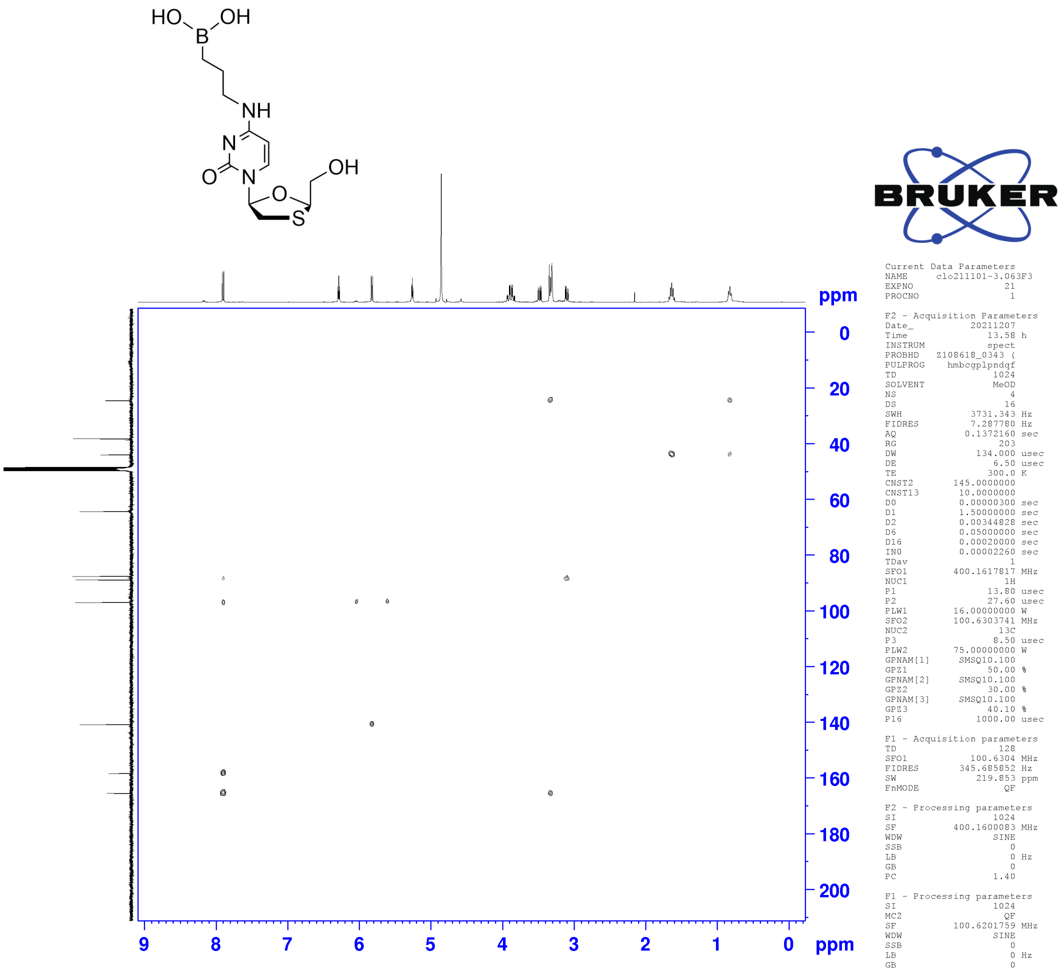

Figure S106 HMBC-NMR spectra of **N3aL** in CD_3OD.

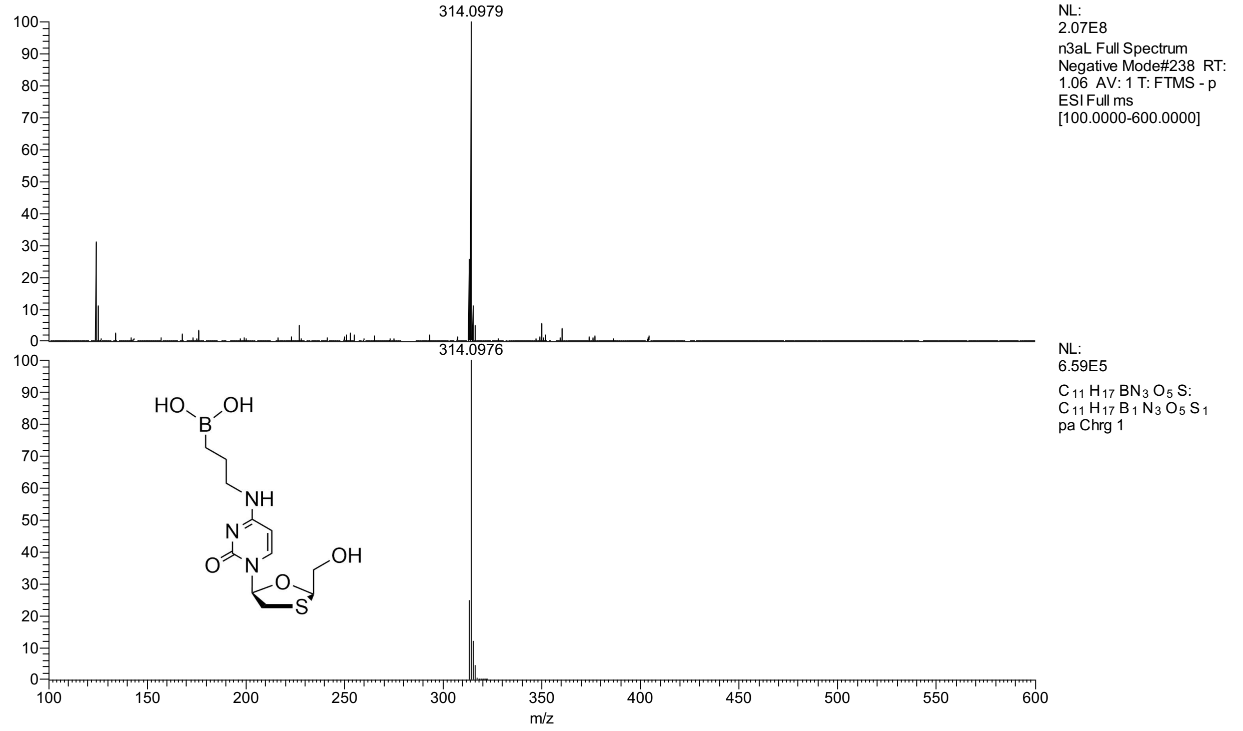

Figure S107 HRMS (ESI-) negative mode m/z calculated for **N3aL** [$C_{11}H_{17}BN_3O_5S$] [M-H]$^-$ 314.0976, found 314.0979.

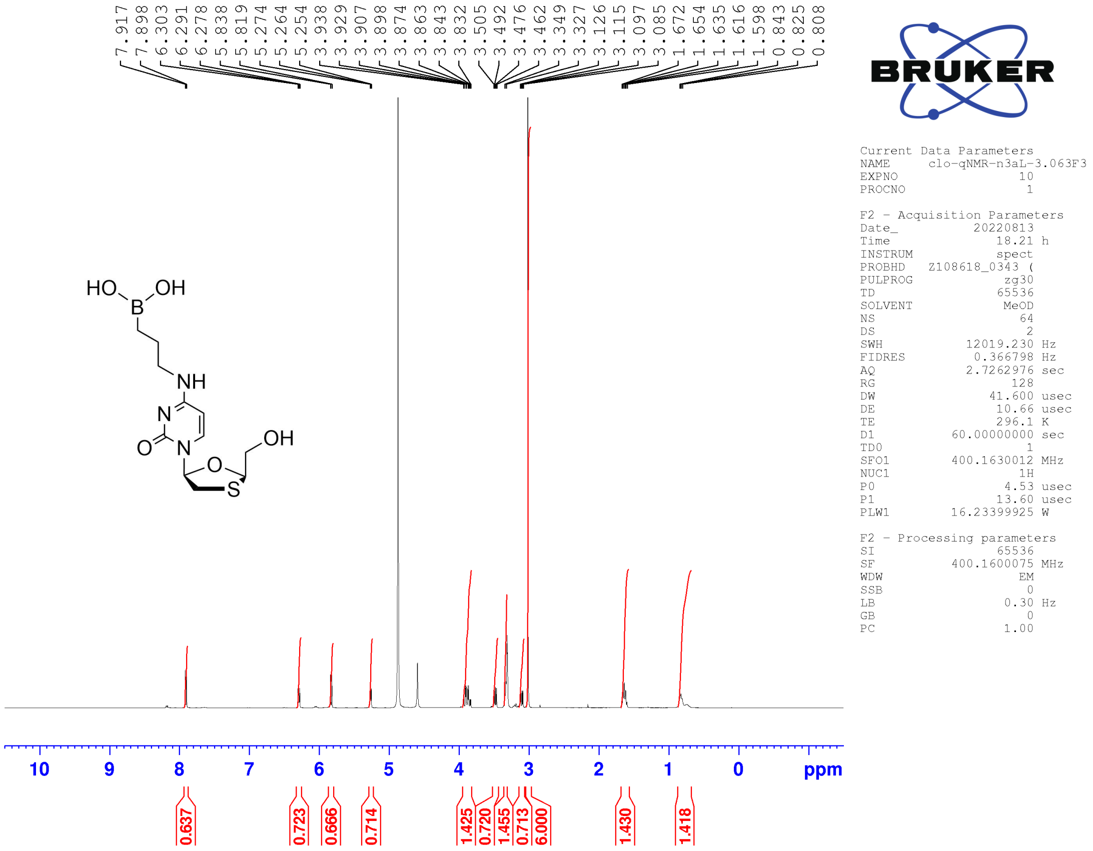

Figure S108 ^{1}H qNMR purity spectrum of **N3aL** with Me_2SO_2 (I.C) in CD_3OD. Purity 96.07%.

$$P_{\text{sample}} = \frac{S_{\text{sample}} \text{ x } N_{\text{std}} \text{ x } m_{\text{std}} \text{ x } M_{\text{sample}}}{S_{\text{std}} \text{ x } N_{\text{sample}} \text{ x } m_{\text{sample}} \text{ x } M_{\text{std}}} \text{ x } P_{\text{std}}$$

$$= \frac{0.714 \text{ x } 6 \text{ x } 3.9 \text{ mg x } 315.15 \text{ g mol}^{-1}}{6 \text{ x } 1 \text{ x } 9.7 \text{ mg x } 94.13 \text{ g mol}^{-1}} \text{ x } 99.96$$

$$= 96.07\%$$

S = Integrated area of the peak
N = Number of protons represented
m = Prepared mass
M = Molecular weight
P = Purity

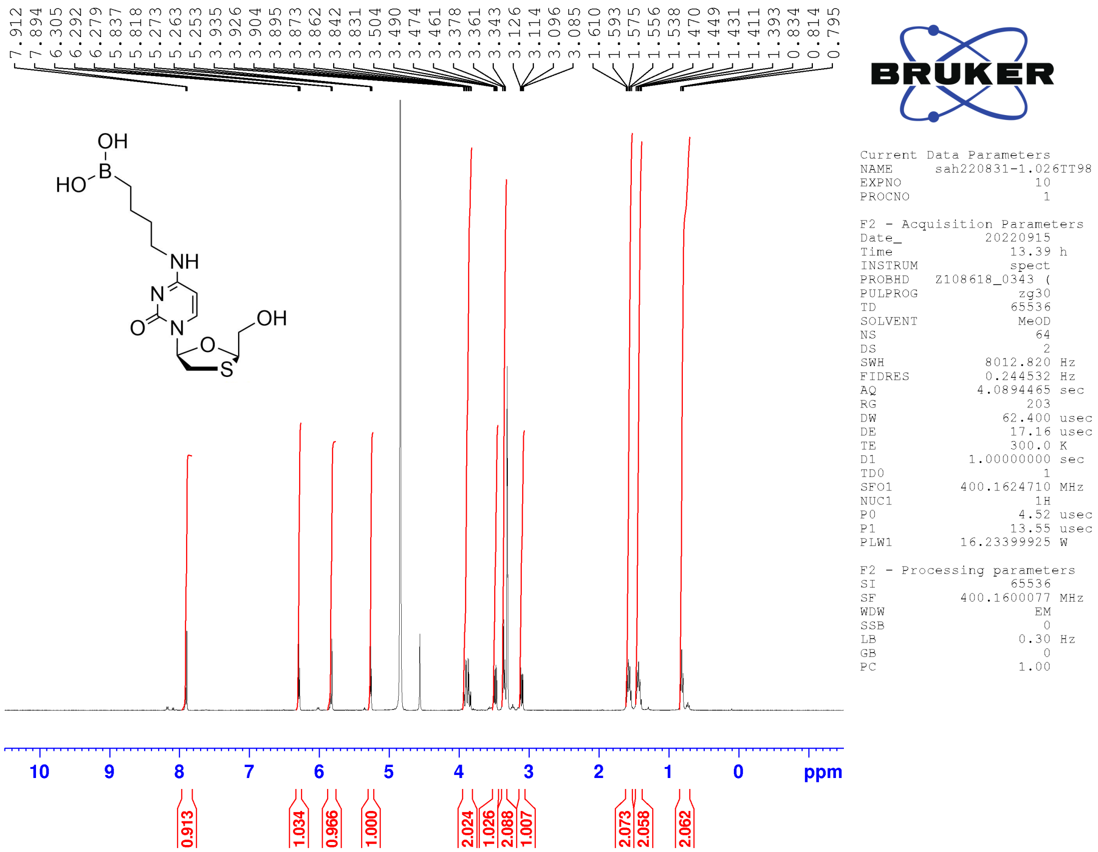

Figure S109 ^{1}HNMR spectra of **N4aL** in CD_3OD.

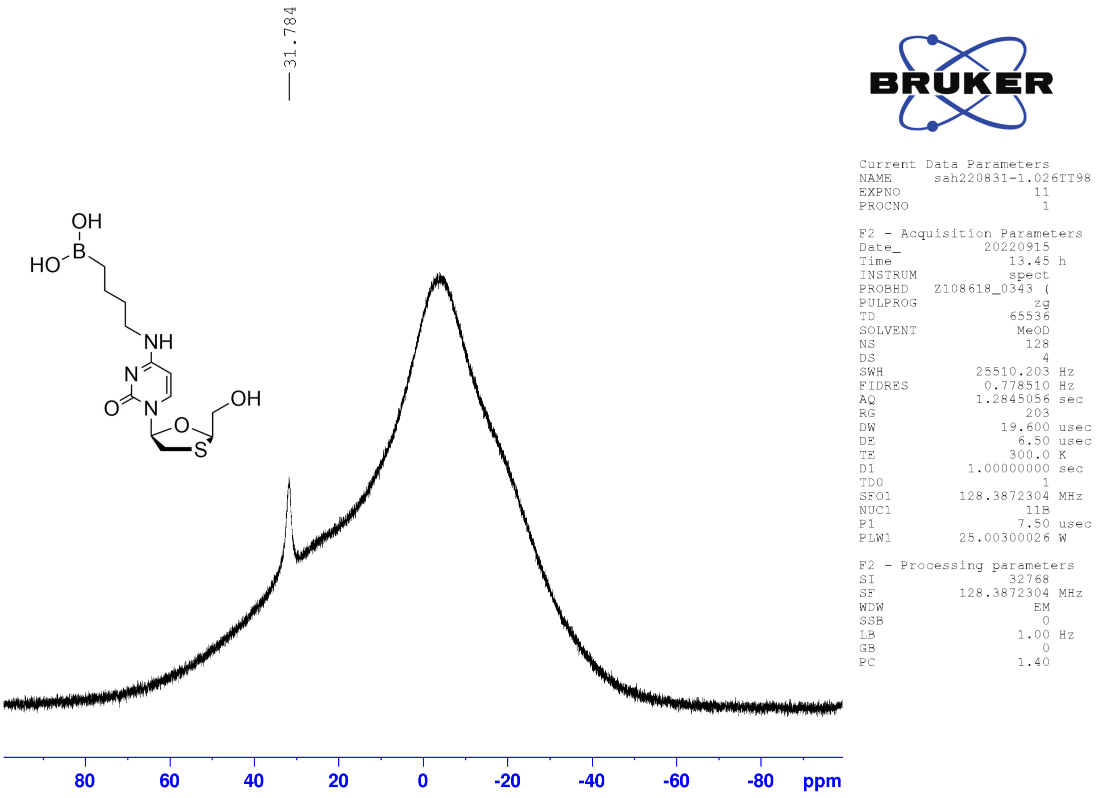

Figure S110 ^{11}BNMR spectra of **N4aL** in CD_3OD.

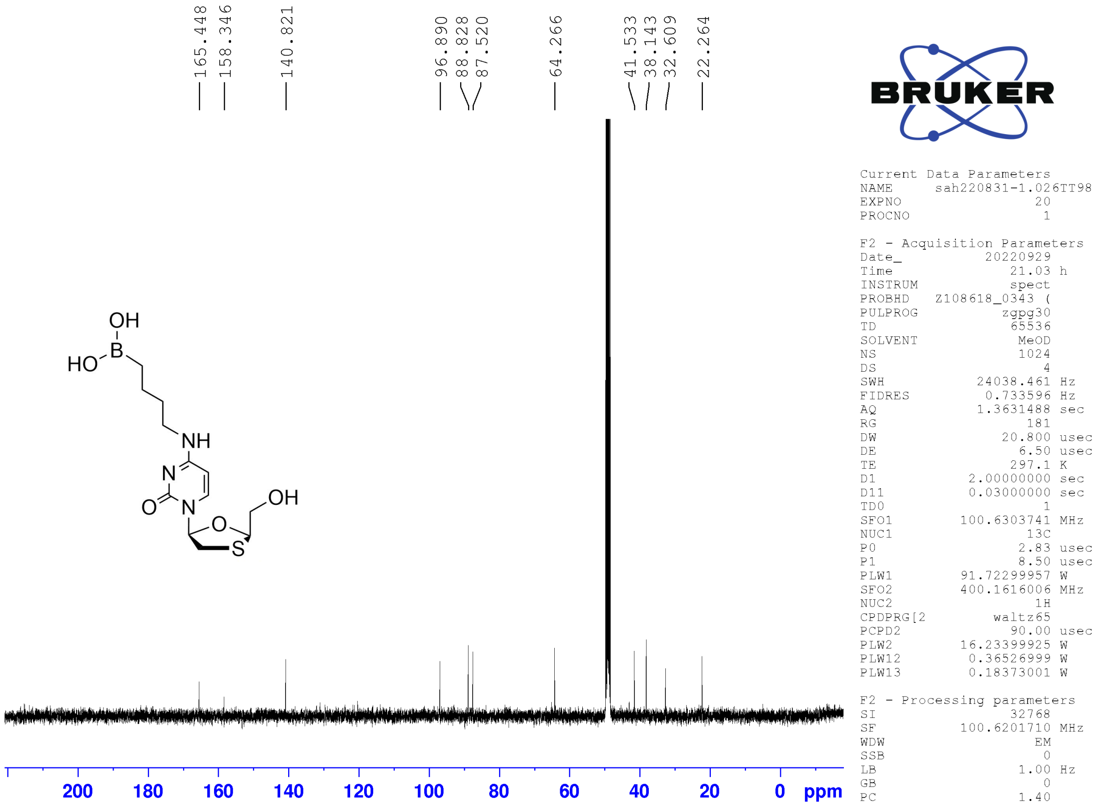

Figure S111 $^{13}C\{^{1}H\}$-NMR spectra of **N4aL** in CD_3OD.

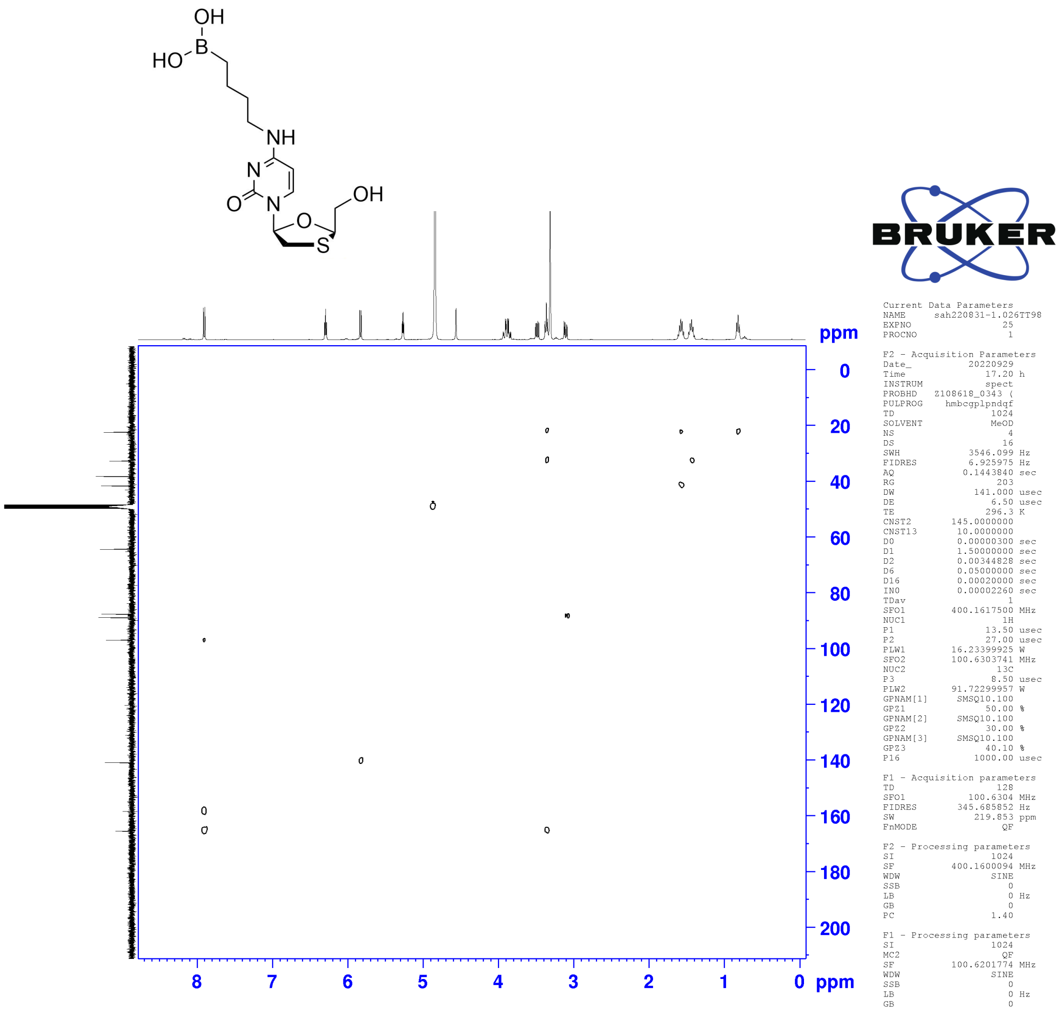

Figure S112 HMBC-NMR spectra of **N4aL** in CD_3OD.

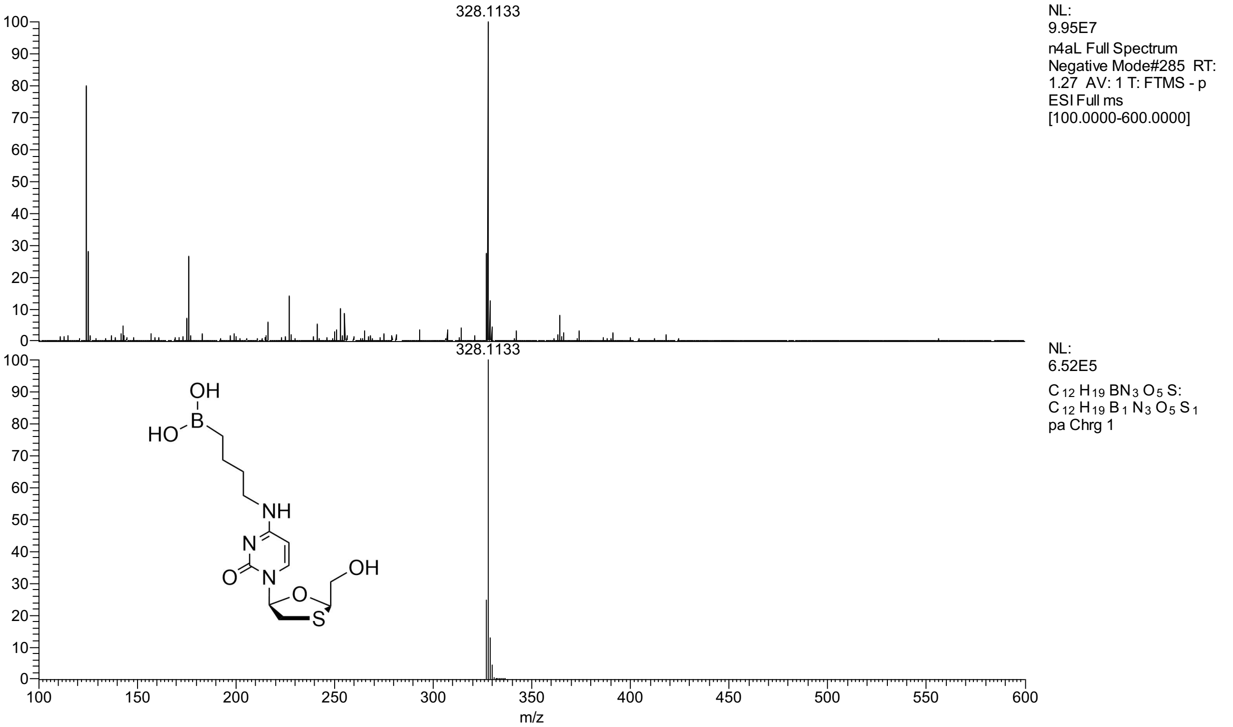

Figure S113 HRMS (ESI-) negative mode m/z calculated for **N4aL** [$C_{12}H_{19}BN_3O_5S$] $[M-H]^-$ 328.1133, found 328.1133.

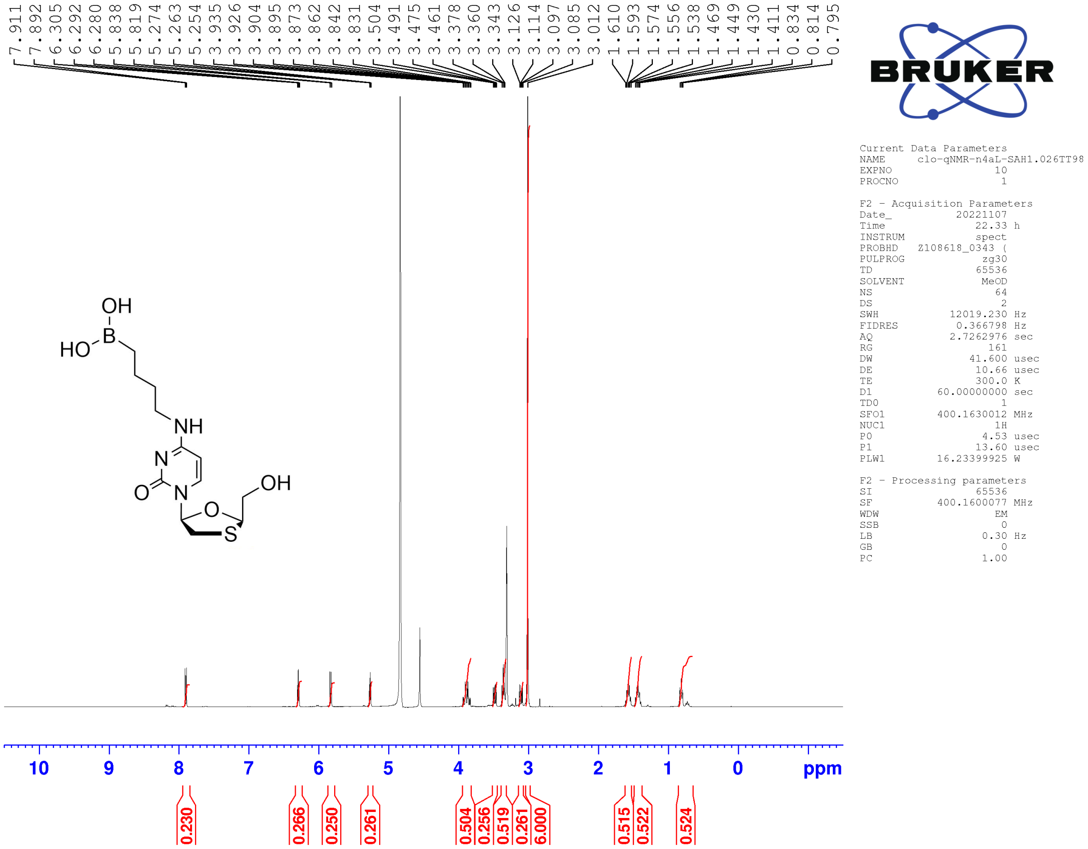

Figure S114 ^{1}H qNMR purity spectrum of **N4aL** with Me_2SO_2 (I.C) in CD_3OD. Purity 95.79%.

$$P_{\text{sample}} = \frac{S_{\text{sample}} \text{ x } N_{\text{std}} \text{ x } m_{\text{std}} \text{ x } M_{\text{sample}}}{S_{\text{std}} \text{ x } N_{\text{sample}} \text{ x } m_{\text{sample}} \text{ x } M_{\text{std}}} \text{ x } P_{\text{std}}$$

$$= \frac{0.266 \text{ x } 6 \text{ x } 3.4 \text{ mg x } 329.12 \text{ g mol}^{-1}}{6 \text{ x } 1 \text{ x } 3.3 \text{ mg x } 94.13 \text{ g mol}^{-1}} \text{ x } 99.96$$

$$= 95.79\%$$

S = Integrated area of the peak
N = Number of protons represented
m = Prepared mass
M = Molecular weight
P = Purity

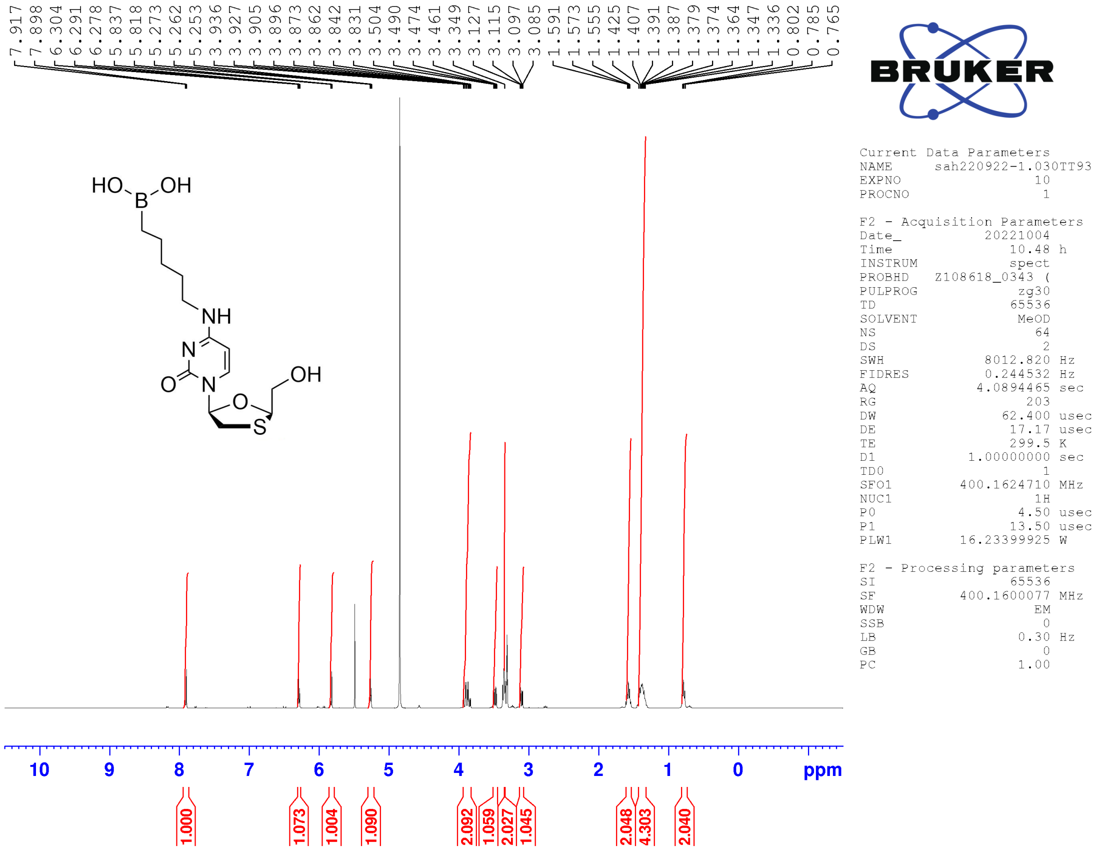

Figure S115 ^{1}HNMR spectra of **N5aL** in CD_3OD.

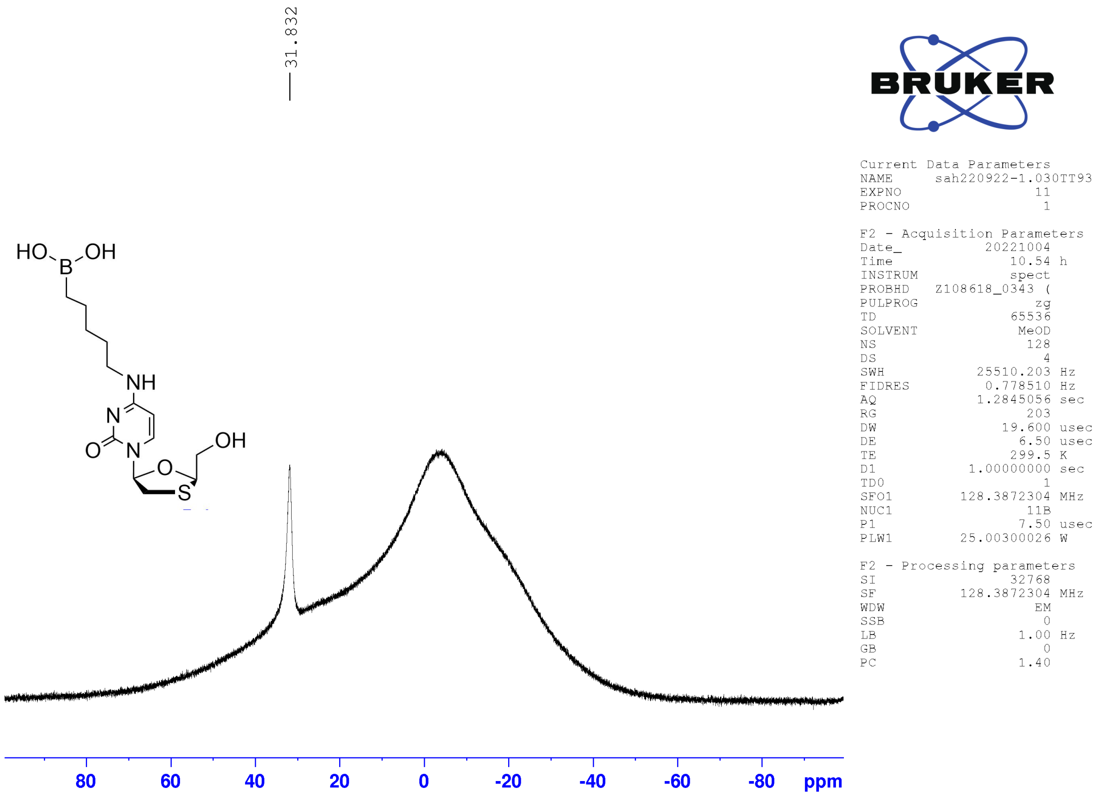

Figure S116 ^{11}BNMR spectra of **N5aL** in CD_3OD.

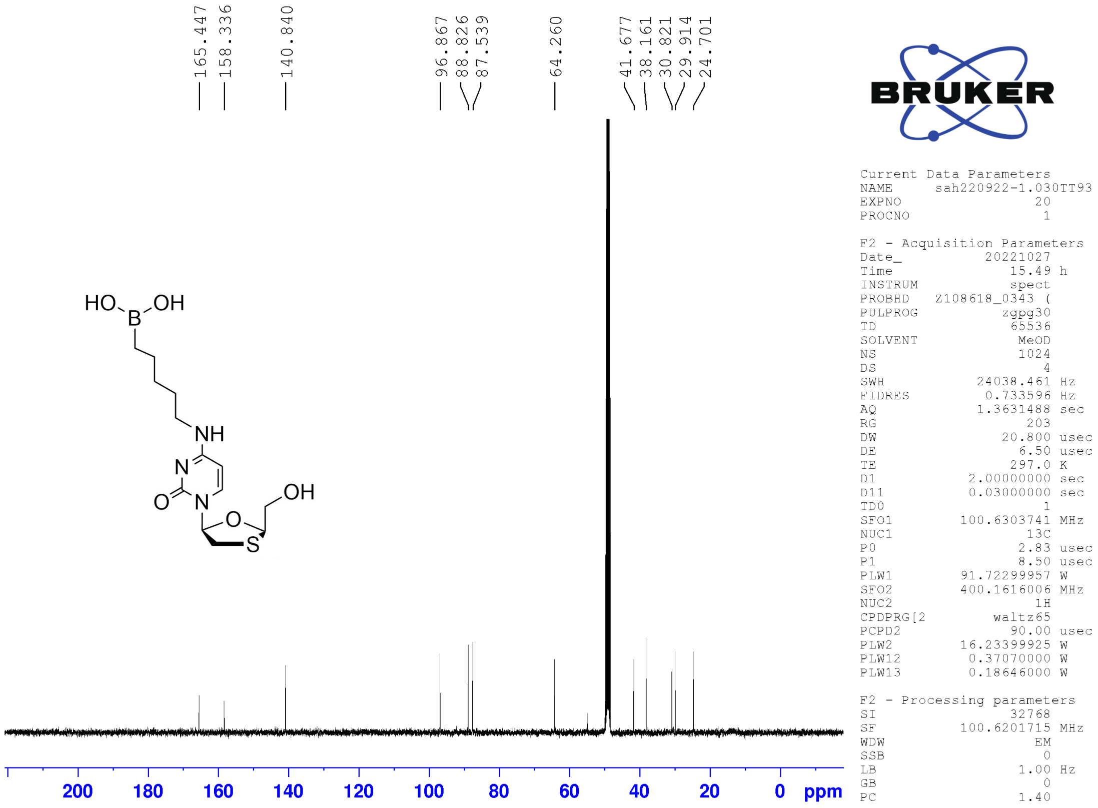

Figure S117 $^{13}C\{^{1}H\}$-NMR spectra of **N5aL** in CD_3OD.

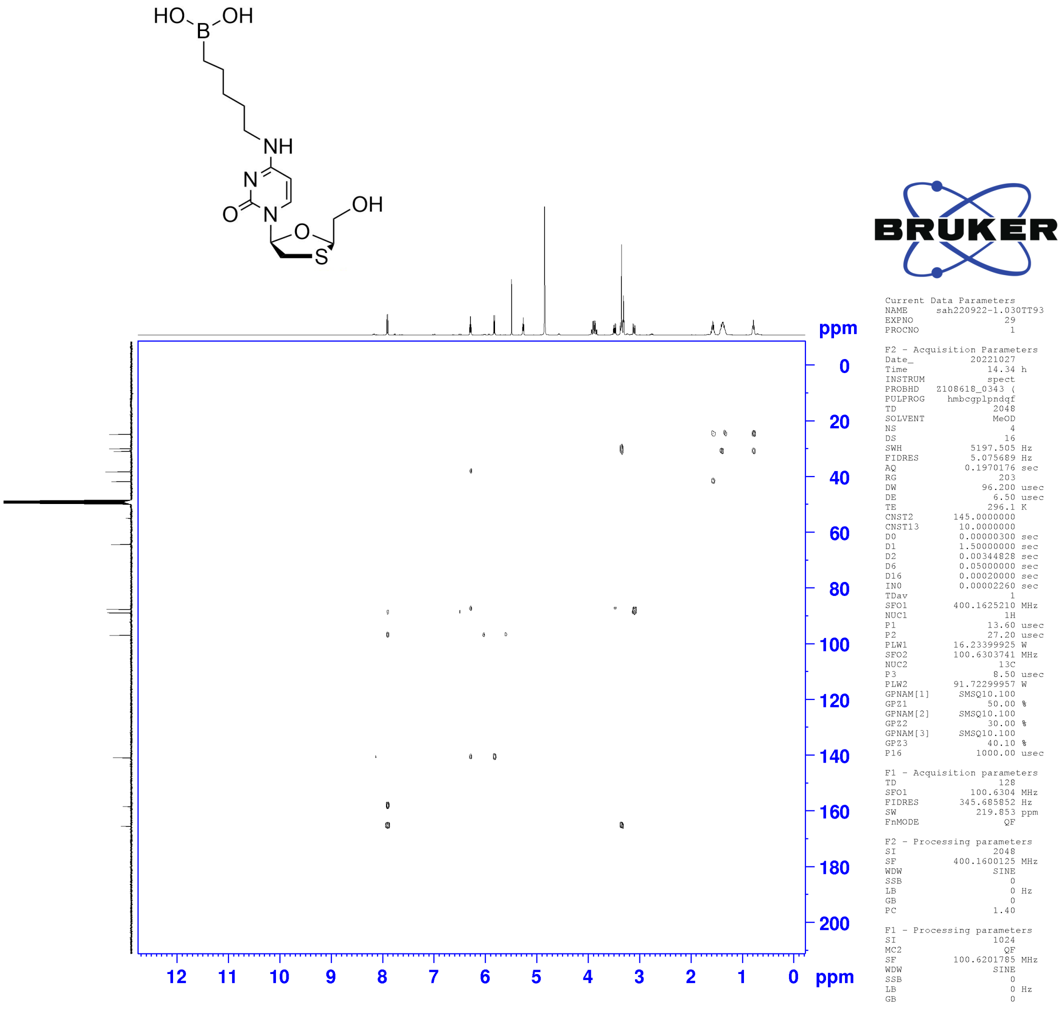

Figure S118 HMBC-NMR spectra of **N5aL** in CD_3OD.

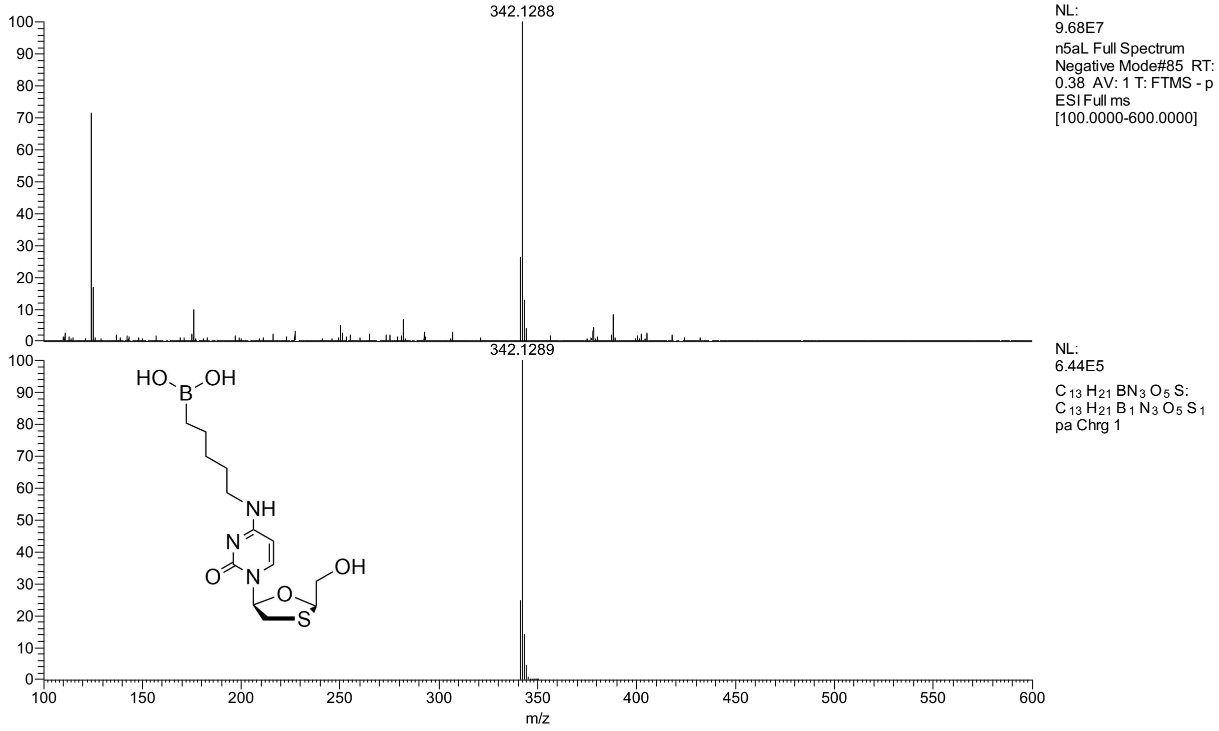

Figure S119 HRMS (ESI-) negative mode m/z calculated for **N5aL** [$C_{13}H_{21}BN_3O_5S$] [M-H]$^-$ 342.1289, found 342.1288.

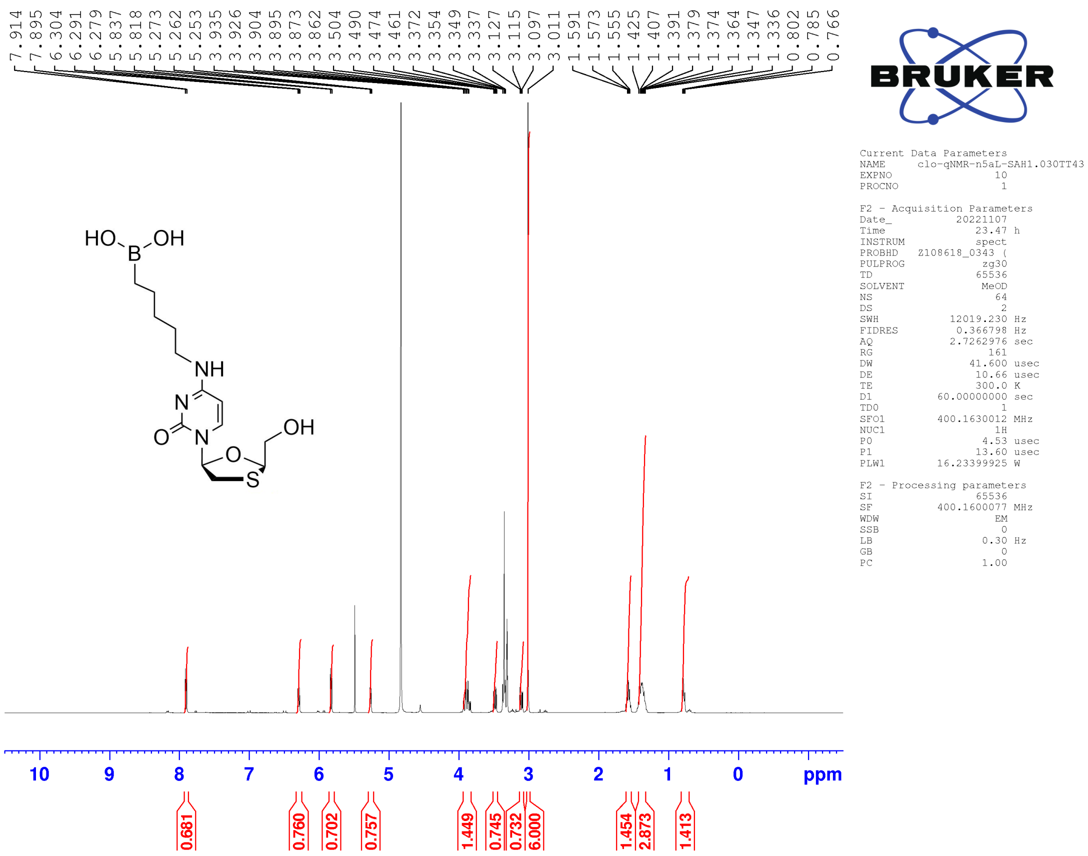

Figure S120 ^{1}H qNMR purity spectrum of **N5aL** with Me_2SO_2 (I.C) in CD_3OD. Purity 95.91%.

$$P_{\text{sample}} = \frac{S_{\text{sample}} \text{ x } N_{\text{std}} \text{ x } m_{\text{std}} \text{ x } M_{\text{sample}}}{S_{\text{std}} \text{ x } N_{\text{sample}} \text{ x } m_{\text{sample}} \text{ x } M_{\text{std}}} \text{ x } P_{\text{std}}$$

$$= \frac{0.702 \text{ x } 6 \text{ x } 3.6 \text{ mg x } 343.14 \text{ g mol}^{-1}}{6 \text{ x } 1 \text{ x } 9.6 \text{ mg x } 94.13 \text{ g mol}^{-1}} \text{ x } 99.96$$

$$= 95.91\%$$

S = Integrated area of the peak
N = Number of protons represented
m = Prepared mass
M = Molecular weight
P = Purity

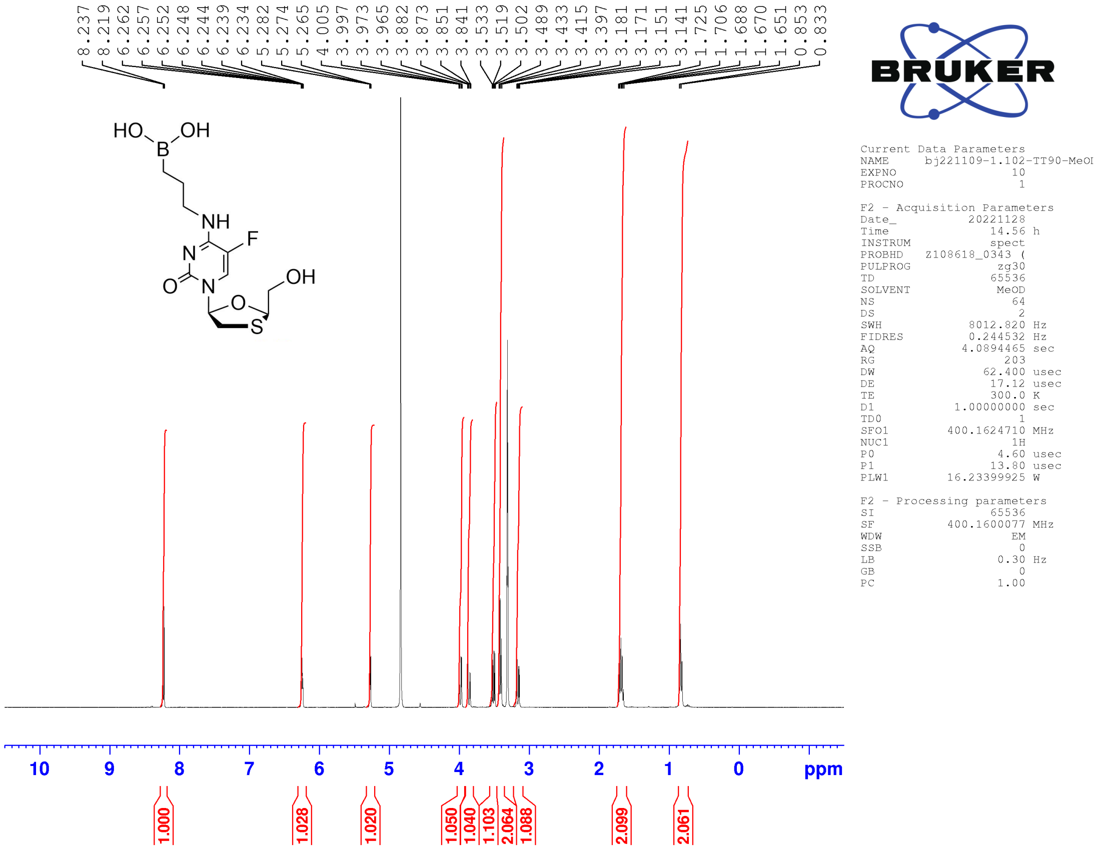

Figure S121 ^{1}HNMR spectra of **N3aE** in CD_3OD.

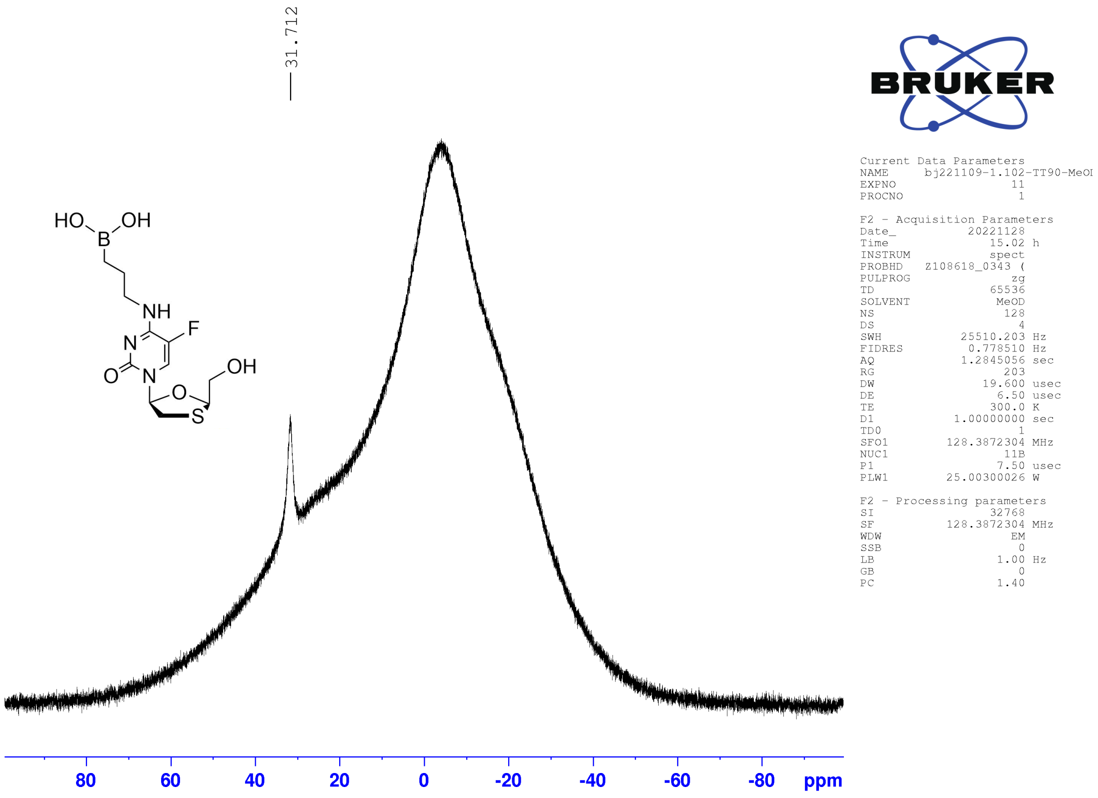

Figure S122 ^{11}BNMR spectra of **N3aE** in CD_3OD.

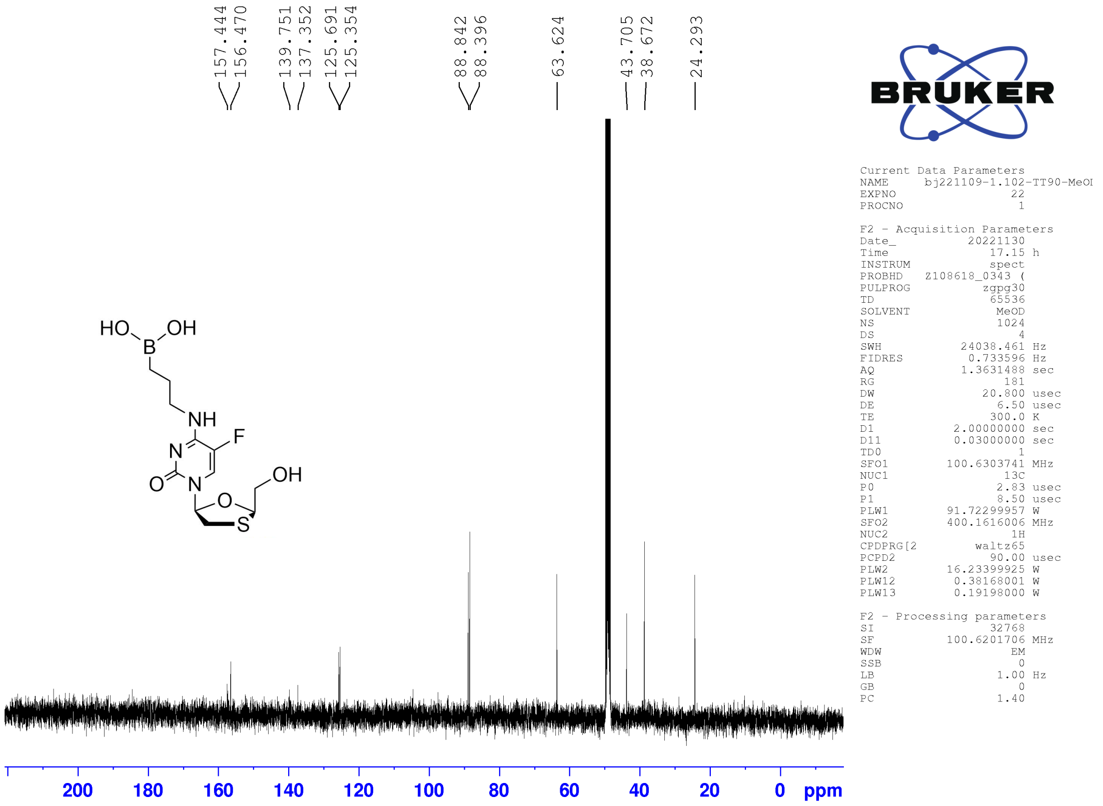

Figure S123 $^{13}C\{^{1}H\}$-NMR spectra of **N3aE** in CD_3OD.

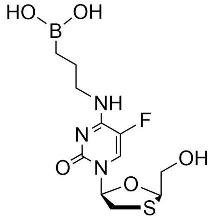

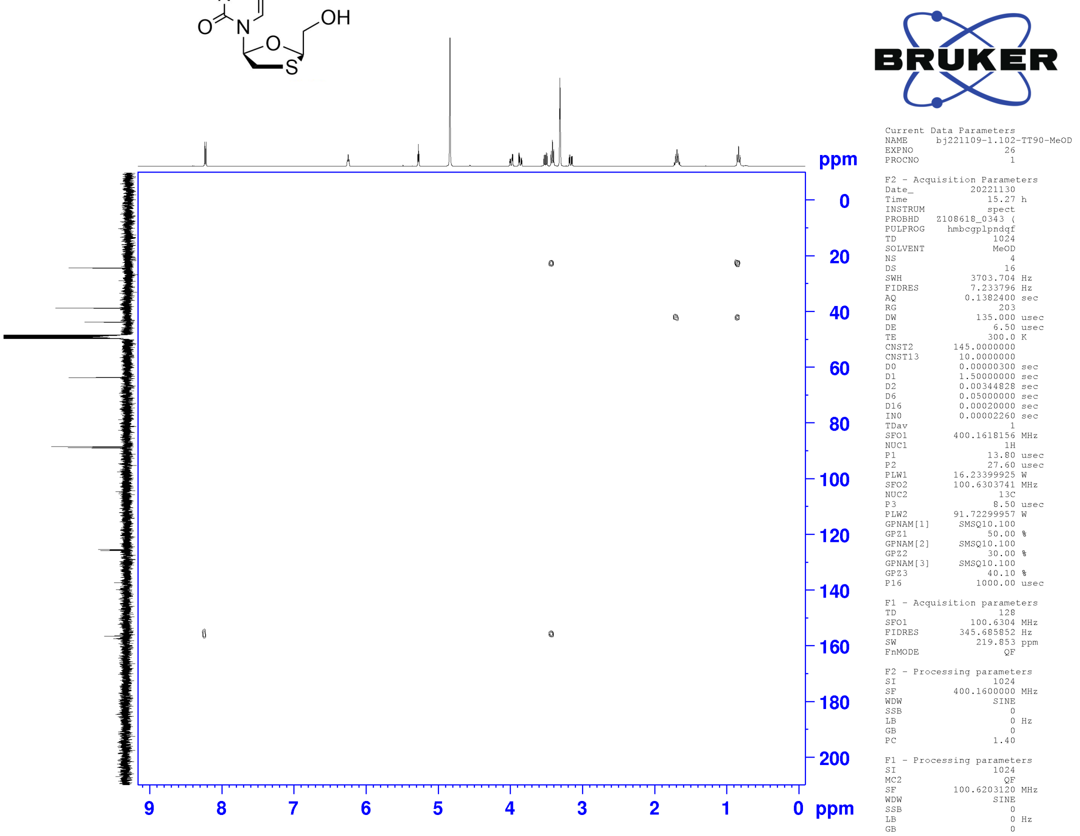

Figure S124 HMBC-NMR spectra of **N3aE** in CD_3OD.

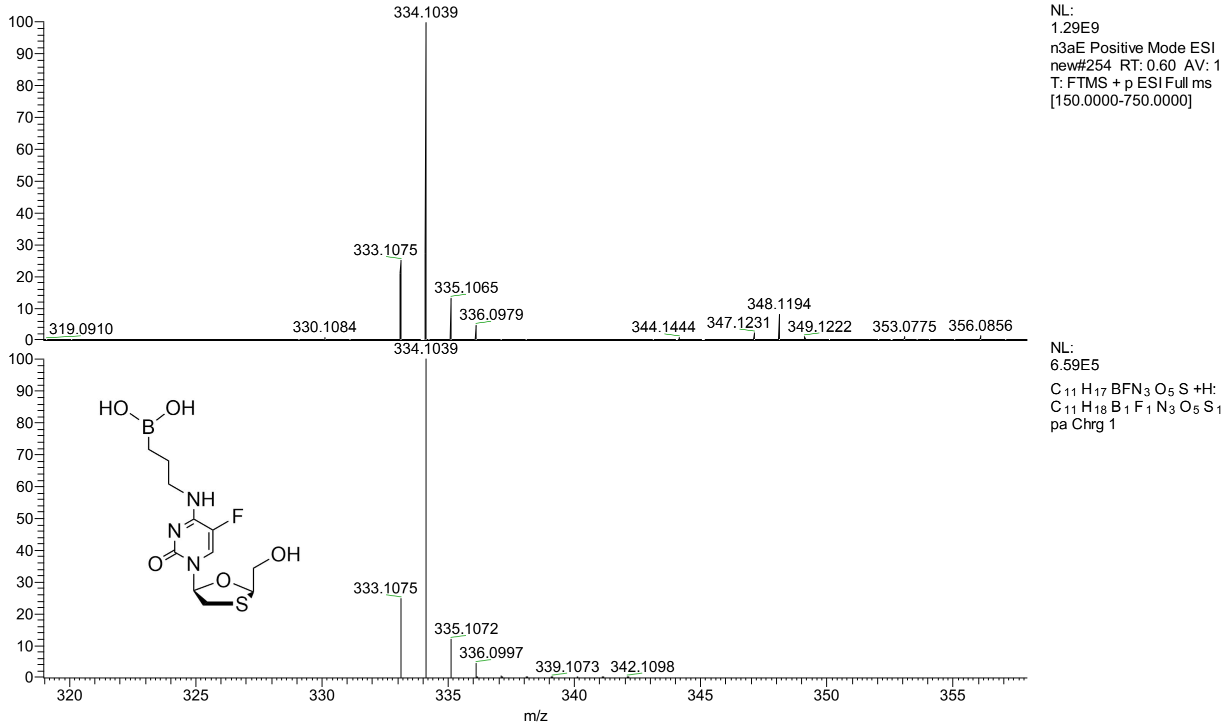

Figure S125 HRMS (ESI+) positive mode m/z calculated for **N3aE** [$C_{11}H_{18}BFN_3O_5S$] $[M+H]^+$ 334.1039, found 334.1039.

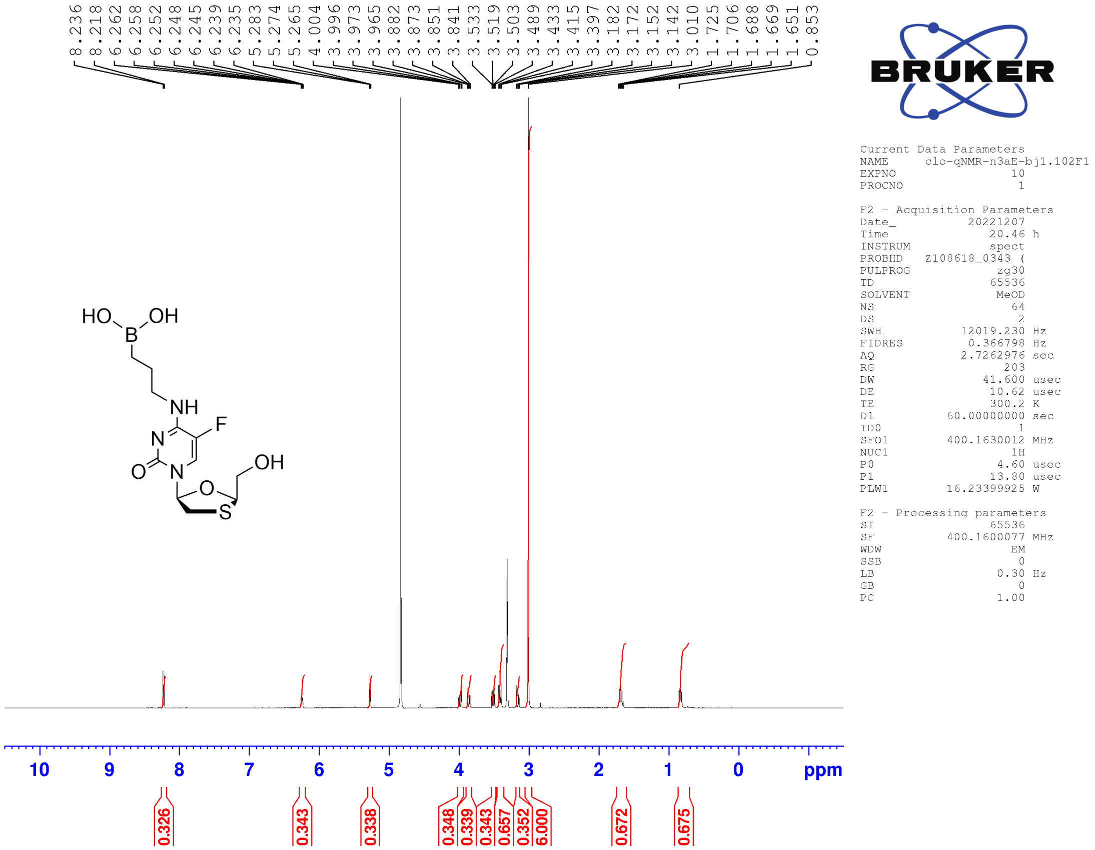

Figure S126 ^{1}H qNMR purity spectrum of **N3aE** with Me_2SO_2 (I.C) in CD_3OD. Purity 97.15%.

$$P_{\text{sample}} = \frac{S_{\text{sample}} \text{ x } N_{\text{std}} \text{ x } m_{\text{std}} \text{ x } M_{\text{sample}}}{S_{\text{std}} \text{ x } N_{\text{sample}} \text{ x } m_{\text{sample}} \text{ x } M_{\text{std}}} \text{ x } P_{\text{std}}$$

$$= \frac{0.338 \text{ x } 6 \text{ x } 2.6 \text{ mg x } 333.14 \text{ g mol}^{-1}}{6 \text{ x } 1 \text{ x } 3.2 \text{ mg x } 94.13 \text{ g mol}^{-1}} \text{ x } 99.96$$

$$= 97.15\%$$

S = Integrated area of the peak
N = Number of protons represented
m = Prepared mass
M = Molecular weight
P = Purity

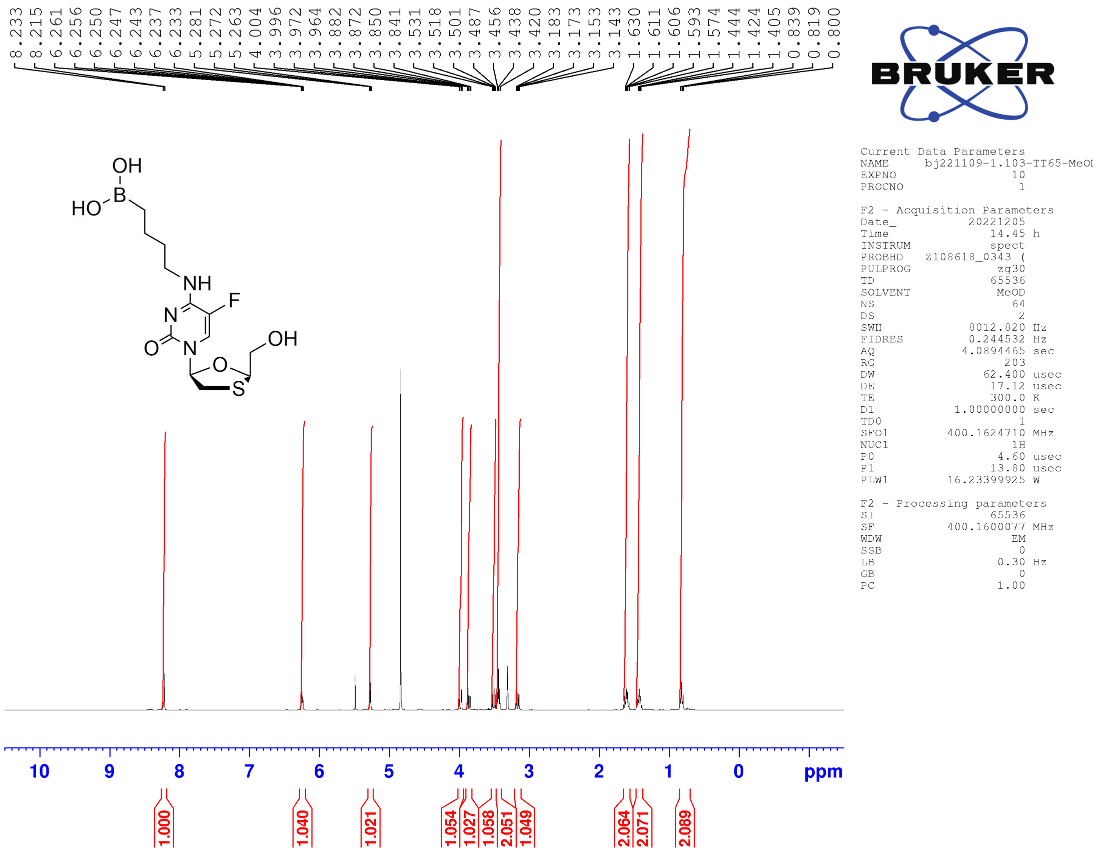

Figure S127 ^{1}HNMR spectra of **N4aE** in CD_3OD.

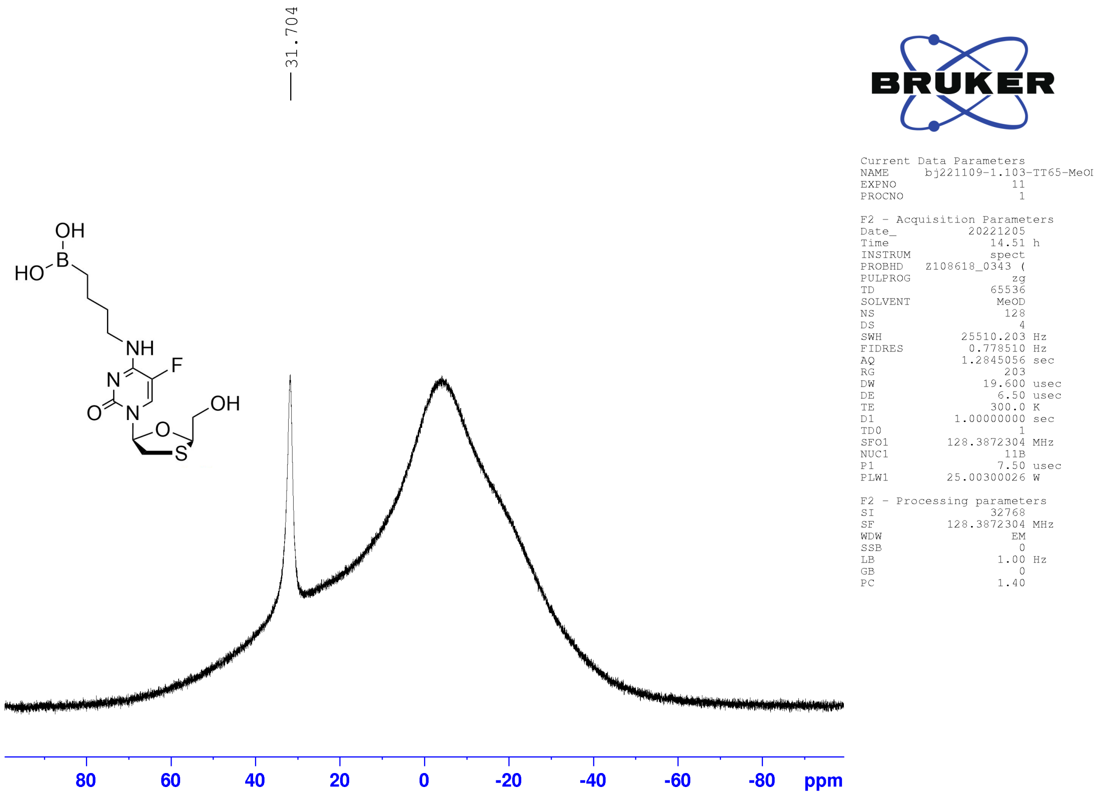

Figure S128 ^{11}BNMR spectra of **N4aE** in CD_3OD.

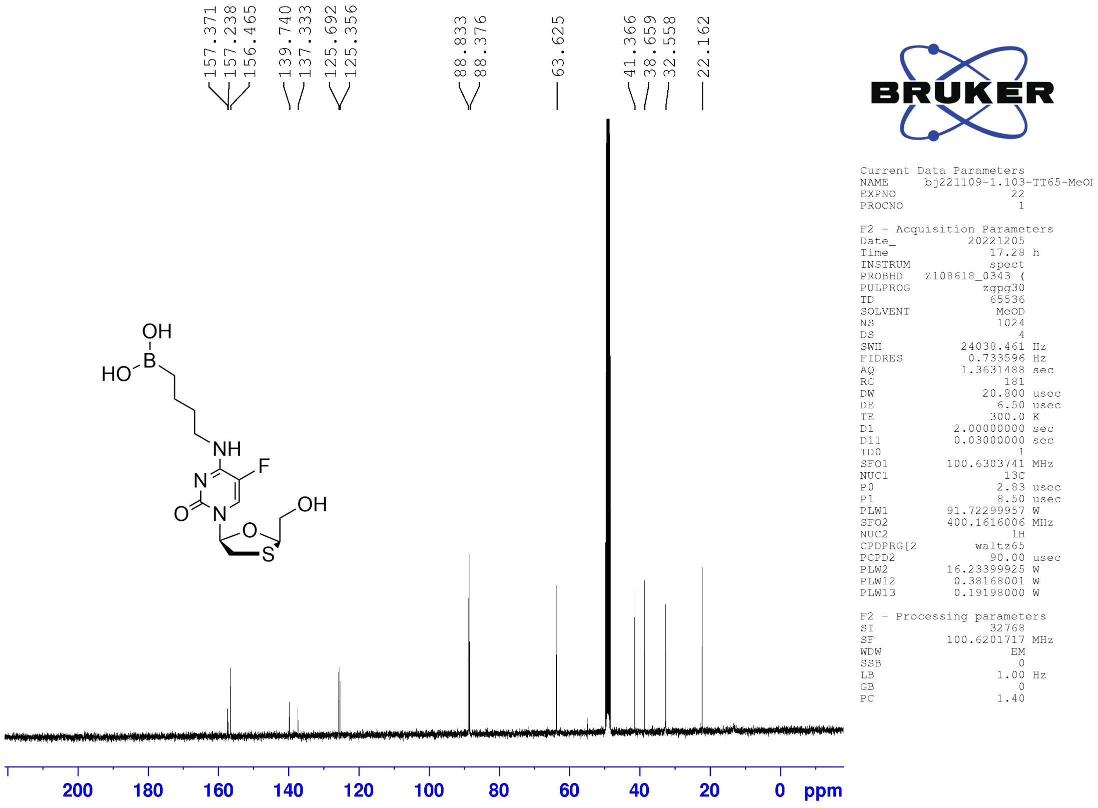

Figure S129 $^{13}C\{^1H\}$-NMR spectra of **N4aE** in CD_3OD.

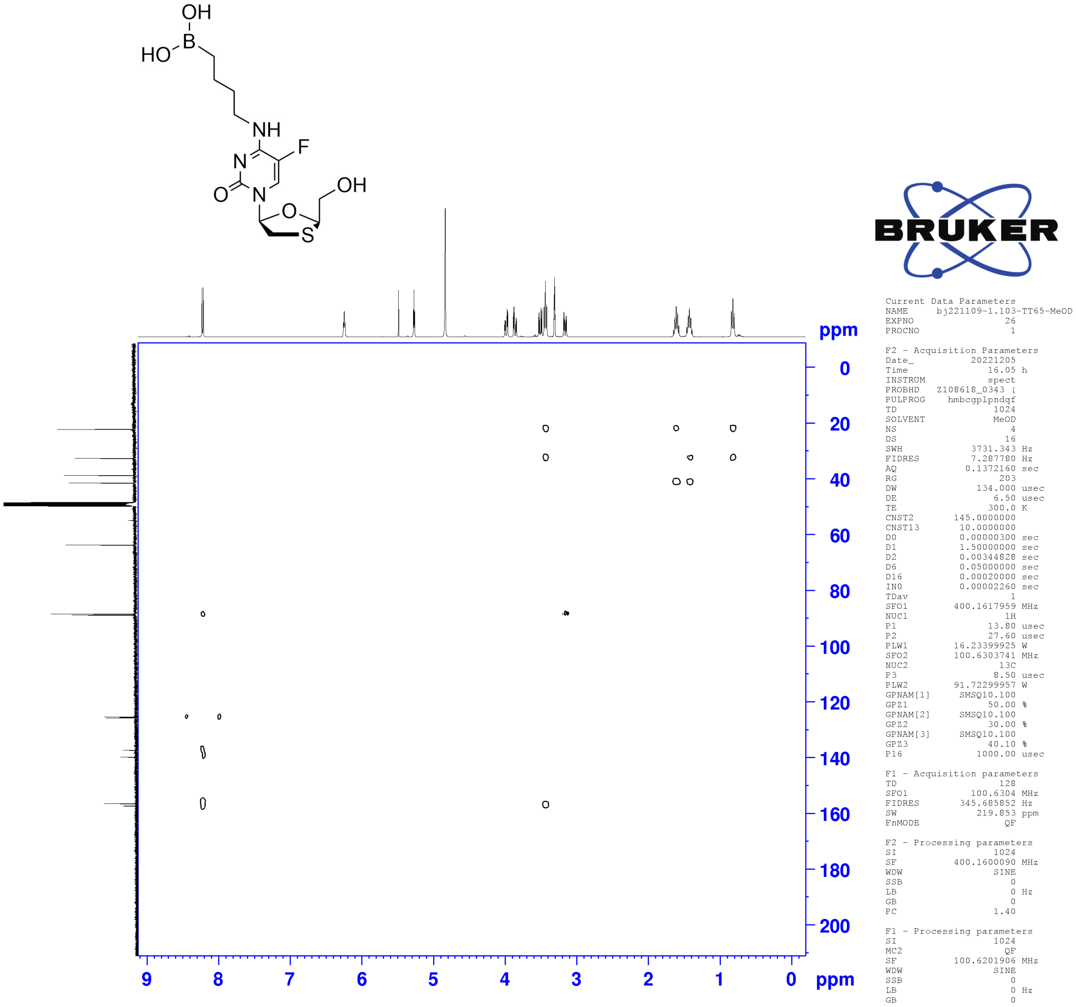

Figure S130 HMBC-NMR spectra of **N4aE** in CD_3OD.

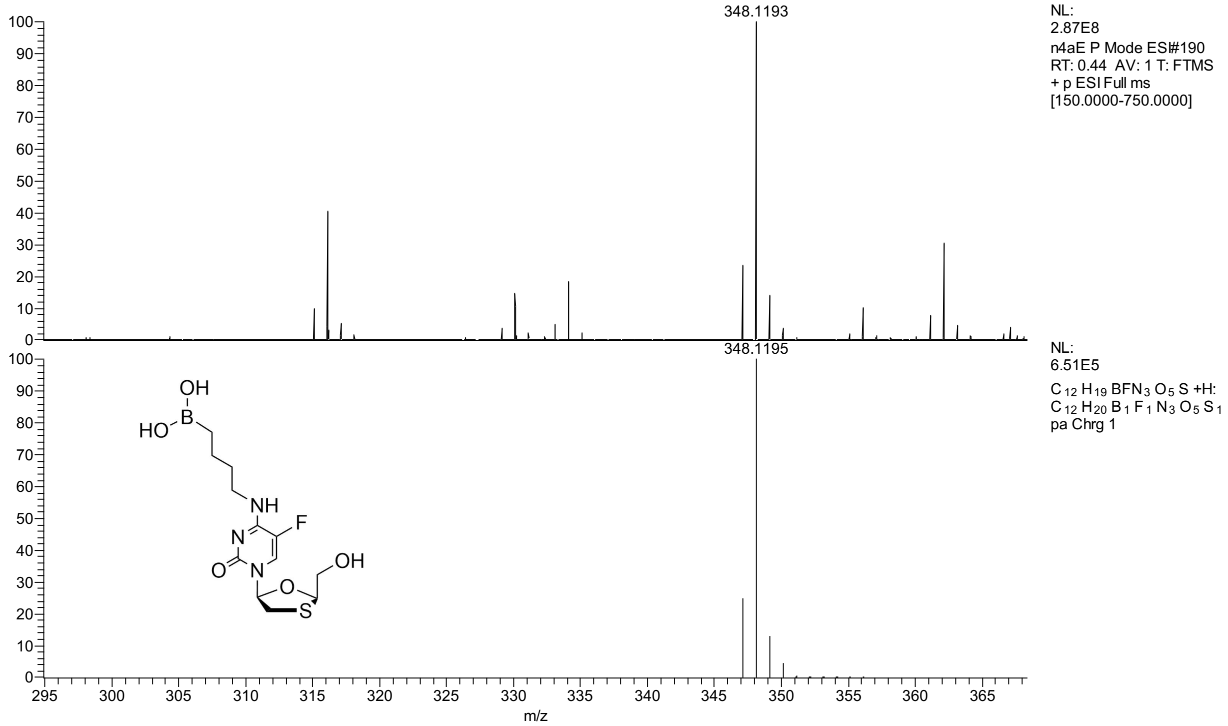

Figure S131 HRMS (ESI+) positive mode m/z calculated for **N4aE** [$C_{12}H_{20}BFN_3O_5S$] $[M+H]^+$ 348.1195, found 348.1193.

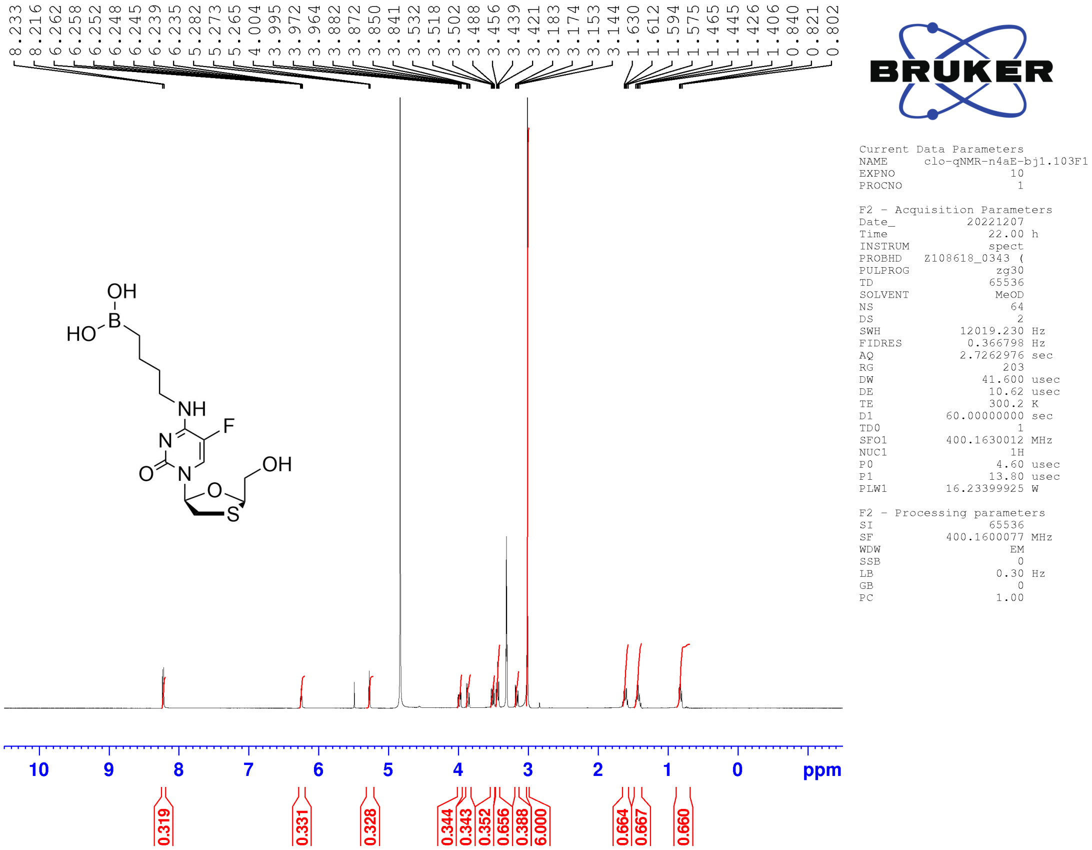

Figure S132 ^{1}H qNMR purity spectrum of **N4aE** with Me_2SO_2 (I.C) in CD_3OD. Purity 97.43%.

$$P_{\text{sample}} = \frac{S_{\text{sample}} \text{ x } N_{\text{std}} \text{ x } m_{\text{std}} \text{ x } M_{\text{sample}}}{S_{\text{std}} \text{ x } N_{\text{sample}} \text{ x } m_{\text{sample}} \text{ x } M_{\text{std}}} \text{ x } P_{\text{std}}$$

$$= \frac{0.328 \text{ x } 6 \text{ x } 2.5 \text{ mg x } 347.17 \text{ g mol}^{-1}}{6 \text{ x } 1 \text{ x } 3.1 \text{ mg x } 94.13 \text{ g mol}^{-1}} \text{ x } 99.96$$

$$= 97.43\%$$

S = Integrated area of the peak
N = Number of protons represented
m = Prepared mass
M = Molecular weight
P = Purity

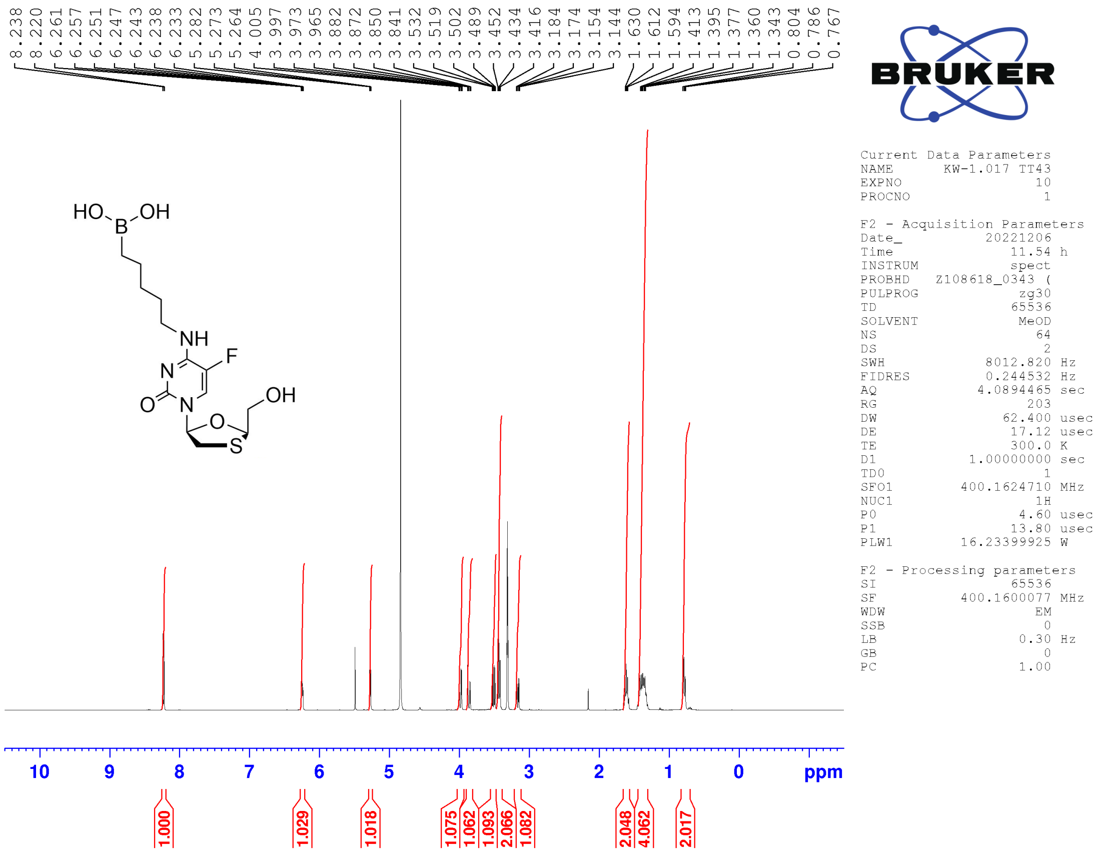

Figure S133 ^{1}HNMR spectra of **N5aE** in CD_3OD.

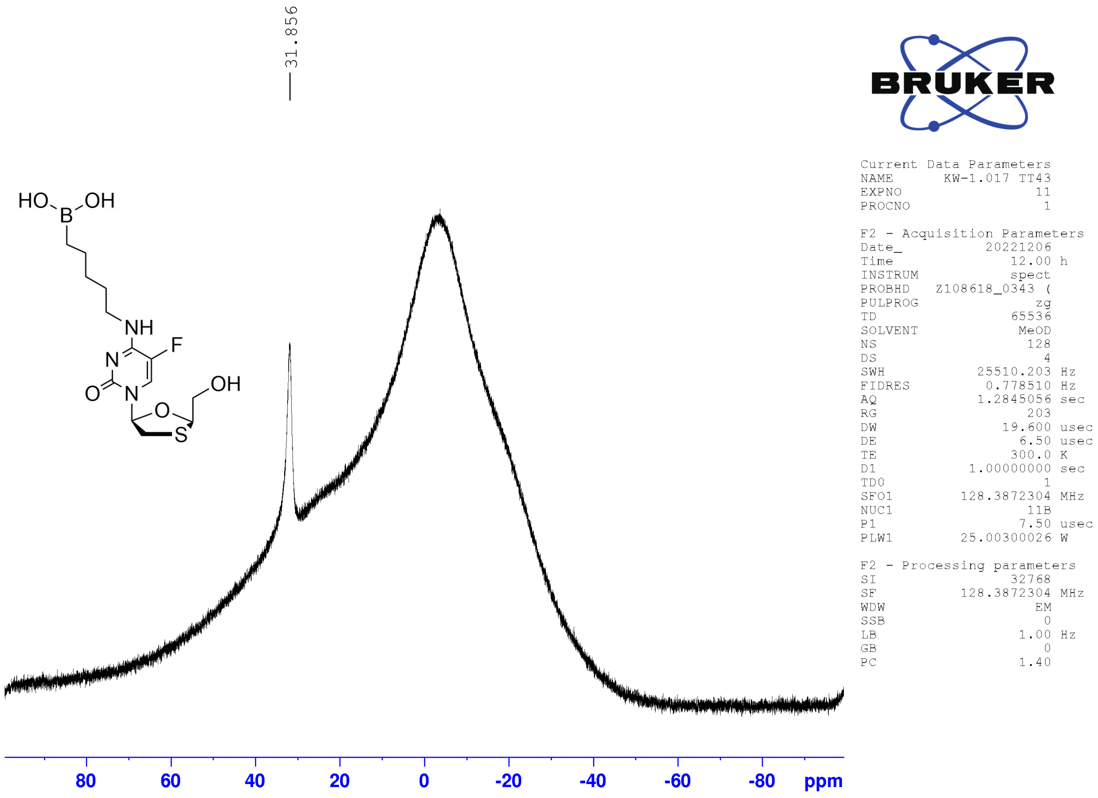

Figure S134 ^{11}BNMR spectra of **N5aE** in CD_3OD.

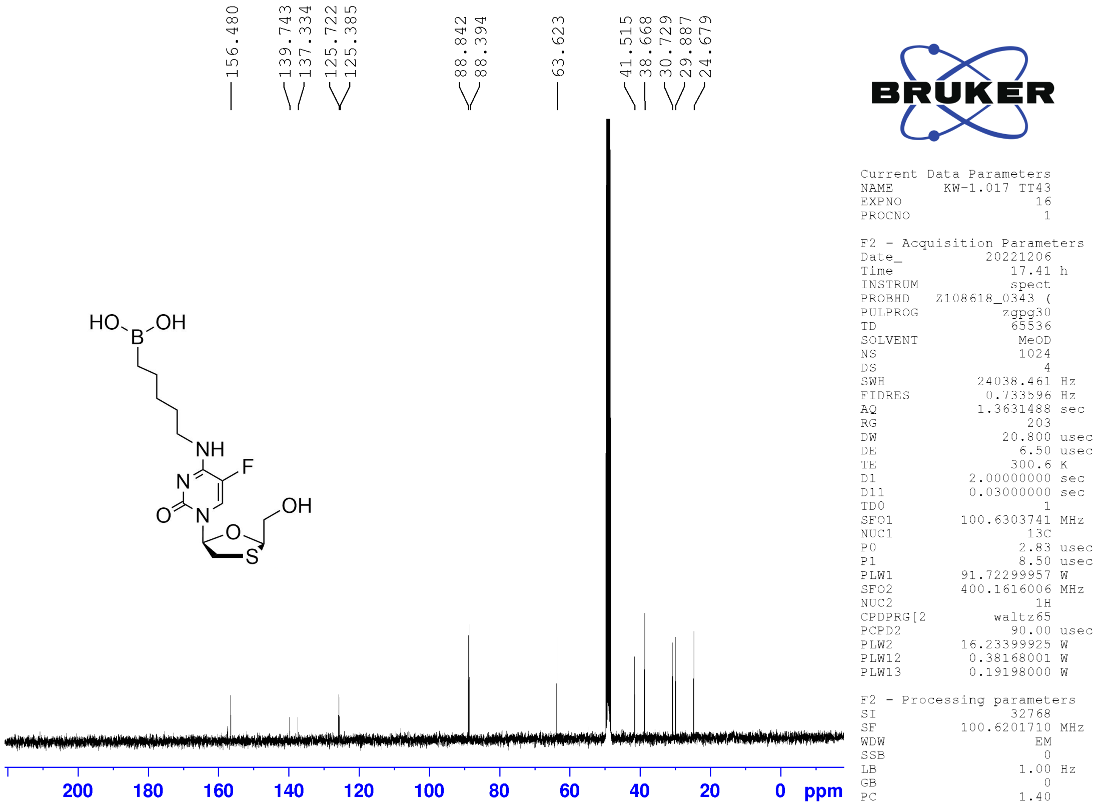

Figure S135 $^{13}C\{^1H\}$-NMR spectra of **N5aE** in CD_3OD.

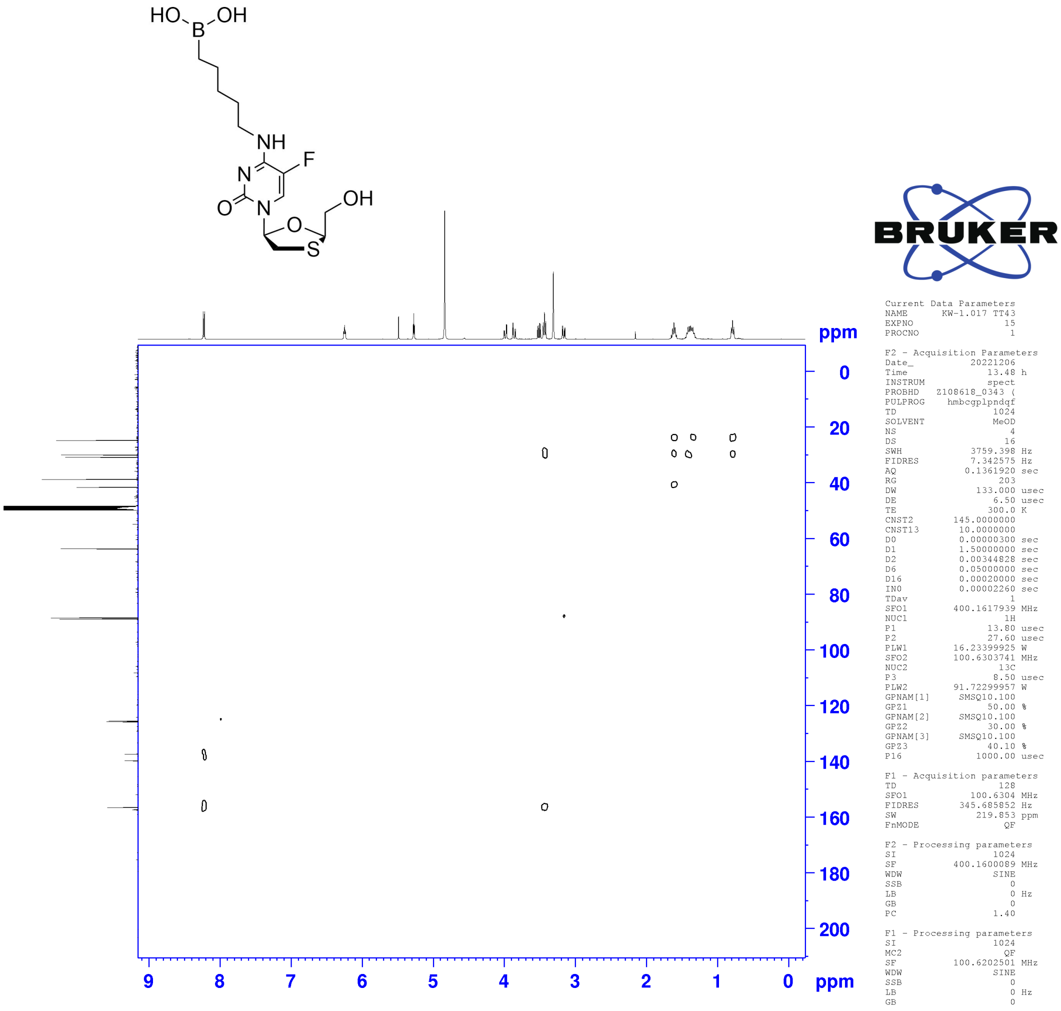

Figure S136 HMBC-NMR spectra of **N5aE** in CD_3OD.

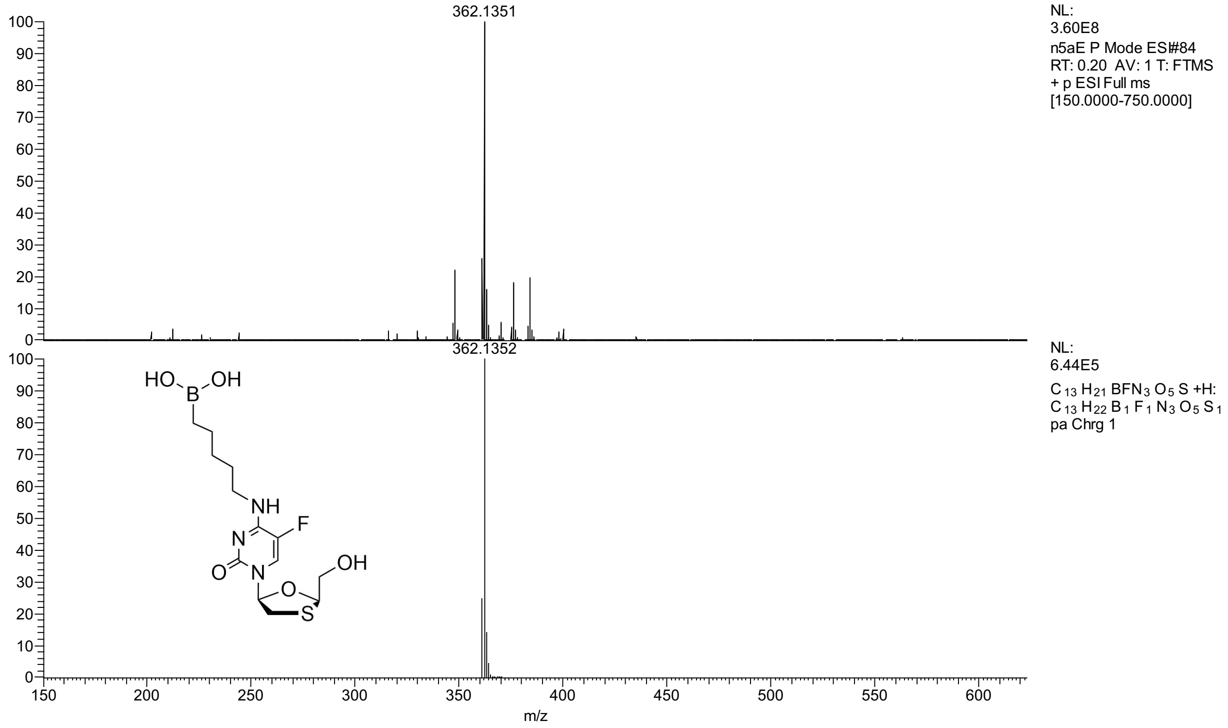

Figure S137 HRMS (ESI+) positive mode m/z calculated for **N5aE** [$C_{13}H_{22}BFN_3O_5S$] $[M+H]^+$ 362.1352, found 362.1351.

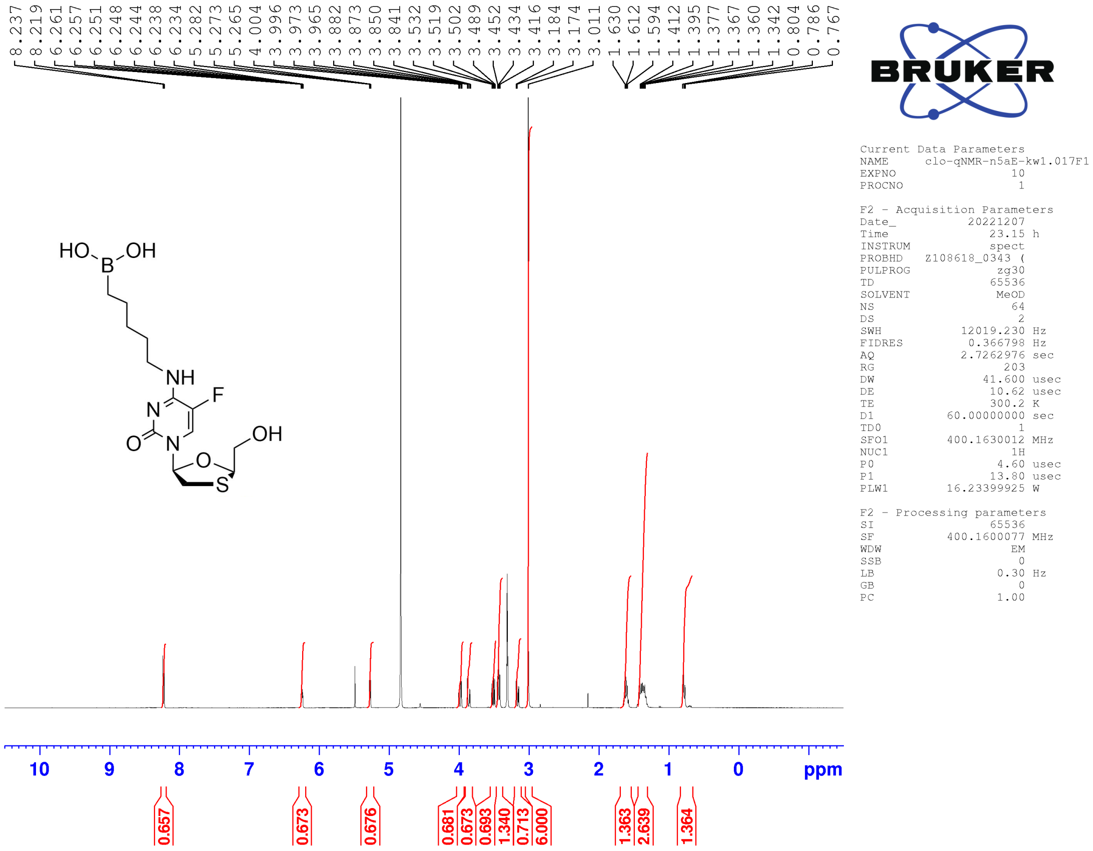

Figure S138 ^{1}H qNMR purity spectrum of **N5aE** with Me_2SO_2 (I.C) in CD_3OD. Purity 97.75%.

$$P_{\text{sample}} = \frac{S_{\text{sample}} \text{ x } N_{\text{std}} \text{ x } m_{\text{std}} \text{ x } M_{\text{sample}}}{S_{\text{std}} \text{ x } N_{\text{sample}} \text{ x } m_{\text{sample}} \text{ x } M_{\text{std}}} \text{ x } P_{\text{std}}$$

$$= \frac{0.676 \text{ x } 6 \text{ x } 2.3 \text{ mg x } 361.20 \text{ g mol}^{-1}}{6 \text{ x } 1 \text{ x } 6.1 \text{ mg x } 94.13 \text{ g mol}^{-1}} \text{ x } 99.96$$

$$= 97.75\%$$

S = Integrated area of the peak
N = Number of protons represented
m = Prepared mass
M = Molecular weight
P = Purity

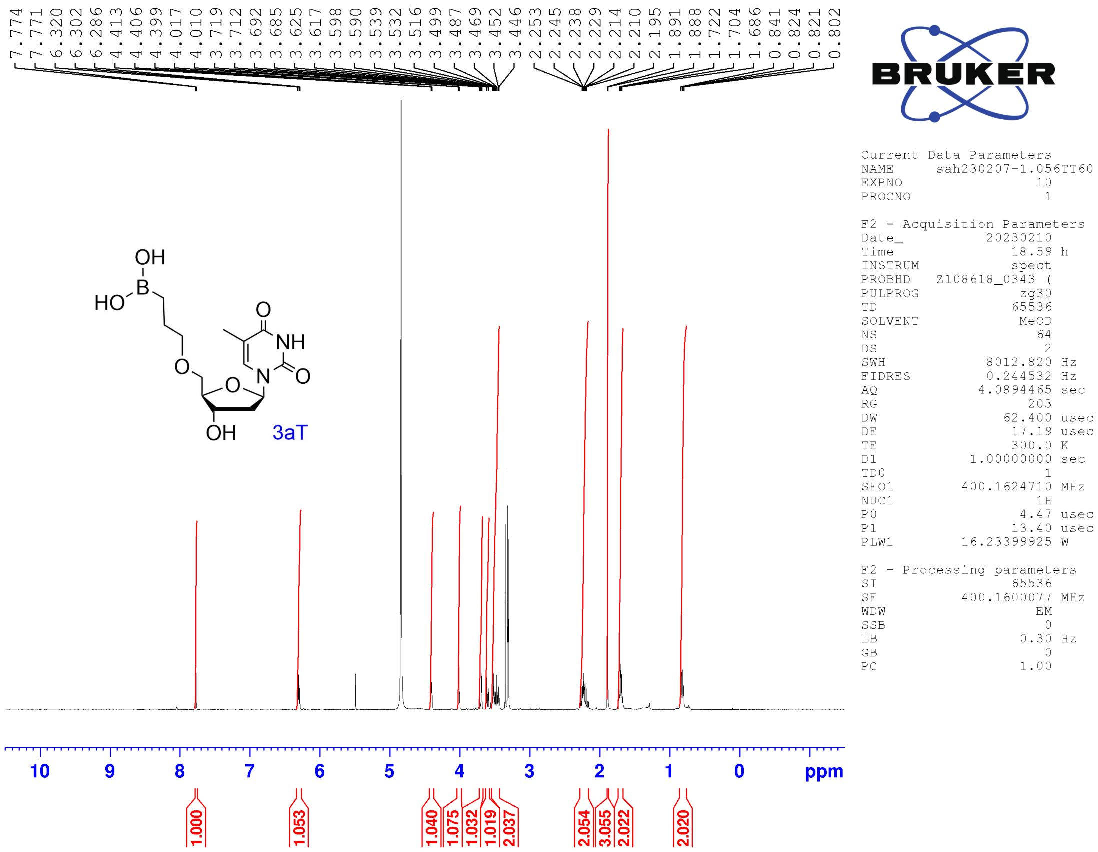

Figure S139 ^{1}HNMR spectra of **3aT** in CD_3OD.

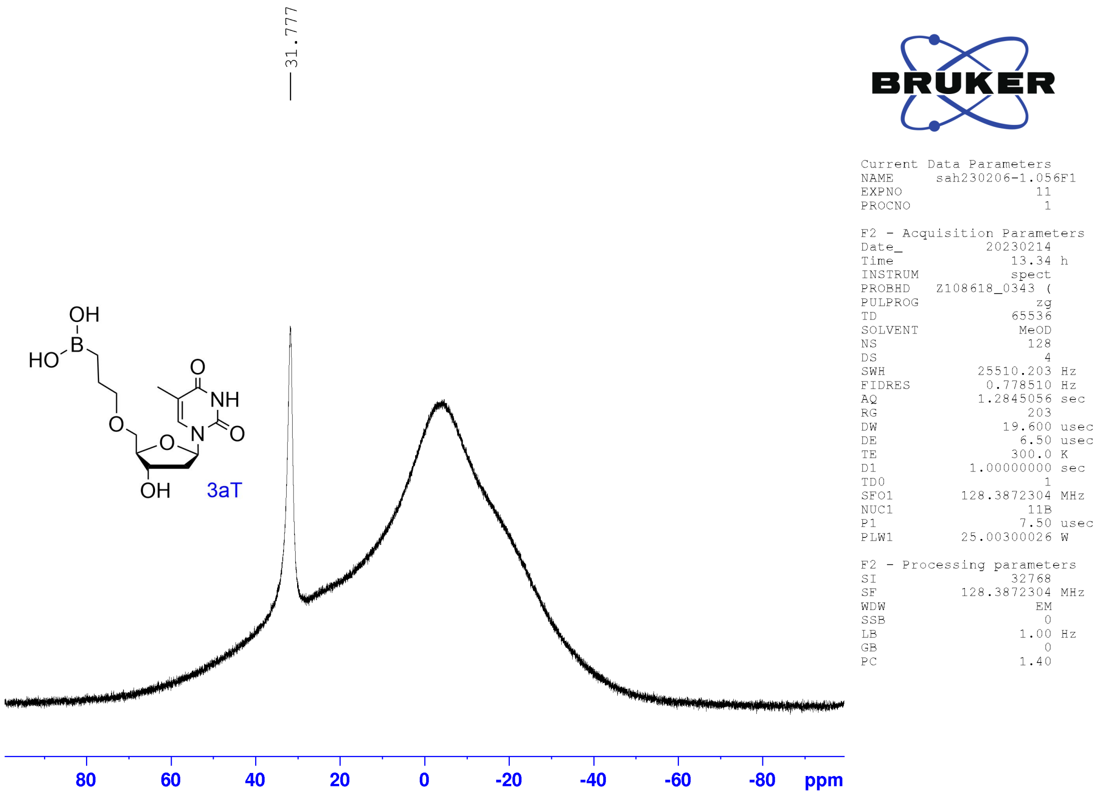

Figure S140 ^{11}BNMR spectra of **3aT** in CD_3OD.

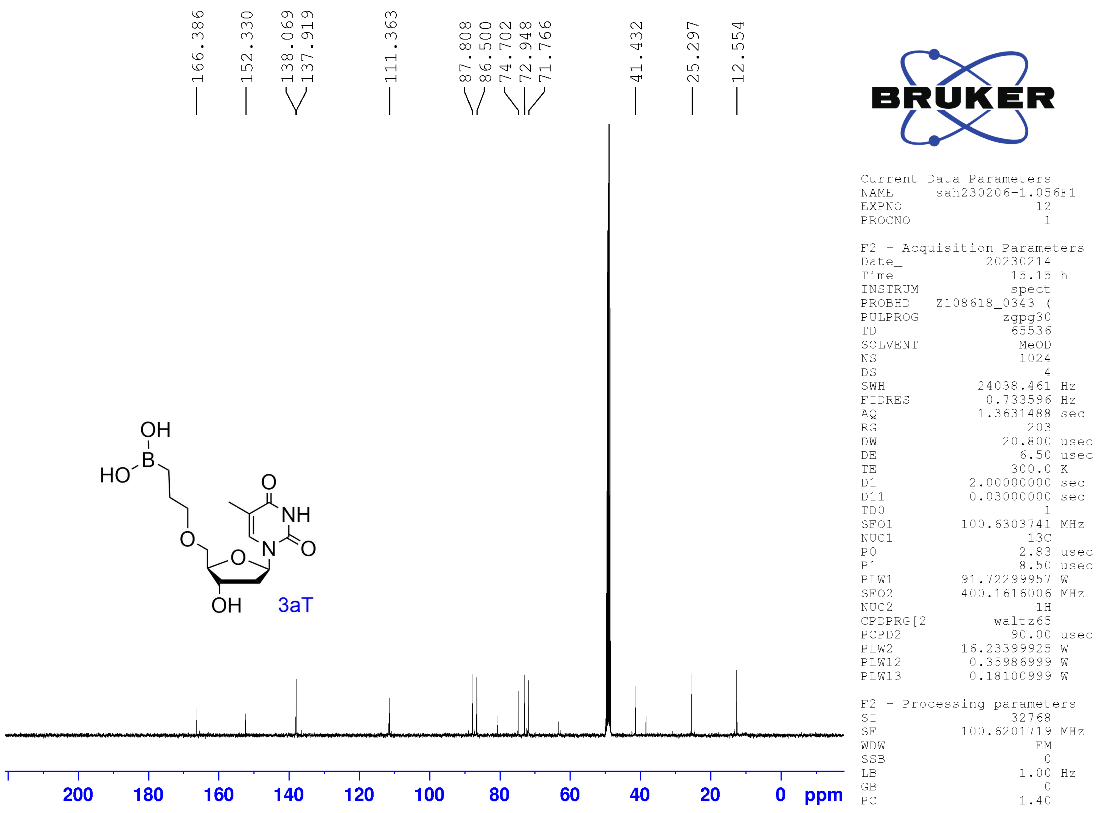

Figure S141 $^{13}C\{^{1}H\}$-NMR spectra of **3aT** in CD_3OD.

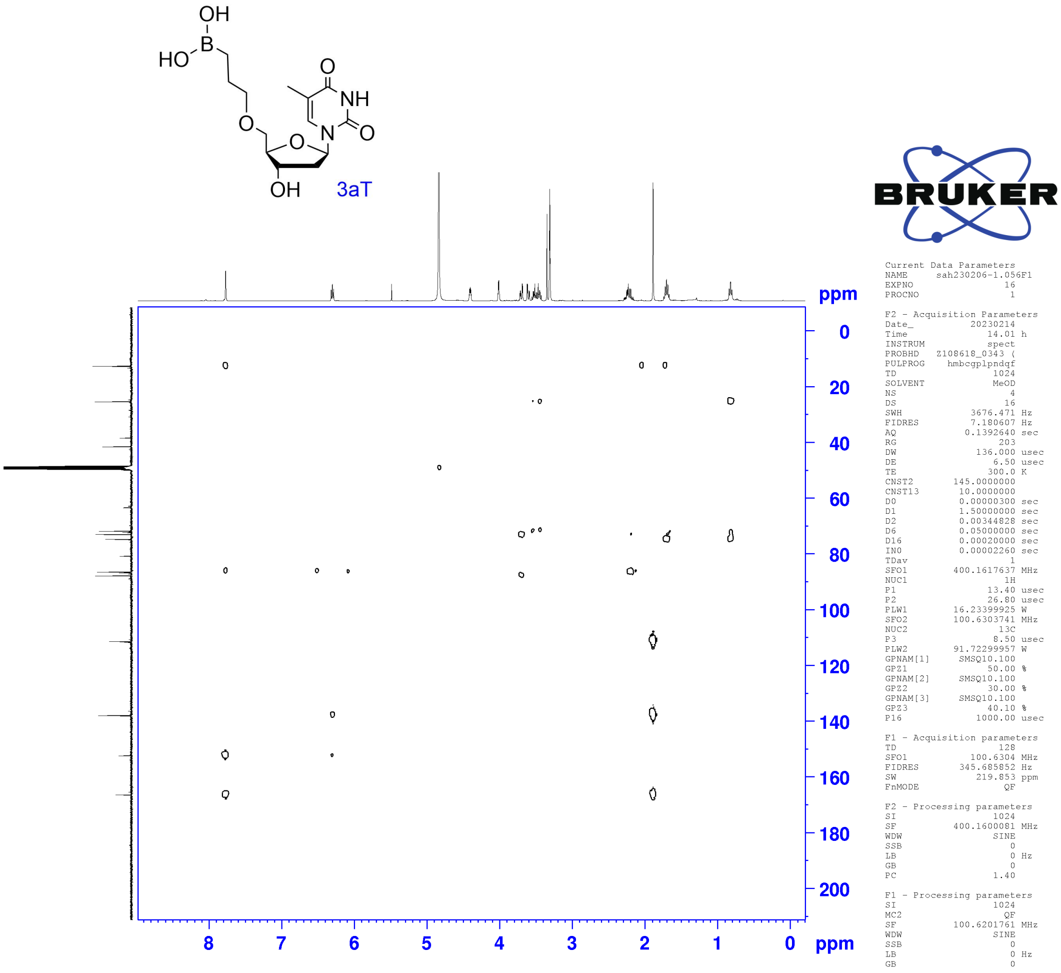

Figure S142 HMBC-NMR spectra of **3aT** in CD_3OD.

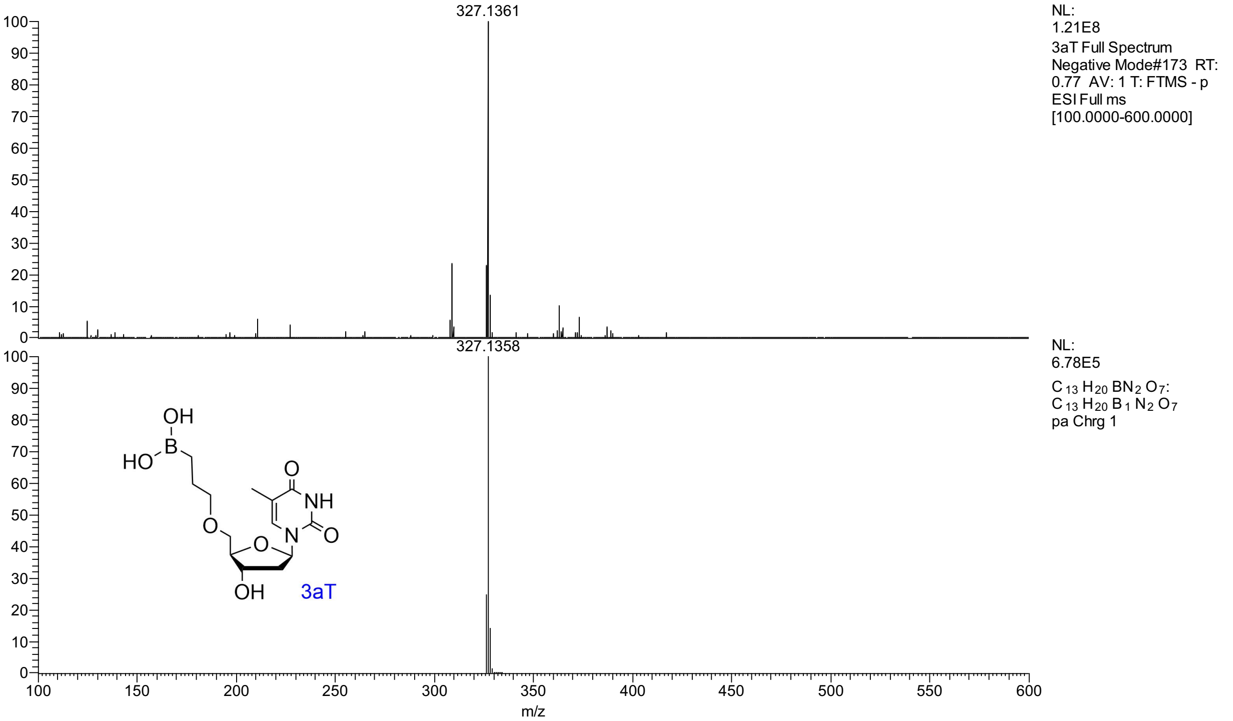

Figure S143 HRMS (ESI-) negative mode m/z calculated for **3aT** [$C_{13}H_{20}BN_2O_7$] [M-H]$^-$ 327.1361, found 327.1360.

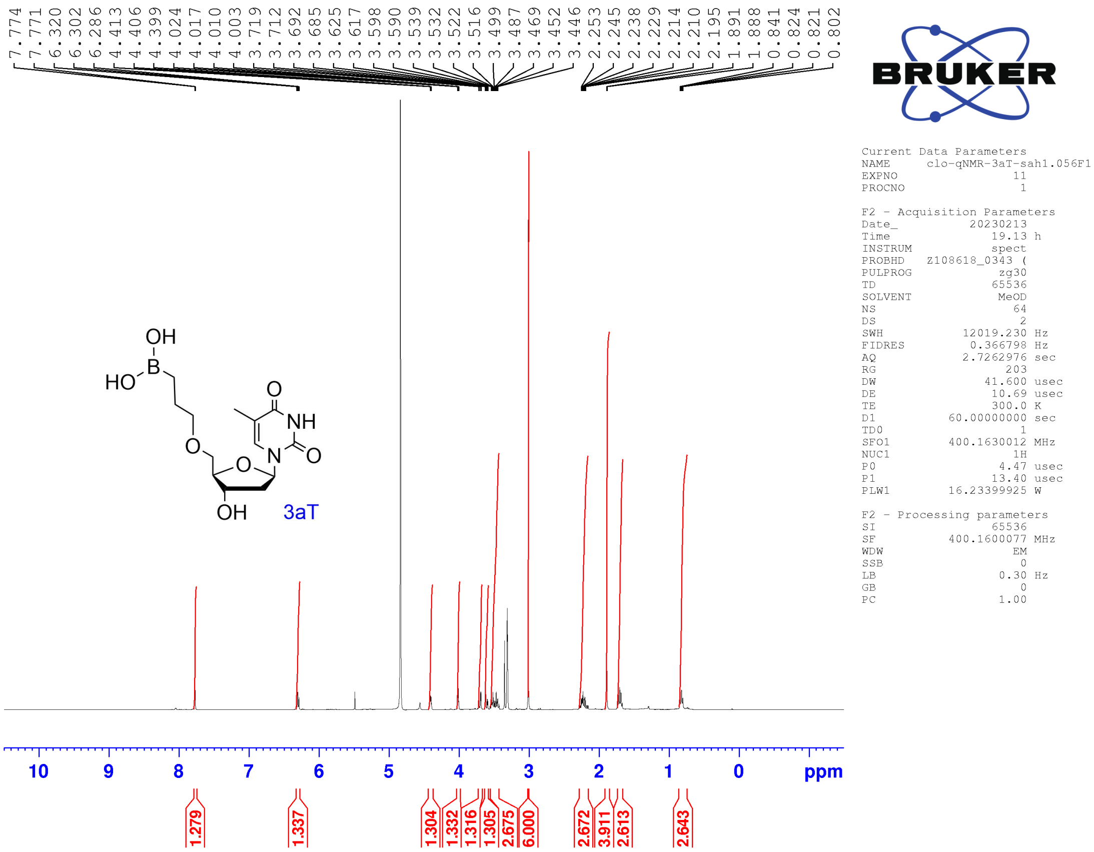

Figure S144 ^{1}H qNMR purity spectrum of **3aT** with Me_2SO_2 (I.C) in CD_3OD. Purity 95.92%.

$$P_{\text{sample}} = \frac{S_{\text{sample}} \text{ x } N_{\text{std}} \text{ x } m_{\text{std}} \text{ x } M_{\text{sample}}}{S_{\text{std}} \text{ x } N_{\text{sample}} \text{ x } m_{\text{sample}} \text{ x } M_{\text{std}}} \text{ x } P_{\text{std}}$$

$$= \frac{1.337 \text{ x } 6 \text{ x } 2.1 \text{ mg x } 328.13 \text{ g mol}^{-1}}{6 \text{ x } 1 \text{ x } 10.2 \text{ mg x } 94.13 \text{ g mol}^{-1}} \text{ x } 99.96$$

$$= 95.92\%$$

S = Integrated area of the peak
N = Number of protons represented
m = Prepared mass
M = Molecular weight
P = Purity

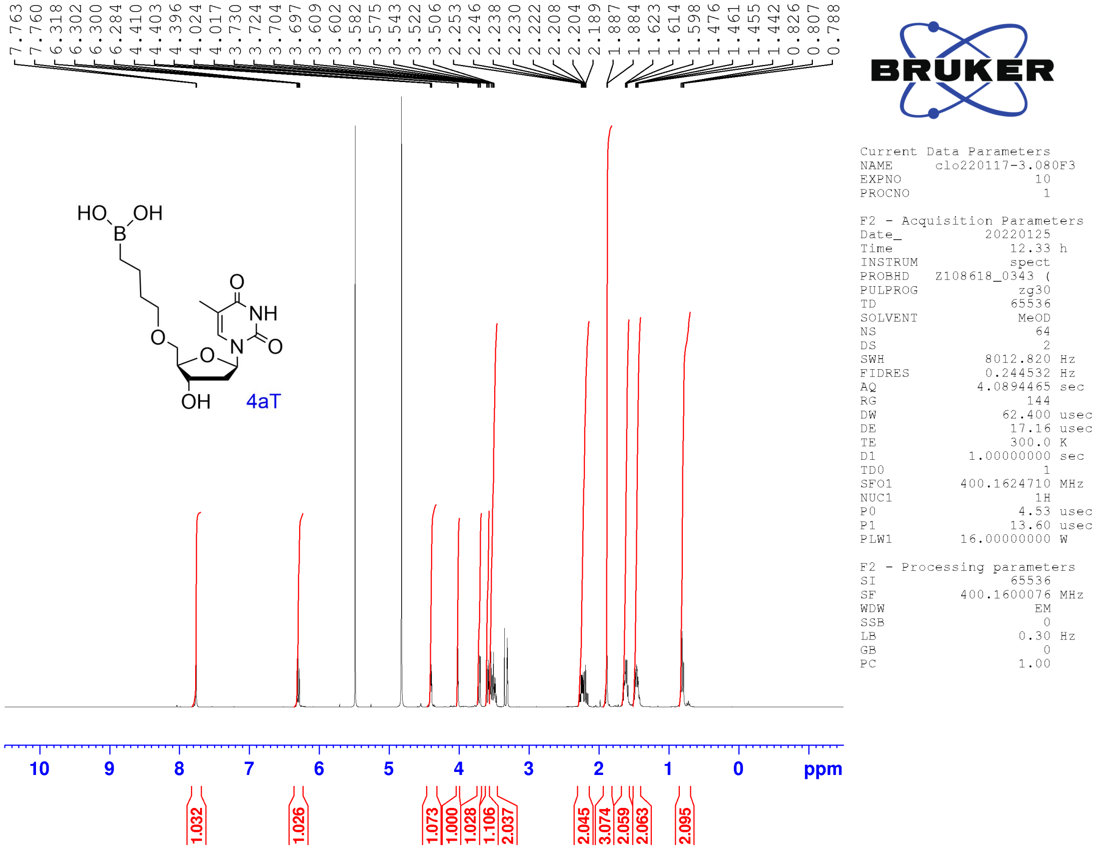

Figure S145 ^{1}HNMR spectra of **4aT** in CD_3OD.

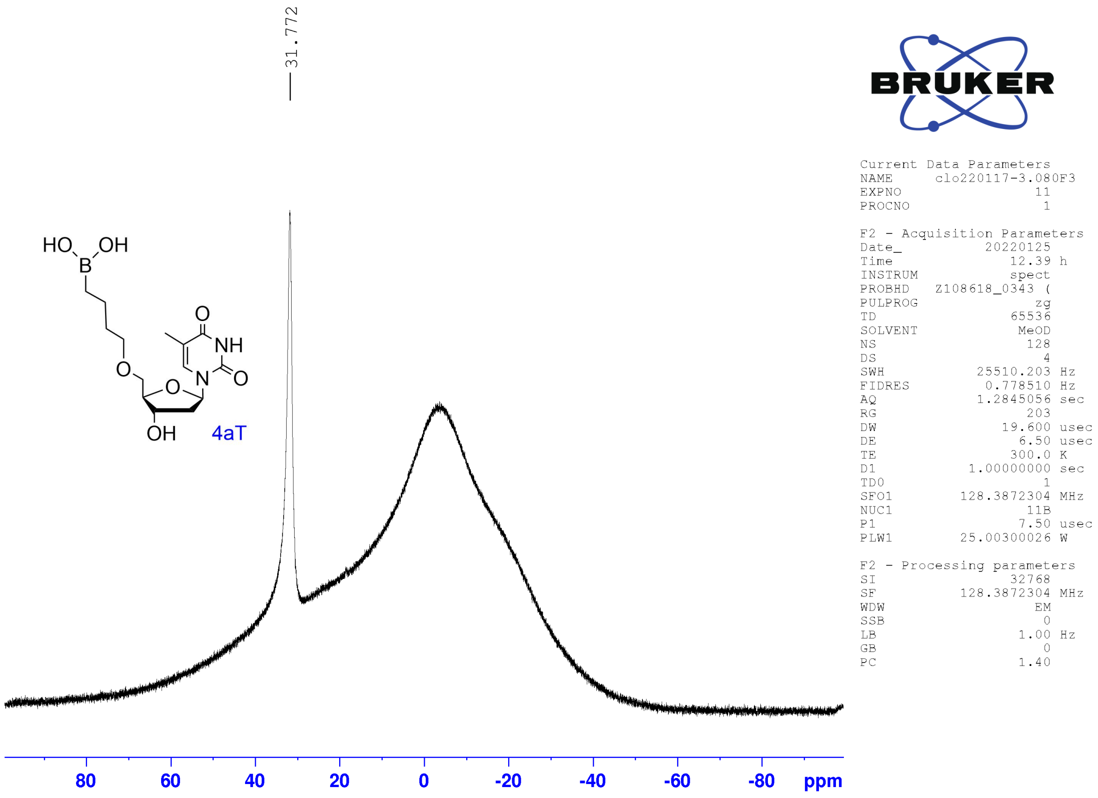

Figure S146 ^{11}BNMR spectra of **4aT** in CD_3OD.

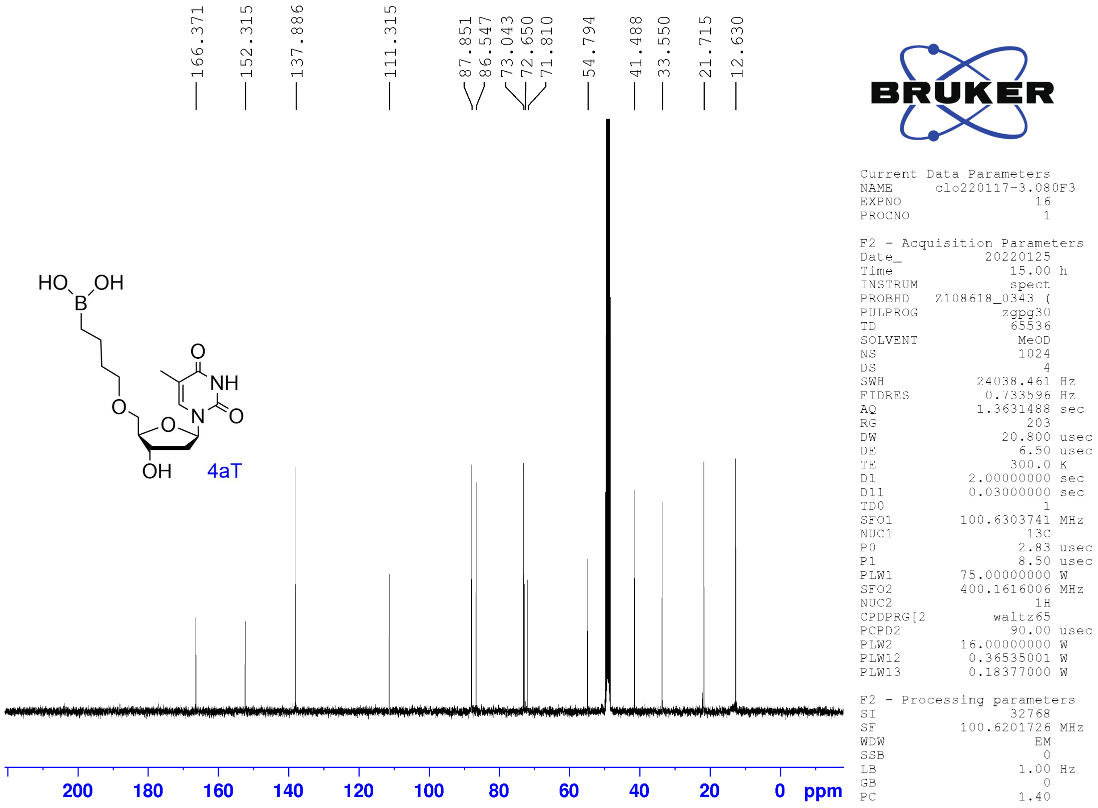

Figure S147 $^{13}C\{^{1}H\}$-NMR spectra of **4aT** in CD_3OD.

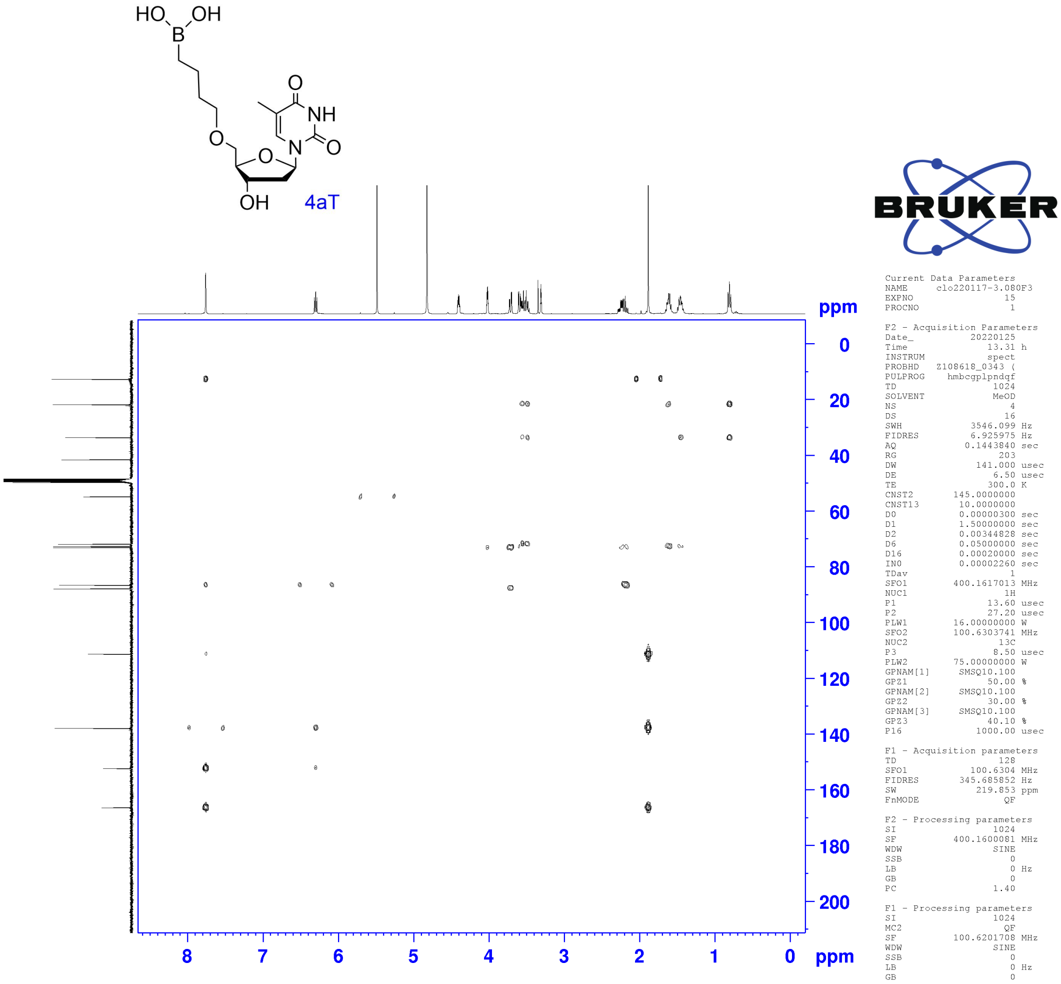

Figure S148 HMBC-NMR spectra of **4aT** in CD_3OD.

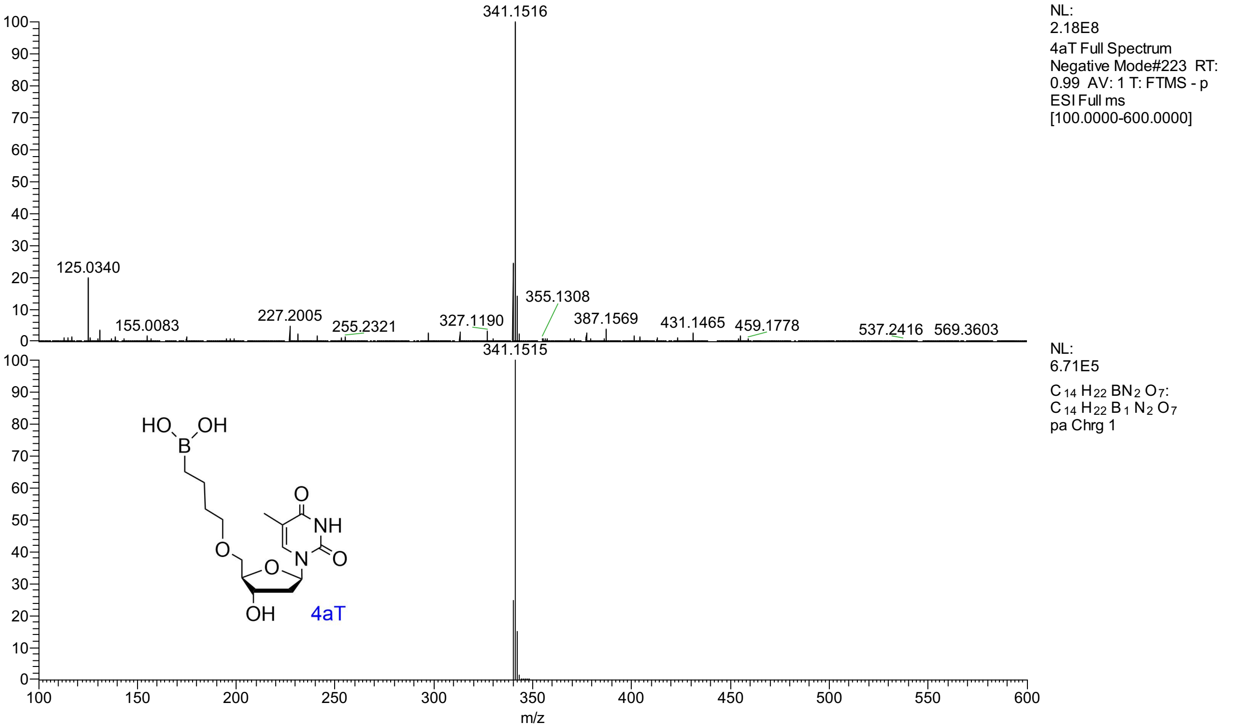

Figure S149 HRMS (ESI-) negative mode m/z calculated for **4aT** [$C_{14}H_{22}BN_2O_7$] [M-H]$^-$ 341.1515, found 341.1516.

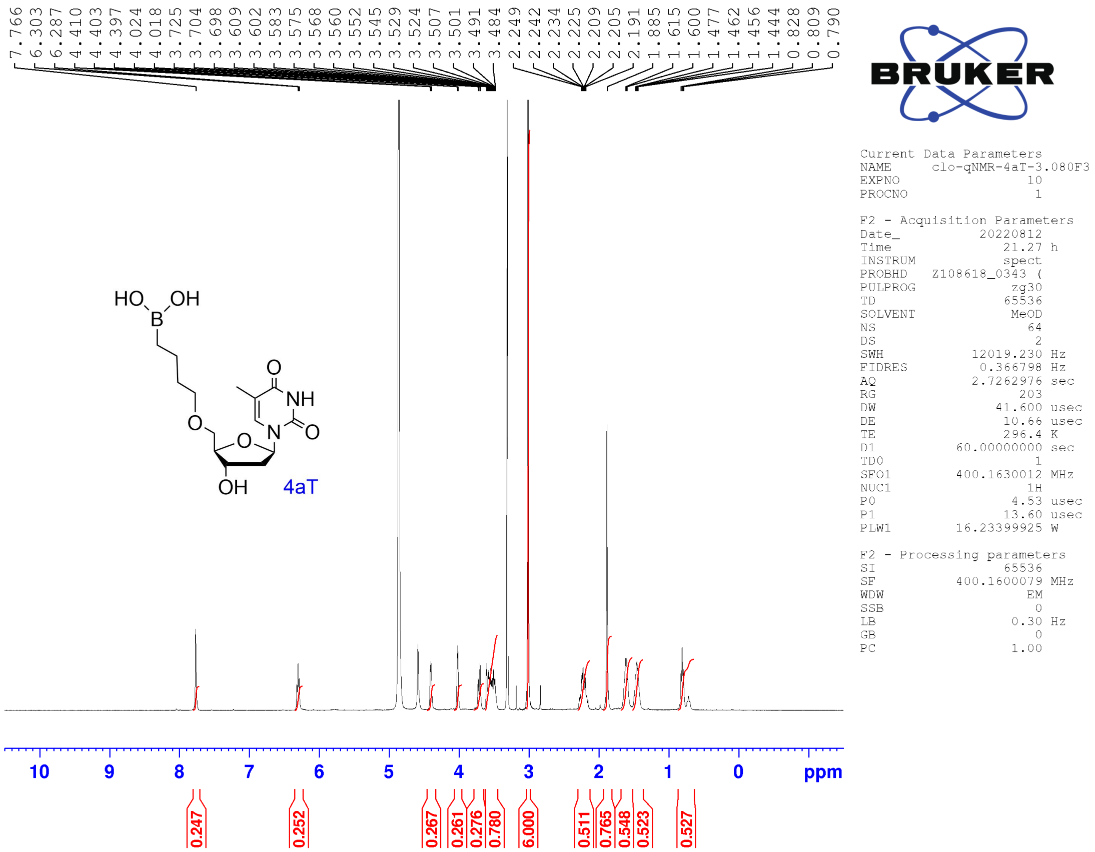

Figure S150 ^{1}H qNMR purity spectrum of **4aT** with Me_2SO_2 (I.C) in CD_3OD. Purity 95.46%.

$$P_{\text{sample}} = \frac{S_{\text{sample}} \text{ x } N_{\text{std}} \text{ x } m_{\text{std}} \text{ x } M_{\text{sample}}}{S_{\text{std}} \text{ x } N_{\text{sample}} \text{ x } m_{\text{sample}} \text{ x } M_{\text{std}}} \text{ x } P_{\text{std}}$$

$$= \frac{0.252 \text{ x } 6 \text{ x } 4.9 \text{ mg x } 342.16 \text{ g mol}^{-1}}{6 \text{ x } 1 \text{ x } 4.7 \text{ mg x } 94.13 \text{ g mol}^{-1}} \text{ x } 99.96$$

$$= 95.46\%$$

S = Integrated area of the peak
N = Number of protons represented
m = Prepared mass
M = Molecular weight
P = Purity

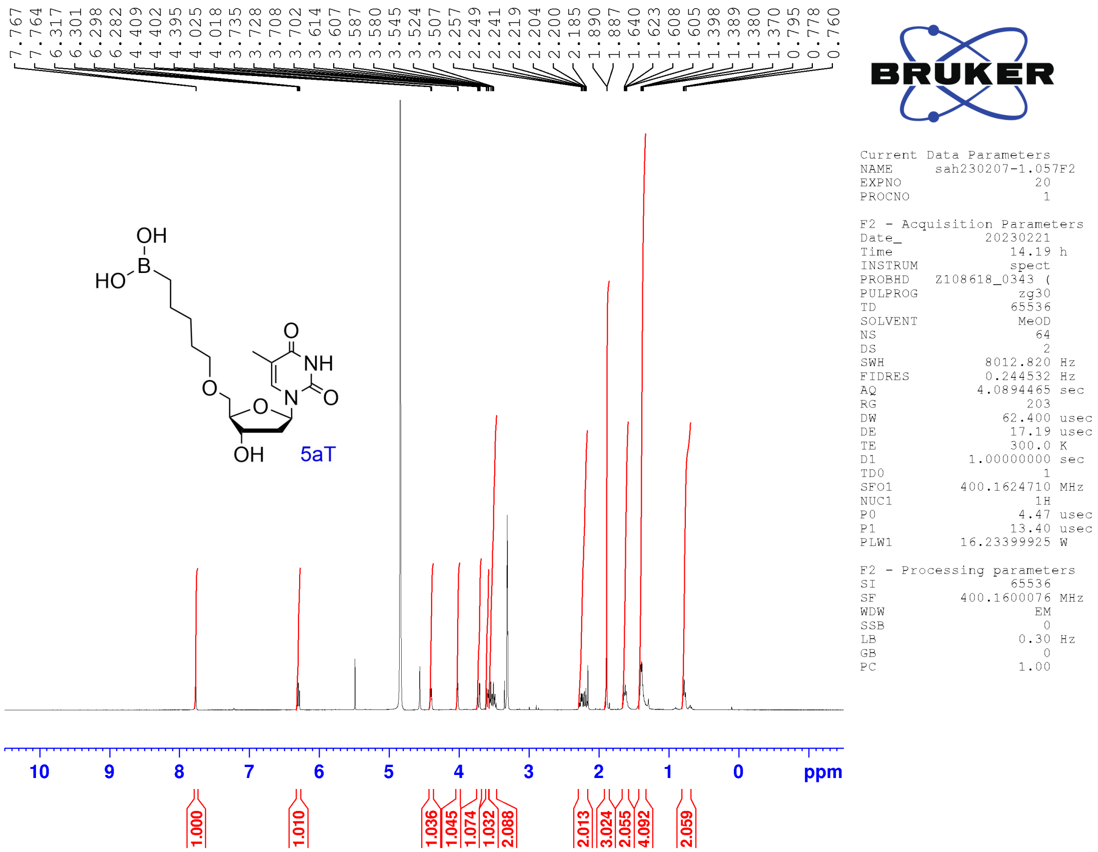

Figure S151 ^{1}HNMR spectra of **5aT** in CD_3OD.

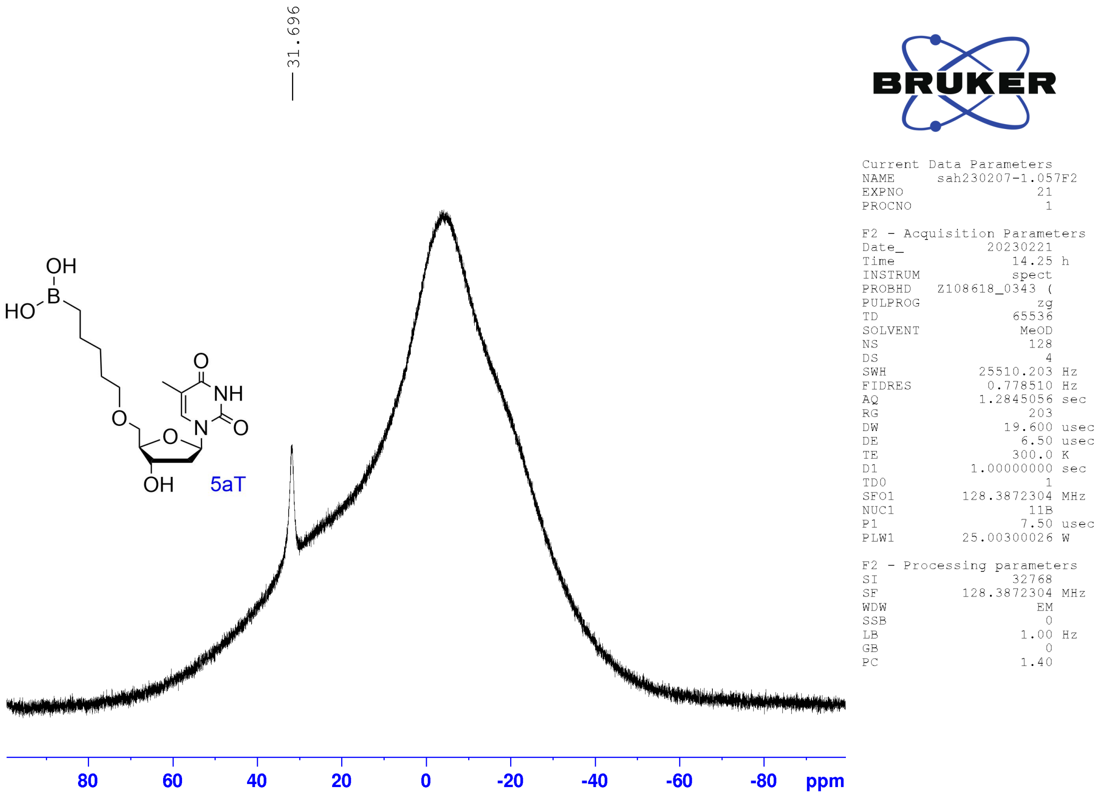

Figure S152 ^{11}BNMR spectra of **5aT** in CD_3OD.

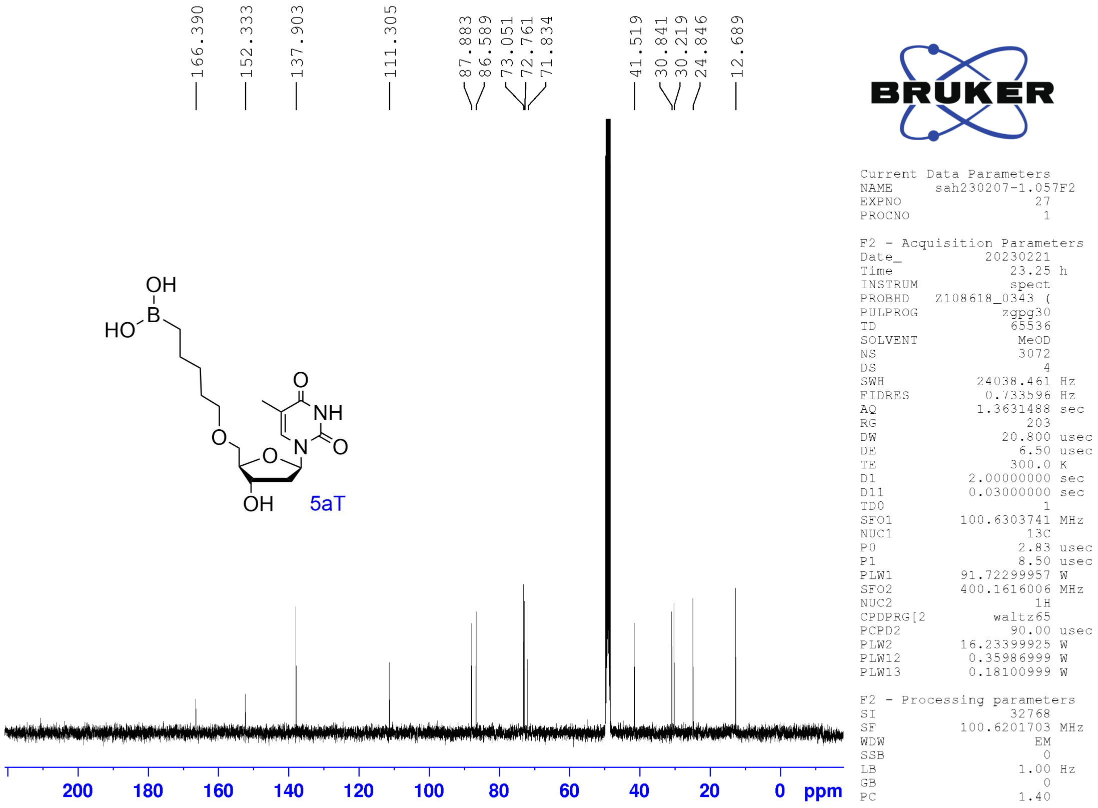

Figure S153 $^{13}C\{^{1}H\}$-NMR spectra of **5aT** in CD_3OD.

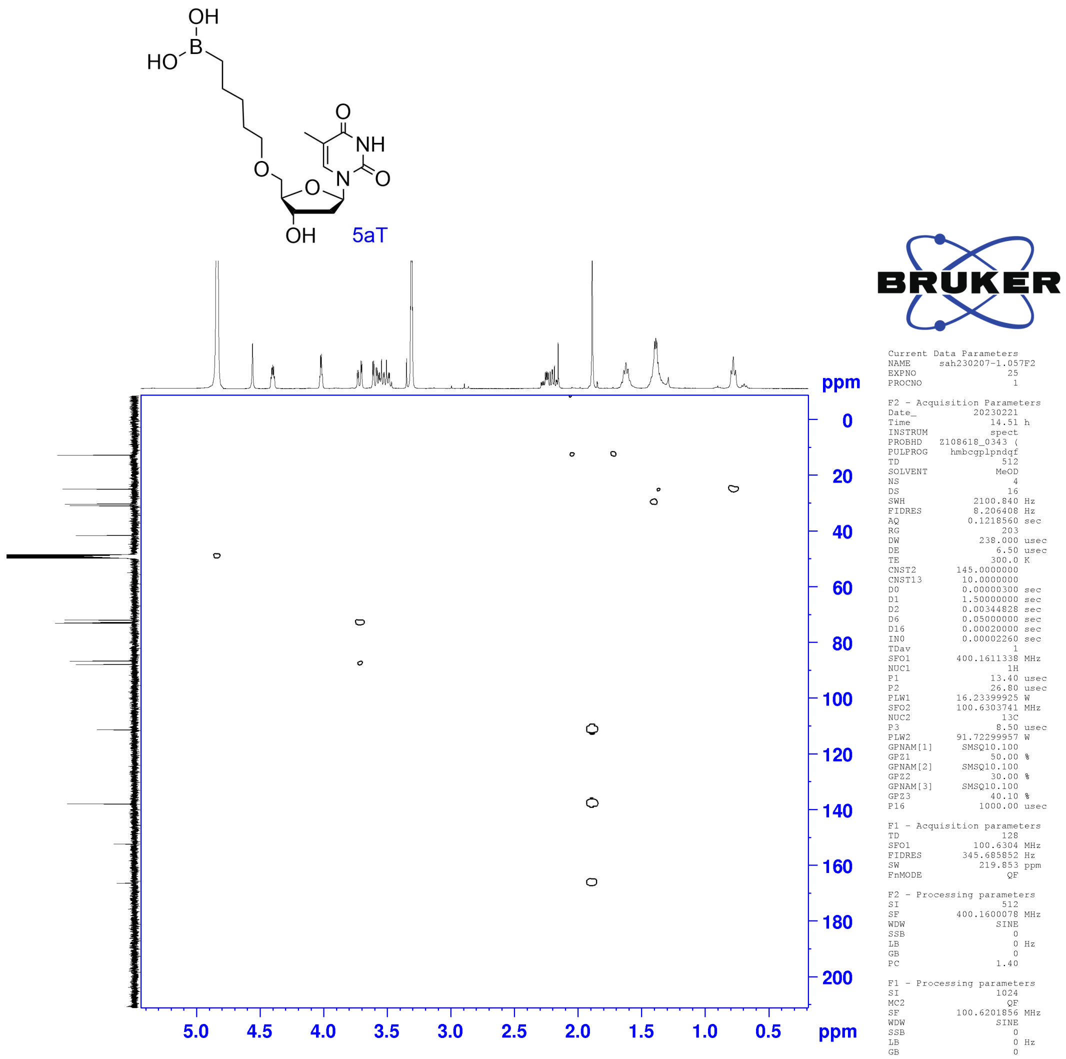

Figure S154 HMBC-NMR spectra of **5aT** in CD_3OD.

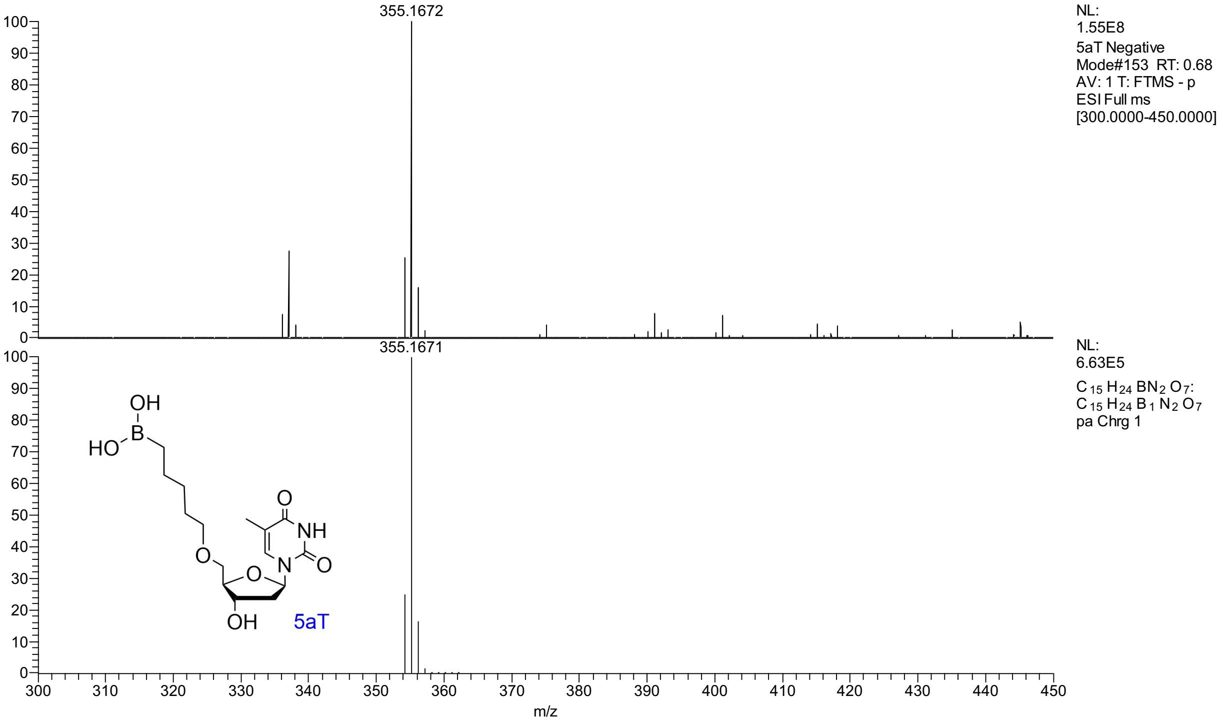

Figure S155 HRMS (ESI-) negative mode m/z calculated for **5aT** [$C_{15}H_{24}BN_2O_7$] [M-H]$^-$ 355.1671, found 355.1672.

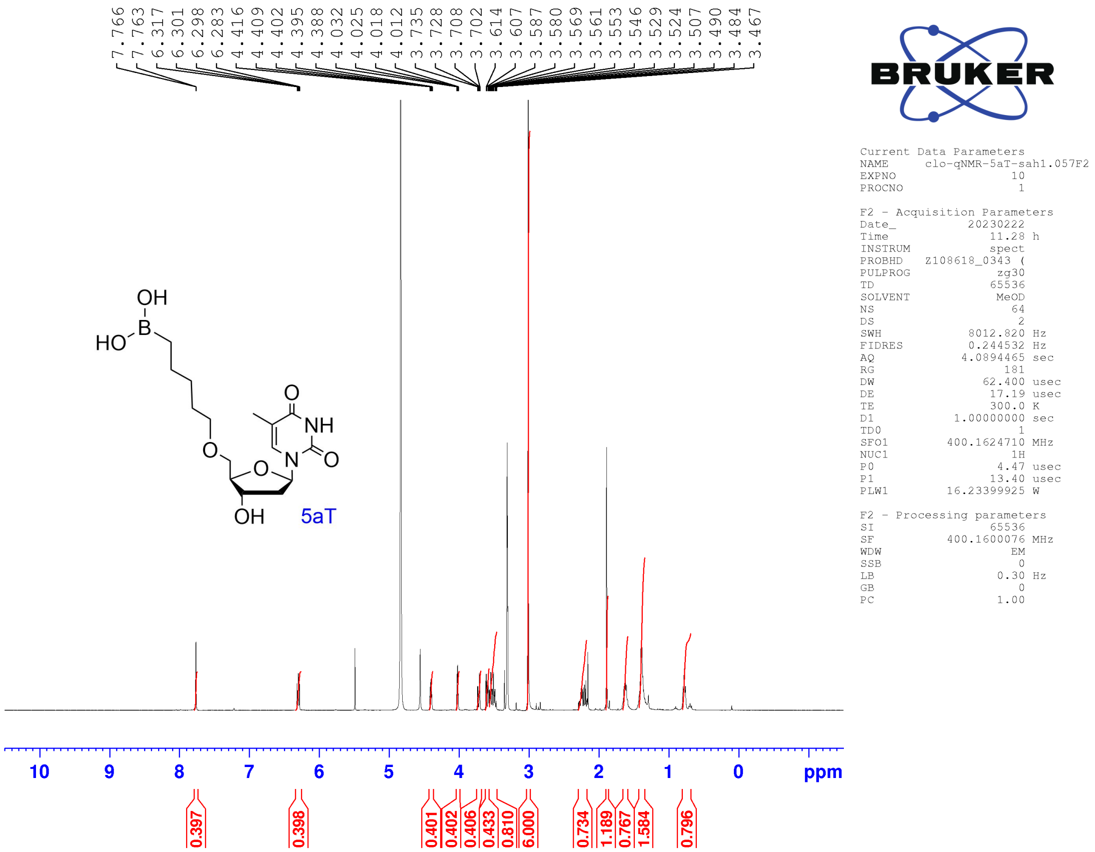

Figure S156 ^{1}H qNMR purity spectrum of **5aT** with Me_2SO_2 (I.C) in CD_3OD. Purity 96.59%.

$$P_{\text{sample}} = \frac{S_{\text{sample}} \text{ x } N_{\text{std}} \text{ x } m_{\text{std}} \text{ x } M_{\text{sample}}}{S_{\text{std}} \text{ x } N_{\text{sample}} \text{ x } m_{\text{sample}} \text{ x } M_{\text{std}}} \text{ x } P_{\text{std}}$$

$$= \frac{0.401 \text{ x } 6 \text{ x } 2.1 \text{ mg x } 356.18 \text{ g mol}^{-1}}{6 \text{ x } 1 \text{ x } 3.3 \text{ mg x } 94.13 \text{ g mol}^{-1}} \text{ x } 99.96$$

$$= 96.59\%$$

S = Integrated area of the peak
N = Number of protons represented
m = Prepared mass
M = Molecular weight
P = Purity

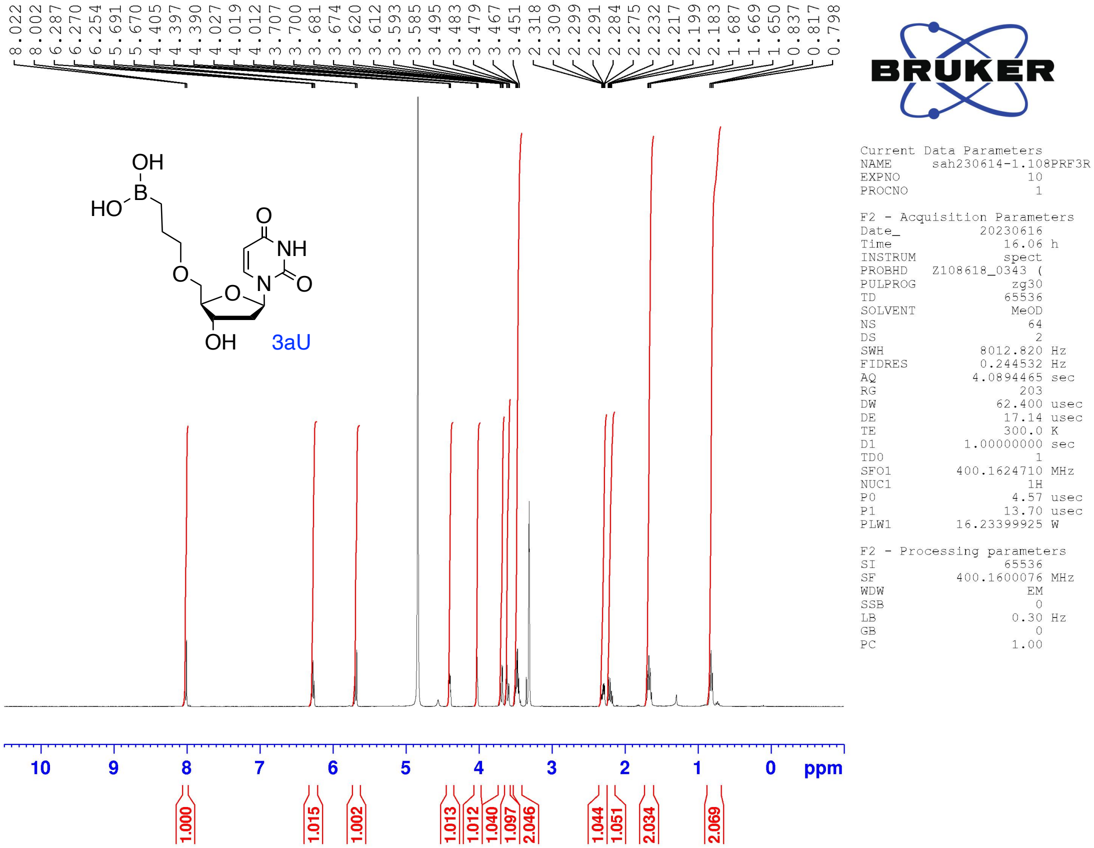

Figure S157 ^{1}HNMR spectra of **3aU** in CD_3OD.

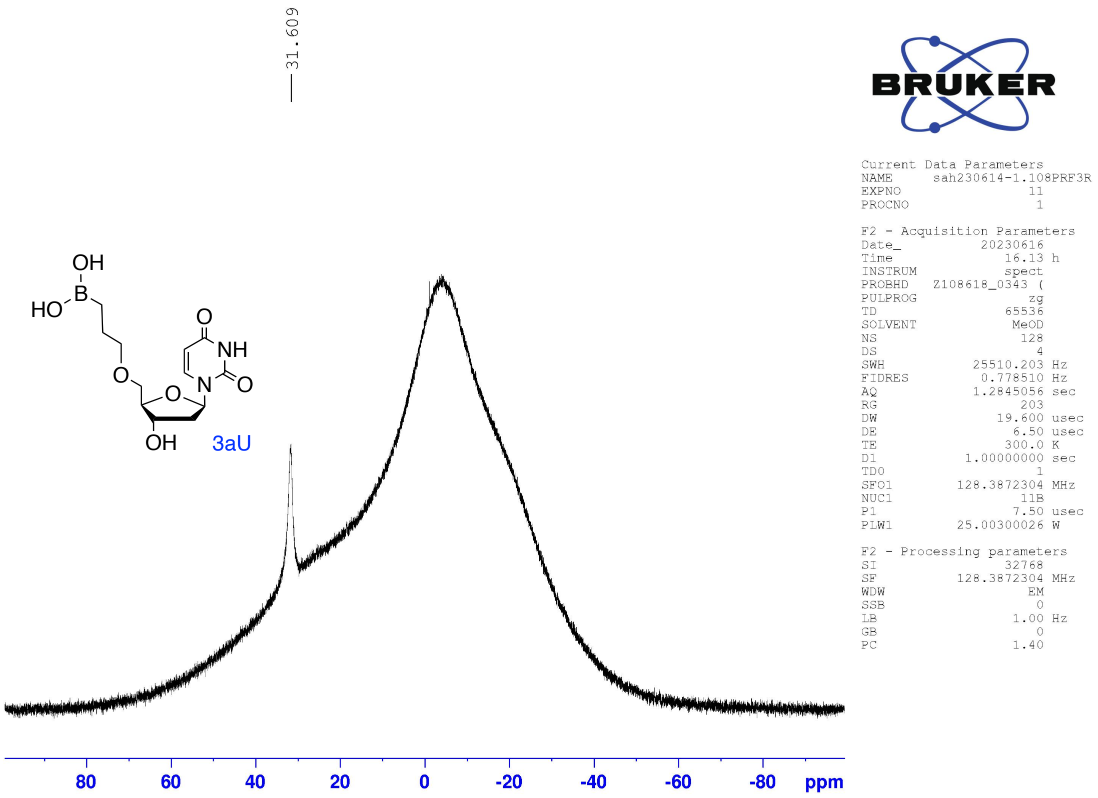

Figure S158 ^{11}BNMR spectra of **3aU** in CD_3OD.

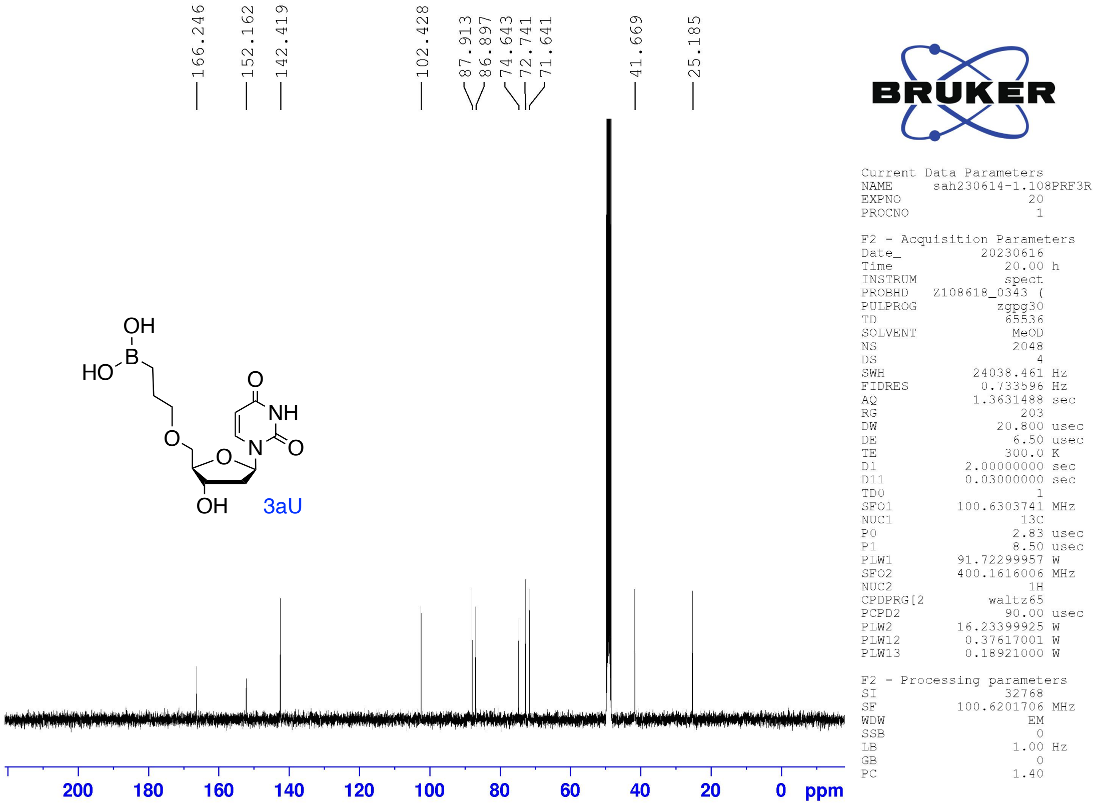

Figure S159 $^{13}C\{^1H\}$-NMR spectra of **3aU** in CD_3OD.

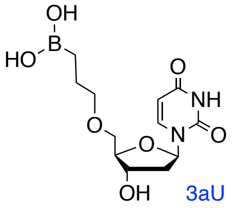

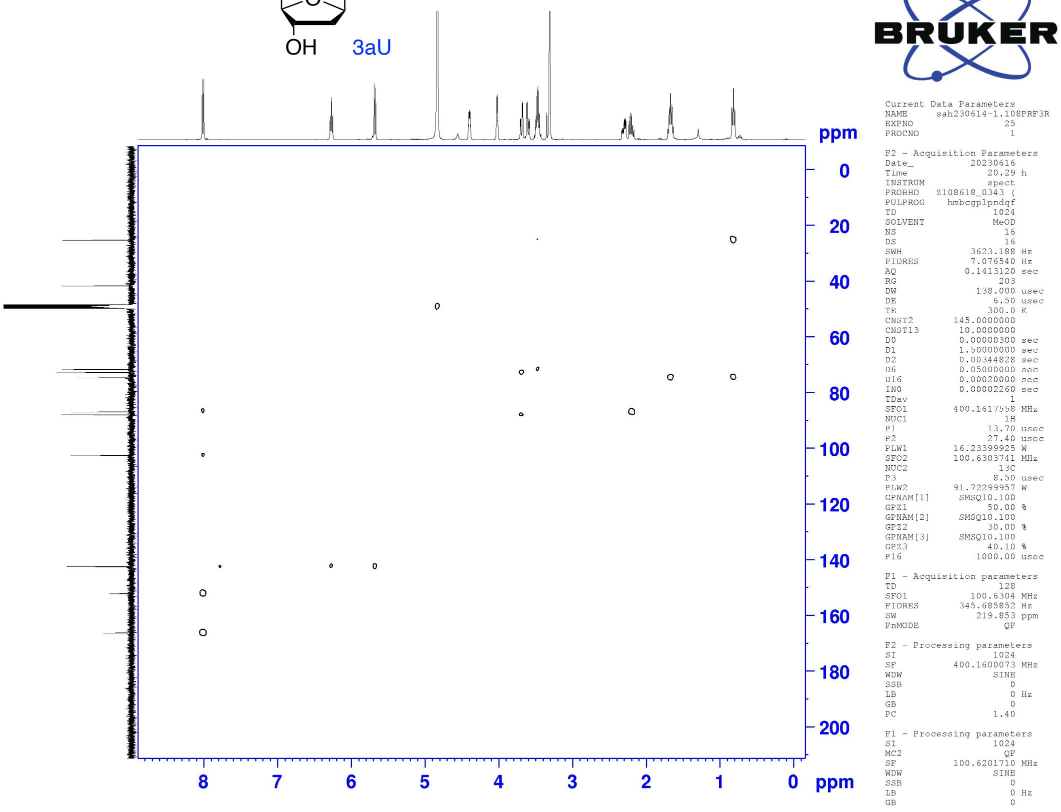

Figure S160 HMBC-NMR spectra of **3aU** in CD_3OD.

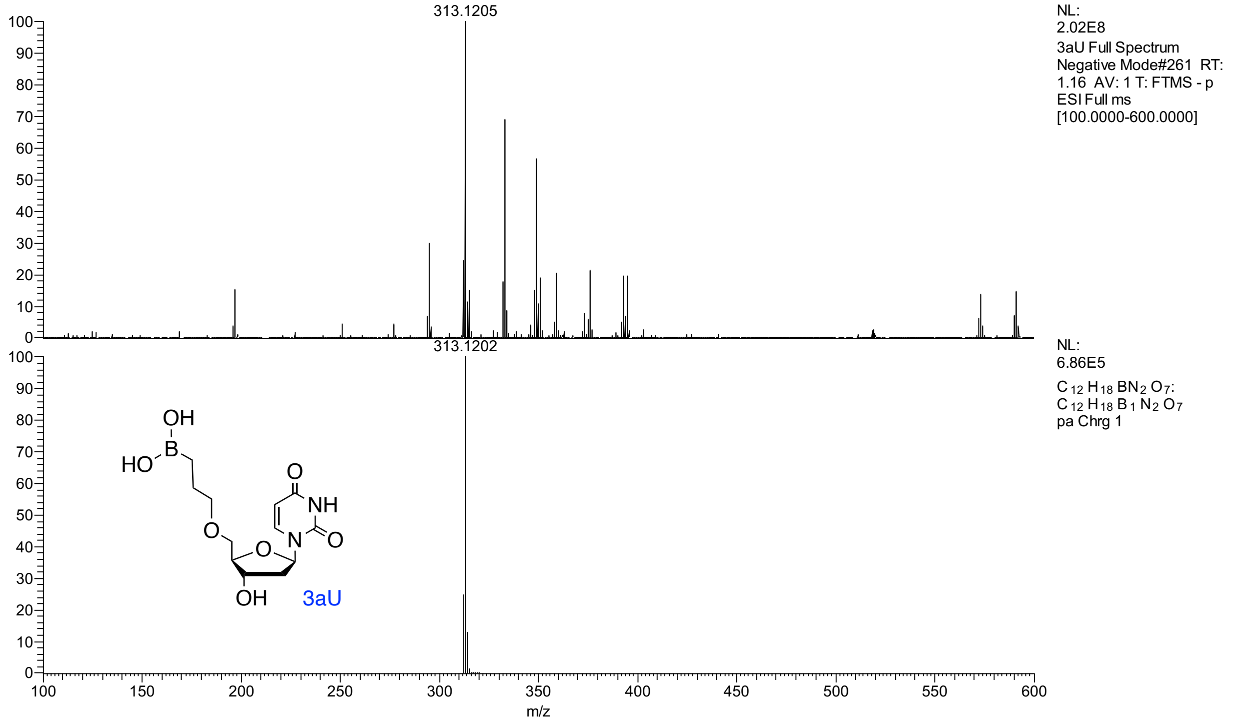

Figure S161 HRMS (ESI-) negative mode m/z calculated for **3aU** [$C_{12}H_{18}BN_2O_7$] $[M\text{-}H]^-$ 313.1202, found 313.1205.

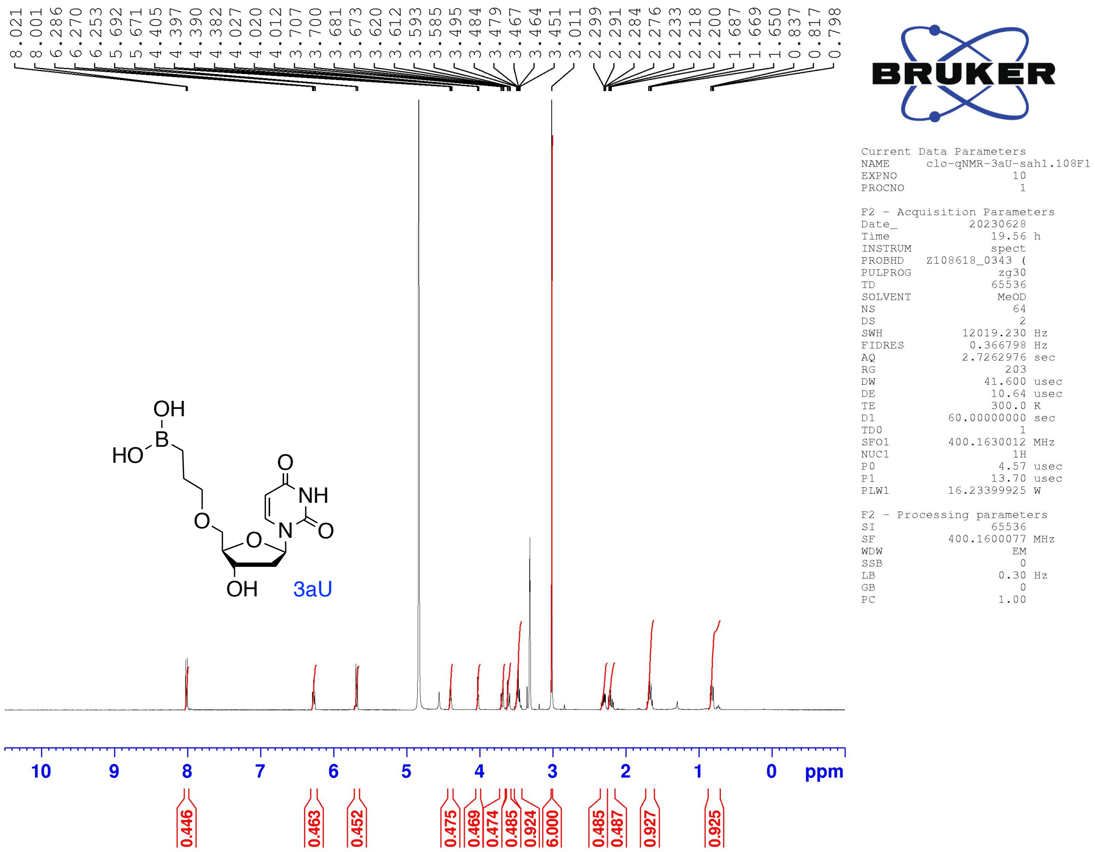

Figure S162 ^{1}H qNMR purity spectrum of **3aU** with Me_2SO_2 (I.C) in CD_3OD. Purity 95.64%.

$$P_{\text{sample}} = \frac{S_{\text{sample}} \text{ x } N_{\text{std}} \text{ x } m_{\text{std}} \text{ x } M_{\text{sample}}}{S_{\text{std}} \text{ x } N_{\text{sample}} \text{ x } m_{\text{sample}} \text{ x } M_{\text{std}}} \text{ x } P_{\text{std}}$$

$$= \frac{0.463 \text{ x } 6 \text{ x } 2.7 \text{ mg x } 314.10 \text{ g mol}^{-1}}{6 \text{ x } 1 \text{ x } 4.4 \text{ mg x } 94.13 \text{ g mol}^{-1}} \text{ x } 99.96$$

$$= 95.64\%$$

S = Integrated area of the peak
N = Number of protons represented
m = Prepared mass
M = Molecular weight
P = Purity

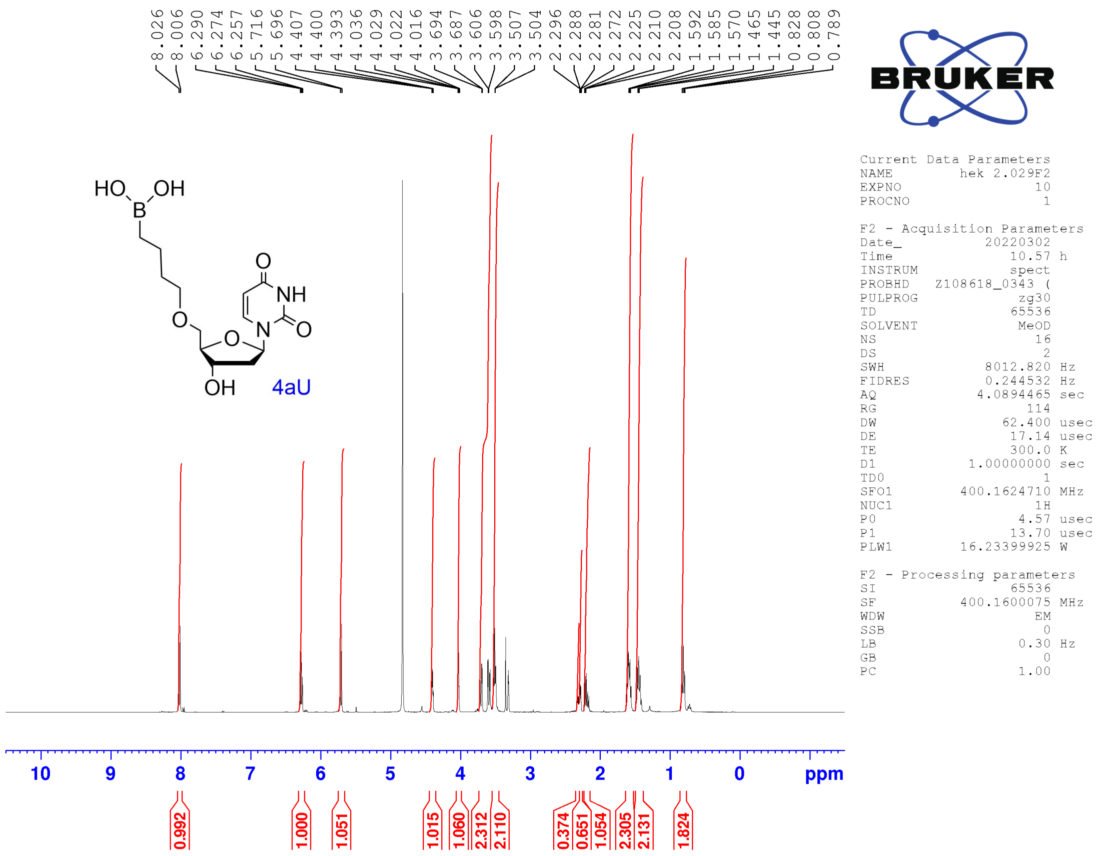

Figure S163 ^{1}HNMR spectra of **4aU** in CD3OD.

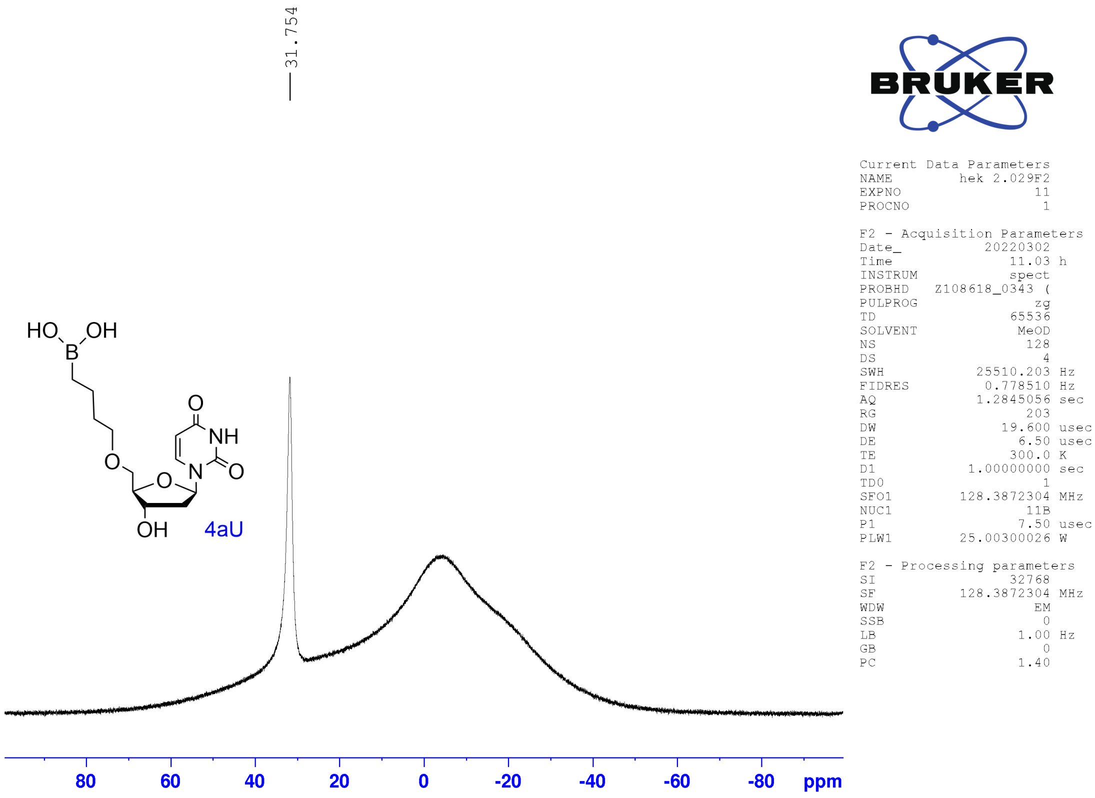

Figure S164 ^{11}BNMR spectra of **4aU** in CD_3OD.

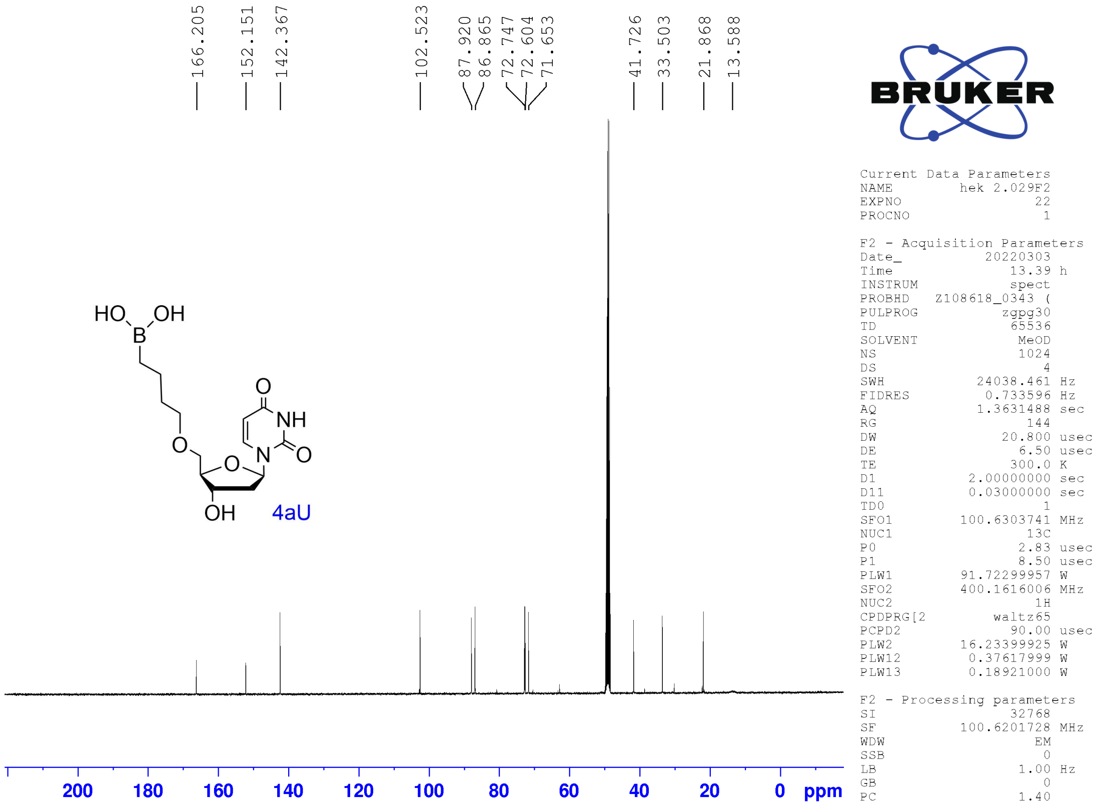

Figure S165 $^{13}C\{^{1}H\}$-NMR spectra of **4aU** in CD_3OD.

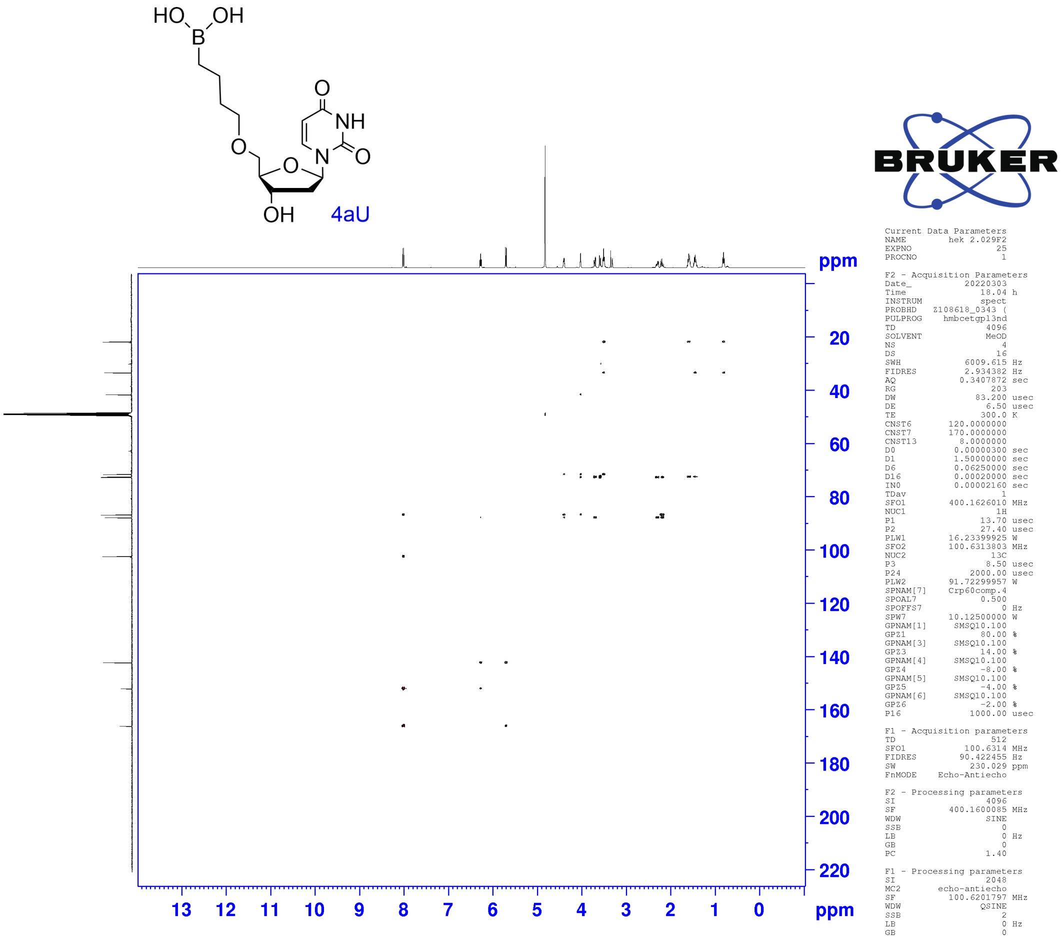

Figure S166 HMBC-NMR spectra of **4aU** in CD_3OD.

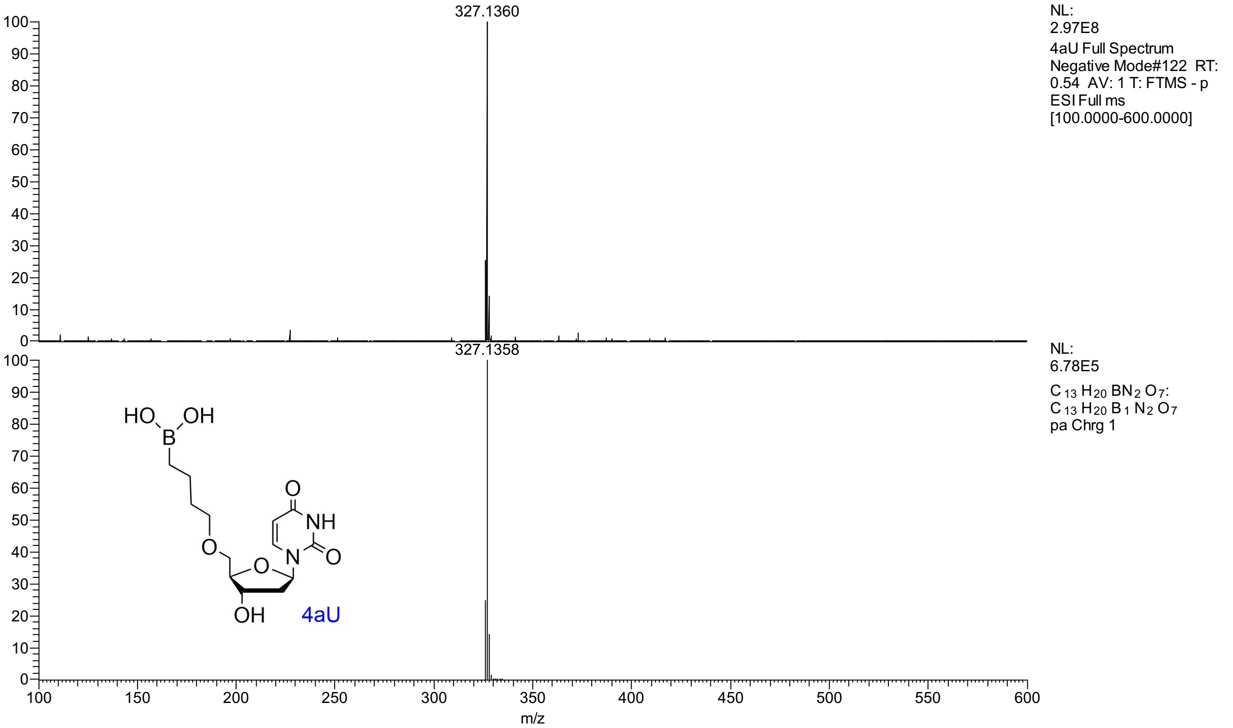

Figure S167 HRMS (ESI-) negative mode m/z calculated for **4aU** [$C_{13}H_{20}BN_2O_7$] [M-H]$^-$ 327.1358, found 327.1360.

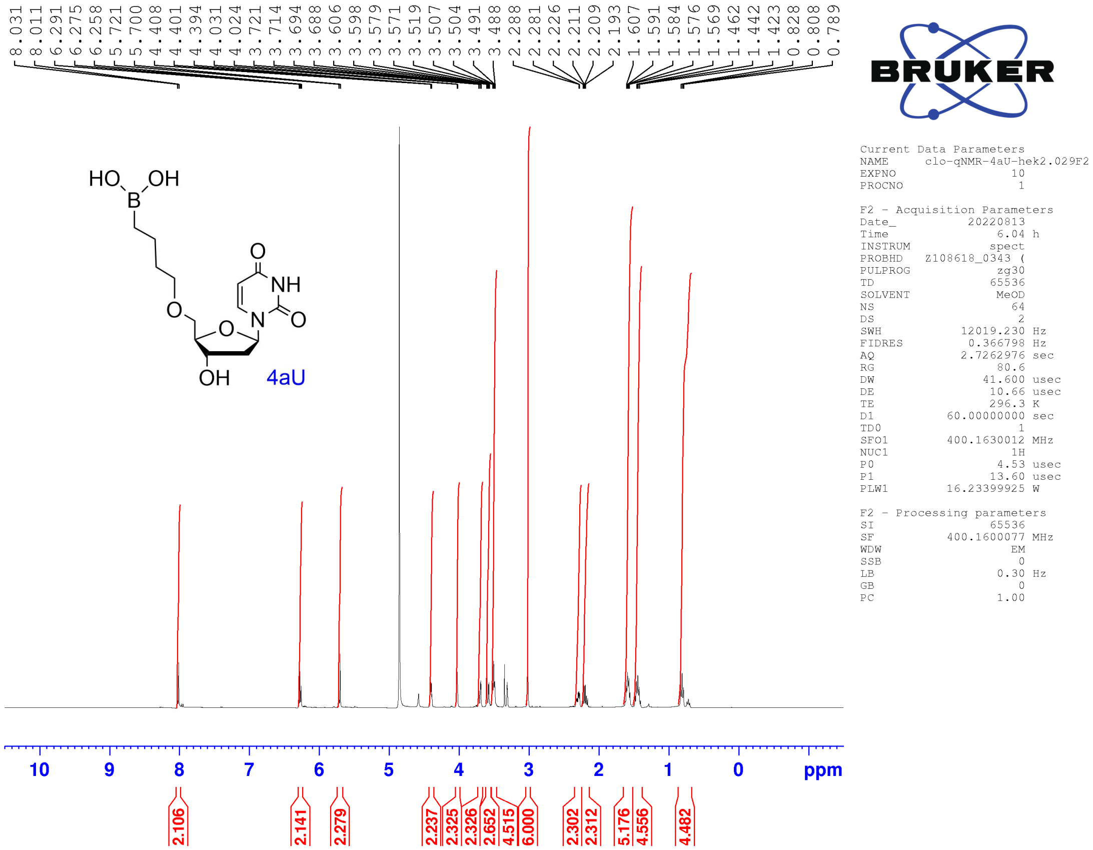

Figure S168 ^{1}H qNMR purity spectrum of **4aU** with Me_2SO_2 (I.C) in CD_3OD. Purity 95.34%.

$$P_{\text{sample}} = \frac{S_{\text{sample}} \text{ x } N_{\text{std}} \text{ x } m_{\text{std}} \text{ x } M_{\text{sample}}}{S_{\text{std}} \text{ x } N_{\text{sample}} \text{ x } m_{\text{sample}} \text{ x } M_{\text{std}}} \text{ x } P_{\text{std}}$$

$$= \frac{2.141 \text{ x } 6 \text{ x } 4.0 \text{ mg x } 328.13 \text{ g mol}^{-1}}{6 \text{ x } 1 \text{ x } 31.3 \text{ mg x } 94.13 \text{ g mol}^{-1}} \text{ x } 99.96$$

$$= 95.34\%$$

S = Integrated area of the peak
N = Number of protons represented
m = Prepared mass
M = Molecular weight
P = Purity

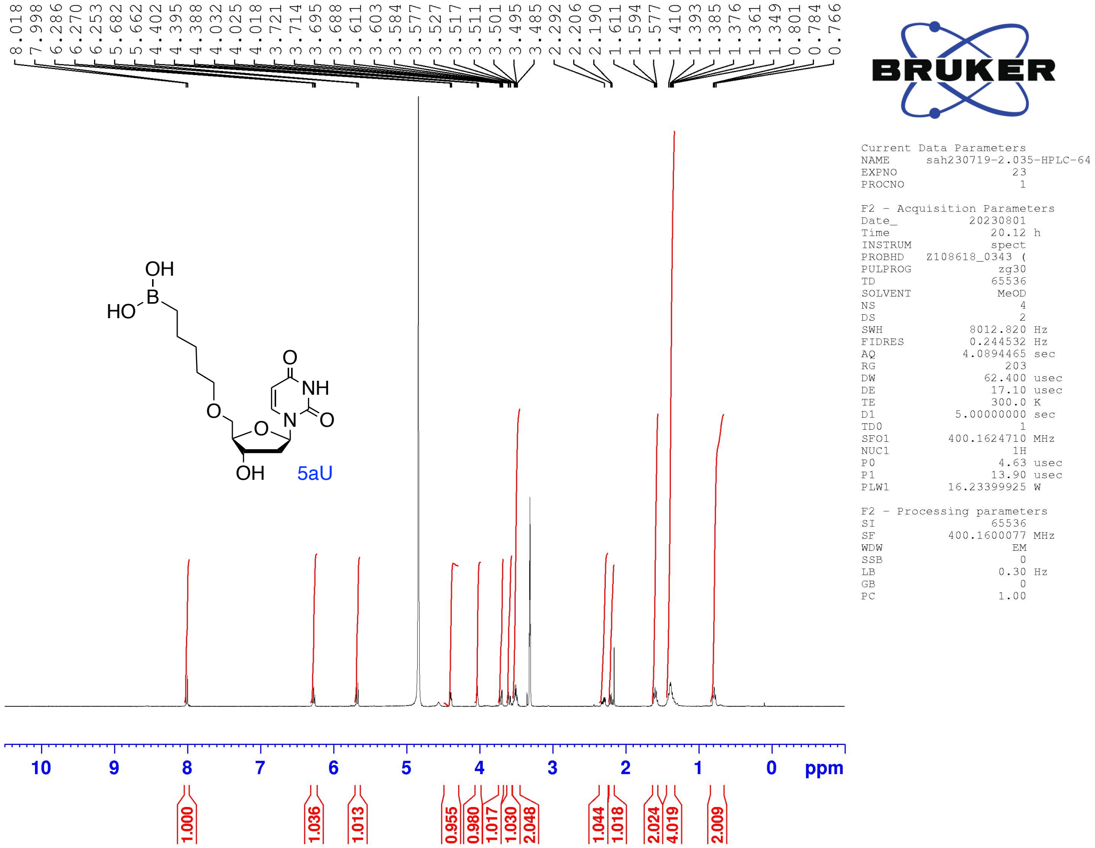

Figure S169 ^{1}HNMR spectra of **5aU** in CD_3OD.

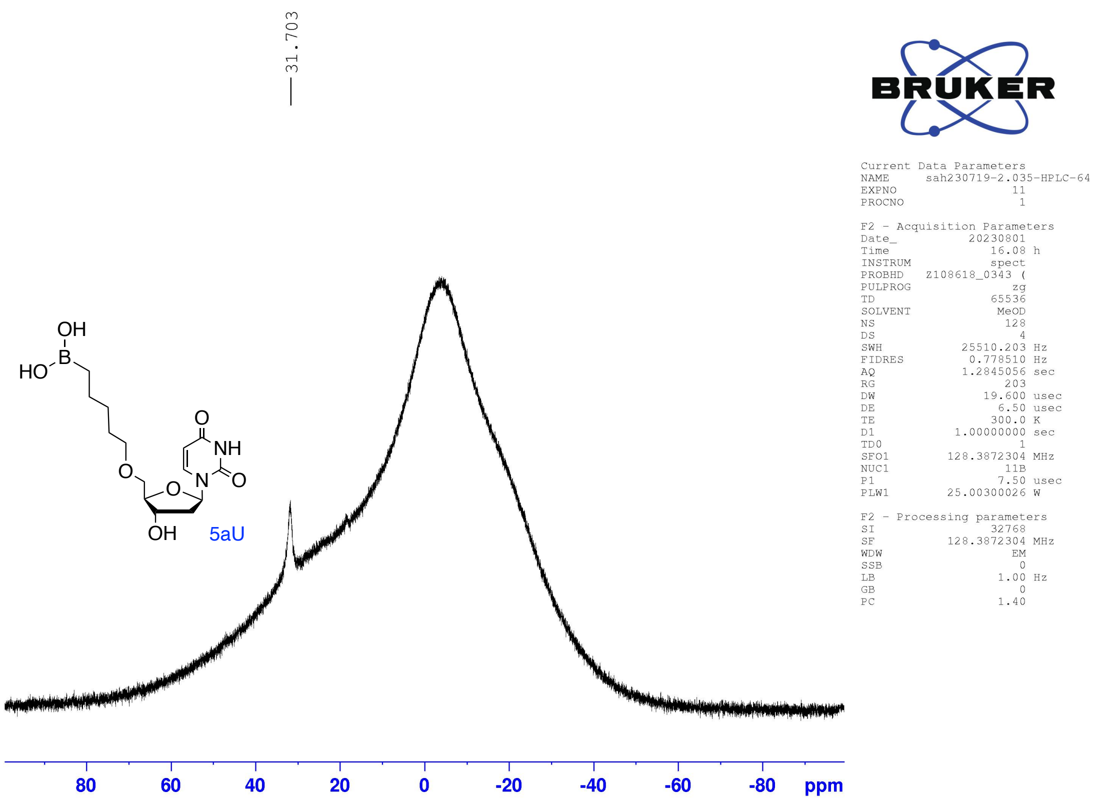

Figure S170 ^{11}BNMR spectra of **5aU** in CD_3OD.

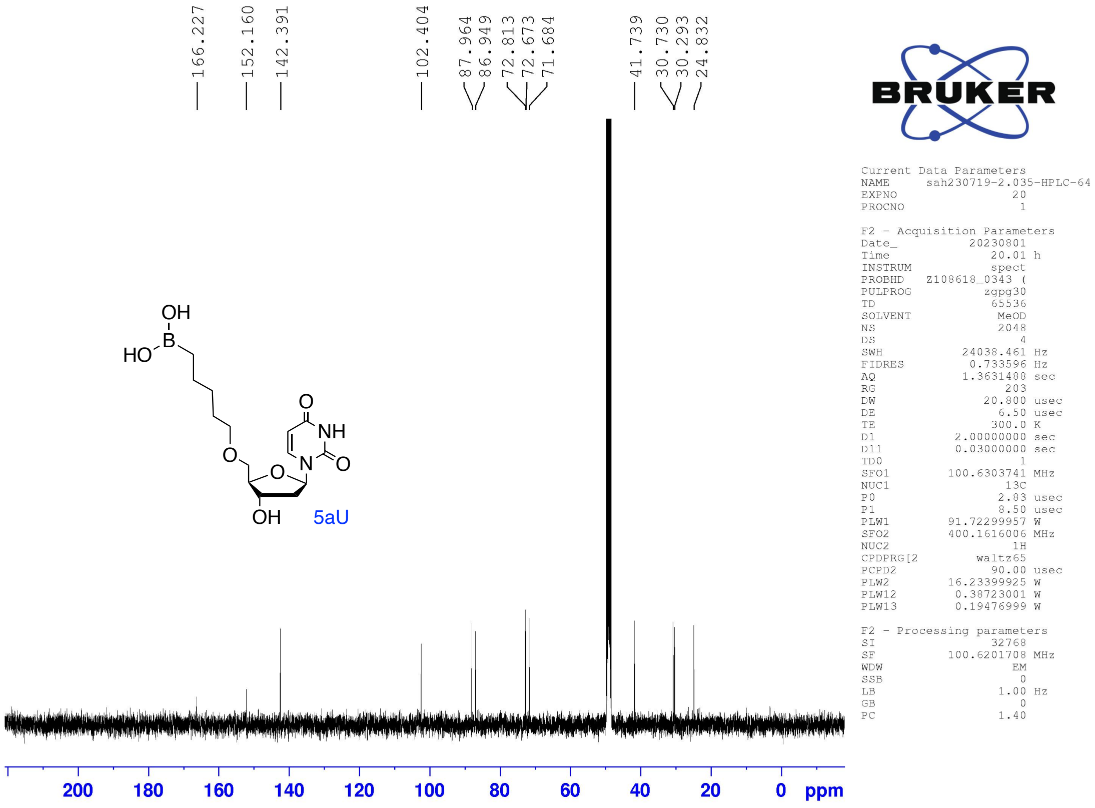

Figure S171 $^{13}C\{^{1}H\}$-NMR spectra of **5aU** in CD_3OD.

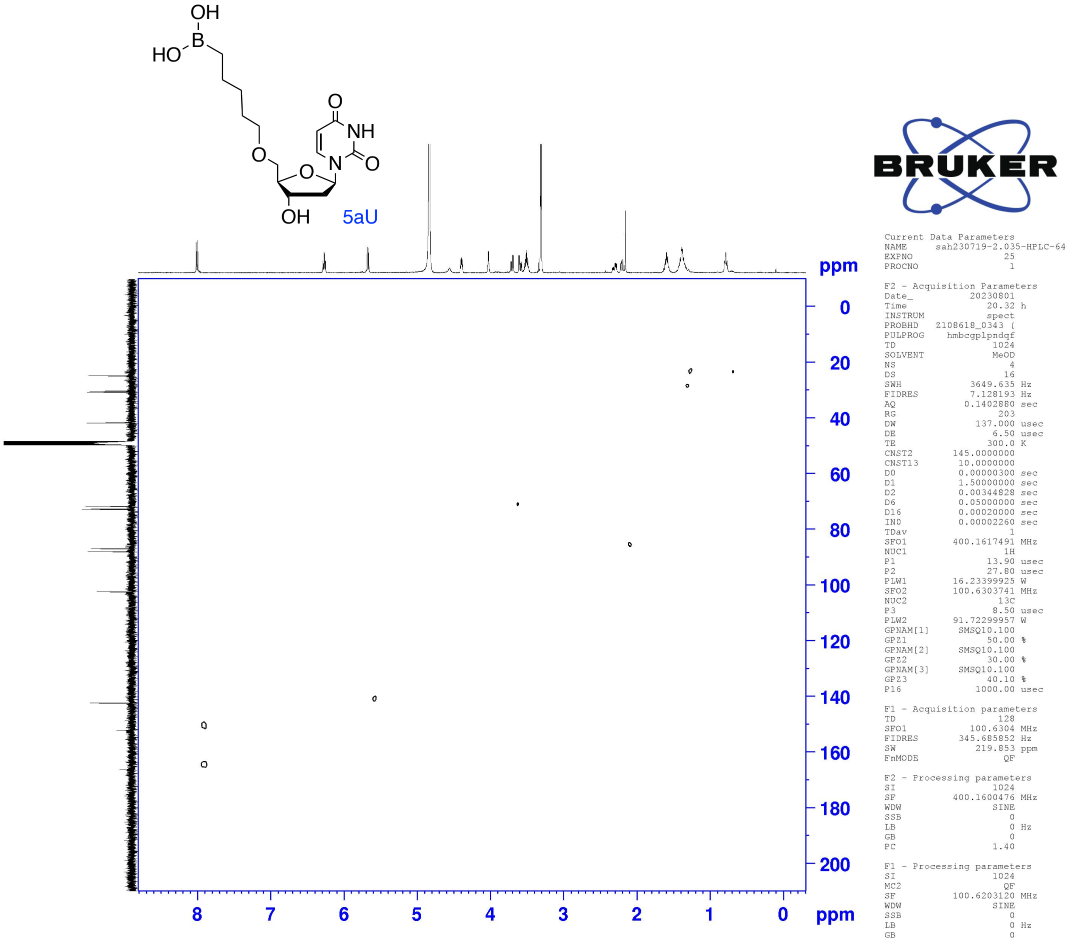

Figure S172 HMBC-NMR spectra of **5aU** in CD_3OD.

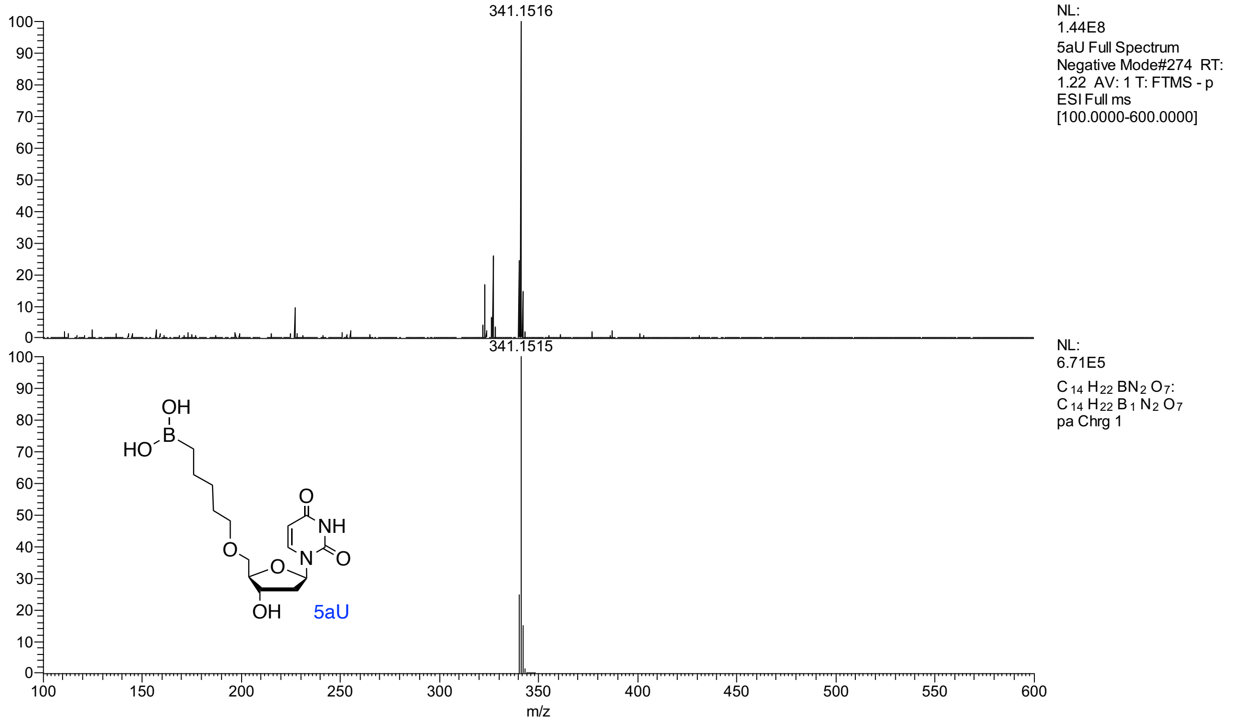

Figure S173 HRMS (ESI-) negative mode m/z calculated for **5aU** [$C_{14}H_{22}BN_2O_7$] [M-H]$^-$ 341.1515, found 341.1516.

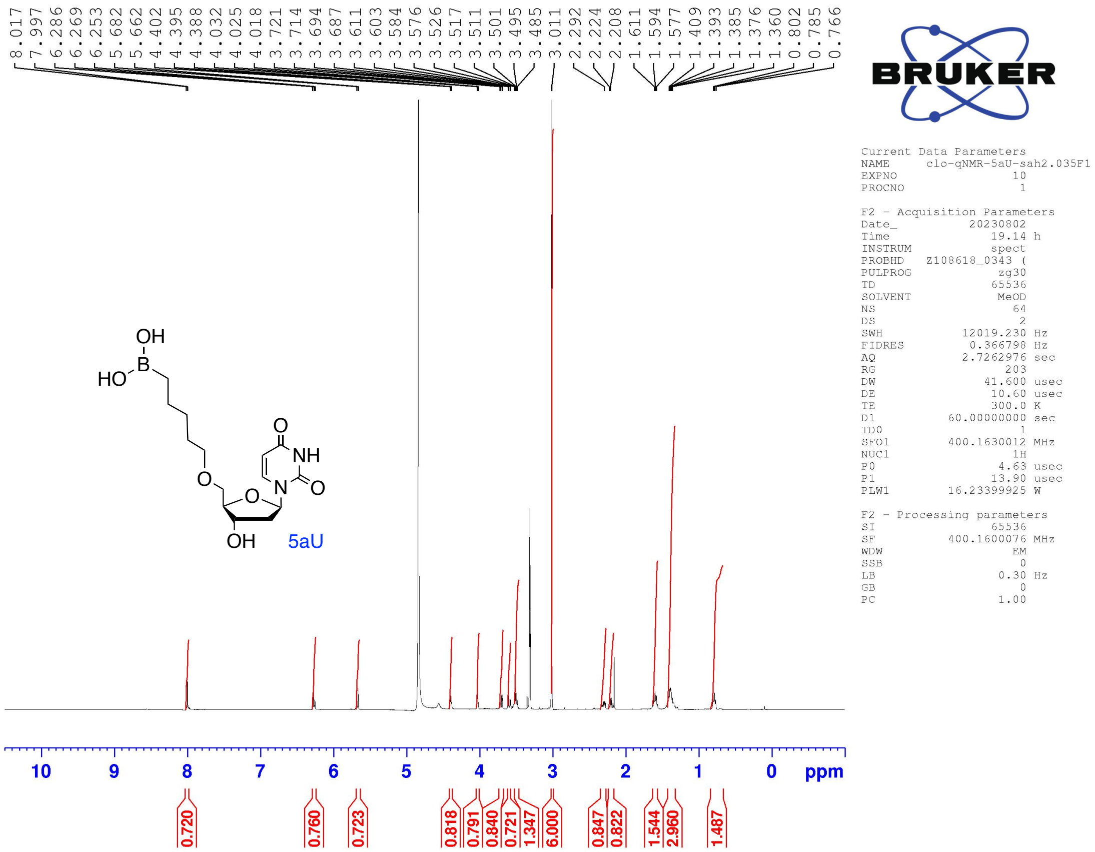

Figure S174 ^{1}H qNMR purity spectrum of **5aU** with Me_2SO_2 (I.C) in CD_3OD. Purity 96.06%.

$$P_{\text{sample}} = \frac{S_{\text{sample}} \text{ x } N_{\text{std}} \text{ x } m_{\text{std}} \text{ x } M_{\text{sample}}}{S_{\text{std}} \text{ x } N_{\text{sample}} \text{ x } m_{\text{sample}} \text{ x } M_{\text{std}}} \text{ x } P_{\text{std}}$$

$$= \frac{0.759 \text{ x } 6 \text{ x } 2.4 \text{ mg x } 342.16 \text{ g mol}^{-1}}{6 \text{ x } 1 \text{ x } 6.9 \text{ mg x } 94.13 \text{ g mol}^{-1}} \text{ x } 99.96$$

$$= 96.06\%$$

S = Integrated area of the peak
N = Number of protons represented
m = Prepared mass
M = Molecular weight
P = Purity

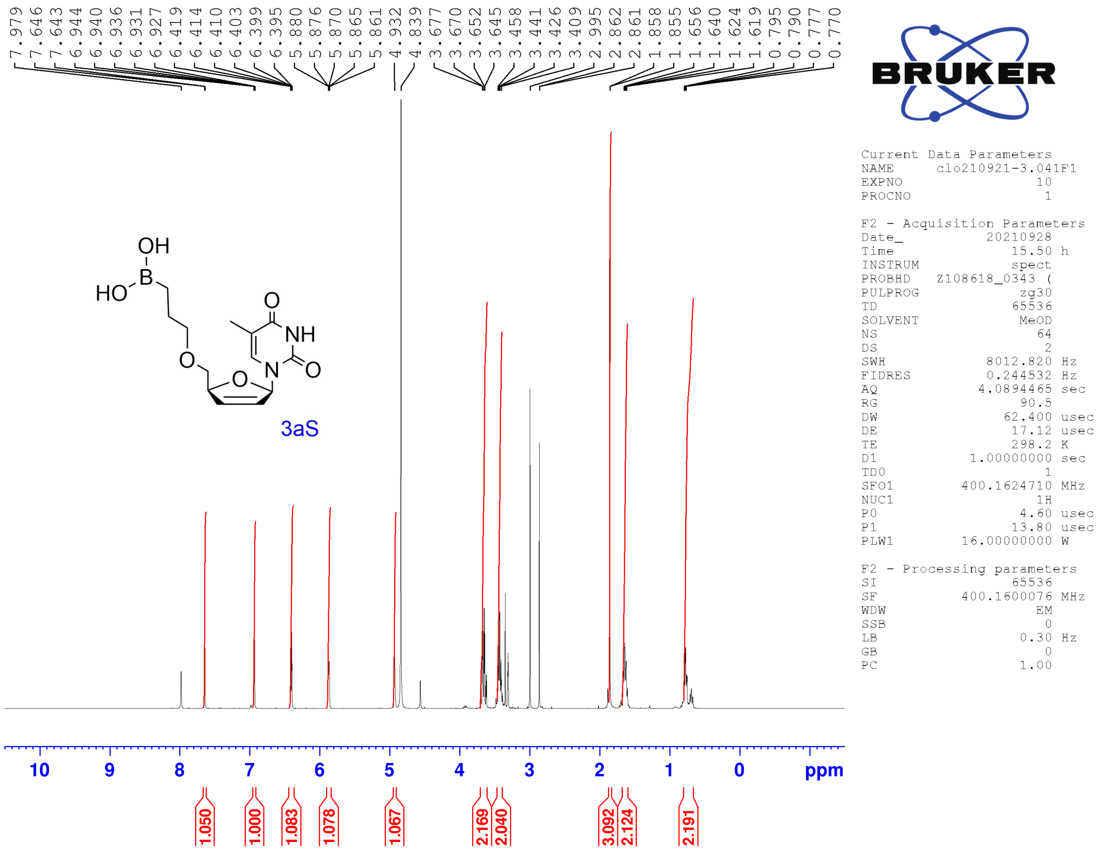

Figure S175 ^{1}HNMR spectra of **3aS** in CD_3OD.

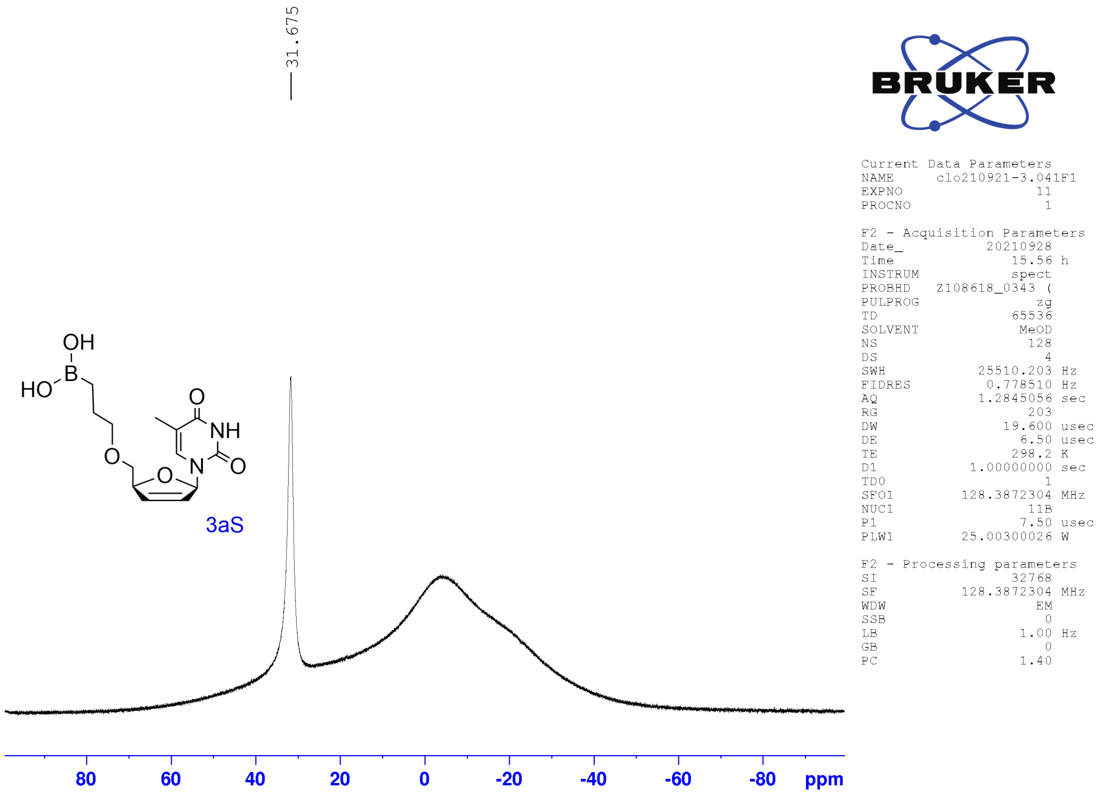

Figure S176 ^{11}BNMR spectra of **3aS** in CD_3OD.

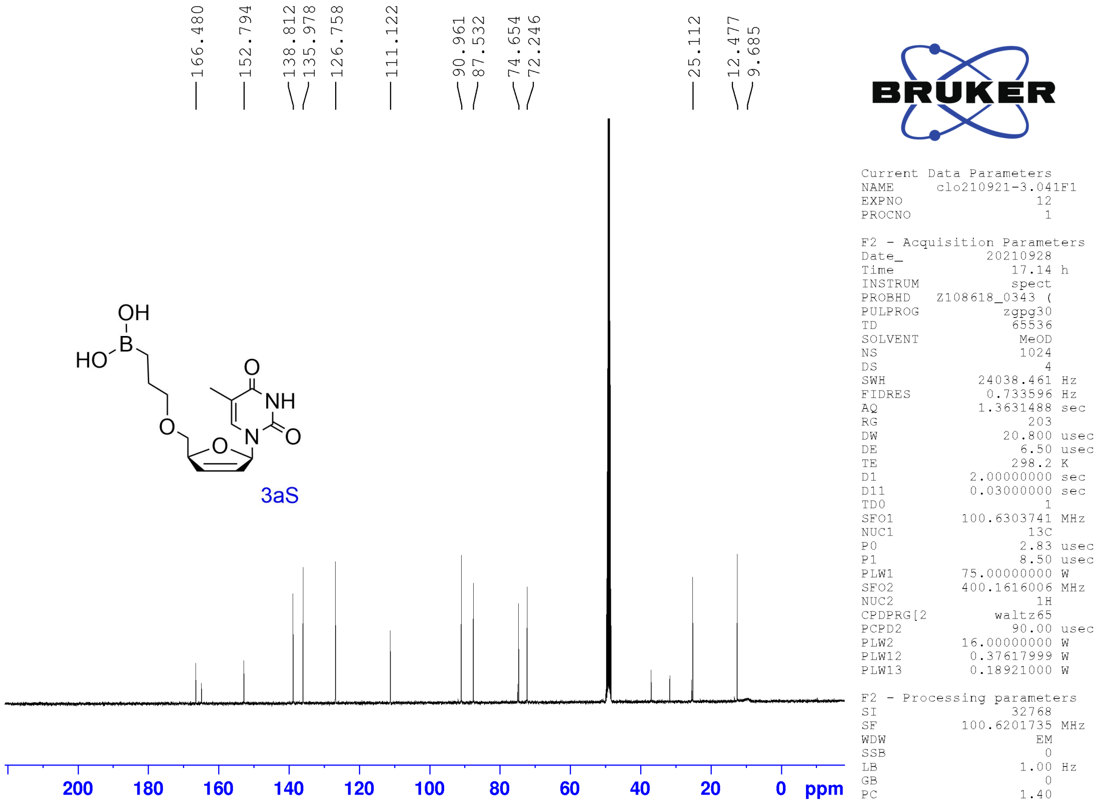

Figure S177 $^{13}C\{^{1}H\}$-NMR spectra of **3aS** in CD_3OD.

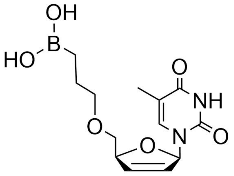

3aS

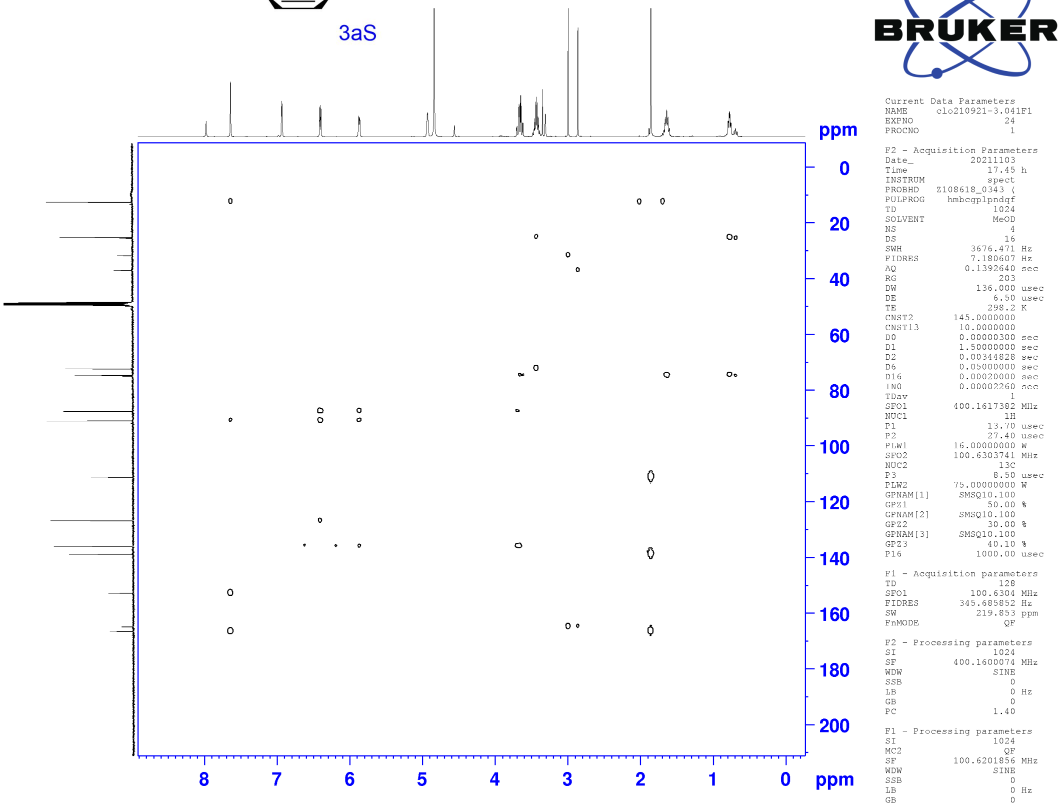

Figure S178 HMBC-NMR spectra of **3aS** in CD_3OD.

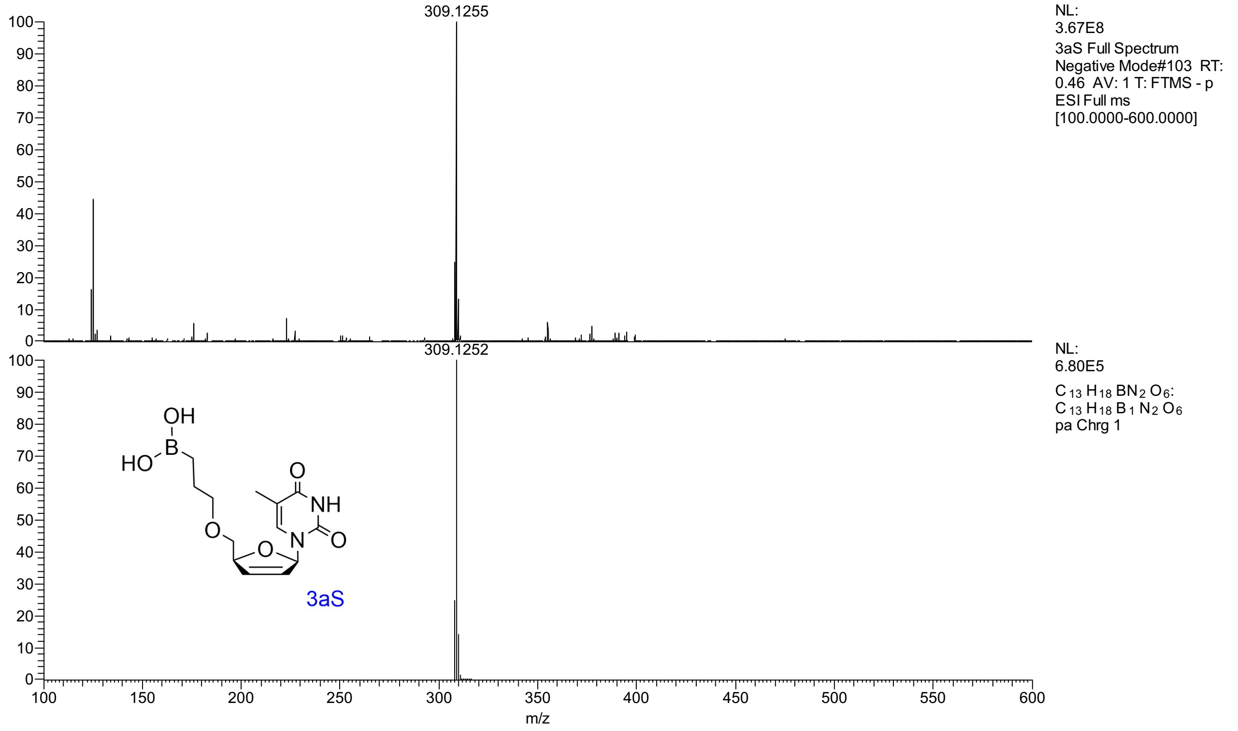

Figure S179 HRMS (ESI-) negative mode m/z calculated for **3aS** [$C_{13}H_{18}BN_2O_6$] [M-H]$^-$ 309.1252, found 309.1255.

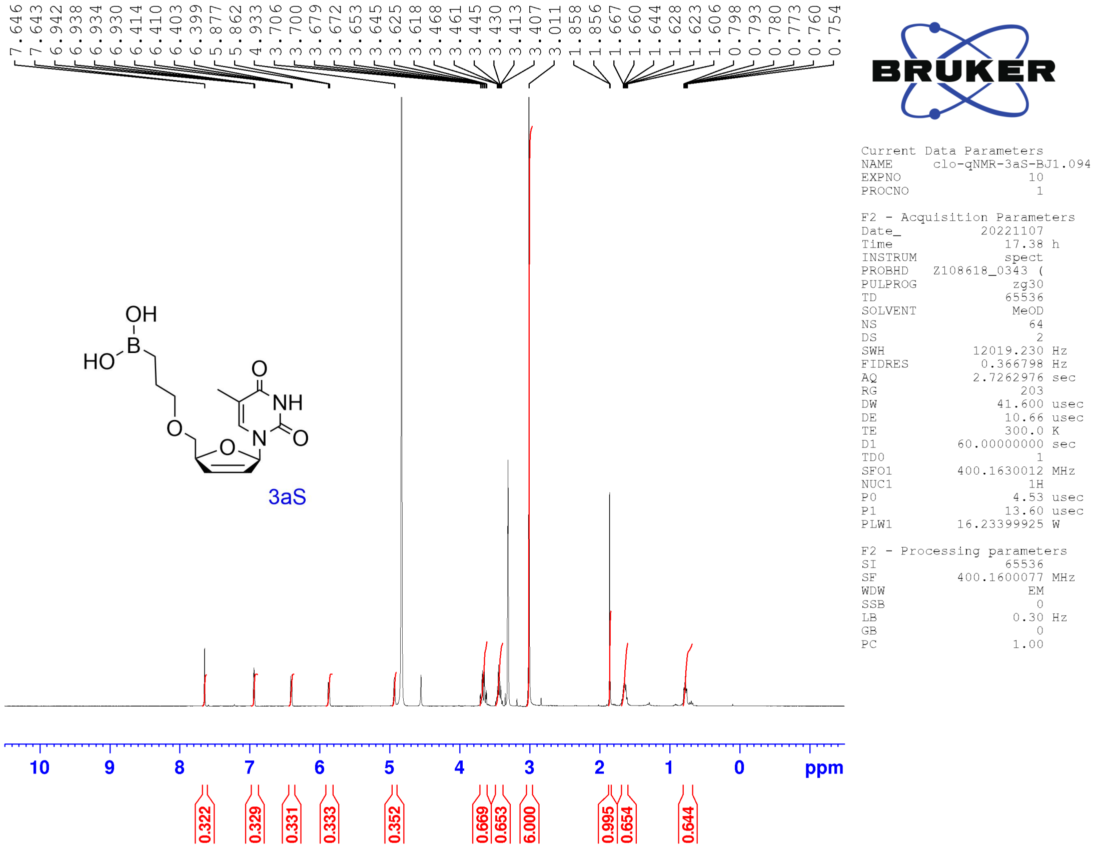

Figure S1380 ^{1}H qNMR purity spectrum of **3aS** with Me_2SO_2 (I.C) in CD_3OD. Purity 96.56%.

$$P_{\text{sample}} = \frac{S_{\text{sample}} \text{ x } N_{\text{std}} \text{ x } m_{\text{std}} \text{ x } M_{\text{sample}}}{S_{\text{std}} \text{ x } N_{\text{sample}} \text{ x } m_{\text{sample}} \text{ x } M_{\text{std}}} \text{ x } P_{\text{std}}$$

$$= \frac{0.331 \text{ x } 6 \text{ x } 3.9 \text{ mg x } 310.11 \text{ g mol}^{-1}}{6 \text{ x } 1 \text{ x } 4.4 \text{ mg x } 94.13 \text{ g mol}^{-1}} \text{ x } 99.96$$

$$= 96.56\%$$

S = Integrated area of the peak
N = Number of protons represented
m = Prepared mass
M = Molecular weight
P = Purity

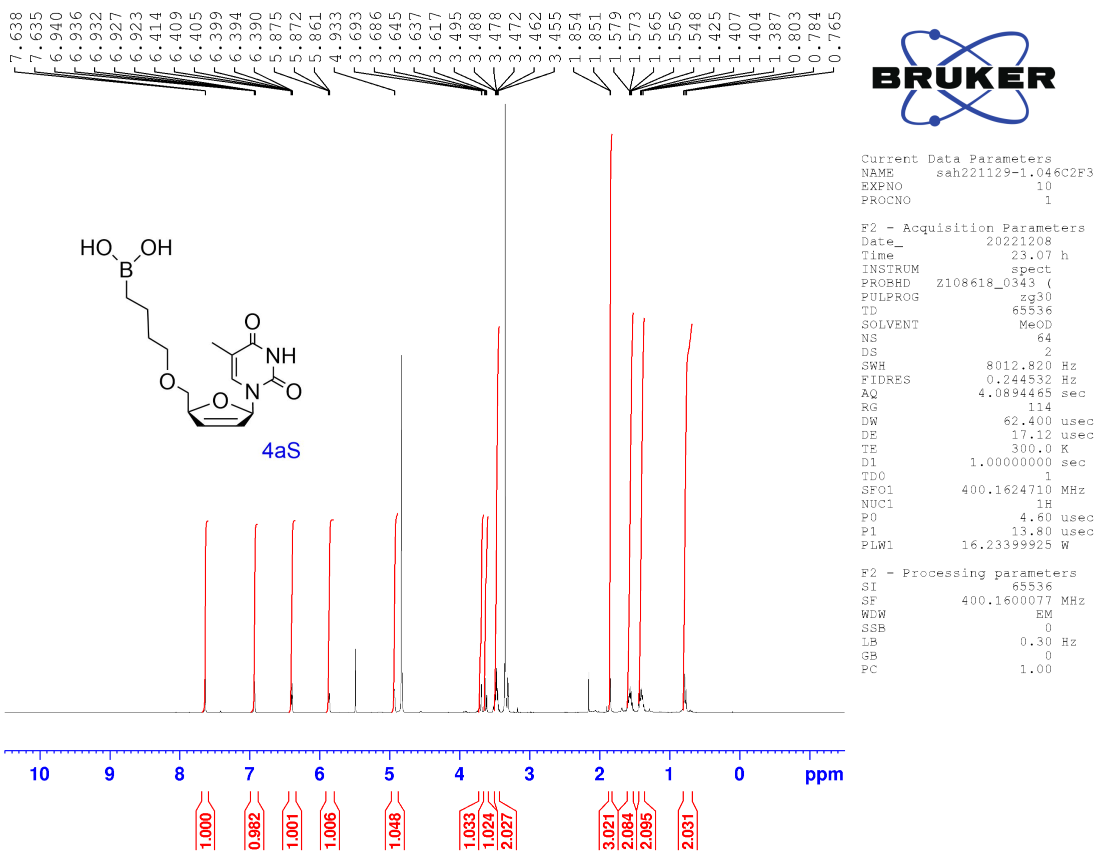

Figure S181 ^{1}HNMR spectra of **4aS** in CD_3OD.

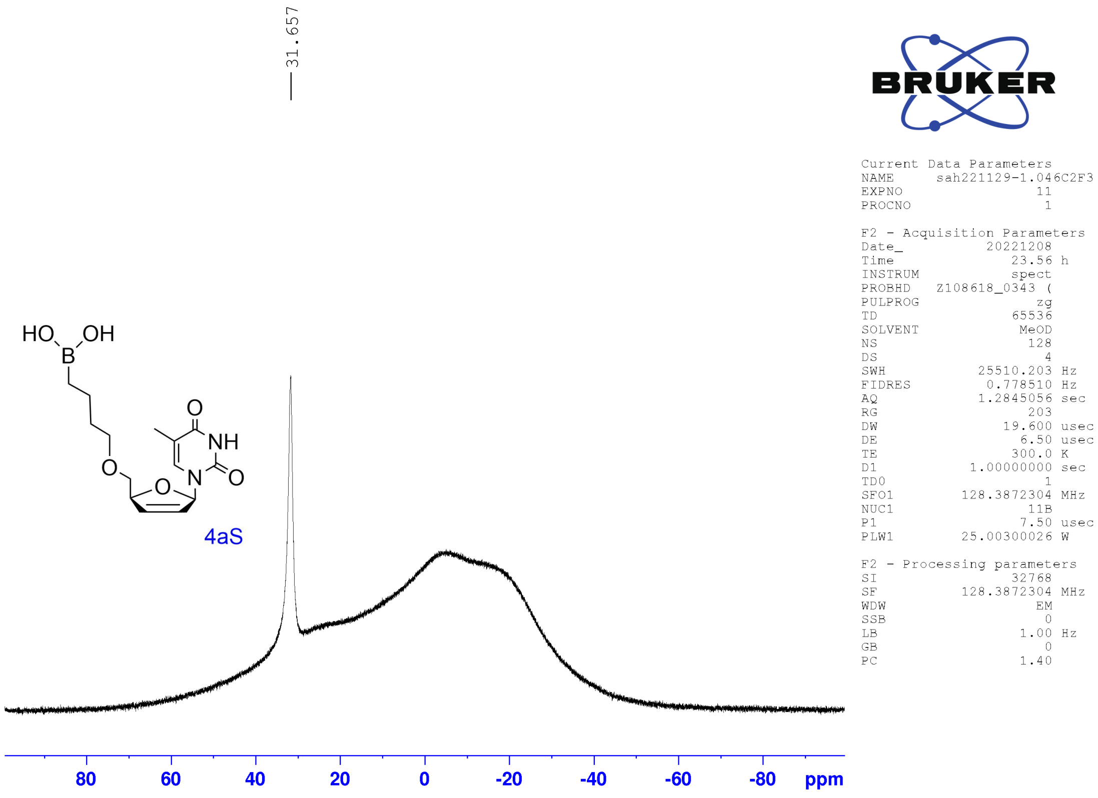

Figure S182 ^{11}BNMR spectra of **4aS** in CD_3OD.

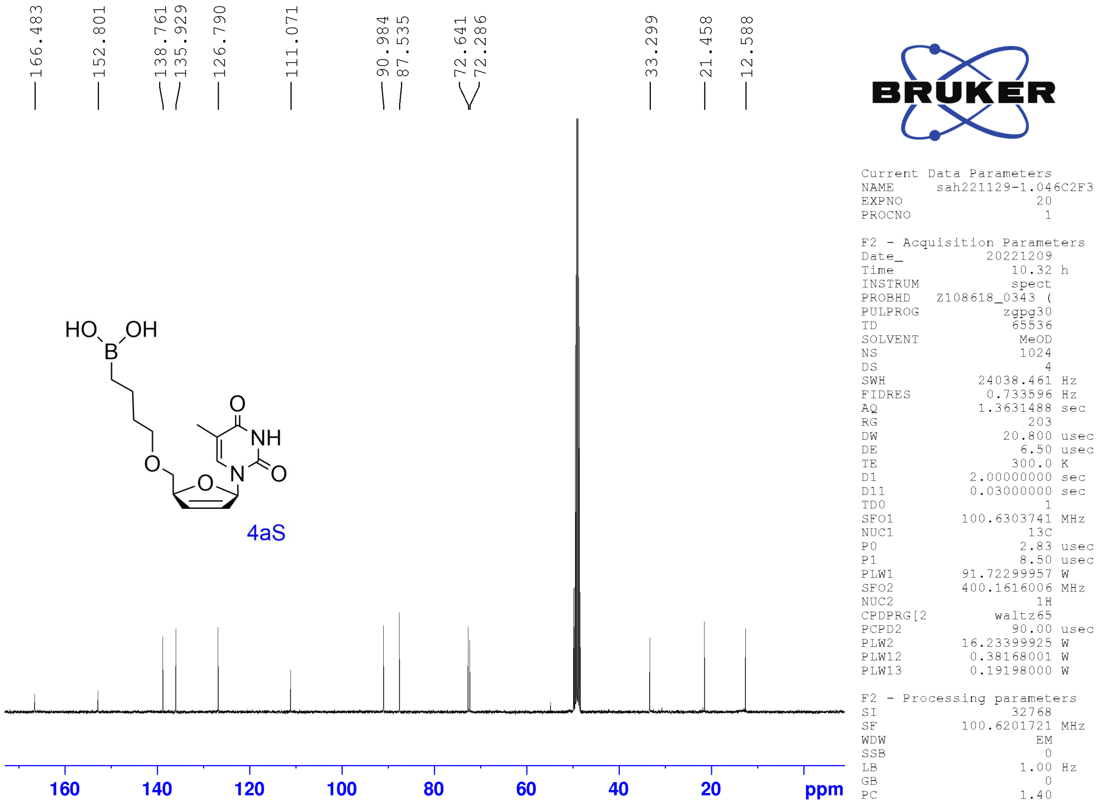

Figure S183 $^{13}C\{^{1}H\}$-NMR spectra of **4aS** in CD_3OD.

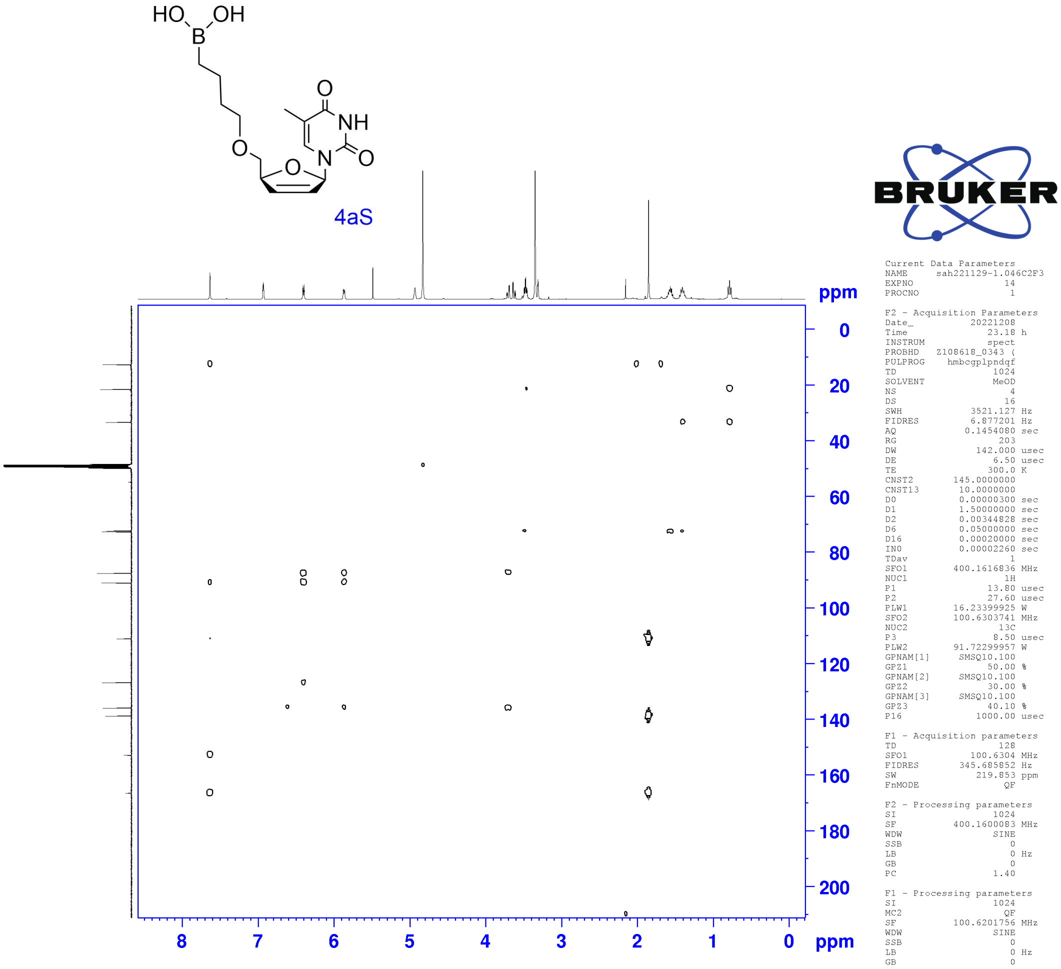

Figure S184 HMBC-NMR spectra of **4aS** in CD_3OD.

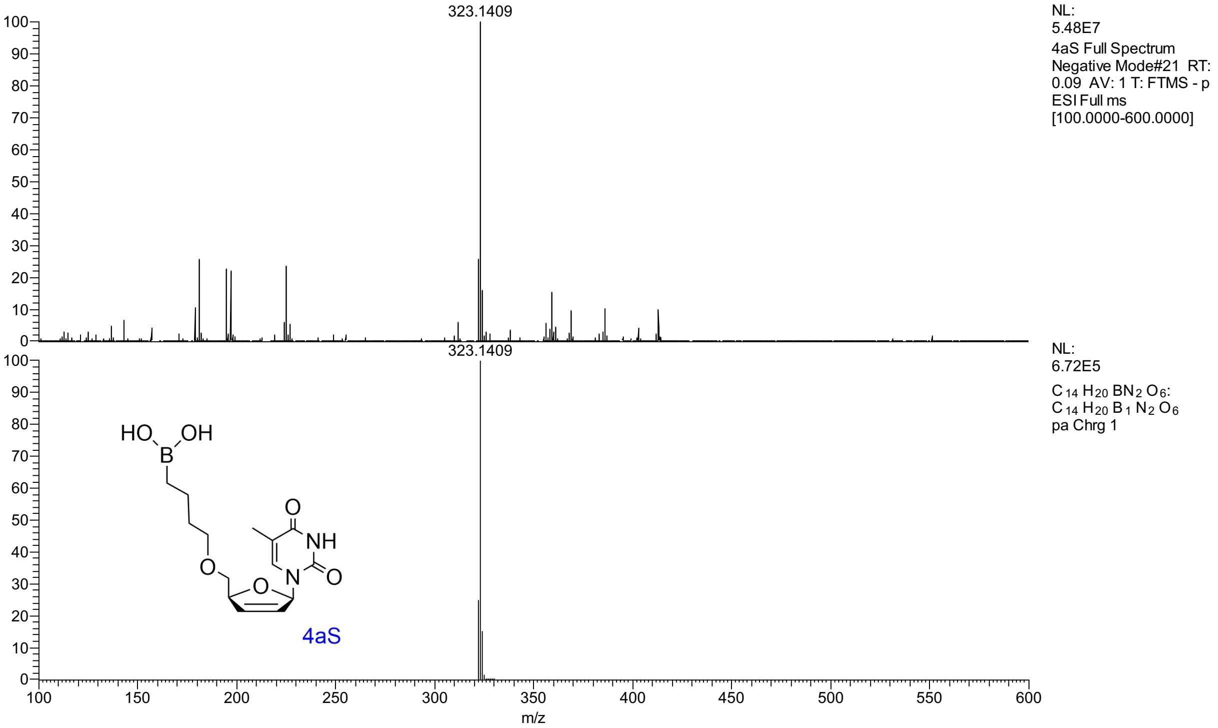

Figure S185 HRMS (ESI-) negative mode m/z calculated for **4aS** [$C_{14}H_{20}BN_2O_6$] [M-H]$^-$ 323.1409, found 323.1409.

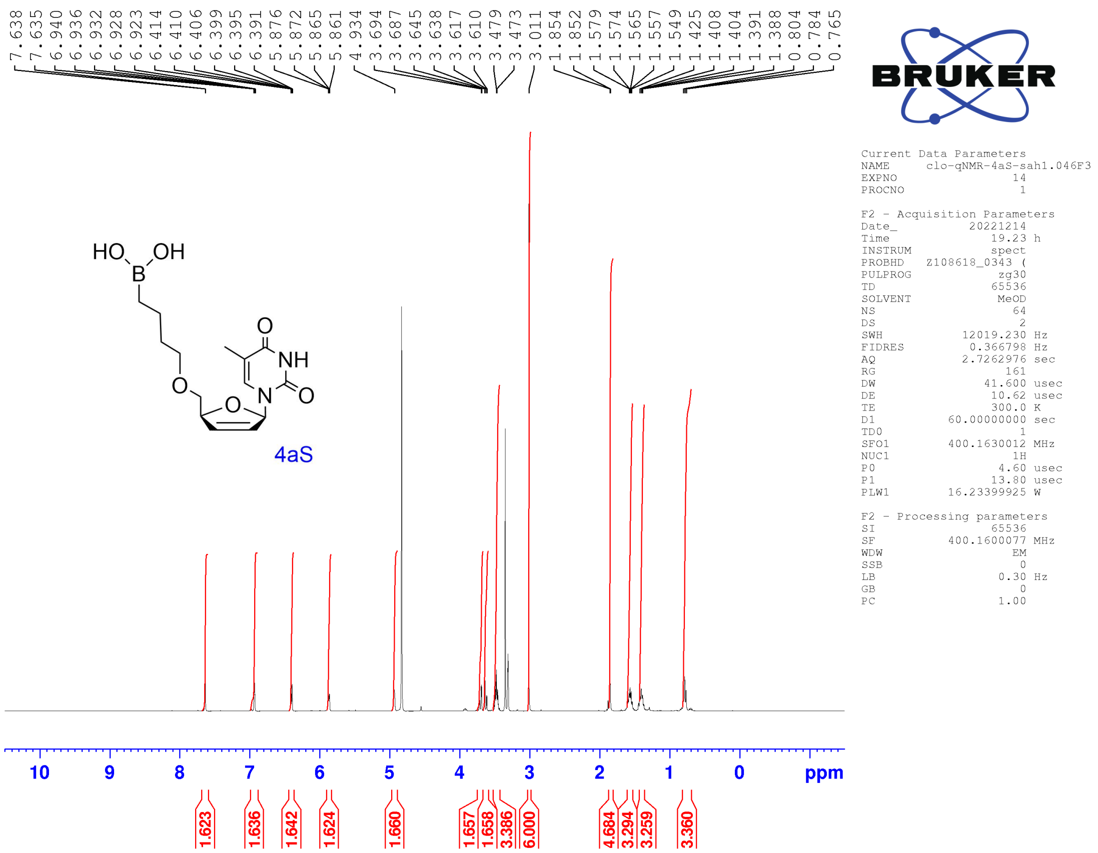

Figure S186 ^{1}H qNMR purity spectrum of **4aS** with Me_2SO_2 (I.C) in CD_3OD. Purity 95.56%.

$$P_{\text{sample}} = \frac{S_{\text{sample}} \text{ x } N_{\text{std}} \text{ x } m_{\text{std}} \text{ x } M_{\text{sample}}}{S_{\text{std}} \text{ x } N_{\text{sample}} \text{ x } m_{\text{sample}} \text{ x } M_{\text{std}}} \text{ x } P_{\text{std}}$$

$$= \frac{1.624 \text{ x } 6 \text{ x } 2.0 \text{ mg x } 324.14 \text{ g mol}^{-1}}{6 \text{ x } 1 \text{ x } 11.7 \text{ mg x } 94.13 \text{ g mol}^{-1}} \text{ x } 99.96$$

$$= 95.56\%$$

S = Integrated area of the peak
N = Number of protons represented
m = Prepared mass
M = Molecular weight
P = Purity

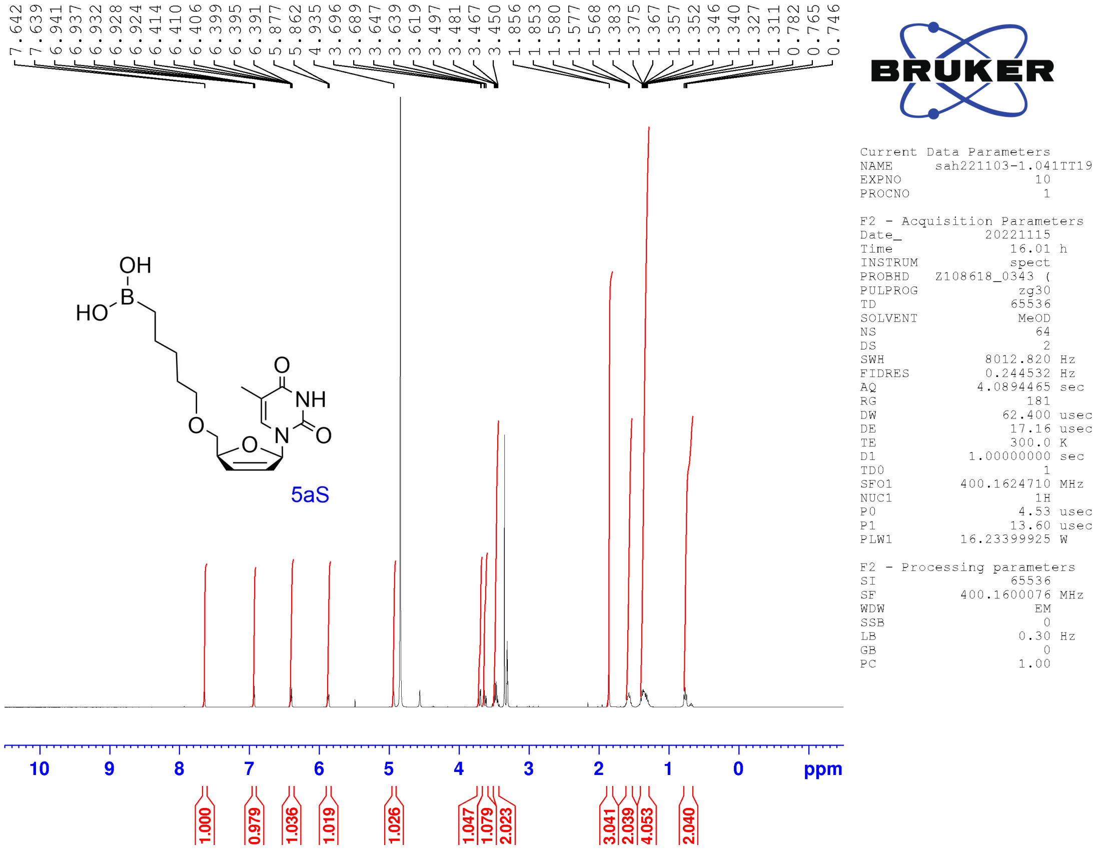

Figure S187 ^{1}HNMR spectra of **5aS** in CD_3OD.

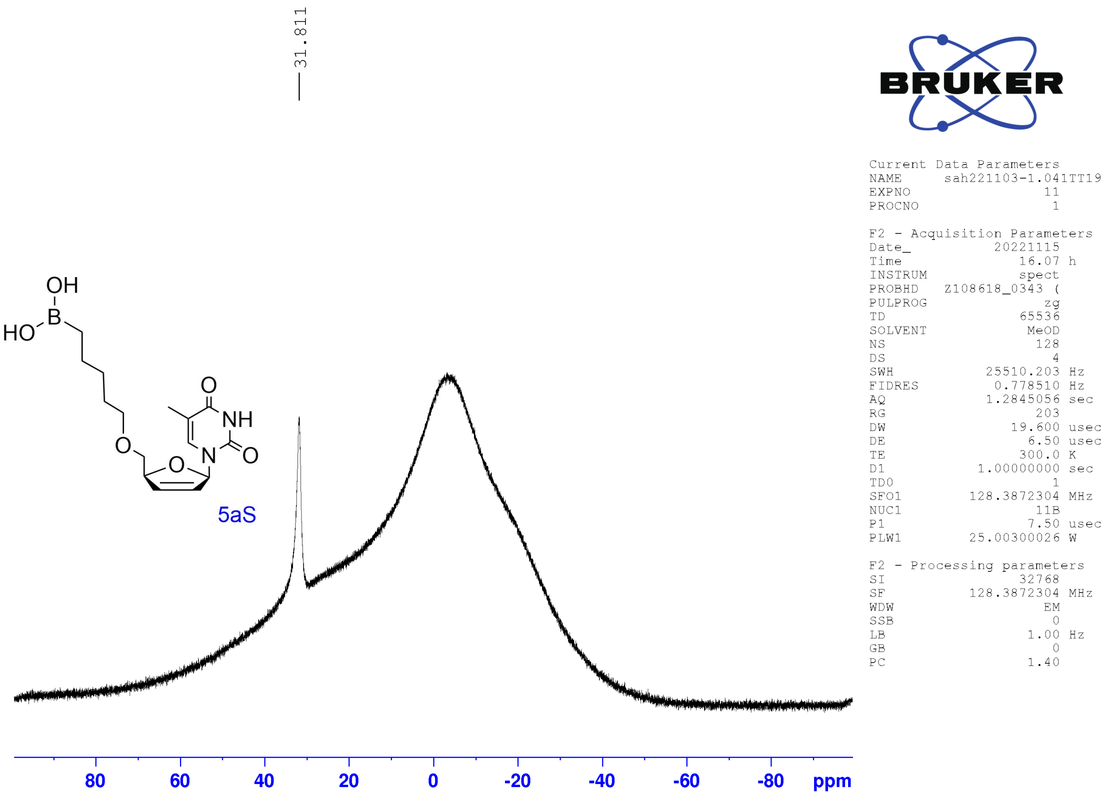

Figure S188 ^{11}BNMR spectra of **5aS** in CD_3OD.

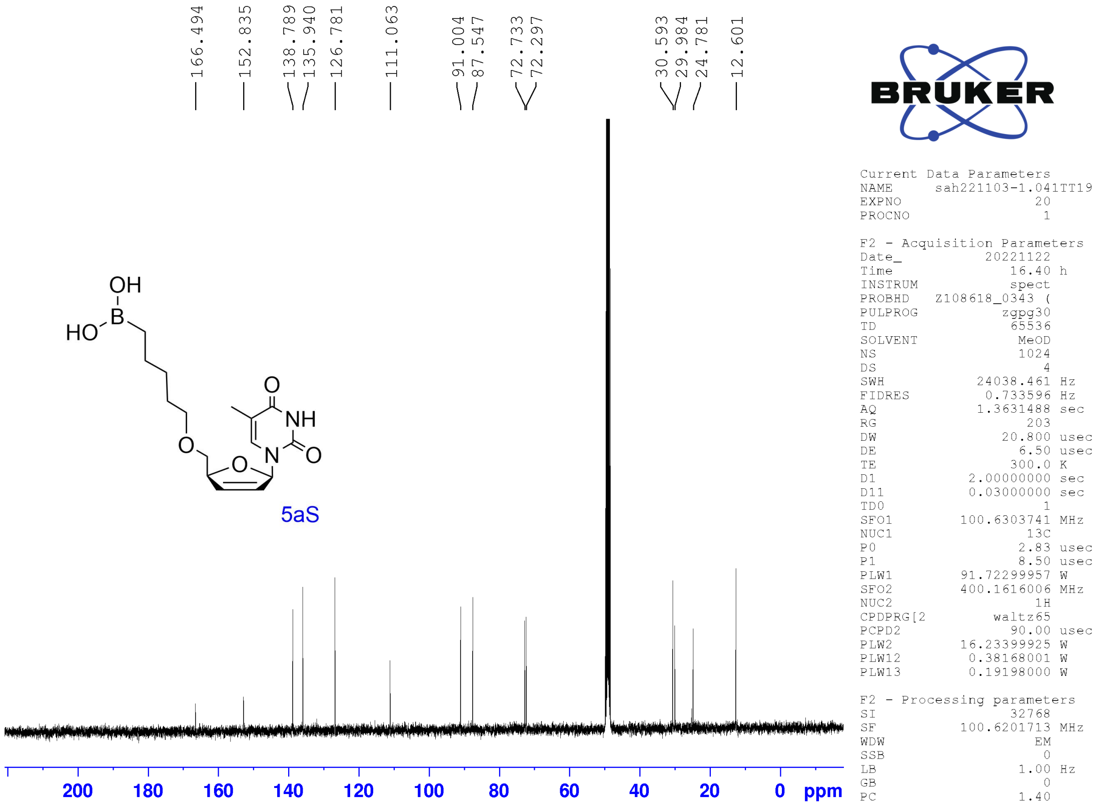

Figure S189 $^{13}C\{^{1}H\}$-NMR spectra of **5aS** in CD_3OD.

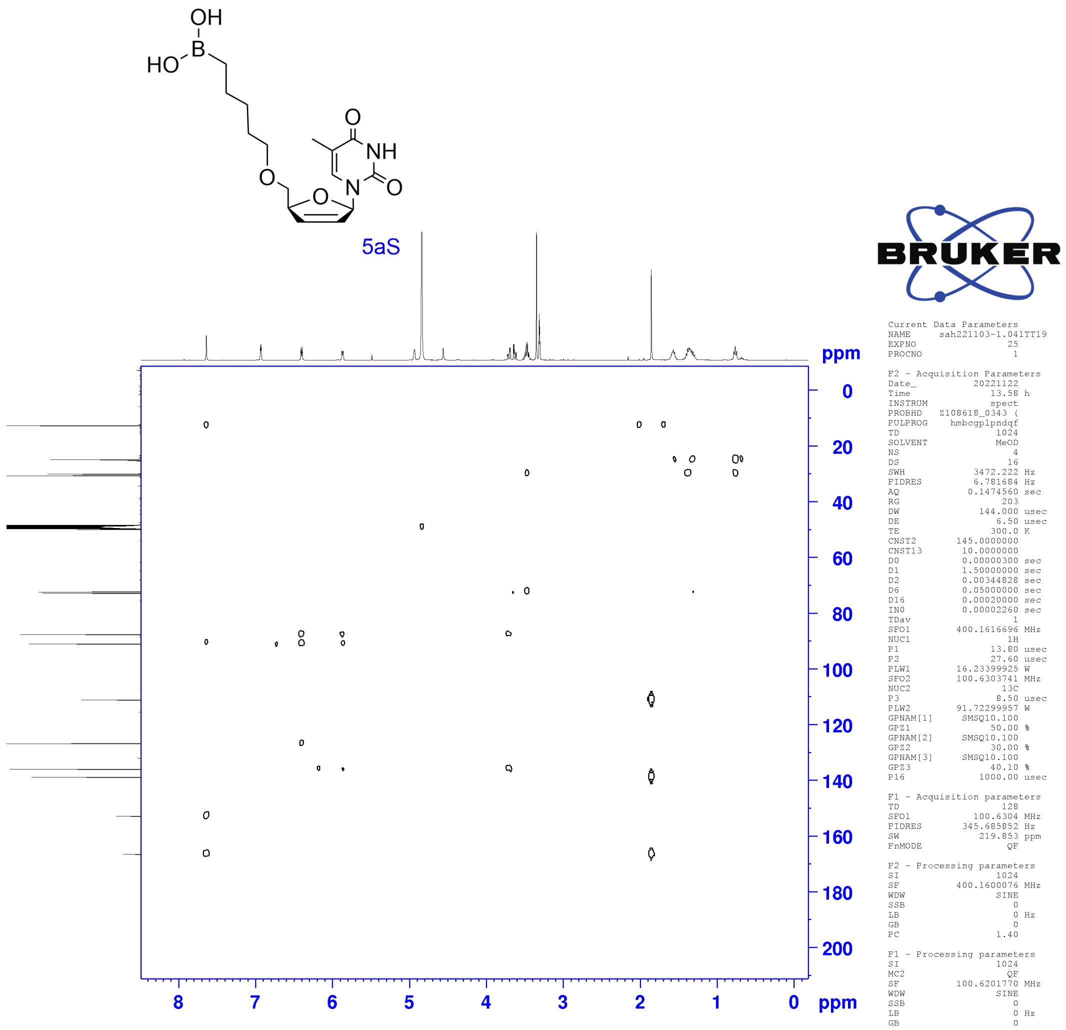

Figure S190 HMBC-NMR spectra of **5aS** in CD_3OD.

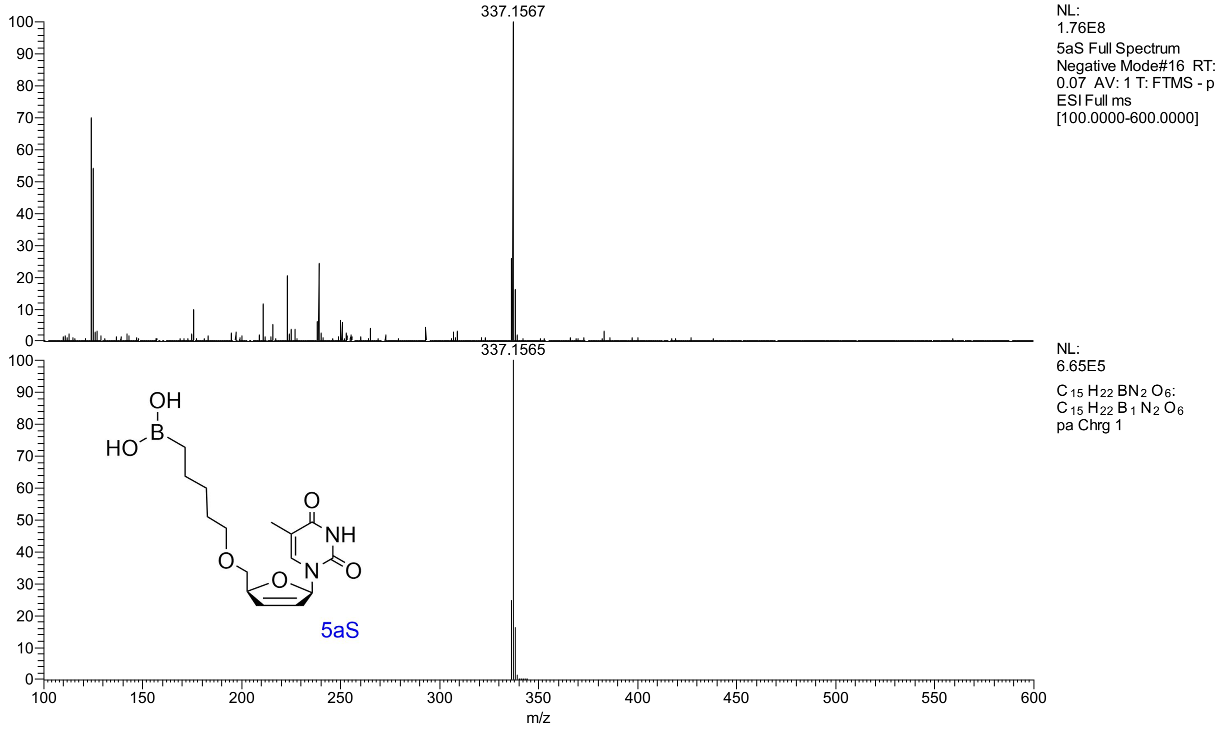

Figure S191 HRMS (ESI-) negative mode m/z calculated for **5aS** [$C_{15}H_{22}BN_2O_6$] $[M-H]^-$ 337.1565, found 337.1567.

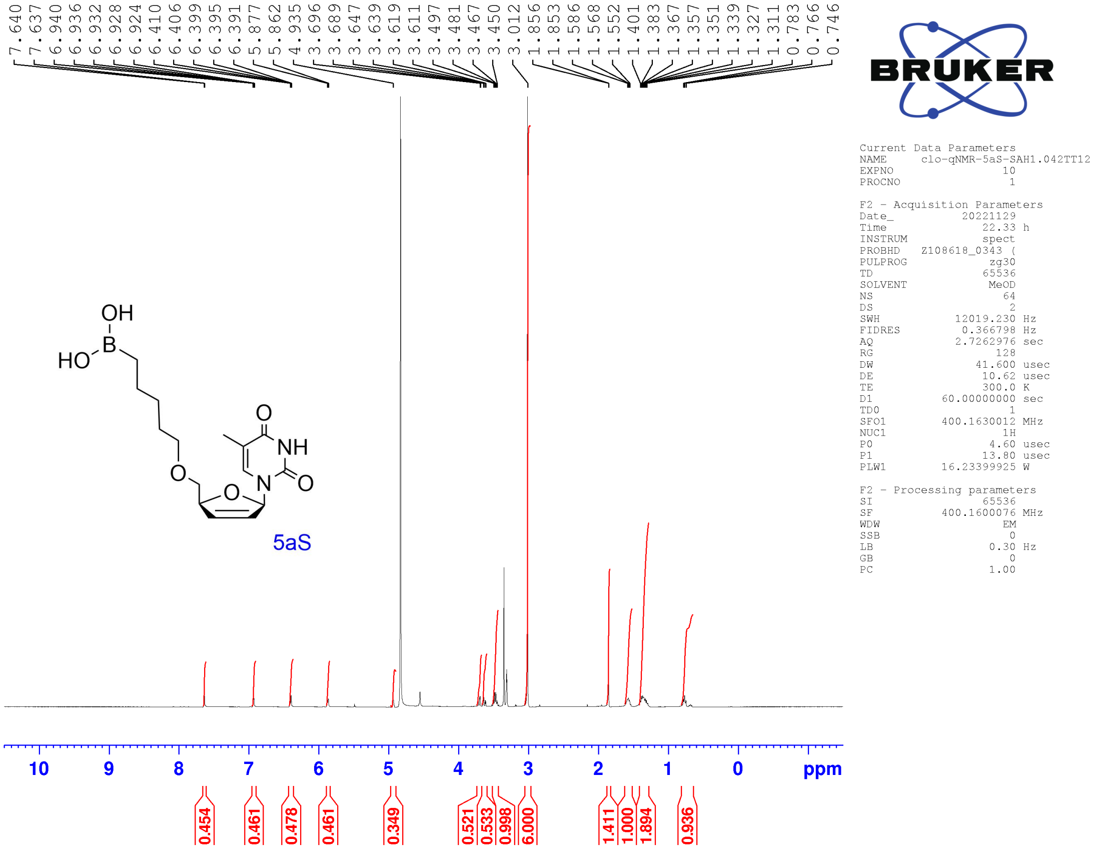

Figure S192 ^{1}H qNMR purity spectrum of **5aS** with Me_2SO_2 (I.C) in CD_3OD. Purity 96.76%.

$$P_{\text{sample}} = \frac{S_{\text{sample}} \text{ x } N_{\text{std}} \text{ x } m_{\text{std}} \text{ x } M_{\text{sample}}}{S_{\text{std}} \text{ x } N_{\text{sample}} \text{ x } m_{\text{sample}} \text{ x } M_{\text{std}}} \text{ x } P_{\text{std}}$$

$$= \frac{0.408 \text{ x } 6 \text{ x } 3.5 \text{ mg x } 338.17 \text{ g mol}^{-1}}{6 \text{ x } 1 \text{ x } 5.3 \text{ mg x } 94.13 \text{ g mol}^{-1}} \text{ x } 99.96$$

$$= 96.76\%$$

S = Integrated area of the peak
N = Number of protons represented
m = Prepared mass
M = Molecular weight
P = Purity

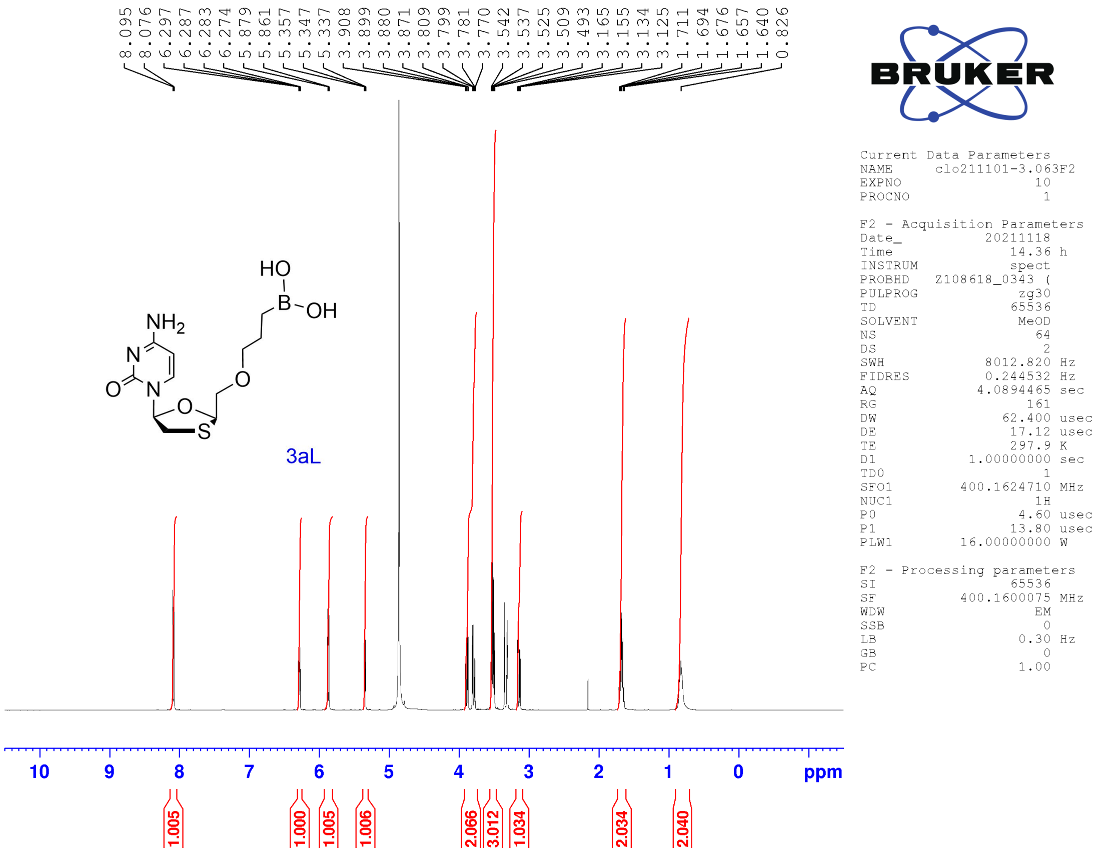

Figure S193 ^{1}HNMR spectra of **3aL** in CD_3OD.

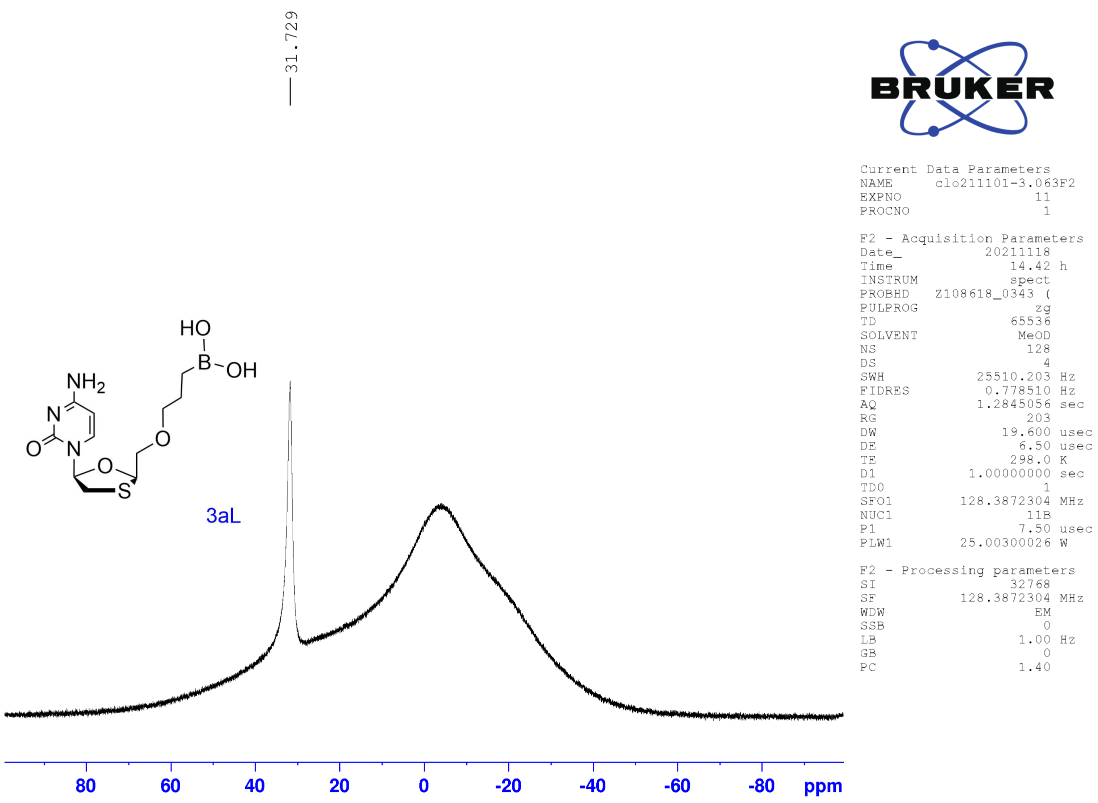

Figure S194 ^{11}BNMR spectra of **3aL** in CD_3OD.

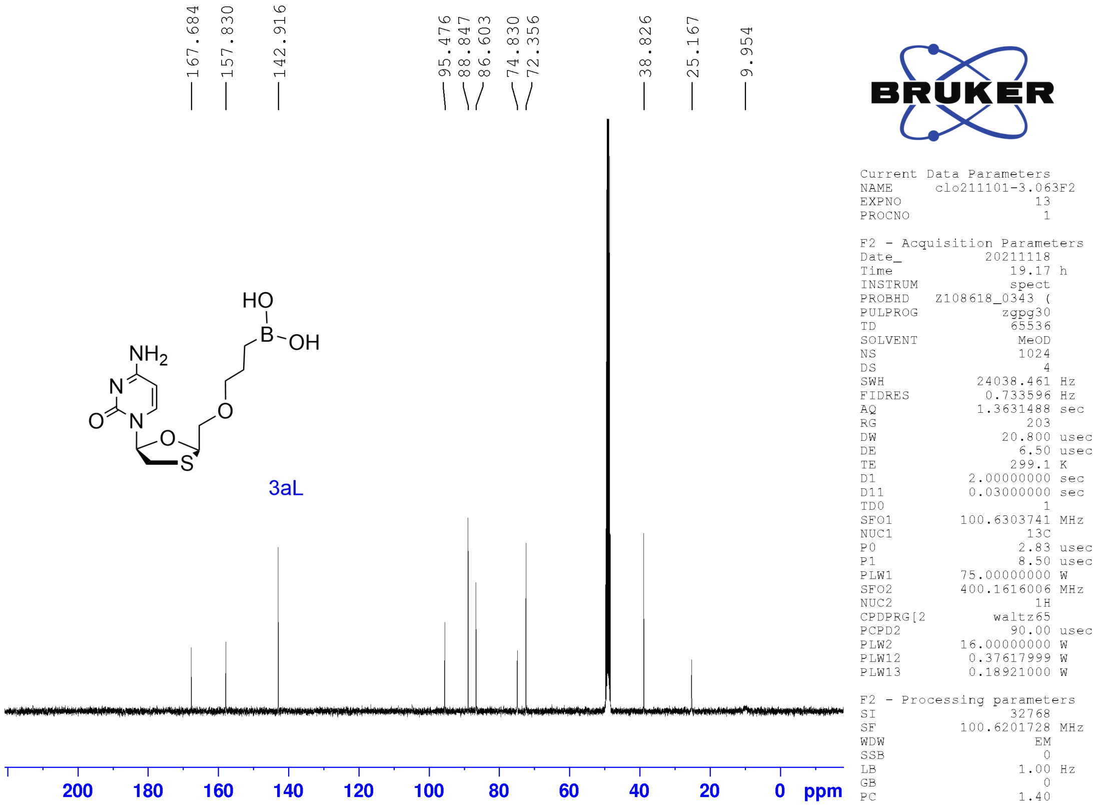

Figure S195 $^{13}C\{^{1}H\}$-NMR spectra of **3aL** in CD_3OD.

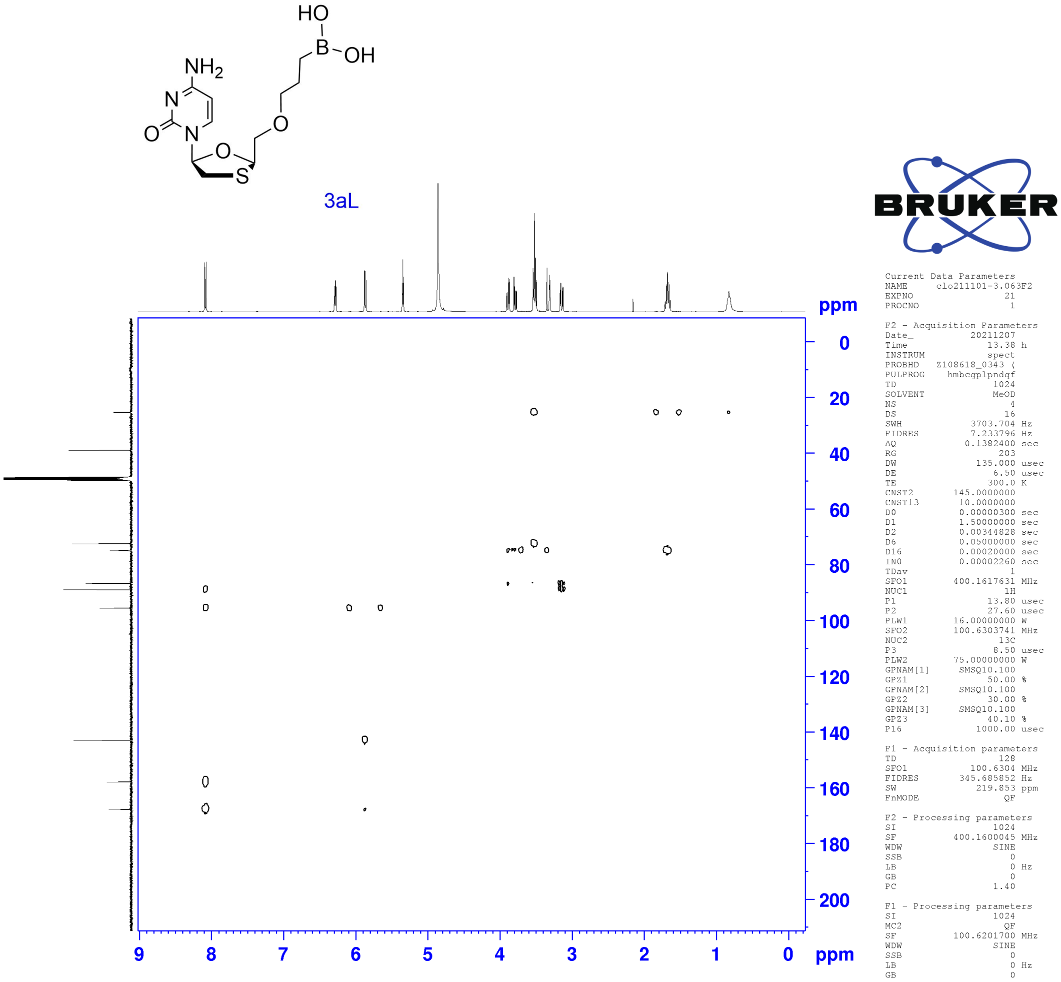

Figure S196 HMBC-NMR spectra of **3aL** in CD_3OD.

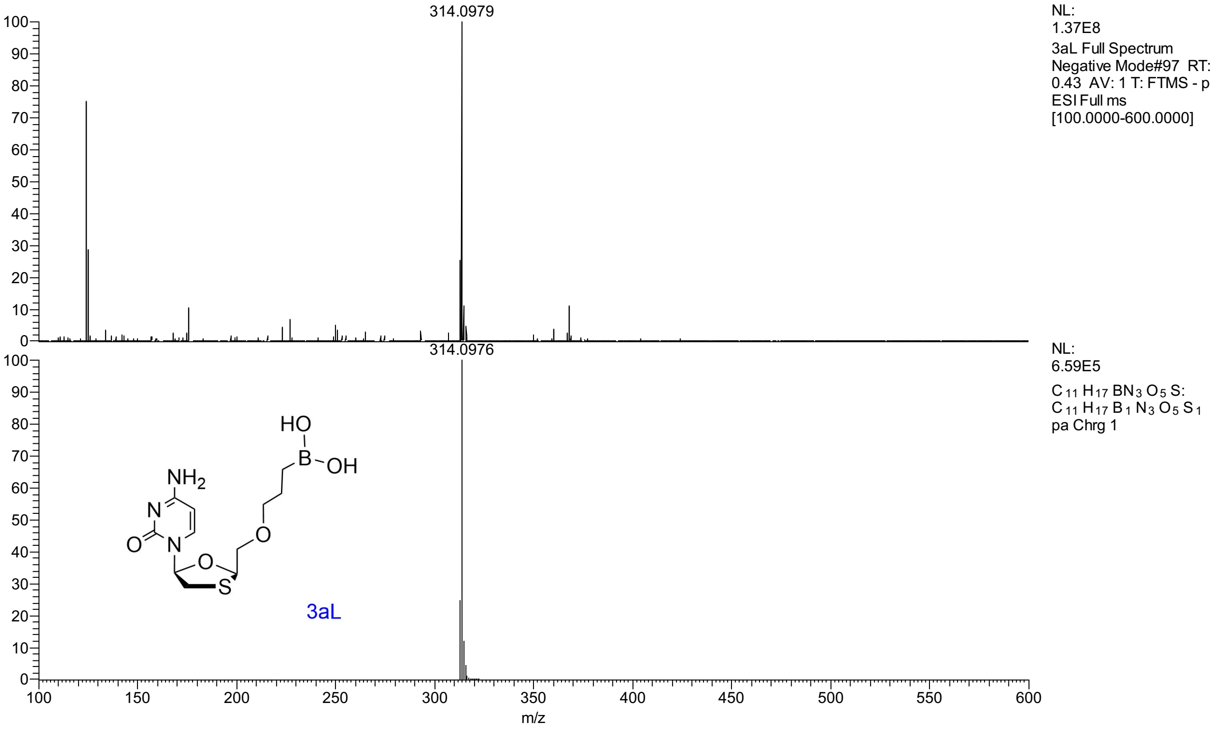

Figure S197 HRMS (ESI-) negative mode m/z calculated for **3aL** [$C_{11}H_{17}BN_3O_5S$] $[M-H]^-$ 314.0976, found 314.0979.

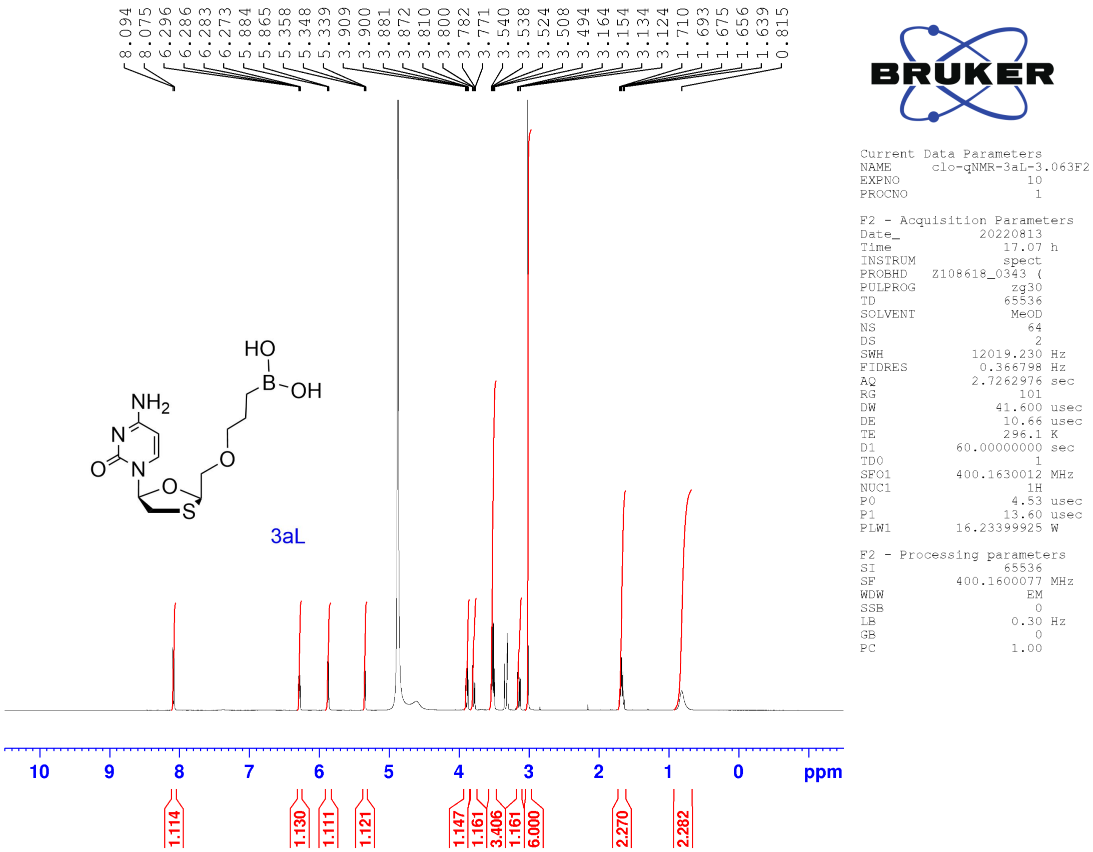

Figure S198 ^{1}H qNMR purity spectrum of **3aL** with Me_2SO_2 (I.C) in CD_3OD. Purity 97.07%.

$$P_{\text{sample}} = \frac{S_{\text{sample}} \text{ x } N_{\text{std}} \text{ x } m_{\text{std}} \text{ x } M_{\text{sample}}}{S_{\text{std}} \text{ x } N_{\text{sample}} \text{ x } m_{\text{sample}} \text{ x } M_{\text{std}}} \text{ x } P_{\text{std}}$$

$$= \frac{1.121 \text{ x } 6 \text{ x } 3.7 \text{ mg x } 315.15 \text{ g mol}^{-1}}{6 \text{ x } 1 \text{ x } 14.3 \text{ mg x } 94.13 \text{ g mol}^{-1}} \text{ x } 99.96$$

$$= 97.07\%$$

S = Integrated area of the peak
N = Number of protons represented
m = Prepared mass
M = Molecular weight
P = Purity

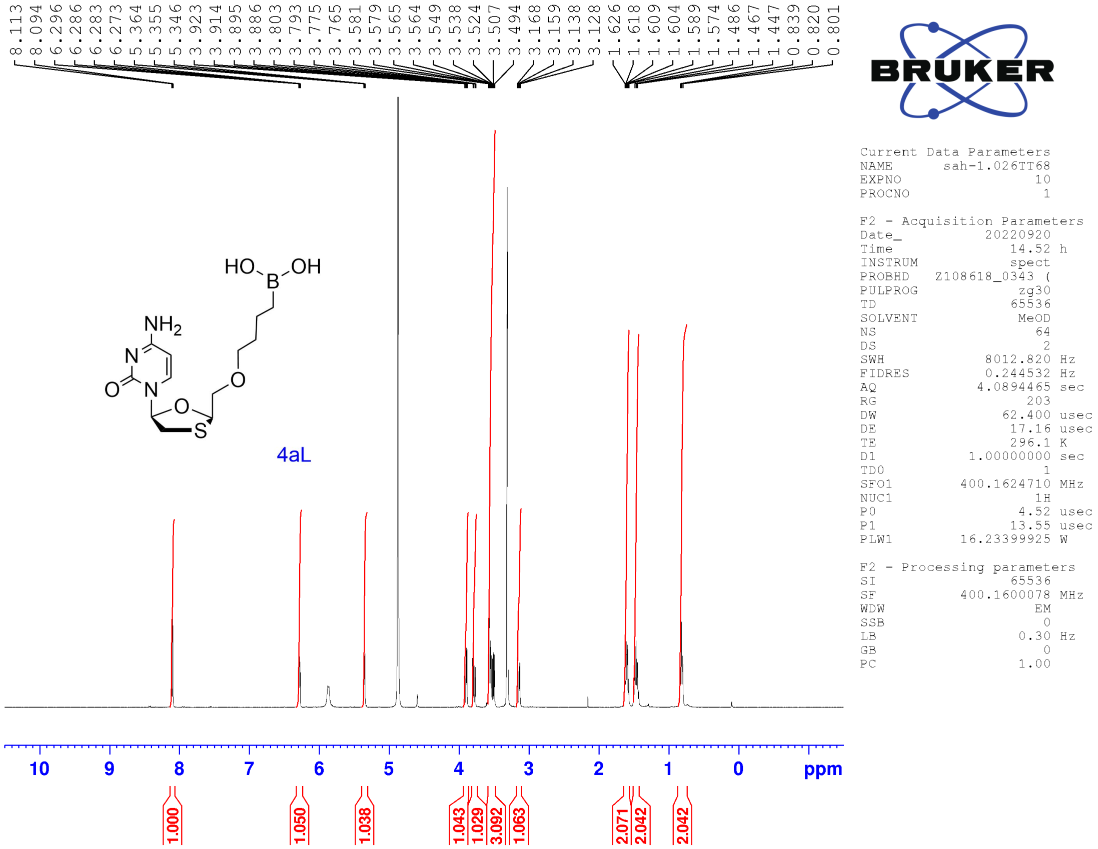

Figure S199 ^{1}HNMR spectra of **4aL** in CD_3OD.

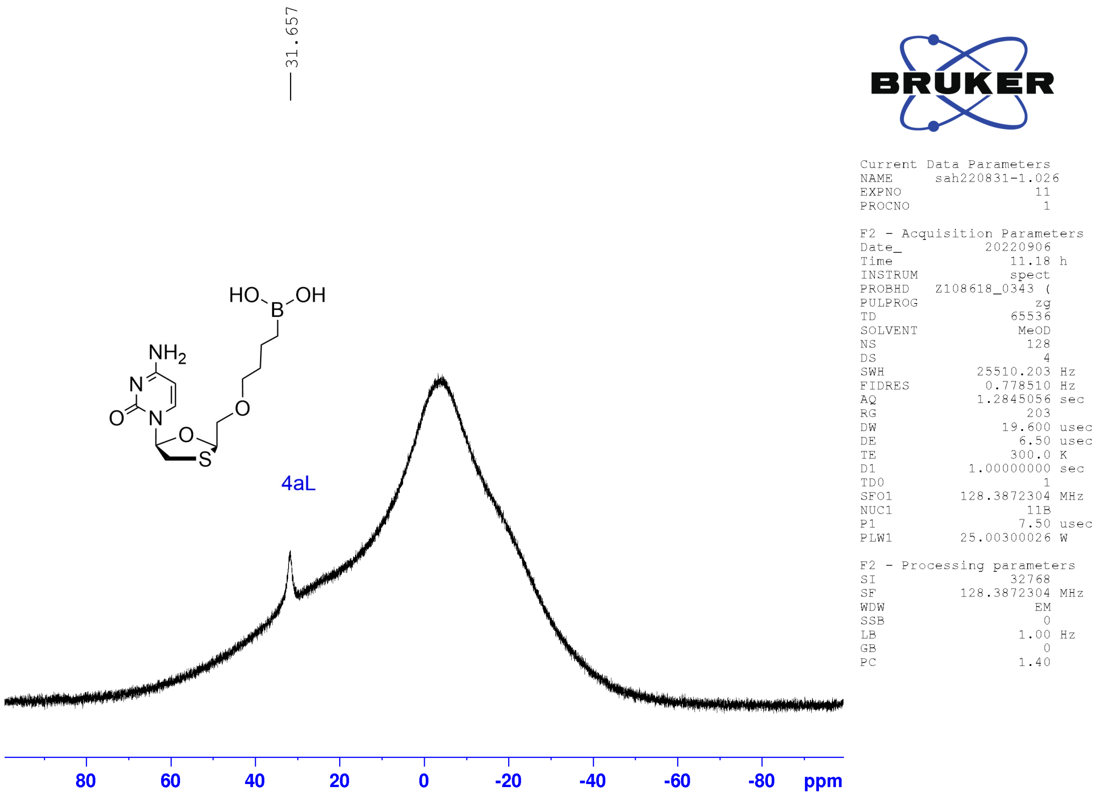

Figure S200 ^{11}BNMR spectra of **4aL** in CD_3OD.

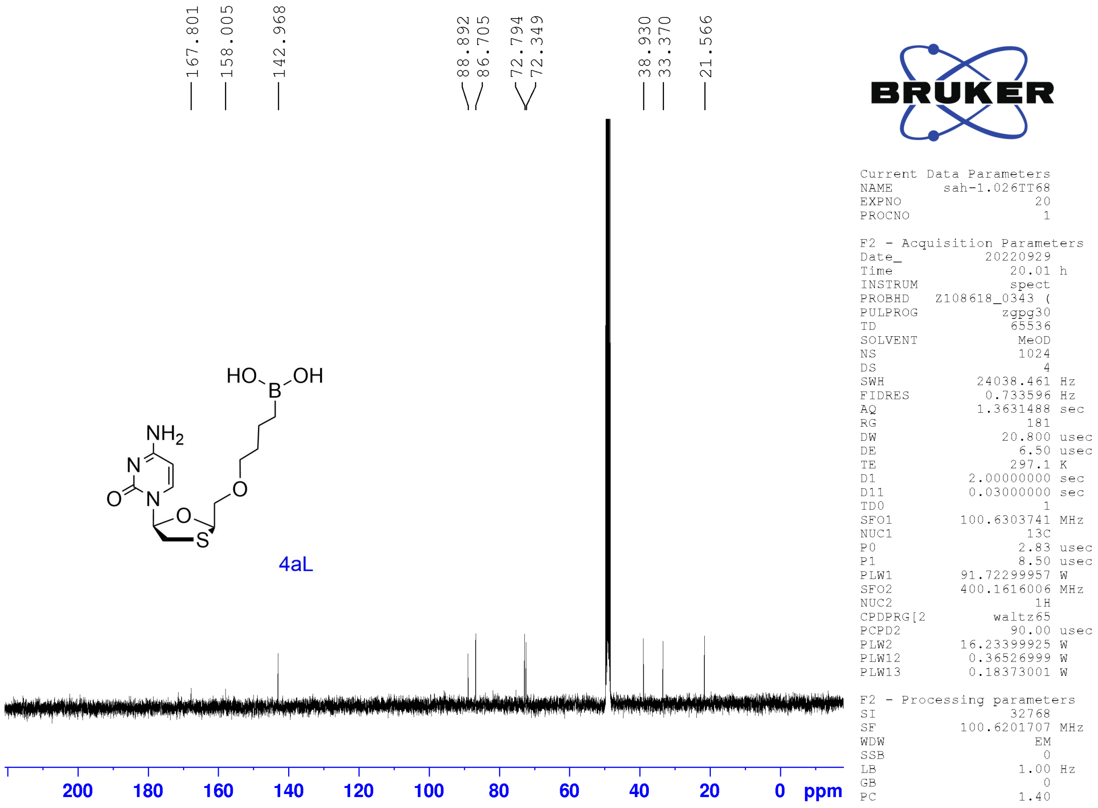

Figure S201 $^{13}C\{^{1}H\}$-NMR spectra of **4aL** in CD_3OD.

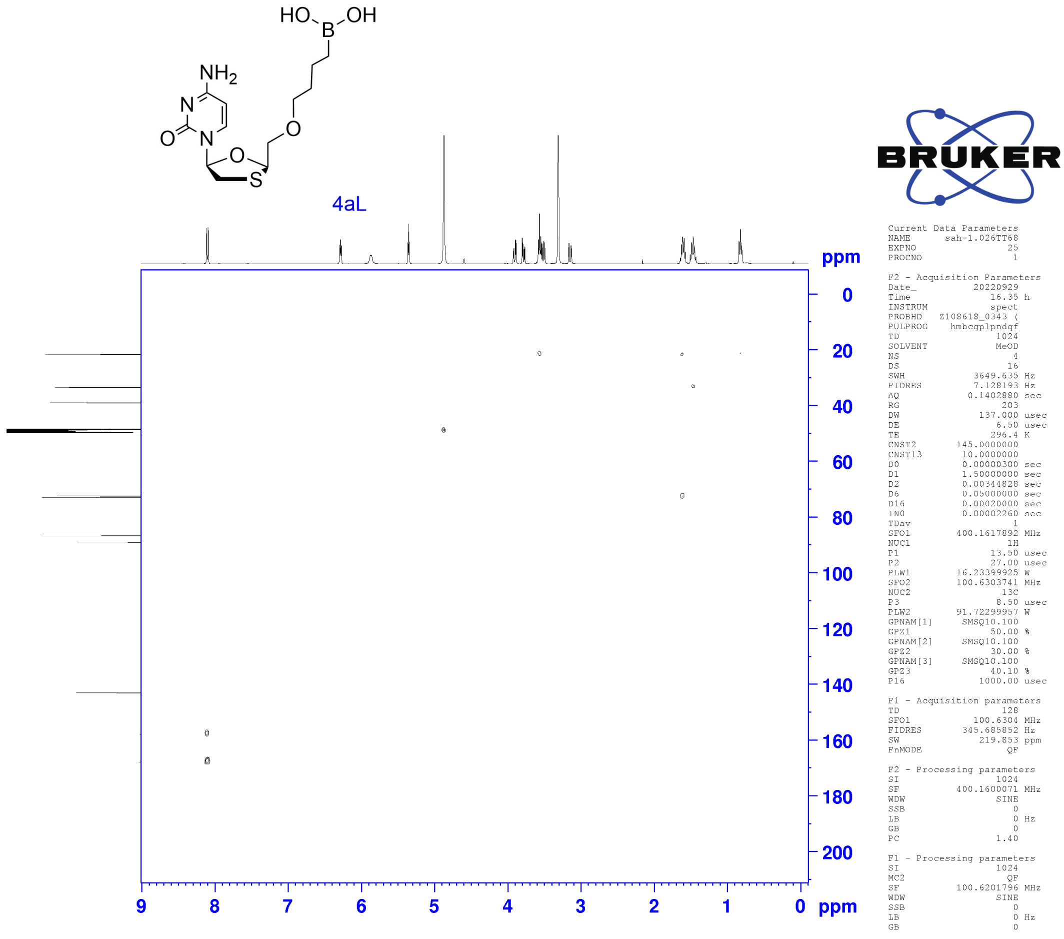

Figure S202 HMBC-NMR spectra of **4aL** in CD_3OD.

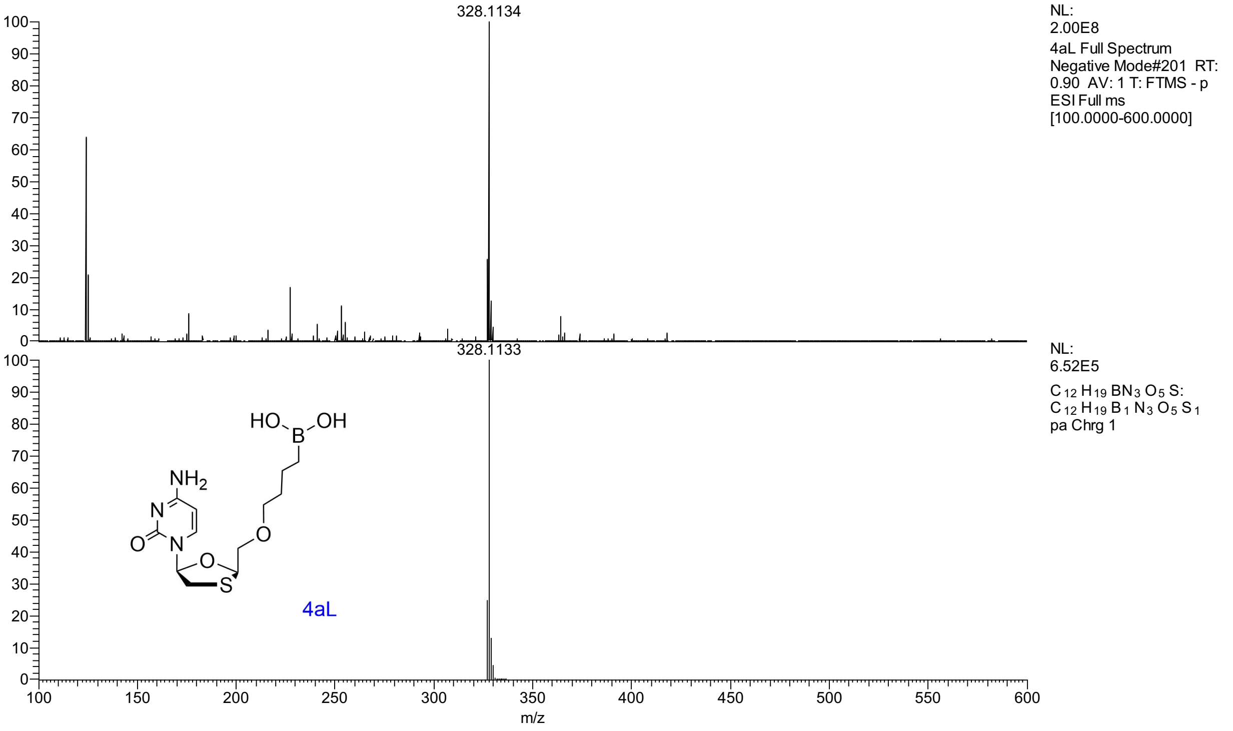

Figure S203 HRMS (ESI-) negative mode m/z calculated for **4aL** [$C_{12}H_{19}BN_3O_5S$] [M-H]$^-$ 328.1133, found 328.1134.

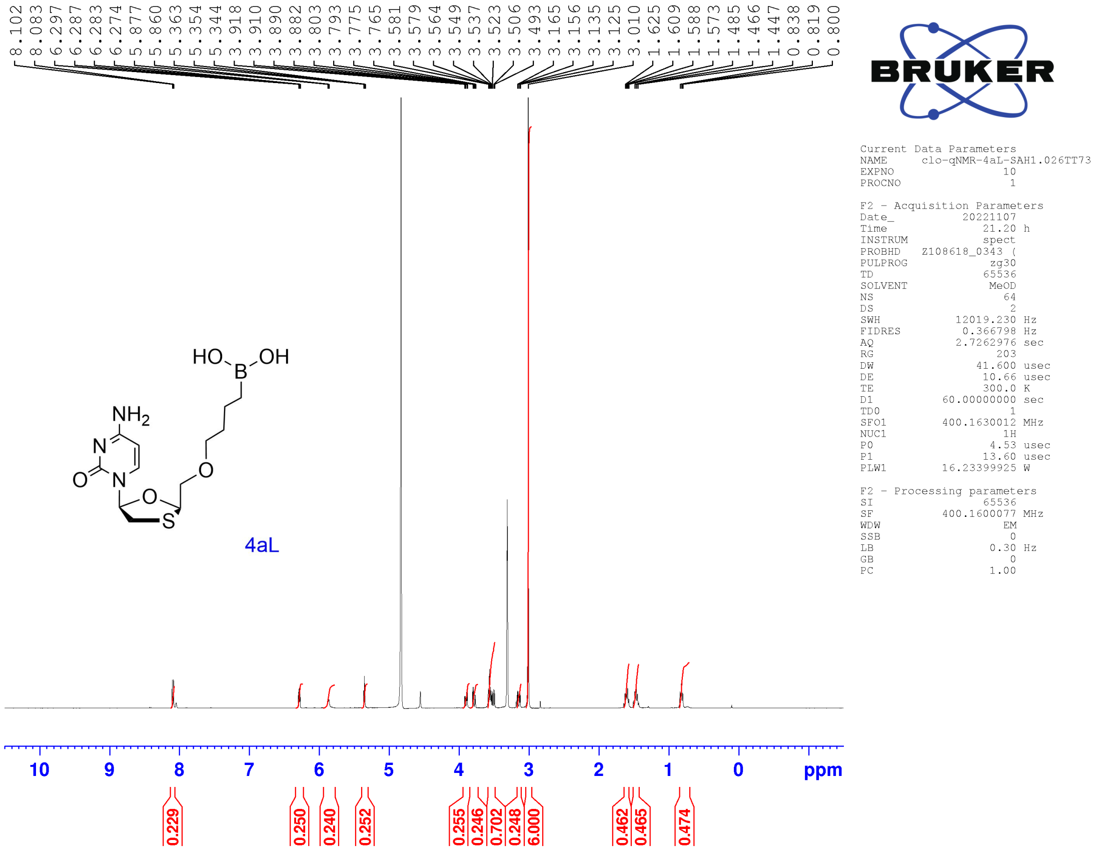

Figure S204 ^{1}H qNMR purity spectrum of **4aL** with Me_2SO_2 (I.C) in CD_3OD. Purity 96.11%.

$$P_{\text{sample}} = \frac{S_{\text{sample}} \text{ x } N_{\text{std}} \text{ x } m_{\text{std}} \text{ x } M_{\text{sample}}}{S_{\text{std}} \text{ x } N_{\text{sample}} \text{ x } m_{\text{sample}} \text{ x } M_{\text{std}}} \text{ x } P_{\text{std}}$$

$$= \frac{0.250 \text{ x } 6 \text{ x } 3.3 \text{ mg x } 329.12 \text{ g mol}^{-1}}{6 \text{ x } 1 \text{ x } 3.0 \text{ mg x } 94.13 \text{ g mol}^{-1}} \text{ x } 99.96$$

$$= 96.11\%$$

S = Integrated area of the peak
N = Number of protons represented
m = Prepared mass
M = Molecular weight
P = Purity

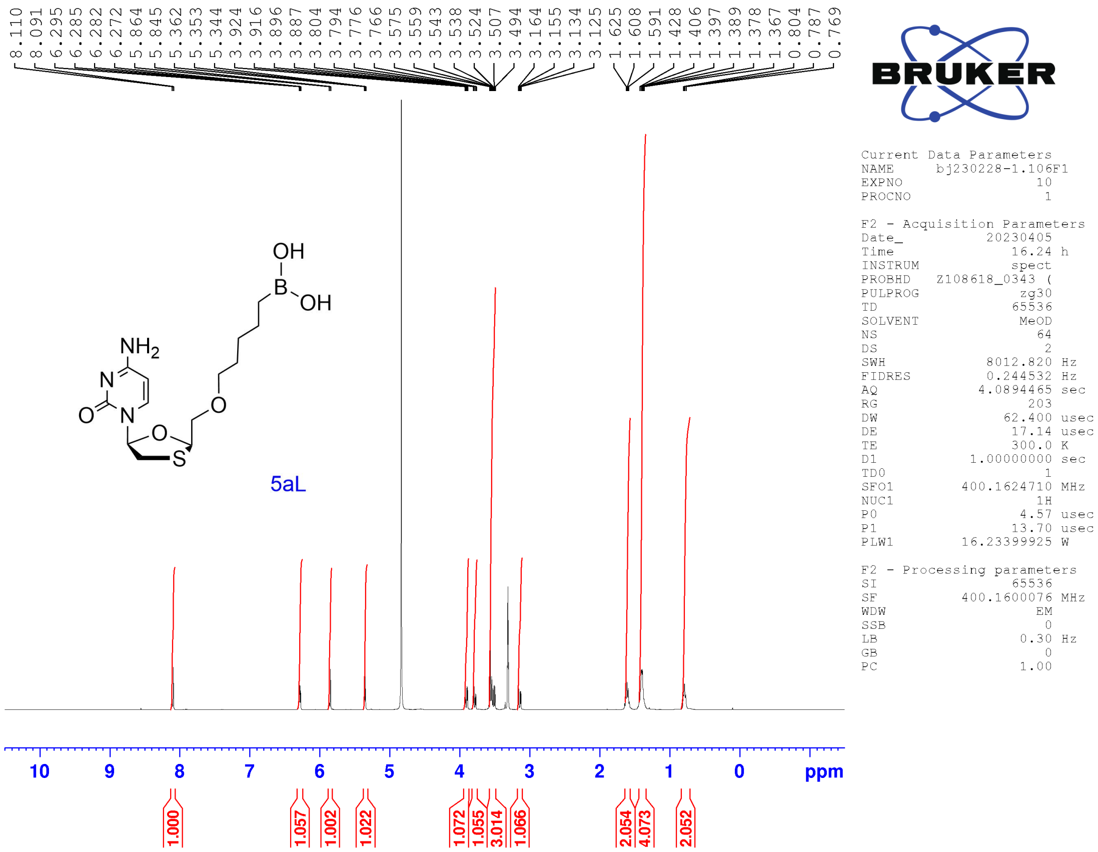

Figure S205 ^{1}HNMR spectra of **5aL** in CD_3OD.

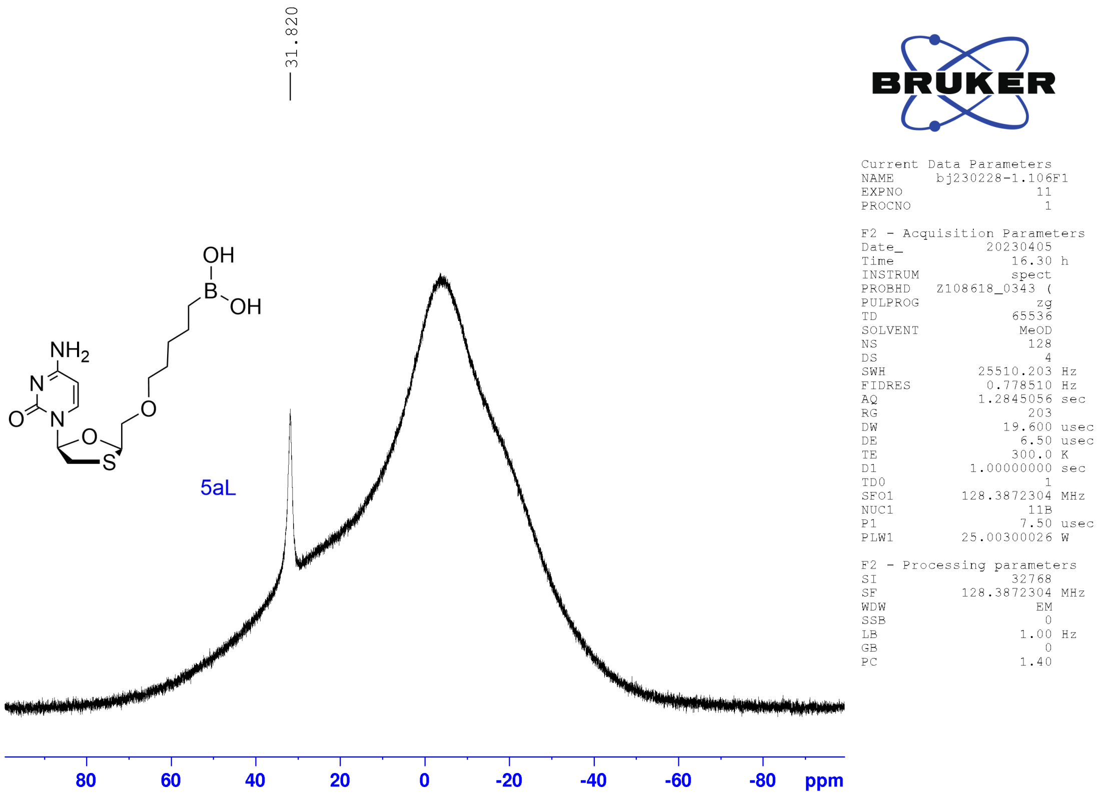

Figure S206 ^{11}BNMR spectra of **5aL** in CD_3OD.

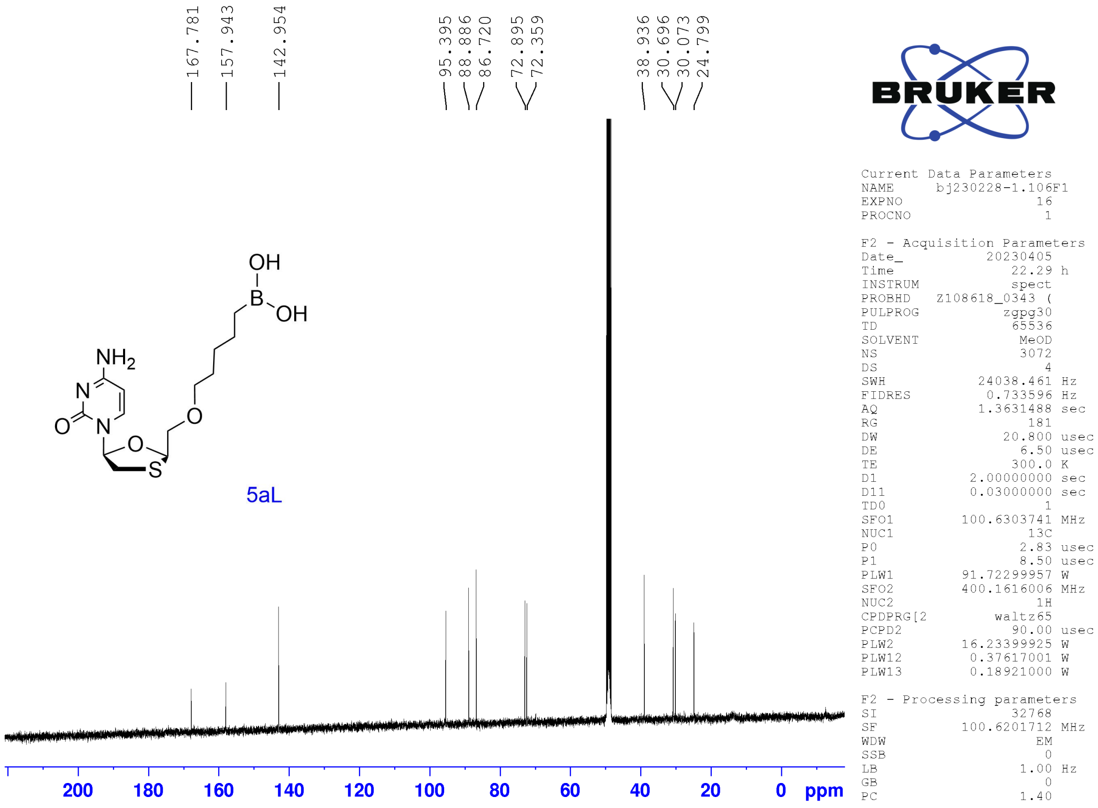

Figure S207 $^{13}C\{^1H\}$-NMR spectra of **5aL** in CD_3OD.

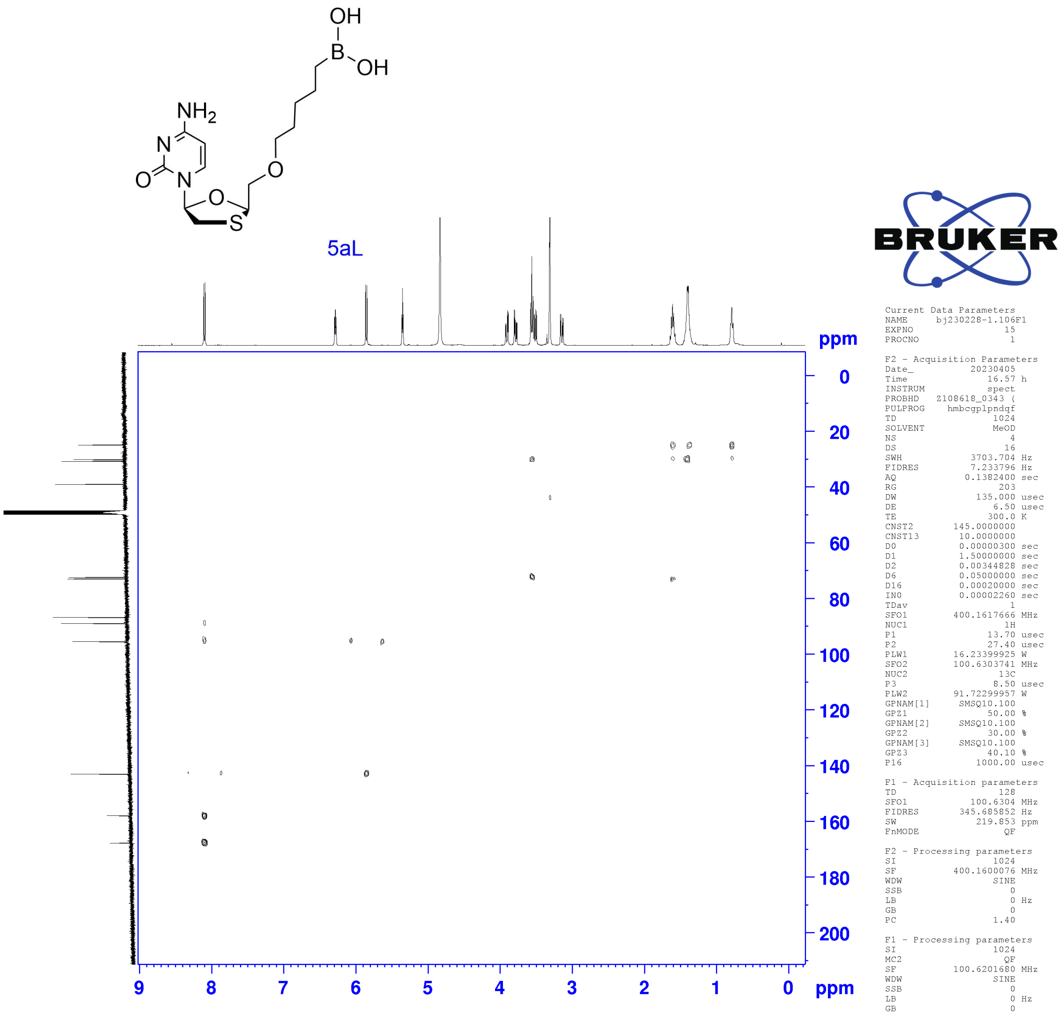

Figure S208 HMBC-NMR spectra of **5aL** in CD_3OD.

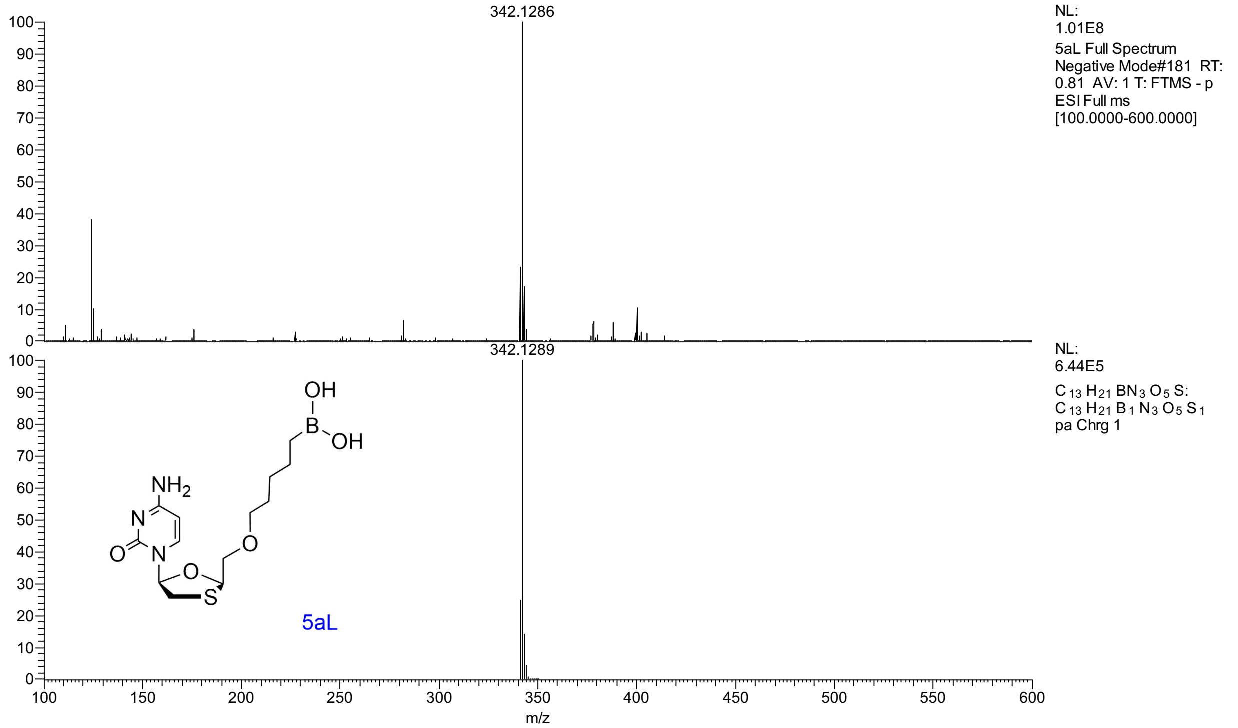

Figure S209 HRMS (ESI-) negative mode m/z calculated for **5aL** [$C_{13}H_{21}BN_3O_5S$] $[M-H]^-$ 342.1289, found 342.1286.

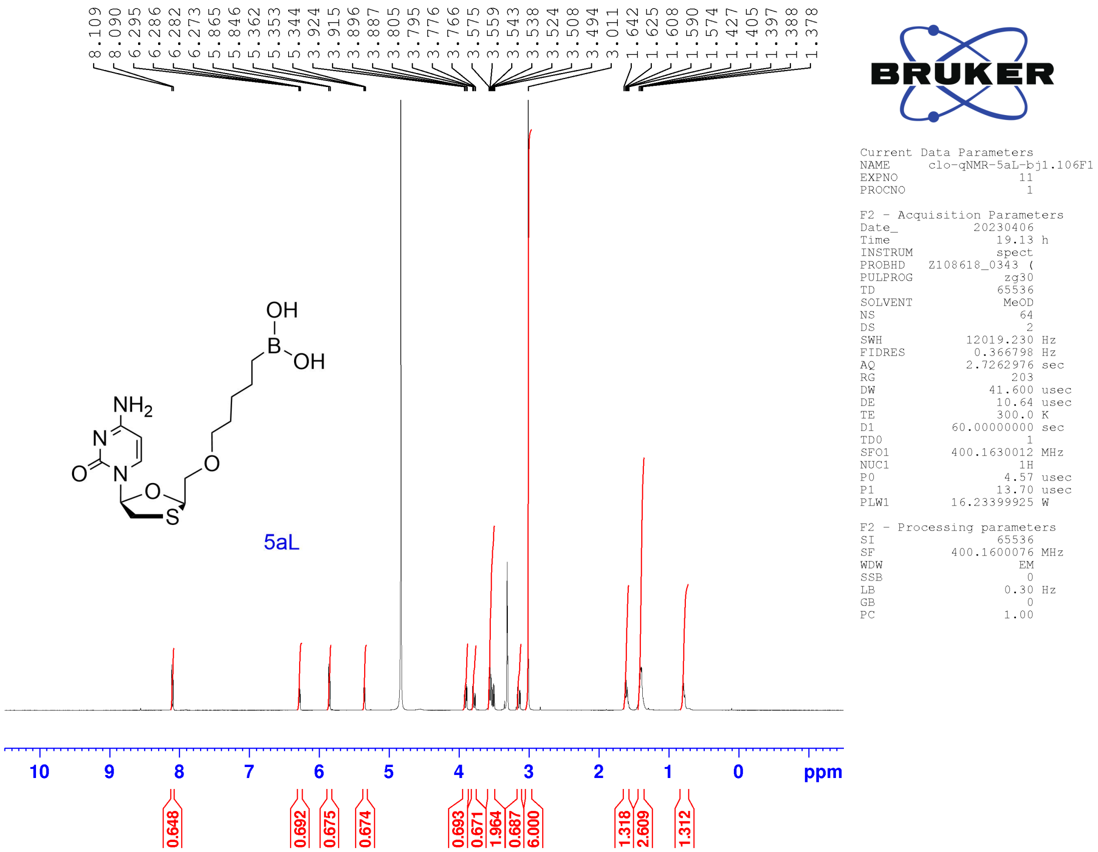

Figure S210 ^{1}H qNMR purity spectrum of **5aL** with Me_2SO_2 (I.C) in CD_3OD. Purity 97.24%.

$$P_{\text{sample}} = \frac{S_{\text{sample}} \text{ x } N_{\text{std}} \text{ x } m_{\text{std}} \text{ x } M_{\text{sample}}}{S_{\text{std}} \text{ x } N_{\text{sample}} \text{ x } m_{\text{sample}} \text{ x } M_{\text{std}}} \text{ x } P_{\text{std}}$$

$$= \frac{0.675 \text{ x } 6 \text{ x } 1.7 \text{ mg x } 343.14 \text{ g mol}^{-1}}{6 \text{ x } 1 \text{ x } 4.3 \text{ mg x } 94.13 \text{ g mol}^{-1}} \text{ x } 99.96$$

$$= 97.24\%$$

S = Integrated area of the peak
N = Number of protons represented
m = Prepared mass
M = Molecular weight
P = Purity

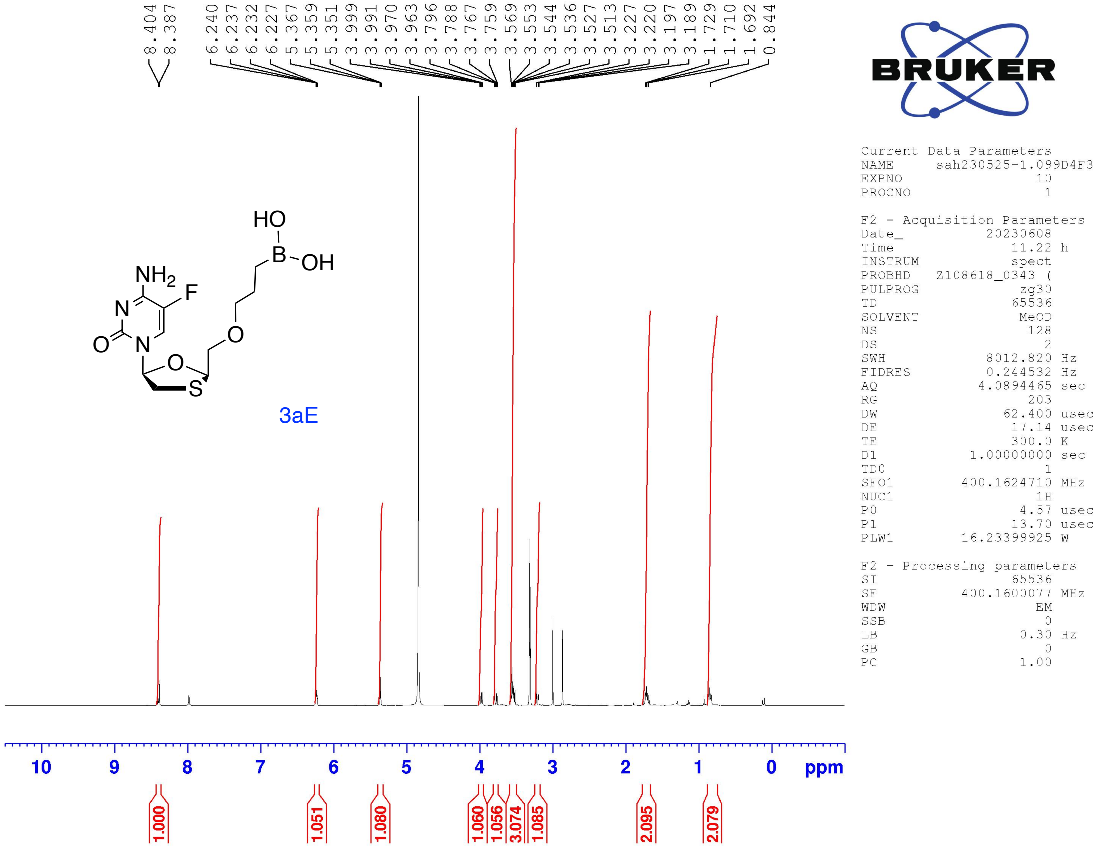

Figure S211 ^{1}HNMR spectra of **3aE** in CD_3OD.

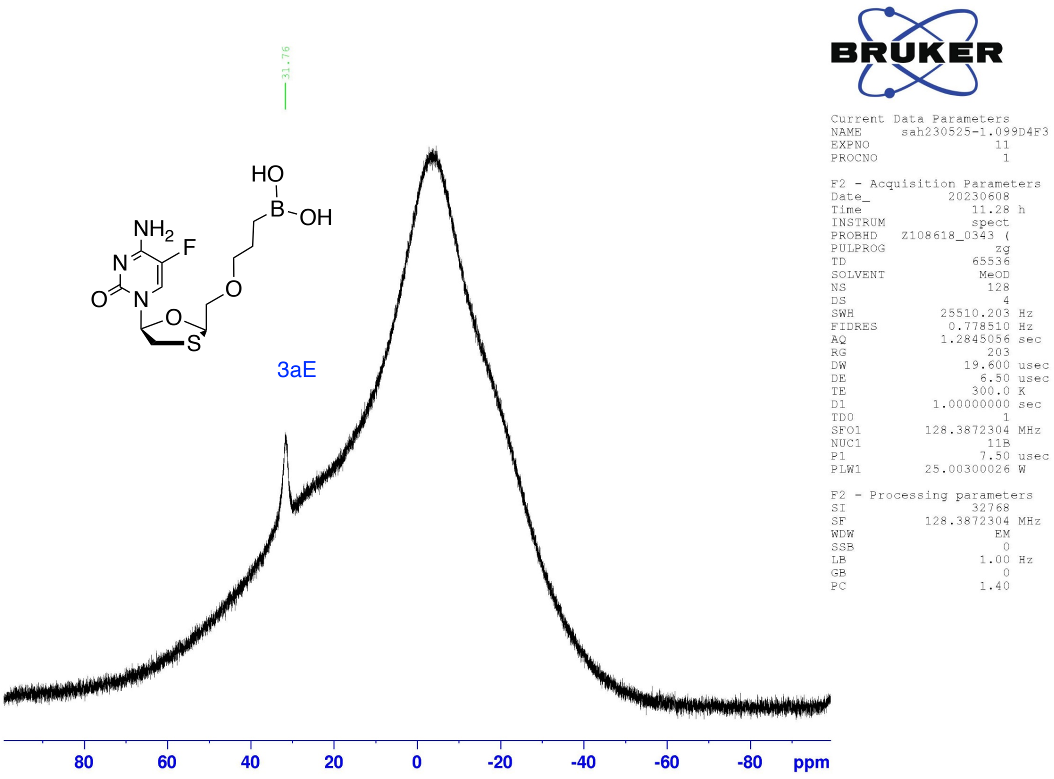

Figure S212 ^{11}BNMR spectra of **3aE** in CD_3OD.

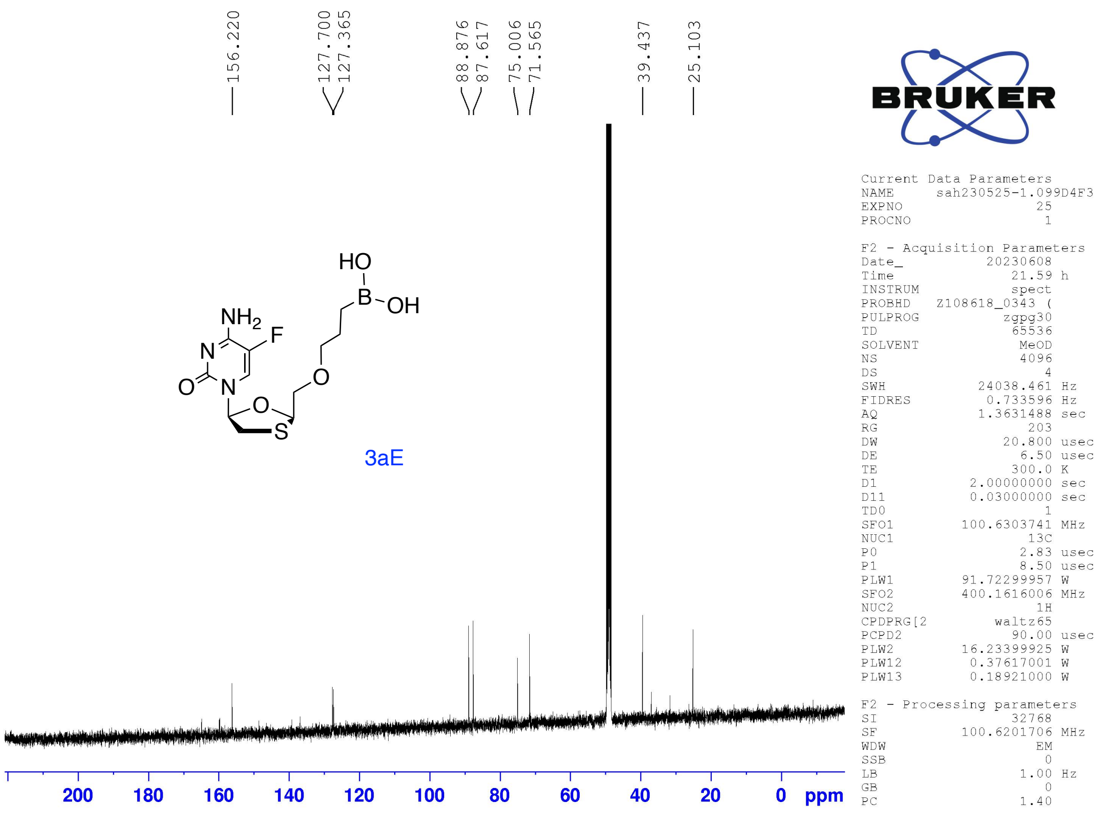

Figure S213 $^{13}C\{^{1}H\}$-NMR spectra of **3aE** in CD_3OD.

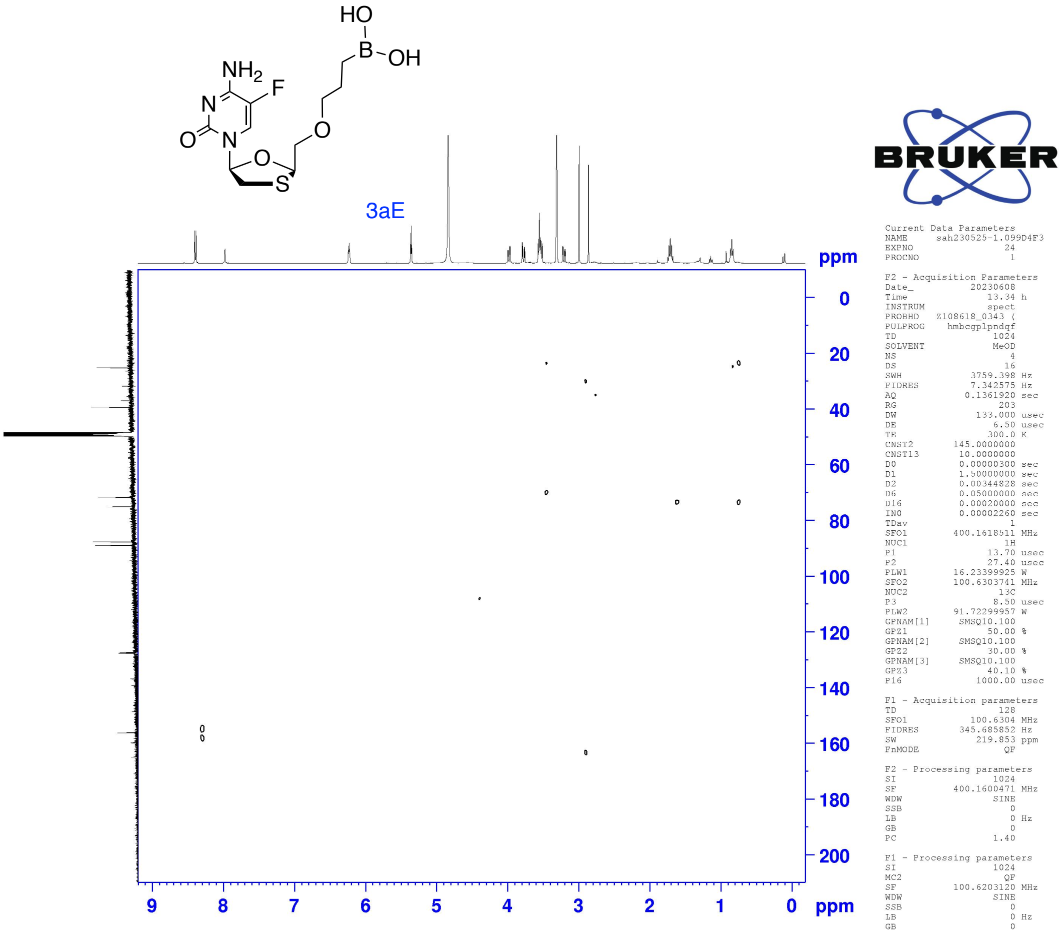

Figure S214 HMBC-NMR spectra of **3aE** in CD_3OD.

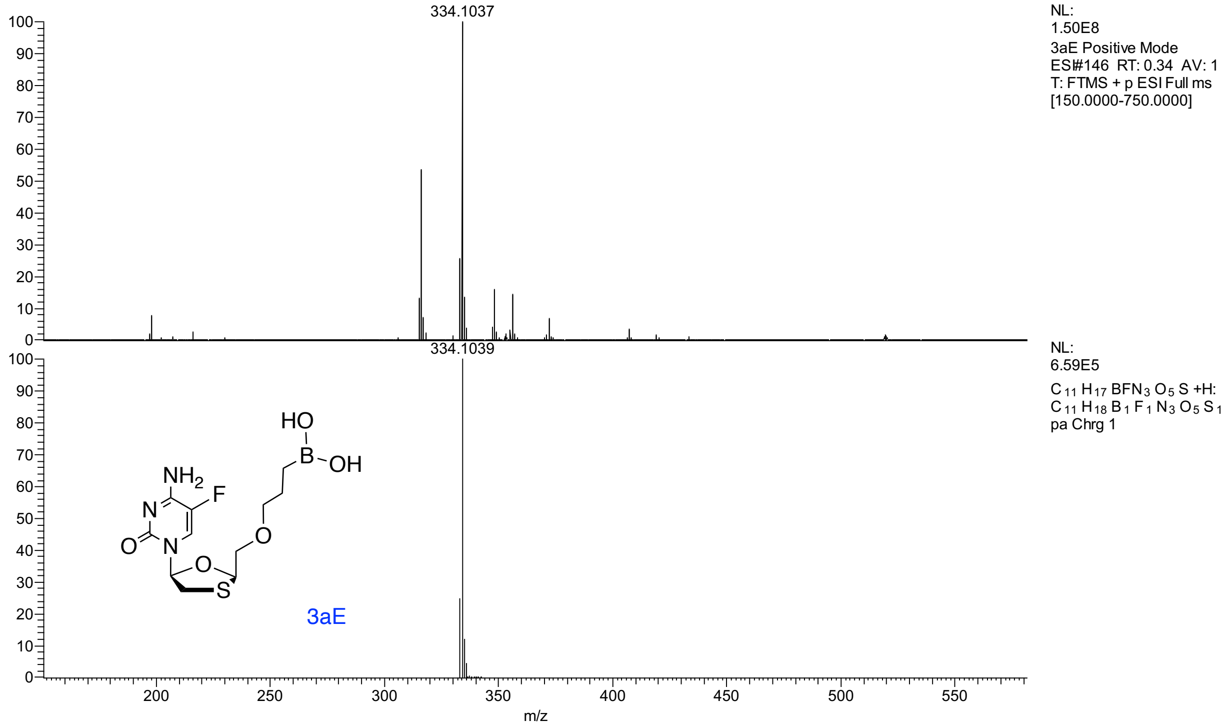

Figure S215 HRMS (ESI+) positive mode m/z calculated for **3aE** [$C_{11}H_{18}BFN_3O_5S$] $[M+H]^+$ 334.1039, found 334.1037.

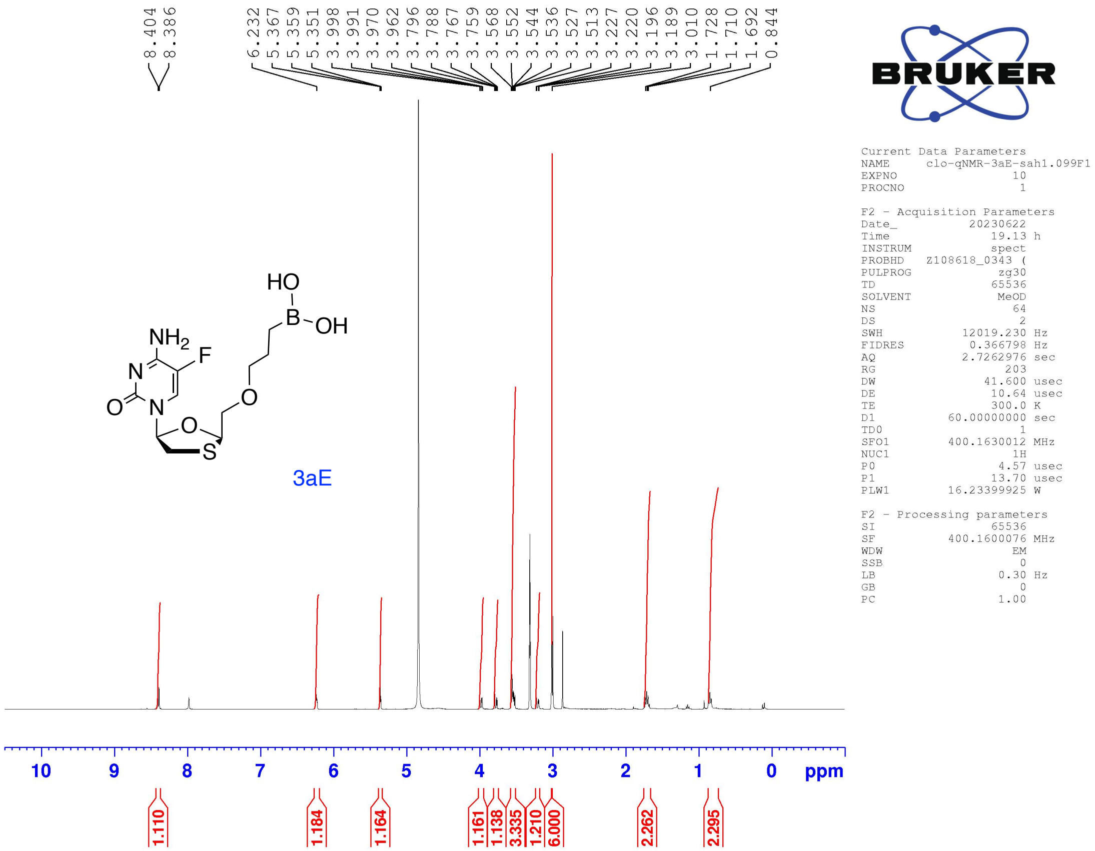

Figure S216 ^{1}H qNMR purity spectrum of **3aE** with Me_2SO_2 (I.C) in CD_3OD. Purity 97.10%.

$$P_{\text{sample}} = \frac{S_{\text{sample}} \text{ x } N_{\text{std}} \text{ x } m_{\text{std}} \text{ x } M_{\text{sample}}}{S_{\text{std}} \text{ x } N_{\text{sample}} \text{ x } m_{\text{sample}} \text{ x } M_{\text{std}}} \text{ x } P_{\text{std}}$$

$$= \frac{1.184 \text{ x } 6 \text{ x } 2.2 \text{ mg x } 314.10 \text{ g mol}^{-1}}{6 \text{ x } 1 \text{ x } 9.5 \text{ mg x } 94.13 \text{ g mol}^{-1}} \text{ x } 99.96$$

$$= 97.10\%$$

S = Integrated area of the peak
N = Number of protons represented
m = Prepared mass
M = Molecular weight
P = Purity

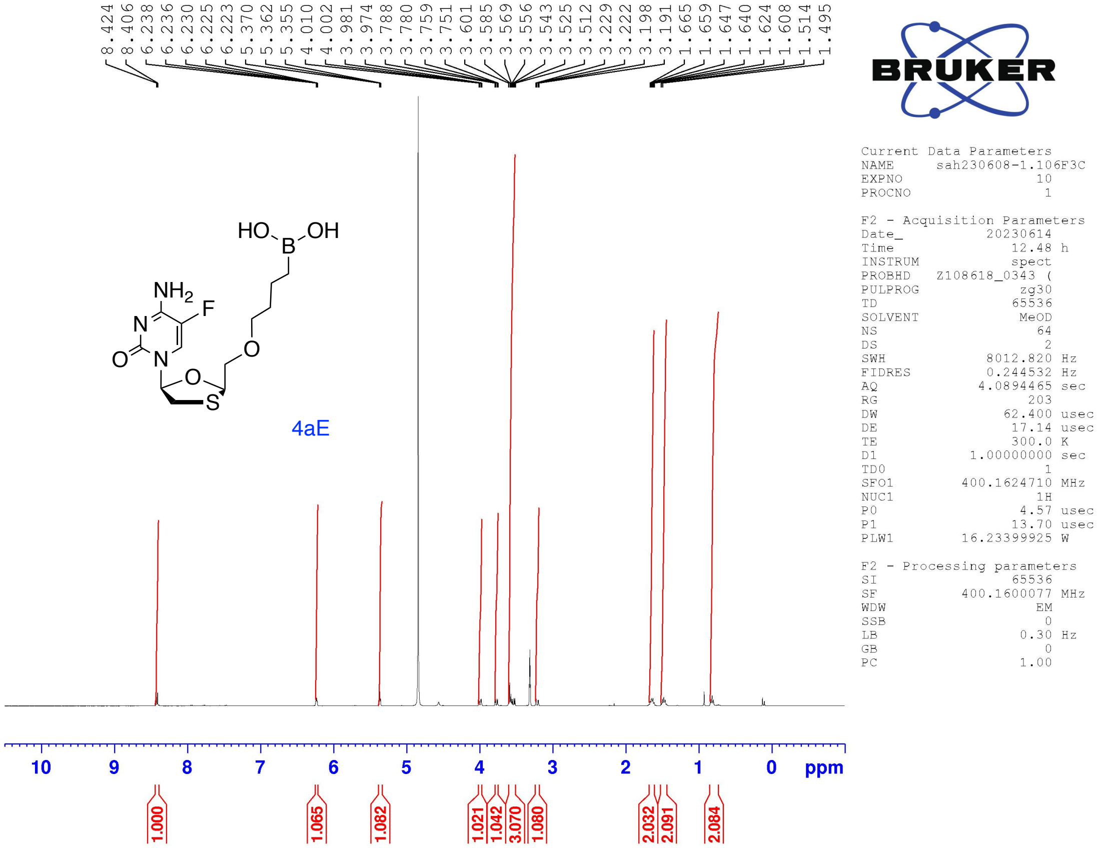

Figure S217 ^{1}HNMR spectra of **4aE** in CD_3OD.

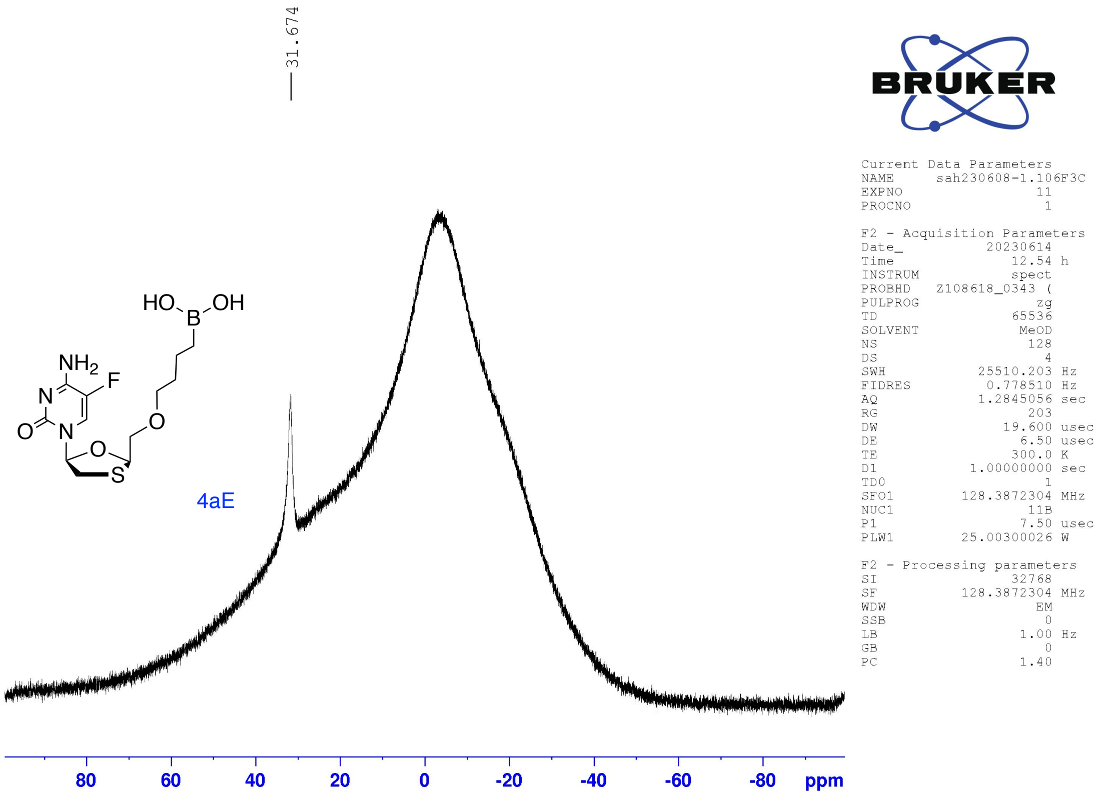

Figure S218 ^{11}BNMR spectra of **4aE** in CD_3OD.

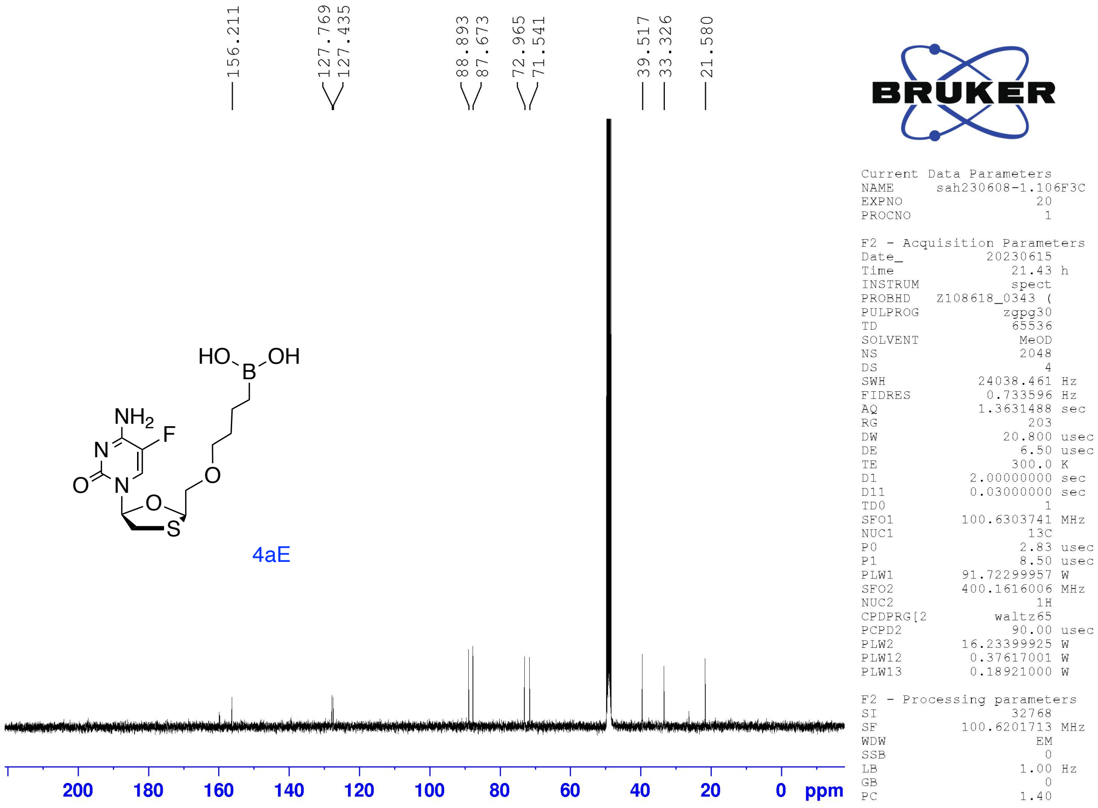

Figure S219 $^{13}C\{^{1}H\}$-NMR spectra of **4aE** in CD_3OD.

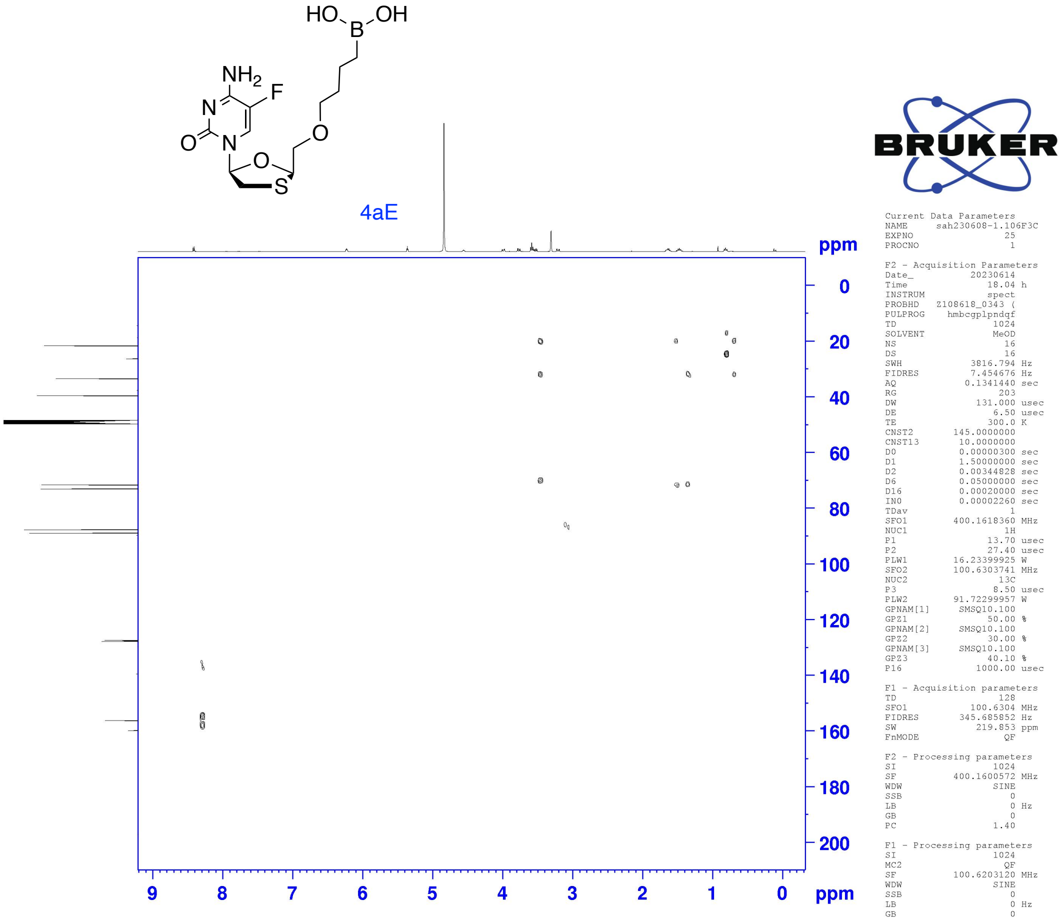

Figure S220 HMBC-NMR spectra of **4aE** in CD_3OD.

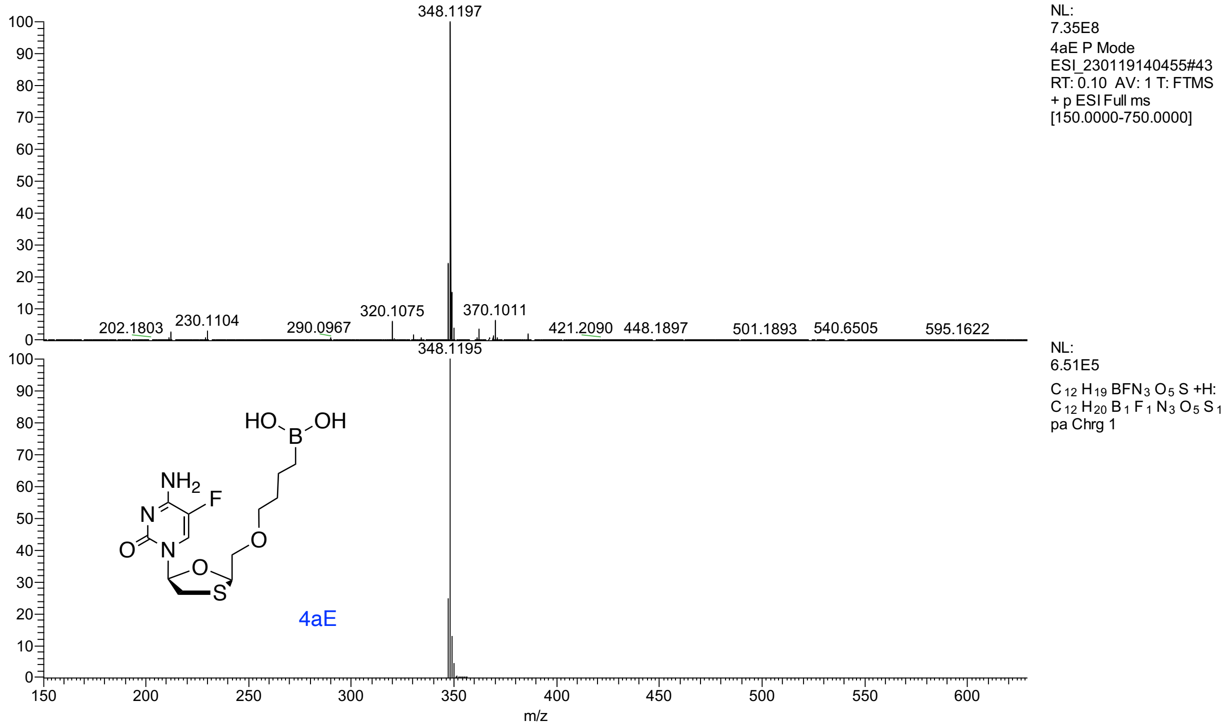

Figure S221 HRMS (ESI+) positive mode m/z calculated for **4aE** [$C_{12}H_{20}BFN_3O_5S$] $[M+H]^+$ 348.1195, found 348.1197.

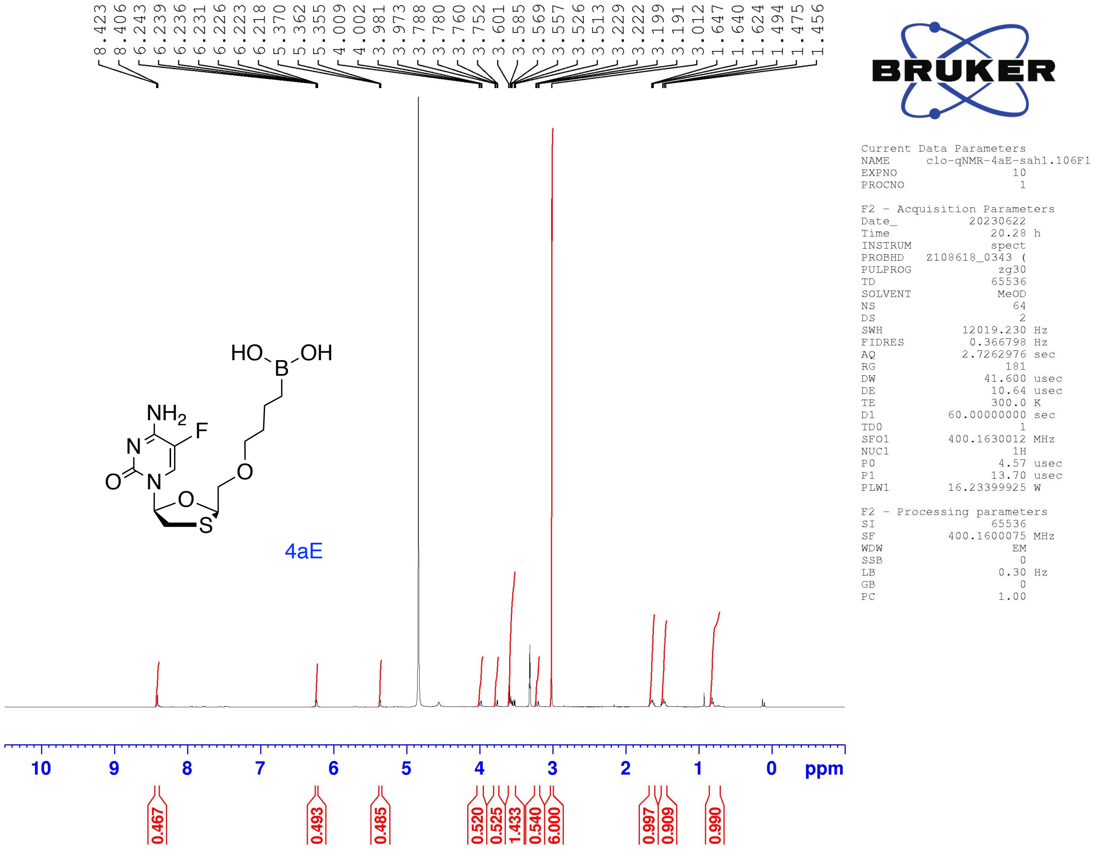

Figure S222 ^{1}H qNMR purity spectrum of **4aE** with Me_2SO_2 (I.C) in CD_3OD. Purity 96.47%.

$$P_{\text{sample}} = \frac{S_{\text{sample}} \text{ x } N_{\text{std}} \text{ x } m_{\text{std}} \text{ x } M_{\text{sample}}}{S_{\text{std}} \text{ x } N_{\text{sample}} \text{ x } m_{\text{sample}} \text{ x } M_{\text{std}}} \text{ x } P_{\text{std}}$$

$$= \frac{0.493 \text{ x } 6 \text{ x } 2.5 \text{ mg x } 314.10 \text{ g mol}^{-1}}{6 \text{ x } 1 \text{ x } 4.7 \text{ mg x } 94.13 \text{ g mol}^{-1}} \text{ x } 99.96$$

$$= 96.47\%$$

S = Integrated area of the peak
N = Number of protons represented
m = Prepared mass
M = Molecular weight
P = Purity

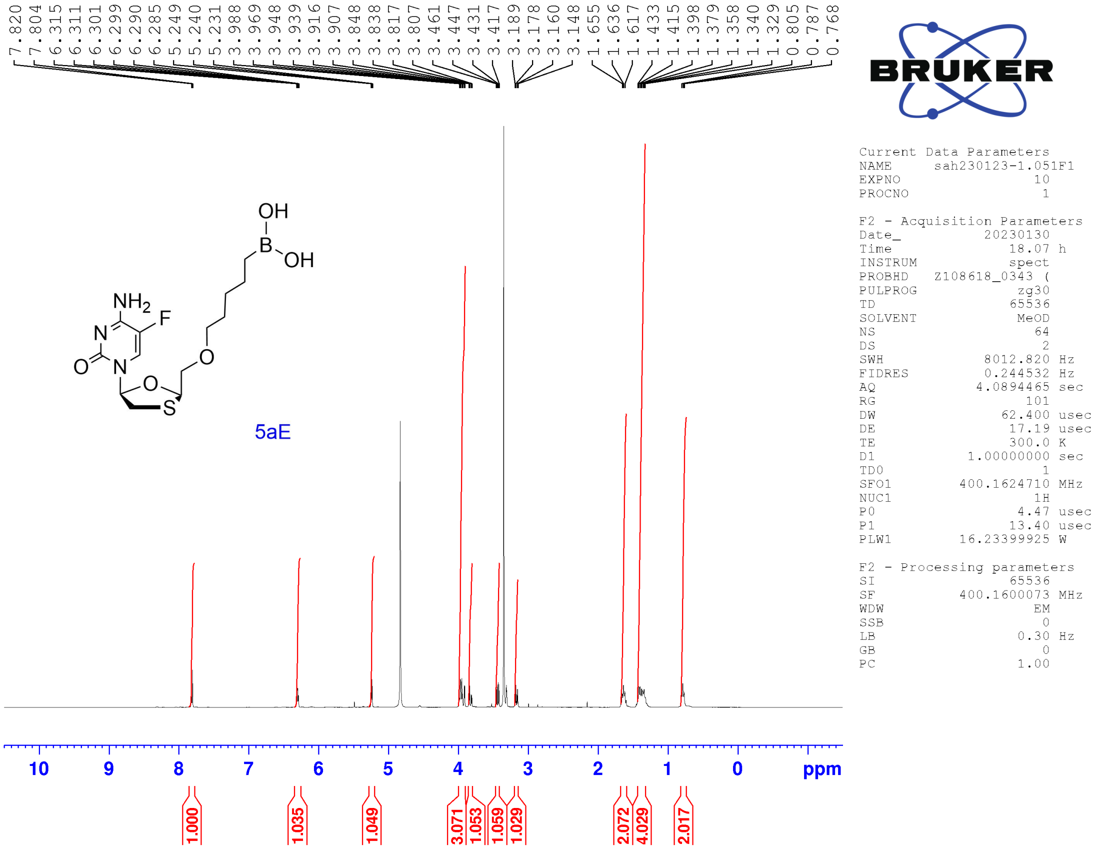

Figure S223 ^{1}HNMR spectra of **5aE** in CD_3OD.

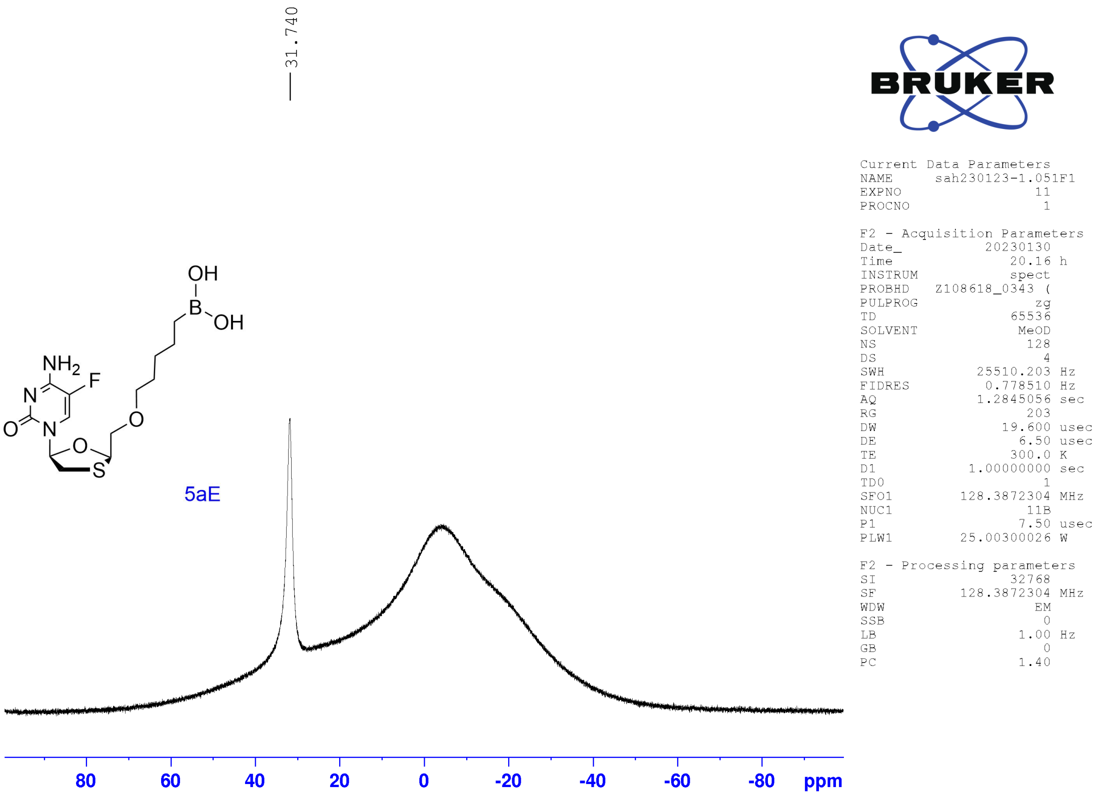

Figure S224 ^{11}BNMR spectra of **5aE** in CD_3OD.

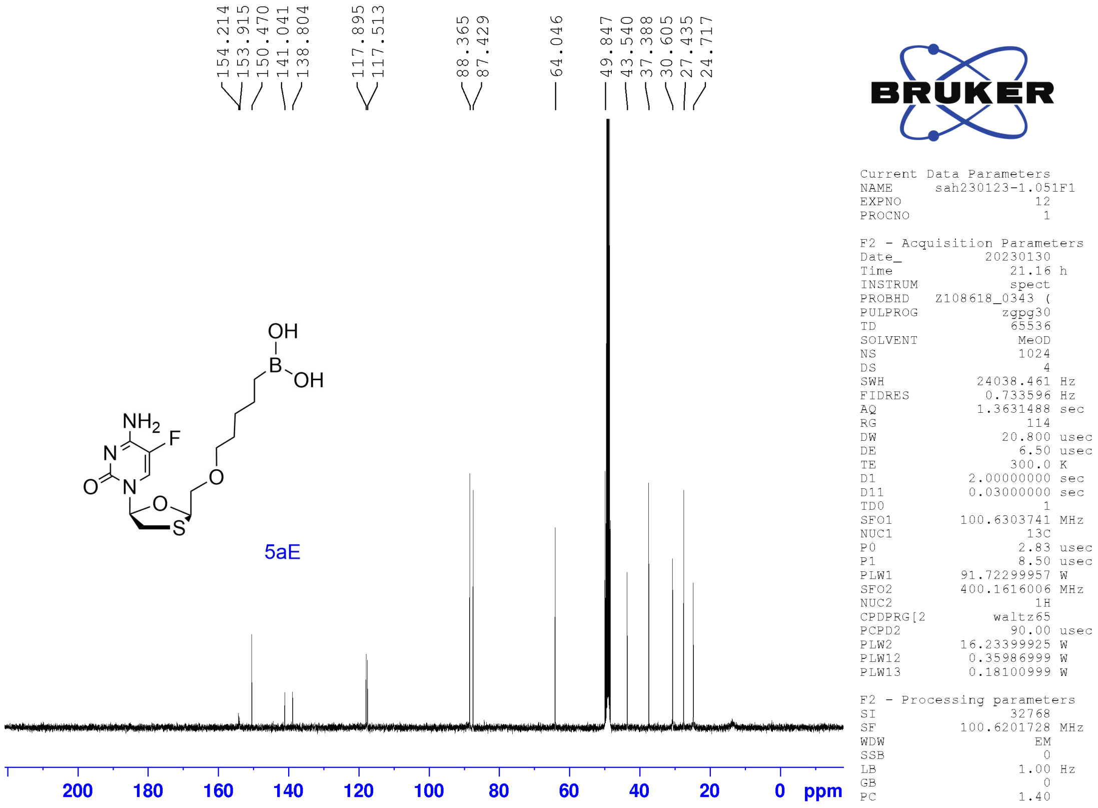

Figure S225 $^{13}C\{^1H\}$-NMR spectra of **5aE** in CD_3OD.

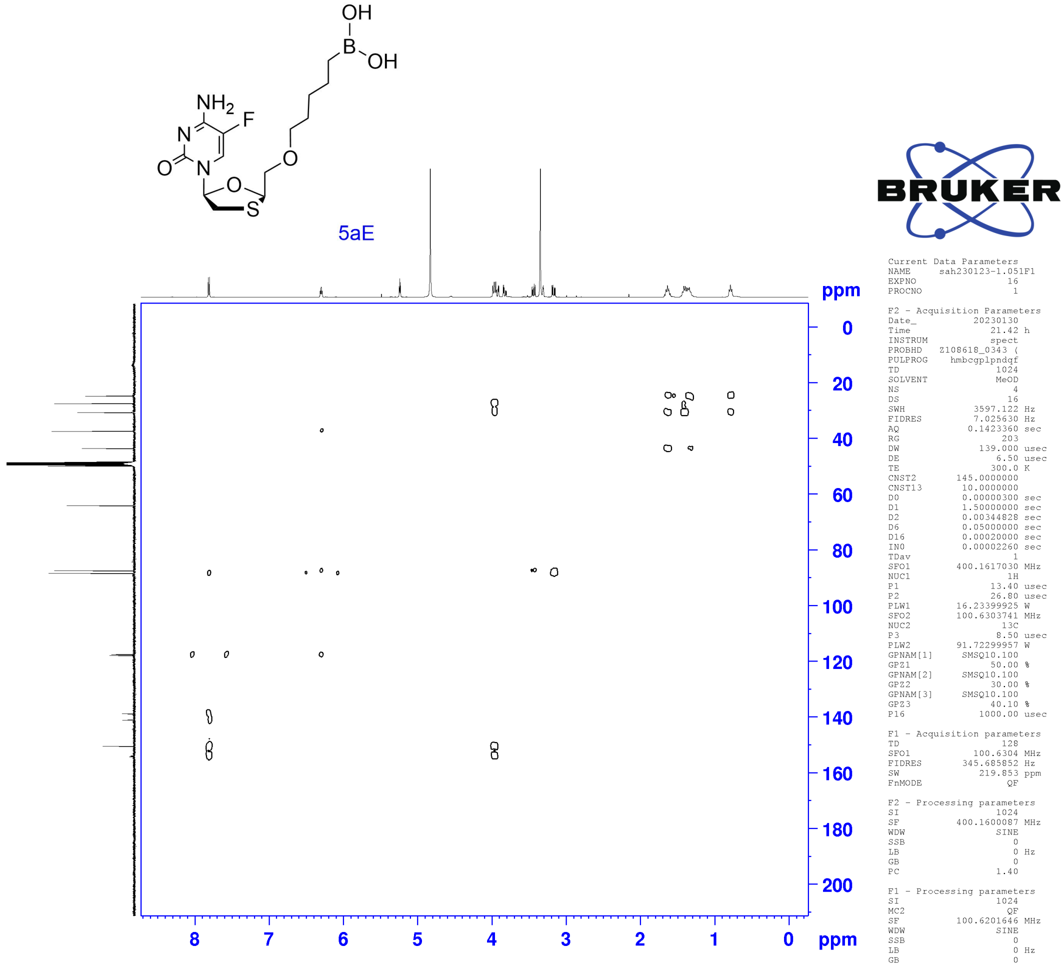

Figure S226 HMBC-NMR spectra of **5aE** in CD_3OD.

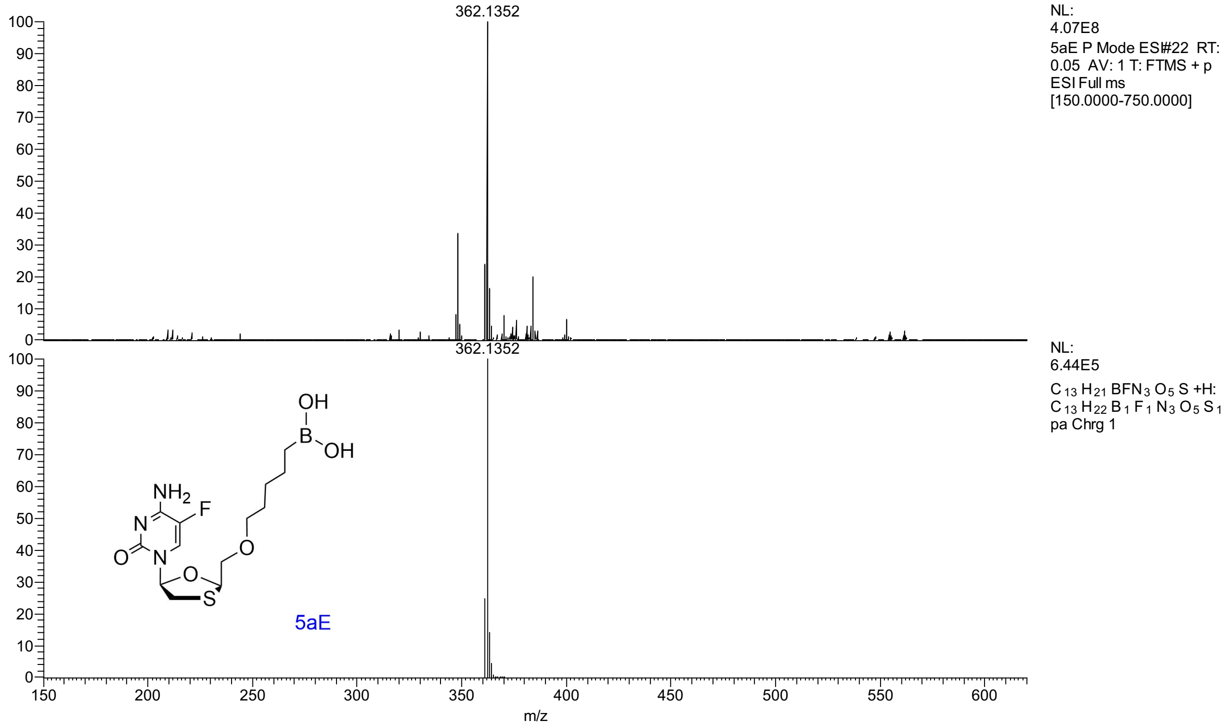

Figure S227 HRMS (ESI+) positive mode m/z calculated for **5aE** [$C_{13}H_{22}BFN_3O_5S$] $[M+H]^+$ 362.1352, found 362.1352.

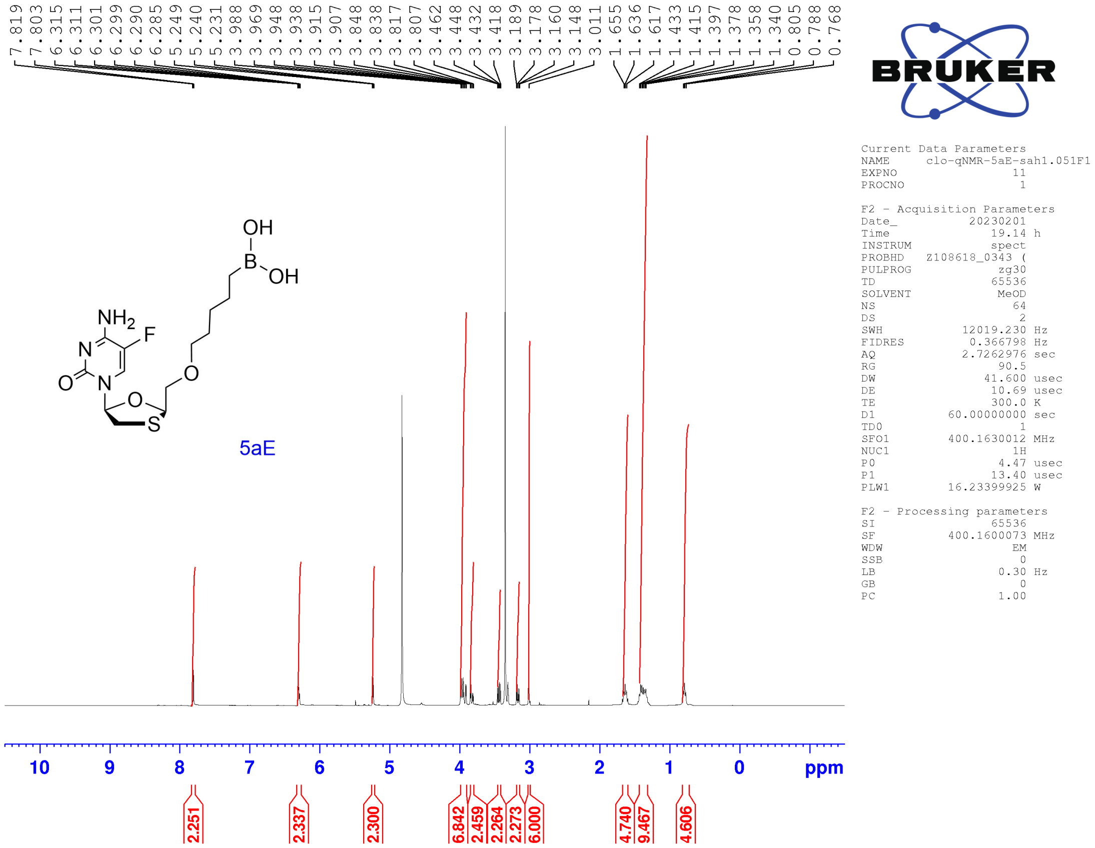

Figure S228 ^{1}H qNMR purity spectrum of **5aE** with Me_2SO_2 (I.C) in CD_3OD. Purity 96.68%.

$$P_{\text{sample}} = \frac{S_{\text{sample}} \text{ x } N_{\text{std}} \text{ x } m_{\text{std}} \text{ x } M_{\text{sample}}}{S_{\text{std}} \text{ x } N_{\text{sample}} \text{ x } m_{\text{sample}} \text{ x } M_{\text{std}}} \text{ x } P_{\text{std}}$$

$$= \frac{2.300 \text{ x } 6 \text{ x } 2.4 \text{ mg x } 361.20 \text{ g mol}^{-1}}{6 \text{ x } 1 \text{ x } 21.9 \text{ mg x } 94.13 \text{ g mol}^{-1}} \text{ x } 99.96$$

$$= 96.68\%$$

S = Integrated area of the peak
N = Number of protons represented
m = Prepared mass
M = Molecular weight
P = Purity

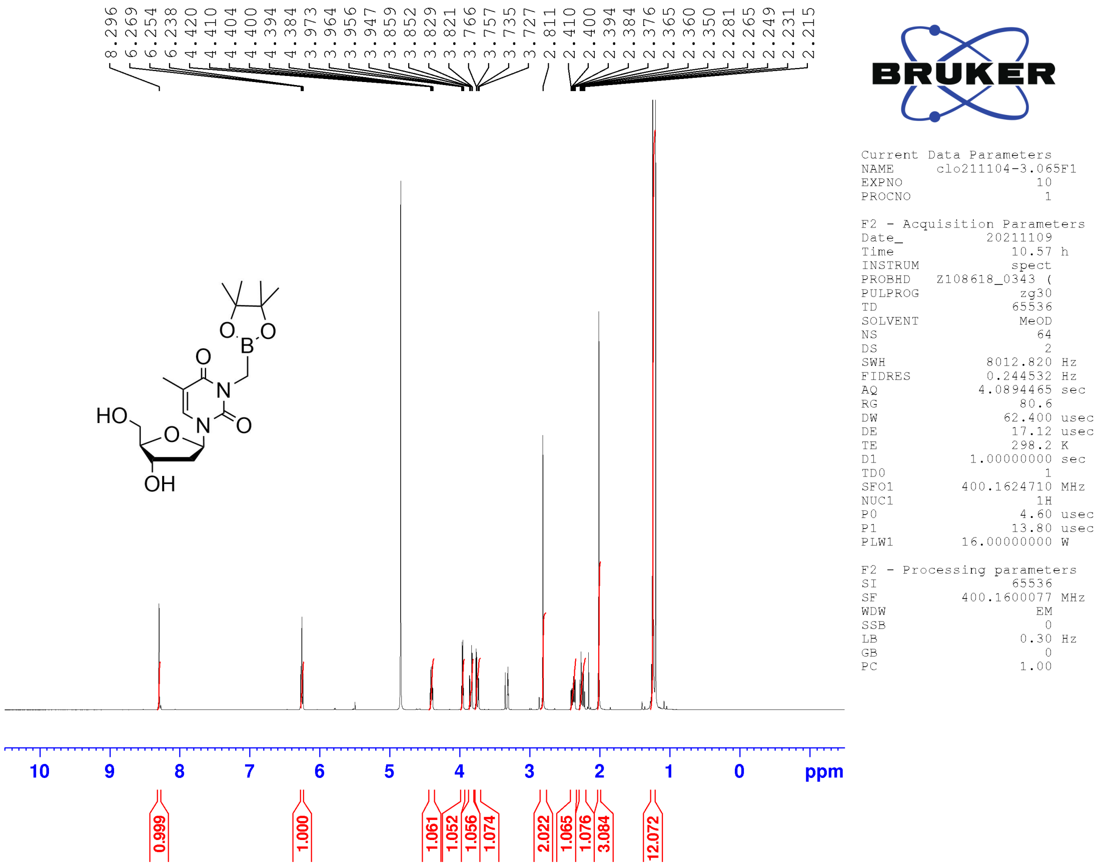

Figure S229 ^{1}HNMR spectra of **N1cT** in CD_3OD.

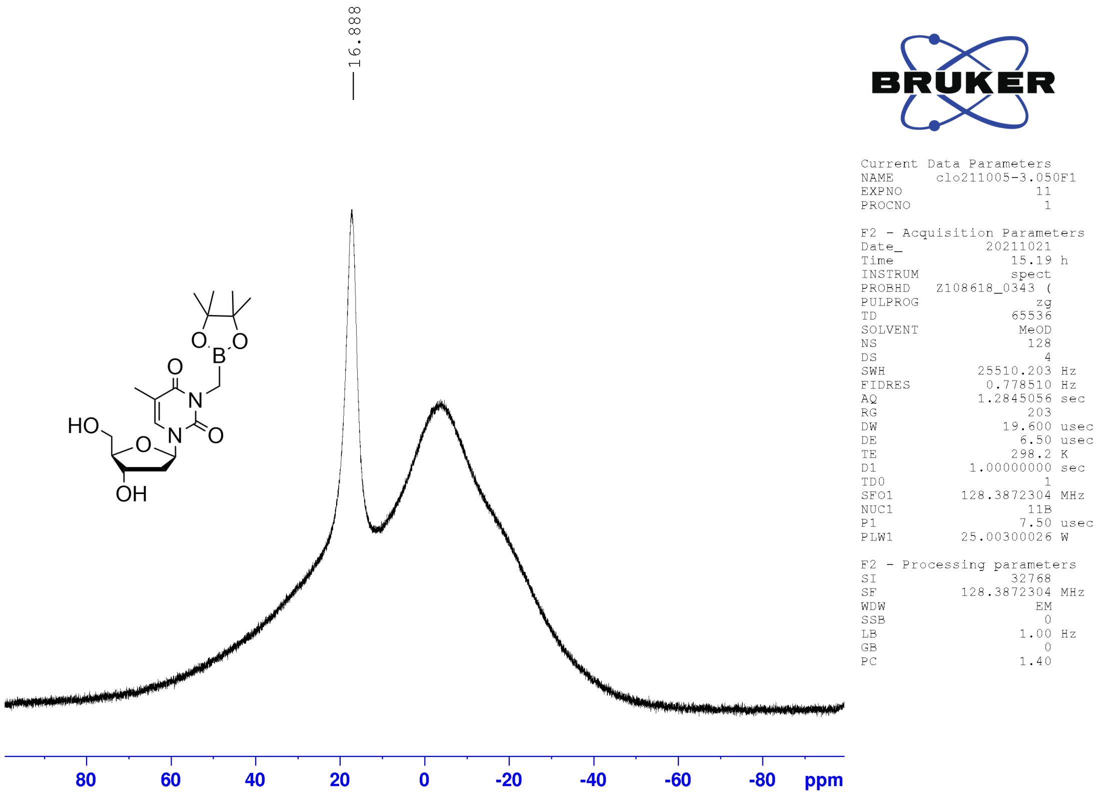

Figure S230 ^{11}BNMR spectra of **N1cT** in CD_3OD.

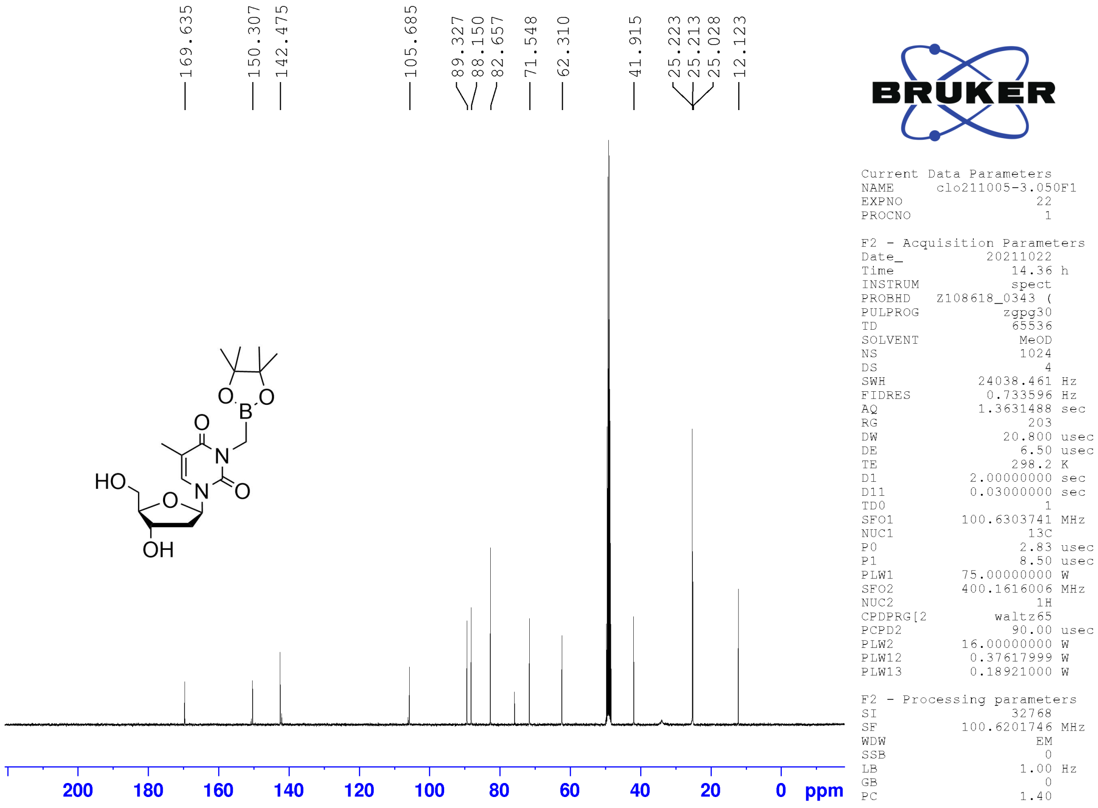

Figure S231 $^{13}C\{^{1}H\}$-NMR spectra of **N1cT** in CD_3OD.

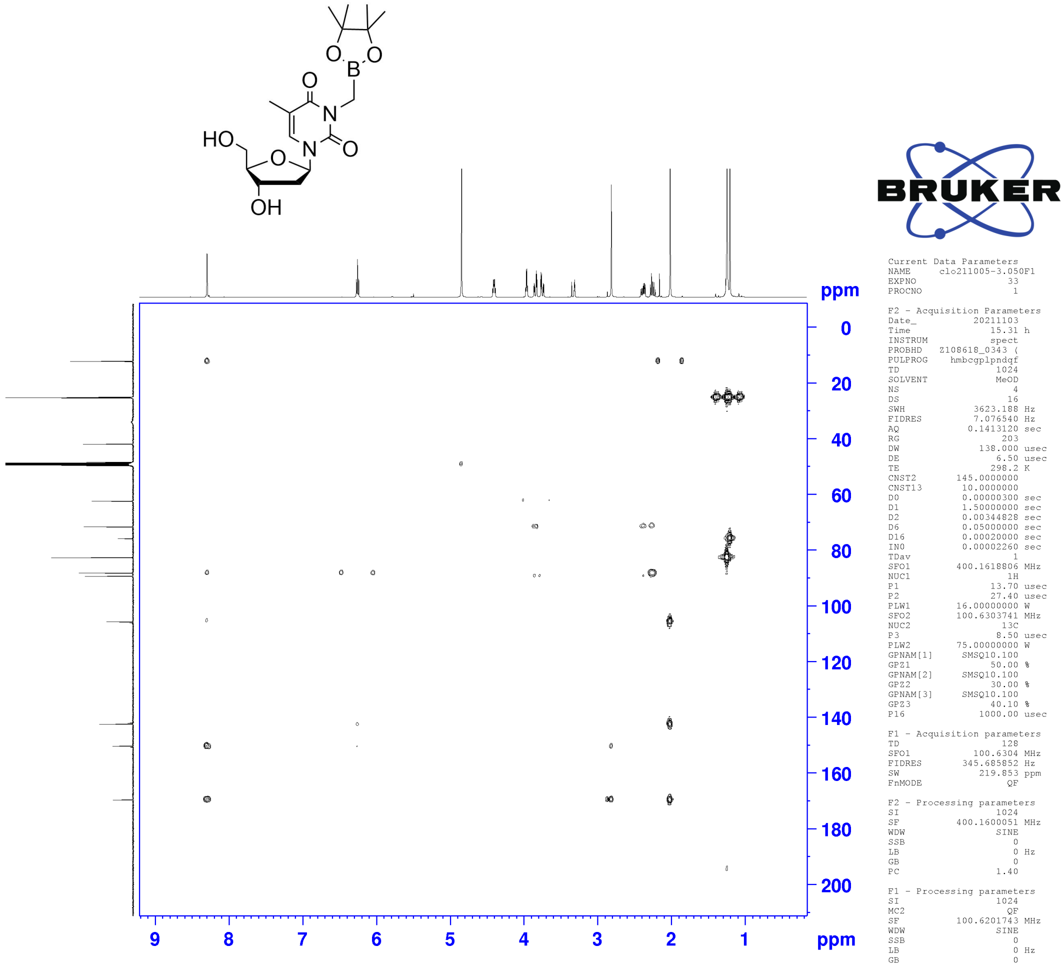

Figure S232 HMBC-NMR spectra of **N1cT** in CD_3OD.

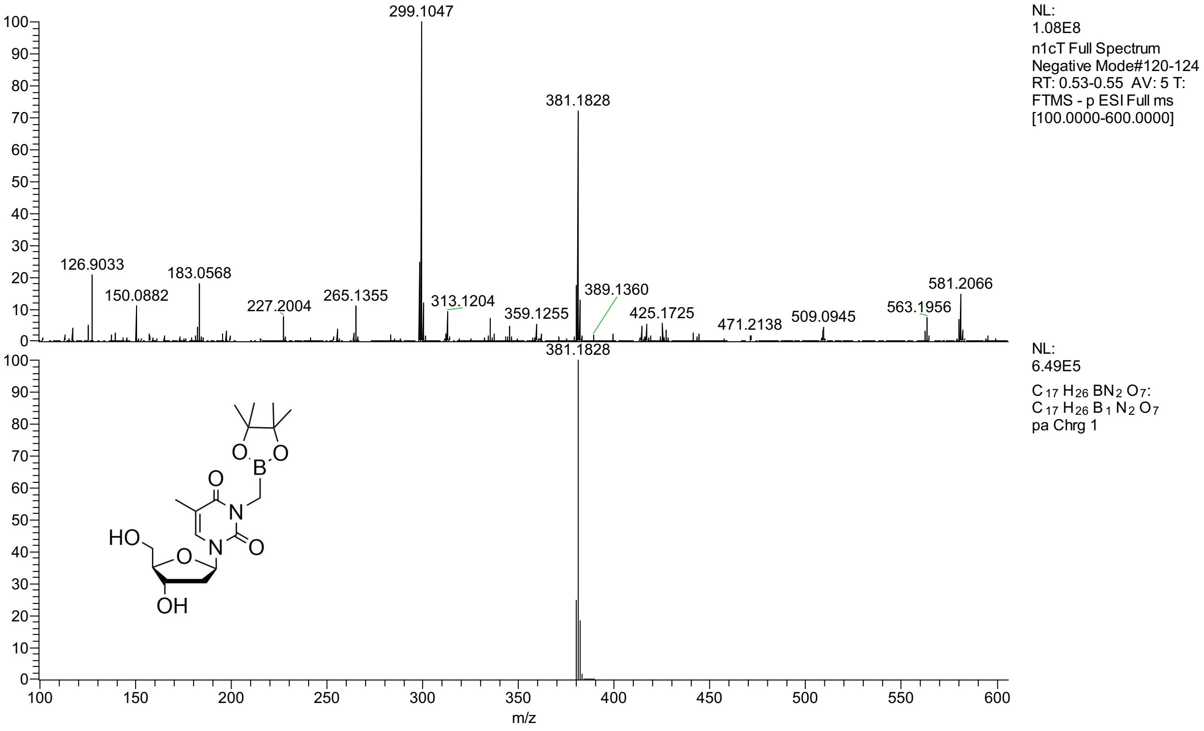

Figure S233 HRMS (ESI-) negative mode m/z calculated for **N1cT** [$C_{17}H_{26}BN_2O_7$] [M-H]$^-$ 381.1828, found 381.1828.

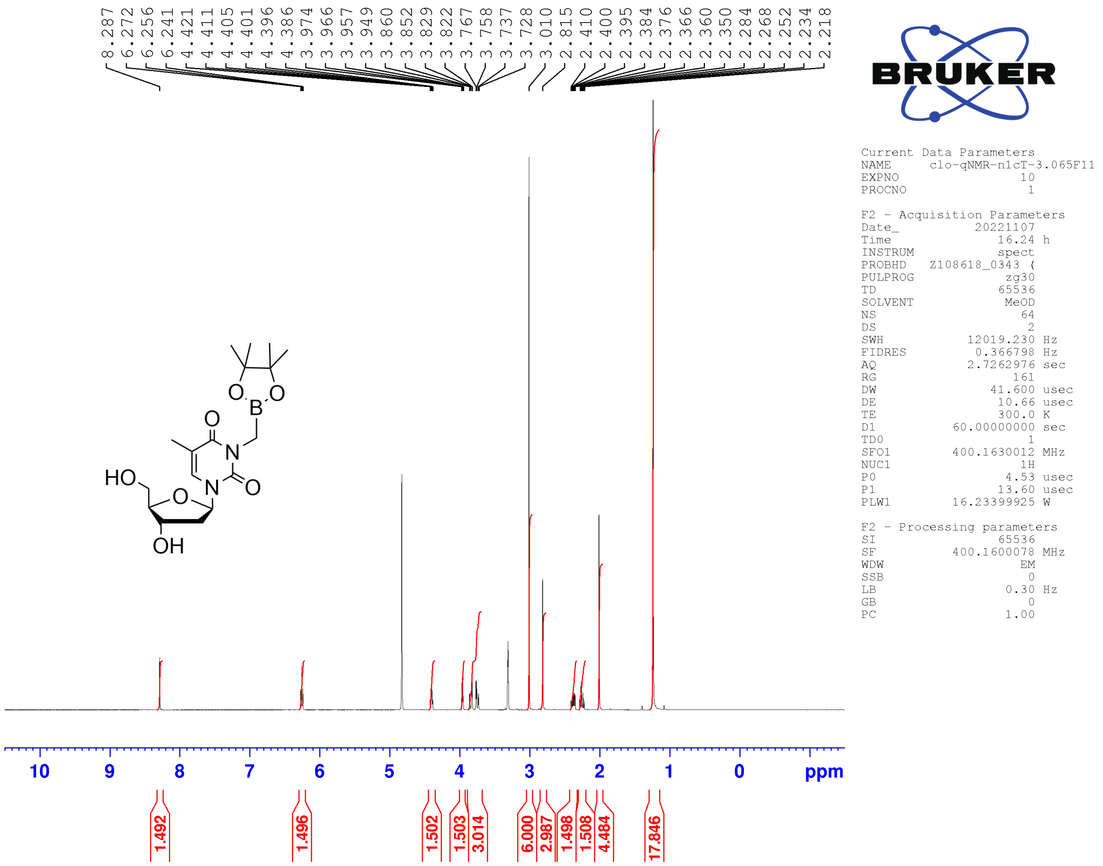

Figure S234 ^{1}H qNMR purity spectrum of **N1cT** with Me_2SO_2 (I.C) in CD_3OD. Purity 97.35%.

$$P_{\text{sample}} = \frac{S_{\text{sample}} \text{ x } N_{\text{std}} \text{ x } m_{\text{std}} \text{ x } M_{\text{sample}}}{S_{\text{std}} \text{ x } N_{\text{sample}} \text{ x } m_{\text{sample}} \text{ x } M_{\text{std}}} \text{ x } P_{\text{std}}$$

$$= \frac{1.492 \text{ x } 6 \text{ x } 2.7 \text{ mg x } 382.22 \text{ g mol}^{-1}}{6 \text{ x } 1 \text{ x } 16.8 \text{ mg x } 94.13 \text{ g mol}^{-1}} \text{ x } 99.96$$

$$= 97.35\%$$

S = Integrated area of the peak
N = Number of protons represented
m = Prepared mass
M = Molecular weight
P = Purity

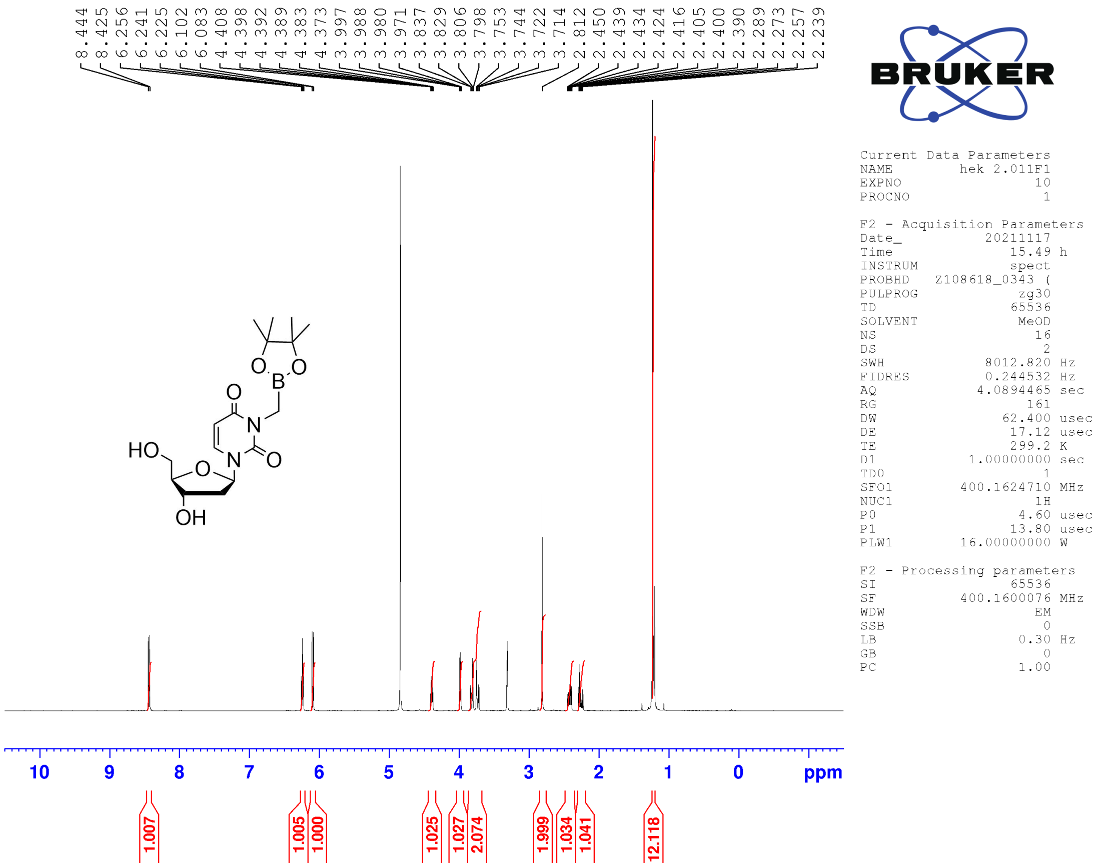

Figure S235 ^{1}HNMR spectra of **N1cU** in CD_3OD.

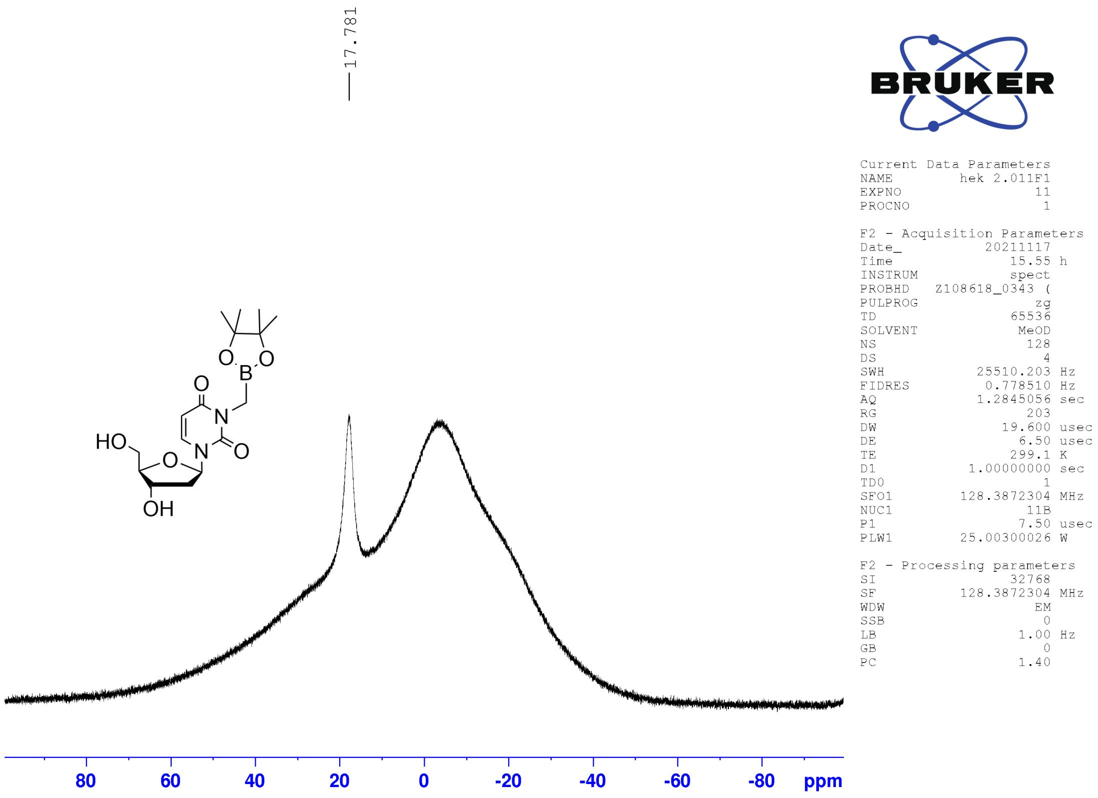

Figure S236 ^{11}BNMR spectra of **N1cU** in CD_3OD.

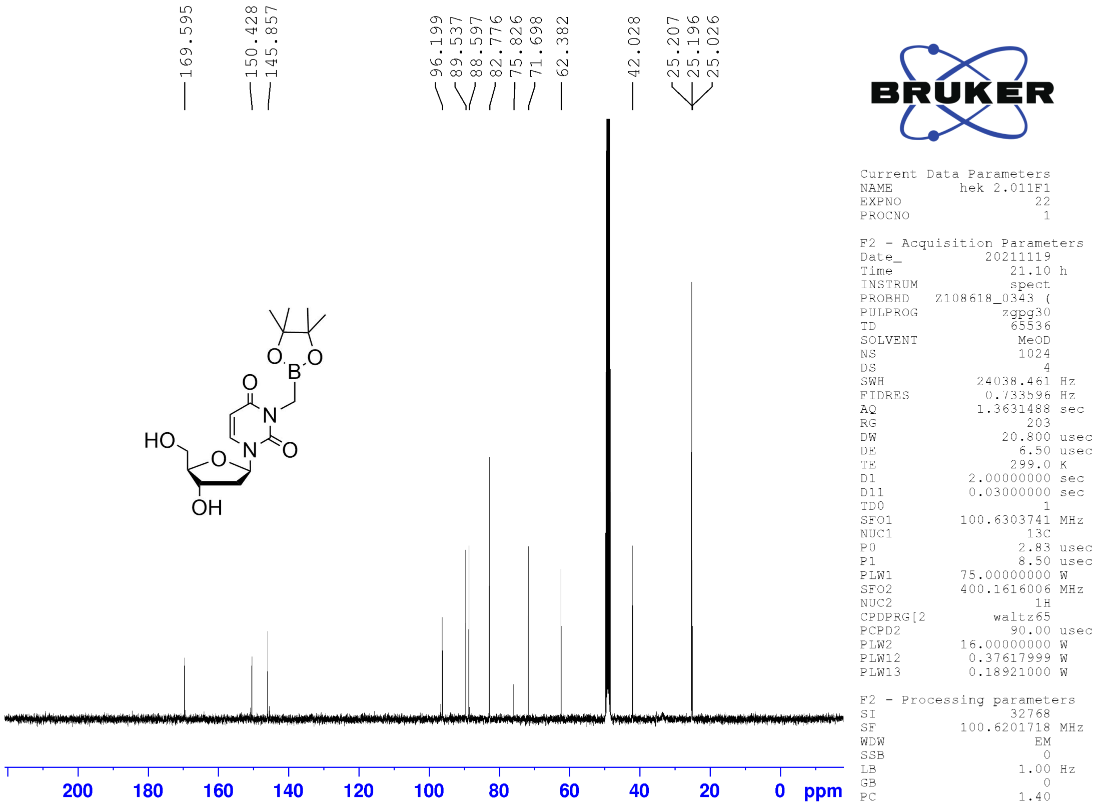

Figure S237 $^{13}C\{^{1}H\}$-NMR spectra of **N1cU** in CD_3OD.

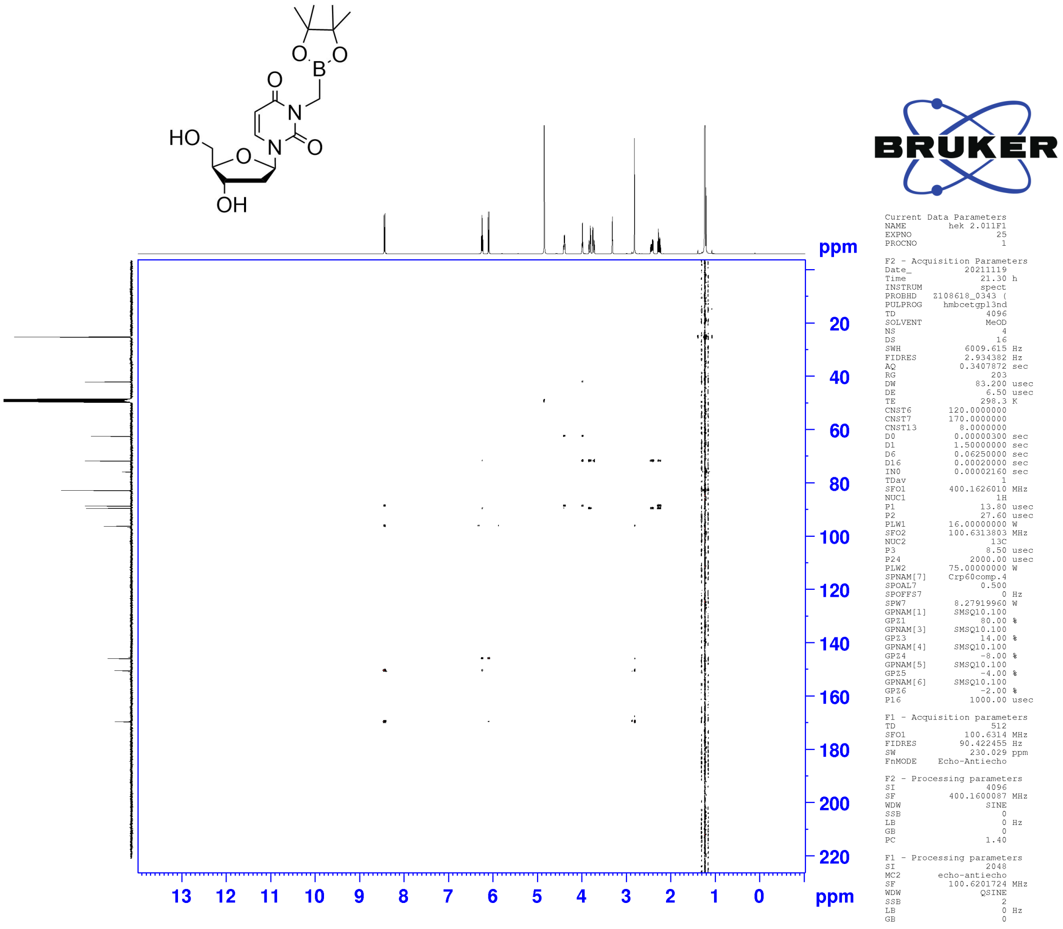

Figure S238 HMBC-NMR spectra of **N1cU** in CD_3OD.

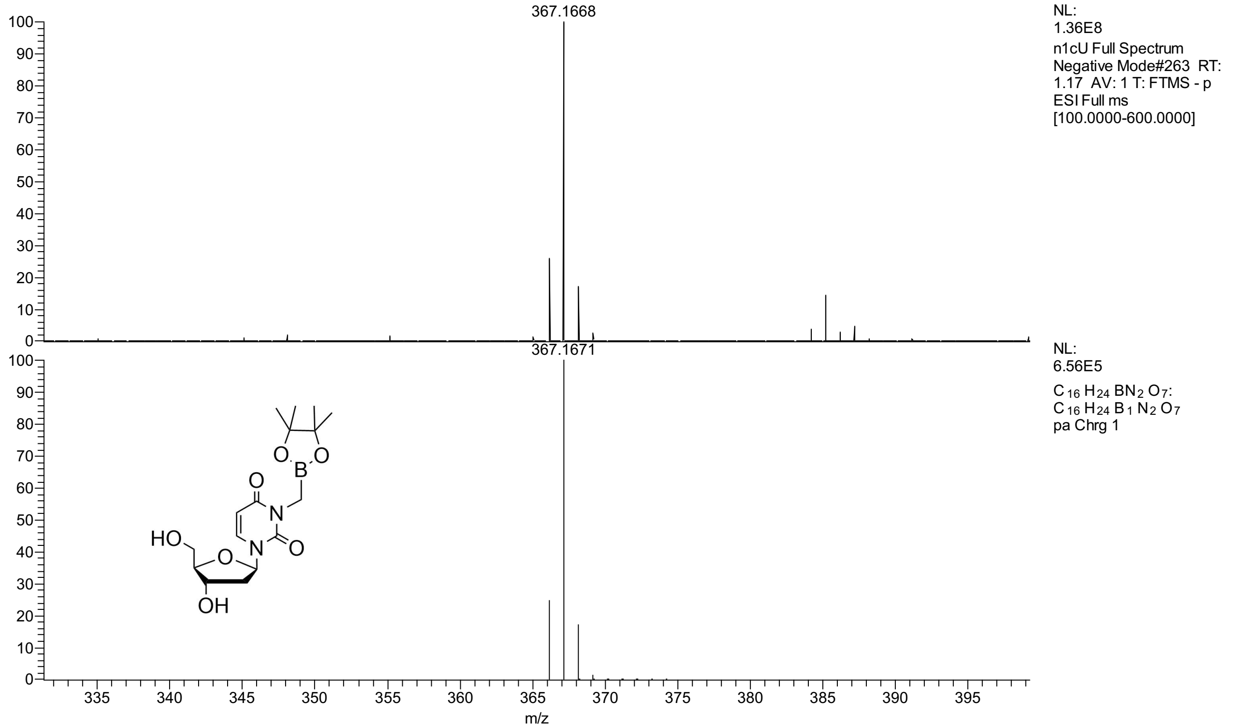

Figure S239 HRMS (ESI-) negative mode m/z calculated for **N1cU** [$C_{16}H_{24}BN_2O_7$] [M-H]$^-$ 367.1671, found 367.1668.

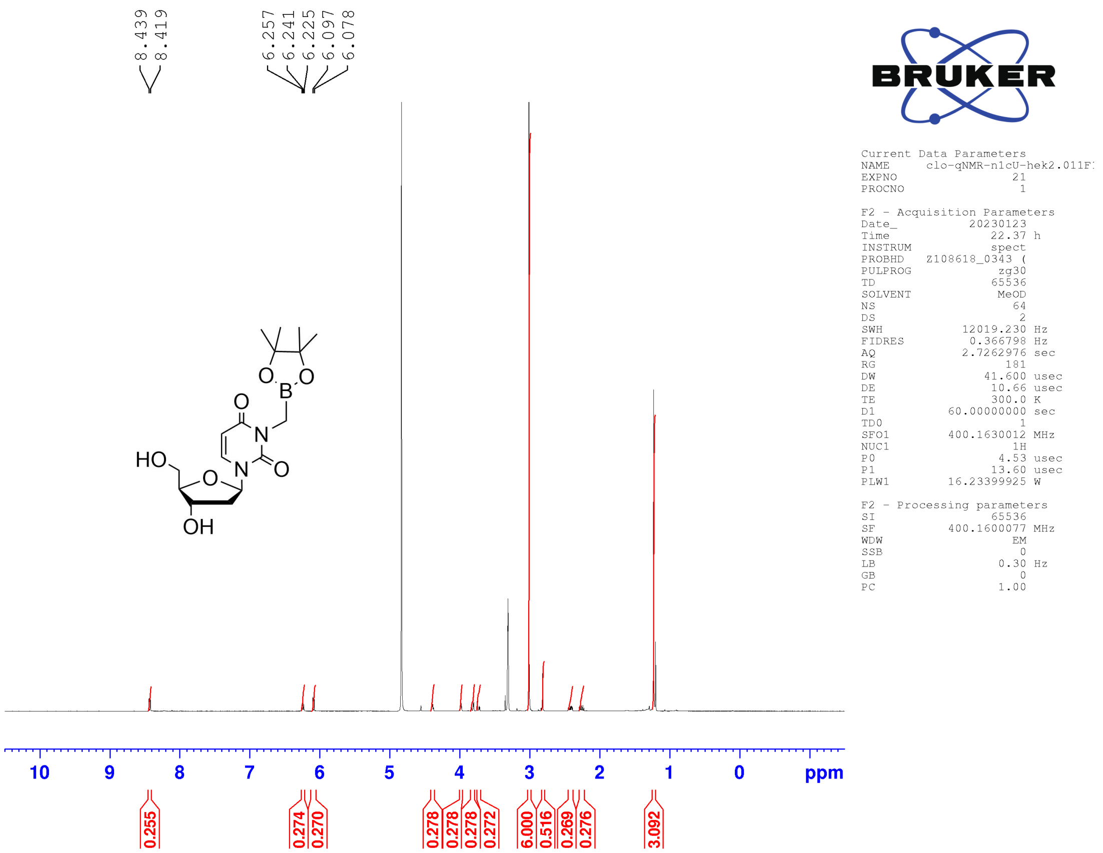

Figure S240 ^{1}H qNMR purity spectrum of **N1cU** with Me_2SO_2 (I.C) in CD_3OD. Purity 97.89%.

$$P_{\text{sample}} = \frac{S_{\text{sample}} \text{ x } N_{\text{std}} \text{ x } m_{\text{std}} \text{ x } M_{\text{sample}}}{S_{\text{std}} \text{ x } N_{\text{sample}} \text{ x } m_{\text{sample}} \text{ x } M_{\text{std}}} \text{ x } P_{\text{std}}$$

$$= \frac{0.274 \text{ x } 6 \text{ x } 2.1 \text{ mg x } 368.19 \text{ g mol}^{-1}}{6 \text{ x } 1 \text{ x } 2.3 \text{ mg x } 94.13 \text{ g mol}^{-1}} \text{ x } 99.96$$

$$= 97.89\%$$

S = Integrated area of the peak
N = Number of protons represented
m = Prepared mass
M = Molecular weight
P = Purity

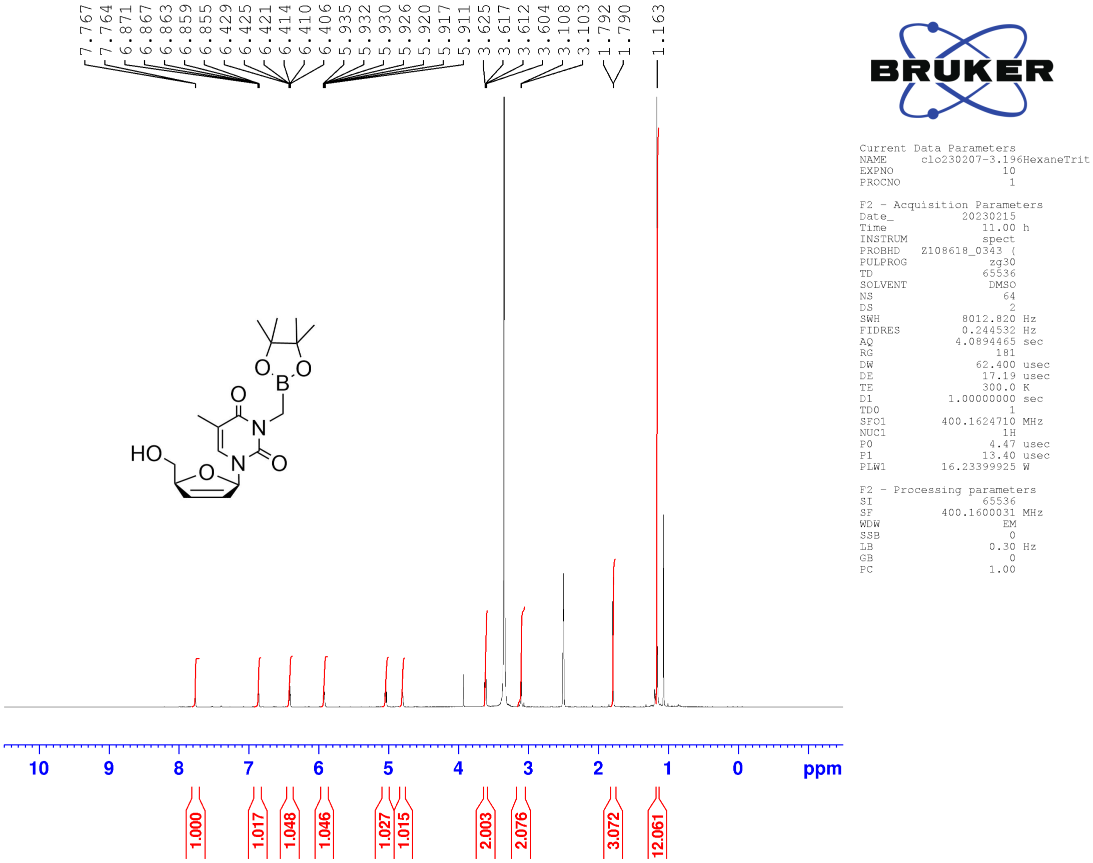

Figure S241 ^{1}HNMR spectra of **N1cS** in d6-DMSO.

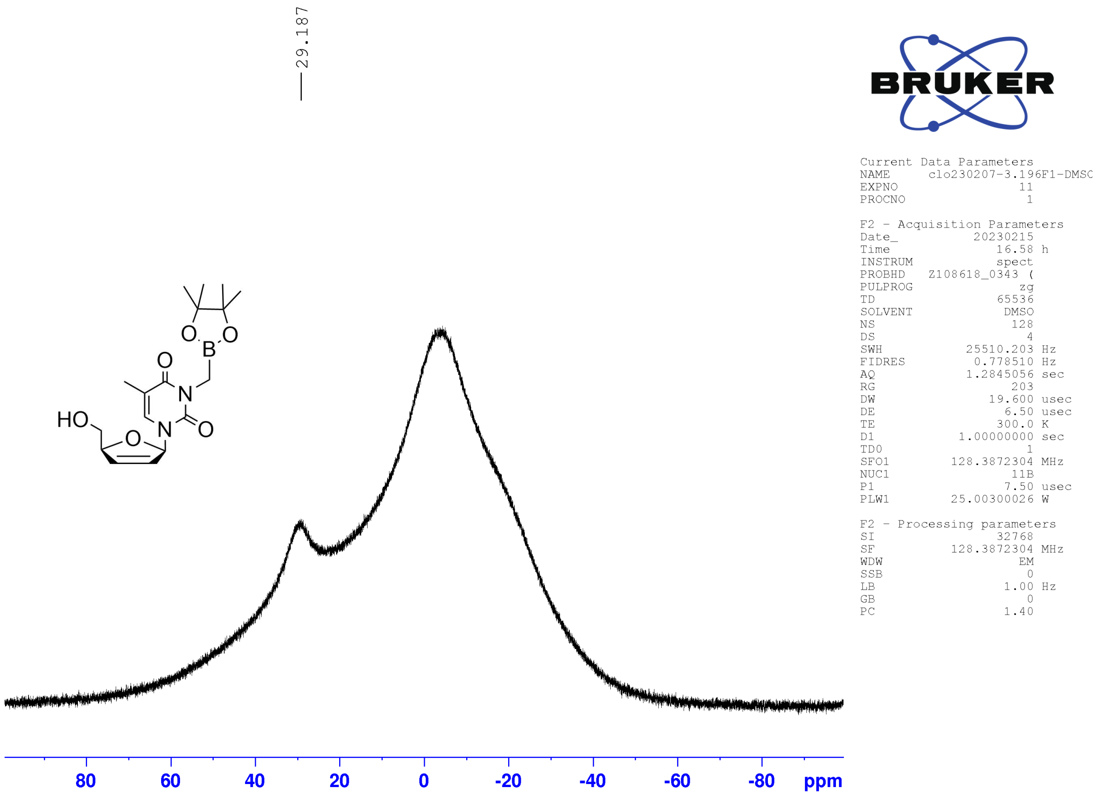

Figure S242 ^{11}BNMR spectra of **N1cS** in d6-DMSO.

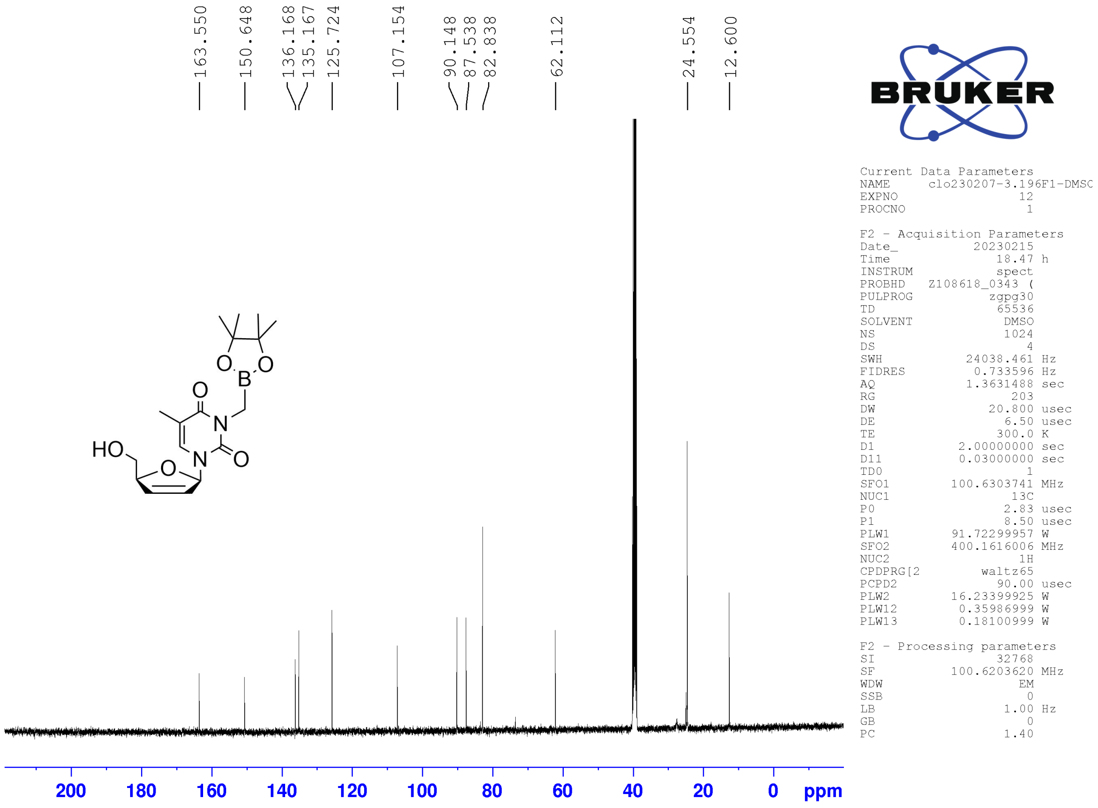

Figure S243 $^{13}C\{^{1}H\}$-NMR spectra of **N1cS** in d6-DMSO.

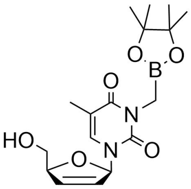

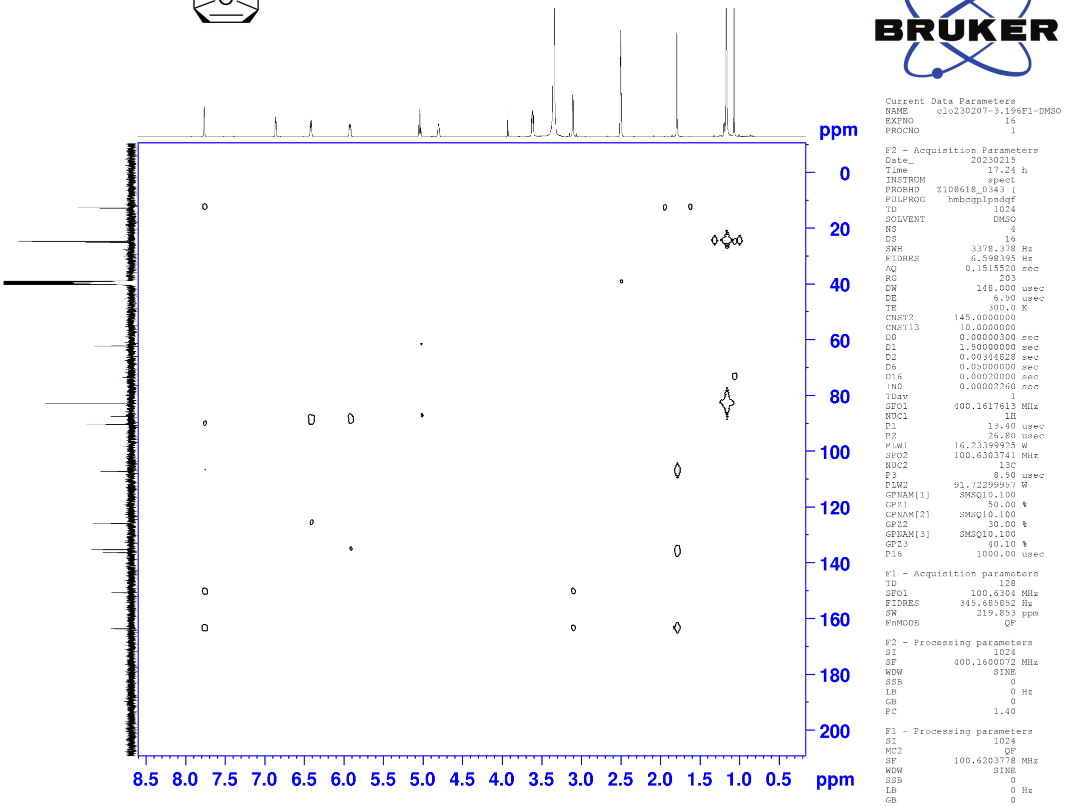

Figure S244 HMBC-NMR spectra of **N1cS** in CD_3OD.

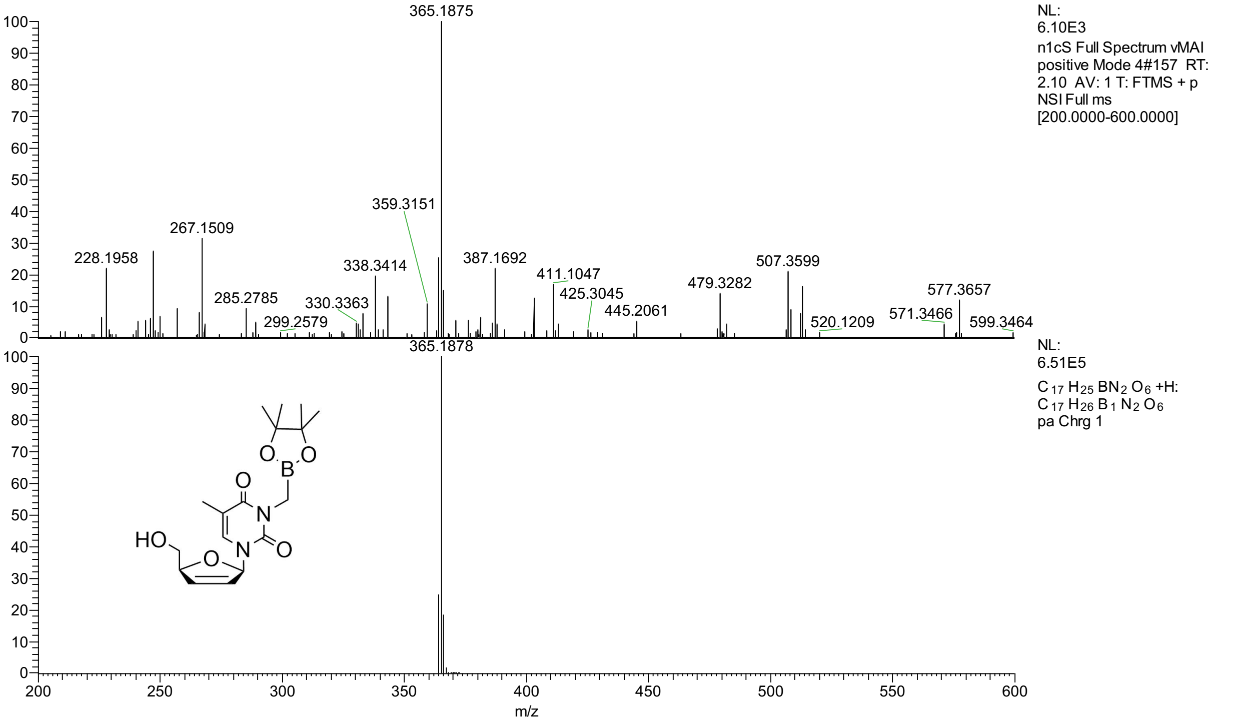

Figure S245 HRMS (ESI+) positive mode m/z calculated for **N1cS** [$C_{17}H_{26}BN_2O_6$] $[M+H]^+$ 365.1878, found 365.1875.

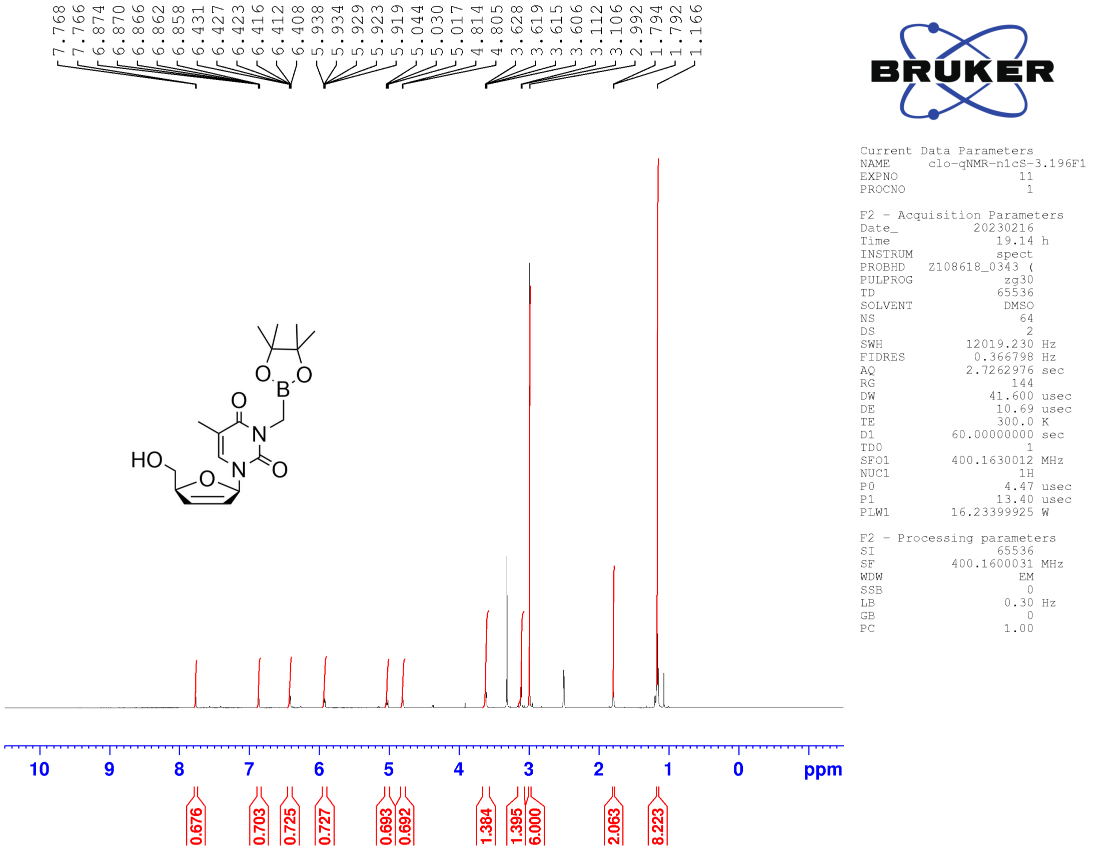

Figure S246 ^{1}H qNMR purity spectrum of **N1cS** with Me_2SO_2 (I.C) in d6-DMSO. Purity 95.52%.

$$P_{\text{sample}} = \frac{S_{\text{sample}} \text{ x } N_{\text{std}} \text{ x } m_{\text{std}} \text{ x } M_{\text{sample}}}{S_{\text{std}} \text{ x } N_{\text{sample}} \text{ x } m_{\text{sample}} \text{ x } M_{\text{std}}} \text{ x } P_{\text{std}}$$

$$= \frac{0.725 \text{ x } 6 \text{ x } 3.1 \text{ mg x } 364.21 \text{ g mol}^{-1}}{6 \text{ x } 1 \text{ x } 9.1 \text{ mg x } 94.13 \text{ g mol}^{-1}} \text{ x } 99.96$$

$$= 95.52\%$$

S = Integrated area of the peak
N = Number of protons represented
m = Prepared mass
M = Molecular weight
P = Purity

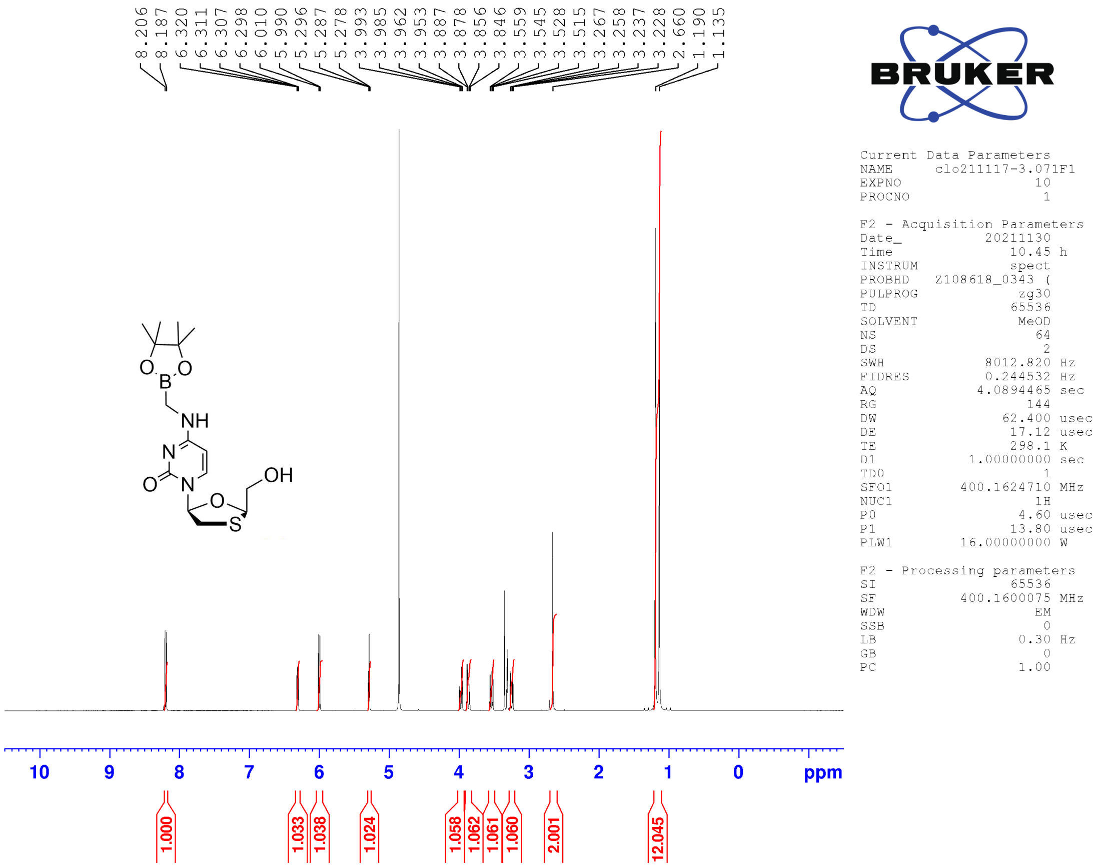

Figure S247 ^{1}HNMR spectra of **N1cL** in CD_3OD.

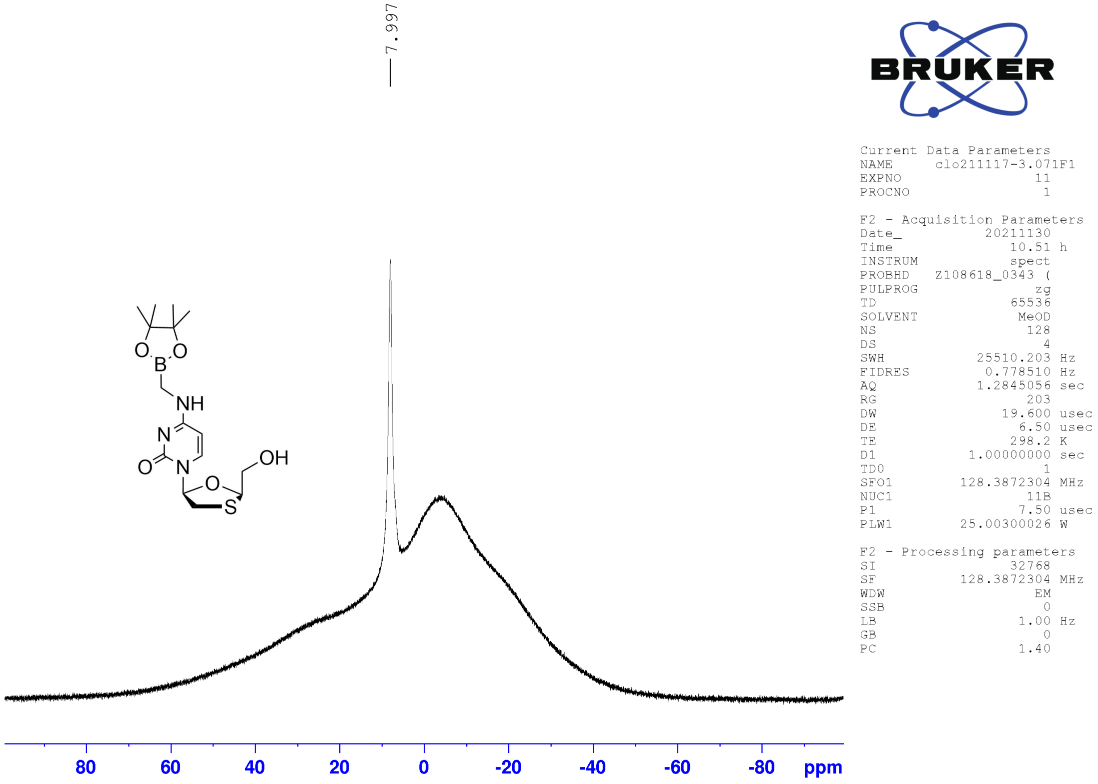

Figure S248 ^{11}BNMR spectra of **N1cL** in CD_3OD.

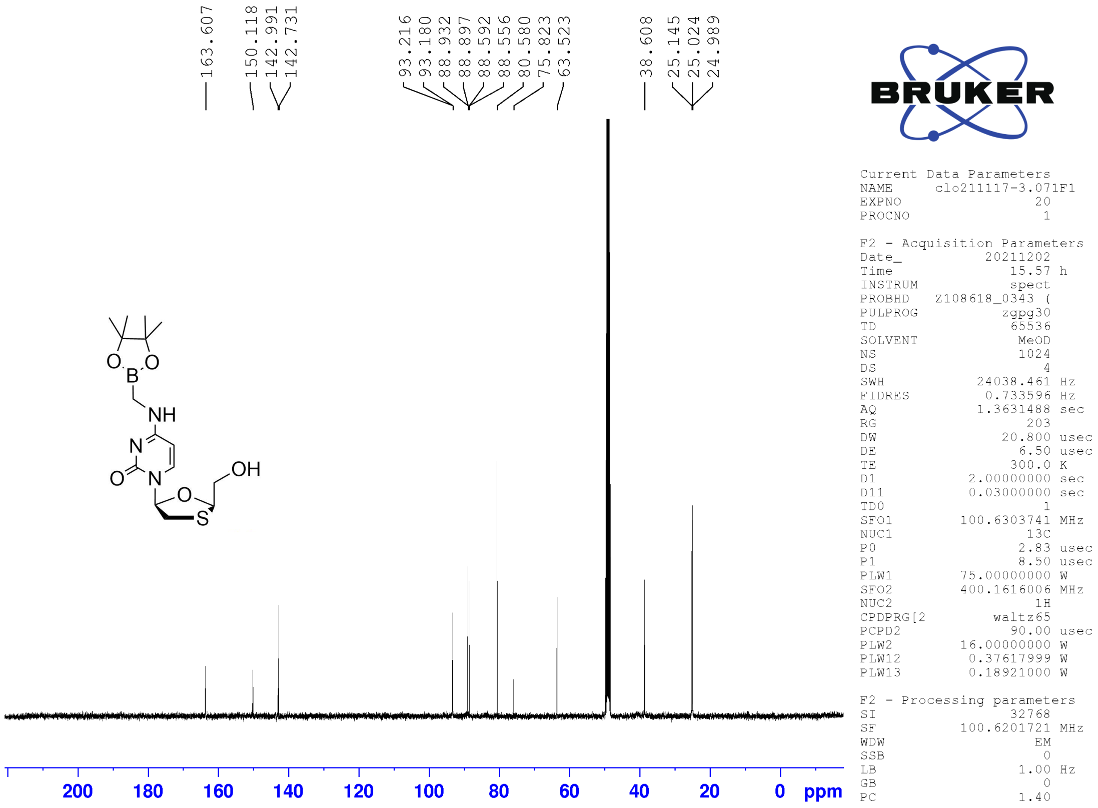

Figure S249 $^{13}C\{^{1}H\}$-NMR spectra of **N1cL** in CD_3OD.

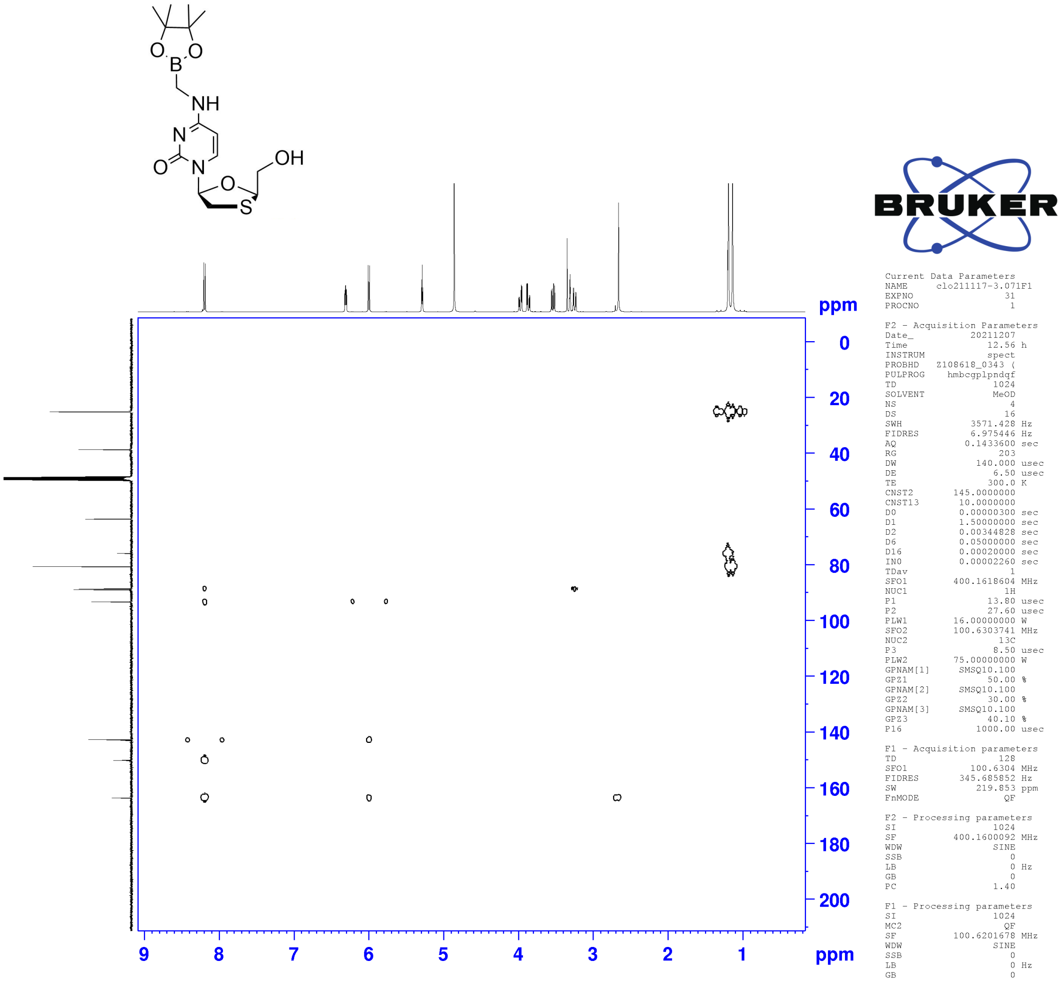

Figure S250 HMBC-NMR spectra of **N1cL** in CD_3OD.

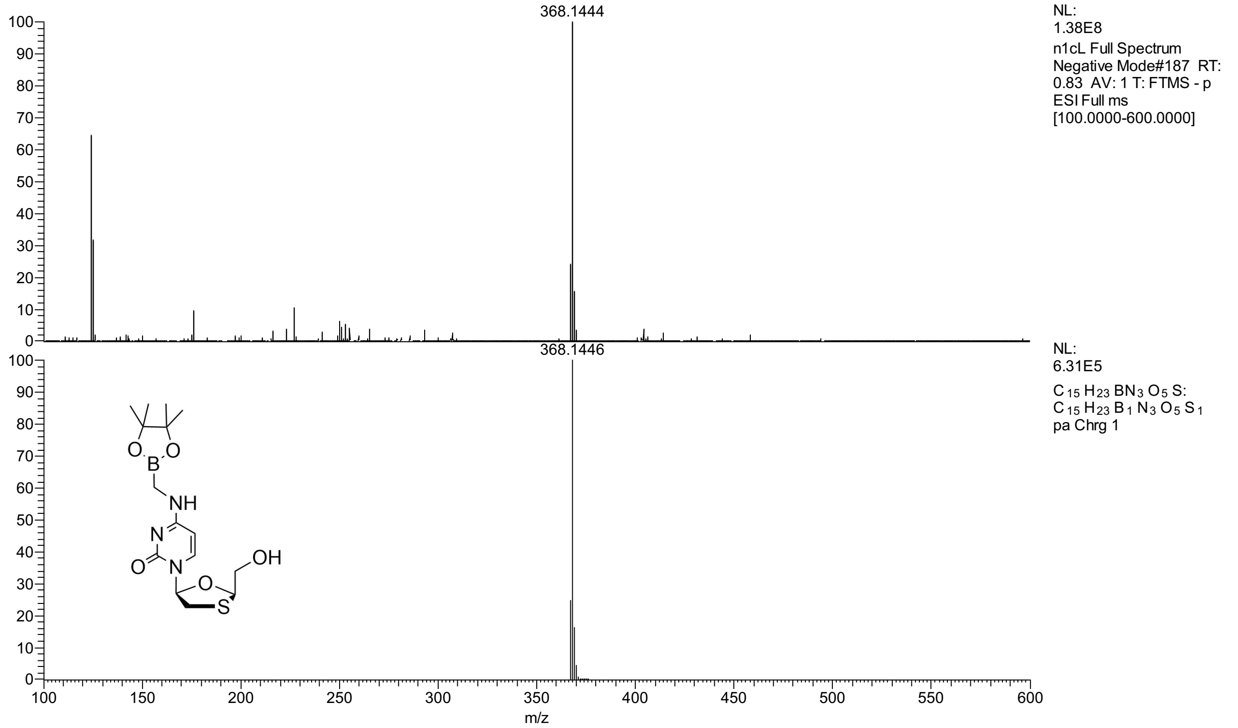

Figure S251 HRMS (ESI-) negative mode m/z calculated for **N1cL** [$C_{15}H_{23}BN_3O_5S$] [M-H]$^-$ 368.1446, found 368.1444.

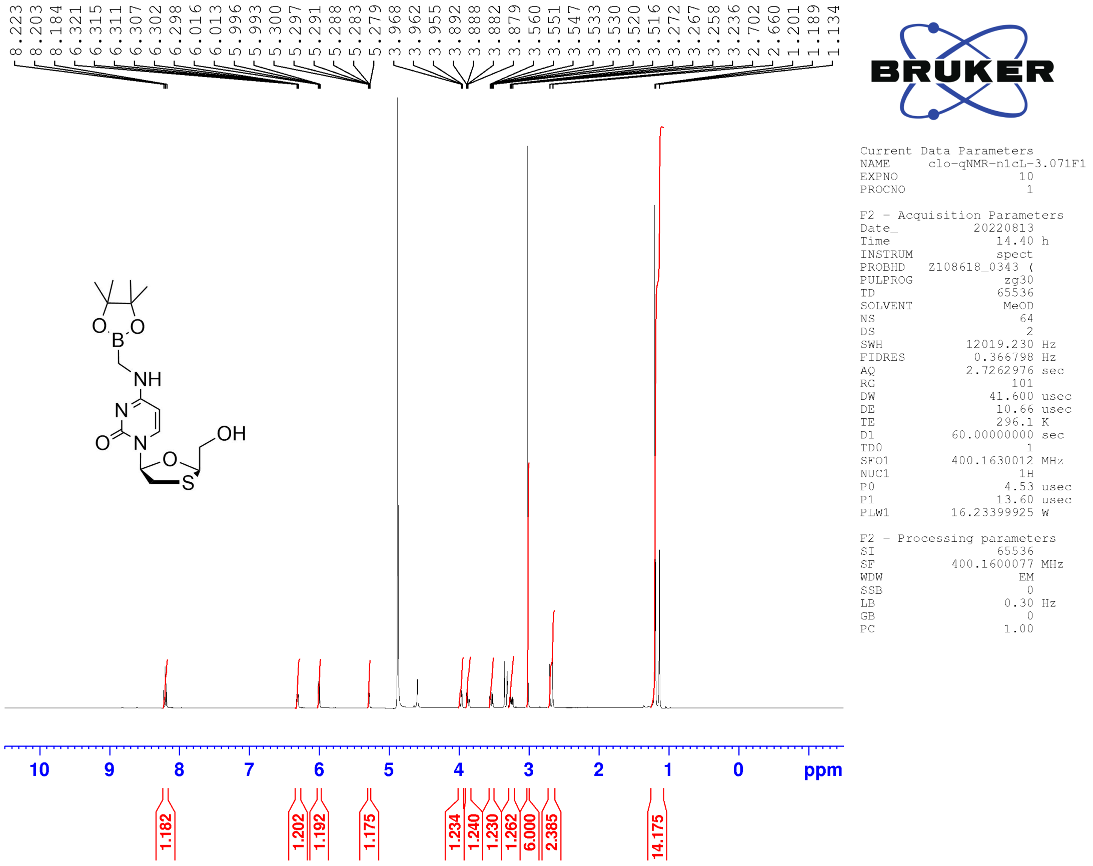

Figure S252 ^{1}H qNMR purity spectrum of **N1cL** with Me_2SO_2 (I.C) in CD_3OD. Purity 97.01%.

$$P_{\text{sample}} = \frac{S_{\text{sample}} \text{ x } N_{\text{std}} \text{ x } m_{\text{std}} \text{ x } M_{\text{sample}}}{S_{\text{std}} \text{ x } N_{\text{sample}} \text{ x } m_{\text{sample}} \text{ x } M_{\text{std}}} \text{ x } P_{\text{std}}$$

$$= \frac{1.192 \text{ x } 6 \text{ x } 4.4 \text{ mg x } 369.24 \text{ g mol}^{-1}}{6 \text{ x } 1 \text{ x } 21.2 \text{ mg x } 94.13 \text{ g mol}^{-1}} \text{ x } 99.96$$

$$= 97.01\%$$

S = Integrated area of the peak
N = Number of protons represented
m = Prepared mass
M = Molecular weight
P = Purity

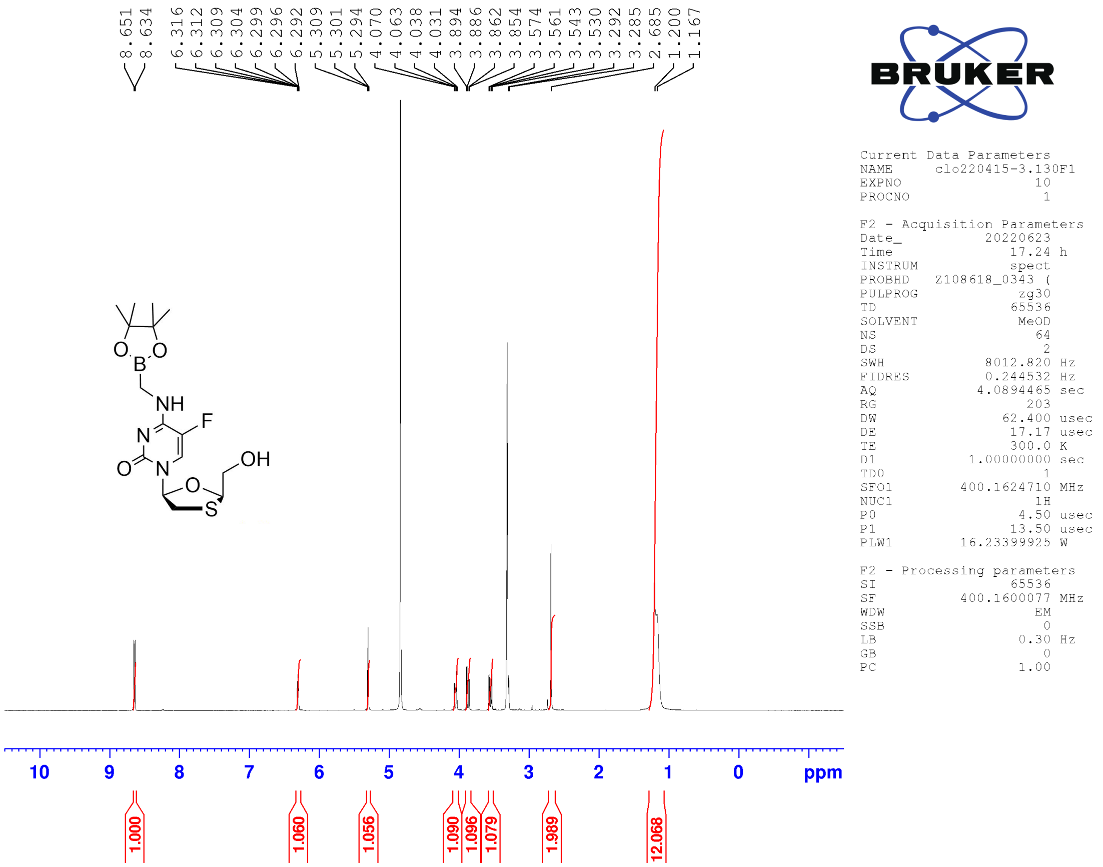

Figure S253 ^{1}HNMR spectra of **N1cE** in CD_3OD.

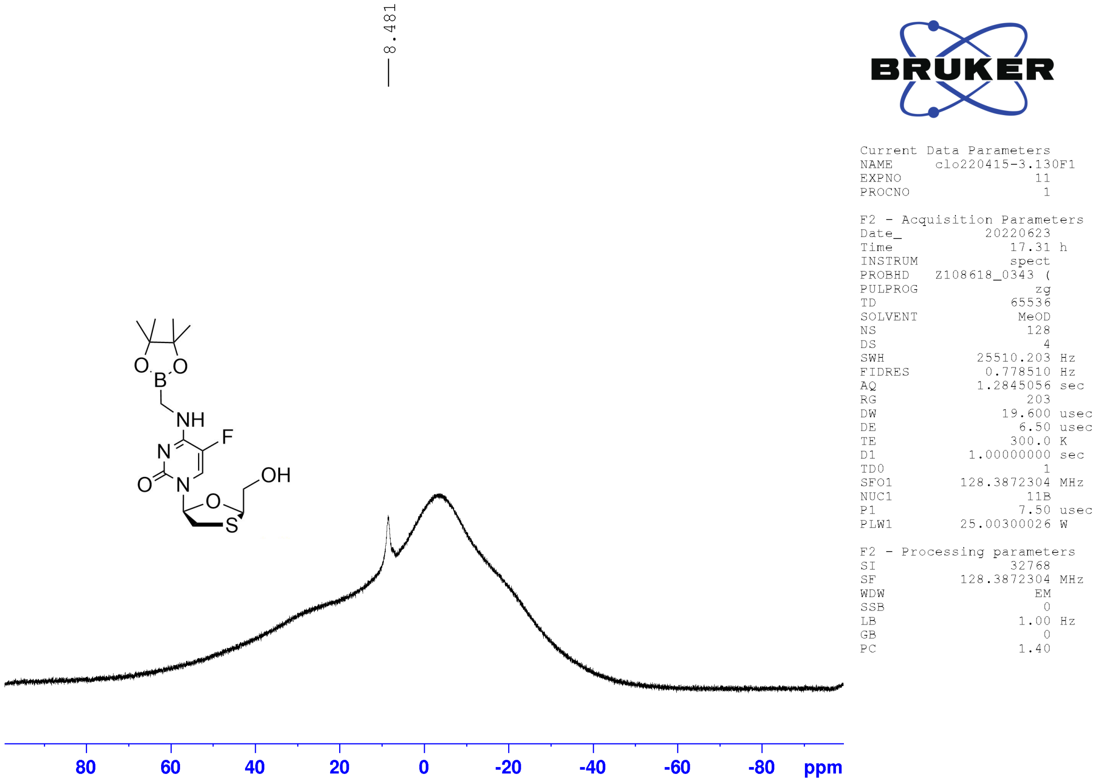

Figure S254 ^{11}BNMR spectra of **N1cE** in CD_3OD.

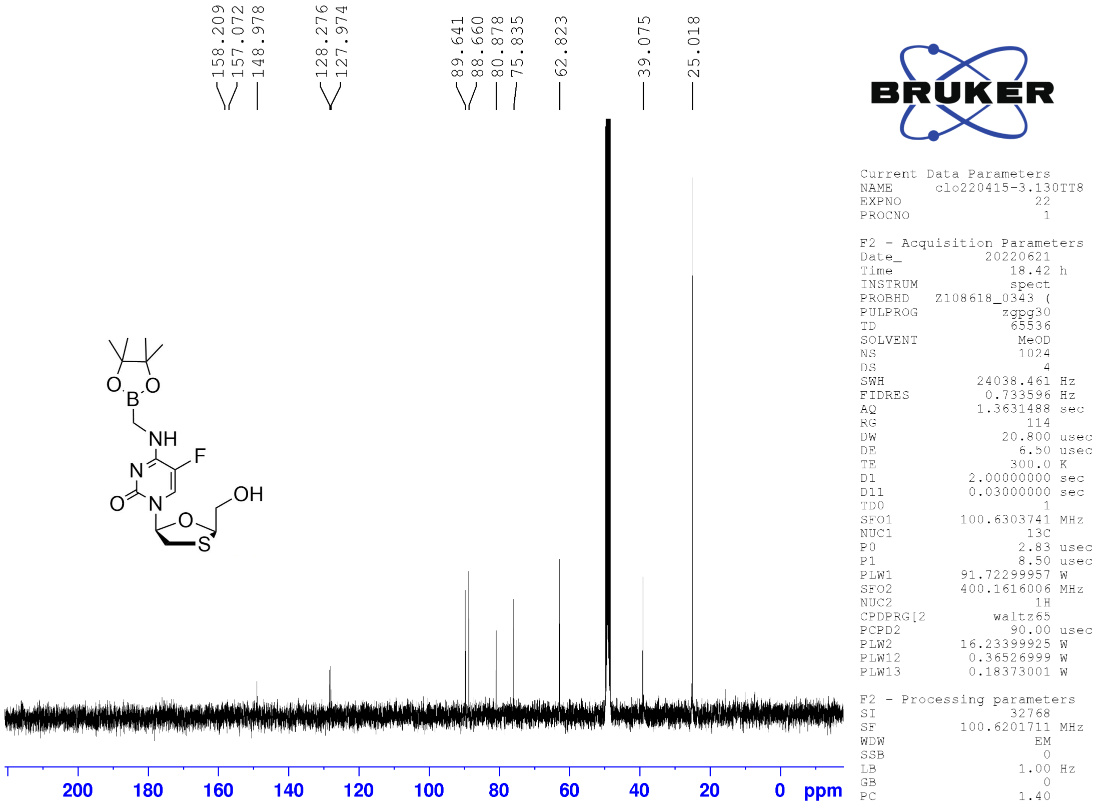

Figure S255 $^{13}C\{^{1}H\}$-NMR spectra of **N1cE** in CD_3OD.

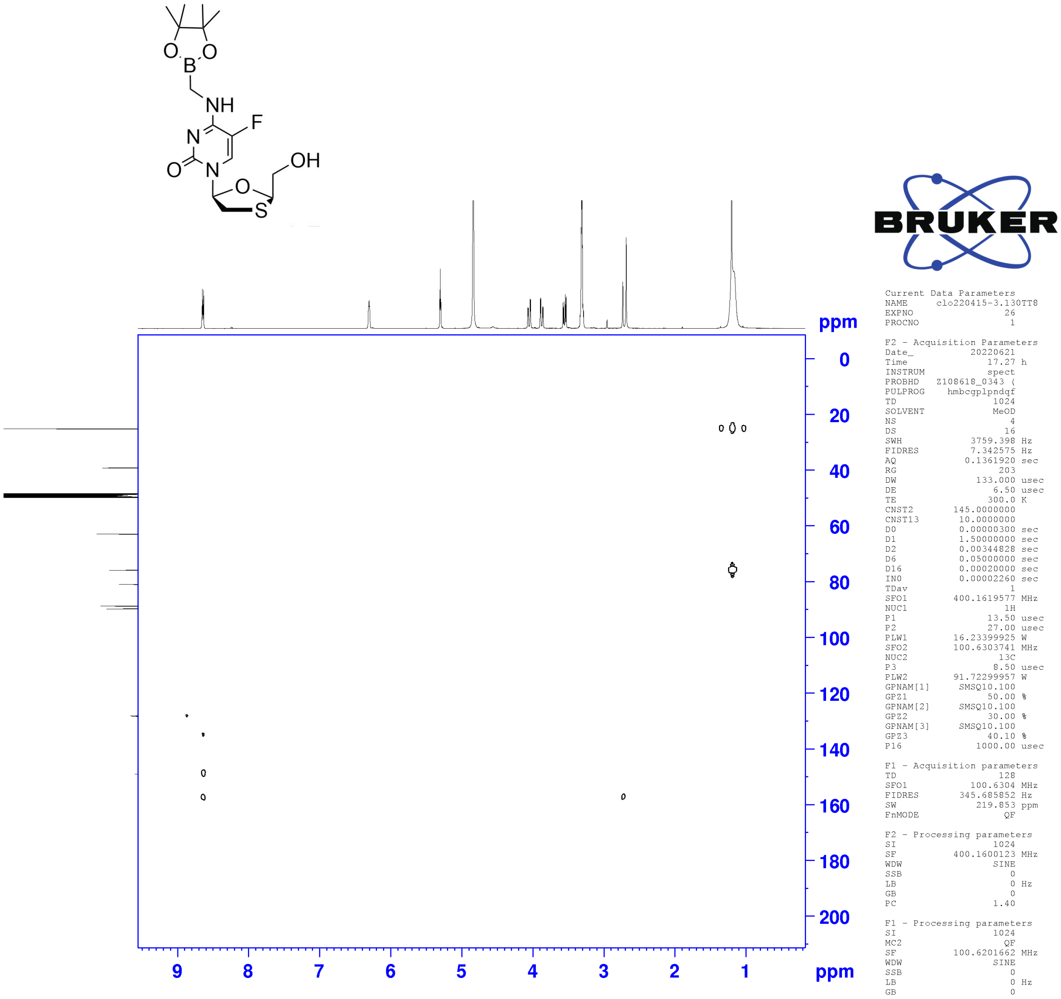

Figure S256 HMBC-NMR spectra of **N1cE** in CD_3OD.

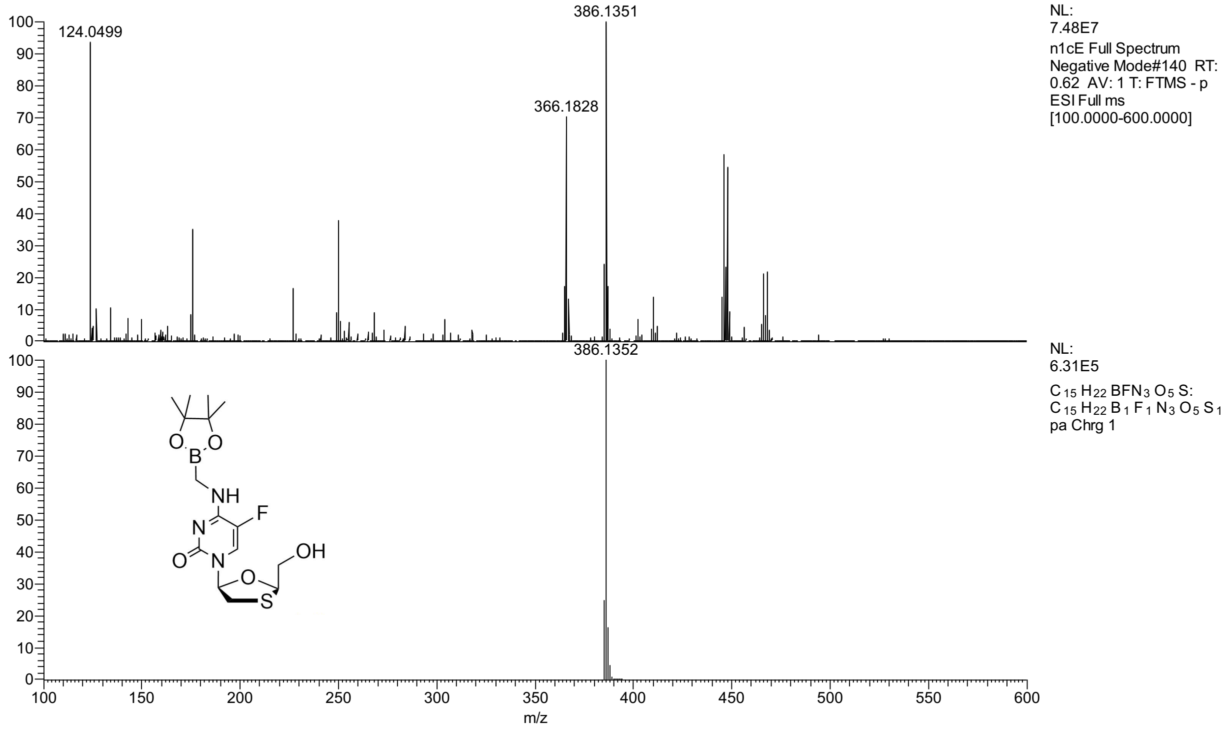

Figure S257 HRMS (ESI-) negative mode m/z calculated for **N1cE** [$C_{15}H_{22}BFN_3O_5S$] [M-H]$^-$ 386.1352, found 386.1351.

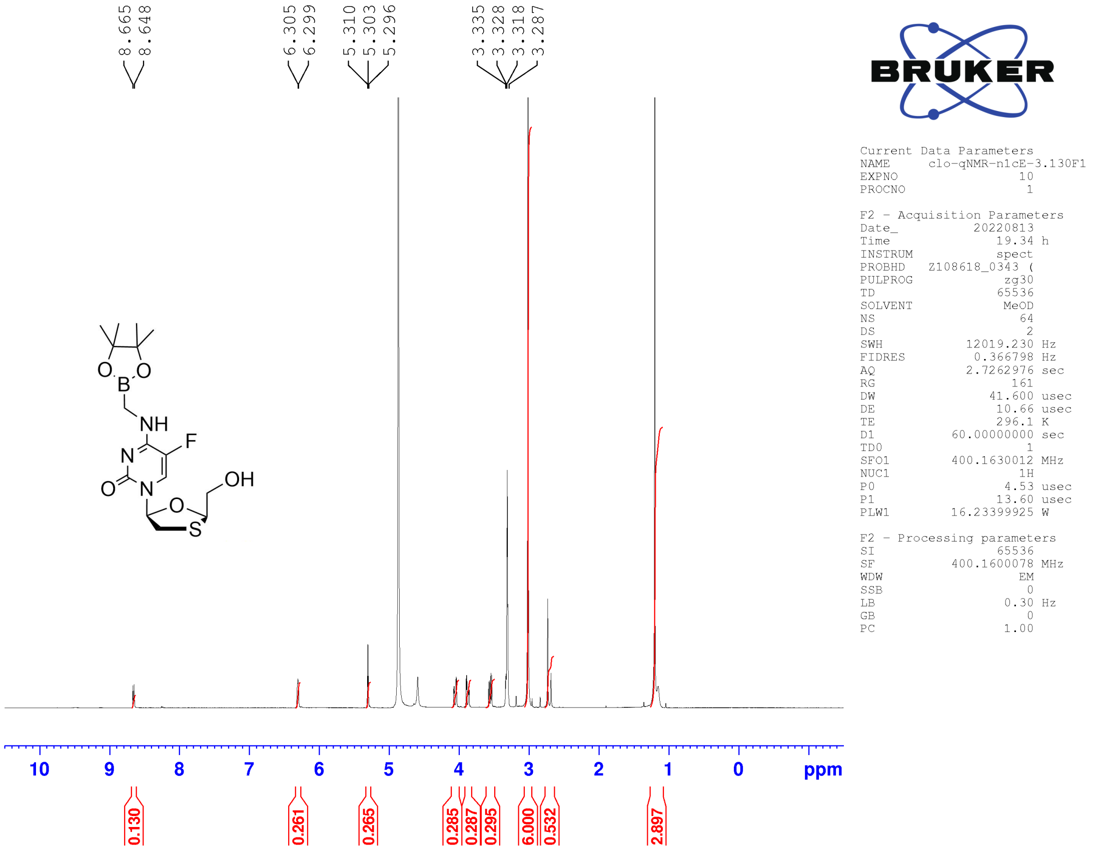

Figure S258 ^{1}H qNMR purity spectrum of **N1cE** with Me_2SO_2 (I.C) in CD_3OD. Purity 95.18%.

$$P_{\text{sample}} = \frac{S_{\text{sample}} \text{ x } N_{\text{std}} \text{ x } m_{\text{std}} \text{ x } M_{\text{sample}}}{S_{\text{std}} \text{ x } N_{\text{sample}} \text{ x } m_{\text{sample}} \text{ x } M_{\text{std}}} \text{ x } P_{\text{std}}$$

$$= \frac{0.261 \text{ x } 6 \text{ x } 4.7 \text{ mg x } 387.23 \text{ g mol}^{-1}}{6 \text{ x } 1 \text{ x } 5.3 \text{ mg x } 94.13 \text{ g mol}^{-1}} \text{ x } 99.96$$

$$= 95.18\%$$

S = Integrated area of the peak
N = Number of protons represented
m = Prepared mass
M = Molecular weight
P = Purity
